# *Helicobacter pylori* base-excision restriction enzyme in stomach carcinogenesis

**DOI:** 10.1093/pnasnexus/pgaf244

**Published:** 2025-08-05

**Authors:** Masaki Fukuyo, Noriko Takahashi, Katsuhiro Hanada, Ken Ishikawa, Česlovas Venclovas, Koji Yahara, Hideo Yonezawa, Takeshi Terabayashi, Yukako Katsura, Naoki Osada, Atsushi Kaneda, M Constanza Camargo, Charles S Rabkin, Ikuo Uchiyama, Takako Osaki, Ichizo Kobayashi

**Affiliations:** Department of Molecular Oncology, Graduate School of Medicine, Chiba University, Chiba 260-8670, Japan; Department of Infectious Diseases, Kyorin University School of Medicine, Mitaka, Tokyo 181-8611, Japan; Clinical Engineering Research Center, Faculty of Medicine, Oita University, Oita 879-5593, Japan; Department of Cell Biology, Institute of Life Science, Kurume University, Kurume, Fukuoka 830-0011, Japan; Institute of Biotechnology, Life Sciences Center, Vilnius University, Vilnius LT-10257, Lithuania; Antimicrobial Resistance Research Center, National Institute of Infectious Diseases, Tokyo 189-0002, Japan; Department of Infectious Diseases, Kyorin University School of Medicine, Mitaka, Tokyo 181-8611, Japan; Department of Microbiology, Tokyo Dental College, Tokyo 101-0061, Tokyo, Japan; Department of Pharmacology, Faculty of Medicine, Oita University, Yufu, Oita, Japan; Center for the Evolutionary Origins of Human Behavior, Kyoto University, Inuyama, Aichi 484-0081, Japan; Graduate School of Information Science and Technology, Hokkaido University, Sapporo 060-0814, Japan; Department of Molecular Oncology, Graduate School of Medicine, Chiba University, Chiba 260-8670, Japan; Health and Disease Omics Center, Chiba University, Chiba 260-8670, Japan; Division of Cancer Epidemiology and Genetics, National Cancer Institute, Rockville, MD 20878, USA; Division of Cancer Epidemiology and Genetics, National Cancer Institute, Rockville, MD 20878, USA; Laboratory of Genome Informatics, National Institute for Basic Biology, Okazaki 444-8585, Japan; Department of Infectious Diseases, Kyorin University School of Medicine, Mitaka, Tokyo 181-8611, Japan; Department of Infectious Diseases, Kyorin University School of Medicine, Mitaka, Tokyo 181-8611, Japan; Laboratory of Genome Informatics, National Institute for Basic Biology, Okazaki 444-8585, Japan; Research Institute for Micro-Nano Technology, Hosei University, Koganei, Tokyo 184-0003, Japan; Department of Medical Genome Sciences, University of Tokyo, Minato-ku, Tokyo 108-8639, Japan

**Keywords:** gastric cancer, genotoxin, restriction glycosylase, bacterial oncogenesis, PabI

## Abstract

Many recent lines of evidence from the human microbiome and other fields indicate bacterial involvement in various types of cancer. *Helicobacter pylori* has been recognized as the major cause of stomach cancer (gastric cancer), but the mechanism by which it destabilizes the human genome to cause cancer remains unclear. Our recent studies have identified a unique family of toxic restriction enzymes that excise a base (**A**: adenine) from their recognition sequence (5′-GT**A**C). At the resulting abasic sites (5′-GT**_**C), its inherent endonuclease activity or that of a separate endonuclease may yield atypical strand breaks that resist repair by ligation. Here, we present evidence demonstrating involvement of its *H. pylori* member, *Hp*PabI, in stomach carcinogenesis: (i) Association of intact *Hp*PabI gene with gastric cancer in the global *H. pylori* Genome Project and the open genomes; (ii) Frequent mutations at **A** in 5′-GT**A**C in the gastric cancer genomes as well as in *H. pylori* genomes; (iii) Its induction of chromosomal double-strand breaks in infected human cells and of mutagenesis in bacterial test systems. In addition, its unique regions that interact with DNA exhibit signs of diversifying selection. Our further analysis revealed similar oncogenic bacterium–restriction–enzyme pairs for other types of cancer. These results set another stage for cancer research and medicine around oncogenic restriction enzymes.

Significance Statement Recent works suggest bacterial involvement in many types of cancer. While *Helicobacter pylori*'s role in causing stomach cancer is established, the mechanisms underlying the oncogenic genome changes remain unclear. Several lines of evidence here show that its novel type of restriction enzyme, excising bases from a target sequence on DNA, causes stomach cancer: (i) Association of its gene with occurrence of stomach cancer in the global *H. pylori* Genome Project; (ii) frequent mutation of its recognition sequence in the stomach cancer genomes; (iii) experimental demonstration of its chromosome breakage and mutagenesis. Similar restriction enzyme–bacterium pairs are identified in other types of cancer. These results will start a new stage in cancer study and medicine featuring bacterial oncogenic restriction enzymes.

## Introduction

*Helicobacter pylori* infection has consistently emerged as the most important etiological factor for gastric adenocarcinoma (1). This Gram-negative bacterium resides in the stomachs of about half of the human population since early childhood. In addition to several other known and potential virulence factors, recent comparisons of hundreds of *H. pylori* genomes have identified several genes (genotypes) associated with gastric cancer (2–4).

The mechanisms by which *H. pylori* infection destabilizes the human genome and induces cancer remain unclear. *Helicobacter pylori* has been shown to induce mutations in mice (5). The gastric cancer genomes exhibit characteristic mutations (6), some of which bear signatures associated with specific mutational processes (7). These include single-base substitution (SBS)1 (deamination of 5-methylcytosine), SBS2 and SBS13 (APOBEC overactivity), SBS3 (homologous recombination defect) (8 ), and SBS18 (reactive oxygen species [ROS] and DNA damage). Several bacterial “genotoxins” have been implicated in host genome damage (9). These include the cytolethal distending toxins, the typhoid toxin, and colibactin. Importantly, these genotoxins are associated with carcinogenesis (10, 11), with colibactin, for instance, being linked to mutational signatures observed in colorectal cancer (12). The potential presence of comparable carcinogenic genotoxins in *H. pylori* has not been addressed.

*Helicobacter pylori* infection of human cells causes chromosomal double-strand breakage (13), which is partially dependent on CagA, a virulence factor (14). Other pathways of DNA breakage and mutagenesis may involve ROS damage and structure-specific nucleases (15). Furthermore, *H. pylori* infection leads to the accumulation of abasic sites (apurinic/apyrimidinic sites, AP sites) (16). The AP sites that arise spontaneously or via base excision by DNA glycosylases are well-established premutagenic lesions, which can also be converted into strand breaks by the action of AP endonucleases (17).

**Fig. 1.**
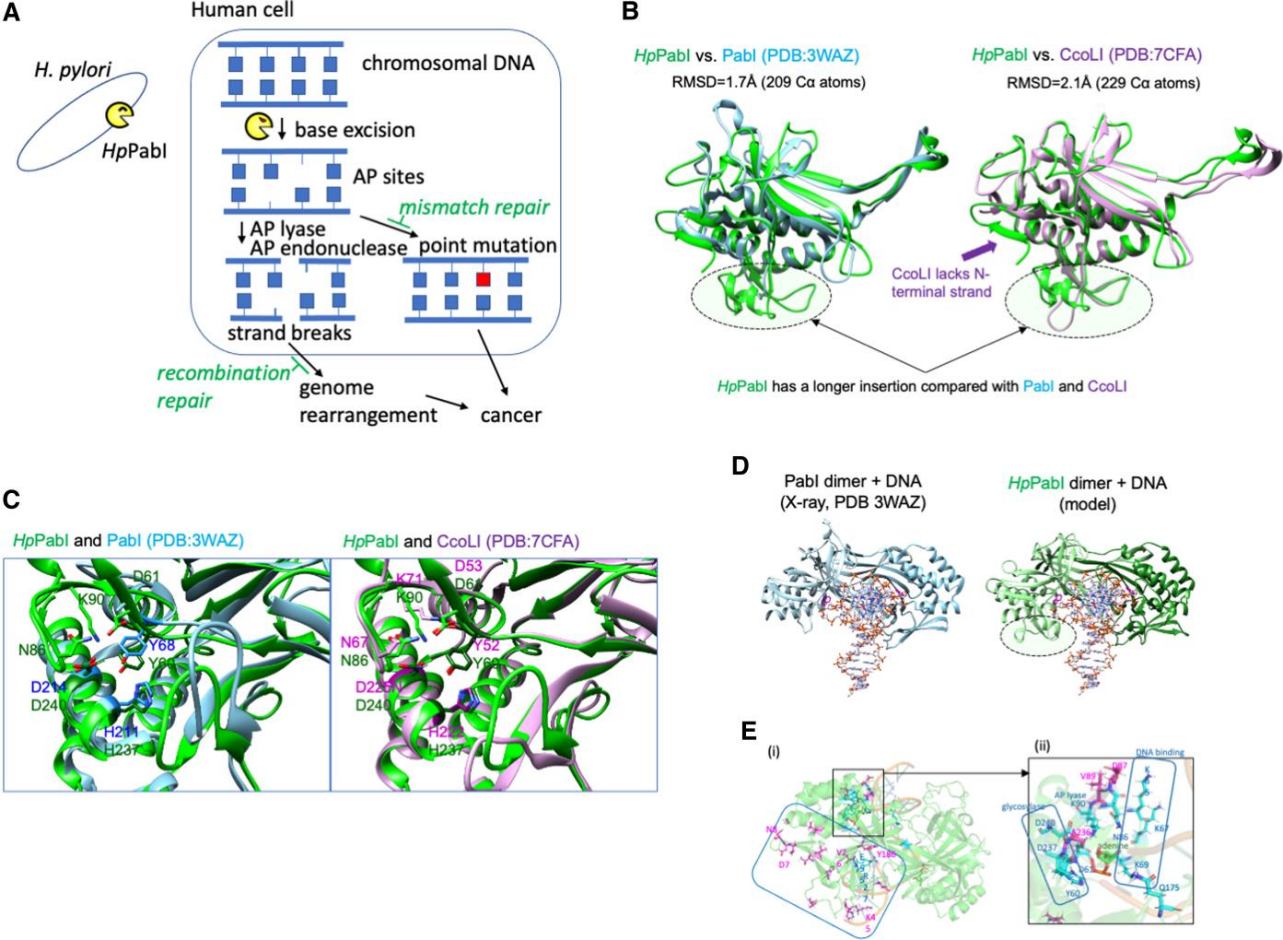
Function and structure of *Hp*PabI. A) Proposed role in stomach carcinogenesis. The base-excision restriction enzyme from *H. pylori*, *Hp*PabI, may play a role in stomach carcinogenesis. *Helicobacter pylori,* through an unidentified process, transfers this restriction glycosylase to human cells where it targets and excises the adenine base at its palindrome recognition sequence, 5′-GTAC, in human chromosomes. This excision creates an AP (abasic) site that can lead to point mutagenesis although the mismatch repair reactions can inhibit this process. The subsequent action of either the AP lyase activity of *Hp*PabI itself or the human AP endonuclease (APE1) on the AP site can cause DNA double-strand breaks. These breaks may contribute to genome rearrangements unless repaired by homologous recombination. The mutations and rearrangements can result in gastric cancer. B) Structural models of *Hp*PabI. (i) Overlay of the *Hp*PabI monomer model with PabI from *Pyrococcus abyssi*. (ii) Overlay of the *Hp*PabI monomer model with CcoLI from *Campylobacter coli*. C) Comparison of the active sites. D) Comparison of the dimers on DNA. (i) PabI dimer in complex with DNA (PDB: 3WAZ). (ii) Predictive model of the *Hp*PabI dimer bound to DNA. The unique insertion absent from PabI is highlighted by an ellipse on one monomer. E) High d*N*/d*S* sites on the *Hp*PabI-DNA model. The sites are represented as follows: the magenta sticks indicate residues with d*N*/d*S* > 1.5; the cyan sticks represent residues with defined functions (Table S7). The larger square in (i) emphasizes a broad cluster, encompassing DNA binding sites, an *H. pylori*-specific N-terminal beta-sheet, and adjacent regions. The square enlarged in (ii) highlights a dense cluster covering active site residues and DNA-binding residues (19).

Recent studies have elucidated a unique family of restriction enzymes, the PabI family of *restriction glycosylases* (18) (Fig. 1A), present in *H. pylori* and certain prokaryotes (20), that excise a specific base (A) from their recognition sequence, 5′-GTAC, unless methylated to 5′-GTmAC (21–23). At the resulting AP site, the enzyme's own AP lyase activity or AP endonucleases on separate molecules generate strand breaks with atypical structures (Fig. 1A) evading straightforward religation repair mechanisms (22, 23). The potentially harmful effects of these PabI family restriction glycosylases have been further substantiated by evolutionary analyses (24). Notably, Helicobacter genomes exhibit a conspicuous avoidance of their recognition sequence, 5′-GTAC [REBASE, http://tools.neb.com/∼vincze/cutfreq/GTAC.html], and the gene encoding this family is frequently found decayed in *H. pylori* strains (24, 25).

Based on the above observations, we hypothesize that the *H. pylori* enzyme *Hp*PabI generates AP sites at its recognition sequence within the human genome. We assume that, subsequently, these AP sites give rise to point mutations and genome-rearranging strand breaks, which initiate and promote cancer (18) (Fig. 1A). In this study, we substantiated this hypothesis through multiple lines of evidence.

## Results

### Association of *Hp*PabI gene with gastric cancer

As part of the *Helicobacter pylori* Genome Project (*Hp*GP) (26), *H. pylori* strains from patients with gastric cancer and from people without gastric cancer were chosen from 50 countries worldwide, and their whole genome sequences (∼1,100 in the entire set) were decoded (26). These genomes were divided into populations through a step-wise Principal Component Analysis followed by Uniform Manifold Approximation and Projection (PCA-UMAP) (Materials and methods). They were first divided into five major groups including EU (primarily from Northern and Eastern Europe) and LA (primarily from Southern Europe and Latin America). Each group was further divided by PCA-UMAP (Dataset 1A, 991 strains).

We assessed the presence or absence of the intact *Hp*PabI gene in these complete *H. pylori* genomes using homology-search-based methods (Materials and methods). (For their amino acid sequences, refer to Fig. S1.) Subsequently, we determined the odds ratio for the presence of the *Hp*PabI gene to cancer risk in each group and each subgroup (Materials and methods) and listed cases with enrichment (lower limit of the 95% CI > 1) (Table 1). We obtained a notable odds ratio of 2 for the EU group and 3 for its two overlapping subgroups, EU-1_3 (first subgroup when divided into three) and EU-3_5 (Tables 1 and S1, Fig. S2A). Intriguingly, when considering only *cagA*-positive strains, the odds ratio rose to ∼4 in these subgroups and was over 9 in another subgroup (EU-3_6), the latter containing strains predominantly from Sweden (Dataset 1C). These three subgroups overlap (Fig. S2A). The highest odds ratio of 9.9 was recorded for a subgroup (LA-6_7) of the LA group, comprising most strains from Spain and Portugal (Tables 1 and S1, Fig. S2B, Dataset 1C).

**Table 1.** Association between HpPabI and gastric cancer in *H. pylori* clusters.

Genomes	*cagA*	Cluster	*Hp*PabI^+^, cancer	*Hp*PabI^+^, noncancer	*Hp*PabI^−^, cancer	*Hp*PabI^−^, noncancer	Odds ratio (95% CI)		*Hp*PabI^+^ fraction
*Hp*GP only									
		All	146	480	63	240	1.2 (0.83 to 1.6)		0.67
	*cagA* ^+/−^								
		EU	51	138	15	83	2.0 (1.1 to 3.9)		0.66
		EU-1_3	33	50	6	28	3.1 (1.2 to 8.3)		0.71
		EU-3_5	33	41	6	24	3.2 (1.2 to 8.8)		0.71
	*cagA* ^+^								
		EU-1_3	31	39	5	23	3.7 (1.3 to 10)		0.71
		EU-3_5	31	36	5	22	3.8 (1.3 to 11)		0.71
		EU-3_6	12	14	0	11	(9.4) (1.1 to 84)		0.68
		LA-6_7	9	10	1	11	9.9 (1.1 to 93)		0.61
*Hp*GP + NCBI									
		All	187	708	112	496	1.2 (0.9 to 1.5)		0.60
	*cagA* ^+/−^								
		EU	68	222	22	179	2.5 (1.5 to 4.2)		0.59
		EU-1/2	56	134	17	82	2.0 (1.1 to 3.7)		0.66
		EU-1/3	38	82	10	52	2.4 (1.1 to 5.3)		0.66
		EU-2/3	6	32	0	53	(9.9) (1.1 to 86)		0.41
		EU-1/4	38	70	10	44	2.4 (1.1 to 5.3)		0.67
		EU-5/7	21	31	3	22	5.0 (1.3 to 19)		0.68
		EU-7/8	20	28	3	20	4.8 (1.2 to 18)		0.68
		EU-13/13	9	12	1	15	11 (1.3 to 100)		0.57
	*cagA* ^+^								
		EU	60	158	17	115	2.6 (1.4 to 4.6)		0.62
		EU-1/2	51	102	15	59	2.0 (1.0 to 3.8)		0.67
		EU-1/3	35	68	8	41	2.6 (1.1 to 6.2)		0.68
		EU-1/4	35	61	8	35	2.5 (1.1 to 6.0)		0.69
		EU-5/7	20	25	2	18	7.2 (1.5 to 35)		0.69
		EU-7/8	20	23	2	17	7.4 (1.5 to 36)		0.69
		EU-13/13	9	10	1	14	13 (1.4 to 120)		0.56

Reinforcing these associations between the presence of the *Hp*PabI gene and cancer risk, additional 2,250 genomes available in NCBI were integrated into our study (Materials and methods). The combined genomes were divided into five major groups and then each group was further divided in a stepwise way (Dataset 1B, 2,357 strains). The odds ratio was over 2 in the EU group for *cagA*^+/−^ (Table 1). It was over two in many EU subgroups and the highest in two nonoverlapping EU subgroups, EU-13/13 (11) and EU-2/3 (>9.9) (Fig. S2C). In EU-13/13, it was 13 for *cagA*^+^ genomes (Table 1). The positive association between *Hp*PabI and gastric cancer was not observed in *cagA*^−^ or East Asian type *cagA* genomes. However, with Western-type *cagA*, there was high *Hp*PabI association in the same EU/LA populations (Fig. S3). Taken together, these findings suggest that the presence of the *Hp*PabI gene is associated with an increased risk of gastric cancer, particularly in many European clusters. Why does this positive association manifest only in individuals from particular regions? Possible underlying factors include the *H. pylori* genotype (26), human genotype, environmental factors, and region-specific criteria for cancer diagnosis.

### Preferential mutation at the *Hp*PabI recognition site 5-GTAC in gastric cancer genomes

**Fig. 2:**
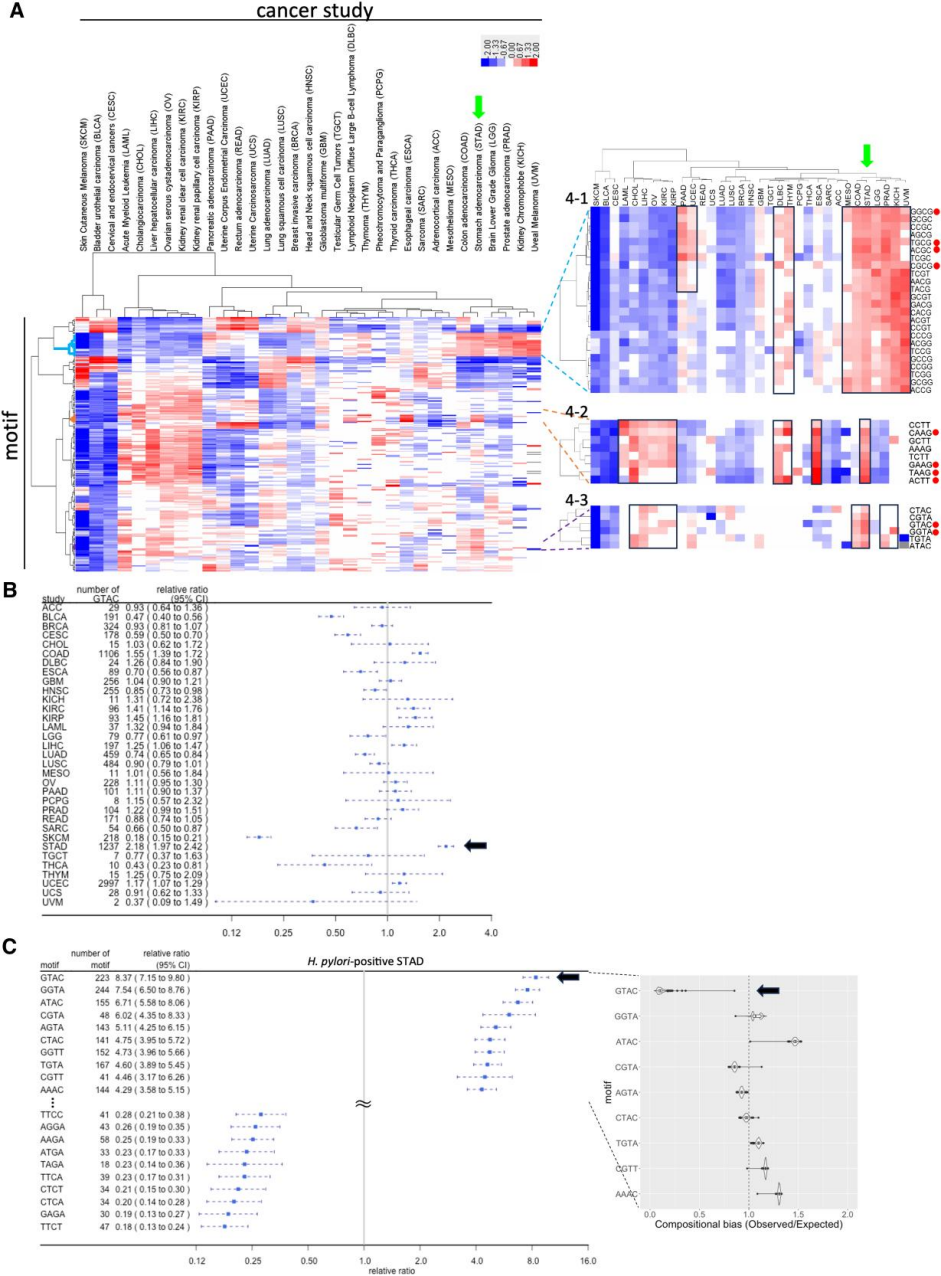
Distinctive 4-mer mutation motifs in cancer genomes. A) 2D hierarchical clustering of 4-mer mutation motifs across diverse cancer genomes as deduced from TCGA dataset. In the expanded section on the right, the 10 highest-ranked motifs by relative ratio in STAD are highlighted with dots. B) 5′-GTAC mutation motif in cancer studies. A forest plot showing the relative ratios of the 5′-GTAC mutation motif across various cancer studies. STAD is indicated with an arrow. c) Frequent/infrequent mutation motifs in *H. pylori*-positive STAD. Forest plot visualizing the top 10 and bottom 10 motifs ranked by relative ratio. The relative ratio is represented by a blue dot, and the 95% CI is represented as a whisker. Shown in the expanded section on the right is a Violine plot depicting the compositional bias in *H. pylori* genomes for the top 10 motifs. Center line represents the median; the edges of the box denote the upper and lower quartiles; individual points show outliers.

If *Hp*PabI causes mutations that lead to cancer, it may likely target its recognition site, 5′-GTAC in gastric cancer genomes for mutagenesis. To investigate this hypothesis, we conducted an extensive analysis of k-mer (*k* = 3, 4, and 5) mutation motifs, examining mutations across all cancer genomes available in The Cancer Genome Atlas (TCGA) (https://www.cancer.gov/tcga). Our analysis encompassed 1,413,287 mutations across 7,220 samples, distributed across 28 distinct cancer disease cohorts within TCGA. Single nucleotide polymorphisms (SNPs), synonymous to single base-pair substitutions in TCGA, accounted for most mutations (82–99%) across various types of cancer. We quantified the relative ratio of each mutation motif's frequency within each cancer and made a two-dimensional (cancer type × mutation motif) hierarchical clustering (as in Fig. 2A).

Initially, we focused on 3-mer mutation motifs, which encompassed 32 (=4^3^/2) distinct classes comprising a mutated base, along with the 5′-flanking base and the 3′-flanking base (Figs. S4 and S5). None of the mutation motifs exhibited exceptional specificity for stomach adenocarcinoma (STAD). Notably, the CGC mutation motif, potentially reflecting C-to-T mutations within the GCG mutation motif (as observed in SBS1; (7)), and the AAG mutation motif, possibly corresponding to T to G mutations within the CTT mutation motif (as seen in SBS17b; (7)), displayed the highest relative ratios of 1.96 and 1.95, respectively. (Note that GCG is consolidated with its reverse complement CGC and that CTT is consolidated with AAG [Materials and methods, Fig. S5B]). Subsequently, we analyzed the 5-mer mutation motifs, which consisted of 512 (4^5^/2) classes comprising 3-mer mutation motifs, along with the 5′-flanking base and 3′-flanking base (Figs. S6B and S7A). The top 20 mutation motifs, ranked by their relative ratio in STAD, were further categorized into five distinct groups (Fig. S7A, right). Notably, most mutation motifs within the top 20 and bottom 20 shared common 4-mers, underscoring the critical role of 4-mer mutation motifs in mutation patterns (Fig. S7B).

We then examined 4-mer mutation motifs, encompassing 256 (4^4^) distinct classes derived from the fusion of 3-mer mutation motifs with their respective 5′-flanking base (with the mutation at the third base pair). The top 10 mutation motifs, as ranked by their relative ratios in STAD, were categorized into three groups: 4-1, 4-2, and 4-3 (Fig. 2A). Among them, GT***A***C (***A*** as the site of substitution), the recognition sequence of *Hp*PabI (*A* as the site of base excision), notably exhibited a high ratio of 2.2 in STAD (Fig. 2B). In *H. pylori*-positive cases in STAD, GTAC showed the highest relative ratio of 8.4 (Fig. 2C). (Refer to Fig. S8 for *H. pylori*-negative cases. We remember the difficulty in judging *H. pylori* positivity as a limitation of these studies.) GGTA (with the second highest ratio of 7.5), CG***T***A, AG***T***A, and TG***T***A in the top 10, collectively represented as NG***T***A, match the first four nucleotides of the reverse complement sequences of the 5-mer GT***A***CN (refer to 5-mer analysis in Fig. S7), which includes GT*A*C.

GTAC stands out as significantly underrepresented in the *H. pylori* genome, exhibiting a compositional bias of 0.11 (Fig. 2C, right panel). This suggests a strong selection against GTAC by the toxic *Hp*PabI in *H. pylori*. We did not observe a similar decrease in the remaining mutation motifs.

**Fig. 3.**
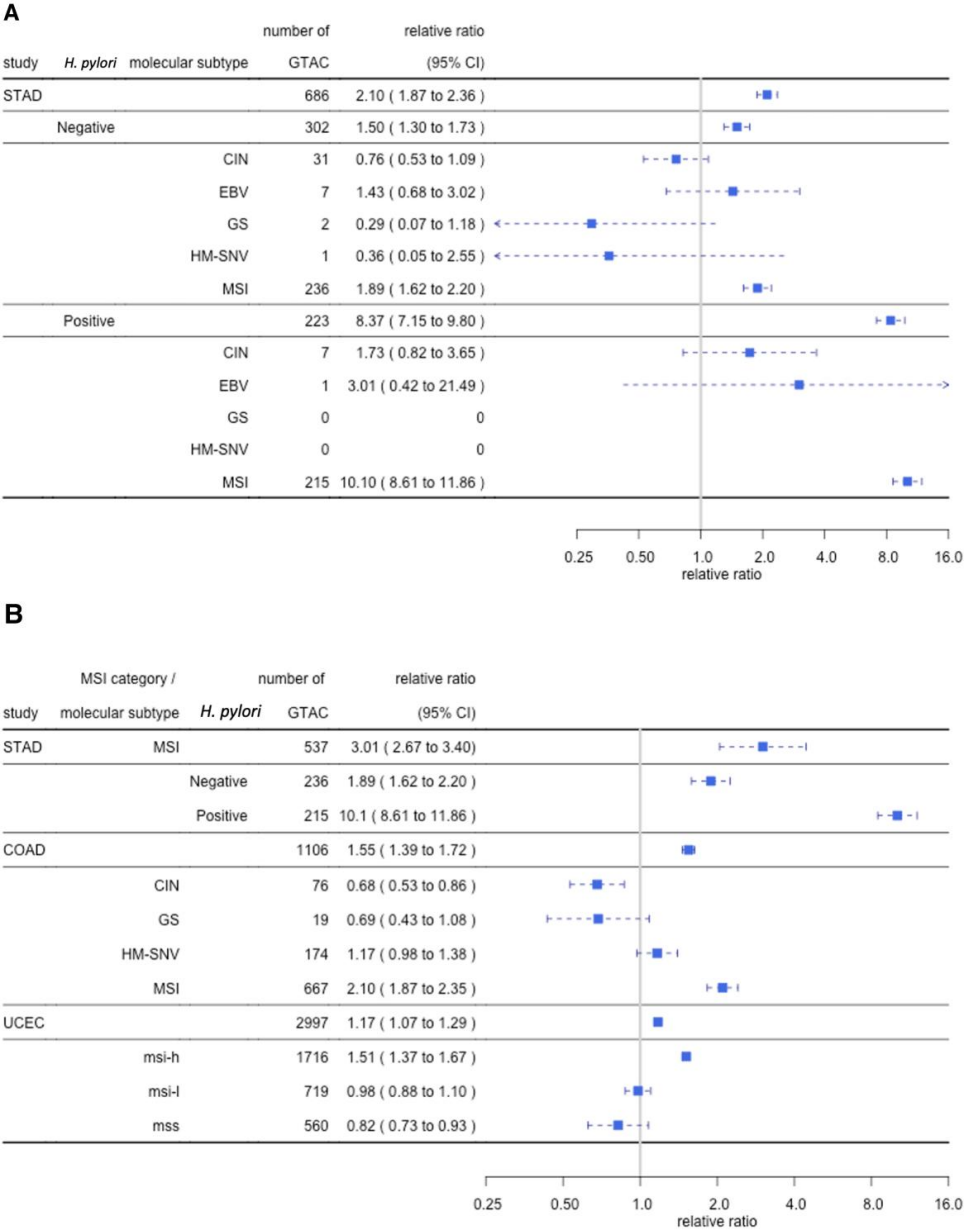
Predominance of the GTAC motif in *H. pylori*-positive MSI subtype of gastric cancer. A) Relative ratio of GTAC mutation motif in molecular subtypes with different *H. pylori* infection states of STAD. For the criteria for testing MSI and *H. pylori*, see Ref. (27). B) Relative ratio of GTAC mutation motif in microsatellite unstable subtypes across three cancer types. The relative ratio is represented by a square, and the 95% CI by a whisker.

Out of the five molecular subtypes of STAD, MSI (microsatellite instability) exhibited a notably high ratio of 10.1 in the presence of *H. pylori* (Fig. 3A). The MSI subtype exhibits an elevated mutation rate due to genetic/epigenetic defects in the mismatch repair system (28, 29). Defective mismatch-repair mutational signature SBS6 (potentially reflecting C-to-T mutations within GCG, CCG, ACG, GCC 3-mer mutation motifs in Fig. S4B. Note that GCG is consolidated with its reverse complement CGC) was found in GC cases. (The MSI subtype of COAD and the msi-h subtype of UCEC displayed a relative ratio of the GTAC mutation motif similar to that observed in *H. pylori-*negative MSI in STAD (Fig. 3B). This suggests no need to postulate involvement of *H. pylori* in these cancer types.)

What could be the roles of the *Hp*PabI-mediated mutagenesis in carcinogenesis? We listed genes with a GT***A***C mutation in these gastric cancer genomes (Table S2) and carried out enrichment analyses (Table S3). KEGG revealed motor proteins such as dynein on microtubules (hsa04814) while GO revealed three groups of categories: (i) cell adhesion/movement/morphogenesis; (ii) transport through membrane; (iii) response to DNA damage among others. We then chose those genes listed in Catalog Of Somatic Mutations In Cancer (Table S4). The genes emerged include well-known ones: TP53, EP300, ATM, FGFR2, DNMT3A (DNA methyltransferase 3A), and MUC16 (a marker for ovarian cancer). Enrichment analysis revealed gene groups mutated in gastric cancer and other types of cancer affecting signal transduction, cell growth/death, and DNA damage response. In molecular terms, many of these genes affect transcription as well as protein phosphorylation. These results give us a concrete picture of how GT***A***C mutagenesis contributes to carcinogenesis/pathogenesis during decades of infection.

In summary, GT***A***C mutations are highly enriched in the *H. pylori*-positive subtype of STAD. This observation is consistent with our hypothesis that *Hp*PabI mediates substitution mutagenesis at A, the site of base excision, within its recognition sequence GTAC, as depicted in Fig. 1A, and ultimately contributes to the development of gastric cancer.

### Preferential mutation at the *Hp*PabI recognition site 5′-GTAC in *H. pylori* genomes

We then analyzed mutagenesis within *H. pylori* genomes, as opposed to human genomes, under the assumption that if *Hp*PabI induces GT***A***C mutations on the human genome, it should induce GT***A***C mutations in *H. pylori* genome. A study isolated multiple *H. pylori* strains from infected individuals and sequenced their whole genomes (30). After reconstructing their phylogenetic trees based on the entire genome, we counted mutations on all the 4-mers (Table S5) and ranked them for mutation frequency at the third letter in each lineage (Table S6, from 4 lineages 100 strains). 5′GT***A***C showed the highest mutation rate among all the 4-mers except for those with central CG, which is known to show high mutation at G because of deamination of its opposite C to U (17). Deamination of 5mC, abundant in *H. pylori* genomes, leads directly to T (17). We concluded that, on *H. pylori* genomes, 5′GTAC shows a high mutation rate at A, the base to be excised by *Hp*PabI (21, 22) on *H. pylori* genomes as in the gastric cancer genomes.

### *Hp*PabI-mediates double-strand breakage in human chromosomes

**Fig. 4:**
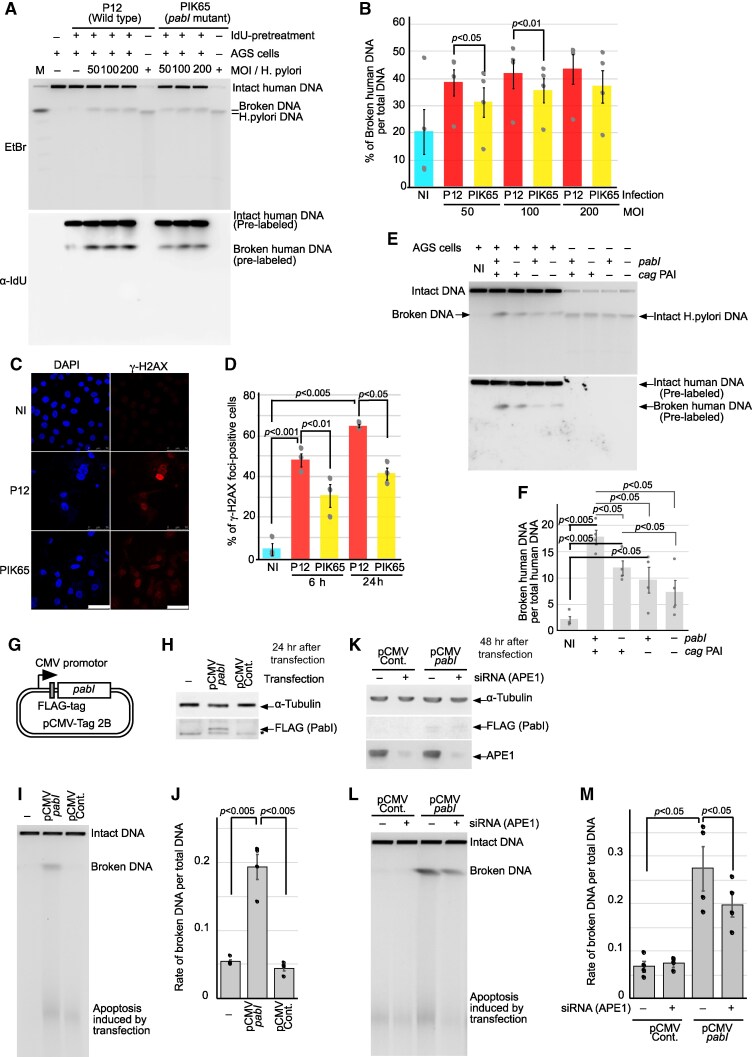
*Hp*PabI-mediated chromosomal breakage during *H. pylori* infection and upon *Hp*PabI gene transfer. A) Analysis of chromosomal breaks postinfection by *H. pylori* and its *Hp*PabI mutant using pulsed-field gel electrophoresis. The total DNA was detected with ethidium bromide. Human chromosomes prelabeled with iododeoxyuridine (IdU) were detected by immunoblotting using an IdU-specific antibody. B) Quantification of broken DNA. Error bars represent SEs. Means and SEs are derived from four independent experiments. C) Detection of γ-H2AX foci, indicative of DNA double-strand breaks, through immunofluorescence analysis. D) Quantification of cells positive for γ-H2AX foci. Error bars represent SEs. E) Chromosomal breaks postinfection with *H. pylori* mutants. F) Quantification based on four separate experiments. G) Expression plasmid. *pabI* = *Hp*PabI. FLAG = DDDDK. CMV, cytomegalovirus. H) Protein expression following transfection. Protein lysates were prepared 24 h after transfection using Lipofectamine 3000. *Hp*PabI expression was detected by immunostaining with anti-FLAG antibody. Alpha-tubulin immunostaining served for control. * denotes a nonspecific band. I) Detection of chromosomal breaks by pulsed-field gel electrophoresis. J) Quantification of broken DNA. The proportion of broken DNA relative to the total DNA was analyzed. The mean and SE values were determined from four independent experiments. K) APE1 depletion using siRNA. Protein lysates were prepared at 48 h posttransfection. L) Impact of APE1 depletion on chromosomal breakage. M) Quantification of broken DNA. The mean and SE values were derived from four independent experiments.

Does *Hp*PabI act on the human chromosomes as expected from the analysis of the cancer genome mutations? We infected cultured human cells (AGS) with *H. pylori*, specifically its wild-type strain (P12) and an *Hp*PabI-deleted mutant strain (Materials and methods). Pulsed-field gel electrophoresis was used to precisely measure chromosomal double-strand breaks. We prelabeled human chromosomal DNA with iododeoxyuridine (IdU) and selectively visualized it by immunoblotting against IdU after transfer to membrane. This allowed distinction between the broken human chromosomes and the *H. pylori* genome (1.7 Mb) migrating at a similar position on agarose gel. We observed accumulation of broken DNAs upon infection, as previously described (13, 14). The broken DNAs were reduced by the *Hp*PabI deletion at multiplicity of infection of 50 and 100 (Figs. 4A, B and S5). These results demonstrate the occurrence of *Hp*PabI-dependent chromosomal double-strand breakage.

This was independently verified by the detection of large foci of γ-H2AX (a phosphorylated form of histone H2AX) (Fig. 4C and D). After human cells on a coverslip were infected with the wild-type or the *Hp*PabI mutant, γ-H2AX was visualized by immunofluorescent staining. The *Hp*PabI mutant gave fewer foci.

Chromosomal double-strand breakage during infection is also decreased by *cagA* deletion and *cag*PAI deletion (14). To investigate the relationship between the roles of CagA (encoded by *cag*PAI) and *Hp*PabI, we compared chromosomal breakage in the *Hp*PabI deletion mutant, a *cag*PAI deletion mutant, and a *Hp*PabI *cag*PAI double mutant (Fig. 4E and F). The breakage in the *Hp*PabI mutant was further decreased by addition of the *cag*PAI mutation (Fig. 4E and F). This aligns with the distinct roles of *Hp*PabI and *cag*PAI in the formation and/or repair of the double-strand breaks.

The contribution of *Hp*PabI to human chromosomal double-strand breakage was further analyzed after transfer and transient expression of its gene in human cells (Fig. 4G–M). Hela cells were used for higher transfection efficiency. The Flag (DDDDK)-tagged *Hp*PabI gene was delivered using an expression vector (Fig. 4G). Successful gene expression was confirmed by an anti-flag antibody (Fig. 4H). *Hp*PabI-dependent double-strand breakage was detected using pulsed-field gel electrophoresis (Fig. 4I and J).

Previous studies have demonstrated that AP sites generated by the bacterial members of PabI family of restriction enzymes give rise to a strand break by their intrinsic AP lyase or separate bacterial AP endonucleases (Fig. 1A) (22 , 23). To clarify the role of human AP endonuclease 1 (APE1) in *Hp*PabI-dependent double-strand break formation in human cells, we transiently depleted APE1 using siRNA (Fig. 4K) under the aforementioned conditions. This depletion resulted in decreased *Hp*PabI-dependent double-strand breakage (Fig. 4L and M). These findings suggest that APE1 contributes to *Hp*PabI-mediated double-strand breakage (Fig. 1A).

Our findings support the role of *Hp*PabI in inducing double-strand breakage in human chromosomes, which is consistent with our hypothesis (Fig. 1A). This occurs through a mechanism different from *cag*PAI action and, at least partly, through base excision followed by AP endonuclease action.

### *Hp*PabI promotes mutagenesis

Our findings so far suggest that *Hp*PabI induces mutations in the human genome, potentially contributing to cancer initiation and development. We further investigated whether *Hp*PabI promotes mutagenesis using an *Escherichia coli* tester system that monitors loss of function of a gene (Materials and methods).

**Fig. 5:**
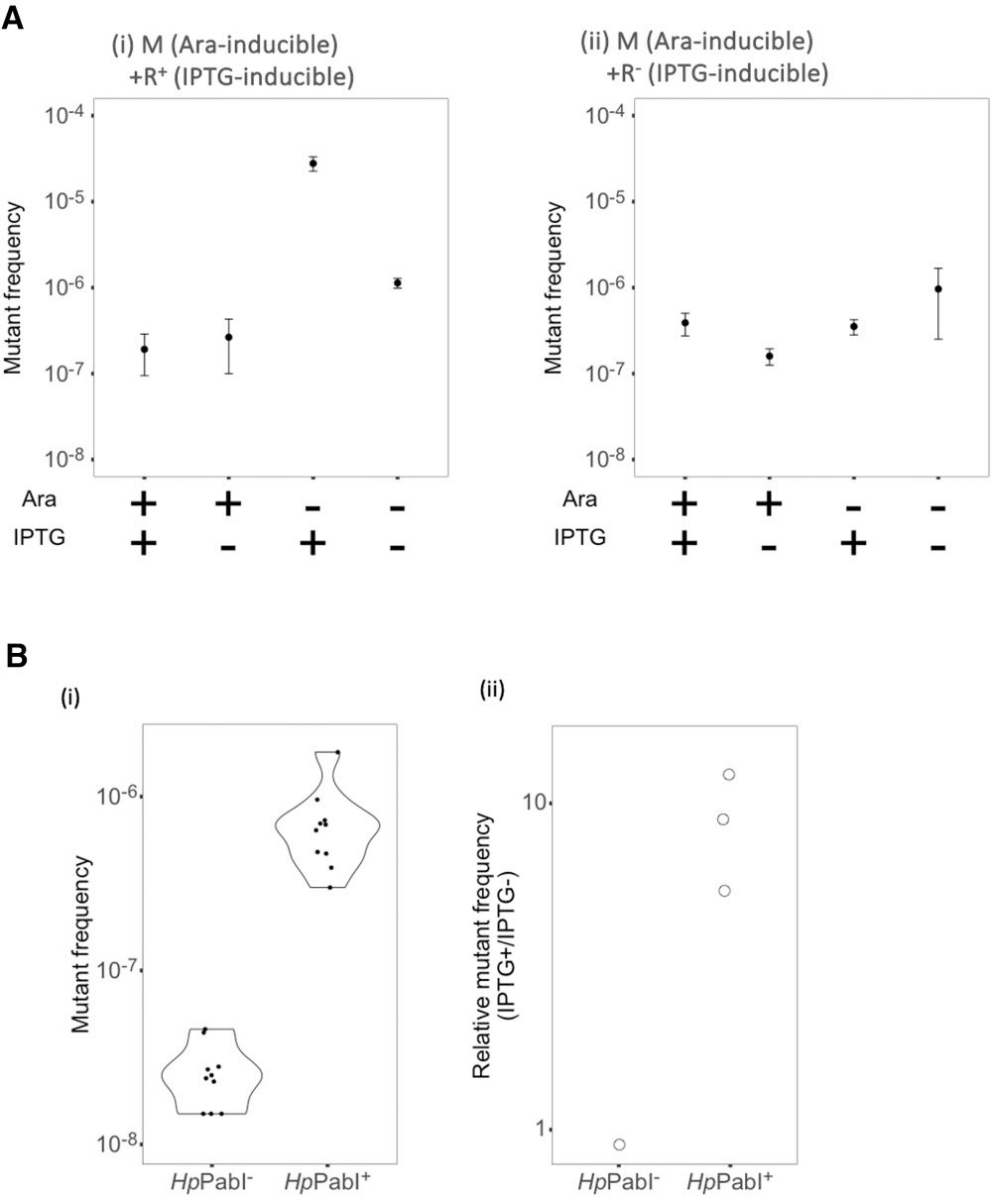
Enhancement of mutant frequency by *Hp*PabI. A) In a tester strain carrying a cognate DNA methyltransferase. An exponential culture derived from a single colony was incubated overnight with or without arabinose/IPTG as indicated in the bottom. Then the streptomycin-resistant mutant cells and the total viable cells were determined by colony formation. Each point represents the average of four measurements, with an error bar denoting the standard error. (i) The *E. coli* strains carry an IPTG-inducible *Hp*PabI gene and an arabinose-inducible DNA methyltransferase gene methylating the same sequence. (ii) The *E. coli* strains lack the *Hp*PabI gene. B) In a tester strain lacking a cognate DNA methyltransferase. (i) *Hp*PabI-mediated mutagenesis. Exponential cultures of *E. coli* strains with or without an IPTG-inducible *Hp*PabI gene was diluted to ∼10^3^ cells/mL and incubated overnight. Then, the streptomycin-resistant mutant cells and the total viable cells were determined. (ii) Increase in mutant frequency upon IPTG-induced *Hp*PabI expression. Exponential cultures of *E. coli* strains with or without an IPTG-inducible *Hp*PabI gene was diluted to ∼10^3^ cells/mL in two tubes. To one of them, IPTG was added before overnight incubation.

In the first set of strains, we introduced an IPTG-inducible *Hp*PabI gene along with arabinose-inducible *cviQIM* for DNA methyltransferase M.CviQI (31) generating GTm6AC (Materials and methods). This DNA methyltransferase methylates the *Hp*PabI recognition sequence 5′-GTAC during arabinose induction, providing protection from restriction attacks and ensuring the viability of the strain. When we removed arabinose and introduced IPTG, we observed an increase in the mutant frequency by nearly two orders of magnitude (Fig. 5A(i)). However, this increase was not observed in the absence of *Hp*PabI (Fig. 5A(ii)). Comparable results were obtained when examining the *Pyrococcus abyssi* homolog (Fig. S9).

In the second strain set, we replaced the Shine-Dalgarno translation initiation signal with a plant-derived translation initiation signal to minimize basal expression, allowing the strain to remain viable even in the absence of the above methyltransferase (Materials and methods). Notably, the presence of *Hp*PabI alone resulted in a one-order-of-magnitude increase in the mutant frequency (Fig. 5B(i)), which was further augmented by an additional order of magnitude upon IPTG induction of *Hp*PabI (Fig. 5B(ii)).

These findings suggest that *Hp*PabI possesses the capacity to promote mutagenesis. This mutagenic effect was attributed to the action of *Hp*PabI on its recognition sequence, 5′-GTAC, as evidenced by prevention through its methylation by a DNA methyltransferase.

### *Hp*PabI structure and evolution

To understand the structural and evolutionary basis underlying *Hp*PabI activity, we generated a computational model of its dimeric structure (Structure S1) using AlphaFold-Multimer v2 (Materials and methods). The model showed high accuracy values (pLDDT 95.1, pTM-score 0.891, and ipTM 0.91) and energy scores comparable to the X-ray structures of PabI from *Pyrococcus abyssi* and CcoLI from *Campylobacter coli* (Fig. S10). The model closely resembled these known structures (Fig. 1B).

Compared with PabI, *Hp*PabI has an insertion next to the catalytic Tyr (Y68 in PabI and Y60 in *Hp*PabI as detailed in Table S7), which is larger than that in CcoLI (Fig. 1C). While *Hp*PabI exhibits a greater overall similarity to CcoLI than to PabI, it is similar to PabI in having a β-strand (-N8) at the N-terminus, a feature absent from CcoLI (Fig. 1B). *Hp*PabI possesses all the triad residues (Y60, H237, and D240; see Table S7) essential for glycosylase activity (Fig. 1C). Furthermore, *Hp*PabI includes a conserved Lys residue (K90), corresponding to K71 of CcoLI implicated in the AP lyase activity (32). Two additional residues explored for lyase activity in CcoLI (D53 and N67) also have counterparts in *Hp*PabI (D61 and N86) (Fig. 1C).

*Hp*PabI dimer bound to DNA was modeled after the structure of PabI dimer in the product-DNA binding state (PDB: 3waz) (structure S2, Fig. 1D). The *Hp*PabI dimer readily accommodated DNA from the PabI–DNA complex. Notably, several positively charged residues within the aforementioned insertion region (K65, K67, and K69) may play a role in DNA binding. Although PabI binds to nonspecific DNA as a tetramer (a dimer of dimers, PDB: 5iff), an attempt to create a tetrameric model of *Hp*PabI based on the corresponding PabI tetramer (Fig. S11) revealed clashes between *Hp*PabI dimers at the characteristic insertion shared by *Hp*PabI and CcoLI. This suggests that *Hp*PabI interacts with nonspecific DNA as a dimer.

The evolution of *Hp*PabI, potentially functioning as an oncoprotein, may have involved interactions with host components and have been associated with diversifying selection, akin to other bacterial virulence proteins. To assess this, we calculated the d*N*/d*S* (rate of nonsynonymous substitution/rate of synonymous substitution) for each codon of *Hp*PabI (Tables S7 and S9) as a metric for diversifying selection within the species (33). As many as 1/4 of the countable residues (24% = 54 aa/227 aa) of *Hp*PabI showed d*N*/d*S* > 1.

Sites with d*N*/d*S* (median) > 1.5 (depicted as magenta sticks) were mapped alongside functionally significant residues (depicted as cyan sticks) on the predicted structure with DNA immediately after the glycosylase reaction (Fig. 1E). Notably, three of these high d*N*/d*S* sites (D87, V89, and A236) clustered at DNA binding sites (K65, K67, and K69), glycosylase active sites (D237, D240, and Y60), and AP lyase active-site candidates (K90, D61, and N86) and may have affected these activities (Fig. 1E (ii), Table S7). Other high d*N*/d*S* sites were proximal to the DNA (R26, Y186, and K45) or around the *Hp*PabI-specific N-terminal beta-strand (D7, N8, and more) (Fig. 1E (i), Table S7). These findings suggest that diversifying selection has acted upon *Hp*PabI interactions with DNA.

### Potential roles of restriction enzymes in various types of cancer

Might cancer types other than gastric cancer be similarly influenced by restriction enzymes from different bacteria? We indeed observed the frequent occurrence of certain 4-mer mutation motifs, such as AC***T***T and TA***A***G in ESCA (esophageal carcinoma) (Fig. S12). In the 2D hierarchical clustering of mutation motifs and cancer types, we observed several areas with high relative ratio and several areas with low relative ratio (Figs. 2A, S4, and S7).

To explore the above intriguing possibility, we expanded our analysis to encompass mutation motifs ranging from 3-mer to 7-mer in various cancer genomes. For the top 10 mutation motifs, ranked by relative ratio, in each *k*-mer mutation motif in each cancer, we listed bacterial species known to carry a restriction–modification system related to the respective motif. We utilized recognition sequence data and Pacbio methylation motif data in REBASE (rebase.neb.com/rebase). In our above analyses, we focused on the specific locations of the mutation (second base in 3-mers, third base in 4-mers, and third base in 5-mers). However, in this comprehensive analysis, we considered mutations occurring at any position within a k-mer.

From the extensive dataset, we filtered the top 10 genera using the sum of the R gene count, M gene count and REBASE PacBio organism count (Dataset 2). Further refinement involved identifying the genera associated with cancer based on the BIC database (which has small-RNA sequencing data linking bacteria to tissues) (34). We also cross-referenced these genera with those documented as residents of the relevant organ or tissue in at least one of the microbiome reviews we preselected. Consequently, we identified 95 genera of bacteria predicted to potentially contribute to cancer by delivering restriction enzymes (Table S8). Each motif was annotated with the relative ratio of cancer mutations and compositional bias in the bacterial genome (Fig. S13). Some of the restriction motifs in Table S8 include a previously identified mutation signature: pyrimidine dimers and SBS7a, b (SKCM), SBS10a, c, d (UCEC), and SBS10b (COAD).

Notably, we found many mutation motifs that contains the start codons (ATG; CTG for MHC Class I (35)) or stop codons (TAA, TAG, TGA) (see Table S8 for underlined sequences) in some types of cancer. Some recognition sequences carry both a start codon and a stop codon: GCT***G***A by Staphylococcus in breast invasive carcinoma and AT***G***AG by Bacteroides, GCT***G***A by Enterococcus, CCT***G***A by Haemophilus, GGCT***G***A by Lactococcus in head and neck squamous cell carcinoma. These bacterial restriction enzymes may target start and stop codons in the human genome to affect its genes' integrity and activity. Earlier a restriction–modification system (IceA) recognizing CATG of *H. pylori* was implicated in cancer (36). DNA methylation at the start or stop codons affects transcription in *H. pylori* (37). We do not know whether targeting these codons helps restriction in bacteria.

Out of the wide implications of Table S8, we here just mention those of gastric cancer (STAD). The presence of Helicobacter restriction enzymes other than the PabI family restriction glycosylase (recognizing only GTAC so far) here suggests that *H. pylori* may also deliver them to cause cancer. Gastric cancer emerges even after *H. pylori* eradication (38). Fusobacterium cells prominent in the posteradication stomach microbiome and their culture medium can cause chromosomal double-strand breaks in human cells in vitro and in mouse stomach cells in vivo with a heat-labile genotoxin (39), which could be their restriction enzymes, possibly with the listed sequence specificity. Fusobacterium may cause colorectal cancer (40) with these restriction enzymes.

## Discussion

We hypothesized that *H. pylori* causes gastric cancer by mutagenizing and rearranging human genome with its toxic base-excision restriction enzyme *Hp*PabI (Fig. 1A) and obtained its evidence in multiple lines: its association with gastric cancer (Table 1), mutation signature at its recognition sequence in gastric cancer genomes (Figs. 2 and 3), human chromosome breakage (Fig. 4), and mutagenesis (Fig. 5). These results prompted intriguing extrapolation about possible influence of restriction enzymes from various bacteria on cancer development across different organ systems (Table S8).

The mutation signature at its recognition sequence 5′GTAC is especially high in MSI subtype characterized by defective mismatch repair. The involvement of *Hp*PabI in gastric carcinogenesis (Fig. 1A) explains the several relations of gastric cancer with mismatch repair deficiency: (i) the mismatch repair deficiency signatures in gastric cancer (see above and Refs. (41, 42)), (ii) the genetic/epigenetic inactivation of mismatch-repair genes in gastric cancer cells (28, 29), and (iii) frequent gastric cancer in Lynch syndrome with hereditary deficiency in mismatch repair (43). The defect in mismatch repair likely has enhanced *Hp*PabI-mediated mutagenesis (Fig. 1A). Our model (Fig. 1) can also explain why this association with mismatch repair deficiency can take place. The lethal double-strand breakage pathway of *Hp*PabI from the intermediate AP site may have selected mismatch-repair deficient cells that allows the alternative mutagenesis pathway (Fig. 1A, right). In general mismatch repair system kills cells by double-strand breakage so that exposure to genotoxins leads to selection of mismatch repair-deficient cells (44). The combined mutagenic effects of *Hp*PabI itself and the mismatch repair deficiency promote high mutagenesis in MSI subtype, which generates many neo-antigens and allows anti-cancer action of immune cells. This picture applies to association of other bacteria and mismatch-repair-deficient MSI subtype of diverse types of cancer such as Fusobacteria and colorectal cancer (45) (Table S8).

The involvement of *Hp*PabI also explains the interaction between *H. pylori* infection and germline variations in homologous-recombination genes with respect to the risk of gastric cancer (8). It also explains SBS3 (BRCA1/2) mutational signature (41). Homologous recombination function would repair chromosomal breaks introduced by *Hp*PabI and prevent its mutagenic/rearrangement effects (Fig. 1A). In cultured human cells, *Hp*PabI and *cag*PAI separately contribute to double-strand breakage (Fig. 4E and F). The breakage promotes expression of targets of NF-κB, a general regulator of inflammation (15). The inflammation is consistent with SBS18 signature (damage by ROS) in the gastric cancer genomes (41). In addition to gastric cancer, *Hp*PabI may be responsible for microsatellite instability and extensive genome rearrangements in gastric MALT lymphoma caused by *H. pylori* (46). PabI family members are present in other Helicobacter, Campylobacter, and related species (24) and could be related to their suspected role in hepatobiliary and other types of cancer (Table S8) (47).

The results using *E. coli* tester strains are clear, suggesting that various genotypes of *E. coli* can be effectively tested. However, it remains uncertain how closely the mechanisms of mutagenesis in *E. coli* mirror those in human cells. Detecting mutagenesis in human cells is both costly and time-consuming. As an alternative, assessing chromosomal double-strand breakage under different conditions in human cells appears more feasible. In our present study, we demonstrated the involvement of APE1, an AP endonuclease (Fig. 4g–m). Previous studies have identified germline mutations in homologous recombination genes involved in chromosomal double-strand break repair (e.g. BRCA2, BRCA1) and mismatch repair genes (MSH6, MSH2, MLH1) as being associated with gastric cancer. This aligns with the proposed involvement of *Hp*PabI-mediated chromosomal double-strand breakage and mutagenesis, as illustrated in Fig. 1A.

We noted that several *H. pylori* strains used for rodent infection experiments have a decayed form of *Hp*PabI gene (see Introduction section), which might have contributed to the difficulty in realizing oncogenesis. Infection in vivo with *Hp*PabI-gene positive strains might overcome the difficulty and serve to validate the above findings. Further investigation in silico, in vitro, and in vivo is warranted to understand this novel mode of bacterial oncogenesis in gastric and other types of cancer. The oncogenic restriction enzymes identified in this work would also make novel targets in cancer therapy.

## Materials and methods

Population structure analysis, Assessing association of *Hp*PabI gene with gastric cancer, Motif analysis with TCGA dataset, Analysis of genes with a GTAC mutation on gastric cancer genomes, Analysis of 4-mer mutations on *H. pylori* genomes, *H. pylori cag*PAI mutant, Cell lines and culture, Detection of chromosomal double-strand breakage by pulsed-field gel electrophoresis, Immunofluorescent staining, Transfection and silencing, Mutation testers, Measuring mutant frequency, Structural modeling and analysis, and d*N*/d*S* calculation are detailed in SI Materials and methods as Supplementary Information.

## Supplementary Material

pgaf244_Supplementary_Data

## Data Availability

The whole genome sequences generated within the *Hp*GP are available at NCBI GenBank database (BioProject accession code PRJNA529500). The computational scripts are available at GitHub (https://github.com/nosada17/Hpylori/tree/main/population_structure) and Zenodo (DOI 10.5281/zenodo.10209881).
