# Supplementary material for: Helicobacter pylori base-excision restriction enzyme in stomach carcinogenesis
Source: PNAS Nexus. 2025 Aug 5;4(8):pgaf244. doi: 10.1093/pnasnexus/pgaf244 (PMC12366791; doi:10.1093/pnasnexus/pgaf244)
Supplement: pgaf244_Supplementary_Data [file pgaf244_supplementary_data.zip › PNASNEXUS-PNASNEXUS-2024-00952RR-s06.pdf]

A

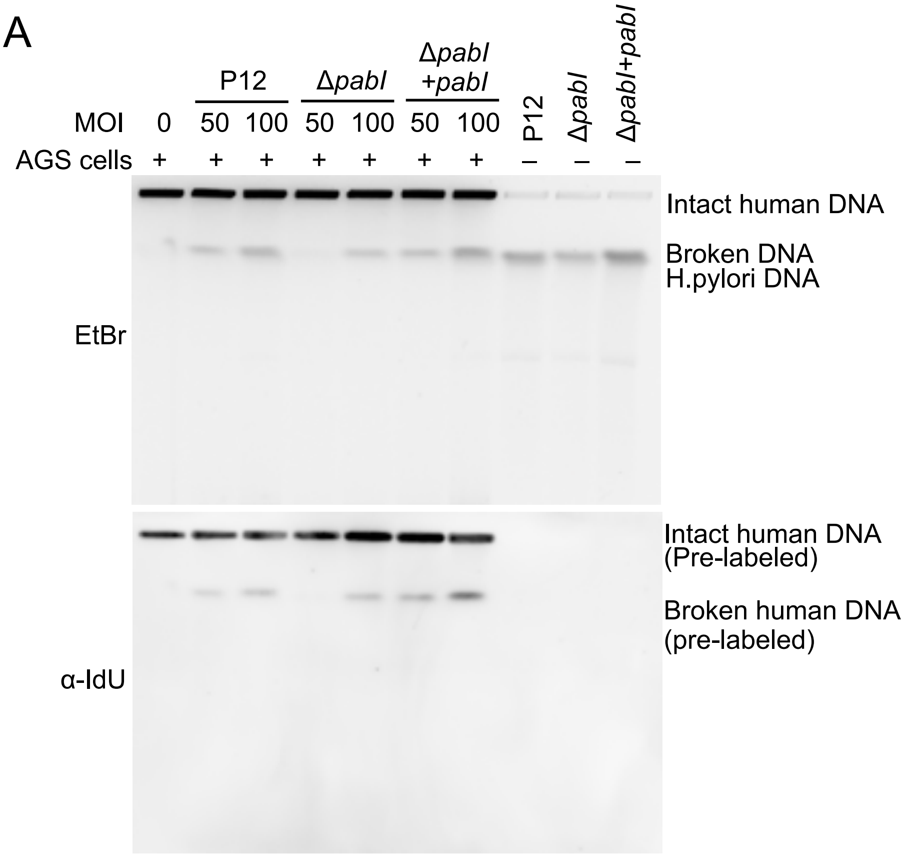

P12
Δpabl
Δpabl +pabl
MOI
0
50
100
50
100
50
100
P12
Δpabl
Δpabl+pabl
AGS cells
+
–
Intact human DNA
Broken DNA
H.pylori DNA
EtBr
Intact human DNA (Pre-labeled)
Broken human DNA (pre-labeled)
α-IdU

B

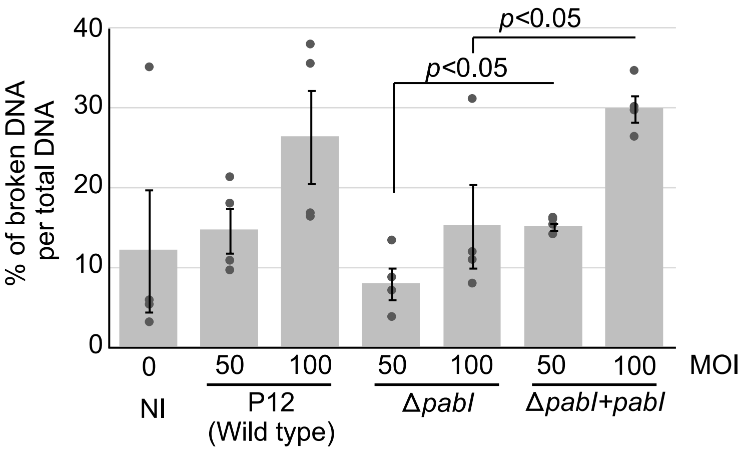

% of broken DNA per total DNA
40
30
20
10
0
p<0.05
p<0.05
0
50
100
50
100
50
100
MOI
NI
P12 (Wild type)
Δpabl
Δpabl+pabl

Supplementary Fig. S5 *Hp*PabI-mediated chromosomal breakage during *H. pylori* infection.

(A) Analysis of chromosomal breaks post-infection by *H. pylori* P12 (wild type), Δ *pabI* mutant, and its complimented strain (Δ*pabI* + *pabI*) using by pulsed-field gel electrophoresis. Total DNA was detected with ethidium bromide. The human chromosomes pre-labeled with iododeoxyuridine (IdU) were detected via immunoblotting using an IdU-specific antibody.

(B) Quantification of the broken DNA. Error bars represent standard error (SE). Mean and SE were derived from four independent experiments.
