# Supplementary material for: Helicobacter pylori base-excision restriction enzyme in stomach carcinogenesis
Source: PNAS Nexus. 2025 Aug 5;4(8):pgaf244. doi: 10.1093/pnasnexus/pgaf244 (PMC12366791; doi:10.1093/pnasnexus/pgaf244)
Supplement: pgaf244_Supplementary_Data [file pgaf244_supplementary_data.zip › PNASNEXUS-PNASNEXUS-2024-00952RR-s07.docx]

**Table S6. Ranking of 4-mers for mutation frequency on *H. pylori* genome during infection.**

| Motif ＼lineage | 479 | 476 | 169 | 25 |
| --- | --- | --- | --- | --- |
| AC***G***T | 1 (0.15) | 1 (0.20) | 1 (0.02) | 1 (0.05) |
| GT***A***C | 2 (0.10) | 3 (0.08) | 2 (0.02) | 4 (0.02) |
| GC***A***C | 3 (0.06) | 4 (0.07) | 3 (0.01) | 5 (0.02) |
| TC***G***A | 4 (0.06) | 2 (0.09) | 75 (0.00) | 3 (0.02) |
| GC***G***C | 5 (0.06) | 5 (0.07) | 5 (0.01) | 2 (0.03) |

The frequency in the parentheses indicate [number of unique mutations in the genome / number of motifs in the genome]. Substitution at the 3rd letter was measured. CG (underlined) shows high mutation at G because of C deamination.
