# Supplementary material for: Helicobacter pylori base-excision restriction enzyme in stomach carcinogenesis
Source: PNAS Nexus. 2025 Aug 5;4(8):pgaf244. doi: 10.1093/pnasnexus/pgaf244 (PMC12366791; doi:10.1093/pnasnexus/pgaf244)
Supplement: pgaf244_Supplementary_Data [file pgaf244_supplementary_data.zip › PNASNEXUS-PNASNEXUS-2024-00952RR-s08.docx]

Table S7. Amino acid sequence with dN/dS of *Hp*PabI.

references

| **AA**  **position** | **low 95%** | **median**  >1.5 | **high 95%** | **HPAG1 AA** | ***P.***  ***abyssi*** | **function**  Important in function |
| --- | --- | --- | --- | --- | --- | --- |
| 1 | 0,112 | 0,643 | 1,92 | V |  | absent from CcoLI |
| 2 | 0,103 | 0,668 | 1,968 | S |  | absent from CcoLI |
| 3 | 0,003 | 0,217 | 1,383 | L |  | absent from CcoLI |
| 4 | 0,119 | 0,724 | 2,001 | I |  | absent from CcoLI |
| 5 | 0,192 | 0,888 | 2,524 | R |  | absent from CcoLI |
| 6 | 0,123 | 0,814 | 2,485 | I |  | absent from CcoLI |
| 7 | 0,508 | 1,557 | 3,331 | D |  | absent from CcoLI |
| 8 | 2,032 | 4,168 | 7,435 | N |  | absent from CcoLI |
| 9 | 0,31 | 1,236 | 2,992 | N |  |  |
| 10 | 0,131 | 0,746 | 2,089 | K |  |  |
| 11 | 0,104 | 0,604 | 1,78 | K |  |  |
| 12 | 0,498 | 1,533 | 3,565 | V |  |  |
| 13 | 0,089 | 0,593 | 1,797 | I |  |  |
| 14 | 0,316 | 1,15 | 2,857 | E |  |  |
| 15 | 0,546 | 1,69 | 3,777 | V |  |  |
| 16 | 0,281 | 1,136 | 2,7 | S |  |  |
| 17 | 0,04 | 0,435 | 1,644 | I |  |  |
| 18 | 0,099 | 0,641 | 1,964 | P |  |  |
| 19 | 0,045 | 0,715 | 2,706 | L |  |  |
| 20 | 0,188 | 0,861 | 2,265 | T |  |  |
| 21 | 0,364 | 1,356 | 3,329 | S |  |  |
| 22 | 0,381 | 1,413 | 3,365 | I |  |  |
| 23 | 0,378 | 1,438 | 3,478 | S |  |  |
| 24 | 0,321 | 1,136 | 2,573 | G |  |  |
| 25 | 0,296 | 1,118 | 2,637 | K |  |  |
| 26 | 0,787 | 2,095 | 4,499 | V |  |  |
| 27 | 0,17 | 0,881 | 2,345 | R | R32 | specific DNA binding |
| 28 | 0,092 | 0,577 | 1,777 | V |  |  |
| 29 | 0,064 | 0,583 | 1,927 | K |  |  |

46

46

| 30 0,093 |  | 0,546 | 1,741 | I |  | |
| --- | --- | --- | --- | --- | --- | --- |
| 31 0,092 |  | 0,611 | 1,869 | R |  |  |
| 32 0,182 |  | 0,818 | 2,225 | H |  |  |
| 33 0,213 |  | 0,933 | 2,517 | A |  |  |
| 34 0,003 |  | 0,163 | 0,874 | F |  |  |
| 35 0,025 |  | 0,357 | 1,312 | S |  |  |
| 36 0,007 |  | 0,161 | 0,884 | D |  |  |
| 37 0,13 |  | 0,695 | 2,032 | Y |  |  |
| 38 0,159 |  | 0,719 | 1,945 | G |  |  |
| 39 0,541 |  | 1,693 | 3,823 | I |  |  |
| 40 0,138 |  | 0,769 | 2,355 | S |  |  |
| 41 0,08 |  | 0,573 | 1,791 | T |  |  |
| 42 0,299 |  | 1,126 | 2,81 | A |  |  |
| 43 0,216 |  | 1,001 | 2,642 | T |  |  |
| 44 0,124 |  | 0,697 | 2,021 | R |  |  |
| 45 0,608 |  | 1,794 | 3,886 | K |  |  |
| 46 0,212 |  | 0,939 | 2,393 | I |  |  |
| 47 0,57 |  | 1,782 | 3,944 | P |  |  |
| 48 0,025 |  | 0,317 | 1,245 | F |  |  |
| 49 0,732 |  | 2,111 | 4,087 | S |  |  |
| 50 0,056 |  | 0,754 | 2,502 | L |  |  |
| 51 0,159 |  | 0,847 | 2,269 | K |  |  |
| 52 0,214 |  | 0,985 | 2,441 | H |  |  |
| 53 0,109 |  | 0,704 | 2,112 | Y |  |  |
| 54 0,398 |  | 1,457 | 3,473 | V |  |  |
| 55 0,271 |  | 0,979 | 2,312 | E | E63 | specific DNA binding |
| 56 0,274 |  | 1,199 | 3,063 | W |  |  |
| 57 NA | NA |  | NA | Q |  |  |
| 58 NA | NA |  | NA | I |  |  |
| 59 NA | NA |  | NA | G |  |  |
| 60 NA | NA |  | NA | Y | Y68 | glycosylase |
| (CcoLI  61 NA NA NA D D53) | | | | | | AP lyase? |

20

1. NA NA NA V

30

HpPabI/CcoLI-specific insertion

1. 0,358 1,236 2,871 P HpPabI/CcoLI-specific insertion
2. 0,037 0,365 1,198 I HpPabI/CcoLI-specific insertion

HpPabI/CcoLI-specific insertion;

DNA binding

1. 0,135 0,752 2,153 K
2. 0,034 0,348 1,272 D HpPabI/CcoLI-specific insertion

HpPabI/CcoLI-specific insertion;

DNA binding

1. 0,114 0,629 1,928 K
2. 0,089 0,618 1,804 E HpPabI/CcoLI-specific insertion

HpPabI/CcoLI-specific insertion;

DNA binding

1. 0,005 0,181 0,977 K
2. 0,033 0,395 1,531 F HpPabI/CcoLI-specific insertion
3. 0,038 0,398 1,471 E HpPabI/CcoLI-specific insertion
4. 0,04 0,409 1,5 L HpPabI/CcoLI-specific insertion
5. 0,051 0,45 1,525 T HpPabI/CcoLI-specific insertion
6. 0,202 0,822 2,228 T HpPabI/CcoLI-specific insertion
7. 0,046 0,576 2,126 L HpPabI/CcoLI-specific insertion
8. 0,035 0,413 1,492 K HpPabI/CcoLI-specific insertion
9. 0,034 0,388 1,365 D HpPabI/CcoLI-specific insertion
10. 0,103 0,627 1,887 E HpPabI/CcoLI-specific insertion

HpPabI/CcoLI-specific insertion HpPabI/CcoLI-specific insertion

| 79 | 0,041 |  | 0,408 | 1,42 | K |  |
| --- | --- | --- | --- | --- | --- | --- |
| 80 | 0,033 |  | 0,377 | 1,335 | Y |  |
| 81 | 0,202 |  | 0,906 | 2,351 | H |  |
| 82 | 0,092 |  | 0,614 | 1,86 | F |  |
| 83 | 0,166 |  | 0,999 | 2,888 | L |  |
| 84 | 0,176 |  | 0,881 | 2,483 | G |  |
| 85 | 0,335 |  | 1,277 | 3,11 | A |  |
| 86 | 0,002 |  | 0,154 | 0,86 | N | Absent  (CcoLI |
|  |  |  |  |  |  | N6) |
| 87 | 0,618 |  | 1,832 | 3,854 | D |  |
| 88 | 0,229 |  | 0,975 | 2,605 | K |  |
| 89 | 0,772 |  | 2,06 | 4,224 | V |  |
| 90 | 0,006 |  | 0,163 | 0,862 | K | K73  (CcoLI |
|  |  |  |  |  |  | K71) |
| 91 | 0,005 |  | 0,136 | 0,775 | T |  |
| 92 | 0,192 |  | 0,886 | 2,264 | L |  |
| 93 | 0,003 |  | 0,172 | 1,053 | Y |  |
| 94 | 0,037 |  | 0,369 | 1,356 | E |  |
| 95 | 0,006 |  | 0,212 | 1,317 | L |  |
| 96 | 0,082 |  | 0,544 | 1,57 | S |  |
| 97 | 0,003 |  | 0,153 | 0,86 | E |  |
| 98 | 0,195 |  | 1,011 | 2,511 | M |  |
| 99 | 0,03 |  | 0,374 | 1,442 | I |  |
| 100 | 0,178 |  | 0,823 | 2,183 | Y |  |
| 101 | 0,04 |  | 0,387 | 1,433 | Y |  |
| 102 | 0,423 |  | 1,394 | 3,147 | A |  |
| 103 | 0,035 |  | 0,36 | 1,413 | K |  |
| 104 | 0,184 |  | 0,964 | 2,752 | Q |  |
| 105 | 0,12 |  | 0,825 | 2,539 | L |  |
| 106 | 0,52 |  | 1,738 | 3,726 | G |  |
| 107 | NA | NA |  | NA | I |  |

30

AP lyase?

30

AP lyase

AP lyase?

1. NA NA NA L
2. NA NA NA S
3. NA NA NA L
4. NA NA NA E
5. NA NA NA N
6. 0,003 0,207 1,259 L
7. 0,038 0,388 1,435 E
8. 0,002 0,156 0,894 N
9. 0,194 0,957 2,525 T
10. 0,005 0,263 1,547 L

118 0,1 0,585 1,787 K

119 0,193 0,976 2,586 Y

120 0,059 0,548 2,016 L

121 0,105 0,602 1,713 E

122 0,037 0,397 1,44 K

123 0,005 0,209 1,253 Q

124 0,03 0,418 1,436 K

125 0,055 0,534 2,051 Q

126 0,005 0,148 0,799 F

127 0,003 0,132 0,779 I

128 0,181 0,845 2,224 E

129 0,322 1,257 2,861 D

1. NA NA NA N
2. NA NA NA F
3. NA NA NA M
4. NA NA NA I
5. NA NA NA T
6. NA NA NA R
7. 0,091 0,589 1,859 E
8. 0,228 0,939 2,494 R
9. 0,041 0,36 1,302 F
10. 0,039 0,437 1,581 R
11. 0,367 1,412 3,451 S
12. 0,197 0,852 2,311 H
13. 0,052 0,551 2,111 Q

46

| 143 | 0,003 | 0,154 | 0,89 | F |  | |
| --- | --- | --- | --- | --- | --- | --- |
| 144 | 0,3 | 1,097 | 2,871 | G |  |  |
| 145 | 0,109 | 0,636 | 1,89 | G |  |  |
| 146 | 0,172 | 0,693 | 1,85 | M |  |  |
| 147 | 0,207 | 0,905 | 2,284 | D |  |  |
| 148 | 0,002 | 0,163 | 0,929 | F |  |  |
| 149 | 0,036 | 0,393 | 1,359 | E |  |  |
| 150 | 0,038 | 0,395 | 1,51 | L |  |  |
| 151 | 0,108 | 0,682 | 2,13 | S |  |  |
| 152 | 0,381 | 1,326 | 3,034 | R |  |  |
| 153 | 0,034 | 0,357 | 1,379 | I |  |  |
| 154 | 0,206 | 0,903 | 2,404 | S |  |  |
| 155 | 0,034 | 0,382 | 1,369 | Y | Y134 | unwinding, cleavage |
| 156 | 0,186 | 0,842 | 2,286 | P |  |  |
| 157 | 0,002 | 0,197 | 1,254 | L |  |  |
| 158 | 0,098 | 0,636 | 1,918 | L |  |  |
| 159 | 0,098 | 0,636 | 1,875 | I |  |  |
| 160 | 0,2 | 0,862 | 2,24 | H |  |  |
| 161 | 0,134 | 0,733 | 2,198 | S |  |  |
| 162 | 0,025 | 0,336 | 1,244 | F |  |  |
| 163 | 0,098 | 0,629 | 1,845 | D |  |  |
| 164 | 0,039 | 0,406 | 1,507 | D |  |  |
| 165 | 0,097 | 0,57 | 1,756 | N |  |  |
| 166 | 0,109 | 0,754 | 2,315 | Q |  |  |
| 167 | 0,006 | 0,218 | 1,282 | L |  |  |
| 168 | 0,193 | 0,864 | 2,296 | S |  |  |
| 169 | 0,028 | 0,379 | 1,279 | E |  |  |
| 170 | 0,136 | 0,693 | 1,881 | I |  |  |
| 171 | 0,05 | 0,465 | 1,642 | V |  |  |
| 172 | 0,002 | 0,155 | 0,858 | I |  |  |
| 173 | 0,304 | 1,091 | 2,634 | R |  |  |
| 174 | 0,032 | 0,375 | 1,36 | E |  |  |
| 175 | 0,292 | 1,268 | 3,337 | Q | K154 | unwinding |
| 176 | 0,058 | 0,539 | 1,911 | Q |  |  |
| 177 | 0,102 | 0,643 | 1,907 | Y |  |  |

46

178 0,113 0,618 1,869 G

179 0,126 0,687 2,119 S

180 0,033 0,364 1,387 K

181 0,335 1,137 2,709 T

182 0,045 0,503 1,723 Q

183 0,226 0,974 2,629 A

184 0,082 0,539 1,572 M

185 0,046 0,483 1,729 L

186 0,691 4,028 Y

1,951

187 0,106 0,603 1,807 F

188 0,287 0,986 2,471 C

189 0,123 0,653 1,852 F

190 0,313 1,122 2,865 S

191 0,39 1,371 3,175 I

192 0,233 1,04 2,841 L

193 0,036 0,347 1,285 E

194 0,054 0,683 2,309 L

195 0,13 0,691 2,044 K

196 0,173 0,894 2,445 T

197 0,342 1,292 3,096 A

198 0,713 4,33 T

2,103

199 0,207 0,949 2,541 P

200 0,049 0,545 1,993 L

201 0,052 0,525 1,938 L

202 0,166 0,705 1,918 N

1. NA NA NA R
2. NA NA NA T
3. NA NA NA A
4. NA NA NA A
5. NA NA NA L
6. NA NA NA K
7. 0,044 0,378 1,337 E
8. 0,519 3,331 H

1,544

1. 0,176 0,808 2,157 A
2. 0,226 1,082 2,748 L

20

| 213 | 0,054 | 0,536 | 2 | L |  | |
| --- | --- | --- | --- | --- | --- | --- |
| 214 | 0,545 | 1,711 | 3,704 | T |  |  |
| 215 | 0,033 | 0,424 | 1,464 | I |  |  |
| 216 | 0,304 | 1,148 | 2,771 | H |  |  |
| 217 | 0,292 | 1,26 | 3,153 | K |  |  |
| 218 | 0,433 | 1,391 | 3,258 | T |  |  |
| 219 | 0,004 | 0,15 | 0,922 | N |  |  |
| 220 | 0,129 | 0,686 | 2,101 | A |  |  |
| 221 | 0,883 | 2,25 | 4,484 | L |  |  |
| 222 | 0,393 | 1,286 | 2,889 | M |  |  |
| 223 | 0,103 | 0,593 | 1,722 | F |  |  |
| 224 | 0,005 | 0,26 | 1,413 | L |  |  |
| 225 | 0,13 | 0,669 | 2,094 | E |  |  |
| 226 | 0,084 | 0,517 | 1,597 | M |  |  |
| 227 | 0,041 | 0,48 | 1,661 | L |  |  |
| 228 | 0,031 | 0,385 | 1,401 | K |  |  |
| 229 | 0,212 | 0,962 | 2,579 | I |  |  |
| 230 | 0,029 | 0,361 | 1,425 | F |  |  |
| 231 | 0,186 | 0,872 | 2,44 | G |  |  |
| 232 | 0,203 | 0,945 | 2,5 | L |  |  |
| 233 | 0,054 | 0,629 | 2,228 | L |  |  |
| 234 | 0,183 | 0,782 | 2,056 | S |  |  |
| 235 | 0,139 | 0,878 | 2,634 | Q |  |  |
| 236 | 0,716 | 2,137 | 4,384 | A |  |  |
| 237 | 0,034 | 0,39 | 1,477 | H | H211 | glycosylase |
| 238 | 0,082 | 0,583 | 1,762 | H |  |  |
| 239 | 0,401 | 1,304 | 2,978 | N |  |  |
| 240 | 0,372 | 1,263 | 2,951 | D | D214 | glycosylase |
| 241 | 0,343 | 1,306 | 3,216 | V |  |  |
| 242 | 0,054 | 0,582 | 2,119 | L |  |  |
| 243 | 0,174 | 0,825 | 2,143 | K |  |  |
| 244 | 0,034 | 0,372 | 1,329 | I |  |  |
| 245 | 0,042 | 0,542 | 1,942 | L |  |  |
| 246 | 0,373 | 1,36 | 3,128 | E |  |  |
| 247 | 0,036 | 0,347 | 1,328 | K |  |  |

20

| 248 | 0,157 | 0,742 | 1,96 | I |
| --- | --- | --- | --- | --- |
| 249 | 0,035 | 0,427 | 1,517 | L |
| 250 | 0,047 | 0,548 | 1,936 | Q |
| 251 | 0,041 | 0,434 | 1,529 | N |

theta 0,535 0,603 0,668

kappa 6,585 7,98 9,396
