# Supplementary material for: Helicobacter pylori base-excision restriction enzyme in stomach carcinogenesis
Source: PNAS Nexus. 2025 Aug 5;4(8):pgaf244. doi: 10.1093/pnasnexus/pgaf244 (PMC12366791; doi:10.1093/pnasnexus/pgaf244)
Supplement: pgaf244_Supplementary_Data [file pgaf244_supplementary_data.zip › PNASNEXUS-PNASNEXUS-2024-00952RR-s09.pdf]

Fig. S8

A *H. pylori*-positive and *H. pylori*-negative STAD

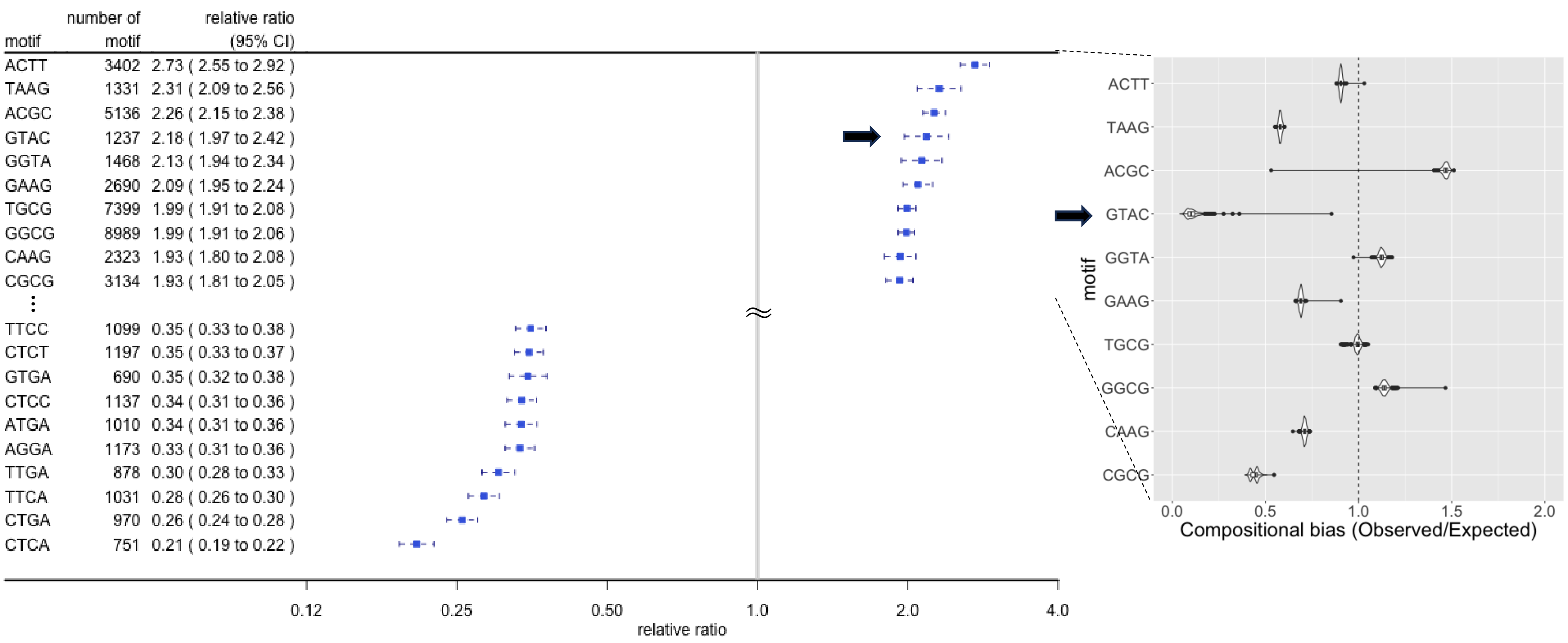

B *H. pylori*-negative STAD

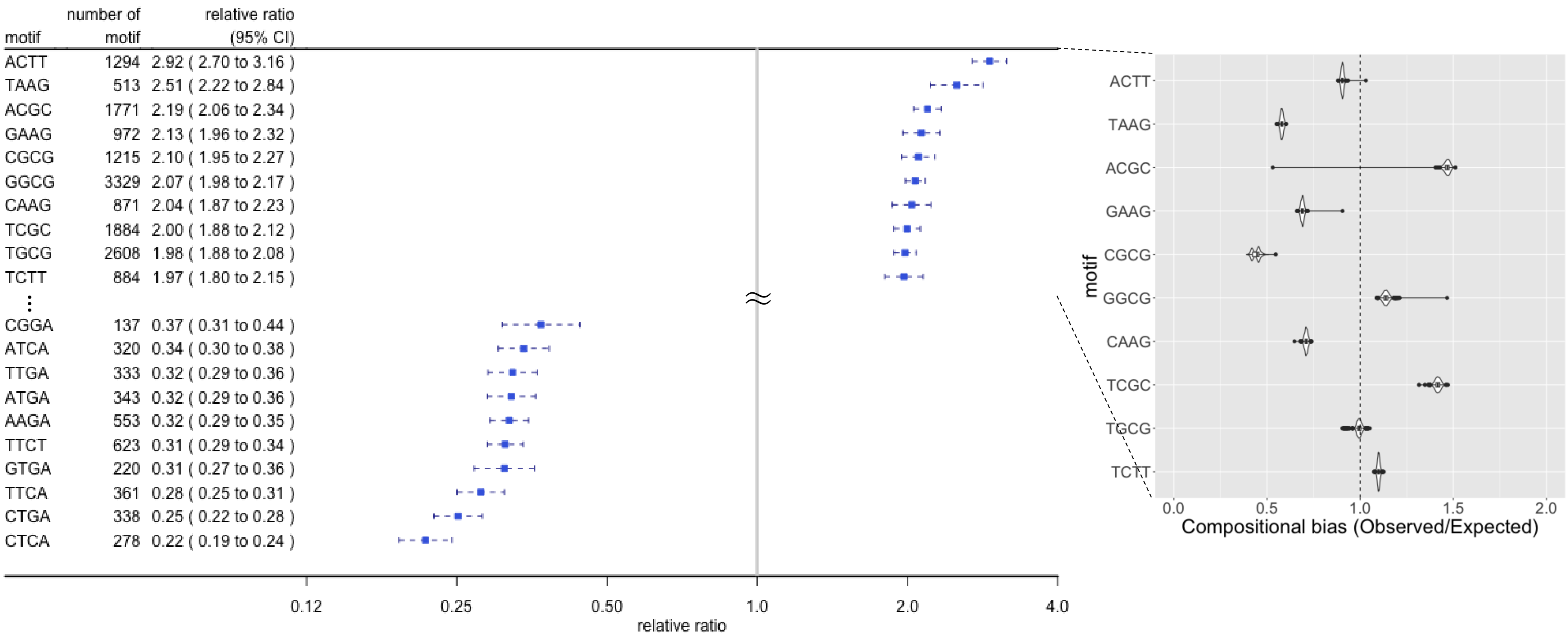

Fig. S8. Top and bottom ten 4-mer motifs in STAD.

(A) *H. pylori*-positive and *H. pylori*-negative STAD. (Left panel) Forest plots displaying the top 10 and bottom 10 motifs by relative ratio. (Right panel) Compositional bias in Violin plot for the top 10 motifs in *Helicobacter pylori* genomes.

(B) *H. pylori*-negative STAD. The average relative ratio is shown as a blue dot, and the 95% CI as whiskers.
