# Supplementary material for: Helicobacter pylori base-excision restriction enzyme in stomach carcinogenesis
Source: PNAS Nexus. 2025 Aug 5;4(8):pgaf244. doi: 10.1093/pnasnexus/pgaf244 (PMC12366791; doi:10.1093/pnasnexus/pgaf244)
Supplement: pgaf244_Supplementary_Data [file pgaf244_supplementary_data.zip › PNASNEXUS-PNASNEXUS-2024-00952RR-s10.pdf]

Fig. S9

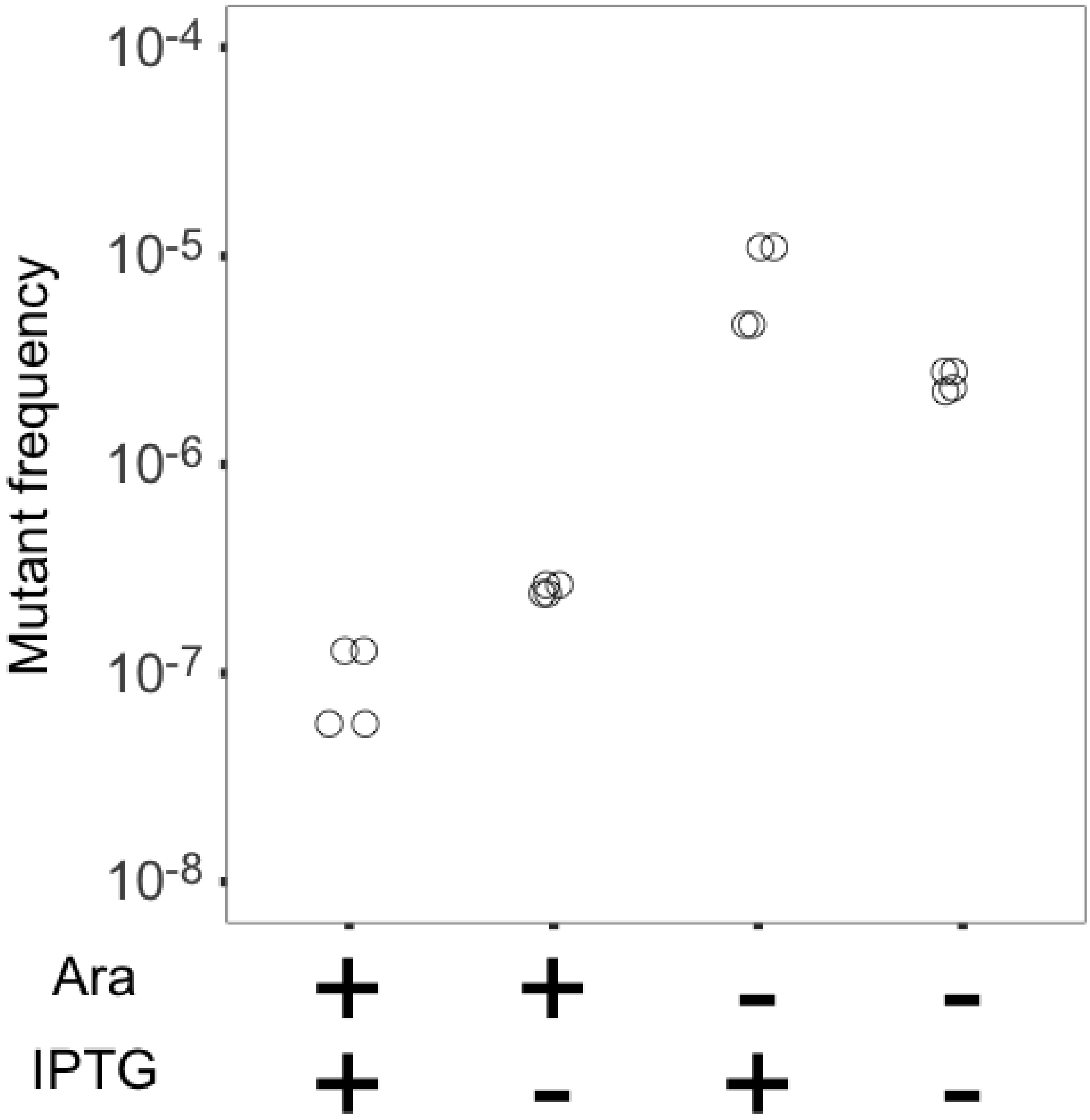

Fig. S9. Mutagenesis by PabI from *Pyrococcus abyssi*.

For methods and presentation, see Figure 5A(i). pYF46 (= pET28a:: *pabIR*) [20] was used instead of pET28a::*Hp*PabI.
