# Supplementary material for: Helicobacter pylori base-excision restriction enzyme in stomach carcinogenesis
Source: PNAS Nexus. 2025 Aug 5;4(8):pgaf244. doi: 10.1093/pnasnexus/pgaf244 (PMC12366791; doi:10.1093/pnasnexus/pgaf244)
Supplement: pgaf244_Supplementary_Data [file pgaf244_supplementary_data.zip › PNASNEXUS-PNASNEXUS-2024-00952RR-s11.pdf]

Fig. S10

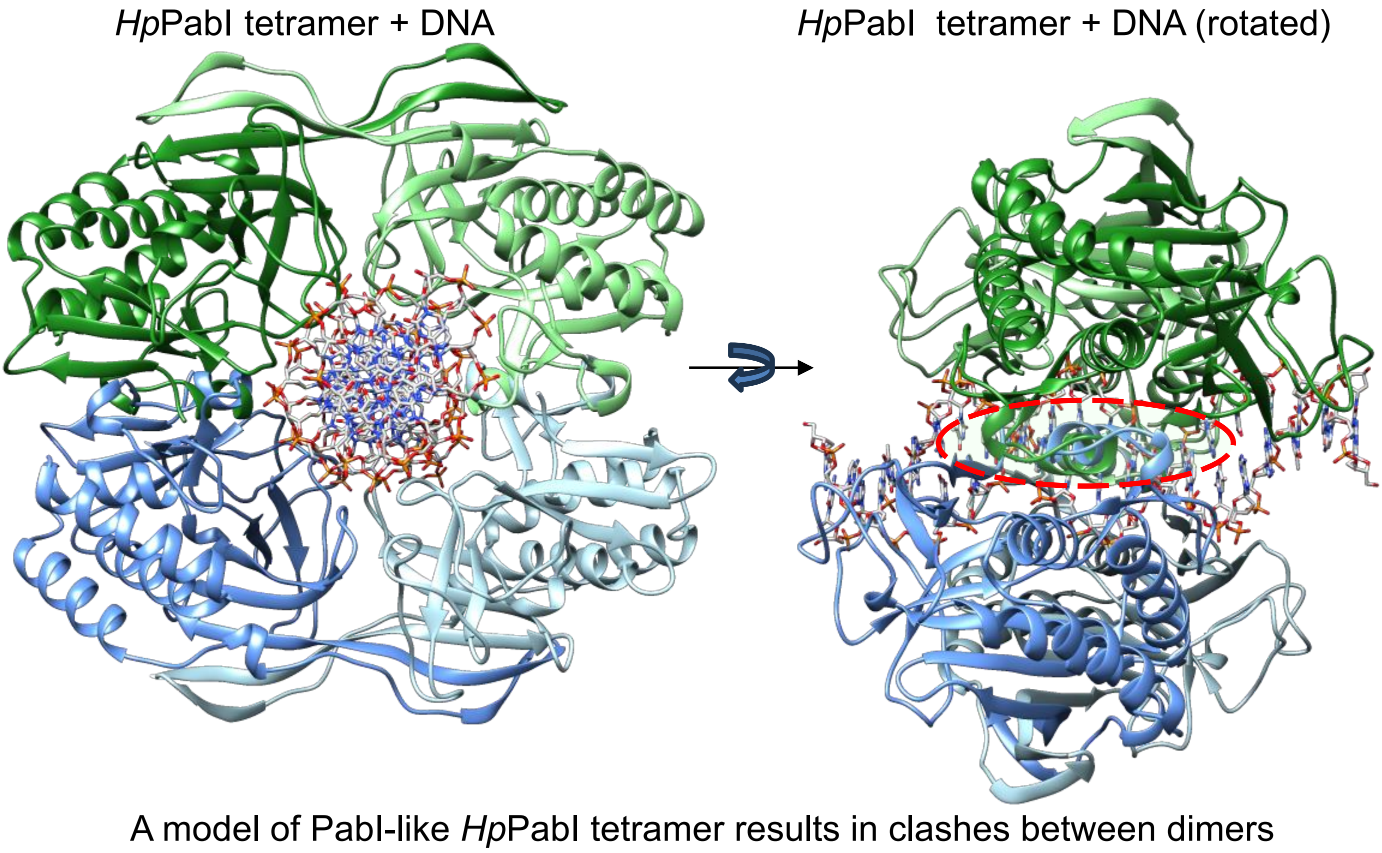


**Fig. S10. Hypothetical HpPabI tetramer on DNA.**

PabI tetramer modelled after PabI bound to non-specific DNA (PDB: 5iff). The red ellipse indicates an overlap of the dimers.
