# Supplementary material for: Helicobacter pylori base-excision restriction enzyme in stomach carcinogenesis
Source: PNAS Nexus. 2025 Aug 5;4(8):pgaf244. doi: 10.1093/pnasnexus/pgaf244 (PMC12366791; doi:10.1093/pnasnexus/pgaf244)
Supplement: pgaf244_Supplementary_Data [file pgaf244_supplementary_data.zip › PNASNEXUS-PNASNEXUS-2024-00952RR-s12.pdf]

Fig. S11

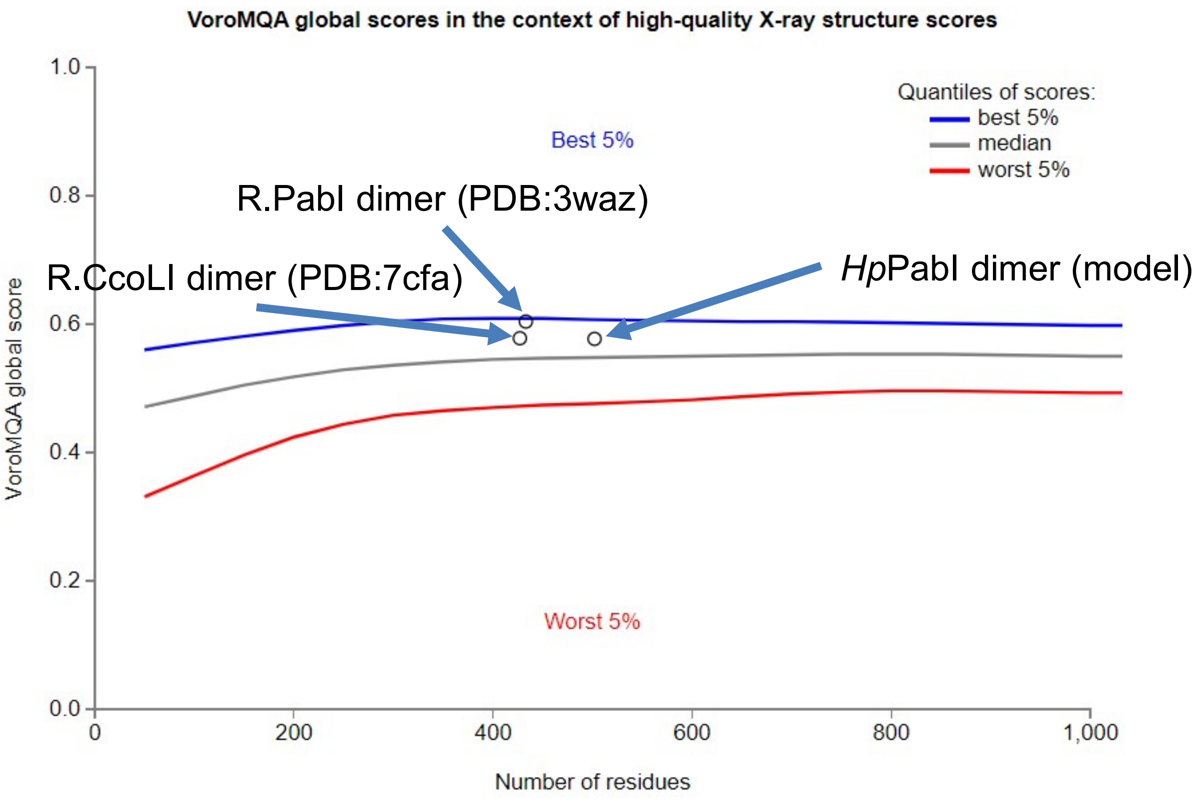

Fig. S11. Assessment of HpPabI structure modelling.

The energy scores for the HpPabI dimer are comparable to those for the X-ray structures of PabI (PDB: 3waz) and CcoLI, a PabI homolog from Campylobacter coli (PDB: 7cfa).
