# Supplementary material for: Helicobacter pylori base-excision restriction enzyme in stomach carcinogenesis
Source: PNAS Nexus. 2025 Aug 5;4(8):pgaf244. doi: 10.1093/pnasnexus/pgaf244 (PMC12366791; doi:10.1093/pnasnexus/pgaf244)
Supplement: pgaf244_Supplementary_Data [file pgaf244_supplementary_data.zip › PNASNEXUS-PNASNEXUS-2024-00952RR-s13.pdf]

Fig. S12.

A

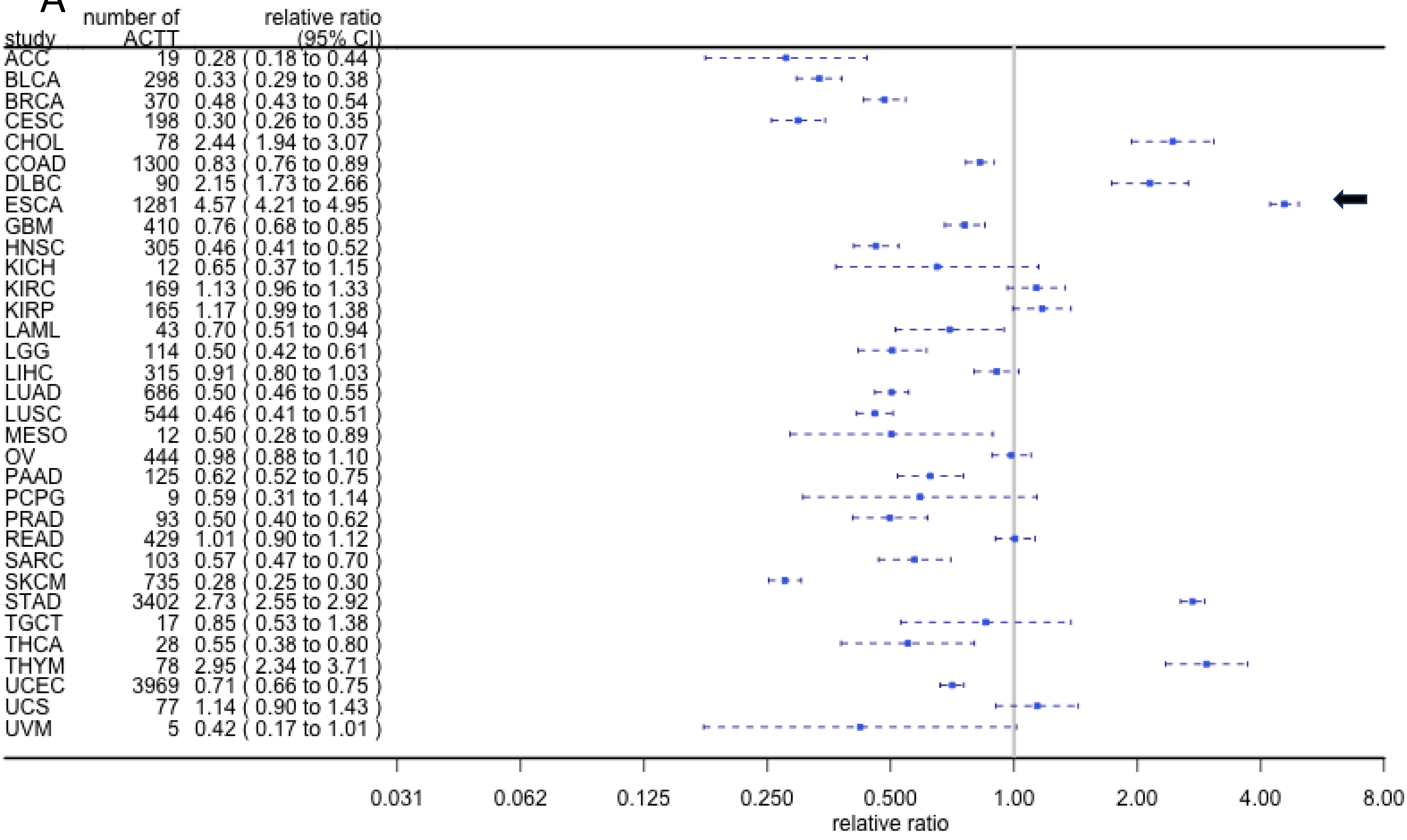
study
number of ACTT
relative ratio (95% CI)
ACC 19 0.28 (0.18 to 0.44)
BLCA 298 0.33 (0.29 to 0.38)
BRCA 370 0.48 (0.43 to 0.54)
CESC 198 0.30 (0.26 to 0.35)
CHOL 78 2.44 (1.94 to 3.07)
COAD 1300 0.83 (0.76 to 0.89)
DLBC 90 2.15 (1.73 to 2.66)
ESCA 1281 4.57 (4.21 to 4.95)
GBM 410 0.76 (0.68 to 0.85)
HNSC 305 0.46 (0.41 to 0.52)
KICH 12 0.65 (0.37 to 1.15)
KIRC 169 1.13 (0.96 to 1.33)
KIRP 165 1.17 (0.99 to 1.38)
LAML 43 0.70 (0.51 to 0.94)
LGG 114 0.50 (0.42 to 0.61)
LIHC 315 0.91 (0.80 to 1.03)
LUAD 686 0.50 (0.46 to 0.55)
LUSC 544 0.46 (0.41 to 0.51)
MESO 12 0.50 (0.28 to 0.89)
OV 444 0.98 (0.88 to 1.10)
PAAD 125 0.62 (0.52 to 0.75)
PCPG 9 0.59 (0.31 to 1.14)
PRAD 93 0.50 (0.40 to 0.62)
READ 429 1.01 (0.90 to 1.12)
SARC 103 0.57 (0.47 to 0.70)
SKCM 735 0.28 (0.25 to 0.30)
STAD 3402 2.73 (2.55 to 2.92)
TGCT 17 0.85 (0.53 to 1.38)
THCA 28 0.55 (0.38 to 0.80)
THYM 78 2.95 (2.34 to 3.71)
UCEC 3969 0.71 (0.66 to 0.75)
UCS 77 1.14 (0.90 to 1.43)
UVM 5 0.42 (0.17 to 1.01)
0.031
0.062
0.125
0.250
0.500
1.00
2.00
4.00
8.00
relative ratio

B

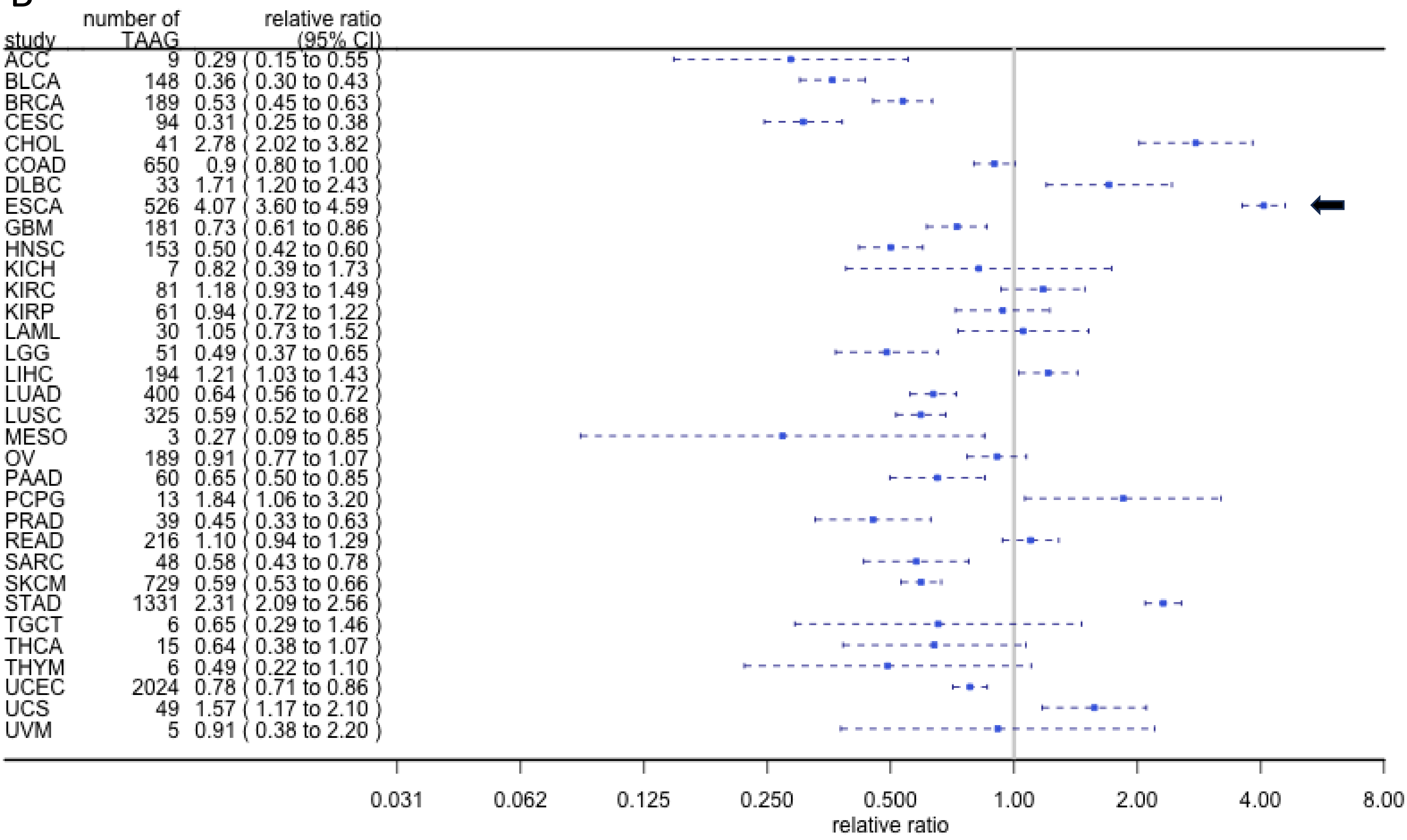
study
number of TAAG
relative ratio (95% CI)
ACC 9 0.29 (0.15 to 0.55)
BLCA 148 0.36 (0.30 to 0.43)
BRCA 189 0.53 (0.45 to 0.63)
CESC 94 0.31 (0.25 to 0.38)
CHOL 41 2.78 (2.02 to 3.82)
COAD 650 0.9 (0.80 to 1.00)
DLBC 33 1.71 (1.20 to 2.43)
ESCA 526 4.07 (3.60 to 4.59)
GBM 181 0.73 (0.61 to 0.86)
HNSC 153 0.50 (0.42 to 0.60)
KICH 7 0.82 (0.39 to 1.73)
KIRC 81 1.18 (0.93 to 1.49)
KIRP 61 0.94 (0.72 to 1.22)
LAML 30 1.05 (0.73 to 1.52)
LGG 51 0.49 (0.37 to 0.65)
LIHC 194 1.21 (1.03 to 1.43)
LUAD 400 0.64 (0.56 to 0.72)
LUSC 325 0.59 (0.52 to 0.68)
MESO 3 0.27 (0.09 to 0.85)
OV 189 0.91 (0.77 to 1.07)
PAAD 60 0.65 (0.50 to 0.85)
PCPG 13 1.84 (1.06 to 3.20)
PRAD 39 0.45 (0.33 to 0.63)
READ 216 1.10 (0.94 to 1.29)
SARC 48 0.58 (0.43 to 0.78)
SKCM 729 0.59 (0.53 to 0.66)
STAD 1331 2.31 (2.09 to 2.56)
TGCT 6 0.65 (0.29 to 1.46)
THCA 15 0.64 (0.38 to 1.07)
THYM 6 0.49 (0.22 to 1.10)
UCEC 2024 0.78 (0.71 to 0.86)
UCS 49 1.57 (1.17 to 2.10)
UVM 5 0.91 (0.38 to 2.20)
0.031
0.062
0.125
0.250
0.500
1.00
2.00
4.00
8.00
relative ratio

C

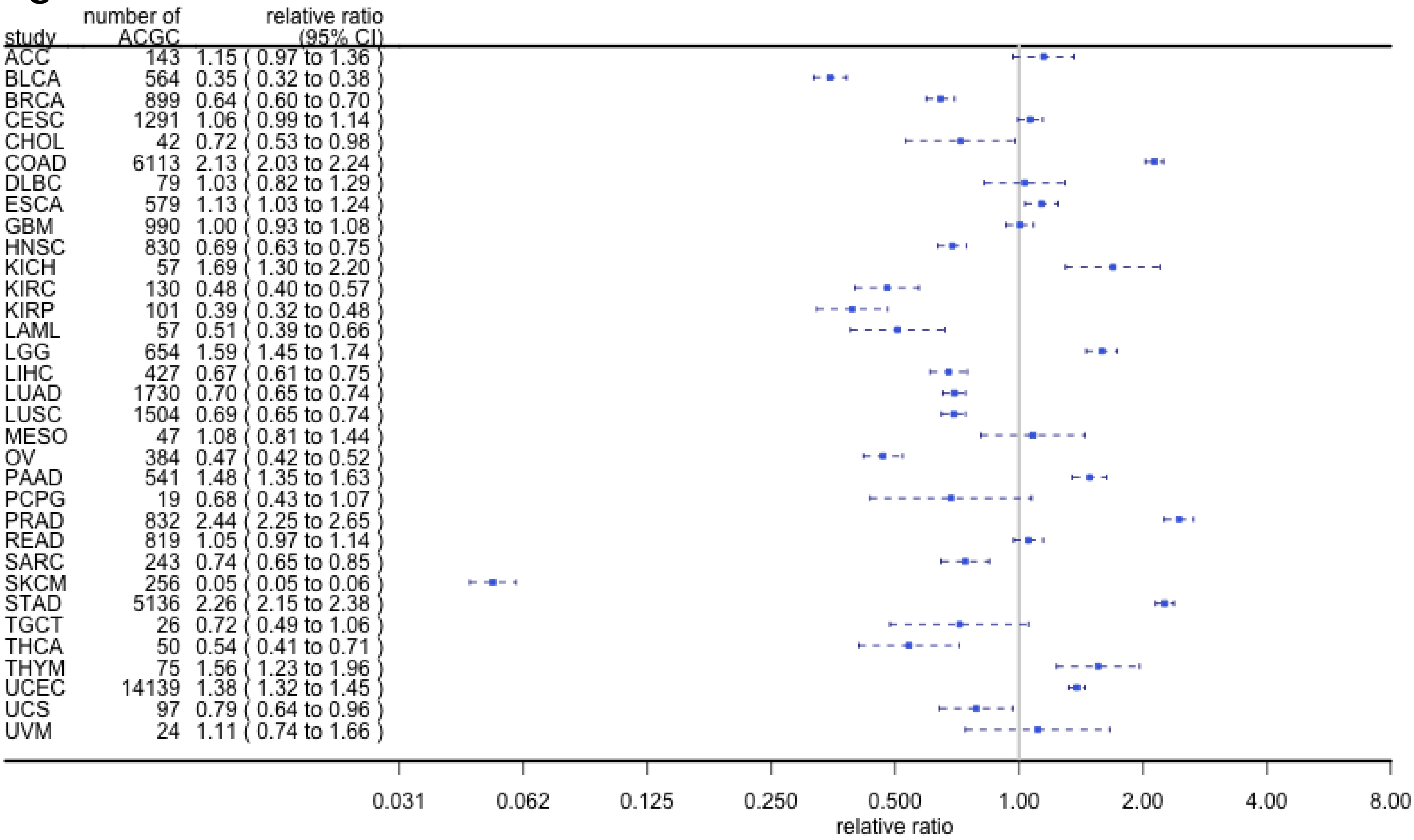
study
number of ACGC
relative ratio (95% CI)
ACC 143 1.15 (0.97 to 1.36)
BLCA 564 0.35 (0.32 to 0.38)
BRCA 899 0.64 (0.60 to 0.70)
CESC 1291 1.06 (0.99 to 1.14)
CHOL 42 0.72 (0.53 to 0.98)
COAD 6113 2.13 (2.03 to 2.24)
DLBC 79 1.03 (0.82 to 1.29)
ESCA 579 1.13 (1.03 to 1.24)
GBM 990 1.00 (0.93 to 1.08)
HNSC 830 0.69 (0.63 to 0.75)
KICH 57 1.69 (1.30 to 2.20)
KIRC 130 0.48 (0.40 to 0.57)
KIRP 101 0.39 (0.32 to 0.48)
LAML 57 0.51 (0.39 to 0.66)
LGG 654 1.59 (1.45 to 1.74)
LIHC 427 0.67 (0.61 to 0.75)
LUAD 1730 0.70 (0.65 to 0.74)
LUSC 1504 0.69 (0.65 to 0.74)
MESO 47 1.08 (0.81 to 1.44)
OV 384 0.47 (0.42 to 0.52)
PAAD 541 1.48 (1.35 to 1.63)
PCPG 19 0.68 (0.43 to 1.07)
PRAD 832 2.44 (2.25 to 2.65)
READ 819 1.05 (0.97 to 1.14)
SARC 243 0.74 (0.65 to 0.85)
SKCM 256 0.05 (0.05 to 0.06)
STAD 5136 2.26 (2.15 to 2.38)
TGCT 26 0.72 (0.49 to 1.06)
THCA 50 0.54 (0.41 to 0.71)
THYM 75 1.56 (1.23 to 1.96)
UCEC 14139 1.38 (1.32 to 1.45)
UCS 97 0.79 (0.64 to 0.96)
UVM 24 1.11 (0.74 to 1.66)
0.031 0.062 0.125 0.250 0.500 1.00 2.00 4.00 8.00
relative ratio

D

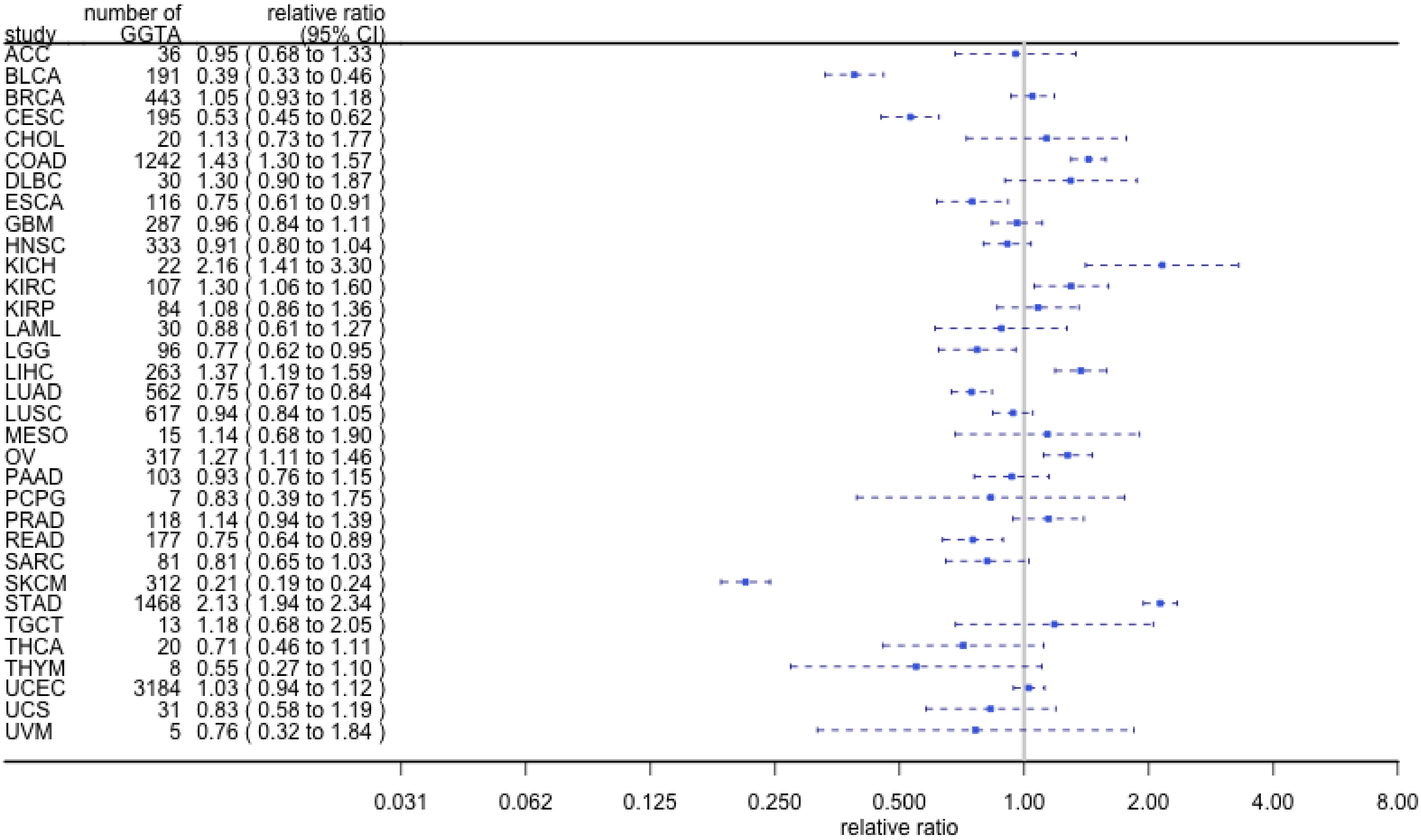
study
number of GGTA
relative ratio (95% CI)
ACC 36 0.95 (0.68 to 1.33)
BLCA 191 0.39 (0.33 to 0.46)
BRCA 443 1.05 (0.93 to 1.18)
CESC 195 0.53 (0.45 to 0.62)
CHOL 20 1.13 (0.73 to 1.77)
COAD 1242 1.43 (1.30 to 1.57)
DLBC 30 1.30 (0.90 to 1.87)
ESCA 116 0.75 (0.61 to 0.91)
GBM 287 0.96 (0.84 to 1.11)
HNSC 333 0.91 (0.80 to 1.04)
KICH 22 2.16 (1.41 to 3.30)
KIRC 107 1.30 (1.06 to 1.60)
KIRP 84 1.08 (0.86 to 1.36)
LAML 30 0.88 (0.61 to 1.27)
LGG 96 0.77 (0.62 to 0.95)
LIHC 263 1.37 (1.19 to 1.59)
LUAD 562 0.75 (0.67 to 0.84)
LUSC 617 0.94 (0.84 to 1.05)
MESO 15 1.14 (0.68 to 1.90)
OV 317 1.27 (1.11 to 1.46)
PAAD 103 0.93 (0.76 to 1.15)
PCPG 7 0.83 (0.39 to 1.75)
PRAD 118 1.14 (0.94 to 1.39)
READ 177 0.75 (0.64 to 0.89)
SARC 81 0.81 (0.65 to 1.03)
SKCM 312 0.21 (0.19 to 0.24)
STAD 1468 2.13 (1.94 to 2.34)
TGCT 13 1.18 (0.68 to 2.05)
THCA 20 0.71 (0.46 to 1.11)
THYM 8 0.55 (0.27 to 1.10)
UCEC 3184 1.03 (0.94 to 1.12)
UCS 31 0.83 (0.58 to 1.19)
UVM 5 0.76 (0.32 to 1.84)
0.031 0.062 0.125 0.250 0.500 1.00 2.00 4.00 8.00
relative ratio

E

study	number of GAAG	relative ratio (95% CI)
ACC	32	0.45 (0.32 to 0.65)
BLCA	340	0.37 (0.33 to 0.42)
BRCA	472	0.60 (0.54 to 0.67)
CESC	218	0.32 (0.28 to 0.37)
CHOL	62	1.88 (1.46 to 2.43)
COAD	1376	0.85 (0.79 to 0.92)
DLBC	72	1.67 (1.31 to 2.12)
ESCA	871	3.02 (2.76 to 3.30)
GBM	607	1.09 (0.99 to 1.20)
HNSC	482	0.71 (0.64 to 0.79)
KICH	21	1.10 (0.72 to 1.70)
KIRC	237	1.54 (1.34 to 1.77)
KIRP	257	1.77 (1.55 to 2.03)
LAML	85	1.34 (1.07 to 1.67)
LGG	171	0.74 (0.63 to 0.86)
LIHC	483	1.35 (1.21 to 1.50)
LUAD	912	0.65 (0.60 to 0.71)
LUSC	884	0.72 (0.66 to 0.79)
MESO	16	0.65 (0.40 to 1.07)
OV	715	1.54 (1.40 to 1.69)
PAAD	128	0.62 (0.52 to 0.75)
PCPG	13	0.83 (0.48 to 1.43)
PRAD	102	0.53 (0.43 to 0.65)
READ	373	0.85 (0.76 to 0.95)
SARC	126	0.68 (0.57 to 0.82)
SKCM	1269	0.46 (0.43 to 0.50)
STAD	2690	2.09 (1.95 to 2.24)
TGCT	16	0.78 (0.48 to 1.28)
THCA	37	0.71 (0.51 to 0.98)
THYM	21	0.77 (0.50 to 1.19)
UCEC	3954	0.68 (0.64 to 0.73)
UCS	74	1.06 (0.84 to 1.34)
UVM	7	0.57 (0.27 to 1.20)

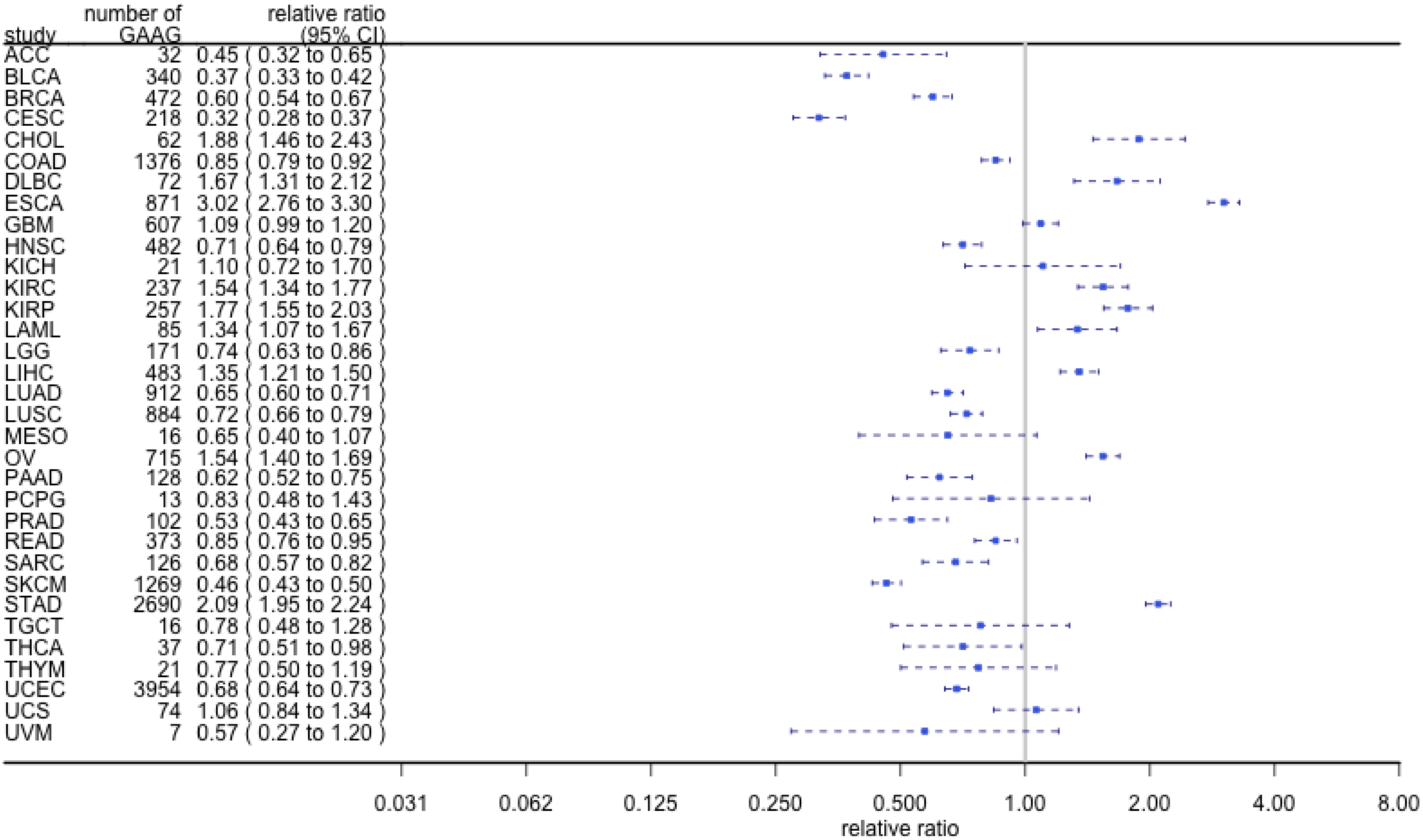

F

study	number of TGCG	relative ratio (95% CI)
ACC	207	1.02 (0.88 to 1.17)
BLCA	942	0.36 (0.33 to 0.38)
BRCA	1426	0.63 (0.59 to 0.67)
CESC	2057	1.04 (0.98 to 1.10)
CHOL	53	0.56 (0.42 to 0.73)
COAD	9279	1.98 (1.90 to 2.06)
DLBC	134	1.07 (0.90 to 1.28)
ESCA	801	0.96 (0.89 to 1.04)
GBM	1747	1.09 (1.02 to 1.15)
HNSC	1403	0.71 (0.67 to 0.76)
KICH	96	1.75 (1.42 to 2.14)
KIRC	209	0.47 (0.41 to 0.54)
KIRP	185	0.44 (0.38 to 0.51)
LAML	93	0.51 (0.41 to 0.62)
LGG	1124	1.67 (1.56 to 1.79)
LIHC	621	0.60 (0.55 to 0.65)
LUAD	2214	0.55 (0.52 to 0.58)
LUSC	1933	0.55 (0.52 to 0.58)
MESO	66	0.93 (0.73 to 1.19)
OV	607	0.45 (0.41 to 0.49)
PAAD	1013	1.70 (1.59 to 1.83)
PCPG	43	0.95 (0.70 to 1.28)
PRAD	1295	2.33 (2.18 to 2.49)
READ	1473	1.16 (1.09 to 1.23)
SARC	391	0.73 (0.66 to 0.81)
SKCM	456	0.06 (0.05 to 0.06)
STAD	7399	1.99 (1.91 to 2.08)
TGCT	49	0.83 (0.62 to 1.10)
THCA	114	0.75 (0.62 to 0.91)
THYM	113	1.44 (1.19 to 1.73)
UCEC	24324	1.45 (1.40 to 1.51)
UCS	189	0.94 (0.81 to 1.09)
UVM	53	1.50 (1.14 to 1.97)

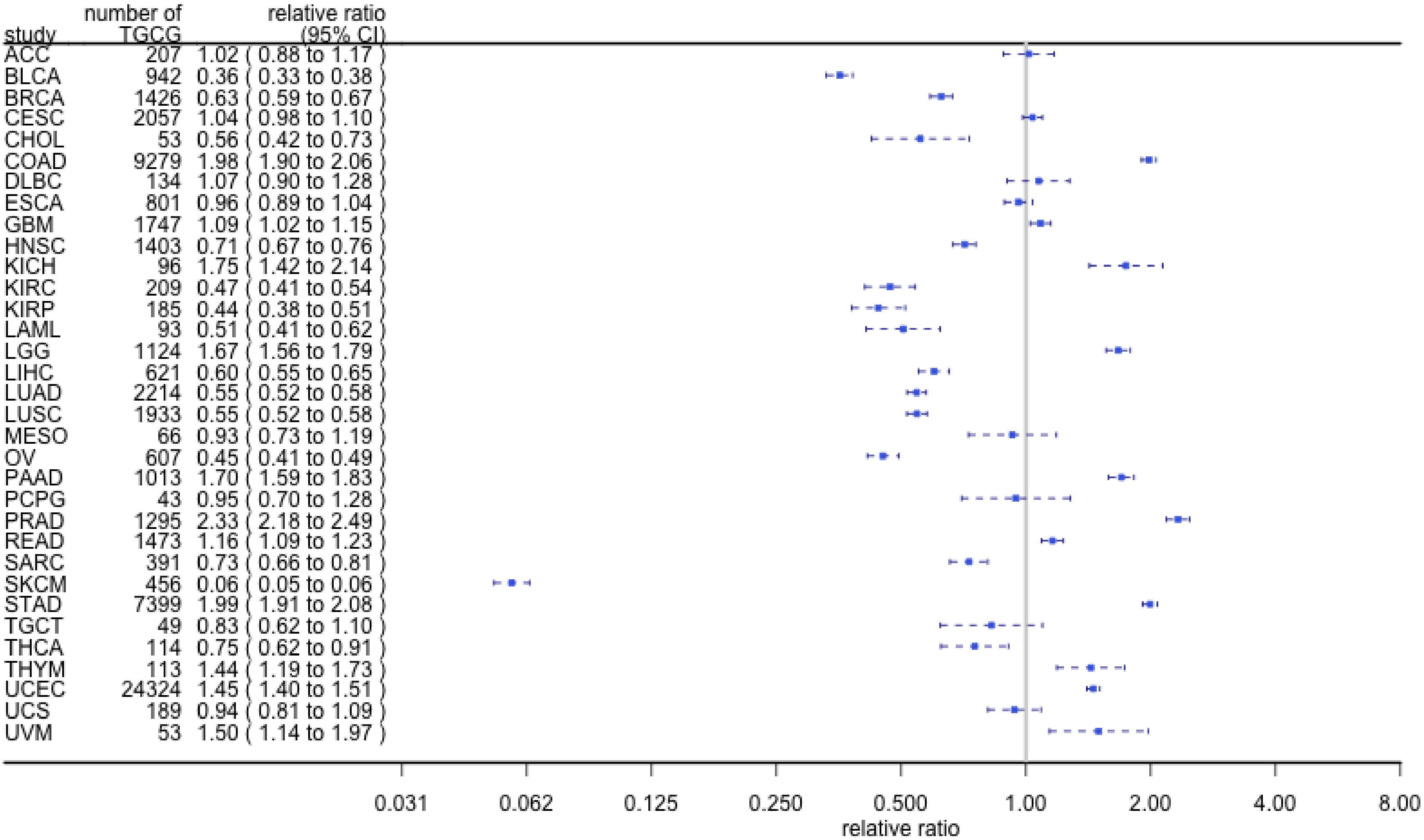

G

study	number of GGCG	relative ratio (95% CI)
ACC	235	0.95 (0.83 to 1.08)
BLCA	1119	0.35 (0.32 to 0.37)
BRCA	1621	0.58 (0.55 to 0.62)
CESC	2459	1.02 (0.97 to 1.07)
CHOL	85	0.73 (0.59 to 0.91)
COAD	10479	1.84 (1.77 to 1.90)
DLBC	166	1.09 (0.93 to 1.28)
ESCA	1119	1.10 (1.03 to 1.18)
GBM	2373	1.21 (1.15 to 1.27)
HNSC	1540	0.64 (0.60 to 0.68)
KICH	137	2.04 (1.72 to 2.43)
KIRC	262	0.48 (0.43 to 0.55)
KIRP	246	0.48 (0.42 to 0.55)
LAML	116	0.52 (0.43 to 0.62)
LGG	1372	1.67 (1.57 to 1.78)
LIHC	905	0.72 (0.67 to 0.77)
LUAD	2479	0.50 (0.48 to 0.53)
LUSC	2395	0.56 (0.53 to 0.58)
MESO	119	1.37 (1.14 to 1.65)
OV	765	0.47 (0.43 to 0.51)
PAAD	1346	1.86 (1.74 to 1.97)
PCPG	42	0.76 (0.56 to 1.03)
PRAD	1272	1.88 (1.76 to 2.00)
READ	1568	1.01 (0.96 to 1.07)
SARC	563	0.86 (0.79 to 0.94)
SKCM	618	0.06 (0.06 to 0.07)
STAD	8989	1.99 (1.91 to 2.06)
TGCT	49	0.68 (0.51 to 0.90)
THCA	107	0.58 (0.48 to 0.70)
THYM	143	1.49 (1.26 to 1.76)
UCEC	24535	1.20 (1.16 to 1.25)
UCS	207	0.84 (0.73 to 0.97)
UVM	61	1.42 (1.10 to 1.83)

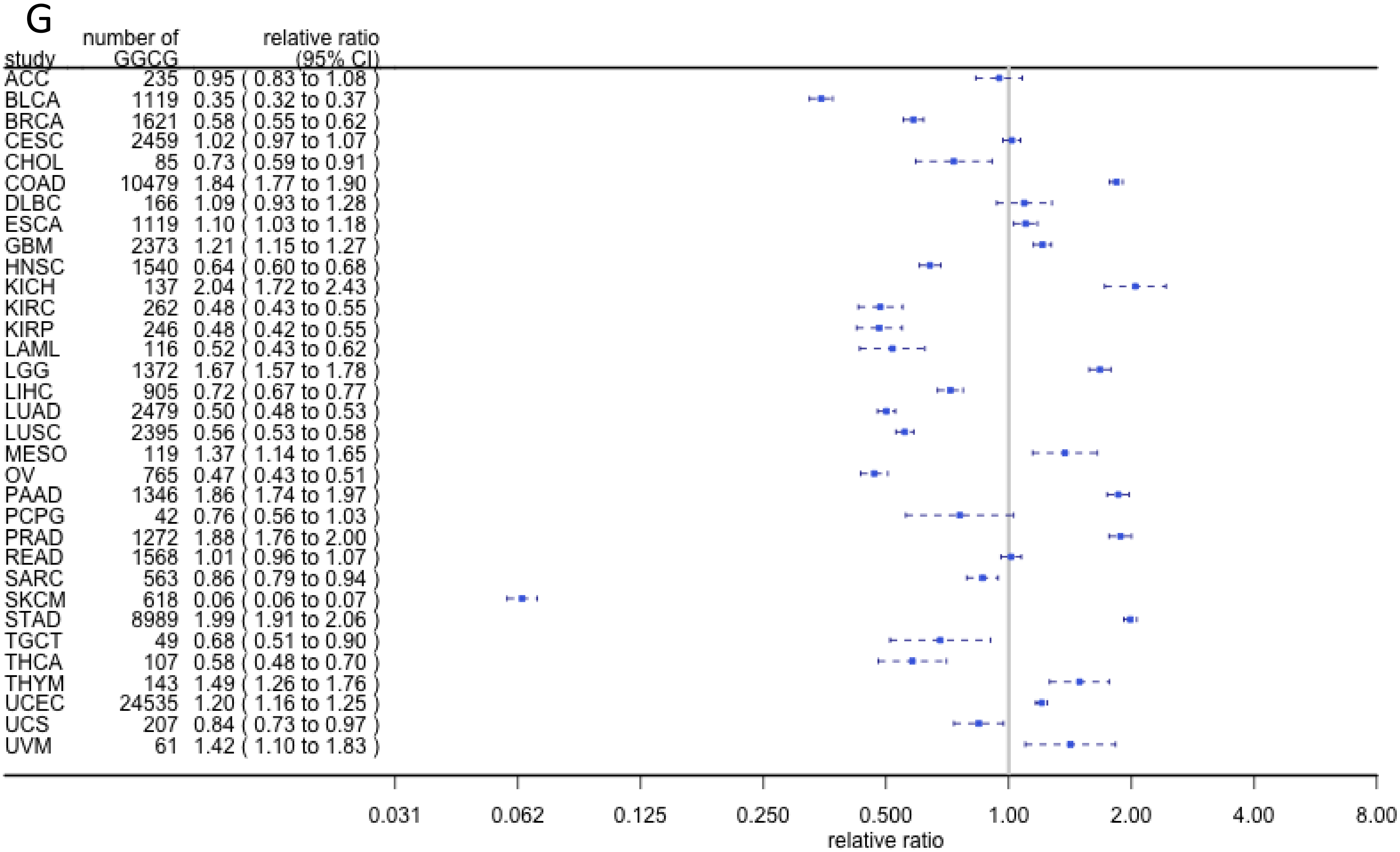

H

study	number of CAAG	relative ratio (95% CI)
ACC	34	0.52 (0.37 to 0.73)
BLCA	345	0.40 (0.36 to 0.45)
BRCA	401	0.54 (0.48 to 0.61)
CESC	176	0.27 (0.23 to 0.32)
CHOL	45	1.46 (1.08 to 1.97)
COAD	1069	0.70 (0.65 to 0.77)
DLBC	62	1.53 (1.19 to 1.98)
ESCA	775	2.87 (2.61 to 3.15)
GBM	429	0.82 (0.74 to 0.92)
HNSC	433	0.68 (0.61 to 0.76)
KICH	16	0.90 (0.55 to 1.47)
KIRC	171	1.19 (1.01 to 1.40)
KIRP	171	1.26 (1.07 to 1.48)
LAML	121	2.03 (1.68 to 2.46)
LGG	155	0.71 (0.60 to 0.84)
LIHC	425	1.27 (1.13 to 1.42)
LUAD	1073	0.82 (0.75 to 0.89)
LUSC	901	0.79 (0.72 to 0.86)
MESO	7	0.30 (0.14 to 0.64)
OV	502	1.16 (1.04 to 1.28)
PAAD	133	0.69 (0.58 to 0.83)
PCPG	9	0.61 (0.32 to 1.18)
PRAD	106	0.59 (0.48 to 0.72)
READ	292	0.71 (0.62 to 0.81)
SARC	118	0.68 (0.56 to 0.82)
SKCM	774	0.30 (0.28 to 0.33)
STAD	2323	1.93 (1.80 to 2.08)
TGCT	15	0.78 (0.47 to 1.30)
THCA	36	0.73 (0.53 to 1.02)
THYM	79	3.10 (2.46 to 3.90)
UCEC	3153	0.58 (0.54 to 0.62)
UCS	59	0.91 (0.70 to 1.18)
UVM	12	1.05 (0.59 to 1.85)

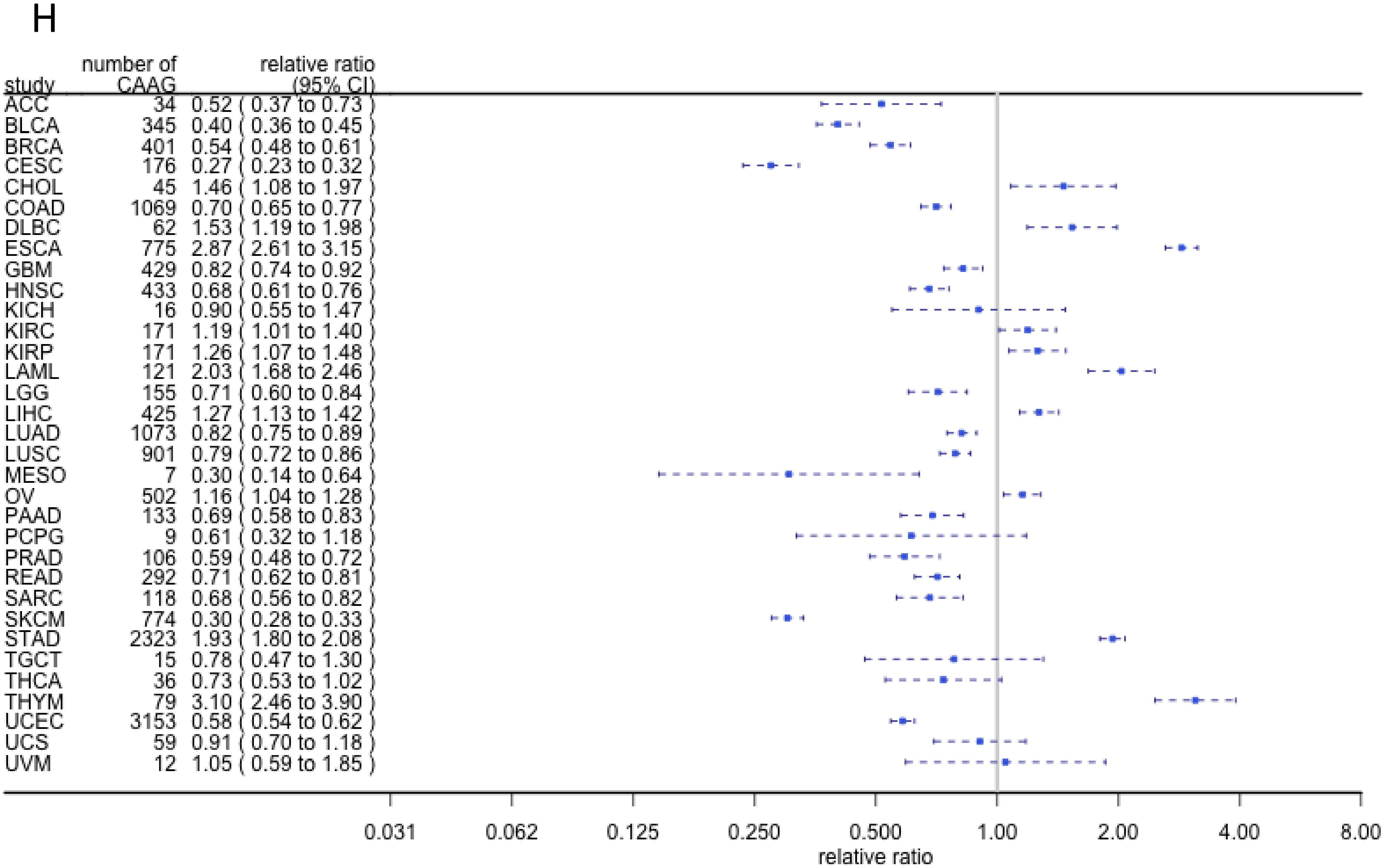

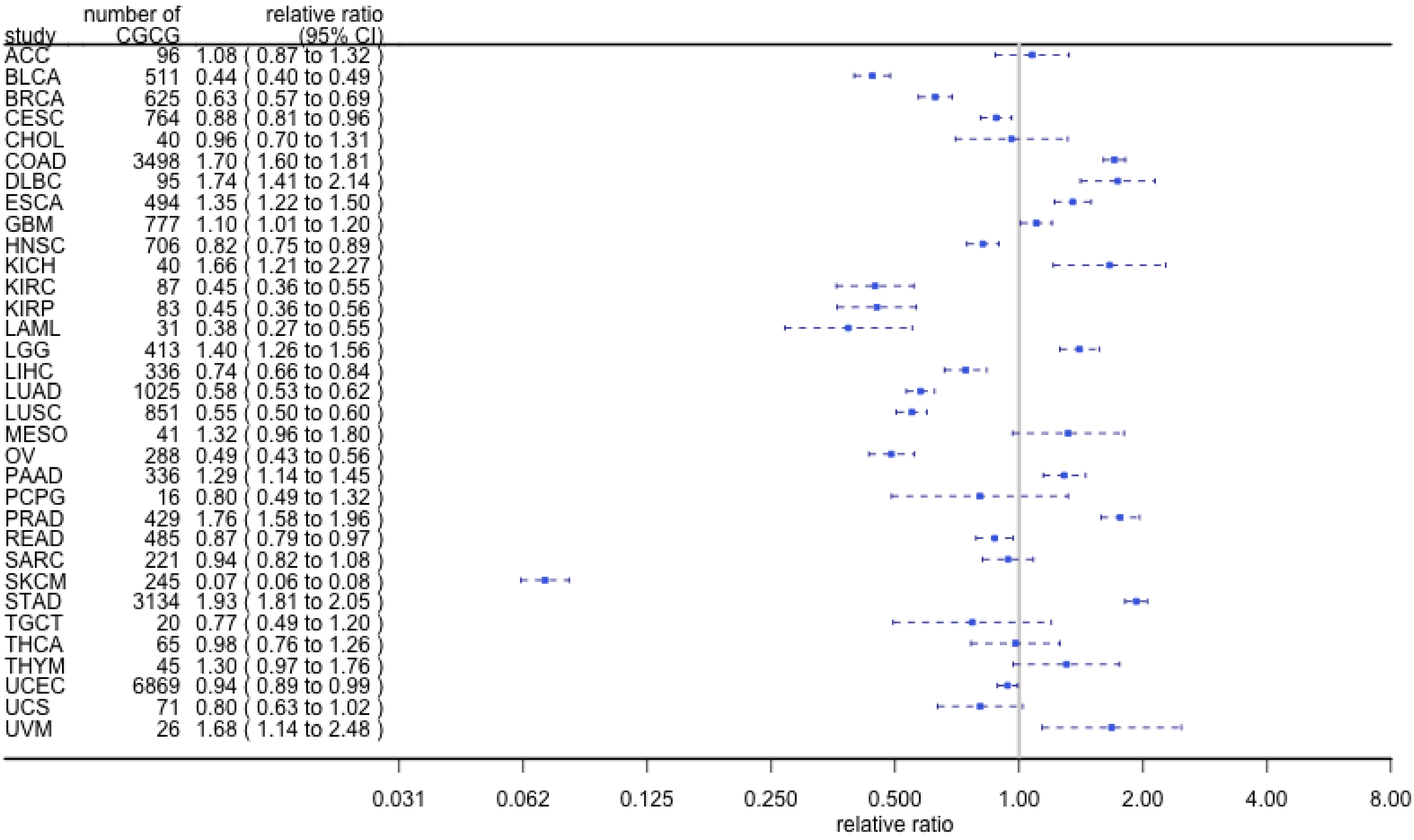

Fig. S12. Forest plots of the top ten 4-mer motifs in all cancer studies.

From TCGA datasets. The average relative ratio is shown as a blue dot, and the 95% CI as whiskers.
