# Supplementary material for: Helicobacter pylori base-excision restriction enzyme in stomach carcinogenesis
Source: PNAS Nexus. 2025 Aug 5;4(8):pgaf244. doi: 10.1093/pnasnexus/pgaf244 (PMC12366791; doi:10.1093/pnasnexus/pgaf244)
Supplement: pgaf244_Supplementary_Data [file pgaf244_supplementary_data.zip › PNASNEXUS-PNASNEXUS-2024-00952RR-s14.pdf]

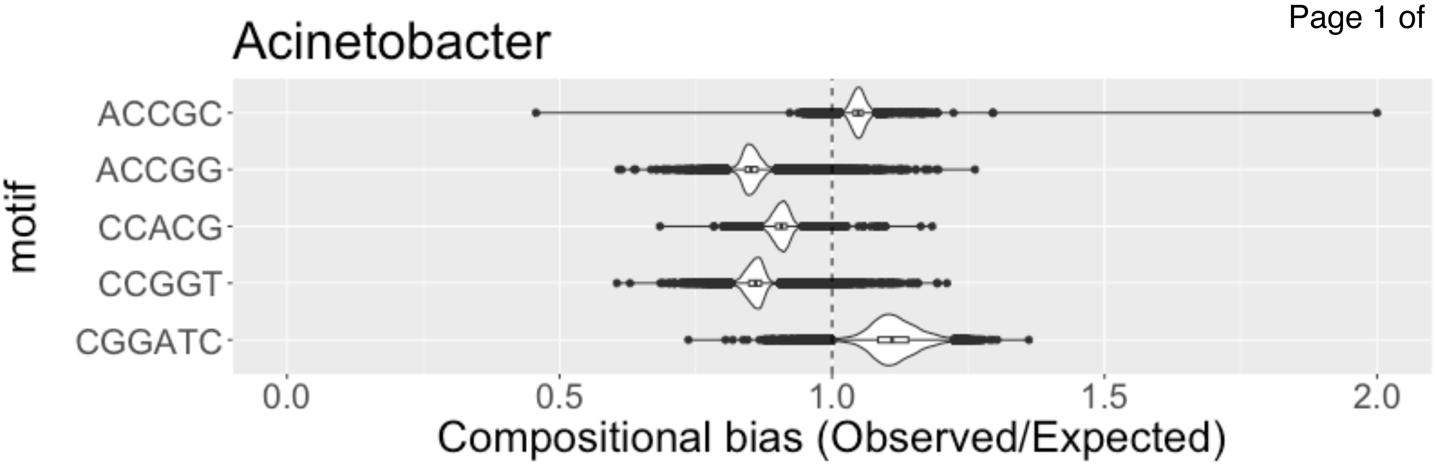
Acinetobacter
motif
ACCGC
ACCGG
CCACG
CCGGT
CGGATC
0.0
0.5
1.0
1.5
2.0
Compositional bias (Observed/Expected)

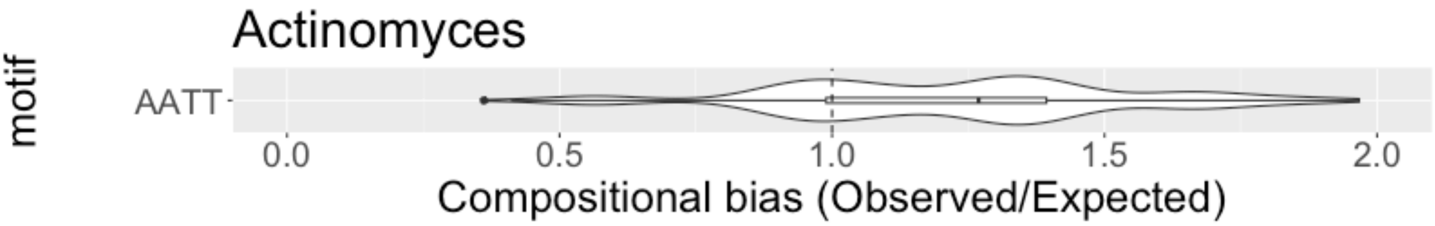
Actinomyces
motif
AATT
0.0
0.5
1.0
1.5
2.0
Compositional bias (Observed/Expected)

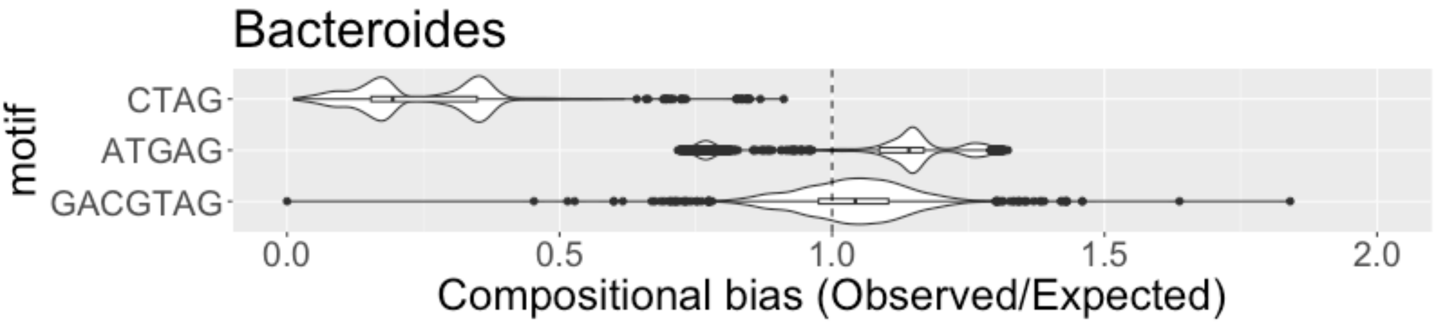
Bacteroides
motif
CTAG
ATGAG
GACGTAG
0.0
0.5
1.0
1.5
2.0
Compositional bias (Observed/Expected)

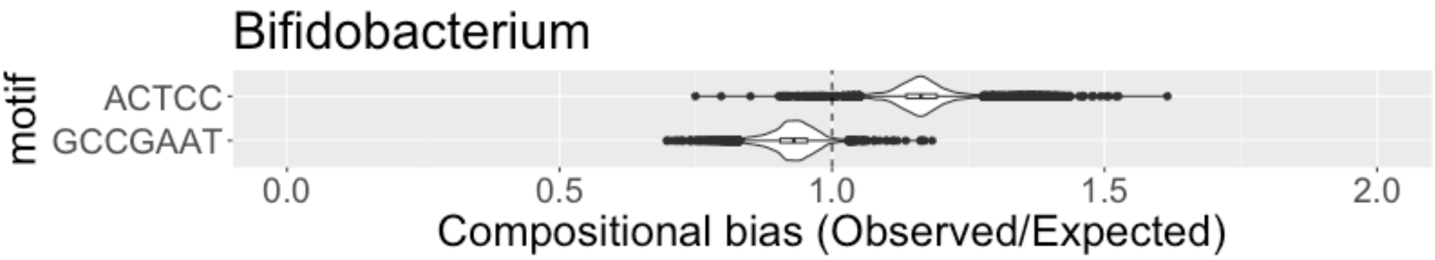
Bifidobacterium
motif
ACTCC
GCCGAAT
0.0
0.5
1.0
1.5
2.0
Compositional bias (Observed/Expected)

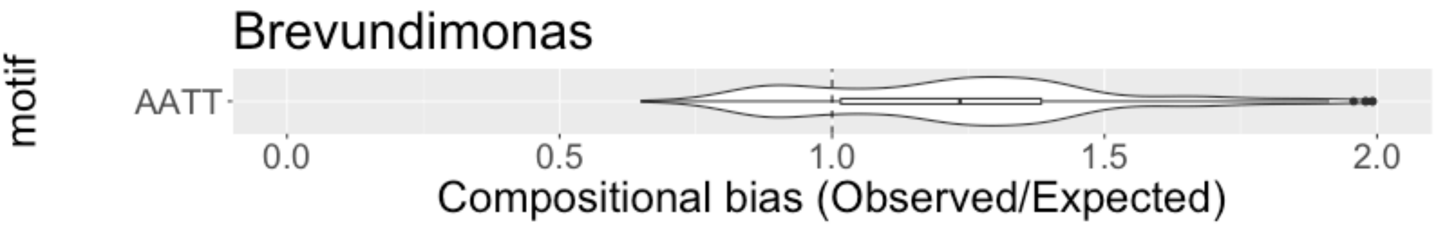
Brevundimonas
motif
AATT
0.0
0.5
1.0
1.5
2.0
Compositional bias (Observed/Expected)

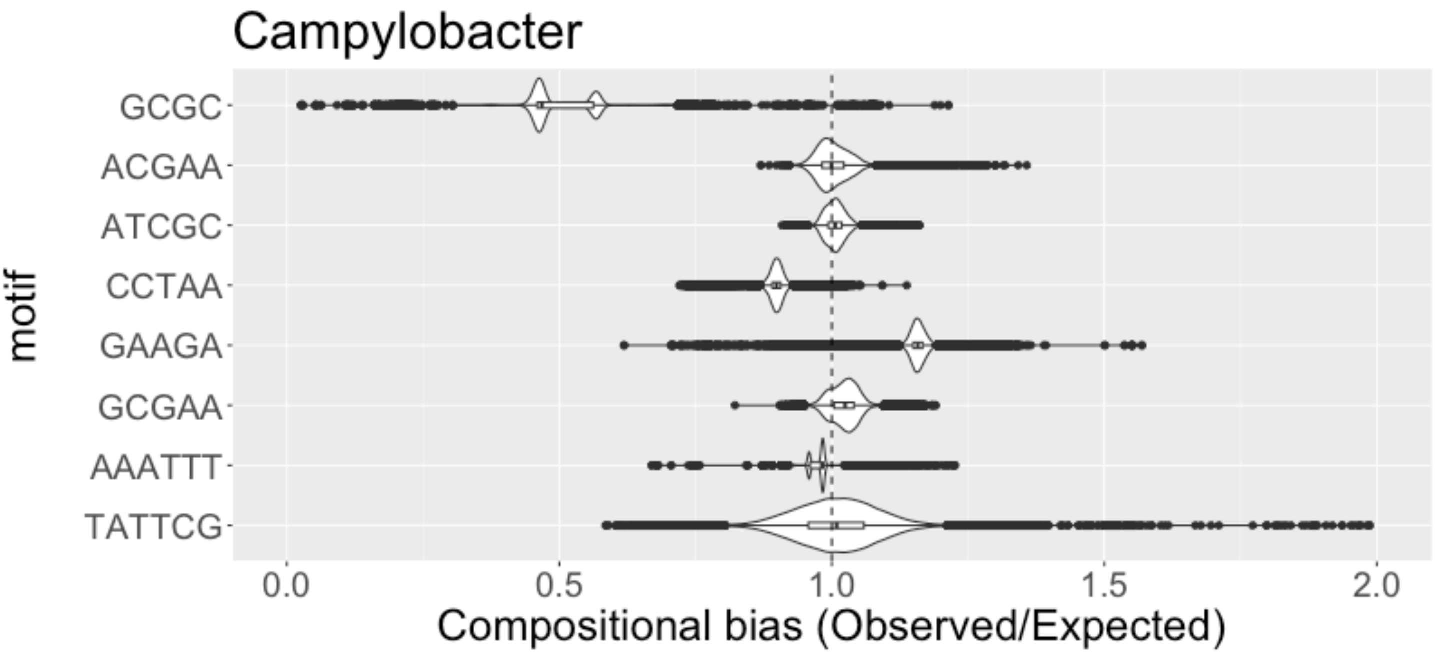
Campylobacter
motif
GCGC
ACGAA
ATCGC
CCTAA
GAAGA
GCGAA
AAATTT
TATTCG
0.0
0.5
1.0
1.5
2.0
Compositional bias (Observed/Expected)

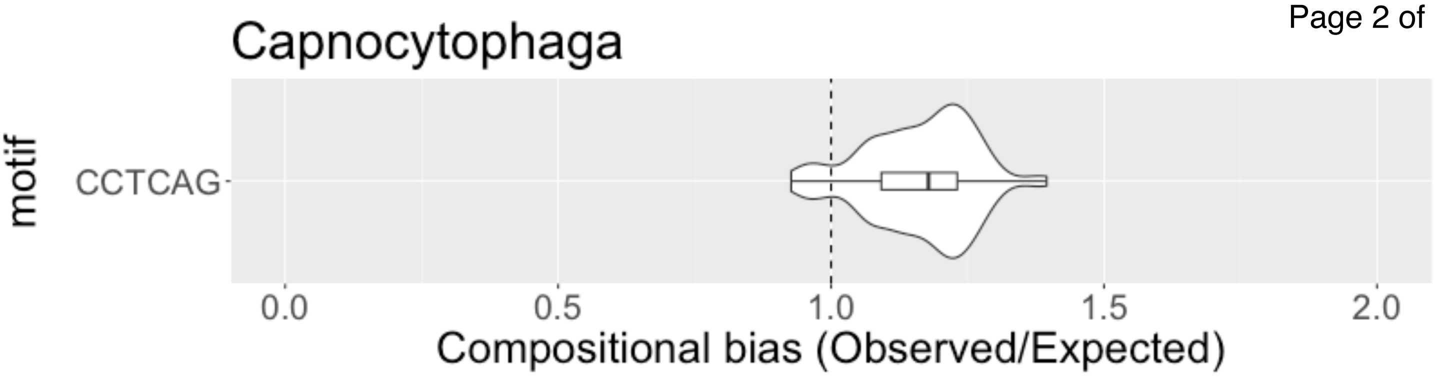
Capnocytophaga
motif
CCTCAG
0.0
0.5
1.0
1.5
2.0
Compositional bias (Observed/Expected)

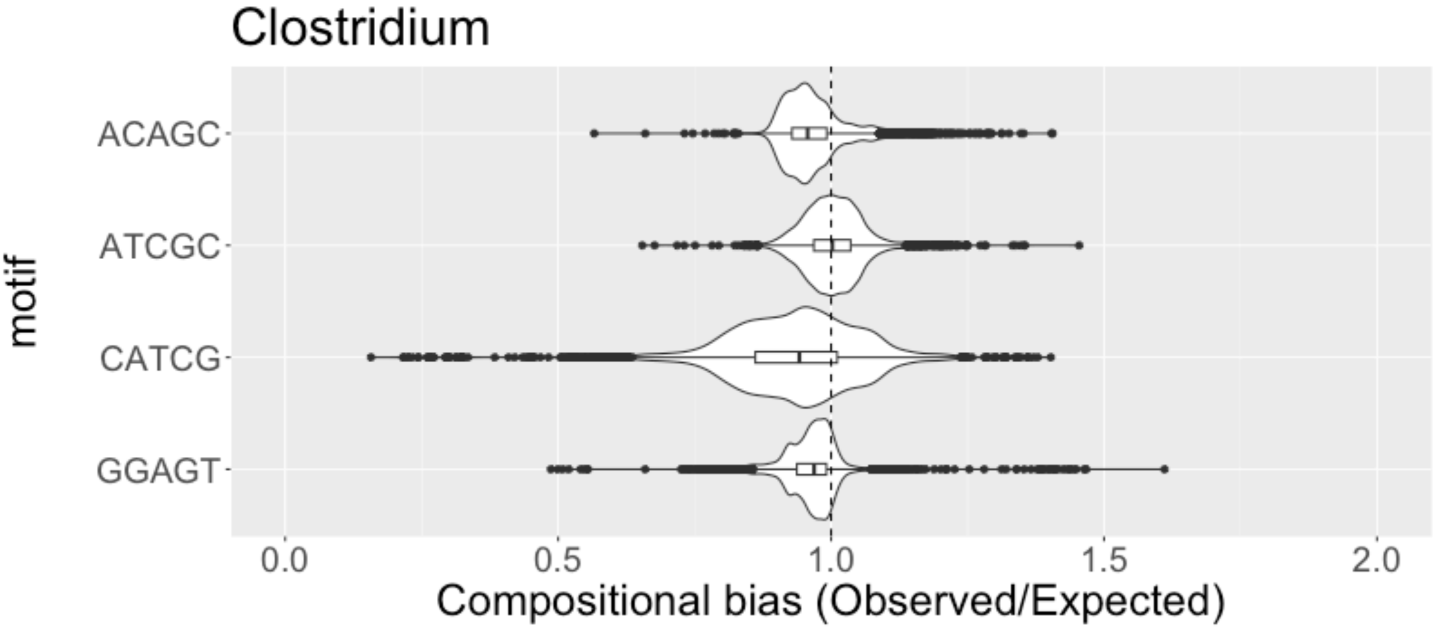
Clostridium
motif
ACAGC
ATCGC
CATCG
GGAGT
0.0
0.5
1.0
1.5
2.0
Compositional bias (Observed/Expected)

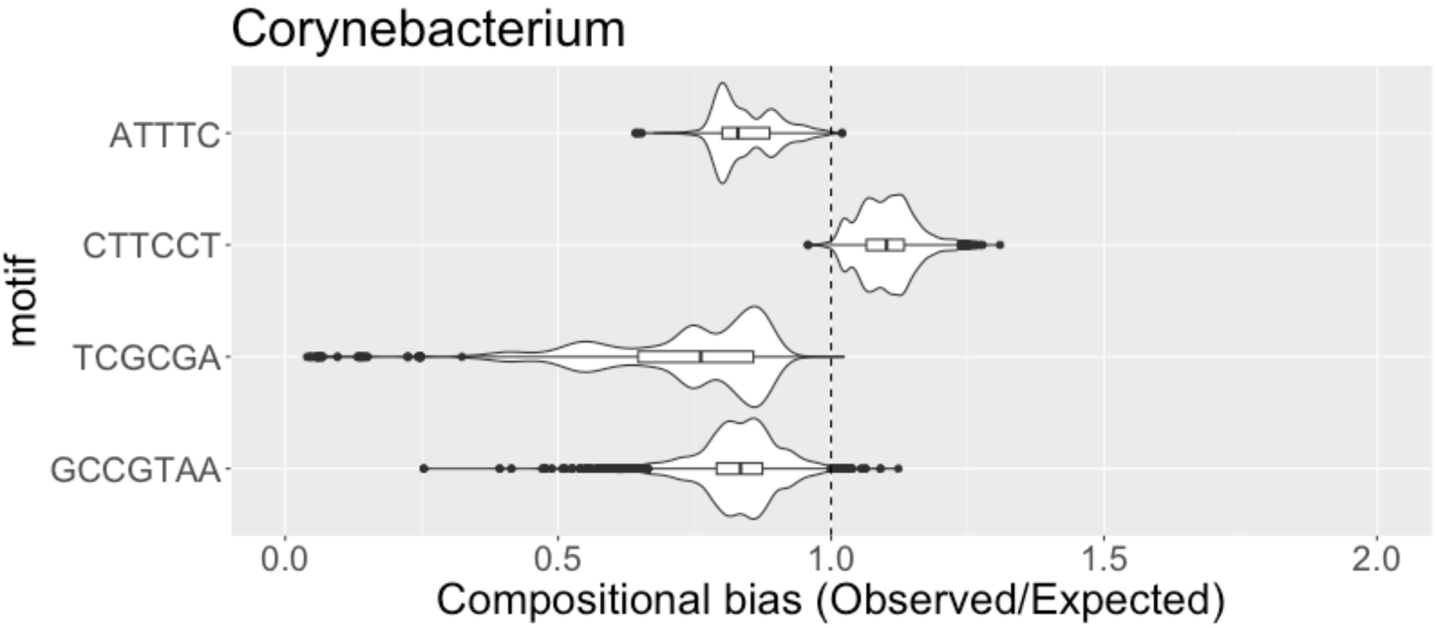
Corynebacterium
motif
ATTTC
CTTCCT
TCGCGA
GCCGTAA
0.0
0.5
1.0
1.5
2.0
Compositional bias (Observed/Expected)

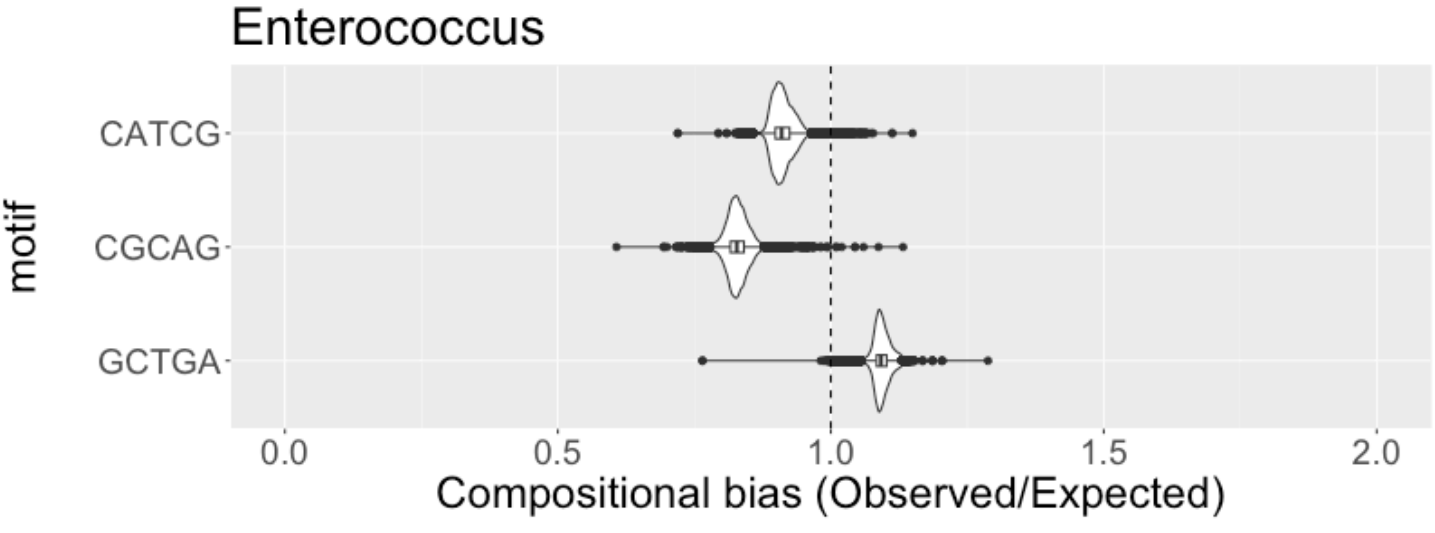
Enterococcus
motif
CATCG
CGCAG
GCTGA
0.0
0.5
1.0
1.5
2.0
Compositional bias (Observed/Expected)

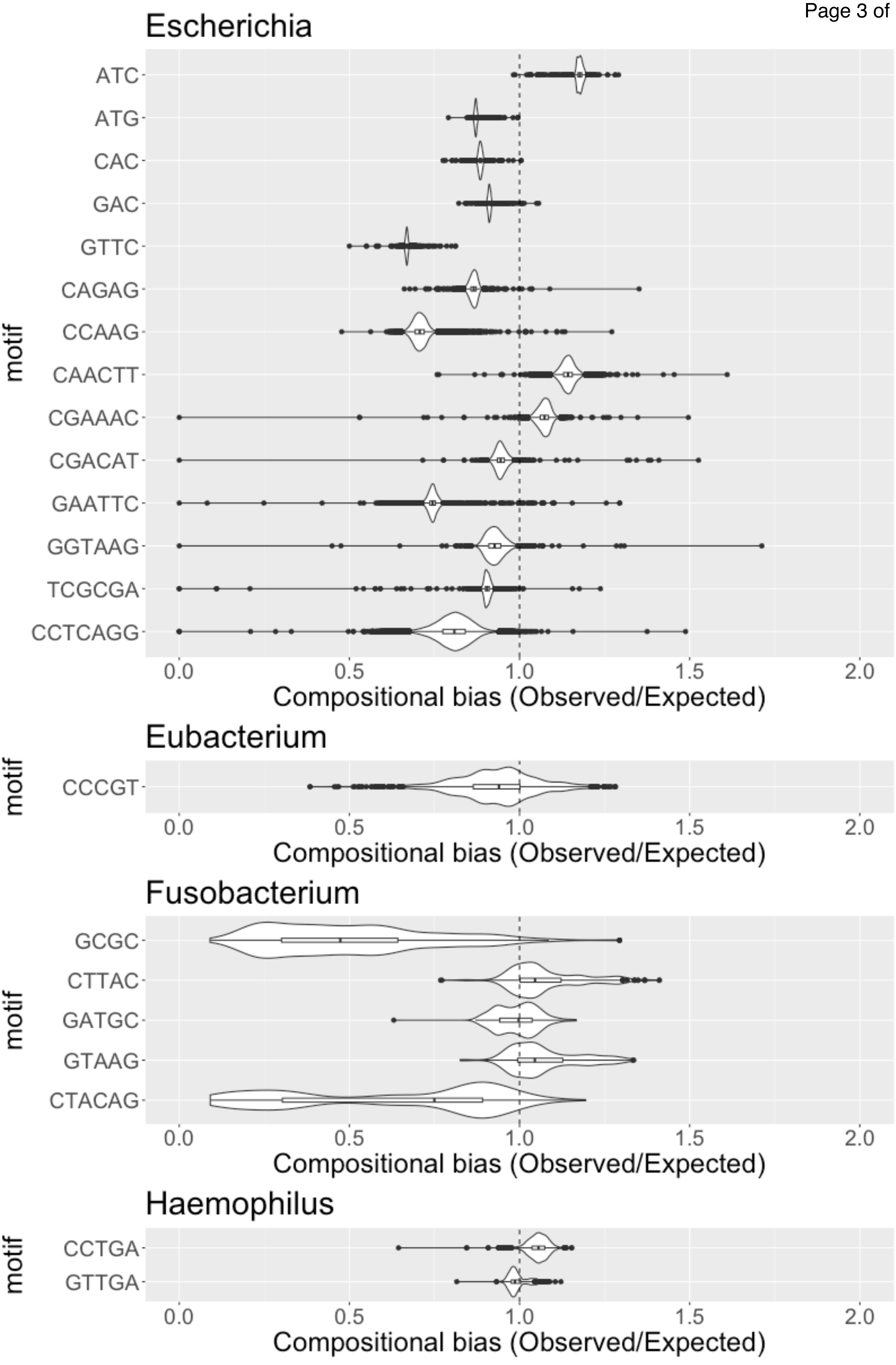
Escherichia
ATC
ATG
CAC
GAC
GTTC
CAGAG
CCAAG
CAACTT
CGAAAC
CGACAT
GAATTC
GGTAAG
TCGCGA
CCTCAGG
motif
0.0
0.5
1.0
1.5
2.0
Compositional bias (Observed/Expected)
Eubacterium
CCCGT
motif
0.0
0.5
1.0
1.5
2.0
Compositional bias (Observed/Expected)
Fusobacterium
GCGC
CTTAC
GATGC
GTAAG
CTACAG
motif
0.0
0.5
1.0
1.5
2.0
Compositional bias (Observed/Expected)
Haemophilus
CCTGA
GTTGA
motif
0.0
0.5
1.0
1.5
2.0
Compositional bias (Observed/Expected)

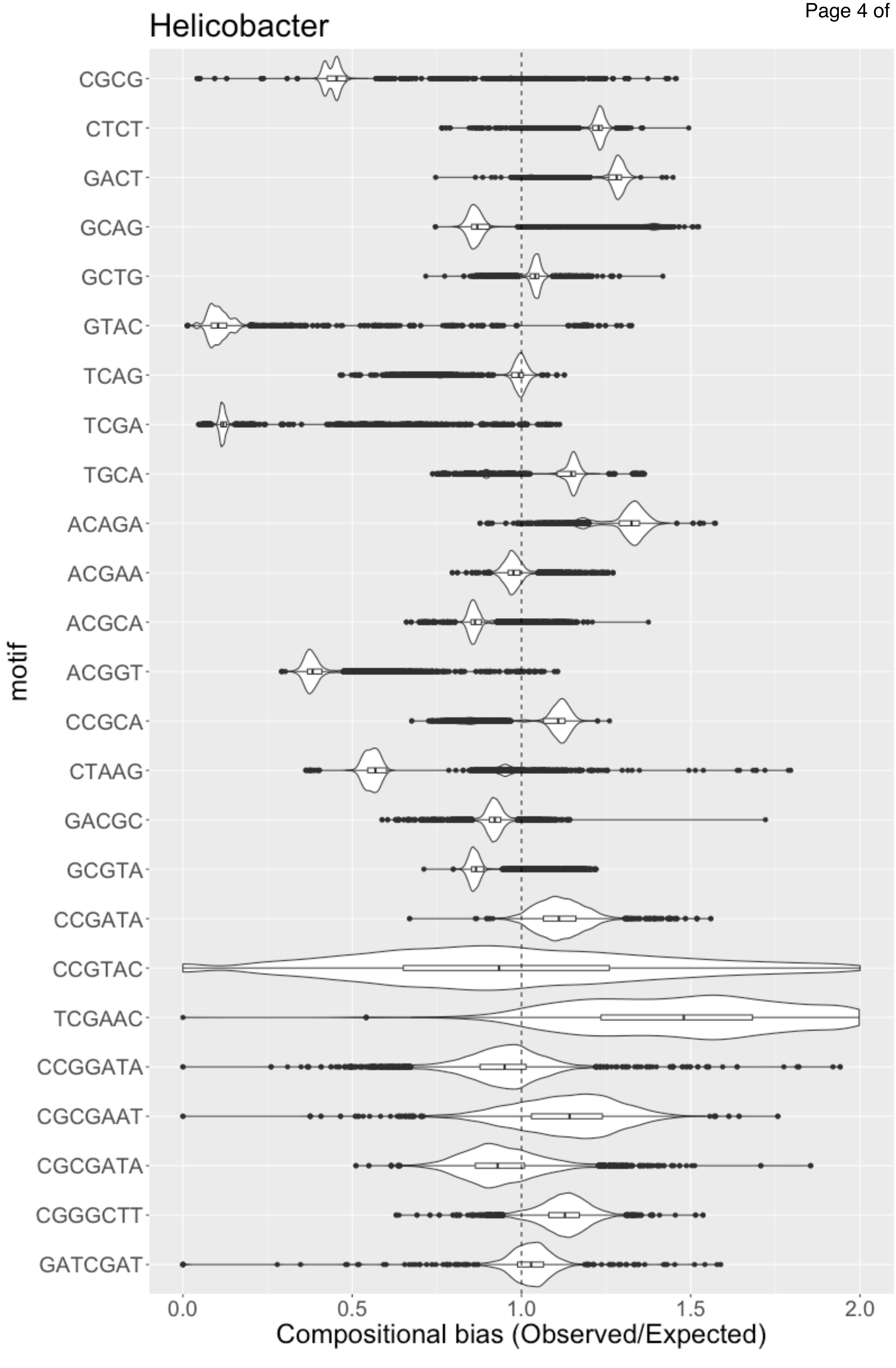
Helicobacter
motif
CGCG
CTCT
GACT
GCAG
GCTG
GTAC
TCAG
TCGA
TGCA
ACAGA
ACGAA
ACGCA
ACGGT
CCGCA
CTAAG
GACGC
GCGTA
CCGATA
CCGTAC
TCGAAC
CCGGATA
CGCGAAT
CGCGATA
CGGGCTT
GATCGAT
0.0
0.5
1.0
1.5
2.0
Compositional bias (Observed/Expected)

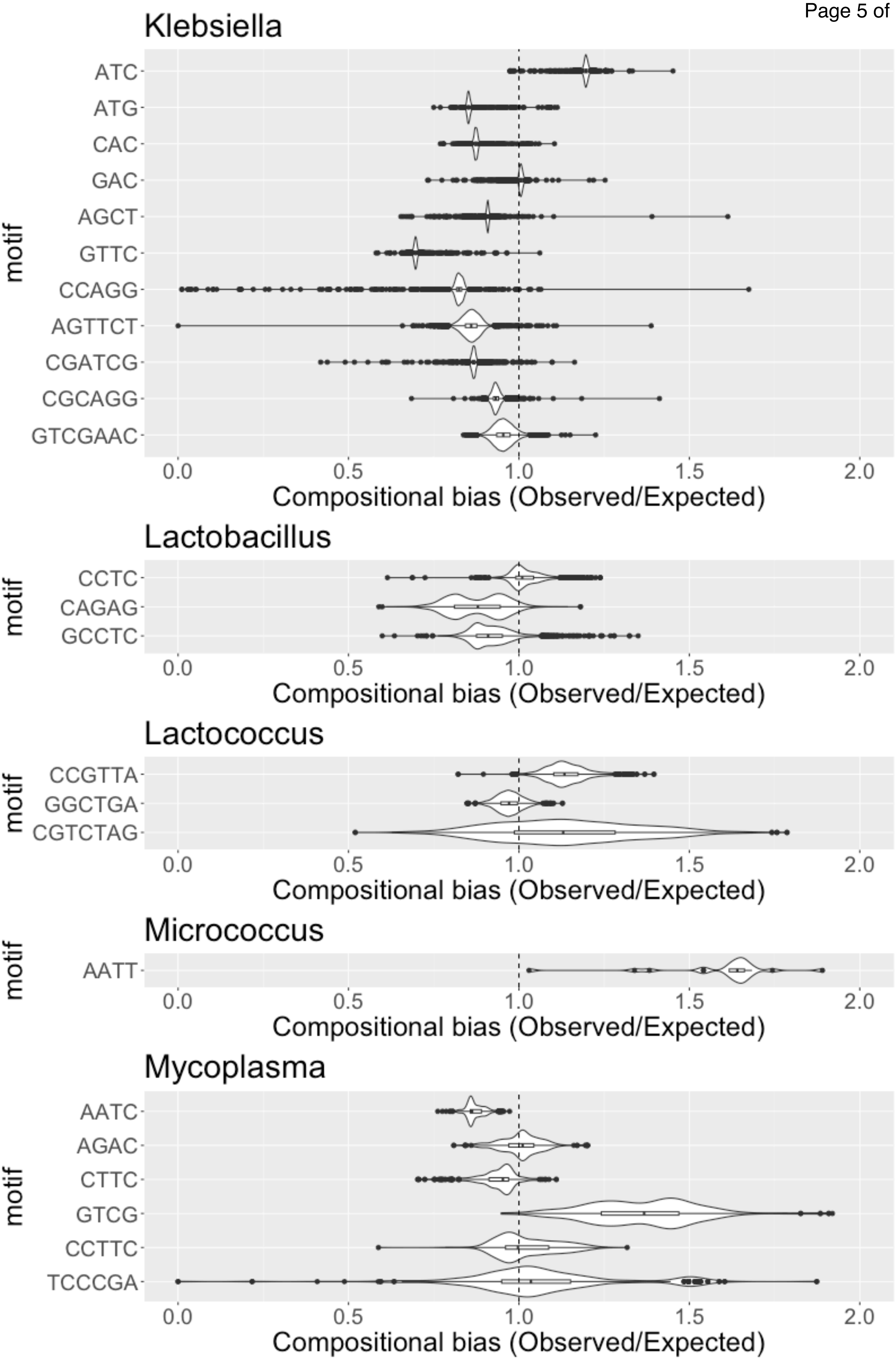
Klebsiella
motif
ATC
ATG
CAC
GAC
AGCT
GTTC
CCAGG
AGTTCT
CGATCG
CGCAGG
GTCGAAC
0.0
0.5
1.0
1.5
2.0
Compositional bias (Observed/Expected)
Lactobacillus
motif
CCTC
CAGAG
GCCTC
0.0
0.5
1.0
1.5
2.0
Compositional bias (Observed/Expected)
Lactococcus
motif
CCGTTA
GGCTGA
CGTCTAG
0.0
0.5
1.0
1.5
2.0
Compositional bias (Observed/Expected)
Micrococcus
motif
AATT
0.0
0.5
1.0
1.5
2.0
Compositional bias (Observed/Expected)
Mycoplasma
motif
AATC
AGAC
CTTC
GTCG
CCTTC
TCCCGA
0.0
0.5
1.0
1.5
2.0
Compositional bias (Observed/Expected)

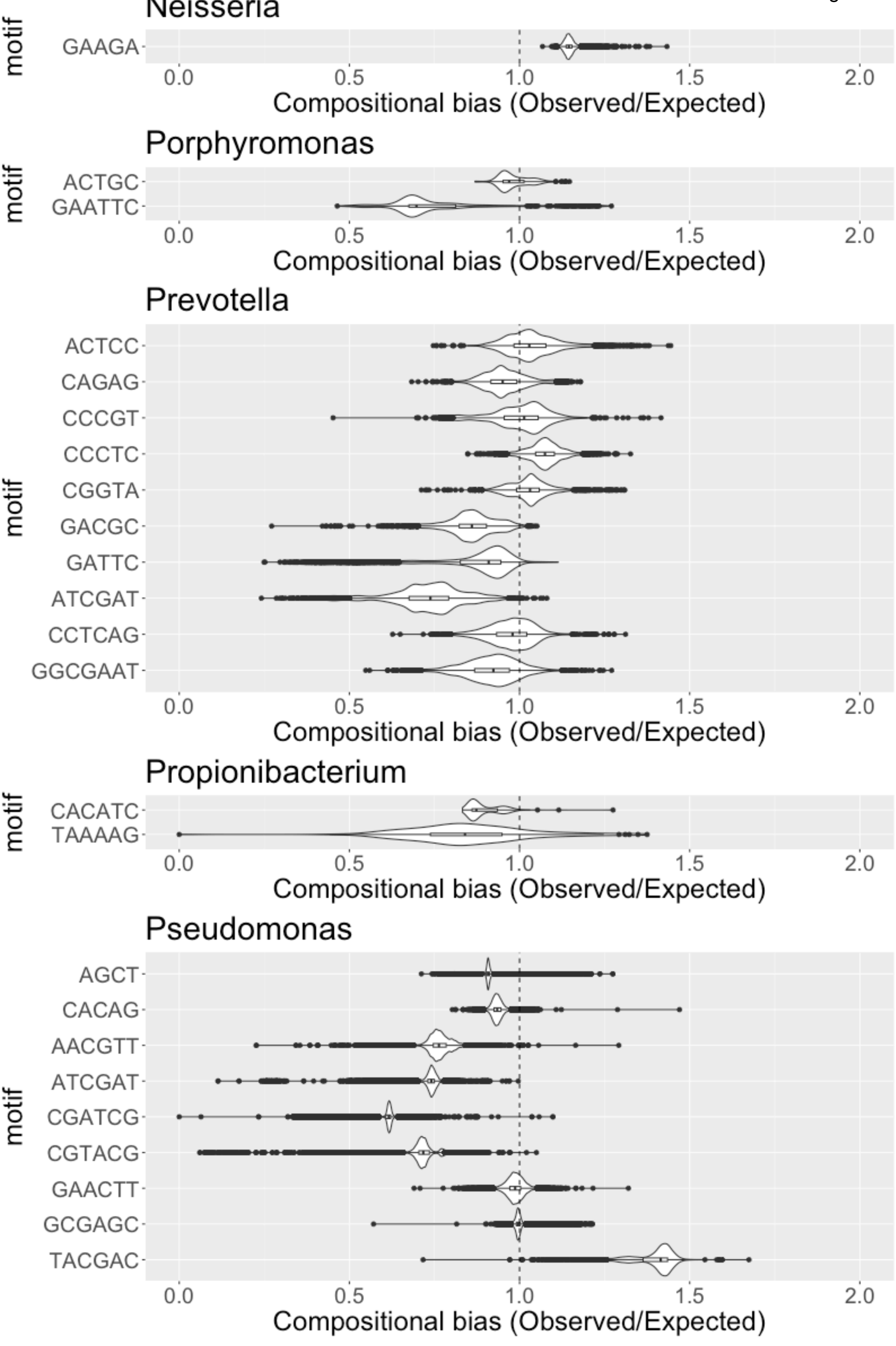
Neisseria
motif
GAAGA
0.0
0.5
1.0
1.5
2.0
Compositional bias (Observed/Expected)
Porphyromonas
motif
ACTGC
GAATTC
0.0
0.5
1.0
1.5
2.0
Compositional bias (Observed/Expected)
Prevotella
motif
ACTCC
CAGAG
CCCGT
CCCTC
CGGTA
GACGC
GATTC
ATCGAT
CCTCAG
GGCGAAT
0.0
0.5
1.0
1.5
2.0
Compositional bias (Observed/Expected)
Propionibacterium
motif
CACATC
TAAAAG
0.0
0.5
1.0
1.5
2.0
Compositional bias (Observed/Expected)
Pseudomonas
motif
AGCT
CACAG
AACGTT
ATCGAT
CGATCG
CGTACG
GAACTT
GCGAGC
TACGAC
0.0
0.5
1.0
1.5
2.0
Compositional bias (Observed/Expected)

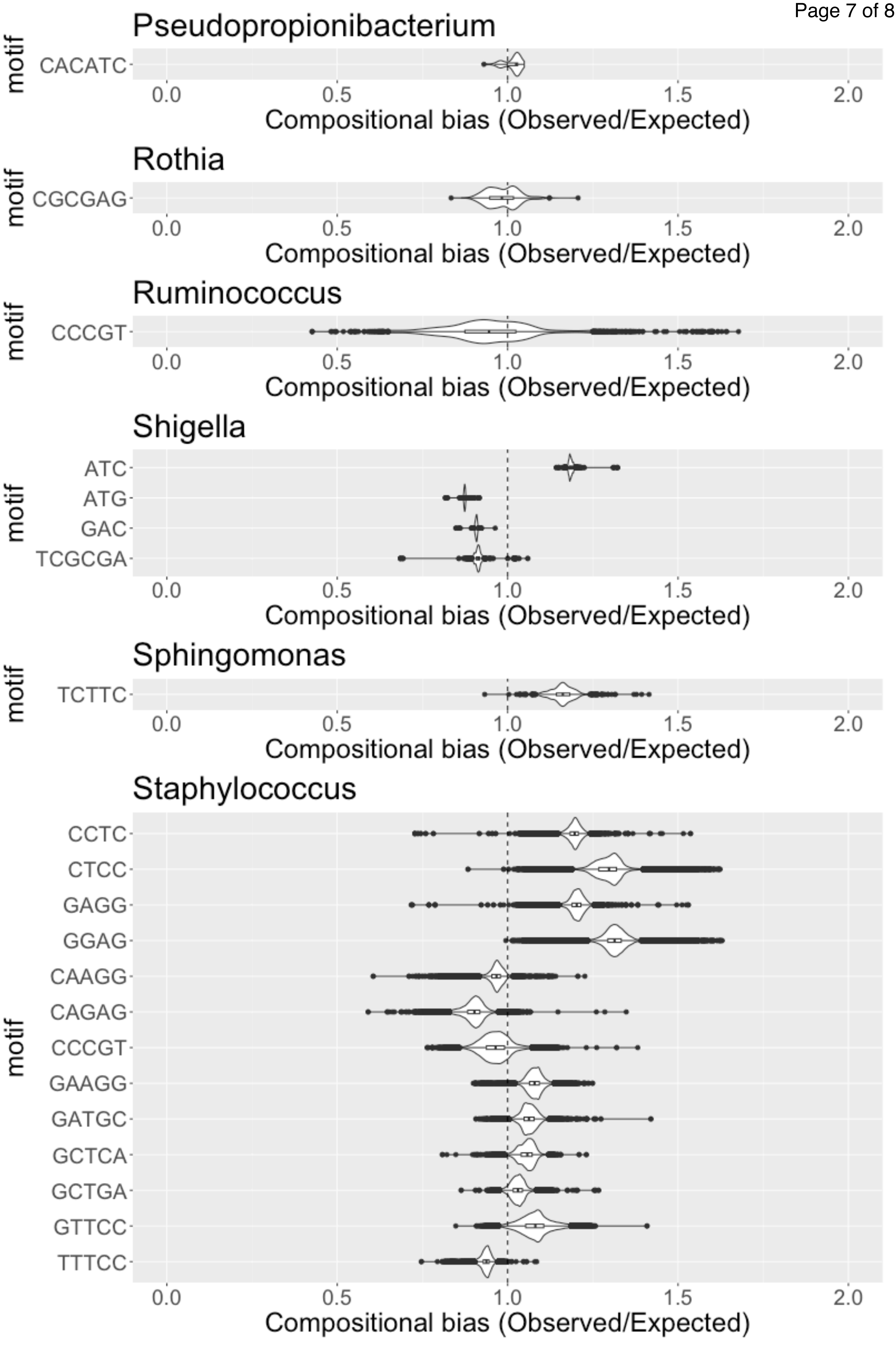

Pseudopropionibacterium
motif
CACATC
0.0
0.5
1.0
1.5
2.0
Compositional bias (Observed/Expected)
Rothia
motif
CGCGAG
Compositional bias (Observed/Expected)
Ruminococcus
motif
CCCGT
Compositional bias (Observed/Expected)
Shigella
motif
ATC
ATG
GAC
TCGCGA
Compositional bias (Observed/Expected)
Sphingomonas
motif
TCTTC
Compositional bias (Observed/Expected)
Staphylococcus
motif
CCTC
CTCC
GAGG
GGAG
CAAGG
CAGAG
CCCGT
GAAGG
GATGC
GCTCA
GCTGA
GTTCC
TTTCC
Compositional bias (Observed/Expected)

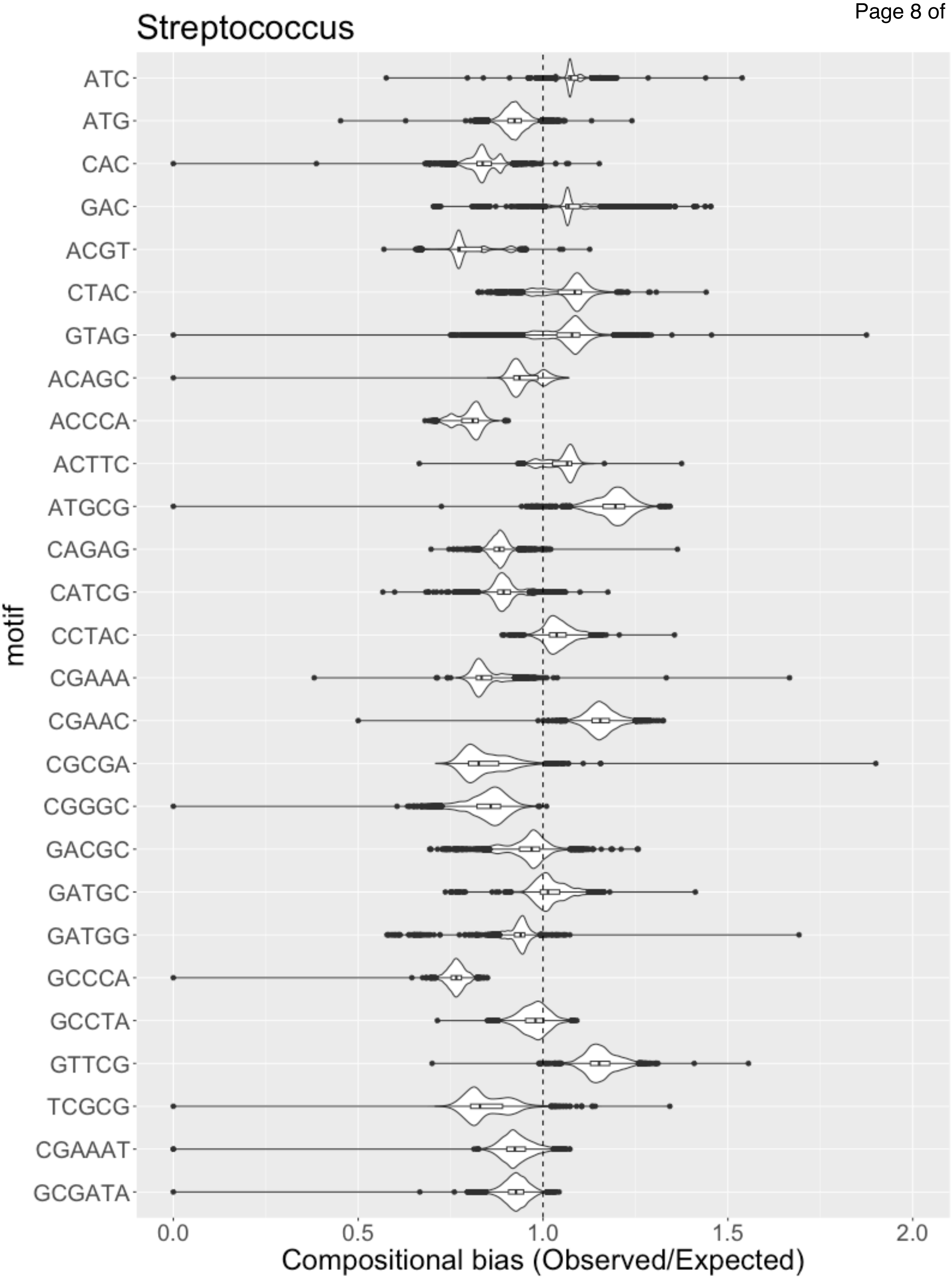
Streptococcus
motif
ATC
ATG
CAC
GAC
ACGT
CTAC
GTAG
ACAGC
ACCCA
ACTTC
ATGCG
CAGAG
CATCG
CCTAC
CGAAA
CGAAC
CGCGA
CGGGC
GACGC
GATGC
GATGG
GCCCA
GCCTA
GTTCG
TCGCG
CGAAAT
GCGATA
0.0
0.5
1.0
1.5
2.0
Compositional bias (Observed/Expected)

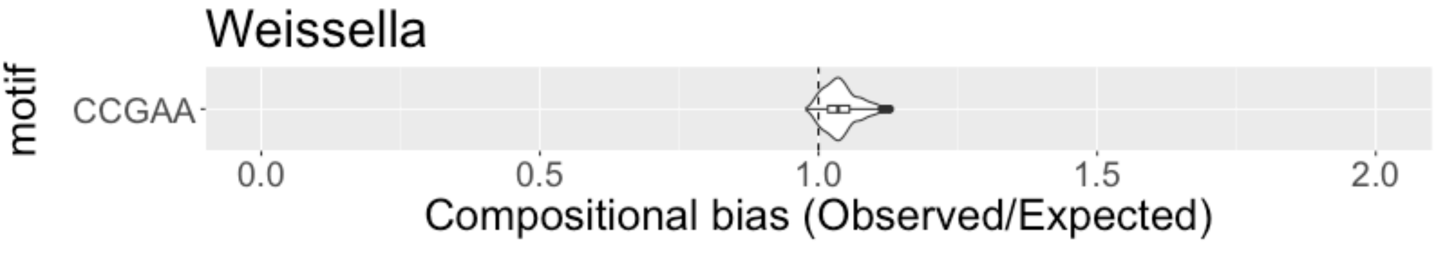
Weissella
motif
CCGAA
0.0
0.5
1.0
1.5
2.0
Compositional bias (Observed/Expected)

Fig. S13. Compositional bias of relevant motifs in bacterial genomes.

Violine plot of compositional bias of the motifs in the genomes of the genera listed in Table S8. Center line represents the median; the edges of the box denote the upper and lower quartiles; individual points show outliers.
