# Supplementary material for: Helicobacter pylori base-excision restriction enzyme in stomach carcinogenesis
Source: PNAS Nexus. 2025 Aug 5;4(8):pgaf244. doi: 10.1093/pnasnexus/pgaf244 (PMC12366791; doi:10.1093/pnasnexus/pgaf244)
Supplement: pgaf244_Supplementary_Data [file pgaf244_supplementary_data.zip › PNASNEXUS-PNASNEXUS-2024-00952RR-s15.docx]

| **Table S1. Association between *Hp*PabI and gastric cancer in *cagA*^+^ *Hp*GP only *H. pylori* clusters.** | | | | | | | | |
| --- | --- | --- | --- | --- | --- | --- | --- | --- |
| Cluster | *Hp*PabI^+^, cancer | *Hp*PabI^+^, non-cancer | *Hp*PabI^-^, cancer | *Hp*PabI^-^, non-cancer | Odds ratio (95% CI) | | *p* value | *Hp*PabI^+^  fraction |
| All | 136 | 320 | 55 | 136 | 1.05 (0.72 to 1.52) | 0.850 | | 0.67 |
| **EU-1_3** | 35 | 68 | 8 | 41 | 2.64 (1.12 to 6.24) | 0.033 | | 0.68 |
| EU-2_3 | 6 | 29 | 0 | 36 | (7.45) (0.85 to 65.41) | 0.052 | | 0.49 |
| EU-3_3 | 19 | 61 | 9 | 38 | 1.32 (0.54 to 3.20) | 0.659 | | 0.63 |
| EU-1_5 | 35 | 61 | 8 | 35 | 2.51 ( 1.05 to 6.01) | 0.047 | | 0.69 |
| EU-2_5 | 5 | 21 | 0 | 18 | (4.29) (0.46 to 40.15) | 0.222 | | 0.59 |
| **EU-3_5** | 19 | 61 | 9 | 38 | 1.32 (0.54 to 3.20) | 0.659 | | 0.63 |
| EU-4_5 | 0 | 7 | 0 | 6 | (0.86) (0.04 to 16.85) | 1.000 | | 0.54 |
| EU-5_5 | 1 | 8 | 0 | 18 | (2.25) (0.12 to 40.66) | 1.000 | | 0.33 |
| EU-1_6 | 12 | 35 | 5 | 12 | 0.82 (0.24 to 2.82) | 0.757 | | 0.73 |
| EU-2_6 | 0 | 0 | 0 | 0 | (1.00) (0.02 to 50.40) | 1.000 | | 0 |
| **EU-3_6** | 12 | 14 | 0 | 11 | (9.43) (1.06 to 84.04) | 0.030 | | 0.70 |
| EU-4_6 | 19 | 24 | 5 | 12 | 1.90 (0.57 to 6.34) | 0.385 | | 0.72 |
| EU-5_6 | 0 | 2 | 0 | 0 | (0.50) (0.01 to 19.56) | 1.000 | | 1.00 |
| EU-6_6 | 2 | 12 | 0 | 7 | (1.17) (0.09 to 15.32) | 1.000 | | 0.67 |
| LA-1_7 | 1 | 10 | 0 | 3 | (0.30) (0.01 to 6.38) | 0.476 | | 0.79 |
| LA-2_7 | 6 | 16 | 5 | 10 | 0.75 (0.18 to 3.12) | 0.728 | | 0.59 |
| LA-3_7 | 1 | 16 | 0 | 4 | (0.25) (0.01 to 4.92) | 0.411 | | 0.81 |
| LA-4_7 | 0 | 0 | 0 | 0 | (1.00) (0.02 to 50.40) | 1.000 | | 0 |
| LA-5_7 | 4 | 29 | 7 | 9 | 0.18 (0.04 to 0.75) | 0.025 | | 0.67 |
| **LA-6_7** | 9 | 10 | 1 | 11 | 9.90 (1.06 to 92.66) | 0.046 | | 0.61 |
| LA-7_7 | 0 | 3 | 0 | 0 | (0.33) (0.01 to 11.94) | 1.000 | | 1.00 |
