# Supplementary material for: Helicobacter pylori base-excision restriction enzyme in stomach carcinogenesis
Source: PNAS Nexus. 2025 Aug 5;4(8):pgaf244. doi: 10.1093/pnasnexus/pgaf244 (PMC12366791; doi:10.1093/pnasnexus/pgaf244)
Supplement: pgaf244_Supplementary_Data [file pgaf244_supplementary_data.zip › PNASNEXUS-PNASNEXUS-2024-00952RR-s16.docx]

| **Table S2. Genes with a GTAC mutation in gastric cancer genomes.** | | | | | | |  |
| --- | --- | --- | --- | --- | --- | --- | --- |
| Gene | chr | position | ref | alt | type | MutationTaster | Tier in  COSMIC |
| AKT3 | chr1 | 243512422 | A | G | Missense_Mutation | disease causing | 2 |
| ARHGEF10L | chr1 | 17695097 | T | C | Intron | . | 2 |
| ASXL2 | chr2 | 25768755 | T | C | Silent | . | 2 |
| BCLAF1 | chr6 | 136273111 | A | G | Silent | . | 2 |
| CRNKL1 | chr20 | 20039716 | T | C | Missense_Mutation | disease causing | 2 |
| CSF1R | chr5 | 150054050 | A | G | 3'UTR | . | 2 |
| ELF3 | chr1 | 202015211 | A | G | Missense_Mutation | disease causing | 2 |
| FAM135B | chr8 | 138242965 | A | G | Missense_Mutation | disease causing | 2 |
| FAM135B | chr8 | 138265757 | T | C | Silent | . | 2 |
| LARP4B | chr10 | 864269 | A | G | Missense_Mutation | polymorphism | 2 |
| LSM14A | chr19 | 34221658 | A | G | Missense_Mutation | polymorphism | 2 |
| MALAT1 | chr11 | 65501635 | A | G | 3'Flank | . | 2 |
| MALAT1 | chr11 | 65501720 | A | G | 3'Flank | . | 2 |
| MUC16 | chr19 | 8936755 | T | A | Silent | . | 2 |
| MUC16 | chr19 | 8956183 | T | C | Missense_Mutation | polymorphism | 2 |
| MUC16 | chr19 | 8960923 | T | C | Missense_Mutation | polymorphism | 2 |
| N4BP2 | chr4 | 40106971 | A | G | Missense_Mutation | disease causing | 2 |
| OMD | chr9 | 92417505 | T | C | Silent | . | 2 |
| PTPN6 | chr12 | 6960171 | T | C | Missense_Mutation | disease causing | 2 |
| SIRPA | chr20 | 1934757 | A | G | Splice_Region | . | 2 |
| SMC1A | chrX | 53413104 | A | T | Missense_Mutation | disease causing | 2 |
| THRAP3 | chr1 | 36301668 | A | G | Missense_Mutation | disease causing | 2 |
| TNC | chr9 | 115046515 | T | C | Missense_Mutation | disease causing | 2 |
| ZCCHC8 | chr12 | 122474265 | T | C | Silent | . | 2 |
| ABL2 | chr1 | 179120201 | A | G | Missense_Mutation | disease causing | 1 |
| AKT1 | chr14 | 104772375 | T | C | Missense_Mutation | disease causing | 1 |
| ATM | chr11 | 108227636 | A | G | Silent | . | 1 |
| ATM | chr11 | 108284386 | T | C | Silent | . | 1 |
| BARD1 | chr2 | 214780751 | T | C | Missense_Mutation | polymorphism | 1 |
| BCL11A | chr2 | 60460945 | T | C | Missense_Mutation | disease causing | 1 |
| BCL3 | chr19 | 44751245 | A | G | Missense_Mutation | polymorphism | 1 |
| BCL9L | chr11 | 118900196 | T | C | Missense_Mutation | polymorphism | 1 |
| BCOR | chrX | 40062744 | A | G | Splice_Site | disease causing | 1 |
| BUB1B | chr15 | 40217616 | A | G | Silent | . | 1 |
| CAMTA1 | chr1 | 7751200 | A | G | Missense_Mutation | disease causing | 1 |
| CCDC6 | chr10 | 59792685 | T | C | 3'UTR | . | 1 |
| CRLF2 | chrX | 1202430 | T | C | Missense_Mutation | polymorphism | 1 |
| CUX1 | chr7 | 102193892 | T | C | Splice_Site | disease causing | 1 |
| CYLD | chr16 | 50796381 | A | G | Missense_Mutation | disease causing | 1 |
| DDIT3 | chr12 | 57517298 | T | C | Missense_Mutation | polymorphism | 1 |
| DNMT3A | chr2 | 25246282 | T | C | Missense_Mutation | disease causing | 1 |
| ELL | chr19 | 18444829 | A | G | Missense_Mutation | disease causing | 1 |
| EP300 | chr22 | 41117712 | A | G | Missense_Mutation | disease causing | 1 |
| FAT1 | chr4 | 186628152 | A | G | Splice_Site | disease causing | 1 |
| FGFR2 | chr10 | 121593826 | T | C | 5'UTR | . | 1 |
| FGFR4 | chr5 | 177093702 | A | G | Silent | . | 1 |
| FLT3 | chr13 | 28033884 | T | C | Splice_Region | . | 1 |
| HIP1 | chr7 | 75553451 | A | G | Splice_Site | disease causing | 1 |
| HOXD13 | chr2 | 176093617 | T | C | Missense_Mutation | disease causing | 1 |
| HRAS | chr11 | 533845 | A | G | Missense_Mutation | disease causing | 1 |
| KAT6A | chr8 | 41932288 | A | G | Missense_Mutation | disease causing | 1 |
| KAT6B | chr10 | 74975783 | T | C | Silent | disease causing | 1 |
| KDSR | chr18 | 63355212 | T | A | Missense_Mutation | polymorphism | 1 |
| KIF5B | chr10 | 32040453 | T | C | Silent | . | 1 |
| LRP1B | chr2 | 140950245 | A | G | Silent | . | 1 |
| LZTR1 | chr22 | 20994647 | T | C | Missense_Mutation | disease causing | 1 |
| MDM4 | chr1 | 204530706 | A | G | Missense_Mutation | disease causing | 1 |
| NCOR2 | chr12 | 124426682 | T | C | Missense_Mutation | disease causing | 1 |
| NUMA1 | chr11 | 72018394 | A | G | Splice_Site | disease causing | 1 |
| NUP214 | chr9 | 131144284 | T | G | Missense_Mutation | disease causing | 1 |
| NUP98 | chr11 | 3773685 | T | C | Missense_Mutation | disease causing | 1 |
| PALB2 | chr16 | 23629957 | T | C | Missense_Mutation | polymorphism | 1 |
| PIK3R1 | chr5 | 68297489 | A | G | Missense_Mutation | disease causing | 1 |
| POLE | chr12 | 132632792 | T | C | Missense_Mutation | disease causing | 1 |
| POLQ | chr3 | 121510220 | A | G | Silent | . | 1 |
| POT1 | chr7 | 124827292 | T | C | Silent | . | 1 |
| PTCH1 | chr9 | 95468978 | A | G | Missense_Mutation | disease causing | 1 |
| PTCH1 | chr9 | 95468998 | T | C | Missense_Mutation | disease causing | 1 |
| PTK6 | chr20 | 63532607 | A | G | Missense_Mutation | polymorphism | 1 |
| PTPRB | chr12 | 70569740 | T | C | Missense_Mutation | disease causing | 1 |
| PTPRT | chr20 | 42352176 | T | C | Missense_Mutation | disease causing | 1 |
| PTPRT | chr20 | 42472556 | A | C | Missense_Mutation | disease causing | 1 |
| RANBP2 | chr2 | 108766778 | T | C | Missense_Mutation | disease causing | 1 |
| RNF213 | chr17 | 80348195 | A | G | Missense_Mutation | disease causing | 1 |
| RNF213 | chr17 | 80371959 | T | C | Missense_Mutation | polymorphism | 1 |
| SUFU | chr10 | 102509207 | A | G | Missense_Mutation | disease causing | 1 |
| TOP1 | chr20 | 41098339 | T | C | Splice_Site | disease causing | 1 |
| TP53 | chr17 | 7673533 | A | G | Splice_Site | disease causing | 1 |
| TP53 | chr17 | 7675236 | A | C | Missense_Mutation | disease causing | 1 |
| ZNF521 | chr18 | 25226941 | T | C | Missense_Mutation | disease causing | 1 |
| A2ML1 | chr12 | 8838402 | T | C | Missense_Mutation | polymorphism | . |
| A2ML1 | chr12 | 8852241 | A | G | Missense_Mutation | polymorphism | . |
| AARS2 | chr6 | 44307328 | A | G | Missense_Mutation | disease causing | . |
| AASDH | chr4 | 56354168 | T | C | Silent | . | . |
| ABCA10 | chr17 | 69153828 | T | C | Splice_Region | . | . |
| ABCB1 | chr7 | 87550466 | A | G | Splice_Site | disease causing | . |
| ABCC10 | chr6 | 43438752 | A | G | Missense_Mutation | disease causing | . |
| ABCC9 | chr12 | 21908121 | A | G | Missense_Mutation | disease causing | . |
| ABHD14A | chr3 | 51980480 | T | C | Missense_Mutation | disease causing | . |
| ABI3BP | chr3 | 100926347 | A | C | Missense_Mutation | disease causing | . |
| ACAD9 | chr3 | 128908209 | T | C | Missense_Mutation | disease causing | . |
| ACLY | chr17 | 41913814 | A | G | Silent | . | . |
| ACOT7 | chr1 | 6339431 | A | G | Splice_Site | disease causing | . |
| ACSS3 | chr12 | 81139186 | A | G | Missense_Mutation | disease causing | . |
| ACTB | chr7 | 5528107 | T | C | Missense_Mutation | disease causing | . |
| ACTRT1 | chrX | 128051028 | T | C | 3'UTR | . | . |
| ACY3 | chr11 | 67642848 | T | C | Missense_Mutation | disease causing | . |
| ADAM18 | chr8 | 39668059 | A | T | Missense_Mutation | disease causing | . |
| ADAM2 | chr8 | 39821165 | A | G | Missense_Mutation | polymorphism | . |
| ADAM32 | chr8 | 39186910 | A | G | Missense_Mutation | disease causing | . |
| ADAM7 | chr8 | 24465759 | A | G | Missense_Mutation | disease causing | . |
| ADAM9 | chr8 | 39055591 | T | C | Silent | . | . |
| ADAMTS10 | chr19 | 8603781 | T | C | Missense_Mutation | disease causing | . |
| ADAMTS12 | chr5 | 33614336 | T | C | Missense_Mutation | disease causing | . |
| ADAMTS15 | chr11 | 130461539 | T | C | Silent | . | . |
| ADAMTSL1 | chr9 | 18574215 | T | C | Silent | . | . |
| ADARB2 | chr10 | 1556878 | A | G | Intron | . | . |
| ADCY6 | chr12 | 48773647 | A | G | Missense_Mutation | disease causing | . |
| ADCYAP1R1 | chr7 | 31086519 | T | C | Missense_Mutation | disease causing | . |
| ADGRA2 | chr8 | 37841499 | A | G | Missense_Mutation | disease causing | . |
| ADGRD1 | chr12 | 131005999 | A | G | Missense_Mutation | disease causing | . |
| ADGRL3 | chr4 | 61892784 | A | G | Missense_Mutation | polymorphism | . |
| ADH6 | chr4 | 99210267 | T | C | Missense_Mutation | polymorphism | . |
| ADI1 | chr2 | 3500924 | A | G | Missense_Mutation | disease causing | . |
| ADIPOR1 | chr1 | 202941449 | T | C | 3'UTR | . | . |
| AEBP1 | chr7 | 44110786 | T | C | Missense_Mutation | disease causing | . |
| AHNAK | chr11 | 62516947 | T | C | Missense_Mutation | disease causing | . |
| AIG1 | chr6 | 143284200 | A | G | Missense_Mutation | polymorphism | . |
| AKAP10 | chr17 | 19936428 | T | C | Missense_Mutation | disease causing | . |
| AKAP12 | chr6 | 151353076 | T | C | Missense_Mutation | polymorphism | . |
| AKAP13 | chr15 | 85719133 | A | G | Missense_Mutation | disease causing | . |
| AKAP17A | chrX | 1594124 | A | G | Missense_Mutation | polymorphism | . |
| AKR7A2 | chr1 | 19307080 | T | C | Missense_Mutation | disease causing | . |
| AKR7A3 | chr1 | 19288565 | A | G | Missense_Mutation | disease causing | . |
| ALDH18A1 | chr10 | 95627470 | A | G | Silent | . | . |
| ALDH4A1 | chr1 | 18875476 | A | G | Missense_Mutation | disease causing | . |
| ALMS1 | chr2 | 73599493 | A | G | Silent | . | . |
| ALOX15 | chr17 | 4631640 | T | C | Missense_Mutation | disease causing | . |
| ALPK2 | chr18 | 58536218 | T | C | Silent | . | . |
| ALPK2 | chr18 | 58579096 | A | G | Silent | . | . |
| AMBP | chr9 | 114061496 | A | G | Missense_Mutation | disease causing | . |
| AMD1 | chr6 | 110892301 | A | G | Missense_Mutation | disease causing | . |
| AMPD3 | chr11 | 10495010 | T | G | Missense_Mutation | disease causing | . |
| AMY1A | chr1 | 103691388 | T | C | Missense_Mutation | disease causing | . |
| ANK2 | chr4 | 113237163 | A | G | Silent | . | . |
| ANKLE2 | chr12 | 132754720 | A | G | Missense_Mutation | disease causing | . |
| ANKRD12 | chr18 | 9258530 | A | G | Missense_Mutation | polymorphism | . |
| ANKRD17 | chr4 | 73090952 | T | A | Missense_Mutation | polymorphism | . |
| ANKRD18A | chr9 | 38572175 | T | C | Intron | . | . |
| ANKRD23 | chr2 | 96840824 | T | C | Missense_Mutation | disease causing | . |
| ANKRD30A | chr10 | 37201272 | T | C | Missense_Mutation | polymorphism | . |
| ANKRD35 | chr1 | 145876238 | A | G | Silent | . | . |
| ANKRD54 | chr22 | 37832649 | A | G | Silent | . | . |
| ANO1 | chr11 | 70182547 | T | C | Missense_Mutation | disease causing | . |
| ANO2 | chr12 | 5578364 | A | G | Splice_Site | disease causing | . |
| ANO9 | chr11 | 429589 | T | C | Missense_Mutation | disease causing | . |
| AP4E1 | chr15 | 50929074 | T | C | Missense_Mutation | disease causing | . |
| APOPT1 | chr14 | 103590188 | T | C | Missense_Mutation | disease causing | . |
| AQR | chr15 | 34884683 | T | C | Missense_Mutation | polymorphism | . |
| ARFGAP2 | chr11 | 47176586 | A | T | Missense_Mutation | disease causing | . |
| ARFGEF2 | chr20 | 49013686 | A | G | Silent | . | . |
| ARHGAP24 | chr4 | 85977584 | T | C | Missense_Mutation | disease causing | . |
| ARHGAP27 | chr17 | 45396665 | T | C | Splice_Region | . | . |
| ARHGAP27 | chr17 | 45398022 | T | C | Missense_Mutation | disease causing | . |
| ARHGDIA | chr17 | 81869205 | T | C | Missense_Mutation | disease causing | . |
| ARHGEF19 | chr1 | 16205536 | A | G | Splice_Site | disease causing | . |
| ARMC3 | chr10 | 23030656 | A | G | Silent | . | . |
| ASAP3 | chr1 | 23431714 | T | C | Missense_Mutation | polymorphism | . |
| ASB4 | chr7 | 95537724 | T | C | Missense_Mutation | disease causing | . |
| ASB6 | chr9 | 129637834 | A | G | Missense_Mutation | disease causing | . |
| ASH1L | chr1 | 155481547 | A | G | Silent | . | . |
| ASIC3 | chr7 | 151050610 | T | C | Splice_Site | disease causing | . |
| ASPM | chr1 | 197102763 | T | C | Missense_Mutation | disease causing | . |
| ASPM | chr1 | 197122493 | T | C | Missense_Mutation | disease causing | . |
| ASPM | chr1 | 197142739 | T | C | Missense_Mutation | polymorphism | . |
| ASTN2 | chr9 | 116618439 | A | T | Missense_Mutation | disease causing | . |
| ATAD2 | chr8 | 123359334 | T | C | Splice_Region | . | . |
| ATAD5 | chr17 | 30834350 | T | C | Missense_Mutation | polymorphism | . |
| ATF7IP | chr12 | 14497944 | T | C | Silent | . | . |
| ATG2A | chr11 | 64910814 | T | C | Missense_Mutation | disease causing | . |
| ATG2A | chr11 | 64910814 | T | C | Missense_Mutation | disease causing | . |
| ATL2 | chr2 | 38298227 | T | C | Missense_Mutation | disease causing | . |
| ATP11A | chr13 | 112826763 | T | C | Missense_Mutation | disease causing | . |
| ATP1B4 | chrX | 120362101 | A | G | 5'Flank | . | . |
| ATP6AP2 | chrX | 40591351 | T | C | Missense_Mutation | disease causing | . |
| ATP6V0A1 | chr17 | 42500925 | T | C | Splice_Site | disease causing | . |
| ATRNL1 | chr10 | 115121807 | A | G | Silent | . | . |
| AURKA | chr20 | 56386325 | A | T | Missense_Mutation | polymorphism | . |
| AVPR1A | chr12 | 63147424 | T | C | Missense_Mutation | polymorphism | . |
| AVPR1A | chr12 | 63150280 | T | C | Missense_Mutation | disease causing | . |
| AZIN2 | chr1 | 33119987 | T | C | Intron | . | . |
| B4GALNT1 | chr12 | 57626835 | T | C | Missense_Mutation | disease causing | . |
| B4GALT6 | chr18 | 31684338 | T | C | Missense_Mutation | disease causing | . |
| B4GALT7 | chr5 | 177608940 | T | C | Missense_Mutation | disease causing | . |
| BAHCC1 | chr17 | 81459526 | T | C | Missense_Mutation | polymorphism | . |
| BAZ1B | chr7 | 73478083 | T | C | Missense_Mutation | polymorphism | . |
| BBOX1 | chr11 | 27119822 | A | G | Silent | . | . |
| BBS2 | chr16 | 56498610 | A | G | Intron | . | . |
| BBS9 | chr7 | 33264291 | T | C | Missense_Mutation | disease causing | . |
| BBS9 | chr7 | 33264298 | T | C | Missense_Mutation | disease causing | . |
| BCAS1 | chr20 | 54028796 | T | C | Missense_Mutation | polymorphism | . |
| BCCIP | chr10 | 125831594 | T | C | Missense_Mutation | disease causing | . |
| BCCIP | chr10 | 125836184 | A | G | Silent | . | . |
| BCL2L2-PABPN1 | chr14 | 23323068 | T | C | Splice_Site | disease causing | . |
| BEND2 | chrX | 18203637 | T | C | Silent | . | . |
| BEST2 | chr19 | 12755438 | A | G | Silent | . | . |
| BLOC1S1 | chr12 | 55721957 | A | G | 3'UTR | . | . |
| BLVRB | chr19 | 40447930 | A | G | Missense_Mutation | disease causing | . |
| BMP4 | chr14 | 53950081 | A | G | Missense_Mutation | disease causing | . |
| BMP7 | chr20 | 57202593 | A | G | Silent | . | . |
| BMPER | chr7 | 34078940 | T | C | Missense_Mutation | disease causing | . |
| BMPR1B | chr4 | 95131399 | T | C | Silent | . | . |
| BMS1P4 | chr10 | 73722575 | T | C | Splice_Region | . | . |
| BRD8 | chr5 | 138150778 | A | G | Silent | . | . |
| BRWD3 | chrX | 80688087 | A | G | Silent | . | . |
| BTAF1 | chr10 | 91996416 | T | C | Silent | . | . |
| C11orf42 | chr11 | 6209888 | A | G | Silent | . | . |
| C11orf63 | chr11 | 122934470 | A | G | Silent | . | . |
| C12orf60 | chr12 | 14823516 | T | C | Missense_Mutation | polymorphism | . |
| C18orf8 | chr18 | 23527873 | A | G | Missense_Mutation | disease causing | . |
| C1GALT1 | chr7 | 7238675 | T | C | Missense_Mutation | disease causing | . |
| C2 | chr6 | 31950600 | A | G | Intron | . | . |
| C21orf59 | chr21 | 32603065 | A | G | Intron | . | . |
| C21orf59 | chr21 | 32604123 | A | G | Splice_Site | disease causing | . |
| C3 | chr19 | 6710724 | T | C | Missense_Mutation | disease causing | . |
| C3orf67 | chr3 | 58869339 | A | G | Missense_Mutation | polymorphism | . |
| C9orf84 | chr9 | 111713171 | A | G | Missense_Mutation | disease causing | . |
| CABYR | chr18 | 24156887 | T | C | Silent | . | . |
| CACNA1E | chr1 | 181651374 | T | C | Missense_Mutation | disease causing | . |
| CACNG1 | chr17 | 67044832 | A | G | Missense_Mutation | disease causing | . |
| CACNG5 | chr17 | 66885005 | A | G | Missense_Mutation | disease causing | . |
| CAND1 | chr12 | 67297820 | A | G | Missense_Mutation | disease causing | . |
| CAPN10 | chr2 | 240595159 | A | G | Missense_Mutation | polymorphism | . |
| CAPN15 | chr16 | 551649 | A | G | Missense_Mutation | disease causing | . |
| CARNMT1 | chr9 | 74999843 | T | C | Silent | . | . |
| CASD1 | chr7 | 94539053 | T | C | Silent | . | . |
| CASP1 | chr11 | 105026181 | T | C | Intron | . | . |
| CBARP | chr19 | 1234740 | T | C | Missense_Mutation | disease causing | . |
| CC2D1B | chr1 | 52356183 | T | C | Splice_Region | . | . |
| CCDC18 | chr1 | 93239828 | A | G | Silent | . | . |
| CCDC88A | chr2 | 55346293 | T | C | Missense_Mutation | disease causing | . |
| CCT6A | chr7 | 56055758 | T | C | Silent | . | . |
| CCZ1B | chr7 | 6824651 | T | C | Silent | . | . |
| CD1C | chr1 | 158292097 | A | G | Silent | . | . |
| CD300LF | chr17 | 74695858 | A | G | Missense_Mutation | polymorphism | . |
| CD6 | chr11 | 61009629 | T | A | Missense_Mutation | disease causing | . |
| CDCA7 | chr2 | 173364945 | T | C | Missense_Mutation | disease causing | . |
| CDH26 | chr20 | 59996468 | T | C | Intron | . | . |
| CDK16 | chrX | 47224990 | T | C | Splice_Region | . | . |
| CDK17 | chr12 | 96298980 | T | C | Missense_Mutation | disease causing | . |
| CDON | chr11 | 125984046 | T | C | Missense_Mutation | disease causing | . |
| CEACAM16 | chr19 | 44699267 | A | G | 5'Flank | . | . |
| CEACAM16 | chr19 | 44705800 | A | G | Missense_Mutation | disease causing | . |
| CEACAM20 | chr19 | 44522656 | A | G | Silent | . | . |
| CECR1 | chr22 | 17181575 | A | G | Missense_Mutation | disease causing | . |
| CECR2 | chr22 | 17541850 | A | G | Silent | . | . |
| CECR5 | chr22 | 17138310 | T | C | Missense_Mutation | disease causing | . |
| CEL | chr9 | 133070556 | A | G | Missense_Mutation | disease causing | . |
| CELF3 | chr1 | 151715893 | T | C | Missense_Mutation | disease causing | . |
| CENPE | chr4 | 103133752 | T | C | Silent | . | . |
| CEP120 | chr5 | 123388521 | T | A | Silent | . | . |
| CEP19 | chr3 | 196708526 | A | G | Splice_Site | disease causing | . |
| CEP192 | chr18 | 13056562 | T | A | Silent | . | . |
| CEP72 | chr5 | 635407 | T | C | Missense_Mutation | polymorphism | . |
| CERKL | chr2 | 181603973 | A | G | Silent | . | . |
| CFAP52 | chr17 | 9585935 | A | G | Missense_Mutation | disease causing | . |
| CFAP53 | chr18 | 50242928 | A | G | Silent | . | . |
| CFAP61 | chr20 | 20196580 | A | G | Missense_Mutation | disease causing | . |
| CFH | chr1 | 196742053 | T | A | Splice_Site | disease causing | . |
| CGN | chr1 | 151518667 | A | G | Missense_Mutation | polymorphism | . |
| CH25H | chr10 | 89206536 | A | G | Missense_Mutation | disease causing | . |
| CHAT | chr10 | 49622124 | A | G | Silent | . | . |
| CHD1L | chr1 | 147295566 | T | C | 3'UTR | . | . |
| CHD6 | chr20 | 41451930 | T | C | Missense_Mutation | disease causing | . |
| CHML | chr1 | 241635550 | T | A | Missense_Mutation | polymorphism | . |
| CHMP4B | chr20 | 33848646 | T | C | Splice_Site | disease causing | . |
| CHORDC1 | chr11 | 90205495 | T | C | Missense_Mutation | disease causing | . |
| CHRNA9 | chr4 | 40354369 | A | G | Missense_Mutation | disease causing | . |
| CHST8 | chr19 | 33773058 | T | C | Missense_Mutation | disease causing | . |
| CHST9 | chr18 | 26917322 | A | G | Missense_Mutation | polymorphism | . |
| CKAP2L | chr2 | 112746570 | T | C | Silent | . | . |
| CLCA2 | chr1 | 86455379 | T | C | Missense_Mutation | polymorphism | . |
| CLCA4 | chr1 | 86567523 | A | G | Missense_Mutation | polymorphism | . |
| CLCN3 | chr4 | 169697327 | T | C | Missense_Mutation | disease causing | . |
| CLCNKA | chr1 | 16029247 | A | G | Missense_Mutation | polymorphism | . |
| CLDN12 | chr7 | 90412656 | T | C | 5'UTR | . | . |
| CLDN16 | chr3 | 190404875 | T | C | Missense_Mutation | polymorphism | . |
| CLDN23 | chr8 | 8702801 | T | C | Missense_Mutation | disease causing | . |
| CLEC12B | chr12 | 10015290 | T | C | Missense_Mutation | polymorphism | . |
| CLK3 | chr15 | 74628679 | A | G | Missense_Mutation | disease causing | . |
| CLMN | chr14 | 95202836 | A | G | Splice_Site | disease causing | . |
| CMYA5 | chr5 | 79733724 | T | C | Silent | . | . |
| CNGA1 | chr4 | 47937064 | A | G | Missense_Mutation | disease causing | . |
| CNGA2 | chrX | 151744338 | A | G | Missense_Mutation | disease causing | . |
| CNOT1 | chr16 | 58559802 | A | G | Intron | . | . |
| CNTN1 | chr12 | 41025217 | A | G | Missense_Mutation | disease causing | . |
| CNTN4 | chr3 | 2745632 | A | G | Missense_Mutation | disease causing | . |
| CNTN5 | chr11 | 100061308 | A | G | Silent | . | . |
| CNTNAP1 | chr17 | 42693400 | T | C | Silent | . | . |
| COBLL1 | chr2 | 164743685 | A | G | Splice_Site | disease causing | . |
| COG5 | chr7 | 107200355 | A | G | Intron | . | . |
| COL14A1 | chr8 | 120278484 | T | C | Silent | . | . |
| COL4A2 | chr13 | 110466064 | T | C | Splice_Site | disease causing | . |
| COL5A2 | chr2 | 189068250 | A | G | Silent | . | . |
| COPG1 | chr3 | 129268937 | A | G | Missense_Mutation | polymorphism | . |
| CORO7-PAM16 | chr16 | 4365044 | T | C | Missense_Mutation | disease causing | . |
| CPM | chr12 | 68871867 | A | G | Silent | . | . |
| CPPED1 | chr16 | 12665069 | A | G | Silent | . | . |
| CPSF1 | chr8 | 144394278 | T | C | Missense_Mutation | disease causing | . |
| CPSF1 | chr8 | 144401056 | A | G | Missense_Mutation | disease causing | . |
| CPSF2 | chr14 | 92143114 | A | G | Silent | . | . |
| CPSF3L | chr1 | 1312924 | T | C | Missense_Mutation | disease causing | . |
| CR2 | chr1 | 207466627 | A | G | Missense_Mutation | polymorphism | . |
| CR2 | chr1 | 207475066 | T | C | Missense_Mutation | polymorphism | . |
| CRAT | chr9 | 129102413 | A | G | Missense_Mutation | disease causing | . |
| CRYZ | chr1 | 74706257 | A | G | 3'UTR | . | . |
| CRYZ | chr1 | 74706977 | A | G | Silent | . | . |
| CSK | chr15 | 74798632 | T | C | Silent | . | . |
| CSTF3 | chr11 | 33085204 | T | C | Silent | . | . |
| CTCFL | chr20 | 57523779 | A | G | Missense_Mutation | polymorphism | . |
| CTSB | chr8 | 11852641 | T | C | Missense_Mutation | disease causing | . |
| CTSS | chr1 | 150751837 | A | G | Missense_Mutation | disease causing | . |
| CTSW | chr11 | 65883081 | A | G | Missense_Mutation | polymorphism | . |
| CUBN | chr10 | 16899151 | T | C | Missense_Mutation | polymorphism | . |
| CUL1 | chr7 | 148730217 | A | G | Missense_Mutation | disease causing | . |
| CUL1 | chr7 | 148759356 | T | C | Splice_Site | disease causing | . |
| CUL1 | chr7 | 148792759 | T | C | Missense_Mutation | disease causing | . |
| CUL4A | chr13 | 113254800 | T | C | Splice_Site | disease causing | . |
| CUL7 | chr6 | 43050295 | T | A | Missense_Mutation | polymorphism | . |
| CUX2 | chr12 | 111320681 | A | G | Missense_Mutation | disease causing | . |
| CYFIP1 | chr15 | 22916676 | T | C | Intron | . | . |
| CYP2B7P | chr19 | 40944505 | A | G | 5'Flank | . | . |
| CYP2R1 | chr11 | 14891988 | T | C | Missense_Mutation | disease causing | . |
| CYP4F8 | chr19 | 15618147 | A | G | Splice_Region | . | . |
| DACH2 | chrX | 86714705 | T | C | Silent | . | . |
| DBNL | chr7 | 44060138 | T | C | Missense_Mutation | disease causing | . |
| DCAF4L2 | chr8 | 87873677 | A | G | Missense_Mutation | disease causing | . |
| DCUN1D5 | chr11 | 103066266 | T | C | Splice_Region | . | . |
| DDHD1 | chr14 | 53091918 | T | C | Missense_Mutation | disease causing | . |
| DDX39A | chr19 | 14409816 | A | G | Missense_Mutation | disease causing | . |
| DEAF1 | chr11 | 687961 | A | G | Missense_Mutation | disease causing | . |
| DENND6B | chr22 | 50317343 | A | G | Missense_Mutation | disease causing | . |
| DGCR2 | chr22 | 19038726 | T | C | 3'UTR | . | . |
| DGKH | chr13 | 42221323 | T | C | Missense_Mutation | disease causing | . |
| DHX30 | chr3 | 47849052 | T | C | Missense_Mutation | disease causing | . |
| DHX36 | chr3 | 154316037 | A | G | Splice_Site | disease causing | . |
| DHX58 | chr17 | 42104777 | A | G | Missense_Mutation | polymorphism | . |
| DIAPH1 | chr5 | 141574035 | A | G | Silent | . | . |
| DIP2B | chr12 | 50640804 | T | C | Missense_Mutation | disease causing | . |
| DIS3 | chr13 | 72776015 | A | G | Silent | . | . |
| DLGAP4 | chr20 | 36446699 | A | G | Splice_Region | . | . |
| DLK2 | chr6 | 43450876 | A | G | Missense_Mutation | polymorphism | . |
| DMC1 | chr22 | 38566694 | T | C | Missense_Mutation | disease causing | . |
| DMRT2 | chr9 | 1055754 | T | C | Intron | disease causing | . |
| DNAH11 | chr7 | 21559000 | T | C | Splice_Site | disease causing | . |
| DNAH11 | chr7 | 21704435 | T | C | Missense_Mutation | disease causing | . |
| DNAH17 | chr17 | 78475342 | T | C | Missense_Mutation | polymorphism | . |
| DNAH3 | chr16 | 21021986 | A | G | Missense_Mutation | disease causing | . |
| DNAH3 | chr16 | 21106661 | A | G | Missense_Mutation | disease causing | . |
| DNAH5 | chr5 | 13824227 | A | T | Missense_Mutation | disease causing | . |
| DNAH8 | chr6 | 38860595 | A | G | Missense_Mutation | disease causing | . |
| DNAH9 | chr17 | 11747619 | A | T | Missense_Mutation | disease causing | . |
| DNAJC14 | chr12 | 55828188 | A | G | Silent | . | . |
| DNAJC6 | chr1 | 65364787 | T | C | Splice_Site | disease causing | . |
| DNAJC7 | chr17 | 41994867 | T | C | Splice_Region | . | . |
| DNER | chr2 | 229512781 | A | T | Splice_Site | disease causing | . |
| DNER | chr2 | 229512897 | T | A | Missense_Mutation | polymorphism | . |
| DNM1P46 | chr15 | 99791661 | T | C | 5'Flank | . | . |
| DNMBP | chr10 | 99896388 | T | C | Missense_Mutation | disease causing | . |
| DOCK11 | chrX | 118593339 | T | C | Splice_Site | disease causing | . |
| DOCK2 | chr5 | 170027926 | T | C | Missense_Mutation | disease causing | . |
| DOCK2 | chr5 | 170036549 | A | G | Missense_Mutation | disease causing | . |
| DOCK5 | chr8 | 25374693 | A | G | Intron | . | . |
| DOCK6 | chr19 | 11252915 | A | G | Missense_Mutation | polymorphism | . |
| DOPEY1 | chr6 | 83135789 | T | C | Missense_Mutation | disease causing | . |
| DPP4 | chr2 | 162018783 | A | G | Missense_Mutation | disease causing | . |
| DPP9 | chr19 | 4690876 | A | G | Splice_Site | disease causing | . |
| DPPA2 | chr3 | 109304613 | A | G | Missense_Mutation | polymorphism | . |
| DPY19L1P1 | chr7 | 32632587 | A | G | RNA | . | . |
| DSC3 | chr18 | 31008157 | A | G | Missense_Mutation | disease causing | . |
| DSC3 | chr18 | 31022501 | A | G | Splice_Region | . | . |
| DSCAM | chr21 | 40347838 | A | G | Missense_Mutation | disease causing | . |
| DSCAML1 | chr11 | 117504000 | T | C | Missense_Mutation | disease causing | . |
| DTX1 | chr12 | 113095095 | T | C | Silent | . | . |
| DUSP10 | chr1 | 221739198 | A | G | Missense_Mutation | disease causing | . |
| DUSP22 | chr6 | 350871 | T | C | 3'UTR | . | . |
| DUSP27 | chr1 | 167127691 | T | C | Missense_Mutation | disease causing | . |
| DYNC1H1 | chr14 | 102017160 | T | C | Missense_Mutation | disease causing | . |
| DYNC1H1 | chr14 | 102039430 | T | C | Missense_Mutation | disease causing | . |
| ECT2 | chr3 | 172773914 | A | C | Silent | . | . |
| EFTUD2 | chr17 | 44854554 | A | G | Splice_Site | disease causing | . |
| EFTUD2 | chr17 | 44860541 | T | C | Missense_Mutation | disease causing | . |
| EHBP1L1 | chr11 | 65583582 | T | C | Silent | . | . |
| EHHADH | chr3 | 185248516 | T | C | Missense_Mutation | disease causing | . |
| EHMT2 | chr6 | 31883867 | T | C | Missense_Mutation | disease causing | . |
| EIF5 | chr14 | 103340988 | T | C | Missense_Mutation | disease causing | . |
| ELL3 | chr15 | 43773186 | T | C | Missense_Mutation | disease causing | . |
| EMB | chr5 | 50403205 | A | G | Missense_Mutation | disease causing | . |
| EMCN | chr4 | 100421308 | T | A | Missense_Mutation | disease causing | . |
| EML1 | chr14 | 99897183 | A | G | Missense_Mutation | disease causing | . |
| EML3 | chr11 | 62607819 | A | G | Splice_Region | . | . |
| EML5 | chr14 | 88625019 | A | G | Missense_Mutation | disease causing | . |
| ENO3 | chr17 | 4956891 | T | A | Splice_Site | disease causing | . |
| ENTPD8 | chr9 | 137435278 | A | G | Missense_Mutation | disease causing | . |
| EPB42 | chr15 | 43216333 | T | C | Missense_Mutation | polymorphism | . |
| EPG5 | chr18 | 45866937 | T | C | Missense_Mutation | disease causing | . |
| EPHA4 | chr2 | 221563790 | A | G | Missense_Mutation | disease causing | . |
| EPHB2 | chr1 | 22784969 | T | C | Missense_Mutation | disease causing | . |
| EPS8L1 | chr19 | 55085967 | A | G | Silent | . | . |
| ERAP1 | chr5 | 96803493 | T | C | Missense_Mutation | disease causing | . |
| ERBIN | chr5 | 66044213 | T | C | Missense_Mutation | disease causing | . |
| ERGIC3 | chr20 | 35542987 | A | G | Intron | . | . |
| ESPL1 | chr12 | 53286586 | A | G | Missense_Mutation | polymorphism | . |
| ESR2 | chr14 | 64260481 | A | G | Missense_Mutation | disease causing | . |
| ESYT2 | chr7 | 158734133 | T | C | 3'UTR | . | . |
| ETFA | chr15 | 76285731 | A | G | Silent | . | . |
| EVI2A | chr17 | 31318694 | A | G | Missense_Mutation | polymorphism | . |
| EVI5L | chr19 | 7853162 | A | G | Missense_Mutation | disease causing | . |
| EXO1 | chr1 | 241857484 | T | C | Splice_Site | disease causing | . |
| EXOC4 | chr7 | 134064362 | A | G | Missense_Mutation | disease causing | . |
| EXOSC10 | chr1 | 11081080 | A | G | Splice_Site | disease causing | . |
| EXOSC8 | chr13 | 37009558 | T | C | 3'UTR | . | . |
| F12 | chr5 | 177405993 | T | C | Missense_Mutation | polymorphism | . |
| F5 | chr1 | 169529680 | A | G | Missense_Mutation | disease causing | . |
| F5 | chr1 | 169552666 | T | C | Missense_Mutation | disease causing | . |
| FAAH2 | chrX | 57310731 | T | C | Splice_Site | disease causing | . |
| FAM111A | chr11 | 59152743 | T | C | Missense_Mutation | polymorphism | . |
| FAM160A2 | chr11 | 6223211 | A | G | Missense_Mutation | disease causing | . |
| FAM183BP | chr7 | 38685644 | T | C | Missense_Mutation | . | . |
| FAM188A | chr10 | 15779134 | T | C | Missense_Mutation | disease causing | . |
| FAM210A | chr18 | 13681934 | T | C | Silent | . | . |
| FAM214A | chr15 | 52584834 | T | C | Silent | . | . |
| FAM71F1 | chr7 | 128716886 | T | C | Missense_Mutation | disease causing | . |
| FAT2 | chr5 | 151521740 | T | C | Missense_Mutation | disease causing | . |
| FAT2 | chr5 | 151568721 | A | G | Missense_Mutation | disease causing | . |
| FAU | chr11 | 65120749 | A | G | Missense_Mutation | disease causing | . |
| FBN2 | chr5 | 128376839 | T | C | Missense_Mutation | disease causing | . |
| FBXO10 | chr9 | 37541445 | A | G | Silent | . | . |
| FBXO21 | chr12 | 117165555 | T | C | Missense_Mutation | disease causing | . |
| FBXO28 | chr1 | 224130539 | A | G | Missense_Mutation | disease causing | . |
| FBXO31 | chr16 | 87335379 | A | G | Silent | . | . |
| FBXO38 | chr5 | 148399000 | T | C | Missense_Mutation | disease causing | . |
| FBXO4 | chr5 | 41927075 | T | C | Silent | . | . |
| FBXW12 | chr3 | 48379488 | T | C | Missense_Mutation | polymorphism | . |
| FCHSD1 | chr5 | 141649431 | A | G | Silent | . | . |
| FCRLA | chr1 | 161711965 | A | G | Silent | . | . |
| FDPS | chr1 | 155320541 | T | C | Missense_Mutation | polymorphism | . |
| FER1L5 | chr2 | 96700443 | A | G | Missense_Mutation | . | . |
| FER1L6 | chr8 | 124061926 | T | C | Silent | . | . |
| FETUB | chr3 | 186646301 | T | C | Silent | . | . |
| FGF23 | chr12 | 4370728 | T | C | Missense_Mutation | disease causing | . |
| FGGY | chr1 | 59346339 | T | C | Missense_Mutation | disease causing | . |
| FIG4 | chr6 | 109825234 | A | G | Missense_Mutation | disease causing | . |
| FIGN | chr2 | 163610568 | A | G | Missense_Mutation | disease causing | . |
| FILIP1L | chr3 | 99848944 | A | G | Missense_Mutation | disease causing | . |
| FILIP1L | chr3 | 99849356 | A | T | Missense_Mutation | disease causing | . |
| FKBP3 | chr14 | 45129890 | A | G | Silent | . | . |
| FNDC1 | chr6 | 159251316 | T | C | Missense_Mutation | disease causing | . |
| FNDC3A | chr13 | 49201842 | A | G | Silent | . | . |
| FOCAD | chr9 | 20789393 | A | G | Missense_Mutation | polymorphism | . |
| FOXB1 | chr15 | 60005015 | T | C | Missense_Mutation | disease causing | . |
| FOXG1 | chr14 | 28768399 | T | C | Missense_Mutation | disease causing | . |
| FOXN1 | chr17 | 28527332 | T | C | Missense_Mutation | disease causing | . |
| FOXRED2 | chr22 | 36506170 | A | G | Missense_Mutation | polymorphism | . |
| FRMPD3 | chrX | 107602466 | A | G | Missense_Mutation | polymorphism | . |
| FSD2 | chr15 | 82765164 | A | G | Splice_Site | disease causing | . |
| FUNDC2 | chrX | 155051688 | T | C | Missense_Mutation | disease causing | . |
| FZD2 | chr17 | 44557974 | T | C | Missense_Mutation | disease causing | . |
| FZD6 | chr8 | 103318657 | T | C | Missense_Mutation | disease causing | . |
| GABPB2 | chr1 | 151090464 | T | C | Missense_Mutation | disease causing | . |
| GABRA1 | chr5 | 161891031 | A | G | Silent | . | . |
| GABRA5 | chr15 | 26937191 | A | G | Missense_Mutation | disease causing | . |
| GABRB2 | chr5 | 161294028 | T | C | 3'UTR | . | . |
| GABRR3 | chr3 | 97992959 | A | G | Missense_Mutation | . | . |
| GAD1 | chr2 | 170859747 | T | C | Silent | . | . |
| GAK | chr4 | 893924 | T | C | Missense_Mutation | disease causing | . |
| GALK2 | chr15 | 49217277 | A | T | Missense_Mutation | polymorphism | . |
| GALNT4 | chr12 | 89523963 | A | G | Missense_Mutation | disease causing | . |
| GAPVD1 | chr9 | 125299050 | T | C | Silent | . | . |
| GBA | chr1 | 155239630 | T | C | Missense_Mutation | disease causing | . |
| GBP5 | chr1 | 89267462 | T | C | Missense_Mutation | disease causing | . |
| GDF5 | chr20 | 35434750 | T | C | Missense_Mutation | disease causing | . |
| GDF9 | chr5 | 132861694 | T | C | Silent | . | . |
| GDI1 | chrX | 154442164 | T | C | Missense_Mutation | disease causing | . |
| GDI2 | chr10 | 5766584 | T | C | Missense_Mutation | disease causing | . |
| GGTLC1 | chr20 | 23986108 | A | T | Missense_Mutation | polymorphism | . |
| GLIPR1L2 | chr12 | 75422947 | A | G | Missense_Mutation | polymorphism | . |
| GLO1 | chr6 | 38702976 | T | C | Missense_Mutation | disease causing | . |
| GLRA3 | chr4 | 174677223 | T | C | Missense_Mutation | disease causing | . |
| GLUD2 | chrX | 121047688 | T | C | Missense_Mutation | disease causing | . |
| GMPPB | chr3 | 49721882 | A | G | Missense_Mutation | disease causing | . |
| GMPR | chr6 | 16278822 | T | C | Missense_Mutation | disease causing | . |
| GNAZ | chr22 | 23123253 | A | T | Missense_Mutation | disease causing | . |
| GNPTG | chr16 | 1361873 | T | C | Missense_Mutation | disease causing | . |
| GPC1 | chr2 | 240462316 | T | C | Missense_Mutation | disease causing | . |
| GPC2 | chr7 | 100172121 | T | C | Missense_Mutation | polymorphism | . |
| GPR12 | chr13 | 26759400 | T | A | Missense_Mutation | disease causing | . |
| GPR132 | chr14 | 105051992 | A | G | Missense_Mutation | disease causing | . |
| GPR151 | chr5 | 146515541 | T | C | Silent | . | . |
| GRAMD4 | chr22 | 46672914 | T | A | Missense_Mutation | disease causing | . |
| GRASP | chr12 | 52014162 | T | C | Missense_Mutation | disease causing | . |
| GRIA1 | chr5 | 153795550 | A | G | Intron | disease causing | . |
| GRIA2 | chr4 | 157312783 | T | C | Missense_Mutation | disease causing | . |
| GRID1 | chr10 | 85856027 | A | G | Splice_Site | disease causing | . |
| GRM1 | chr6 | 146398884 | A | G | Silent | . | . |
| GRM6 | chr5 | 178989064 | A | G | Missense_Mutation | disease causing | . |
| GRM8 | chr7 | 126769917 | A | G | Silent | . | . |
| GRN | chr17 | 44352723 | T | C | Silent | . | . |
| GTF2IRD1 | chr7 | 74558977 | A | G | Missense_Mutation | disease causing | . |
| GTF3C5 | chr9 | 133031097 | A | G | Missense_Mutation | disease causing | . |
| GUCY2C | chr12 | 14683064 | T | A | Missense_Mutation | polymorphism | . |
| GUSB | chr7 | 65980319 | A | G | Missense_Mutation | disease causing | . |
| HAPLN3 | chr15 | 88879093 | A | G | Missense_Mutation | disease causing | . |
| HEATR1 | chr1 | 236582734 | A | C | Splice_Site | disease causing | . |
| HECTD4 | chr12 | 112212572 | T | C | Missense_Mutation | disease causing | . |
| HEG1 | chr3 | 125012769 | T | C | Missense_Mutation | polymorphism | . |
| HELLS | chr10 | 94554165 | T | C | Missense_Mutation | disease causing | . |
| HELZ | chr17 | 67128680 | T | C | Missense_Mutation | polymorphism | . |
| HERC1 | chr15 | 63612420 | T | C | Missense_Mutation | disease causing | . |
| HERC2 | chr15 | 28121392 | A | G | Missense_Mutation | disease causing | . |
| HERC2 | chr15 | 28198622 | A | T | Missense_Mutation | disease causing | . |
| HERC2 | chr15 | 28202345 | A | G | Splice_Site | disease causing | . |
| HIF3A | chr19 | 46297104 | T | C | Splice_Site | disease causing | . |
| HIP1R | chr12 | 122858236 | T | C | Silent | . | . |
| HIRA | chr22 | 19355787 | T | C | Missense_Mutation | disease causing | . |
| HIST1H2BD | chr6 | 26158534 | A | G | Missense_Mutation | disease causing | . |
| HIST1H2BL | chr6 | 27807779 | T | C | Missense_Mutation | disease causing | . |
| HIST1H4G | chr6 | 26246773 | A | G | Missense_Mutation | polymorphism | . |
| HIVEP3 | chr1 | 41584022 | T | C | Missense_Mutation | disease causing | . |
| HLA-DQB1 | chr6 | 32664942 | A | G | Missense_Mutation | polymorphism | . |
| HMGCLL1 | chr6 | 55435641 | T | C | 3'UTR | . | . |
| HNF4A | chr20 | 44406222 | T | C | Missense_Mutation | disease causing | . |
| HOMER1 | chr5 | 79451106 | T | C | Missense_Mutation | disease causing | . |
| HOOK2 | chr19 | 12771468 | A | G | Missense_Mutation | disease causing | . |
| HRH2 | chr5 | 175683582 | T | C | Missense_Mutation | disease causing | . |
| HRH4 | chr18 | 24476906 | T | C | Missense_Mutation | disease causing | . |
| HS1BP3 | chr2 | 20640970 | T | C | Splice_Region | disease causing | . |
| HSDL2 | chr9 | 112404010 | T | C | Silent | . | . |
| HTR5A | chr7 | 155071452 | T | C | Missense_Mutation | disease causing | . |
| HVCN1 | chr12 | 110651214 | T | C | Splice_Region | . | . |
| HYAL2 | chr3 | 50318398 | T | C | Missense_Mutation | polymorphism | . |
| IBTK | chr6 | 82194615 | T | C | Missense_Mutation | polymorphism | . |
| IFI16 | chr1 | 159052092 | A | G | Silent | . | . |
| IFNGR2 | chr21 | 33421531 | T | C | Silent | . | . |
| IGF2BP3 | chr7 | 23319136 | A | G | Splice_Site | disease causing | . |
| IGSF9B | chr11 | 133930982 | A | G | Splice_Site | disease causing | . |
| IGSF9B | chr11 | 133935632 | A | G | Missense_Mutation | disease causing | . |
| IL12A | chr3 | 159989181 | A | T | Splice_Region | . | . |
| IL31RA | chr5 | 55890016 | A | G | Missense_Mutation | disease causing | . |
| IL4I1 | chr19 | 49894413 | T | C | Missense_Mutation | disease causing | . |
| IMMP1L | chr11 | 31433457 | T | C | Splice_Region | . | . |
| INSL4 | chr9 | 5233697 | T | C | Silent | . | . |
| INSM1 | chr20 | 20369151 | A | G | Missense_Mutation | disease causing | . |
| IQSEC2 | chrX | 53239254 | T | C | Missense_Mutation | disease causing | . |
| IRX5 | chr16 | 54931265 | T | C | Missense_Mutation | disease causing | . |
| ISG15 | chr1 | 1013531 | T | C | 5'UTR | . | . |
| ISLR2 | chr15 | 74134153 | T | C | Missense_Mutation | disease causing | . |
| ITFG1 | chr16 | 47375905 | T | C | Missense_Mutation | polymorphism | . |
| ITIH5 | chr10 | 7585928 | A | T | Missense_Mutation | disease causing | . |
| IZUMO2 | chr19 | 50152645 | A | G | Silent | . | . |
| JADE1 | chr4 | 128871486 | A | G | Missense_Mutation | disease causing | . |
| JAML | chr11 | 118196839 | T | C | Intron | . | . |
| JARID2 | chr6 | 15501071 | T | C | Missense_Mutation | disease causing | . |
| JKAMP | chr14 | 59495152 | A | G | Missense_Mutation | disease causing | . |
| JMJD1C | chr10 | 63184646 | A | G | Missense_Mutation | disease causing | . |
| JPH1 | chr8 | 74245108 | T | C | Silent | . | . |
| JUNB | chr19 | 12792394 | A | G | Missense_Mutation | disease causing | . |
| KANK4 | chr1 | 62266730 | A | G | Splice_Site | disease causing | . |
| KANSL1L | chr2 | 210154137 | A | G | Missense_Mutation | polymorphism | . |
| KAT14 | chr20 | 18187347 | A | G | Missense_Mutation | disease causing | . |
| KCNA2 | chr1 | 110604580 | T | A | Missense_Mutation | disease causing | . |
| KCNAB2 | chr1 | 6085215 | T | C | Missense_Mutation | disease causing | . |
| KCNK1 | chr1 | 233650043 | A | G | Intron | polymorphism | . |
| KCNQ5 | chr6 | 73192693 | T | C | Splice_Site | disease causing | . |
| KCTD1 | chr18 | 26476638 | A | G | Silent | . | . |
| KCTD4 | chr13 | 45193687 | T | C | 3'UTR | . | . |
| KDM4B | chr19 | 5047629 | T | C | Missense_Mutation | disease causing | . |
| KDM4C | chr9 | 7013988 | A | G | Silent | . | . |
| KDM5D | chrY | 19715731 | T | C | Missense_Mutation | disease causing | . |
| KIAA0196 | chr8 | 125057591 | A | C | Missense_Mutation | disease causing | . |
| KIAA0232 | chr4 | 6860902 | T | C | Missense_Mutation | disease causing | . |
| KIAA0319L | chr1 | 35554398 | A | G | Missense_Mutation | polymorphism | . |
| KIAA0430 | chr16 | 15609578 | T | C | Missense_Mutation | disease causing | . |
| KIAA1024 | chr15 | 79457081 | T | C | Missense_Mutation | disease causing | . |
| KIAA1161 | chr9 | 34372837 | T | C | Missense_Mutation | disease causing | . |
| KIAA1191 | chr5 | 176350639 | A | C | Missense_Mutation | disease causing | . |
| KIAA1324 | chr1 | 109200184 | T | C | Silent | . | . |
| KIF18A | chr11 | 28097894 | T | C | Silent | . | . |
| KIF24 | chr9 | 34256010 | T | C | Silent | . | . |
| KIF3C | chr2 | 25951904 | A | G | Missense_Mutation | disease causing | . |
| KIF6 | chr6 | 39596178 | A | G | Missense_Mutation | disease causing | . |
| KIF9 | chr3 | 47277369 | A | G | Silent | . | . |
| KLC1 | chr14 | 103654827 | T | C | Splice_Site | disease causing | . |
| KLF12 | chr13 | 73715365 | T | C | Splice_Region | . | . |
| KLF17 | chr1 | 44130593 | T | A | Missense_Mutation | polymorphism | . |
| KLF3 | chr4 | 38689730 | A | G | Splice_Region | . | . |
| KLF8 | chrX | 56270277 | A | G | Missense_Mutation | disease causing | . |
| KLHL11 | chr17 | 41865010 | A | G | Missense_Mutation | disease causing | . |
| KLHL2 | chr4 | 165238896 | A | G | Silent | . | . |
| KLHL22 | chr22 | 20457915 | A | G | Missense_Mutation | disease causing | . |
| KNDC1 | chr10 | 133224742 | A | G | Missense_Mutation | disease causing | . |
| KRIT1 | chr7 | 92201319 | T | C | Silent | . | . |
| KRT39 | chr17 | 40966778 | T | C | Missense_Mutation | polymorphism | . |
| KRTAP13-1 | chr21 | 30396683 | T | C | 3'UTR | . | . |
| KRTAP5-8 | chr11 | 71538758 | A | G | 3'UTR | . | . |
| LAMA4 | chr6 | 112131069 | A | T | Silent | . | . |
| LARP1 | chr5 | 154804309 | T | C | Splice_Site | disease causing | . |
| LARP6 | chr15 | 70831892 | T | C | 3'UTR | . | . |
| LCE2C | chr1 | 152676075 | T | C | Silent | . | . |
| LCN8 | chr9 | 136755418 | A | G | Missense_Mutation | polymorphism | . |
| LCTL | chr15 | 66553139 | A | G | Missense_Mutation | disease causing | . |
| LGALS14 | chr19 | 39706599 | A | G | Splice_Region | . | . |
| LIMCH1 | chr4 | 41619310 | T | C | Missense_Mutation | disease causing | . |
| LIN54 | chr4 | 82984165 | A | G | Missense_Mutation | disease causing | . |
| LINC00680-GUSBP4 | chr6 | 57961438 | A | G | 5'Flank | . | . |
| LINC00691 | chr3 | 24097118 | A | G | RNA | . | . |
| LINC00696 | chr3 | 52063337 | T | C | IGR | . | . |
| LIPJ | chr10 | 88594766 | T | C | Silent | . | . |
| LNP1 | chr3 | 100451807 | T | C | Missense_Mutation | polymorphism | . |
| LNPEP | chr5 | 97026751 | T | C | Missense_Mutation | polymorphism | . |
| LOC101929415 | chr8 | 56445757 | T | C | Intron | polymorphism | . |
| LOC105378828 | chr1 | 86642558 | T | C | Intron | . | . |
| LOC340113-TARS | chr5 | 33162455 | T | C | Intron | . | . |
| LOC349160 | chr7 | 137164318 | A | G | Intron | . | . |
| LOX | chr5 | 122074028 | A | G | Silent | . | . |
| LPA | chr6 | 160532648 | A | G | Splice_Region | . | . |
| LPAR2 | chr19 | 19624429 | A | G | Missense_Mutation | disease causing | . |
| LPCAT1 | chr5 | 1466886 | T | C | Missense_Mutation | disease causing | . |
| LPIN3 | chr20 | 41357901 | T | G | Missense_Mutation | disease causing | . |
| LRBA | chr4 | 150315607 | T | C | Silent | . | . |
| LRCH3 | chr3 | 197832276 | A | G | Missense_Mutation | disease causing | . |
| LRFN1 | chr19 | 39313964 | T | C | Missense_Mutation | disease causing | . |
| LRP2 | chr2 | 169275098 | T | G | Missense_Mutation | polymorphism | . |
| LRP2BP | chr4 | 185378079 | A | G | Splice_Site | disease causing | . |
| LRP5 | chr11 | 68425109 | T | C | Missense_Mutation | disease causing | . |
| LRP5 | chr11 | 68425294 | T | C | Splice_Site | disease causing | . |
| LRRC37A8P | chr17 | 35909482 | T | C | RNA | . | . |
| LRRC41 | chr1 | 46278854 | T | C | 3'UTR | disease causing | . |
| LRRC8E | chr19 | 7895691 | T | C | Missense_Mutation | disease causing | . |
| LSM14B | chr20 | 62124686 | A | G | Missense_Mutation | disease causing | . |
| LSR | chr19 | 35250394 | T | C | Silent | . | . |
| LTN1 | chr21 | 28953259 | A | G | Missense_Mutation | polymorphism | . |
| LTN1 | chr21 | 28981125 | T | C | Silent | . | . |
| LYST | chr1 | 235806570 | T | C | Missense_Mutation | polymorphism | . |
| MAB21L3 | chr1 | 116124150 | T | C | Missense_Mutation | disease causing | . |
| MAG | chr19 | 35295909 | T | C | Missense_Mutation | disease causing | . |
| MAG | chr19 | 35310026 | T | C | Missense_Mutation | disease causing | . |
| MAGIX | chrX | 49164841 | A | T | Intron | . | . |
| MAN2A2 | chr15 | 90918251 | T | A | Missense_Mutation | disease causing | . |
| MAN2B1 | chr19 | 12665715 | A | G | Missense_Mutation | disease causing | . |
| MAOA | chrX | 43744485 | A | T | Missense_Mutation | polymorphism | . |
| MAP2 | chr2 | 209694508 | A | G | Missense_Mutation | disease causing | . |
| MAP2K7 | chr19 | 7910132 | A | G | Splice_Region | . | . |
| MAP3K4 | chr6 | 161073517 | T | C | Missense_Mutation | disease causing | . |
| MAP6 | chr11 | 75587836 | T | C | Silent | . | . |
| MAPK8 | chr10 | 48427145 | T | C | Splice_Site | disease causing | . |
| MARCH3 | chr5 | 126870778 | T | C | Missense_Mutation | disease causing | . |
| MARS2 | chr2 | 197706927 | A | T | Missense_Mutation | disease causing | . |
| MBD5 | chr2 | 148470100 | T | A | Missense_Mutation | disease causing | . |
| MBTPS1 | chr16 | 84063352 | A | G | Missense_Mutation | disease causing | . |
| MBTPS1 | chr16 | 84067716 | A | G | Missense_Mutation | disease causing | . |
| MCM3AP | chr21 | 46265991 | A | G | Missense_Mutation | disease causing | . |
| MDN1 | chr6 | 89751491 | T | C | Missense_Mutation | disease causing | . |
| ME2 | chr18 | 50920526 | T | C | Missense_Mutation | disease causing | . |
| MED13 | chr17 | 61982636 | T | C | Missense_Mutation | disease causing | . |
| MED14 | chrX | 40692851 | A | G | Missense_Mutation | disease causing | . |
| MEF2D | chr1 | 156482489 | T | C | Missense_Mutation | disease causing | . |
| MELTF | chr3 | 197016227 | T | C | Missense_Mutation | disease causing | . |
| MEPE | chr4 | 87845747 | T | C | Silent | . | . |
| METTL13 | chr1 | 171792237 | T | C | Splice_Site | disease causing | . |
| METTL25 | chr12 | 82477292 | A | G | Silent | . | . |
| MFAP3L | chr4 | 169991474 | T | C | Silent | . | . |
| MFSD6L | chr17 | 8797781 | T | C | Missense_Mutation | disease causing | . |
| MGEA5 | chr10 | 101800384 | A | G | Silent | . | . |
| MIDN | chr19 | 1257112 | A | G | Missense_Mutation | disease causing | . |
| MIGA1 | chr1 | 77783334 | T | C | Missense_Mutation | disease causing | . |
| MIGA1 | chr1 | 77861296 | A | G | Missense_Mutation | disease causing | . |
| MINPP1 | chr10 | 87505279 | A | G | Missense_Mutation | polymorphism | . |
| MIR4436A-LOC101927050 | chr2 | 88885975 | A | G | Missense_Mutation | . | . |
| MIR4539 | chr14 | 105854389 | T | A | Missense_Mutation | . | . |
| MITD1 | chr2 | 99173936 | A | G | Missense_Mutation | disease causing | . |
| MKI67 | chr10 | 128122929 | A | G | Missense_Mutation | polymorphism | . |
| MMRN1 | chr4 | 89935940 | A | G | Missense_Mutation | polymorphism | . |
| MORF4L1 | chr15 | 78886170 | T | A | Missense_Mutation | disease causing | . |
| MPHOSPH9 | chr12 | 123163957 | A | G | Silent | . | . |
| MPP5 | chr14 | 67321254 | A | G | Missense_Mutation | disease causing | . |
| MRC2 | chr17 | 62680835 | T | A | Missense_Mutation | disease causing | . |
| MROH8 | chr20 | 37137861 | T | C | Missense_Mutation | disease causing | . |
| MRPL13 | chr8 | 120414034 | A | G | Missense_Mutation | disease causing | . |
| MRPL14 | chr6 | 44114167 | T | C | Silent | . | . |
| MRPS2 | chr9 | 135503699 | T | C | Missense_Mutation | polymorphism | . |
| MRS2 | chr6 | 24403238 | T | C | Splice_Site | disease causing | . |
| MS4A1 | chr11 | 60463098 | T | C | Missense_Mutation | disease causing | . |
| MSI1 | chr12 | 120345599 | A | G | Missense_Mutation | disease causing | . |
| MSR1 | chr8 | 16140866 | T | C | Intron | . | . |
| MSTO1 | chr1 | 155614652 | A | G | 3'UTR | . | . |
| MTFR1 | chr8 | 65669916 | A | G | 5'UTR | . | . |
| MTFR1 | chr8 | 65707035 | T | C | Silent | . | . |
| MTR | chr1 | 236824185 | T | C | Silent | . | . |
| MTR | chr1 | 236831992 | T | C | Missense_Mutation | disease causing | . |
| MUC17 | chr7 | 101035389 | A | G | Missense_Mutation | polymorphism | . |
| MYEF2 | chr15 | 48152204 | A | G | Intron | . | . |
| MYH7B | chr20 | 34993205 | T | C | Missense_Mutation | disease causing | . |
| MYO15A | chr17 | 18120041 | T | C | Missense_Mutation | polymorphism | . |
| MYO19 | chr17 | 36506995 | A | G | Missense_Mutation | disease causing | . |
| MYO5C | chr15 | 52247455 | T | C | Splice_Region | . | . |
| MYO7A | chr11 | 77174916 | T | C | Splice_Site | disease causing | . |
| MYO9A | chr15 | 71898543 | A | G | Silent | . | . |
| MYOM1 | chr18 | 3126755 | T | C | Silent | . | . |
| MYOM2 | chr8 | 2072369 | A | T | Missense_Mutation | disease causing | . |
| N4BP1 | chr16 | 48546243 | T | C | Missense_Mutation | disease causing | . |
| N4BP3 | chr5 | 178121910 | A | G | Missense_Mutation | disease causing | . |
| NADK | chr1 | 1765226 | A | G | Splice_Site | disease causing | . |
| NALCN | chr13 | 101082857 | T | G | Silent | . | . |
| NALCN | chr13 | 101082858 | A | G | Missense_Mutation | disease causing | . |
| NARS | chr18 | 57620643 | A | G | Missense_Mutation | disease causing | . |
| NAV3 | chr12 | 78006601 | A | G | Missense_Mutation | polymorphism | . |
| NAV3 | chr12 | 78021769 | A | G | Missense_Mutation | disease causing | . |
| NBAS | chr2 | 15461327 | A | G | Missense_Mutation | disease causing | . |
| NBEAL2 | chr3 | 47004528 | T | C | Missense_Mutation | disease causing | . |
| NCBP3 | chr17 | 3818455 | T | C | Missense_Mutation | disease causing | . |
| NCKAP1 | chr2 | 182953238 | T | C | Silent | . | . |
| NDUFA2 | chr5 | 140641855 | T | C | Intron | . | . |
| NEB | chr2 | 151644008 | T | C | Missense_Mutation | disease causing | . |
| NEDD9 | chr6 | 11190134 | A | G | Missense_Mutation | polymorphism | . |
| NEFL | chr8 | 24955798 | A | G | Missense_Mutation | . | . |
| NEIL2 | chr8 | 11786078 | T | C | Silent | . | . |
| NEK5 | chr13 | 52089302 | T | C | Missense_Mutation | polymorphism | . |
| NEK5 | chr13 | 52127472 | T | C | Missense_Mutation | disease causing | . |
| NEMF | chr14 | 49802667 | A | G | Splice_Site | disease causing | . |
| NEURL4 | chr17 | 7323120 | A | G | Silent | . | . |
| NEURL4 | chr17 | 7326283 | T | C | Missense_Mutation | disease causing | . |
| NEUROG2 | chr4 | 112515227 | T | C | Silent | . | . |
| NFATC2IP | chr16 | 28959000 | T | C | Missense_Mutation | disease causing | . |
| NFX1 | chr9 | 33290544 | A | G | 5'UTR | . | . |
| NIP7 | chr16 | 69339722 | A | G | 5'UTR | . | . |
| NIPAL3 | chr1 | 24449527 | T | C | Silent | . | . |
| NLGN3 | chrX | 71167086 | A | G | Missense_Mutation | disease causing | . |
| NLGN4X | chrX | 5893179 | A | G | Missense_Mutation | disease causing | . |
| NLRC4 | chr2 | 32224464 | A | G | 3'UTR | . | . |
| NLRP3 | chr1 | 247423313 | T | C | Missense_Mutation | polymorphism | . |
| NMD3 | chr3 | 161233401 | A | G | Splice_Region | . | . |
| NOMO2 | chr16 | 18531507 | A | G | Missense_Mutation | polymorphism | . |
| NPHP3 | chr3 | 132681881 | A | G | 3'UTR | . | . |
| NPHP3 | chr3 | 132683505 | A | G | Missense_Mutation | disease causing | . |
| NPHS1 | chr19 | 35841770 | A | G | Silent | . | . |
| NPR3 | chr5 | 32712287 | T | C | Missense_Mutation | disease causing | . |
| NPY2R | chr4 | 155214847 | A | G | Missense_Mutation | disease causing | . |
| NR1I3 | chr1 | 161231408 | T | C | Silent | polymorphism | . |
| NR2C1 | chr12 | 95028400 | T | C | Silent | . | . |
| NR3C2 | chr4 | 148435877 | A | G | Silent | . | . |
| NRAP | chr10 | 113597178 | A | G | Missense_Mutation | disease causing | . |
| NT5DC2 | chr3 | 52529182 | T | C | Missense_Mutation | polymorphism | . |
| NTAN1 | chr16 | 15038639 | A | G | Missense_Mutation | disease causing | . |
| NTNG1 | chr1 | 107324664 | A | G | Missense_Mutation | disease causing | . |
| NUDCD2 | chr5 | 163459986 | T | C | Missense_Mutation | disease causing | . |
| NUDCD2 | chr5 | 163460015 | T | C | Silent | . | . |
| NUP133 | chr1 | 229508213 | T | A | Missense_Mutation | polymorphism | . |
| OBSCN | chr1 | 228268646 | T | C | Missense_Mutation | disease causing | . |
| OBSCN | chr1 | 228278832 | T | C | Missense_Mutation | disease causing | . |
| OBSL1 | chr2 | 219554619 | T | C | Silent | . | . |
| OCA2 | chr15 | 27955155 | T | C | Splice_Region | . | . |
| ODF3B | chr22 | 50531260 | A | G | Missense_Mutation | disease causing | . |
| OGG1 | chr3 | 9750981 | A | G | Silent | . | . |
| OGG1 | chr3 | 9759803 | A | G | 3'Flank | . | . |
| OGN | chr9 | 92390001 | A | G | Silent | . | . |
| OPCML | chr11 | 132657194 | T | C | Missense_Mutation | disease causing | . |
| OPHN1 | chrX | 68192973 | A | G | Missense_Mutation | disease causing | . |
| OPRM1 | chr6 | 154093328 | A | G | Intron | polymorphism | . |
| OR10J3 | chr1 | 159314554 | A | G | Missense_Mutation | polymorphism | . |
| OR13F1 | chr9 | 104504567 | A | G | Missense_Mutation | disease causing | . |
| OR1L3 | chr9 | 122675637 | A | G | Missense_Mutation | polymorphism | . |
| OR1Q1 | chr9 | 122614840 | T | C | Missense_Mutation | disease causing | . |
| OR2D2 | chr11 | 6892173 | T | C | Missense_Mutation | polymorphism | . |
| OR2J3 | chr6 | 29112077 | T | C | Missense_Mutation | disease causing | . |
| OR2T33 | chr1 | 248273223 | A | G | Missense_Mutation | polymorphism | . |
| OR2V2 | chr5 | 181155008 | T | C | Silent | . | . |
| OR4E1-DAD1 | chr14 | 21797750 | T | C | Missense_Mutation | . | . |
| OR4E1-DAD1 | chr14 | 21894894 | T | C | Missense_Mutation | . | . |
| OR4E1-DAD1 | chr14 | 22124264 | T | C | Missense_Mutation | . | . |
| OR51E2 | chr11 | 4681972 | A | G | Missense_Mutation | polymorphism | . |
| OR52I1 | chr11 | 4594153 | T | C | Missense_Mutation | . | . |
| OR52N4 | chr11 | 5754974 | T | C | Silent | . | . |
| OR5D18 | chr11 | 55819810 | T | C | Missense_Mutation | disease causing | . |
| OR5T1 | chr11 | 56275989 | T | C | Silent | . | . |
| OR5T2 | chr11 | 56233178 | T | C | Missense_Mutation | polymorphism | . |
| OR6F1 | chr1 | 247712652 | T | C | Missense_Mutation | disease causing | . |
| OR7E24 | chr19 | 9251930 | A | G | Missense_Mutation | polymorphism | . |
| OR7G3 | chr19 | 9126176 | A | G | Missense_Mutation | polymorphism | . |
| OR8B2 | chr11 | 124382627 | A | G | Silent | . | . |
| OR8B3 | chr11 | 124396716 | A | G | Silent | . | . |
| OR8G2 | chr11 | 124225126 | A | G | RNA | . | . |
| OR9G4 | chr11 | 56743365 | A | G | Silent | . | . |
| ORM1 | chr9 | 114323711 | T | C | Missense_Mutation | polymorphism | . |
| OSBP | chr11 | 59577006 | A | G | Missense_Mutation | disease causing | . |
| OSBPL6 | chr2 | 178392487 | T | A | Missense_Mutation | disease causing | . |
| OTUD7B | chr1 | 149959707 | A | G | Silent | . | . |
| OTX2 | chr14 | 56801936 | A | G | Silent | . | . |
| OXSR1 | chr3 | 38221689 | T | C | Splice_Site | disease causing | . |
| P2RY12 | chr3 | 151338197 | A | G | Missense_Mutation | disease causing | . |
| PACS1 | chr11 | 66235333 | T | C | Missense_Mutation | disease causing | . |
| PAF1 | chr19 | 39389336 | T | C | Missense_Mutation | disease causing | . |
| PAM | chr5 | 102948401 | A | G | Missense_Mutation | disease causing | . |
| PARG | chr10 | 49933915 | A | G | Missense_Mutation | . | . |
| PARP14 | chr3 | 122701237 | T | C | Missense_Mutation | polymorphism | . |
| PAXIP1 | chr7 | 154947901 | A | G | Splice_Site | disease causing | . |
| PBX3 | chr9 | 125963082 | A | G | Missense_Mutation | disease causing | . |
| PCDH17 | chr13 | 57634786 | A | G | Missense_Mutation | disease causing | . |
| PCDH17 | chr13 | 57724739 | A | C | Silent | . | . |
| PCDHA4 | chr5 | 140809066 | T | C | Missense_Mutation | disease causing | . |
| PCDHA6 | chr5 | 140828196 | A | C | Silent | . | . |
| PCDHA8 | chr5 | 140842873 | T | C | Missense_Mutation | disease causing | . |
| PCDHA8 | chr5 | 140842873 | T | C | Missense_Mutation | disease causing | . |
| PCDHB19P | chr5 | 141236281 | T | C | Intron | . | . |
| PCDHB6 | chr5 | 141157302 | A | G | 3'Flank | . | . |
| PCDHB6 | chr5 | 141157811 | A | G | 3'Flank | . | . |
| PCDHB8 | chr5 | 141179725 | A | G | Missense_Mutation | disease causing | . |
| PCDHGA10 | chr5 | 141415262 | A | G | Missense_Mutation | disease causing | . |
| PCDHGA3 | chr5 | 141346108 | A | G | Missense_Mutation | disease causing | . |
| PCDHGA9 | chr5 | 141403302 | A | G | Missense_Mutation | polymorphism | . |
| PCDHGB4 | chr5 | 141389569 | A | G | Missense_Mutation | disease causing | . |
| PCDHGC4 | chr5 | 141485523 | A | G | Missense_Mutation | disease causing | . |
| PCGF3 | chr4 | 737505 | A | G | Silent | . | . |
| PCID2 | chr13 | 113181221 | T | C | Missense_Mutation | disease causing | . |
| PCMTD1 | chr8 | 51860866 | T | C | Missense_Mutation | disease causing | . |
| PCNA | chr20 | 5115524 | A | G | Missense_Mutation | disease causing | . |
| PCSK4 | chr19 | 1487293 | T | C | Missense_Mutation | polymorphism | . |
| PDE12 | chr3 | 57557641 | A | G | Missense_Mutation | disease causing | . |
| PDE5A | chr4 | 119520933 | A | C | Splice_Site | disease causing | . |
| PDE7A | chr8 | 65726956 | A | G | Missense_Mutation | disease causing | . |
| PDE9A | chr21 | 42762085 | A | G | Missense_Mutation | disease causing | . |
| PDHX | chr11 | 34970210 | T | C | Silent | . | . |
| PDXK | chr21 | 43741721 | A | G | Missense_Mutation | disease causing | . |
| PDZD2 | chr5 | 31799382 | A | G | Missense_Mutation | disease causing | . |
| PDZD8 | chr10 | 117283215 | A | G | 3'UTR | . | . |
| PDZRN3 | chr3 | 73384422 | T | C | Missense_Mutation | disease causing | . |
| PGAP2 | chr11 | 3811263 | T | C | Missense_Mutation | disease causing | . |
| PHACTR3 | chr20 | 59755227 | T | C | Missense_Mutation | polymorphism | . |
| PHACTR3 | chr20 | 59845217 | A | G | Missense_Mutation | disease causing | . |
| PHC1 | chr12 | 8936891 | T | C | Missense_Mutation | disease causing | . |
| PHC1 | chr12 | 8936903 | T | A | Missense_Mutation | disease causing | . |
| PHF21A | chr11 | 45969869 | T | C | Silent | . | . |
| PHLDA1 | chr12 | 76030996 | T | C | Missense_Mutation | disease causing | . |
| PHPT1 | chr9 | 136849299 | A | G | 5'UTR | . | . |
| PI16 | chr6 | 36963035 | T | C | Silent | . | . |
| PI16 | chr6 | 36963035 | T | C | Silent | . | . |
| PIGL | chr17 | 16317820 | A | G | Missense_Mutation | disease causing | . |
| PIGM | chr1 | 160031115 | A | G | Missense_Mutation | polymorphism | . |
| PIGO | chr9 | 35091745 | A | G | Silent | . | . |
| PIK3R4 | chr3 | 130745117 | A | G | Silent | . | . |
| PIK3R5 | chr17 | 8890845 | T | C | Missense_Mutation | polymorphism | . |
| PITX3 | chr10 | 102230537 | A | G | Missense_Mutation | disease causing | . |
| PKHD1L1 | chr8 | 109429422 | T | C | Missense_Mutation | polymorphism | . |
| PKHD1L1 | chr8 | 109461866 | A | G | Silent | . | . |
| PLA2G4F | chr15 | 42145598 | T | C | Missense_Mutation | polymorphism | . |
| PLAA | chr9 | 26920383 | A | G | Splice_Region | . | . |
| PLAGL2 | chr20 | 32197326 | A | G | Missense_Mutation | disease causing | . |
| PLB1 | chr2 | 28516842 | T | C | Silent | . | . |
| PLCH1 | chr3 | 155497359 | A | G | Missense_Mutation | disease causing | . |
| PLCXD3 | chr5 | 41382090 | T | C | Missense_Mutation | disease causing | . |
| PLEC | chr8 | 143921230 | T | C | Missense_Mutation | disease causing | . |
| PLEC | chr8 | 143933203 | T | C | Missense_Mutation | disease causing | . |
| PLEKHM3 | chr2 | 208001576 | T | C | Missense_Mutation | polymorphism | . |
| PLIN2 | chr9 | 19120906 | T | C | Missense_Mutation | disease causing | . |
| PLK2 | chr5 | 58457570 | T | C | Missense_Mutation | disease causing | . |
| PLXNA1 | chr3 | 127017823 | T | C | Silent | . | . |
| PLXNA4 | chr7 | 132179881 | T | C | Missense_Mutation | disease causing | . |
| PLXNB2 | chr22 | 50275900 | A | G | Missense_Mutation | disease causing | . |
| PMM1 | chr22 | 41578803 | T | C | Splice_Region | . | . |
| PNMA3 | chrX | 153058330 | A | G | Silent | . | . |
| PODN | chr1 | 53078519 | T | C | Missense_Mutation | disease causing | . |
| POLH | chr6 | 43613991 | A | G | Missense_Mutation | polymorphism | . |
| POLR2A | chr17 | 7513886 | T | C | Silent | . | . |
| POLR3A | chr10 | 78013742 | T | C | Missense_Mutation | disease causing | . |
| POLR3D | chr8 | 22249311 | T | C | Splice_Site | disease causing | . |
| POMT1 | chr9 | 131523172 | T | C | 3'UTR | . | . |
| POTEC | chr18 | 14537969 | T | C | Silent | . | . |
| POU4F1 | chr13 | 78603383 | T | C | 5'UTR | . | . |
| PPIP5K2 | chr5 | 103173205 | T | C | Silent | . | . |
| PPIP5K2 | chr5 | 103173930 | A | G | Silent | . | . |
| PPP1R13L | chr19 | 45382685 | A | G | Missense_Mutation | disease causing | . |
| PPP1R1C | chr2 | 182063793 | T | C | Splice_Site | disease causing | . |
| PPP1R21 | chr2 | 48479925 | T | C | Splice_Region | . | . |
| PPP2R1B | chr11 | 111742118 | A | G | Missense_Mutation | disease causing | . |
| PPP2R3B | chrX | 338818 | T | C | Missense_Mutation | polymorphism | . |
| PREP | chr6 | 105288871 | A | G | Silent | . | . |
| PRICKLE2 | chr3 | 64198782 | A | G | Splice_Site | disease causing | . |
| PRIM1 | chr12 | 56741433 | A | G | Splice_Site | disease causing | . |
| PRKACB | chr1 | 84144469 | T | C | Intron | . | . |
| PRKD1 | chr14 | 29638745 | A | G | Missense_Mutation | disease causing | . |
| PRKDC | chr8 | 47902768 | T | C | Missense_Mutation | disease causing | . |
| PRKDC | chr8 | 47935739 | A | G | Silent | . | . |
| PRLR | chr5 | 35065028 | A | G | 3'UTR | . | . |
| PRR19 | chr19 | 42310735 | T | C | Missense_Mutation | polymorphism | . |
| PRUNE2 | chr9 | 76706664 | T | C | Silent | . | . |
| PRUNE2 | chr9 | 76709012 | A | G | Missense_Mutation | polymorphism | . |
| PSENEN | chr19 | 35745982 | T | C | Missense_Mutation | disease causing | . |
| PSMB2 | chr1 | 35641425 | T | C | Missense_Mutation | disease causing | . |
| PSPC1 | chr13 | 19782526 | A | G | Missense_Mutation | disease causing | . |
| PTCHD1 | chrX | 23393139 | T | C | Missense_Mutation | disease causing | . |
| PTGDR | chr14 | 52268410 | A | G | Missense_Mutation | polymorphism | . |
| PTPRF | chr1 | 43621202 | T | C | Silent | . | . |
| PTPRH | chr19 | 55198711 | T | C | Missense_Mutation | polymorphism | . |
| PTRH2 | chr17 | 59697934 | A | G | Silent | . | . |
| PUM1 | chr1 | 30941148 | T | C | Splice_Region | . | . |
| PXDNL | chr8 | 51409445 | A | G | Missense_Mutation | disease causing | . |
| PYGO1 | chr15 | 55546067 | T | C | Missense_Mutation | disease causing | . |
| RAB11FIP2 | chr10 | 118046067 | T | C | Missense_Mutation | disease causing | . |
| RABGAP1 | chr9 | 123074303 | T | C | Missense_Mutation | disease causing | . |
| RABGAP1L | chr1 | 174958025 | A | G | 3'UTR | . | . |
| RAD18 | chr3 | 8935907 | A | G | Missense_Mutation | disease causing | . |
| RAD23A | chr19 | 12948195 | A | G | Missense_Mutation | disease causing | . |
| RAD51AP1 | chr12 | 4545794 | T | A | Silent | . | . |
| RAI1 | chr17 | 17803851 | T | C | Splice_Site | disease causing | . |
| RASGRF2 | chr5 | 81225688 | A | G | Missense_Mutation | disease causing | . |
| RB1CC1 | chr8 | 52661582 | A | G | Silent | . | . |
| RBBP6 | chr16 | 24567224 | A | T | Silent | . | . |
| RBM23 | chr14 | 22905106 | T | C | Silent | . | . |
| RBM38 | chr20 | 57407641 | A | G | Missense_Mutation | disease causing | . |
| RCN3 | chr19 | 49539174 | A | T | Missense_Mutation | disease causing | . |
| REV3L | chr6 | 111365284 | A | G | Missense_Mutation | polymorphism | . |
| RFX1 | chr19 | 13966461 | A | G | Missense_Mutation | polymorphism | . |
| RGS12 | chr4 | 3317338 | T | C | Missense_Mutation | disease causing | . |
| RGS22 | chr8 | 99962392 | A | G | Splice_Site | . | . |
| RGS22 | chr8 | 100066211 | A | G | Missense_Mutation | polymorphism | . |
| RHOBTB3 | chr5 | 95783842 | A | G | Missense_Mutation | disease causing | . |
| RIF1 | chr2 | 151416624 | T | C | Missense_Mutation | polymorphism | . |
| RINL | chr19 | 38870761 | T | C | Missense_Mutation | polymorphism | . |
| RIOK3 | chr18 | 23477032 | T | C | Silent | . | . |
| RMND5B | chr5 | 178147759 | T | C | Missense_Mutation | disease causing | . |
| RNASE7 | chr14 | 21043354 | A | T | Missense_Mutation | disease causing | . |
| RNASE9 | chr14 | 20556910 | T | C | Missense_Mutation | polymorphism | . |
| RNF139 | chr8 | 124487379 | A | G | Missense_Mutation | disease causing | . |
| RNF144A | chr2 | 6996942 | T | C | Missense_Mutation | disease causing | . |
| RNF31 | chr14 | 24150640 | T | C | Missense_Mutation | disease causing | . |
| RNF34 | chr12 | 121417718 | T | C | Missense_Mutation | disease causing | . |
| RNFT1 | chr17 | 59963008 | T | A | Silent | . | . |
| ROR1 | chr1 | 64178205 | T | C | Missense_Mutation | disease causing | . |
| RPGRIP1 | chr14 | 21343133 | A | G | Missense_Mutation | disease causing | . |
| RPGRIP1L | chr16 | 53664867 | T | C | Splice_Region | . | . |
| RPL35 | chr9 | 124857950 | A | C | Missense_Mutation | disease causing | . |
| RPLP0 | chr12 | 120197381 | A | G | Missense_Mutation | disease causing | . |
| RPSA | chr3 | 39407762 | T | C | Missense_Mutation | disease causing | . |
| RPSAP58 | chr19 | 23827766 | A | G | Missense_Mutation | . | . |
| RSAD1 | chr17 | 50482386 | A | G | Missense_Mutation | disease causing | . |
| RTTN | chr18 | 70114443 | A | T | Splice_Site | disease causing | . |
| RTTN | chr18 | 70139700 | A | G | Missense_Mutation | polymorphism | . |
| RUBCN | chr3 | 197681325 | T | C | Missense_Mutation | disease causing | . |
| RYR3 | chr15 | 33503631 | T | C | Missense_Mutation | disease causing | . |
| SAAL1 | chr11 | 18086859 | T | C | Splice_Region | disease causing | . |
| SAC3D1 | chr11 | 65044620 | A | G | Missense_Mutation | polymorphism | . |
| SAMD9 | chr7 | 93104860 | T | C | Missense_Mutation | disease causing | . |
| SARS | chr1 | 109235993 | A | G | Missense_Mutation | disease causing | . |
| SARS2 | chr19 | 38915665 | A | G | Missense_Mutation | . | . |
| SASS6 | chr1 | 100122409 | A | G | Silent | . | . |
| SAT1 | chrX | 23785827 | T | C | Missense_Mutation | disease causing | . |
| SAXO2 | chr15 | 82271690 | T | C | Silent | . | . |
| SBF2 | chr11 | 9789141 | T | C | Missense_Mutation | polymorphism | . |
| SCEL | chr13 | 77591462 | T | C | Splice_Site | disease causing | . |
| SCML2 | chrX | 18242571 | T | C | Silent | . | . |
| SCN11A | chr3 | 38847741 | A | G | Splice_Region | . | . |
| SCNN1D | chr1 | 1287178 | T | C | Missense_Mutation | disease causing | . |
| SDK1 | chr7 | 3639005 | T | C | Missense_Mutation | disease causing | . |
| SDK1 | chr7 | 4077022 | A | G | Missense_Mutation | polymorphism | . |
| SEC14L1 | chr17 | 77209382 | A | G | Missense_Mutation | disease causing | . |
| SEC1P | chr19 | 48680421 | A | G | RNA | . | . |
| SEC23A | chr14 | 39067188 | A | G | Silent | . | . |
| SEC23IP | chr10 | 119904279 | T | C | Splice_Site | disease causing | . |
| SEL1L3 | chr4 | 25822028 | A | G | Missense_Mutation | disease causing | . |
| SEMA3G | chr3 | 52441347 | A | G | Missense_Mutation | disease causing | . |
| SENP7 | chr3 | 101364888 | A | G | Silent | . | . |
| SERPINI2 | chr3 | 167467269 | T | C | Silent | . | . |
| SETD9 | chr5 | 56913923 | A | G | Missense_Mutation | polymorphism | . |
| SETMAR | chr3 | 4313464 | A | G | Silent | . | . |
| SETX | chr9 | 132329473 | T | C | Missense_Mutation | polymorphism | . |
| SFRP1 | chr8 | 41265366 | T | C | Missense_Mutation | disease causing | . |
| SGK223 | chr8 | 8318629 | T | C | Missense_Mutation | disease causing | . |
| SGO2 | chr2 | 200572845 | A | G | Silent | . | . |
| SH2D1A | chrX | 124370189 | T | C | Missense_Mutation | disease causing | . |
| SH3BP2 | chr4 | 2820732 | A | G | Missense_Mutation | disease causing | . |
| SH3PXD2A | chr10 | 103601890 | A | G | Missense_Mutation | disease causing | . |
| SH3TC1 | chr4 | 8227439 | A | T | Missense_Mutation | disease causing | . |
| SHPRH | chr6 | 145941792 | A | G | Missense_Mutation | disease causing | . |
| SI | chr3 | 165018066 | A | G | Missense_Mutation | disease causing | . |
| SIGLEC5 | chr19 | 51626044 | A | G | Silent | . | . |
| SIGMAR1 | chr9 | 34635786 | T | C | Missense_Mutation | disease causing | . |
| SIRT7 | chr17 | 81914291 | T | C | Splice_Region | . | . |
| SLAMF9 | chr1 | 159952259 | T | C | Splice_Region | . | . |
| SLC12A2 | chr5 | 128112853 | A | G | Missense_Mutation | disease causing | . |
| SLC12A7 | chr5 | 1083811 | A | G | Missense_Mutation | disease causing | . |
| SLC17A2 | chr6 | 25913288 | A | G | 3'Flank | . | . |
| SLC17A6 | chr11 | 22365640 | A | G | Missense_Mutation | disease causing | . |
| SLC25A10 | chr17 | 81715018 | T | C | Silent | . | . |
| SLC25A29 | chr14 | 100293376 | A | G | Missense_Mutation | disease causing | . |
| SLC25A31 | chr4 | 127730501 | A | G | 5'UTR | . | . |
| SLC2A3 | chr12 | 7930416 | A | G | Intron | . | . |
| SLC35A1 | chr6 | 87511418 | T | C | Silent | . | . |
| SLC38A1 | chr12 | 46204331 | A | G | Silent | . | . |
| SLC39A6 | chr18 | 36109514 | T | C | 3'UTR | . | . |
| SLC40A1 | chr2 | 189563995 | A | G | Missense_Mutation | disease causing | . |
| SLC44A4 | chr6 | 31865989 | T | C | Silent | . | . |
| SLC45A1 | chr1 | 8337923 | T | C | Missense_Mutation | disease causing | . |
| SLC45A4 | chr8 | 141215776 | A | G | Missense_Mutation | disease causing | . |
| SLC4A10 | chr2 | 161839808 | A | G | Silent | . | . |
| SLC5A4 | chr22 | 32224270 | T | C | Silent | . | . |
| SLC5A5 | chr19 | 17872678 | T | C | Splice_Site | disease causing | . |
| SLC6A1 | chr3 | 11034654 | T | C | Missense_Mutation | disease causing | . |
| SLCO5A1 | chr8 | 69673231 | A | G | Missense_Mutation | disease causing | . |
| SLIT3 | chr5 | 168708004 | T | C | Missense_Mutation | disease causing | . |
| SMARCA2 | chr9 | 2161726 | T | C | Missense_Mutation | disease causing | . |
| SMG1P5 | chr16 | 30299889 | T | C | RNA | . | . |
| SMG9 | chr19 | 43747439 | T | C | Splice_Region | . | . |
| SNAI3 | chr16 | 88681482 | T | C | Silent | . | . |
| SNAPC2 | chr19 | 7922537 | T | C | Missense_Mutation | polymorphism | . |
| SNIP1 | chr1 | 37540262 | T | C | Missense_Mutation | disease causing | . |
| SNRPD2 | chr19 | 45687680 | A | G | Missense_Mutation | disease causing | . |
| SNX18 | chr5 | 54543533 | A | G | 3'UTR | . | . |
| SNX30 | chr9 | 112817707 | T | C | Splice_Region | . | . |
| SOCS6 | chr18 | 70325822 | A | G | Missense_Mutation | disease causing | . |
| SOST | chr17 | 43755751 | T | G | Missense_Mutation | disease causing | . |
| SOX7 | chr8 | 10726624 | T | C | Missense_Mutation | disease causing | . |
| SPARCL1 | chr4 | 87482546 | T | C | Missense_Mutation | polymorphism | . |
| SPAST | chr2 | 32154456 | A | G | Missense_Mutation | disease causing | . |
| SPATA16 | chr3 | 172956775 | T | C | Missense_Mutation | polymorphism | . |
| SPEG | chr2 | 219480061 | T | C | Missense_Mutation | disease causing | . |
| SPEM1 | chr17 | 7421583 | A | G | Missense_Mutation | disease causing | . |
| SPG11 | chr15 | 44596337 | A | C | Missense_Mutation | polymorphism | . |
| SPG20 | chr13 | 36335148 | A | G | Missense_Mutation | disease causing | . |
| SPINK5 | chr5 | 148111818 | T | C | Silent | . | . |
| SPINK5 | chr5 | 148123962 | T | C | Splice_Site | disease causing | . |
| SPON2 | chr4 | 1170286 | T | C | Intron | . | . |
| SREBF1 | chr17 | 17820284 | T | C | Missense_Mutation | polymorphism | . |
| SREBF2 | chr22 | 41900982 | A | G | Intron | . | . |
| SRPRA | chr11 | 126264020 | A | G | Missense_Mutation | disease causing | . |
| ST7 | chr7 | 117209924 | T | C | Silent | disease causing | . |
| STAB1 | chr3 | 52505913 | T | C | Missense_Mutation | polymorphism | . |
| STK3 | chr8 | 98749326 | A | G | Missense_Mutation | disease causing | . |
| STK36 | chr2 | 218689922 | A | G | Missense_Mutation | polymorphism | . |
| STT3B | chr3 | 31576501 | T | C | Silent | . | . |
| STX11 | chr6 | 144186761 | A | G | Missense_Mutation | disease causing | . |
| STX8 | chr17 | 9505036 | A | G | Splice_Site | disease causing | . |
| STXBP4 | chr17 | 55000834 | A | G | Silent | . | . |
| SUB1 | chr5 | 32598976 | T | C | Missense_Mutation | disease causing | . |
| SUCLA2 | chr13 | 47996841 | A | G | Splice_Site | disease causing | . |
| SUDS3 | chr12 | 118403501 | T | C | Missense_Mutation | disease causing | . |
| SULF2 | chr20 | 47666485 | T | C | Missense_Mutation | disease causing | . |
| SUPT20H | chr13 | 37044076 | A | G | Splice_Site | disease causing | . |
| SUSD2 | chr22 | 24184810 | T | C | Missense_Mutation | polymorphism | . |
| SV2A | chr1 | 149908055 | A | G | Missense_Mutation | disease causing | . |
| SV2A | chr1 | 149913705 | A | G | Missense_Mutation | disease causing | . |
| SV2C | chr5 | 76174122 | A | G | Intron | . | . |
| SVEP1 | chr9 | 110379465 | T | C | Silent | . | . |
| SWT1 | chr1 | 185168365 | A | G | Missense_Mutation | polymorphism | . |
| SYNGAP1 | chr6 | 33440893 | A | G | Missense_Mutation | disease causing | . |
| SYNPO2L | chr10 | 73650906 | A | G | Intron | disease causing | . |
| SYNRG | chr17 | 37577569 | T | C | Missense_Mutation | disease causing | . |
| SYT17 | chr16 | 19183681 | A | G | Missense_Mutation | disease causing | . |
| TACR1 | chr2 | 75198640 | A | G | Missense_Mutation | disease causing | . |
| TAF7 | chr5 | 141319083 | A | G | Missense_Mutation | disease causing | . |
| TAF8 | chr6 | 42066316 | A | G | Missense_Mutation | disease causing | . |
| TAOK2 | chr16 | 29979457 | T | C | Missense_Mutation | disease causing | . |
| TARS | chr5 | 33460967 | T | C | Missense_Mutation | disease causing | . |
| TBR1 | chr2 | 161418920 | A | G | Missense_Mutation | disease causing | . |
| TBRG1 | chr11 | 124626966 | T | A | Missense_Mutation | disease causing | . |
| TBX22 | chrX | 80024119 | A | G | Missense_Mutation | disease causing | . |
| TCAF2P1 | chr7 | 143812965 | T | C | RNA | . | . |
| TCF21 | chr6 | 133889827 | T | C | Missense_Mutation | disease causing | . |
| TCTE3 | chr6 | 169743141 | T | C | Missense_Mutation | polymorphism | . |
| TDRD1 | chr10 | 114213406 | T | C | Missense_Mutation | polymorphism | . |
| TDRD6 | chr6 | 46693937 | A | T | Missense_Mutation | polymorphism | . |
| TENM3 | chr4 | 182754387 | A | G | Splice_Region | . | . |
| TEP1 | chr14 | 20403779 | A | G | Missense_Mutation | disease causing | . |
| TES | chr7 | 116250227 | T | A | Missense_Mutation | disease causing | . |
| TEX2 | chr17 | 64212953 | T | C | Missense_Mutation | disease causing | . |
| TEX2 | chr17 | 64213833 | T | C | Missense_Mutation | polymorphism | . |
| THG1L | chr5 | 157731508 | A | G | Missense_Mutation | polymorphism | . |
| THOC7 | chr3 | 63835185 | A | T | Missense_Mutation | disease causing | . |
| THSD7A | chr7 | 11474528 | T | C | Silent | . | . |
| THSD7B | chr2 | 137056436 | T | C | Silent | . | . |
| THSD7B | chr2 | 137115262 | A | T | Silent | . | . |
| THUMPD3 | chr3 | 9384617 | A | G | Missense_Mutation | disease causing | . |
| TIAM1 | chr21 | 31187042 | T | C | Missense_Mutation | disease causing | . |
| TIMM22 | chr17 | 1001158 | T | C | 3'UTR | . | . |
| TJP2 | chr9 | 69212581 | T | C | Missense_Mutation | disease causing | . |
| TLL1 | chr4 | 165994415 | A | G | Silent | . | . |
| TLR4 | chr9 | 117714484 | T | C | Missense_Mutation | disease causing | . |
| TLR9 | chr3 | 52222525 | A | G | Silent | . | . |
| TM9SF3 | chr10 | 96547970 | T | C | Missense_Mutation | disease causing | . |
| TM9SF4 | chr20 | 32157951 | A | G | Missense_Mutation | disease causing | . |
| TMC2 | chr20 | 2613234 | A | G | Missense_Mutation | disease causing | . |
| TMC7 | chr16 | 19023124 | A | G | Missense_Mutation | disease causing | . |
| TMEM132E | chr17 | 34637682 | A | G | Missense_Mutation | disease causing | . |
| TMEM133 | chr11 | 100992432 | T | C | Missense_Mutation | polymorphism | . |
| TMEM135 | chr11 | 87236668 | T | C | Missense_Mutation | disease causing | . |
| TMEM150A | chr2 | 85599006 | T | C | 3'UTR | . | . |
| TMEM168 | chr7 | 112775173 | T | C | Splice_Region | . | . |
| TMEM198 | chr2 | 219544827 | T | C | Missense_Mutation | disease causing | . |
| TMEM200A | chr6 | 130440976 | A | G | Missense_Mutation | polymorphism | . |
| TMEM39A | chr3 | 119447111 | A | G | Missense_Mutation | disease causing | . |
| TMEM41A | chr3 | 185496920 | A | G | Missense_Mutation | disease causing | . |
| TMEM8C | chr9 | 133518920 | T | C | Missense_Mutation | disease causing | . |
| TMEM94 | chr17 | 75471894 | T | C | 5'UTR | . | . |
| TMEM94 | chr17 | 75489642 | T | C | Missense_Mutation | disease causing | . |
| TMF1 | chr3 | 69047949 | A | G | Silent | . | . |
| TMLHE | chrX | 155511730 | T | C | Missense_Mutation | disease causing | . |
| TMPRSS11F | chr4 | 68062576 | A | G | Intron | . | . |
| TMPRSS13 | chr11 | 117918502 | A | G | Missense_Mutation | polymorphism | . |
| TNFAIP8L2 | chr1 | 151158843 | A | G | Missense_Mutation | disease causing | . |
| TNFRSF11A | chr18 | 62368728 | A | G | Missense_Mutation | polymorphism | . |
| TNFSF11 | chr13 | 42606685 | T | C | Missense_Mutation | disease causing | . |
| TNFSF12-TNFSF13 | chr17 | 7565788 | T | C | Intron | . | . |
| TNKS | chr8 | 9764724 | T | C | Silent | . | . |
| TNPO1 | chr5 | 72877326 | A | G | Silent | . | . |
| TNRC6A | chr16 | 24790200 | A | G | Missense_Mutation | disease causing | . |
| TNS1 | chr2 | 217817702 | A | G | Missense_Mutation | disease causing | . |
| TOMM70 | chr3 | 100375115 | T | A | Missense_Mutation | disease causing | . |
| TOP3B | chr22 | 21962867 | A | G | Missense_Mutation | disease causing | . |
| TPO | chr2 | 1423113 | T | C | Missense_Mutation | polymorphism | . |
| TPST2 | chr22 | 26540922 | A | G | Missense_Mutation | disease causing | . |
| TRAF6 | chr11 | 36489855 | T | C | Missense_Mutation | disease causing | . |
| TRAP1 | chr16 | 3672698 | A | G | Splice_Site | disease causing | . |
| TRG-AS1 | chr7 | 38349509 | T | C | Missense_Mutation | . | . |
| TRG-AS1 | chr7 | 38349509 | T | C | Missense_Mutation | . | . |
| TRIM6-TRIM34 | chr11 | 5634638 | T | C | Missense_Mutation | polymorphism | . |
| TRO | chrX | 54930456 | T | C | Silent | . | . |
| TRPA1 | chr8 | 72055808 | A | G | Silent | . | . |
| TRPC4 | chr13 | 37782954 | A | G | Splice_Site | disease causing | . |
| TRPC5 | chrX | 111776642 | A | G | Missense_Mutation | disease causing | . |
| TRPS1 | chr8 | 115414594 | T | C | Missense_Mutation | disease causing | . |
| TRPT1 | chr11 | 64224716 | A | G | Missense_Mutation | disease causing | . |
| TRPV3 | chr17 | 3532761 | A | G | Missense_Mutation | disease causing | . |
| TRPV6 | chr7 | 142873573 | A | G | Missense_Mutation | disease causing | . |
| TSHZ1 | chr18 | 75286339 | A | T | Missense_Mutation | disease causing | . |
| TSHZ2 | chr20 | 53254051 | A | G | Missense_Mutation | disease causing | . |
| TSPAN15 | chr10 | 69485148 | A | T | Missense_Mutation | disease causing | . |
| TSPYL1 | chr6 | 116278923 | T | C | Missense_Mutation | disease causing | . |
| TTC16 | chr9 | 127724131 | A | G | Missense_Mutation | disease causing | . |
| TTC17 | chr11 | 43405553 | A | G | Missense_Mutation | polymorphism | . |
| TTC21B | chr2 | 165890639 | A | T | Missense_Mutation | disease causing | . |
| TTC23 | chr15 | 99156130 | T | C | Intron | . | . |
| TTC23 | chr15 | 99199994 | T | C | Silent | . | . |
| TTK | chr6 | 80040282 | T | C | Splice_Site | disease causing | . |
| TTN | chr2 | 178528799 | T | C | Missense_Mutation | disease causing | . |
| TTN | chr2 | 178559711 | A | G | Silent | . | . |
| TTN | chr2 | 178565688 | T | C | Missense_Mutation | disease causing | . |
| TTN | chr2 | 178571455 | T | C | Missense_Mutation | polymorphism | . |
| TTN | chr2 | 178773583 | T | C | Silent | . | . |
| TTN | chr2 | 178775530 | A | G | Missense_Mutation | disease causing | . |
| TTN | chr2 | 178794920 | A | C | Splice_Site | disease causing | . |
| TTYH3 | chr7 | 2661668 | T | C | Missense_Mutation | disease causing | . |
| TUFT1 | chr1 | 151574286 | A | G | Missense_Mutation | disease causing | . |
| U2AF2 | chr19 | 55661191 | T | C | Splice_Site | disease causing | . |
| UBA2 | chr19 | 34438705 | A | G | Missense_Mutation | disease causing | . |
| UBE3A | chr15 | 25370678 | T | C | Missense_Mutation | disease causing | . |
| UBE3C | chr7 | 157207406 | T | C | Missense_Mutation | disease causing | . |
| UBR3 | chr2 | 170061082 | A | G | Missense_Mutation | polymorphism | . |
| UBR4 | chr1 | 19117218 | T | A | Splice_Region | . | . |
| UBR7 | chr14 | 93219343 | T | C | Silent | . | . |
| UGGT1 | chr2 | 128107939 | T | C | Splice_Region | . | . |
| UGT1A1 | chr2 | 233760486 | T | C | Missense_Mutation | polymorphism | . |
| UNC13B | chr9 | 35378384 | A | G | Missense_Mutation | disease causing | . |
| UNC79 | chr14 | 93597428 | A | G | Missense_Mutation | disease causing | . |
| UNC93B1 | chr11 | 67999291 | T | C | Missense_Mutation | disease causing | . |
| USF3 | chr3 | 113657766 | T | C | Missense_Mutation | polymorphism | . |
| USF3 | chr3 | 113659502 | A | G | Missense_Mutation | disease causing | . |
| USH2A | chr1 | 215900161 | T | C | Missense_Mutation | polymorphism | . |
| USH2A | chr1 | 216199769 | A | G | Silent | . | . |
| USP28 | chr11 | 113813933 | A | G | Silent | . | . |
| USP33 | chr1 | 77697262 | A | G | 3'UTR | . | . |
| USP45 | chr6 | 99446141 | T | C | Missense_Mutation | polymorphism | . |
| USPL1 | chr13 | 30631261 | A | G | Missense_Mutation | polymorphism | . |
| USPL1 | chr13 | 30659097 | T | C | Missense_Mutation | polymorphism | . |
| VIM | chr10 | 17234766 | A | G | Missense_Mutation | disease causing | . |
| VIT | chr2 | 36808743 | A | G | Missense_Mutation | disease causing | . |
| VOPP1-FKBP9P1 | chr7 | 55646094 | A | T | RNA | . | . |
| VPS13D | chr1 | 12276508 | T | C | Missense_Mutation | disease causing | . |
| VPS33A | chr12 | 122261325 | T | C | Missense_Mutation | disease causing | . |
| VPS41 | chr7 | 38756980 | T | C | Missense_Mutation | disease causing | . |
| VPS8 | chr3 | 184839724 | T | C | Silent | . | . |
| VWCE | chr11 | 61274516 | T | C | Missense_Mutation | disease causing | . |
| VWF | chr12 | 6121243 | A | G | Missense_Mutation | disease causing | . |
| WAPL | chr10 | 86500357 | A | G | Missense_Mutation | disease causing | . |
| WBP1L | chr10 | 102798014 | A | G | Missense_Mutation | polymorphism | . |
| WBSCR17 | chr7 | 71421051 | A | G | Missense_Mutation | disease causing | . |
| WDFY3 | chr4 | 84679096 | T | C | Missense_Mutation | polymorphism | . |
| WDR3 | chr1 | 117949825 | T | A | Missense_Mutation | disease causing | . |
| WDR35 | chr2 | 19936317 | T | C | Silent | . | . |
| WDR60 | chr7 | 158902646 | A | G | Intron | . | . |
| WFDC6 | chr20 | 45538042 | A | G | Silent | . | . |
| WIPI2 | chr7 | 5229708 | A | G | Missense_Mutation | disease causing | . |
| WISP1 | chr8 | 133220655 | T | C | Missense_Mutation | disease causing | . |
| WNK1 | chr12 | 885137 | A | G | Missense_Mutation | polymorphism | . |
| WRNIP1 | chr6 | 2785064 | T | C | Missense_Mutation | disease causing | . |
| WWC1 | chr5 | 168385364 | A | G | Missense_Mutation | disease causing | . |
| WWC2 | chr4 | 183253902 | A | G | Missense_Mutation | disease causing | . |
| XKR7 | chr20 | 31995070 | A | G | Missense_Mutation | disease causing | . |
| XRCC1 | chr19 | 43546602 | T | C | Silent | . | . |
| XXYLT1 | chr3 | 195069932 | T | C | Missense_Mutation | disease causing | . |
| YME1L1 | chr10 | 27147496 | A | G | Splice_Site | polymorphism | . |
| ZAP70 | chr2 | 97732944 | T | C | Missense_Mutation | disease causing | . |
| ZAP70 | chr2 | 97737790 | T | C | Missense_Mutation | disease causing | . |
| ZBBX | chr3 | 167240799 | A | G | Silent | . | . |
| ZBTB1 | chr14 | 64522745 | T | G | Missense_Mutation | polymorphism | . |
| ZBTB11 | chr3 | 101671235 | A | G | Missense_Mutation | disease causing | . |
| ZBTB40 | chr1 | 22490041 | T | C | Silent | . | . |
| ZC3H12D | chr6 | 149456855 | T | C | Missense_Mutation | disease causing | . |
| ZCCHC7 | chr9 | 37356836 | A | G | Splice_Region | . | . |
| ZDBF2 | chr2 | 206305407 | T | C | Silent | . | . |
| ZDBF2 | chr2 | 206309105 | T | C | Missense_Mutation | polymorphism | . |
| ZDHHC4 | chr7 | 6588746 | T | C | Missense_Mutation | disease causing | . |
| ZFAND6 | chr15 | 80137517 | A | G | Silent | . | . |
| ZFYVE26 | chr14 | 67783389 | T | C | Missense_Mutation | polymorphism | . |
| ZFYVE28 | chr4 | 2304556 | T | C | Missense_Mutation | polymorphism | . |
| ZFYVE28 | chr4 | 2305331 | A | G | Missense_Mutation | disease causing | . |
| ZHX1 | chr8 | 123253780 | A | G | Missense_Mutation | disease causing | . |
| ZKSCAN5 | chr7 | 99506165 | T | C | Missense_Mutation | disease causing | . |
| ZKSCAN7 | chr3 | 44571149 | T | C | Missense_Mutation | polymorphism | . |
| ZNF106 | chr15 | 42451516 | T | C | Silent | . | . |
| ZNF197 | chr3 | 44631136 | A | T | Silent | . | . |
| ZNF226 | chr19 | 44172170 | A | G | Missense_Mutation | disease causing | . |
| ZNF250 | chr8 | 144889865 | T | C | Intron | . | . |
| ZNF280D | chr15 | 56678689 | A | G | Silent | . | . |
| ZNF292 | chr6 | 87255710 | A | G | Missense_Mutation | disease causing | . |
| ZNF317 | chr19 | 9160291 | T | C | Missense_Mutation | polymorphism | . |
| ZNF334 | chr20 | 46502010 | A | G | Silent | . | . |
| ZNF366 | chr5 | 72460701 | A | T | Missense_Mutation | disease causing | . |
| ZNF404 | chr19 | 43873939 | T | C | Missense_Mutation | polymorphism | . |
| ZNF433 | chr19 | 12017901 | A | G | Missense_Mutation | polymorphism | . |
| ZNF480 | chr19 | 52322326 | T | C | Missense_Mutation | polymorphism | . |
| ZNF518B | chr4 | 10445264 | A | G | Silent | . | . |
| ZNF614 | chr19 | 52016142 | T | C | Missense_Mutation | polymorphism | . |
| ZNF638 | chr2 | 71406198 | T | C | Missense_Mutation | disease causing | . |
| ZNF644 | chr1 | 90939784 | A | G | Missense_Mutation | disease causing | . |
| ZNF644 | chr1 | 90940144 | T | C | Missense_Mutation | disease causing | . |
| ZNF7 | chr8 | 144842445 | T | C | Silent | . | . |
| ZNF701 | chr19 | 52583704 | T | C | 3'UTR | . | . |
| ZNF749 | chr19 | 57445024 | A | G | Missense_Mutation | polymorphism | . |
| ZNF75D | chrX | 135292340 | A | G | Missense_Mutation | polymorphism | . |
| ZNF783 | chr7 | 149266413 | T | C | Missense_Mutation | polymorphism | . |
| ZNF804A | chr2 | 184866454 | A | G | Missense_Mutation | disease causing | . |
| ZNF830 | chr17 | 34962784 | A | G | 3'UTR | . | . |
| ZNF845 | chr19 | 53352494 | T | C | Missense_Mutation | polymorphism | . |
| ZNF862 | chr7 | 149862393 | A | G | Missense_Mutation | polymorphism | . |
| ZRANB1 | chr10 | 124942805 | T | C | Silent | . | . |
| ZSCAN20 | chr1 | 33491449 | T | C | Silent | . | . |
| ZSCAN31 | chr6 | 28326628 | T | C | Silent | . | . |
| ZW10 | chr11 | 113768937 | A | G | Missense_Mutation | disease causing | . |
|  |  |  |  |  |  |  |  |
