# Supplementary material for: Helicobacter pylori base-excision restriction enzyme in stomach carcinogenesis
Source: PNAS Nexus. 2025 Aug 5;4(8):pgaf244. doi: 10.1093/pnasnexus/pgaf244 (PMC12366791; doi:10.1093/pnasnexus/pgaf244)
Supplement: pgaf244_Supplementary_Data [file pgaf244_supplementary_data.zip › PNASNEXUS-PNASNEXUS-2024-00952RR-s17.docx]

**Table S3. Enrichment analyses of genes with a GTAC mutation before filtering of cancer-related genes.**

**Category Term -log10(p) Genes**

GOTERM_BP_DIRECT

PTPRT, SPON2, CNTNAP1, PCDHGB4, TNC, HAPLN3, PTPRF, ADAMTSL1, CLDN23, STAB1, SIRPA, SVEP1, EMB, DGCR2, CDON, EPHA4, VWF, DSCAM, PCDHGA3, OMD, RPSA, PCDHGA9, ADAM2, ADAM18, MAG, OBSCN, MMRN1,

GO:0007155~cell adhesion 6.10 ADAM9, CLDN16, DSCAML1, DSC3, NLGN3, LAMA4, NEDD9, KLC1, TM9SF4, PCDH17, DPP4,

PDZD2, CLCA2, ABL2, PCDHA4, PCDHA8, PCDHA6, OPCML, NLGN4X, AMBP, MUC16, PCDHGC4, ADAM32, PCDHGA10, NPHS1, FAT1, PCDHB6, CNTN1, CNTN4, IGSF9B, PCDHB8, SIGLEC5

PTPRT, TENM3, PCDHGB4, PCDH17, TRO, PCDHA4, CDH26,

GO:0007156~homophilic cell adhesion

via plasma membrane adhesion molecules

5.37 ME2, EMB, PCDHA8, PCDHA6, DSCAM, PCDHGA3, PCDHGC4, PCDHGA9, PCDHGA10, SDK1, PCDHB6, FAT1,

FAT2, PLXNB2, CNTN4, OBSL1, DSCAML1, PCDHB8, DSC3

SLC12A2, GABRB2, OCA2, GABRA1, TTYH3, GABRA5,

GO:1902476~chloride transmembrane transport 4.76 CLCNKA, ANO9, SLC6A1, BEST2, CLCN3, ANO2, GABRR3,

ANO1, GLRA3, CLCA2, SLC17A6, SLC12A7, CLCA4 GRIA1, NLGN3, GABRB2, GRIA2, CHRNA9, SLC6A1, CACNA1E, GRM1, GABRR3, GLRA3, SV2C, PTCHD1, HRH2,

GO:0007268~chemical synaptic transmission 4.07 GRM6, SV2A, HRH4, PENK, SPG11, SLC12A7, UNC13B, GABRA1, NLGN4X, HOMER1, GABRA5, GAD1, CEL, HTR5A, OR5T2, PCDHB6, EXOC4

SLC12A2, GABRB2, GABRA1, TTYH3, GABRA5, SLC4A10,

GO:0006821~chloride transport 3.72 CLCNKA, ANO9, BEST2, CLCN3, ANO1, GLRA3, CLCA2, CLCA4

DNAH3, DYNC1H1, DNAH11, DNAH17, DNAH8, DNAH5,

GO:0007018~microtubule-based movement 3.69 KIF24, DNAH9, KLC1, KIF6, CENPE, KIF18A, KIF9, KIF5B, KIF3C

DOCK5, NCKAP1, WWC1, USP33, WWC2, ASAP3, RHOBTB3, NEDD9, ADAMTS12, PTPRF, GPC1, SIRPA, GPC2,

GO:0016477~cell migration 3.49 IL12A, NTNG1, ZRANB1, ATRNL1, OR51E2, USP45, PTK6, USH2A, CCDC88A, TIAM1, TAOK2, FAT1, ADAM9, CORO7, INSM1, DOCK2, FGFR4

GO:0060687~regulation of branching involved in prostate gland morphogenesis

2.88 BMP4, SFRP1, HOXD13, BMP7

KEGG_PATHWAY

GO:0060078~regulation of postsynaptic membrane potential 2.79 GRIA1, GABRB2, GABRA1, GRIA2, GABRA5, GRID1, KCNA2,

GRM1

RIF1, DCUN1D5, PRKDC, STXBP4, OGG1, ZBTB1, ZBTB40,

GO:0006974~cellularresponse to DNAdamage stimulus 2.69 RAD51AP1, BCLAF1, HERC2, RBBP6, BARD1, POLQ, SHPRH,

CHD1L, SIRT7, BAZ1B, USP28, FBXO31, CUL4A, SETX,

FAM111A, TRAF6, DDIT3, TAOK2, BCL3, ATM, TP53, RAD18

GRIA1, GABRB2, GRIA2, GABRA1, TTYH3, GRID1, GABRA5,

GO:0034220~ion transmembrane transport 2.60 CHRNA9, ANO9, TMC7, NALCN, ANO2, GABRR3, ANO1, GLRA3, CLCA2, CLCA4

GO:0042220~response to cocaine 2.56 GRIA1, EFTUD2, TIAM1, DNMT3A, UBE3A, OPRM1, SLC6A1, PITX3

DNAH3, MYH7B, DYNC1H1, DNAH11, DNAH17, DNAH8,

hsa04814:Motorproteins 2.52 DNAH5, KIF24, DNAH9, MYO7A, KLC1, MYO9A, KIF6, MYO19, ACTB, CENPE, KIF18A, KIF9, MYO15A, KIF5B,

MYO5C, KIF3C
