# Supplementary material for: Helicobacter pylori base-excision restriction enzyme in stomach carcinogenesis
Source: PNAS Nexus. 2025 Aug 5;4(8):pgaf244. doi: 10.1093/pnasnexus/pgaf244 (PMC12366791; doi:10.1093/pnasnexus/pgaf244)
Supplement: pgaf244_Supplementary_Data [file pgaf244_supplementary_data.zip › PNASNEXUS-PNASNEXUS-2024-00952RR-s18.docx]

**Table S4. Enrichment analyses of genes with a GTAC mutation after filtering of cancer-related genes by COSMIC.** Category Term -log10(p) Genes

# GOTERM_BP_DIRECT

GO:0045944~positive regulation of transcription

# BCL11A, HOXD13, PIK3R1, BCLAF1, ELF3, KAT6B,

from RNA polymerase II promoter 7.37 THRAP3, DDIT3, KAT6A, ASXL2, BCL3, AKT1, EP300, CAMTA1, ATM, HRAS, ZNF521, TP53, FGFR2, BCL9L

GO:0018108~peptidyl-tyrosine phosphorylation 5.69 CSF1R, FLT3, ABL2, PTPN6, FGFR4, FGFR2 GO:0048568~embryonic organ development 5.13 PTCH1, PALB2, POLE, TP53, FGFR2

GO:0007275~multicellular organism development 4.88 CSF1R, CUX1, FLT3, SUFU, HOXD13, FGFR4, FGFR2

GO:0042981~regulation of apoptotic process 4.55 HIP1, FLT3, BCL3, ABL2, AKT1, PTPN6, ATM, TP53 GO:0006606~protein importinto nucleus 4.40 NUP214, BCL3, AKT1, NUP98, PIK3R1, TP53

GO:0045892~negative regulation of transcription, DNA-templated

# 4.32 NCOR2, BCLAF1, ELF3, KAT6B, DDIT3, KAT6A, BCL3, DNMT3A, MDM4, BCOR, TP53

GO:0046777~protein autophosphorylation 4.31 CSF1R, FLT3, AKT1, ATM, PTK6, FGFR4, FGFR2

GO:0071300~cellular response to retinoic acid 3.88 TNC, ABL2, ATM, PTK6, FGFR2

GO:0007169~transmembrane receptorprotein tyrosine kinase signaling pathway

# 3.87 PTPRT, CSF1R, FLT3, PTK6, FGFR4, FGFR2

GO:0009887~animal organ morphogenesis 3.80 PTCH1, ASXL2, EP300, HRAS, PALB2, FGFR2

GO:0051726~regulation of cell cycle 3.39 BARD1, NUP214, DDIT3, MDM4, ATM, HRAS, TP53

GO:1901796~regulation of signal transduction byp53 class mediator

# 3.33 KAT6A, EP300, AKT1, ATM

GO:0001756~somitogenesis 3.30 EP300, ATM, PALB2, TP53

GO:0006974~cellular response to DNA damage stimulus 3.15 BARD1, POLQ, BCLAF1, DDIT3, BCL3, ATM, TP53

GO:0008284~positive regulation of cell proliferation 3.13 CSF1R, FLT3, TNC, AKT1, PTPN6, FGFR4, HRAS,

# FGFR2, CRLF2

GO:0000122~negative regulation of transcription from RNA polymerase II promoter

# 3.02 NCOR2, CUX1, BCL11A, DDIT3, SUFU, PTCH1, DNMT3A, EP300, MDM4, BCOR, TP53, FGFR2

GO:0090398~cellular senescence 2.94 KAT6A, ATM, HRAS, TP53

GO:0045893~positive regulation of transcription, DNA-templated

# 2.94 KAT6B, THRAP3, DDIT3, KAT6A, PTCH1, BCL3, EP300, AKT1, NUP98, TP53

GO:0071456~cellular response to hypoxia 2.86 DNMT3A, MDM4, MALAT1, TP53, FGFR2

GO:0030330~DNA damage response,

signal transduction byp53 class mediator

# 2.78 BCL3, MDM4, TP53

KEGG_PATHWAY

GO:0033674~positive regulation of kinase activity 2.65 CSF1R, FLT3, FGFR4, FGFR2 GO:0010506~regulation of autophagy 2.60 DDIT3, EP300, ABL2, ATM GO:0019221~cytokine-mediated signaling pathway 2.58 CSF1R, FLT3, AKT1, PTPN6, CRLF2

hsa05230:Central carbon metabolism in cancer 6.09 FLT3, AKT3, AKT1, PIK3R1, HRAS, TP53, FGFR2

hsa05200:Pathways in cancer 5.41 CSF1R, FLT3, PTCH1, PIK3R1, SUFU, AKT3, CCDC6,

# AKT1, EP300, FGFR4, HRAS, TP53, FGFR2

hsa05215:Prostate cancer 5.25 AKT3, EP300, AKT1, PIK3R1, HRAS, TP53, FGFR2

hsa04014:Ras signaling pathway 4.92 CSF1R, FLT3, AKT3, ABL2, AKT1, PIK3R1, FGFR4,

# HRAS, FGFR2

hsa05221:Acute myeloid leukemia 4.84 CSF1R, FLT3, AKT3, AKT1, PIK3R1, HRAS

hsa05223:Non-small cell lung cancer 4.68 KIF5B, AKT3, AKT1, PIK3R1, HRAS, TP53

hsa04151:PI3K-Akt signaling pathway 4.45 CSF1R, FLT3, AKT3, TNC, AKT1, PIK3R1, FGFR4,

# HRAS, TP53, FGFR2

hsa04210:Apoptosis 4.41 DDIT3, AKT3, AKT1, ATM, PIK3R1, HRAS, TP53

hsa04550:Signaling pathways regulating pluripotency of stem cells

# 4.29 KAT6A, AKT3, AKT1, PIK3R1, FGFR4, HRAS, FGFR2

hsa04010:MAPK signaling pathway 4.16 CSF1R, FLT3, DDIT3, AKT3, AKT1, FGFR4, HRAS,

# TP53, FGFR2

hsa05166:Human T-cell leukemia virus 1 infection 4.14 AKT3, EP300, BUB1B, AKT1, ATM, PIK3R1, HRAS, TP53

hsa04630:JAK-STAT signaling pathway 3.93 AKT3, EP300, AKT1, PTPN6, PIK3R1, HRAS, CRLF2

hsa04625:C-type lectin receptorsignaling pathway 3.92 CYLD, AKT3, BCL3, AKT1, PIK3R1, HRAS hsa05213:Endometrial cancer 3.81 AKT3, AKT1, PIK3R1, HRAS, TP53

hsa04919:Thyroid hormone signaling pathway 3.61 AKT3, EP300, AKT1, PIK3R1, HRAS, TP53

hsa05211:Renal cell carcinoma 3.52 AKT3, EP300, AKT1, PIK3R1, HRAS

hsa05218:Melanoma 3.45 AKT3, AKT1, PIK3R1, HRAS, TP53

hsa04068:FoxO signaling pathway 3.45 AKT3, EP300, AKT1, ATM, PIK3R1, HRAS

hsa05205:Proteoglycans in cancer 3.43 PTCH1, AKT3, AKT1, PTPN6, PIK3R1, HRAS, TP53

hsa01524:Platinum drug resistance 3.43 AKT3, AKT1, ATM, PIK3R1, TP53

hsa04380:Osteoclast differentiation 3.39 CYLD, CSF1R, AKT3, SIRPA, AKT1, PIK3R1

hsa05214:Glioma 3.38 AKT3, AKT1, PIK3R1, HRAS, TP53

hsa04015:Rap1 signaling pathway 3.37 CSF1R, AKT3, AKT1, PIK3R1, FGFR4, HRAS, FGFR2

hsa05220:Chronic myeloid leukemia 3.36 AKT3, AKT1, PIK3R1, HRAS, TP53 hsa01521:EGFR tyrosine kinase inhibitor resistance 3.30 AKT3, AKT1, PIK3R1, HRAS, FGFR2

hsa05206:MicroRNAs in cancer 3.25 DNMT3A, TNC, EP300, MDM4, ATM, PIK3R1, HRAS,

# TP53

hsa04662:B cell receptorsignaling pathway 3.19 AKT3, AKT1, PTPN6, PIK3R1, HRAS

hsa05226:Gastric cancer 3.19 AKT3, AKT1, PIK3R1, HRAS, TP53, FGFR2

hsa04012:ErbB signaling pathway 3.17 AKT3, ABL2, AKT1, PIK3R1, HRAS

hsa05210:Colorectal cancer 3.16 AKT3, AKT1, PIK3R1, HRAS, TP53

hsa05235:PD-L1 expression and PD-1 checkpoint pathway in cancer

# 3.10 AKT3, AKT1, PTPN6, PIK3R1, HRAS

hsa04211:Longevity regulating pathway 3.10 AKT3, AKT1, PIK3R1, HRAS, TP53

hsa04218:Cellular senescence 3.10 AKT3, AKT1, ATM, PIK3R1, HRAS, TP53

hsa05165:Human papillomavirus infection 3.08 AKT3, TNC, EP300, AKT1, ATM, PIK3R1, HRAS, TP53

hsa05161:Hepatitis B 3.02 AKT3, EP300, AKT1, PIK3R1, HRAS, TP53

hsa01522:Endocrine resistance 2.94 AKT3, AKT1, PIK3R1, HRAS, TP53

hsa04668:TNF signaling pathway 2.70 CYLD, AKT3, BCL3, AKT1, PIK3R1

hsa05167:Kaposi sarcoma-associated herpesvirus infection 2.68 AKT3, EP300, AKT1, PIK3R1, HRAS, TP53 hsa04722:Neurotrophin signaling pathway 2.63 AKT3, AKT1, PIK3R1, HRAS, TP53

hsa04935:Growth hormone synthesis, secretion and action 2.62 AKT3, EP300, AKT1, PIK3R1, HRAS hsa04071:Sphingolipid signaling pathway 2.61 AKT3, AKT1, PIK3R1, HRAS, TP53

hsa04660:T cell receptorsignaling pathway 2.61 AKT3, AKT1, PTPN6, PIK3R1, HRAS

hsa04370:VEGF signaling pathway 2.57 AKT3, AKT1, PIK3R1, HRAS hsa04213:Longevity regulating pathway - multiple species 2.53 AKT3, AKT1, PIK3R1, HRAS
