# Supplementary material for: Helicobacter pylori base-excision restriction enzyme in stomach carcinogenesis
Source: PNAS Nexus. 2025 Aug 5;4(8):pgaf244. doi: 10.1093/pnasnexus/pgaf244 (PMC12366791; doi:10.1093/pnasnexus/pgaf244)
Supplement: pgaf244_Supplementary_Data [file pgaf244_supplementary_data.zip › PNASNEXUS-PNASNEXUS-2024-00952RR-s19.docx]

**Table S5. Number of unique mutations of 4-mers on *H. pylori* genome during infection.**

| Motif ＼linage | 479 | 476 | 169 | 25 |
| --- | --- | --- | --- | --- |
| AA***A***A | 283 | 385 | 49 | 50 |
| AA***A***C | 176 | 345 | 43 | 51 |
| AA***A***G | 195 | 319 | 39 | 42 |
| AA***A***T | 155 | 235 | 23 | 43 |
| AA***C***A | 212 | 285 | 38 | 77 |
| AA***C***C | 159 | 199 | 23 | 52 |
| AA***C***G | 168 | 221 | 30 | 64 |
| AA***C***T | 144 | 217 | 29 | 44 |
| AA***G***A | 232 | 313 | 37 | 82 |
| AA***G***C | 254 | 319 | 38 | 83 |
| AA***G***G | 186 | 302 | 28 | 63 |
| AA***G***T | 128 | 188 | 22 | 41 |
| AA***T***A | 204 | 307 | 25 | 33 |
| AA***T***C | 117 | 212 | 33 | 22 |
| AA***T***G | 152 | 242 | 27 | 32 |
| AA***T***T | 173 | 274 | 21 | 41 |
| AC***A***A | 98 | 163 | 12 | 24 |
| AC***A***C | 55 | 98 | 12 | 16 |
| AC***A***G | 52 | 92 | 4 | 19 |
| AC***A***T | 43 | 74 | 14 | 14 |
| AC***C***A | 157 | 183 | 21 | 52 |
| AC***C***C | 65 | 92 | 11 | 32 |
| AC***C***G | 75 | 100 | 10 | 24 |
| AC***C***T | 87 | 133 | 17 | 17 |
| AC***G***A | 91 | 135 | 10 | 28 |
| AC***G***C | 106 | 135 | 20 | 35 |
| AC***G***G | 65 | 72 | 8 | 18 |
| AC***G***T | 35 | 55 | 6 | 17 |
| AC***T***A | 109 | 154 | 10 | 26 |
| AC***T***C | 60 | 88 | 12 | 14 |
| AC***T***G | 41 | 81 | 4 | 8 |
| AC***T***T | 77 | 124 | 12 | 11 |
| AG***A***A | 77 | 128 | 16 | 28 |
| AG***A***C | 41 | 50 | 5 | 10 |
| AG***A***G | 94 | 121 | 14 | 19 |
| AG***A***T | 59 | 98 | 15 | 15 |
| AG***C***A | 110 | 164 | 16 | 33 |
| AG***C***C | 71 | 107 | 11 | 28 |
| AG***C***G | 266 | 342 | 35 | 104 |
| AG***C***T | 136 | 178 | 28 | 48 |
| AG***G***A | 72 | 82 | 15 | 21 |
| AG***G***C | 62 | 71 | 11 | 28 |
| AG***G***G | 84 | 118 | 8 | 32 |
| AG***G***T | 72 | 94 | 15 | 20 |
| AG***T***A | 82 | 98 | 15 | 19 |
| AG***T***C | 63 | 64 | 7 | 8 |
| AG***T***G | 157 | 203 | 40 | 41 |
| AG***T***T | 107 | 184 | 15 | 24 |
| AT***A***A | 145 | 243 | 19 | 41 |
| AT***A***C | 63 | 113 | 13 | 17 |
| AT***A***G | 99 | 157 | 20 | 25 |
| AT***A***T | 97 | 159 | 13 | 20 |
| AT***C***A | 179 | 284 | 28 | 68 |
| AT***C***C | 90 | 135 | 11 | 29 |
| AT***C***G | 137 | 156 | 24 | 40 |
| AT***C***T | 196 | 231 | 34 | 54 |
| AT***G***A | 148 | 196 | 28 | 40 |
| AT***G***C | 95 | 136 | 21 | 35 |
| AT***G***G | 123 | 162 | 23 | 36 |
| AT***G***T | 81 | 133 | 15 | 29 |
| AT***T***A | 161 | 282 | 20 | 39 |
| AT***T***C | 83 | 142 | 13 | 23 |
| AT***T***G | 127 | 173 | 26 | 31 |
| AT***T***T | 190 | 301 | 40 | 40 |
| CA***A***A | 100 | 150 | 22 | 29 |
| CA***A***C | 63 | 107 | 9 | 15 |
| CA***A***G | 76 | 122 | 12 | 20 |
| CA***A***T | 75 | 105 | 8 | 16 |
| CA***C***A | 62 | 112 | 11 | 29 |
| CA***C***C | 36 | 62 | 13 | 19 |
| CA***C***G | 39 | 57 | 9 | 15 |
| CA***C***T | 72 | 100 | 8 | 22 |
| CA***G***A | 62 | 98 | 11 | 20 |
| CA***G***C | 96 | 115 | 11 | 34 |
| CA***G***G | 71 | 92 | 5 | 17 |
| CA***G***T | 53 | 80 | 6 | 18 |
| CA***T***A | 43 | 81 | 5 | 10 |
| CA***T***C | 36 | 33 | 4 | 10 |
| CA***T***G | 53 | 50 | 13 | 6 |
| CA***T***T | 74 | 107 | 6 | 14 |
| CC***A***A | 74 | 94 | 12 | 25 |
| CC***A***C | 72 | 99 | 12 | 19 |
| CC***A***G | 44 | 69 | 6 | 11 |
| CC***A***T | 81 | 113 | 14 | 19 |
| CC***C***A | 145 | 184 | 24 | 52 |
| CC***C***C | 55 | 104 | 11 | 25 |
| CC***C***G | 42 | 68 | 5 | 18 |
| CC***C***T | 114 | 189 | 12 | 51 |
| CC***G***A | 47 | 57 | 7 | 11 |
| CC***G***C | 86 | 96 | 10 | 24 |
| CC***G***G | 50 | 77 | 9 | 17 |
| CC***G***T | 58 | 71 | 5 | 20 |
| CC***T***A | 127 | 160 | 25 | 22 |
| CC***T***C | 42 | 68 | 12 | 11 |
| CC***T***G | 42 | 65 | 5 | 13 |
| CC***T***T | 98 | 161 | 20 | 21 |
| CG***A***A | 25 | 31 | 3 | 6 |
| CG***A***C | 18 | 28 | 1 | 3 |
| CG***A***G | 14 | 29 | 6 | 3 |
| CG***A***T | 32 | 63 | 8 | 18 |
| CG***C***A | 45 | 76 | 12 | 16 |
| CG***C***C | 39 | 49 | 9 | 18 |
| CG***C***G | 66 | 103 | 10 | 36 |
| CG***C***T | 132 | 162 | 16 | 43 |
| CG***G***A | 12 | 27 | 3 | 4 |
| CG***G***C | 24 | 50 | 2 | 16 |
| CG***G***G | 12 | 29 | 1 | 9 |
| CG***G***T | 38 | 50 | 6 | 15 |
| CG***T***A | 27 | 50 | 1 | 5 |
| CG***T***C | 18 | 19 | 2 | 5 |
| CG***T***G | 61 | 63 | 13 | 7 |
| CG***T***T | 85 | 119 | 19 | 19 |
| CT***A***A | 95 | 141 | 10 | 35 |
| CT***A***C | 48 | 62 | 3 | 9 |
| CT***A***G | 71 | 113 | 12 | 16 |
| CT***A***T | 69 | 110 | 7 | 20 |
| CT***C***A | 117 | 154 | 22 | 32 |
| CT***C***C | 39 | 53 | 14 | 15 |
| CT***C***G | 36 | 71 | 7 | 10 |
| CT***C***T | 112 | 151 | 17 | 46 |
| CT***G***A | 66 | 118 | 9 | 22 |
| CT***G***C | 49 | 57 | 6 | 17 |
| CT***G***G | 65 | 95 | 10 | 27 |
| CT***G***T | 71 | 110 | 9 | 34 |
| CT***T***A | 94 | 130 | 17 | 19 |
| CT***T***C | 38 | 49 | 6 | 16 |
| CT***T***G | 49 | 76 | 5 | 19 |
| CT***T***T | 139 | 217 | 27 | 36 |
| GA***A***A | 113 | 180 | 22 | 23 |
| GA***A***C | 71 | 104 | 15 | 17 |
| GA***A***G | 63 | 93 | 8 | 10 |
| GA***A***T | 88 | 135 | 15 | 20 |
| GA***C***A | 91 | 153 | 18 | 43 |
| GA***C***C | 18 | 41 | 4 | 7 |
| GA***C***G | 52 | 91 | 8 | 19 |
| GA***C***T | 76 | 104 | 9 | 24 |
| GA***G***A | 117 | 159 | 28 | 40 |
| GA***G***C | 122 | 151 | 14 | 50 |
| GA***G***G | 72 | 96 | 14 | 23 |
| GA***G***T | 81 | 123 | 15 | 28 |
| GA***T***A | 96 | 148 | 10 | 23 |
| GA***T***C | 63 | 68 | 5 | 21 |
| GA***T***G | 81 | 101 | 19 | 20 |
| GA***T***T | 101 | 122 | 17 | 18 |
| GC***A***A | 101 | 135 | 21 | 30 |
| GC***A***C | 199 | 259 | 55 | 55 |
| GC***A***G | 65 | 86 | 12 | 11 |
| GC***A***T | 108 | 159 | 21 | 16 |
| GC***C***A | 155 | 218 | 23 | 46 |
| GC***C***C | 77 | 117 | 8 | 28 |
| GC***C***G | 92 | 102 | 11 | 25 |
| GC***C***T | 135 | 177 | 19 | 38 |
| GC***G***A | 126 | 166 | 30 | 35 |
| GC***G***C | 327 | 402 | 54 | 155 |
| GC***G***G | 96 | 106 | 12 | 30 |
| GC***G***T | 129 | 174 | 27 | 51 |
| GC***T***A | 126 | 202 | 23 | 24 |
| GC***T***C | 71 | 110 | 13 | 18 |
| GC***T***G | 66 | 112 | 11 | 22 |
| GC***T***T | 140 | 225 | 20 | 35 |
| GG***A***A | 50 | 90 | 4 | 18 |
| GG***A***C | 36 | 38 | 6 | 5 |
| GG***A***G | 68 | 122 | 15 | 12 |
| GG***A***T | 80 | 118 | 13 | 19 |
| GG***C***A | 86 | 131 | 23 | 35 |
| GG***C***C | 47 | 55 | 7 | 21 |
| GG***C***G | 137 | 155 | 34 | 44 |
| GG***C***T | 155 | 186 | 17 | 62 |
| GG***G***A | 57 | 100 | 11 | 22 |
| GG***G***C | 51 | 76 | 10 | 20 |
| GG***G***G | 89 | 135 | 20 | 41 |
| GG***G***T | 69 | 118 | 17 | 36 |
| GG***T***A | 82 | 113 | 8 | 13 |
| GG***T***C | 28 | 44 | 4 | 8 |
| GG***T***G | 87 | 135 | 22 | 33 |
| GG***T***T | 78 | 200 | 22 | 32 |
| GT***A***A | 66 | 99 | 8 | 15 |
| GT***A***C | 12 | 12 | 2 | 3 |
| GT***A***G | 66 | 115 | 16 | 19 |
| GT***A***T | 51 | 77 | 6 | 8 |
| GT***C***A | 55 | 107 | 9 | 27 |
| GT***C***C | 21 | 31 | 3 | 8 |
| GT***C***G | 36 | 60 | 12 | 12 |
| GT***C***T | 64 | 107 | 11 | 30 |
| GT***G***A | 87 | 111 | 8 | 28 |
| GT***G***C | 55 | 85 | 12 | 19 |
| GT***G***G | 105 | 177 | 15 | 35 |
| GT***G***T | 71 | 106 | 12 | 29 |
| GT***T***A | 77 | 127 | 11 | 33 |
| GT***T***C | 25 | 45 | 1 | 5 |
| GT***T***G | 54 | 77 | 4 | 10 |
| GT***T***T | 107 | 139 | 15 | 24 |
| TA***A***A | 161 | 274 | 27 | 53 |
| TA***A***C | 88 | 151 | 14 | 20 |
| TA***A***G | 72 | 119 | 11 | 19 |
| TA***A***T | 112 | 170 | 18 | 25 |
| TA***C***A | 76 | 125 | 9 | 20 |
| TA***C***C | 57 | 73 | 10 | 24 |
| TA***C***G | 47 | 68 | 7 | 27 |
| TA***C***T | 72 | 103 | 10 | 33 |
| TA***G***A | 109 | 168 | 22 | 43 |
| TA***G***C | 117 | 176 | 18 | 39 |
| TA***G***G | 95 | 140 | 15 | 33 |
| TA***G***T | 102 | 134 | 13 | 35 |
| TA***T***A | 89 | 115 | 8 | 19 |
| TA***T***C | 49 | 89 | 10 | 16 |
| TA***T***G | 53 | 86 | 11 | 13 |
| TA***T***T | 84 | 142 | 20 | 30 |
| TC***A***A | 66 | 92 | 6 | 12 |
| TC***A***C | 59 | 91 | 14 | 16 |
| TC***A***G | 47 | 78 | 8 | 10 |
| TC***A***T | 109 | 136 | 21 | 21 |
| TC***C***A | 142 | 204 | 28 | 43 |
| TC***C***C | 45 | 62 | 5 | 19 |
| TC***C***G | 52 | 54 | 10 | 13 |
| TC***C***T | 89 | 131 | 14 | 30 |
| TC***G***A | 16 | 28 | 1 | 9 |
| TC***G***C | 75 | 99 | 21 | 27 |
| TC***G***G | 50 | 73 | 7 | 15 |
| TC***G***T | 85 | 138 | 16 | 37 |
| TC***T***A | 108 | 197 | 27 | 26 |
| TC***T***C | 50 | 64 | 7 | 4 |
| TC***T***G | 59 | 68 | 10 | 8 |
| TC***T***T | 89 | 147 | 18 | 22 |
| TG***A***A | 78 | 123 | 8 | 15 |
| TG***A***C | 42 | 60 | 8 | 9 |
| TG***A***G | 47 | 60 | 9 | 13 |
| TG***A***T | 95 | 128 | 16 | 17 |
| TG***C***A | 79 | 105 | 10 | 42 |
| TG***C***C | 42 | 55 | 8 | 18 |
| TG***C***G | 126 | 137 | 26 | 57 |
| TG***C***T | 164 | 233 | 20 | 52 |
| TG***G***A | 77 | 103 | 13 | 27 |
| TG***G***C | 61 | 69 | 12 | 21 |
| TG***G***G | 58 | 91 | 6 | 22 |
| TG***G***T | 93 | 115 | 12 | 31 |
| TG***T***A | 51 | 83 | 11 | 8 |
| TG***T***C | 28 | 49 | 7 | 6 |
| TG***T***G | 81 | 144 | 18 | 25 |
| TG***T***T | 125 | 203 | 25 | 29 |
| TT***A***A | 241 | 386 | 37 | 48 |
| TT***A***C | 118 | 157 | 17 | 16 |
| TT***A***G | 232 | 327 | 37 | 38 |
| TT***A***T | 213 | 300 | 22 | 37 |
| TT***C***A | 209 | 272 | 29 | 58 |
| TT***C***C | 67 | 95 | 14 | 21 |
| TT***C***G | 74 | 97 | 5 | 21 |
| TT***C***T | 146 | 251 | 36 | 55 |
| TT***G***A | 257 | 389 | 43 | 95 |
| TT***G***C | 123 | 200 | 22 | 55 |
| TT***G***G | 308 | 359 | 48 | 95 |
| TT***G***T | 217 | 326 | 40 | 76 |
| TT***T***A | 214 | 329 | 26 | 48 |
| TT***T***C | 82 | 136 | 11 | 23 |
| TT***T***G | 109 | 157 | 16 | 31 |
| TT***T***T | 221 | 333 | 38 | 55 |

The number of unique mutations are shown. Substitution at the 3rd letter was measured. CG (underlined) shows high mutation at G because of C deamination.
