# Supplementary material for: Helicobacter pylori base-excision restriction enzyme in stomach carcinogenesis
Source: PNAS Nexus. 2025 Aug 5;4(8):pgaf244. doi: 10.1093/pnasnexus/pgaf244 (PMC12366791; doi:10.1093/pnasnexus/pgaf244)
Supplement: pgaf244_Supplementary_Data [file pgaf244_supplementary_data.zip › PNASNEXUS-PNASNEXUS-2024-00952RR-s22.docx]

| Table S8. Bacteria and restriction enzymes predicted to cause cancer. | | | | |  |
| --- | --- | --- | --- | --- | --- |
| study | genus | | motif (Relative Ratio in each cancer study,  *Compositional bias in genus*) | | ref |
| STAD (Stomach adenocarcinoma) | | | | | |
|  | Fusobacterium | | GTA***A***G (2.7, *1.05*) | | [26] |
|  | Helicobacter | | CG***C***G (2.0, *0.45*), CGC***G*** (1.8, *0.45*), GAC***T*** (1.8, *1.28*),  GT***A***C (2.3, *0.10*), AC***G***CA (2.5, *0.86*), ACGG***T*** (2.3, *0.38*),  CTA***A***G (2.5, *0.57*), GAC***G***C (2.4, *0.92*), GCG***T***A (2.4, *0.87*),  GCGT***A*** (2.5, *0.87*), CCGAT***A*** (3.6, *1.11*), CCG***T***AC (3.7, *0.97*),  CGGGC***T***T (8.0, *1.12*), CGCG***A***AT (45.2, *1.14*) | | [26]^,^ [27, 28] |
|  | Streptococcus | | C***A***C (1.3, *0.84*), G***A***C (1.5, *1.07*), AC***T***TC (3.3, *1.07*),  GAC***G***C (2.4, *0.97*) | | [26] |
| Other cancers | | |  | |  |
| BLCA (Bladder urothelial carcinoma) | | | | |  |
|  | Escherichia | | AT***C*** (1.9, *1.18*), AT***G*** (1.4, *0.87*), GA***C*** (1.1, *0.91*),  CA***G***AG (4.7, *0.87*), CCT***C***AGG (6.6, *0.81*) | | [29] |
| BRCA (Breast invasive carcinoma) | | | | | |
|  | Escherichia | | AT***C*** (1.5, *1.18*), CA***C*** (1.0, *0.88*), GA***C*** (1.1, *0.91*), CA***G***AG (2.6, *0.87*) | | [29] |
|  | Pseudopropionibacterium | | CAC***A***TC (3.4, *1.03*) | | [29] |
|  | Staphylococcus | | CCT***C*** (2.3, *1.20*), CA***G***AG (2.6, *0.90*), GCT***C***A (2.7, *1.06*),  GCT***G***A (2.8, *1.03*) | | [29] |
|  | Streptococcus | | AT***C*** (1.5, *1.07*), CA***C*** (1.0, *0.84*), GA***C*** (1.1, *1.07*), CA***G***AG (2.6, *0.88*) | | [29] |
| CHOL (Cholangiocarcinoma) | | |  | |  |
|  | Helicobacter | | TCG***A*** (2.3, *0.12*), ACGA***A*** (3.6, *0.98*), CTA***A***G (5.0, *0.57*), GATCG***A***T (20.4, *1.03*) | | [28] |
| COAD (Colon adenocarcinoma) | | |  | |  |
|  | Actinomyces | | AAT***T*** (1.6, *1.29*) | | [27] |
|  | Campylobacter | | GC***G***C (1.8, *0.47*), AC***G***AA (2.0, *1.00*), ATC***G***C (2.1, *1.01*),  GC***G***AA (1.9, *1.02*), AAAT***T***T (2.6, *0.98*), TATTC***G*** (2.4, *1.01*) | | [28] |
|  | Enterococcus | | CATC***G*** (1.8, *0.91*) | | [28] |
|  | Escherichia | | G***A***C (1.3, *0.91*) | | [27]^,^ [28]^,^ [29] |
|  | Fusobacterium | | GC***G***C (1.8, *0.47*) | | [26]^,^ [27]^,^ [28]^,^ [29]^,^ [30] |
|  | Prevotella | | GAC***G***C (2.2, 0.86) | | [30] |
|  | Streptococcus | | G***A***C (1.3, *1.07*), ATG***C***G (2.2, *1.20*), CATC***G*** (1.8, *0.89*),  GAC***G***C (2.2, *0.97*), GTT***C***G (2.1, *1.15*), TCGC***G*** (1.8, *0.83*) | | [28] |
| ESCA (Esophageal carcinoma) | | |  | |  |
|  | Campylobacter | | GA***A***GA (2.8, *1.16*), CCTA***A*** (2.9, *0.90*) | | [28]^,^ [29] |
|  | Escherichia | | CCA***A***G (3.6, *0.71*), GGTA***A***G (6.2, *0.93*), CAAC***T***T (5.3, *1.14*) | | [28]^,^ [29] |
|  | Fusobacterium | | GTA***A***G (4.8, *1.05*) | | [28]^,^ [31] |
|  | Neisseria | | GA***A***GA (2.8, *1.15*) | | [31] |
|  | Prevotella | | ATCGA***T*** (4.7, *0.74*) | | [31] |
|  | Pseudomonas | | GAAC***T***T (6.1, *0.99*), ATCGA***T*** (4.7, *0.74*) | | [31] |
|  | Streptococcus | | AC***T***TC (5.1, *1.07*) | | [31] |
| HNSC (Head and Neck squamous cell carcinoma) | | | |  |  |
|  | Bacteroides | AT***G***AG (2.4, *1.14*) | | | [32] |
|  | Capnocytophaga | CCT***C***AG (2.8, *1.18*) | | | [32] |
|  | Enterococcus | GCT***G***A (2.6, *1.09*) | | | [32] |
|  | Haemophilus | CCT***G***A (2.4, *1.06*), GTT***G***A (2.3, *0.99*) | | | [32] |
|  | Lactobacillus | CCT***C*** (2.2, *1.01*), CA***G***AG (2.2, *0.88*), GCCT***C*** (2.1, *0.91*) | | | [32] |
|  | Lactococcus | GGCT***G***A (2.7, *0.97*) | | | [32] |
|  | Prevotella | CA***G***AG (2.2, *0.95*), CCCT***C*** (2.3, *1.07*), CCT***C***AG (2.8, *0.98*) | | | [32] |
|  | Rothia | CGCGA***G*** (2.5, *0.98*) | | | [32] |
|  | Streptococcus | AT***C*** (1.4, *1.07*), AT***G*** (1.3, *0.92*), GA***C*** (1.1, *1.07*), CA***G***AG (2.2, *0.88*) | | | [32] |
| KICH (Kidney Chromophobe) | |  | |  |  |
|  | Acinetobacter | AC***C***GG (2.8, *0.85*), CCGG***T*** (3.7, *0.86*) | | | [33] |
|  | Corynebacterium | GCCG***T***AA (27.5, *0.83*) | | | [33] |
|  | Escherichia | A***T***C (1.2, *1.18*), G***A***C (1.1, *0.91*), GA***C*** (1.3, *0.91*) | | | [33] |
|  | Eubacterium | CCC***G***T (3.3, *0.94*) | | | [33] |
|  | Klebsiella | A***T***C (1.2, *1.20*), G***A***C (1.1, *1.01*), GA***C*** (1.3, *1.01*) | | | [33] |
|  | Lactococcus | CCGT***T***A (14.7, *1.13*) | | | [33] |
|  | Mycoplasma | AGA***C*** (2.2, *1.01*), GTC***G*** (2.1, *1.37*) | | | [33] |
|  | Prevotella | GA***C***GC (3.1, *0.86*), CCC***G***T (3.3, *1.01*), CGG***T***A (7.0, *1.03*) | | | [33] |
|  | Pseudomonas | GCG***A***GC (10.1, *1.00*) | | | [33] |
|  | Ruminococcus | CCC***G***T (3.3, *0.95*) | | | [33] |
|  | Shigella | A***T***C (1.2, *1.18*), G***A***C (1.1, *0.91*), GA***C*** (1.3, *0.91*) | | | [33] |
|  | Staphylococcus | CCC***G***T (3.3, *0.96*) | | | [33] |
|  | Streptococcus | A***T***C (1.2, *1.07*), G***A***C (1.1, *1.07*), GA***C*** (1.3, *1.07*), CTA***C*** (2.1, *1.09*),  GA***C***GC (3.1, *0.97*), GCC***T***A (3.4, *0.98*), GCGAT***A*** (7.5, *0.93*) | | | [33] |
|  | Weissella | CCGA***A*** (3.2, *1.04*) | | | [33] |
| KIRC (Kidney renal clear cell carcinoma) | | | | | |
|  | Bifidobacterium | AC***T***CC (2.6, *1.16*) | | | [33] |
|  | Corynebacterium | TCGCG***A*** (6.1, *0.76*) | | | [33] |
|  | Escherichia | A***T***C (1.7, *1.18*), A***T***G (1.7, *0.87*), CGA***A***AC (6.5, *1.07*),  TCGCG***A*** (6.1, *0.90*) | | | [33] |
|  | Klebsiella | A***T***C (1.7, *1.20*), A***T***G (1.7, *0.85*) | | | [33] |
|  | Mycoplasma | AA***T***C (2.1, *0.86*) | | | [33] |
|  | Prevotella | AC***T***CC (2.6, *1.03*), CGGT***A*** (3.2, *1.03*) | | | [33] |
|  | Shigella | A***T***C (1.7, *1.18*), A***T***G (1.7, *0.87*), TCGCG***A*** (6.1, *0.91*) | | | [33] |
|  | Streptococcus | A***T***C (1.7, *1.07*), A***T***G (1.7, *0.92*), GT***A***G (2.3, *1.08*),  CC***T***AC (2.7, *1.04*), CG***A***AC (2.5, *1.16*), CGAA***A*** (2.5, *0.83*) | | | [33] |
| KIRP (Kidney renal papillary cell carcinoma) | | | |  |  |
|  | Actinomyces | AA***T***T (2.4, *1.29*) | | | [33] |
|  | Bacteroides | GACGT***A***G (13.7, *1.04*) | | | [33] |
|  | Brevundimonas | AA***T***T (2.4, *1.24*) | | | [33] |
|  | Escherichia | A***T***C (1.8, *1.18*), A***T***G (1.9, *0.87*), AT***G*** (1.4, *0.87*) | | | [33] |
|  | Fusobacterium | CT***T***AC (3.1, *1.05*) | | | [33] |
|  | Klebsiella | A***T***C (1.8, *1.20*), A***T***G (1.9, *0.85*), AT***G*** (1.4, *0.85*),  CGA***T***CG (5.3, *0.87*) | | | [33] |
|  | Lactococcus | CGTC***T***AG (18.2, *1.13*) | | | [33] |
|  | Micrococcus | AA***T***T (2.4, *3.23*) | | | [33] |
|  | Prevotella | CGGT***A*** (4.6, *1.03*) | | | [33] |
|  | Pseudomonas | CGA***T***CG (5.3, *0.62*) | | | [33] |
|  | Shigella | A***T***C (1.8, *1.18*), A***T***G (1.9, *0.87*), AT***G*** (1.4, *0.87*) | | | [33] |
|  | Staphylococcus | GG***A***G (2.4, *1.31*) | | | [33] |
|  | Streptococcus | A***T***C (1.8, *1.07*), A***T***G (1.9, *0.92*), AT***G*** (1.4, *0.92*), GT***A***G (3.6, *1.08*),  CC***T***AC (3.7, *1.04*), GCC***T***A (4.3, *0.98*) | | | [33] |
| LIHC (Liver hepatocellular carcinoma) | | | | | |
|  | Bacteroides | CT***A***G (3.1, *0.19*) | | | [29] |
|  | Bifidobacterium | GCCGAA***T*** (15.3, *0.93*) | | | [34] |
|  | Enterococcus | CGC***A***G (3.9, *0.83*) | | | [29] |
|  | Helicobacter | CTC***T*** (2.4, *1.23*), GC***A***G (2.8, *0.87*), GC***T***G (2.8, *1.04*),  TC***A***G (2.4, *0.99*), TGC***A*** (2.4, *1,15*), AC***A***GA (3.1, *1.33*),  ACGC***A*** (4.0, *0.86*), CCGC***A*** (3.0, *1.11*), TCG***A***AC (5.8, *2.60*), CCGGAT***A*** (16.6, *0.95*), CGCGA***T***A (23.2, *0.93*) | | | [28] |
|  | Klebsiella | A***T***C (1.5, *1.20*), A***T***G (2.1, *0.85*), C***A***C (1.6, *0.87*), G***A***C (1.6, *1.00*), AGC***T*** (2.4, *0.91*), CC***A***GG (2.4, *0.82*), CGC***A***GG (5.0, *0.93*), GTCG***A***AC (13.5, *0.95*) | | | [27] |
| LUAD (Lung adenocarcinoma) | | | | | |
|  | Streptococcus | AT***G*** (1.3, *0.92*), CA***C*** (1.3, *0.84*), CG***G***GC (2.5, *0.86*),  ACC***C***A (2.8, *0.81*), GCC***C***A (2.5, *0.77*), GAT***G***G (2.5, *0.94*) | | | [29] |
| LUSC (Lung squamous cell carcinoma) | | | | | |
|  | Streptococcus | A***T***G (1.2, *0.92*), AT***C*** (1.2, *1.07*), AT***G*** (1.3, *0.92*), CA***C*** (1.2, *0.84*),  CG***G***GC (2.1, *0.86*), ACC***C***A (2.2, *0.81*), GAT***G***G (2.1, *0.94*) | | | [29] |
| OV (Ovarian serous cystadenocarcinoma) | | | |  |  |
|  | Acinetobacter | CGGA***T***C (3.8, *1.11*) | | | [35]^,^ [36] |
|  | Clostridium | GGAG***T*** (2.6, *0.97*) | | | [35] |
|  | Escherichia | A***T***C (1.6, *1.18*), A***T***G (1.5, *0.87*), C***A***C (1.7, *0.88*), G***A***C (1.6, *0.91*),  GT***T***C (2.0, *0.67*), CGA***A***AC (3.6, *1.07*) | | | [35] |
|  | Mycoplasma | CT***T***C (2.2, *0.95*), CCT***T***C (2.3, *1.00*), TCCCG***A*** (3.8, *1.04*) | | | [36] |
|  | Sphingomonas | TCT***T***C (2.4, *1.16*) | | | [35]^,^ [36] |
| PAAD (Pancreatic adenocarcinoma) | | | | | |
|  | Fusobacterium | CTACA***G*** (3.8, *0.75*) | | | [27] |
|  | Pseudomonas | AG***C***T (2.0, *0.91*), CACA***G*** (2.3, *0.93*), AACG***T***T (4.1, *0.76*),  CGT***A***CG (4.5, *0.72*) | | | [28] |
| PRAD (Prostate adenocarcinoma) | | | | | |
|  | Acinetobacter | ACC***G***C (2.4, *0.85*), CCAC***G*** (2.2, *0.91*) | | | [26, 37] |
|  | Escherichia | G***A***C (1.1, *0.91*), GA***C*** (1.2, *0.91*), CGA***C***AT (3.4, *0.94*) | | | [37] |
|  | Fusobacterium | GC***G***C (2.1, *0.47*), GATG***C*** (2.1, *1.00*) | | | [37] |
|  | Staphylococcus | GATG***C*** (2.1, *1.06*) | | | [26]^,^ [37] |
|  | Streptococcus | G***A***C (1.1, *1.07*), GA***C*** (1.2, *1.07*), AC***G***T (2.3, *0.77*),  ATG***C***G (2.8, *1.20*), GAC***G***C (2.6, *0.97*), CGCG***A*** (2.1, *0.83*),  GATG***C*** (2.1, *1.01*) | | | [37] |
| READ (Rectum adenocarcinoma) | | | | | |
|  | Porphyromonas | GAATT***C*** (5.2, *0.70*) | | | [30] |
|  | Prevotella | GATT***C*** (3.7, *0.91*) | | | [30] |
| SKCM (Skin Cutaneous Melanoma) | | | | | |
|  | Staphylococcus | CT***C***C (3.3, *1.30*), GAG***G*** (3.0, *1.20*), GTT***C***C (5.5, *1.08*),  TTT***C***C (9.2, *0.94*), CAAG***G*** (4.1, *0.97*), GAAG***G*** (4.5, *1.08*) | | | [26] |
|  | Corynebacterium | CTT***C***CT (7.8, *1.10*) | | | [26] |
| UCEC (Uterine Corpus Endometrial Carcinoma) | | | |  |  |
|  | Actinomyces | AAT***T*** (2.3, *1.29*) | | | [36] |
|  | Corynebacterium | AT***T***TC (2.4, *0.83*) | | | [36] |
|  | Escherichia | G***A***C (1.2, *0.91*), AT***C*** (1.2, *1.18*), GAATT***C*** (3.2, *0.74*) | | | [36] |
|  | Klebsiella | G***A***C (1.2, *1.01*), AT***C*** (1.2, *1.20*), AGTT***C***T (3.4, *0.86*) | | | [36] |
|  | Prevotella | GGCG***A***AT (5.9, *0.92*) | | | [36] |
|  | Propionibacterium | TAAAA***G*** (3.5, *0.84*) | | | [36] |
|  | Streptococcus | G***A***C (1.2, *1.07*), AT***C*** (1.2, *1.07*) | | | [36] |
| UCS (Uterine Carcinosarcoma) | |  | |  |  |
|  | Actinomyces | AA***T***T (3.3, *1.29*) | | | [36] |
|  | Corynebacterium | AT***T***TC (3.8, *0.83*) | | | [36] |
|  | Escherichia | G***A***C (1.1, *0.91*), AT***C*** (1.3, *1.18*), GA***C*** (1.1, *0.91*), GT***T***C (2.5, *0.67*) | | | [36] |
|  | Klebsiella | G***A***C (1.1, *1.01*), AT***C*** (1.3, *1.20*), GA***C*** (1.1, *1.01*), GT***T***C (2.5, *0.70*),  AGTT***C***T (5.0, *0.86*) | | | [36] |
|  | Pseudomonas | TACG***A***C (5.7, *1.41*) | | | [36] |
|  | Streptococcus | G***A***C (1.1, *1.07*), AT***C*** (1.3, *1.07*), GA***C*** (1.1, *1.07*),  CGA***A***AT (7.9, *0.92*) | | | [36] |
