# Supplementary material for: Helicobacter pylori base-excision restriction enzyme in stomach carcinogenesis
Source: PNAS Nexus. 2025 Aug 5;4(8):pgaf244. doi: 10.1093/pnasnexus/pgaf244 (PMC12366791; doi:10.1093/pnasnexus/pgaf244)
Supplement: pgaf244_Supplementary_Data [file pgaf244_supplementary_data.zip › PNASNEXUS-PNASNEXUS-2024-00952RR-s23.docx]

**Table S9. *Hp*PabI nucleotide alignments used for dN/dS calculation.**

>MHP39

GTGAGTTTGATTAAAGTTAATGATAATAAAAAAGTGATTGAGGTTTCTATTCCTTTAACT

------------TCCATTTCAGGCAAAGTTCGTGTGAAAATCAGGCACGCCTTTAGCGAT

TATGGCATTTCAACAGCGACTAGAAAAATCCCTTTCAGTTTAAAGCATTATGTAGAGTGG

CAAATCGGTTATGATGTCCCCATTAAAGATAAAGAA---AAATTTGAACTCACTACCCTA

AAAGATGAAAAATATCATTTTTTAGGGGCTAATAATAAAGTAAAAACCCTTTATGAATTG

AGCGAAATAATTGATTACGCTAAGCGATTGGGTTTAATCAGT---------TTAGAAAAT

TTAGAAAATACTTTAAAATATTTAGAAAAACAAAAACAATTTATAGAAGATAATTTTATG

ATTACAAGAGAAAGATTTAGATCGCATCAATTTGGTGGCATGGATTTTGAACTTTCACGC

ATTTCTTATCCTTTACTCATTCATTCTTTTAATGATAACCAGTTGAGTGAAATCGTTATT

AGAGAGCAACAATATGGCTCTAAAACCCAAGCCATG---CTGTATTTTTGCTTTTCTATT

CTGGAATTAAAAACCGCTACACCCTTATTAAATAGAACGGCTGCACTCAAAGAACATGCC

CTTTTAACTATCCATAAAACCAACGCTCCCATGTTTTTAGAAATGCTTAAAATTTTTGGA

CTTTTAAGCCAAGCGCACCATAACGATGTGTTAAAGATTTTAGAAAAAATACTTCAAAAT

>73

GTGAGTTTGATTAAAGTTAGTGGTGATAAAAAAGTGATTGAGATTTCTATTCCTTTAACT

------------TCAATTTCAGGCAAAGCGCGTGTGAAAATCAGACATGCCTTTAGCGAT

TATGGTATTTCAACAGCGACCAGAAAAATCCCTTTTAGTTTAAAACATTATGTAGAGTGG

CAGATCGGTTATGATGTCCCCATTAAAGATAAAGAA---AAATTTGAACTCACTACTTTA

AAAGATGAAAAATATCATTTTTTAGGGGCTAATAATAAAGTAAAAACTCTTTATGAATTG

AGTGAAATGATTTATTACGCTAAGCAATTAGATTTAATCAGT---------TTAGAAAAT

TTAGAAAATACTTTAAAATATTTAGAAAAACAAAAACAATTTATAGAAGATAATTTTATG

ATTACAAGAGAAAGATTTAGATTGCATCAATTTGGTGGCATGGATTTTGAACTCTCACGC

ATTTCTTATCCTTTGCTCATTTATTCTTTTAATGATAATCAGTTGAGCGAAATCGTTATT

AGAGAACAACAATATGGCTCTAAAACCCAAGCCATG---CTGTATTTTTGCTTTTCAATT

TTGGAGTTAAAAACCGCTACCCCCTTATTAAACAGAACGGCTATGCTCAAAGAGCATGCT

CTTTTGATTATCCATAAAACCAACGCTCCCATGTTTTTAGAAATGCTTAAAATTTTTGGA

CTTTTAAGCCAAGCGCACCATAACGATGTGTTAAAGATTTTAGAAAAAATACTTCAAAAT

>ZH76

GTGAGTTTGATTGAGATTGATAATAATAAAAAAGTAATTGAGATTTCTATTCCTTTAACT

------------TCAATTTCAGGCAAAGTGCGTGTGAAAATCAGACATGCCTTTAGCGAT

TATGGTATTTCAACAGCGACTAGAAAAATCCCTTTTAGTTTAAAACATTATGTAGAGTGG

CAGATCGGTTATGATGTCCCCATTAAAGATAAAGAA---AAATTTGAACTCACTACTTTA

AAAGATGAAAAATATCATTTTTTAGGGGCTAACAATAAAGTAAAAACTCTTTATGAATTG

AGCGAAATGATTTATTACGCTAAGCAATTAGGTTTAATCAGT---------TTAGAAAAT

TTAGAAAATACTTTAAAATATTTAGAAAAACAAAAACAATTTATAGAAGATAATTTTATG

ATCACAAGAGAAAGATTTAGATCGCATCAATTTGGTGGCATGGATTTTGAACTCTCACGC

ATTTCTTATCCCTTACTCATTCATTCTTTTGATGATAATCAGTTGAGTGAAATCGTTATT

AGAGAGCAACAATATGGCTCTAAAACCCAAGCCATG---CTGTATTTTTGCTTTTCTATT

TTGGAATTAAAAACCGCTACCCCCTTATTAAATAGAACGGCTGCACTCAAAGAACATGCC

CTTTTAACTATCCATAAAACCAACGCTCTCATGTTTTTAGAAATGCTTAAAATTTTTGGA

CTTTTAAGCCAAGCACACCATAACGATGTGTTAAAGATTTTAGAAAAAATACTTCAAAAT

>2019-5

GTGAGTTTGATTAAGATTGATAATAATAAAAAAGTAATTGAGGTTTCTATTCCTTTAACT

------------TCCATTTCAGGCAAAGTGCGTGTGAAAATCAGACATGCCTTTAGCGAT

TATGGCATTTCAACAGCGACTAGAAAAATCCCTTTCAGTTTAAAGCATTATGTAGAGTGG

CAAATCGGTTATGATGTCCCCATTAAAGATAAAGAA---AAATTTGAACTCACTACCCTA

AAAGATGAAAAATATCATTTTTTAGGGGCTAATAATAAAGTAAAGACTCTTTATGAATTG

AGCGAAATGATTTATTACGCTAAGCAATTAGGTTTAATCAGT---------TTAGAAAAT

TTAGAAAATACTTTAAAATATTTAGAAAAACAAAAACAATTTATAGAAGATAATTTTATG

ATTACAAGAGAAAGATTTAGATCGCATCAATTTGGTGGCATGGATTTTGAACTTTCACGC

ATCTCTTATCCTTTACTCATTCATTCTTTTAATGATAATCAGTTGAGCGAAATCGTTATT

AGAGAGCAACAATATGGCTCTAAAACCCAAGCCATG---CTGTATTTTTGCTTTTCTATT

TTGGAGTTAAAAACCGCTACCCCTTTATTAAATAGAACCGCTGCACTCAAAGAACATGCT

TTTTTAATTATCCATAAAACTAACGCTCTCATGTTTTTAGAAATGCTTAAAATTTTTGGA

CTTTTAAGCCAAGCGCACCATAACGATGTGTTAAAGATTTTAGAAAAAATACTTCAAAAT

>KH19

GTGAGTTTGATTAAGATTGATAATAATAAAAAAGTAATTGAGATTTCTATTCCTTTAACT

------------TCAATTTCAGGCAAAGCGCGTGTGAAAATCAGACATGCCTTTAGCGAT

TATGGTATTTCAACAGCGACCAGAAAAATCCCTTTTAGTTTAAAACATTATGTAGAGTGG

CAAATCGGTTATGATGTCCCCATTAAAGATAAAGAA---AAATTTGAACTCACTACCCTA

AAAGATGAAAAATATCATTTTTTAGGGGCTAATAATAAAATAAAAACCCTTTATGAATTG

AGCGAAATGATTTATTACGCTAAGCAATTAAATTTAATCAGT---------TTAGAAAAT

TTAGAAAATACTTTAAAATATTTAGAAAAACAAAAACAATTTATAGAAGATAATTTTATG

ATTACAAGAGAAAGATTTAGATCGCATCAATTTGGTGGCATGGATTTTGAACTTTCACGC

ATTTCTTATCCTTTGCTCATTCATTCTTTTAATGATAATCAATTGAGCGAAATCGTTATT

AGAGAGCAACAATATGGCTCTAAAACCCAAGCCATG---CTGTATTTTTGCTTTTCTATT

TTGGAATTAAAAACCGCTACCCCCTTATTAAATAGAACGGCTGCACTCAAAGAACATGCC

CTTTTAACTATCCATAAAACCAACGCTCTTATGTTTTTAGAAATGCTTAAAATTTTTGGA

CTTTTAAGCCAAGCGCACCATAGCGATGTGTTAAAGATTTTAGAAAAAATACTTCAAAAT

>HP15013

GTGAGTTTGATTAGGATTGATAATAATAAAAAAGCGATTGAGGTTTCTATTCCTTTAACT

------------TCCATTTCAGGCAAAGTGCGTGTGAAAATCAGACATGCCTTTAGCGAT

TATGGTATTTCAACAGCGACCAGAAAAATCCCTTTTAGCTTAAAACATTATGTAGAGTGG

CAAATCGGTTATGATGTCCCCATTAAAGATAAAGAA---AAATTTGAGCTCACTACCCTA

AAAGATGAAAAATATCATTTTTTAGGGGCTAATAATAAAGTGAAAACTCTTTATGAATTA

AGCGAAATGATTTATTACGCTAAGCAATTAGGTTTAATTGGT---------TTAGAAAAT

TTAGAAAATACTTTAAAATATTTAGAAAAACAAAAACAATTTATAGAAGATAATTTCACG

ATTACAAGAGAAAGATTTAGATCGCATCAATTTGGTGGCATGGATTTTGAACTCTCACGC

ATTTCTTATCCCTTACTCATTCATTCTTTTAATGATAATCAGTTGAGCGAAATTGTTATT

AGAGAGCAACAATATGGTTCTAAAACCCAAGCCATG---CTGTATTTTTGCTTTTCTATT

TTGGAATTAAAAACCGCTACTCCCTTATTAAATAGAACGGCTGCACTCAAAGAACATGCC

CTTTTAACTATCCATAAAACCAACGCTCTTATGTTTTTAGAAATGCTTAAAATTTTTGGA

CTTTTAAGCCAAGTGCACCATAGCGATGTGTTAAAGATTTTAGAAAAAATACTTCAAAAT

>3699

GTGAGTTTGATTAAGATTGATAATAATAAAAAAGTAATTGAGATTTCTATTCCTTTAACT

------------TCAATTTCAGGCAAAGTGCGTGTGAAAATCAGACATGCCTTTAGTGAT

TATGGTATTTCAACAGCGACTAGAAAAATCCCTTTTAGTTTAAAGCATTATGTAGAGTGG

CAAATCGGTTATGATGTCCCCATTAAAGATAAAGAA---AAATTGGAGCTCACTACTTTA

AAAGATGAAAAATATCATTTTTTAGGGGCTAATAATAAAGTAAAGACTCTTTATGAATTG

AGCGAAATGATTTATTACGCTAAGCGATTGGGTTTAATCAGT---------TTAGAAAAT

TTAGAAAATACTTTAAAATATTTAGAAAAACAAAAACAATTTATAGAAGATAATTTCACG

ATTACAAGAGAAAGATTCAGATCGCATCAATTTGGTGGCATGGATTTTGAACTTTCACGC

ATTTCTTATCCTTTGCTCATTCATTCTTTTAATGATAATCAATTGAGTGAAATCGTTATT

AGAGAGCAACAATATGGCTCTAAAACCCAAGCCATG---CTGTATTTTTGCTTTTCTATT

TTGGAGTTAAAAACCGCTACTCCCTTATTAAATAGAACGGCTGCACTCAAAGAACATGCC

CTTTTAACTATCCATAAAACCAACGCTCTTGTGTTTTTAGAAATGCTTAAAATTTTTGGA

CTTTTAAGCCAAGCGCACCATAACGATGTGTTAAAGATTTTAGAAAAAATACTTCAAAAT

>CC22402

GTGAGTTTGATTAGGATTGATGATAGTAAAAAAGCGATTGAGGTTTCTATTCCTTTAACT

------------TCAATTTCAGGCAAAGTGCGTGTGAAAATCAGACATGCCTTTAGCGAT

TATGGTATTTCAACAGCGACTAGAAAAATCCCTTTTAGTTTAAAACATTATATAGAGTGG

CAGATCGGTTATGATGTCCCCATTAAAGATAAAGAA---AAATTTGAACTCACTACTTTA

AAAGATGAAAAATATCATTTTTTAGGGGCTAATAATAAAGTGAAAACTCTTTATGAATTA

AGCGAAATGATTTATTACGCTAAGCAATTAGGTTTAATCAGT---------TTAGAAAAT

TTAGAAAATACTTTAAAATATTTAGAAAAACAAAAACAATTTATAGAAGATAATTTTATG

ATTACAAGAGAAAGATTTAGATTACATCAATTTGGTGGCATGGATTTTGAACTTTCACGC

ATTTCTTACCCTTTACTCATTCATTCTTTTAATGATAATCAGTTGAGCGAAATTATTATT

AGAGAGCAACAATATGGCTCTAAAACCCAAGCCATG---CTGTATTTTTGCTTTTCTATT

TTGGAATTAAAAACCGCTACTCCCTTATTAAATAGAACGGCTGCACTCAAAGAACATGCC

CTTTTAACTATCCATAAAACCAACGCTCTTATGTTTTTAGAAATGCTTAAAATTTTTGGA

CTTTTAAGCCAAGCACACCATAACGATGTGTTAAAGATTTTAGAAAAAATACTTCAAAAT

>22402

GTGAGTTTGATTAGGATTGATGATAGTAAAAAAGCGATTGAGGTTTCTATTCCTTTAACT

------------TCAATTTCAGGCAAAGTGCGTGTGAAAATCAGACATGCCTTTAGCGAT

TATGGTATTTCAACAGCGACTAGAAAAATCCCTTTTAGTTTAAAACATTATATAGAGTGG

CAGATCGGTTATGATGTCCCCATTAAAGATAAAGAA---AAATTTGAACTCACTACTTTA

AAAGATGAAAAATATCATTTTTTAGGGGCTAATAATAAAGTGAAAACTCTTTATGAATTA

AGCGAAATGATTTATTACGCTAAGCAATTAGGTTTAATCAGT---------TTAGAAAAT

TTAGAAAATACTTTAAAATATTTAGAAAAACAAAAACAATTTATAGAAGATAATTTTATG

ATTACAAGAGAAAGATTTAGATTACATCAATTTGGTGGCATGGATTTTGAACTTTCACGC

ATTTCTTACCCTTTACTCATTCATTCTTTTAATGATAATCAGTTGAGCGAAATTATTATT

AGAGAGCAACAATATGGCTCTAAAACCCAAGCCATG---CTGTATTTTTGCTTTTCTATT

TTGGAATTAAAAACCGCTACTCCCTTATTAAATAGAACGGCTGCACTCAAAGAACATGCC

CTTTTAACTATCCATAAAACCAACGCTCTTATGTTTTTAGAAATGCTTAAAATTTTTGGA

CTTTTAAGCCAAGCACACCATAACGATGTGTTAAAGATTTTAGAAAAAATACTTCAAAAT

>HP13029

GTGAGTTTGATTAAGATTGATAATAATAAAAAAGTGATTGAGGTTTCTATTCCTTTAACT

------------TCCATTTCAGGCAAAGTGCGTGTGAAAATCAGACATGCCTTTAGCGAT

TATGGTATTTCAACAGCGACTAGAAAAATCCCTTTTAGTTTAAAGCATTATGTAGAGTGG

CAAATCGGTTATGATGTCCCCATTAAAGATAAAGAA---AAATTTGAGCTCACTACTTTA

AAAGATGAAAAATATCATTTTTTAGGGGCTAATAATAAAGTAAAAACCCTTTATGAATTG

AGTGAGATAATCTATTACGCTAAGCAATTAAATTTAATCAGT---------TTAGAAAAT

TTAGAAAATACTTTAAAATATTTAGAAAAACAAAAACAATTTATAGAAGATAATTTTATG

ATTACAAGAGAAAGATTTAGATCGCATCAATTTGGTGGCATGGATTTTGAACTCTCACGC

ATTTCTTATCCTTTGCTCATTCATTCTTTTAATGATAATCAGTTGAGCGAAATTGTTATT

AGAGAACAACAATATGGCTCTAAAACCCAAGCCATG---CTGTATTTTTGCTTTTCTATT

TTGGAGTTAAAAACCGCTACCCCTTTATTAAATAGGACCGCTGCACTCAAAGAACATGCC

ATTTTAACTATCCATAAAACCAACGCTCCCATGTTTTTAGAAATGCTTAAAATTTTTGGA

CTTTTAAGCCAAGCGCACCATAACGATGTGTTAAAGATTTTAGAAAAAATACTTCAAAAT

>ZH12

GTGAGTTTGATTAAGATTGATAATAATAAAAAAGTAATTGAGGTTTCTATTCCTTTAACT

------------TCCATTTCAGGCAAAGTGCGTGTGAAAATCAGACATGCCTTTAGCGAT

TATGGCATTTCAACAGCGACTAGAAAAATCCCTTTTAGTTTAAAGCATTATGTAGAGTGG

CAAATCGGTTATGATGTCCCTATTAAAGATAAAGAA---AAATTTGAGCTCACTACTTTA

AAAGATGAAAAATATCATTTTTTAGGGGCTAATAATAAAGTAAAGACTCTTTATGAATTG

AGTGAGATAATCTATTACGCTAAGCAATTAAATTTAATCAGT---------TTAGAAAAT

TTAGAAAATACTTTAAAATATTTAGAAAAACAAAAACAATTTATAGAAGATAATTTCACG

ATTACAAGAGAAAGATTTAGATCGCATCAATTTGGTGGCATGGATTTTGAACTTTCACGC

ATTTCTTATCCTTTACTCATTCATTCTTTTAATGATAATCAATTGAGTGAAATCGTTATT

AGAGAGCAACAATATGGCTCTAAAACCCAAGCCATG---CTGTATTTTTGCTTTTCTATT

TTGGAATTAAAAACCGCTACTCCCTTATTAAATAGAACGGCTGCACTCAAAGAACATGCC

CTTTTAACTATCCATAAAACCAACGCTCTTATGTTTTTAGAAATGCTTAAAATTTTTGGA

CTTTTAAGCCAAGCGCACCATAGCGATGTGTTAAAGATTTTAGAAAAAATACTTCAAAAT

>ZH75

GTGAGTTTGATTAGGATTGATGATAGTAAAAAAGCGATTGAGGTTTCTATTCCTTTAACT

------------TCCATTTCAGGCAAAGTGCGTGTGAAAATCAGACATGCCTTTAGCGAT

TATGGCATTTCAACAGCGACTAGAAAAATCCCTTTTAGTTTAAAACATTATGTAGAGTGG

AAAATCGGTTATGATGTCCCCATTAAAGATAAAGAA---AAATTTGAACTCACTACTTTA

AAAGATGAAAAATATCATTTTTTAGGGGCTAATAATAAAGTAAAGACTCTTTATGAATTG

AGCGAAATGATTTATTACGCTAAGCGATTGGGTTTAATCAGT---------TTAGAAAAT

TTAGAAAATACTTTAAAATATTTAGAAAAACAAAAACAATTTATAGAAGATAATTTTATG

ATTACAAGAGAAAGATTTAGATCGCATCAATTTGGTGGCATGGATTTTGAACTTTCACGC

ATTTCTTACCCTTTACTCATTCATTCTTTCAATGATAATCAGTTGAGCGAAATCGTTATT

AGAGAGCAACAATACGGCTCTAAAACCCAAGCCATG---CTGTATTTTTGCTTTTCTATT

TTGGAATTAAAAACCGCTACTCCCTTATTAAACAGAACGGCTGCACTCAAAGAACATGCC

CTTTTAACTATCCATAAAACCAACGCTCTTGTGTTTTTAGAAATGCTTAAAATTTTTGGA

CTTTTAAGCCAAGCACACCATAACGATGTGTTAAAGATTTTAGAAAAAATACTTCAAAAT

>ZH128

GTGAGTTTGATTAGGATTGATAATAATAAAAAAGTAATTGGGGTTTCTATTCCTTTAACT

------------TCAATTTCAGGCAAAGTGCGTGTGAAAATCAGACATGCCTTTAGCGAT

TATGGTATTTCAACAGCGACCAGAAAAATCCCTTTTAGCTTAAAACATTATGTAGAGTGG

CAAATCGGTTATGATGTCCCCATTAAAGATAAAGAA---AAATTTGAGCTCACTACCCTA

AAAGATGAAAAATATCATTTTTTAGGGGCTAATAATAAAGTGAAAACTCTTTATGAATTA

AGCGAAATGATTTATTACGCTAAGCAATTAGGTTTAATTGGT---------TTAGAAAAT

TTAGAAAATACTTTAAAATATTTAGAAAAACAAAAACAATTTATAGAAGATAATTTCACG

ATTACAAGAGAAAGATTTAGATCGCATCAATTTGGTGGCATGGATTTTGAACTCTCACGC

ATTTCTTATCCCTTACTCATTCATTCTTTTAATGATAATCAGTTGAGCGAAATTGTTATT

AGAGAGCAACAATATGGTTCTAAAACCCAAGCCATG---CTGTATTTTTGCTTTTCTATT

TTGGAATTAAAAACCGCTACTCCCTTATTAAATAGAACGGCTGCACTCAAAGAACATGCC

CTTTTAACTATCCATAAAACCAACGCTCTTATGTTTTTAGAAATGCTTAAAATTTTTGGA

CTTTTAAGCCAAGTGCACCATAGCGATGTGTTAAAGATTTTAGAAAAAATACTTCAAAAT

>Nic35-A

GTGAGTTTGATTAGGATTGATGATAGTAAAAAAGCGATTGAGGTTTCCATTCCTTTAACT

------------TCAATTTCAGGCAAAGCGCGTGTGAAAATCAGACATGCCTTTAGCGAT

TATGGCATTTCAACAGCGACCAGAAAAATCCCTTTTAGTTTAAAGCATTATGTAGAGTGG

CAAATCGGTTATGATGTCCCCATTAAAGATAAAGAA---AAATTTAAACTCACTACTTTA

AAAGATGAAAAATACCATTTTTTAGGGGCTAATAATAAAGTAAAAACTCTTTATGAATTG

AGCGAAATGATTTATTACGCTAATCAATTAGGTTTAATCAGT---------TTAGAAAAT

TTAGAAAATACTTTAAAATATTTAGAAAAACAAAAACAATTCATAGAAGATAATTTTATG

ATTACAAGAGAAAGATTTAGATCGCATCAATTTGGTGGCATGGATTTTGAACTTTCACGC

ATTTCTTATCCTTTACTTATTCATTCTTTTAATGATAATCAATTGAGCGAAATCGTTATT

AGAGAGCAACAATATGGCTCTAAAACCCAAGCCATG---CTGTATTTTTGCTTTTCTATT

TTGGAATTAAAAACCGCTACCCCCTTATTAAATAGAACGGCTGCACTCAAAGAACATGCC

CTTTTAACTATCCATAAAACCAACGCTCTCATGTTTTTAGAAATGCTTAAAATTTTTGGA

CTTTTAAGCCAAGCGCACCATAACGATGTGTTAAAGATTTTAGAAAAAATACTTCAAAAT

>HP04086

GTGAGTTTGATTAGGATTGATAATAATAAAAAAGTAATTGGGGTTTCTATTCCTTTAACT

------------TCCATTTCAGGCAAAGCACGTGTGAAAATCAGACATGCCTTTAGCGAT

TATGGCATTTCAACAGCGACTAGAAAAATCCCTTTTAGTTTAAAGCATTATGTAGAGTGG

CAAATCGGTTATGATGTCCCCATTAAAGATAAAGAA---AAATTTGAACTCACTACTTTA

AAAGATGAAAAATATCATTTTTTAGGGGCTAATAATAAAGTAAAAACTCTTTATGAATTG

AGTGAAATGATTTATTACGCTAAGCAATTAGGTTTAATCAGT---------TTAGAAAAT

TTAGAAAATACTTTAAAATATTTAGAAAAACAAAAACAATTTATAGAAGATAATTTCACG

ATTACAAGAGAAAGATTTAGATCGCATCAATTTGGTGGCATGGATTTTGAACTTTCACGC

ATTTCTTATCCTTTACTCATTCATTCTTTTAATGATAATCAATTGAGTGAAATCGTTATT

AGAGAGCAACAATATGGCTCTAAAACCCAAGCCATG---CTGTATTTTTGCTTTTCTATT

TTGGAATTAAAAACCGCTACCCCCTTATTAAATAGAACGGCTGCACTCAAAGAACATGCT

TTTTTAACCATCAATAAAACCAACGCTCTTATGTTTTTAGAAATGCTTAAAATTTTTGGA

CTTTTAAGCCAAGCGCACCATAACGATGTGTTAAAGATTTTAGAAAAAATACTTCAAAAT

>ZH106

GTGAGTTTGATTAGGATTGATAATAATAAAAAAGTAATTGGGGTTTCTATTCCTTTAACT

------------TCAATTTCAGGCAAAGTGCGTGTGAAAATCAGACATGCCTTTAGCGAT

TATGGTATTTCAACAGCGACTAGAAAAATCCCTTTTAGCTTAAAACATTATGTAGAGTGG

CAAATCGGTTATGATGTCCCCATTAAAGATAAAGAA---AAATTTGAGCTCACTACCCTA

AAAGATGAAAAATATCATTTTTTAGGGGCTAATAATAAAGTAAAAACCCTTTATGAATTG

AGTGAGATAATCTATTACGCTAAGCAATTAAATTTAATCAGT---------TTAGAAAAT

TTAGAAAATACTTTAAAATATTTAGAAAAACAAAAACAATTTATAGAAGATAATTTTATG

ATTACAAGAGAAAGATTTAGATCACATCAATTTGGTGGCATGGATTTTGAACTTTCACGC

ATTTCTTATCCTTTGCTCATTCATTCTTTTAATGATAATCAGTTGAGCGAAATTGTTATT

AGAGAGCAACAATATGGCTCTAAAACCCAAGCCATG---CTGTATTTTTGCTTTTCTATT

TTGGAGTTAAAAACCGCTACCCCCTTATTAAATAGAACCGCTACACTCAAAGAACATGCT

CTTTTGATTATCCATAAAACCAACGCTCTCATGTTTTTAGAAATGCTTAAAATTTTTGGA

CTTTTAAGCCAAGCGCACCATAACGATGTGTTAAAGATTTTAGAAAAAATACTTCAAAAT

>G-Mx-2003-136

GTGAGTTTGATTAAGATTGATAATAATAAAAAAGTAATTGAGGTTTCTATTCCTTTAACT

------------TCCATTTCAGGCAAAGTGCGTGTGAAAATCAGACATGCCTTTAGCGAT

TATGGCATTTCAACAGCGACTAGAAAAATCCCTTTTAGTTTAAAGCATTATGTAGAGTGG

CAAATCGGTTATGATGTCCCTATTAAAGATAAAGAA---AAATTTGAGCTCACTACTTTA

AAAGATGAAAAATATCATTTTTTAGGGGCTAATAATAAAGTAAAAACCCTTTATGAATTG

AGTGAAATAATTTATTACGCTAAGCGATTGGGTTTAATCGGT---------TTAGAAAAT

TTAGAAAATACTTTAAAATATTTAGAAAAACAAAAACAATTCATAGAAGATAATTTTACG

ATTACAAGAGAAAGATTTAGATCGCATCAATTTGGTGGCATGGATTTTGAACTTTCACGC

ATTTCTTACCCTTTACTCATTCATTCTTTCAATGATAATCAGTTGAGCGAAATCGTTATT

AGAGAGCAACAATACGGCTCTAAAACCCAAGCCATG---CTGTATTTTTGCTTTTCTATT

TTGGAATTAAAAACCGCTACTCCCTTATTAAACAGAACGGCTGCACTCAAAGAACATGCC

CTTTTAACTATCCATAAAACCAACGCTCTTGTGTTTTTAGAAATGCTTAAAATTTTTGGA

CTTTTAAGCCAAGCACACCATAACGATGTGTTAAAGATTTTAGAAAAAATACTTCAAAAT

>ZH133

GTGAGTTTGATTAAGATTGATAATGATAAAAAAGCGATTGAGGTTTCTATTCCTTTAACT

------------TCAATTTCAGGCAAAGTGCGTGTGAAAATCAGACATGCCTTTAGCGAT

TATGGTATTTCAACAGCGACTAGAAAAATCCCTTTTAGTTTAAAACATTATATAGAGTGG

CAGATCGGTTATGATGTCCCCATTAAAGATAAAGAA---AAATTTGAACTCACTACTTTA

AAAGATGAAAAATATCATTTTTTAGGGGCTAATAATAAAGTGAAAACTCTTTATGAATTA

AGCGAAATGATTTATTACGCTAAGCAATTAGGTTTAATCAGT---------TTAGAAAAT

TTAGAAAATACTTTAAAATATTTAGAAAAACAAAAACAATTTATAGAAGATAATTTTATG

ATTATAAGAGAAAGATTTAGATCGCATCAATTTGGTGGCATGGATTTTGAACTCTCACGC

ATTTCTTATCCCTTACTCATTCATTCTTTTAATGATAATGAGTTGAGCGAAATCGTTATT

AGAGAGCAACAATATGGCTCTAAAACCCAGGCCATG---CTGTATTTTTGCTTTTCTATT

TTGGAATTAAAAACCGCTACTCCCTTATTAAATAGAACGGCTGCACTCAAAGAACATGCC

CTTTTAACTATCCATAAAACCAACGCTCTTGTGTTTTTAGAAATGCTTAAAATTTTTGGA

CTTTTAAGCCAAGCGCACCATAACGATGTGTTAAAGATTTTAGAAAAAATACTTCAAAAT

>KH40

GTGAGTTTGATTAAAGTTGACTATGATAAAAAAGTGATTGAGGTTTCTATTCCTTTAACT

------------TCAATTTCAGGCAAAGTGCGTGTGAAAATCAGACATGCCTTTAGCGAT

TATGGTATTTCAACAGCGACTAGAAAAATCCCTTTTAGTTTAAAACATTATGTAGAGTGG

CAGATCGGTTATGATGTCCCCATTAAAGATAAAGAA---AAATTTGAACTCACTACTTTA

AAAGATGAAAAATATCATTTTTTAGGAGCTAATAATAAAGTAAAAACTCTTTATGAATTG

AGCGAAATGATTTATTACGCTAAGCAATTAGGTTTAATCAGT---------TTAGAAAAT

TTAGAAAATACTTTAAAATATTTAGAAAAACAAAAACAATTTATAGAAGATAATTTTATG

ATTGCAAGAGAAAGATTTAGATCGCATCAATTTGGCGGCATGGATTTTGAACTTTCACGC

ATTTCTTATCCTTTACTCATTCATTCTTTTAACGATAATCAATTGAGTGAAATAGTTATT

AGAGAACAACAATACGGCTCTAAGACCCAAGCCATG---CTGTATTTTTGCTTTTCTATT

TTGGAGTTAAAAACCGCTACTCCCTTATTAAACAGAACGGCTGCACTCAAAGAACATGCC

CTTTTAACTATCCATAAAACCAACGCTCTTGTGTTTTTAGAAATGCTTAAAATTTTTGGG

CTTTTAAGCCAAGCGCACCATAACGATGTGGTGAAGATTTTAGAAAAAATACTTCAAAAT

>HP12038

GTGAGTTTGATTAAGATTGATGATGATAAAAAAGCGATTGAGGTTTCTATTCCTTTAACT

------------TCAATTTCAGGCAAAGTGCGTGTGAAAATCAGACATGCCTTTAGCGAT

TATGGTATTTCAACAGCGACTAGAAAAATCCCTTTTAGTTTAAAACATTATGTAGAGTGG

CAAATCGGTTATGATGTCCCCATTAAAGATAAAGAA---AAATTTGAGCTCACTACCCTA

AAAGATGAAAAATATCATTTTTTAGGGGCTAATAATAAAATAAAAACCCTTTATGAATTG

AGCGAAATGATTTATTACGCTAAGCAATTAAATTTAATCAGT---------TTAGAAAAT

TTAGAAAATACTTTAAAATATTTAGAAAAACAAAAACAATTTATAGAAGATAATTTTATG

ATTACAAGAGAAAGATTTAGATCGCATCAATTTGGTGGCATGGATTTTGAACTTTCACGC

ATTTCTTATCCTTTGCTCATTCATTCTTTTAATGATAATCAATTGAGCGAAATCGTTATT

AGAGAGCAACAATATGGCTCTAAAACCCAAGCCATG---CTGTATTTTTGCTTTTCTATT

TTGGAATTAAAAACCGCTACCCCCTTATTAAATAGAACGGCTGCACTCAAAGAACATGCC

CTTTTAACTATCCATAAAACCAACGCTCTTATGTTTTTAGAAATGCTTAAAATTTTTGGA

CTTTTAAGCCAAGCGCACCATAGCGATGTGTTAAAGATTTTAGAAAAAATACTTCAAAAT

>HP16056

GTGAGTTTGATTGAGATTGATAATAATAAAAAAGTAATTGAGATTTCTATTCCTTTAACT

------------TCAATTTCAGGCAAAGTGCGTGTGAAAATCAGACATGCCTTTAGCGAT

TATGGTATTTCAACAGCGACTAGAAAAATCCCTTTTAACTTAAAACATTATGTAGAGTGG

CAAATCGGTTATGATGTCCCCATTAAAGATAAAGAA---AAATTTGAGCTCACTACTTTA

AAAGATGAAAAATATCATTTTTTAGGGGCTAATAATAAAGTAAAAACTCTTTATGAATTG

AGTGAAATAATCTATTACGCTAAGCAATTAAATTTAATCAGT---------TTAGAAAAT

TTAGAAAATACTTTAAAATATTTAGAAAAACAAAAACAATTTATAGAAGATAATTTTATG

ATTACAAGAGAAAGATTTAGATTACATCAATTTGGTGGCATGGATTTTGAACTCTCACGC

ATTTCTTATCCTTTACTCATTCATTCTTTTAATGATAATCAGTTGAGCGAAATCGTTATT

AGAGAGCAACAATATGGCTCTAAAACCCAAGCCATG---CTGTATTTTTGCTTTTCTATT

TTGGAATTAAAAACCGCTACCCCCTTATTAAATAGAACGGCTGCACTCAAAGAACATGCC

CTTTTAACTATCCATAAAACCAACGCTCTCATGTTTTTAGAAATGCTTAAAATTTTTGGA

CTTTTAAGCCAAGCACACCATAACGATGTGTTAAAGATTTTAGAAAAAATACTTCAAAAT

>ZH66

GTGAGTTTGATTAGGATTGATAATAATAAAAAAGTAATTGGGGTTTCTATTCCTTTAACT

------------TCCATTTCAGGCAAAGTGCGTGTGAAAATCAGACATGCCTTTAGCGAT

TATGGTATTTCAACAGCGACTAGAAAAATCCCTTTTAGCTTAAAGCATTATGTAGAGTGG

CAAATCGGTTATGATGTCCCCATTAAAGATAAAGAA---AAATTTGAGCTCACTACTTTA

AAAGATGAAAAATATCATTTTTTAGGGGCTAATAATAAAGTAAAAACCCTTTATGAATTG

AGTGAGATAATCTATTACGCTAAGCAATTAAATTTAATCAGT---------TTAGAAAAT

TTAGAAAATACTTTAAAATATTTAGAAAAACAAAAACAATTTATAGAAGATAATTTCACG

ATTACAAGAGAAAGATTTAGATCGCATCAATTTGGTGGCATGGATTTTGAACTCTCACGC

ATTTCTTATCCTTTGCTCATTCATTCTTTTAATGATAATCAATTGAGTGAAATCGTTATT

AGAGAGCAACAATATGGCTCTAAAACCCAAGCCATG---CTGTATTTTTGCTTTTCTATT

TTGGAGTTAAAAACCGCTACCCCCTTATTAAATAGAACGGCTGCACTCAAAGAACAGGCT

CTTTTAACTATCCATAAAACCAACGCTCTTATGTTTTTAGAAATGCTTAAAATTTTTGGA

CTTTTAAGCCAAGCACACCATAACGATGTGTTAAAGATTTTAGAAAAAATACTTCAAAAT

>22395

GTGAATTTGATTAAGATTGATAATAATAAAAAAGTAATTGAGATTTCTGTTCCTTTAACT

------------TCAATTTCAGGCAAAGTGCGTGTGAAAATCAGACATGCCTTTAGCGAT

TATGGTATTTCAACAGCGACTAGAAAAATCCCTTTTAGCTTAAAACATTATGTAGAGTGG

CAAATCGGTTATGATGTCCCCATTAAAGATAAAGAA---AAATTGGAGCTCACTACCCTA

AAAGATGAAAAATATCATTTTTTAGGGGCTAATAATAAAGTAAAAACCCTTTATGAATTG

AGTGAGATAATCTATTACGCTAAGCAATTAAATTTAATCAGT---------TTAGAAAAT

TTAGAAAATACTTTAAAATATTTAGAAAAACAAAAACAATTTATAGAAGATAATTTTATG

ATTACAAGAGAAAGATTTAGATCGCATCAATTTGGTGGCATGGATTTTGAACTCTCACGC

ATTTCTTATCCTTTGCTCATTCATTCTTTTAATGATAATCAGTTGAGCGAAATCGTTATT

AGAGAGCAACAATATGGCTCTAAAACCCAAGCCATG---CTGTATTTTTGCTTTTCTATT

TTGGAGTTAAAAACCGCTACTCCCTTATTAAACAGAACGGCTGCACTCAAAGAACATGCC

CTTTTAACTATCCATAAAACCAACGCTCTTATGTTTTTAGAAATGCTTAAAATTTTTGGA

CTTTTAAGCCAAGTGCACCATAACGATGTGTTAAAGATTTTAGAAAAAATACTTCAAAAT

>MCms1063

GTGAGTTTGATTAAGATTGATAATAATAAAAAAGTAATTGAGGTTTCTATTCCTTTAACT

------------TCCATTTCAGGCAAAGCGCGTGTGAAAATCAGACATGCCTTTAGCGAT

TATGGCATTTCAACAGCGACTAGAAAAATCCCTTTTAGTTTAAAGCATTATGTAGAGTGG

CAAATCGGTTATGATGTCCCTATTAAAGATAAAGAA---AAATTTGAGCTCACTACTTTA

AAAGATGAAAAATATCATTTTTTAGGGGCTAATAATAAAGTAAAAACCCTTTATGAATTG

AGTGAAATAATTTATTACGCTAAGCGATTGGGTTTAATCGGT---------TTAGAAAAT

TTAGAAAATACTTTAAAATATTTAGAAAAACAAAAACAATTCATAGAAGATAATTTTACG

ATTACAAGAGAAAGATTTAGATCGCATCAATTTGGTGGCATGGATTTTGAACTTTCACGC

ATTTCTTACCCTTTACTCATTCATTCTTTCAATGATAATCAGTTGAGCGAAATCGTTATT

AGAGAGCAACAATACGGCTCTAAAACCCAAGCCATG---CTGTATTTTTGCTTTTCTATT

TTGGAATTAAAAACCGCTACTCCCTTATTAAACAGAACGGCTGCACTCAAAGAACATGCC

CTTTTAACTATCCATAAAACCAACGCTCTTGTGTTTTTAGAAATGCTTAAAATTTTTGGA

CTTTTAAGCCAAGCACACCATAACGATGTGTTAAAGATTTTAGAAAAAATACTTCAAAAT

>G-Mx-2010-64

GTGAGTTTGATTAAGATTGATAATAATAAAAAAGTAATTGAGGTTTCTATTCCTTTAACT

------------TCCATTTCAGGCAAAGCGCGTGTGAAAATCAGACATGCCTTTAGCGAT

TATGGCATTTCAACAGCGACTAGAAAAATCCCTTTTAGTTTAAAGCATTATGTAGAGTGG

CAAATCGGTTATGATGTCCCTATTAAAGATAAAGAA---AAATTTGAGCTCACTACTTTA

AAAGATGAAAAATATCATTTTTTAGGGGCTAATAATAAAGTAAAAACCCTTTATGAATTG

AGTGAAATAATTTATTACGCTAAGCGATTGGGTTTAATCGGT---------TTAGAAAAT

TTAGAAAATACTTTAAAATATTTAGAAAAACAAAAACAATTCATAGAAGATAATTTTACG

ATTACAAGAGAAAGATTTAGATCGCATCAATTTGGTGGCATGGATTTTGAACTTTCACGC

ATTTCTTACCCTTTACTCATTCATTCTTTCAATGATAATCAGTTGAGCGAAATCGTTATT

AGAGAGCAACAATACGGCTCTAAAACCCAAGCCATG---CTGTATTTTTGCTTTTCTATT

TTGGAATTAAAAACCGCTACTCCCTTATTAAACAGAACGGCTGCACTCAAAGAACATGCC

CTTTTAACTATCCATAAAACCAACGCTCTTGTGTTTTTAGAAATGCTTAAAATTTTTGGA

CTTTTAAGCCAAGCACACCATAACGATGTGTTAAAGATTTTAGAAAAAATACTTCAAAAT

>M-Mx-2008-34

GTGAGTTTGATTAAGATTGATAATAATAAAAAAGTAATTGAGGTTTCTATTCCTTTAACT

------------TCCATTTCAGGCAAAGTGCGTGTGAAAATCAGACATGCCTTTAGCGAT

TATGGCATTTCAACAGCGACTAGAAAAATCCCTTTTAGTTTAAAGCATTATGTAGAGTGG

CAAATCGGTTATGATGTCCCCATTAAAGATAAAGAA---AAATTTGAGCTCACTACTTTA

AAAGATGAAAAATATCATTTTTTAGGGGCTAATAATAAAGTAAAAACCCTTTATGAATTG

AGTGAAATAATTTATTACGCTAAGCGATTGGGTTTAATCGGT---------TTAGAAAAT

TTAGAAAATACTTTAAAATATTTAGAAAAACAAAAACAATTCATAGAAGATAATTTTACG

ATTACAAGAGAAAGATTTAGATCGCATCAATTTGGTGGCATGGATTTTGAACTTTCACGC

ATTTCTTACCCTTTACTCATTCATTCTTTCAATGATAATCAGTTGAGCGAAATCGTTATT

AGAGAGCAACAATACGGCTCTAAAACCCAAGCCATG---CTGTATTTTTGCTTTTCTATT

TTGGAATTAAAAACCGCTACTCCCTTATTAAATAGAACGGCTGCACTCAAAGAACATGCT

TTTTTAACTATCCATAAAACCAACGCTCTTGTGTTTTTAGAAATGCTTAAAATTTTTGGA

CTTTTAAGCCAAGCGCACCATAACGATGTGTTAAAGATTTTAGAAAAAATACTTCAAAAT

>PZ5006_3A3

GTGAGTTTGATTAAAGTTAATGATGATAAAAAAGCGATTGAGGTTTCTATTCCTTTAACT

------------TCCATTTCAGGCAAAGTTCGTGTGAAAATCAGGCATGCCTTTAGCGAT

TATGGTATTTCAACAGCGACTAGAAAAATCCCTTTCAGTTTAAAGCATTATGTAGAGTGG

CAAATCGGTTATGATGTCCCCATTAAAGATAAAGAA---AAATTTGAACTCACTACCCTA

AAAGATGAAAAATATCATTTTTTAGGGGCTAATAATAAAGTAAAGACTCTTTATGAATTG

AGCGAAATGATTTATTACGCTAAGCGATTGGGTTTAATCAGT---------TTAGAAAAT

TTAGAAAATACTTTAAAATATTTAGAAAAACAAAAACAATTCATAGAAGATAATTTTATG

ATTACAAGAGAAAGATTTAGATCGCATCAATTTGGGGGCATGGATTTTGAACTTTCACGC

ATTTCTTATCCTTTACTCATTCATTCTTTTAATGATAACCAATTGAGTGAAATCGTTATT

AGAGAGCAACAATACGGCTCTAAAACCCAAGCCATG---CTGTATTTTTGCTTTTCTATT

CTAGAATTAAAAACCGCTACCCCCTTATTAAATAGAACGGCTGCACTCAAAGAACATGCC

CTTTTAACTATCCATAAAACCAACGCTCTTGTGTTTTTAGAAATGCTTAAAATTTTTGGC

CTTTTAAGCCAAGCGCACCATAACGATGTGTTAAAGATTTTAGAAAAAATACTTCAAAAT

>BM013B

GTGAGTTTGATTAAGATTGATAATAATAAAAAAGTAATTGAGATTTCTATTCCTTTAACT

------------TCAATTTCAGGCAAAGTGCGTGTGAAAATCAGGCATGCCTTTAGTGAT

TATGGTATTTCAACAGCGACCAAAAAAATCCCTTTTAGTTTAAAACATTATATAGAGTGG

CAGATCGGTTATGATGTCCCCATTAAAGATAAAGAA---AAATTTGAACTCACTGCTTTA

AAAGATGAAAAATACCATTTTTTAGGGGCTAATAATAAAGTAAAAACTCTTTATGAATTG

AGCGAAATGATTGATTACGCTAAGCAATTAGGTTTAATCAGT---------TTAGAAAAT

TTAGAAAATACTTTAAAATATTTAGAAAAACAAAAACAATTTATAGAAGATAATTTTATG

ATTACAAGAGAAAGATTTAGATCGCATCAATTTGGTGGCATGGATTTTGAACTTTCACGC

ATTTCTTATCCTTTACTCATTCATTCTTTTAATGATAATCAATTGAGTGAAATCGTTATT

AGAGAGCAACAATACGGCTCTAAAACCCAAGCCATG---CTGTATTTTTGCTTTTCTATT

TTGGAATTAAAAACCGCTACCCCTTTATTAAATAGAACGGCTGCACTCAAAGAACAGGCT

CTTTTAACCATCCATAAAACCAACGCTCTTATGTTTTTAGAAATGCTTAAAATTTTTGGA

CTTTTAAGCCAAGCGCACCATAACGATGTGTTAAAAATTTTAGAAAAAATACTTCAAAAT

>BM013A

GTGAGTTTGATTAAGATTGATAATAATAAAAAAGTAATTGAGATTTCTATTCCTTTAACT

------------TCAATTTCAGGCAAAGTGCGTGTGAAAATCAGGCATGCCTTTAGTGAT

TATGGTATTTCAACAGCGACCAAAAAAATCCCTTTTAGTTTAAAACATTATATAGAGTGG

CAGATCGGTTATGATGTCCCCATTAAAGATAAAGAA---AAATTTGAACTCACTGCTTTA

AAAGATGAAAAATACCATTTTTTAGGGGCTAATAATAAAGTAAAAACTCTTTATGAATTG

AGCGAAATGATTGATTACGCTAAGCAATTAGGTTTAATCAGT---------TTAGAAAAT

TTAGAAAATACTTTAAAATATTTAGAAAAACAAAAACAATTTATAGAAGATAATTTTATG

ATTACAAGAGAAAGATTTAGATCGCATCAATTTGGTGGCATGGATTTTGAACTTTCACGC

ATTTCTTATCCTTTACTCATTCATTCTTTTAATGATAATCAATTGAGTGAAATCGTTATT

AGAGAGCAACAATACGGCTCTAAAACCCAAGCCATG---CTGTATTTTTGCTTTTCTATT

TTGGAATTAAAAACCGCTACCCCTTTATTAAATAGAACGGCTGCACTCAAAGAACAGGCT

CTTTTAACCATCCATAAAACCAACGCTCTTATGTTTTTAGAAATGCTTAAAATTTTTGGA

CTTTTAAGCCAAGCGCACCATAACGATGTGTTAAAAATTTTAGAAAAAATACTTCAAAAT

>KH1

GTGAGTTTGATTAGGATTGATGATAGTAAAAAAGTAATTGAGGTTTCTATTCCTTTAACT

------------TCAATTTCAGGCAAAGTGCGTGTGAAAATCAGGCATGCCTTTAGCGAT

TATGGTGTTTCAACAGCGACTAGAAAAATCCCTTTTAGTTTAAAGCATTATGTAGAGTGG

CAAATCGGTTATGATGTCCCCATTGAAGATAAAGAA---AAATTTGAGCTCACTACCCTA

AAAGATGAAAAATATCATTTTTTAGGGGCTAATAATAAAGTAAAAACTCTTTATGAATTG

AGCGAAATGATTTATTACGCTAAGCAATTAGGTTTAATCAGT---------TTAGAAAAT

TTAGAAAATACTTTAAAATATTTAGAAAAACAAAAACAATTTATAGAAGATAATTTCACG

ATTACAAGAGAAAGATTTAGATCGCATCAATTTGGGGGCATGGATTTTGAACTTTCACGC

ATTTCTTATCCCTTACTCATTCATTCTTTTAATGATAATCAATTGAGCGAAATCGTTATT

AGAGAACAACAATACGGCTCTAAGACACAAGCCATG---CTGTATTTTTGCTTTTCTATT

TTGGAGTTAAAAACCGCTACTCCCTTATTAAATAGAACCGCTACGCTCAAAGAACATGCC

CTTTTGATTATCCATAAAACCAACGCCCTCATGTTTTTAGAAATGCTTAAAATTTTTGGG

CTTTTAAGCCAAGCGCACCATAACGATGTGTTAAAGATTTTAGAAAAAATACTTCAAAAT

>GC23-HL

GTGAGTTTGATTAGGATTGATGATAGTAAAAAAGCGATTGAGGTTTCCATTCCTTTAACT

------------TCAATTTCAGGCAAAGTGCGTGTGAAAATCAGACATGCTTTTAGCGAT

TATGGCATTTCAACAGCGACCAGAAAAATCCCTTTTAGTTTAAAACATTATGTAGAGTGG

CAAATCGGTTATGATGTCCCCATTAAAGATAAAGAA---AAATTTGAACTCACTACTTTA

AAAGATGAAAAATATCATTTTTTAGGGGCTAATAATAAAGTAAAGACTCTTTATGAATTG

AGCGAAATGATTTATTACGCTAAGCGATTGGGTTTAATCAGT---------TTAGAAAAT

TTAGAAAATACTTTAAAATATTTAGAAAAACAAAAACAATTTATAGAAGATAATTTTATG

ATTACAAGAGAAAGATTTAGATCGCATCAATTTGGTGGCATGGATTTTGAACTCTCACGC

ATTTCTTATCCTTTACTCATTCATTCTTTCAACGATAATCAATTGAGCGAAATAGTTATT

AGAGAGCAACAATACGGCTCTAAAACCCAAGCCATG---CTGCATTTTTGCTTTTCTATT

TTGGAGTTAAAAACCGCTACTCCCTTATTAAATAGAACGGCTGCACTCAAAGAACATGCC

CTTTTAACTATCCATAAAACCAACGCTCTTATGTTTTTAGAAATGCTTAAAATTTTTGGA

CTTTTAAGCCAAGCGCACCATAGCGATGTGTTAAAGATTTTAGAAAAAATACTTGAAAAT

>B659-C2

GTGAGTTTGATTGAGATTGATAATAATAAAAAAGTGATTGAGGTTTCTATTCCTTTAACT

------------TCCATTTCAGGCAAAGTGCGTGTGAAAATCAGACATGCCTTTAGCGAT

TATGGCATTTCAACAGCGACCAGAAAAATCCCTTTTAGTTTAAAACATTATGTAGAGTGG

CAAATCGGTTATGATGTCCCCATTAAAGATAAAGAA---AAATTTGAGCTCACTACTTTA

AAAGATGAAAAATATCATTTTTTAGGGGCTAACAATAAAGTAAAAACCCTTTATGAATTG

AGTGAGATAATCTATTATGCTAAGCAATTAAATTTAATCAGT---------TTAGAAAAT

TTAGAAAATACTTTAAAATATTTAGAAAAACAAAAACAATTTATAGAAGATAATTTCACG

ATTACAAGAGAAAGATTTAGATCGCATCAATTTGGTGGCATGGATTTTGAACTTTCACGC

ATTTCTTATCCTTTACTCATTCATTCTTTTAATGATAATCAGTTGAGCGAAATCGTTATT

AGAGAGCAACAATACGGCTCTAAAACCCAAGCCATG---CTGTATTTTTGCTTTTCTATT

TTGGAGTTAAAAACCGCTACCCCCTTATTAAACAGAACCGCTACACTCAAAGAACAGGCT

CTTTTAACCATCCATAAAACCAACGCTCTTATGTTTTTAGAAATGCTTAAAATTTTTGGA

CTTTTAAGCCAAGCGCACCATAACGATGTGTTAAAGATTTTAGAAAAAATACTTCAAAAT

>B659-A1

GTGAGTTTGATTGAGATTGATAATAATAAAAAAGTGATTGAGGTTTCTATTCCTTTAACT

------------TCCATTTCAGGCAAAGTGCGTGTGAAAATCAGACATGCCTTTAGCGAT

TATGGCATTTCAACAGCGACCAGAAAAATCCCTTTTAGTTTAAAACATTATGTAGAGTGG

CAAATCGGTTATGATGTCCCCATTAAAGATAAAGAA---AAATTTGAGCTCACTACTTTA

AAAGATGAAAAATATCATTTTTTAGGGGCTAACAATAAAGTAAAAACCCTTTATGAATTG

AGTGAGATAATCTATTATGCTAAGCAATTAAATTTAATCAGT---------TTAGAAAAT

TTAGAAAATACTTTAAAATATTTAGAAAAACAAAAACAATTTATAGAAGATAATTTCACG

ATTACAAGAGAAAGATTTAGATCGCATCAATTTGGTGGCATGGATTTTGAACTTTCACGC

ATTTCTTATCCTTTACTCATTCATTCTTTTAATGATAATCAGTTGAGCGAAATCGTTATT

AGAGAGCAACAATACGGCTCTAAAACCCAAGCCATG---CTGTATTTTTGCTTTTCTATT

TTGGAGTTAAAAACCGCTACCCCCTTATTAAACAGAACCGCTACACTCAAAGAACAGGCT

CTTTTAACCATCCATAAAACCAACGCTCTTATGTTTTTAGAAATGCTTAAAATTTTTGGA

CTTTTAAGCCAAGCGCACCATAACGATGTGTTAAAGATTTTAGAAAAAATACTTCAAAAT

>G-Mx-2003-356

GTGAGTTTGATTAAGATTGATAATGATAAAAAAGTGATTGAGGTTTCTATTCCTTTAACT

------------TCCATTTCAGGCAAAGCGCGTGTGAAAATCAGACATGCCTTTAGCGAT

TATGGCATTTCAACAGCGACTAGAAAAATCCCTTTCAGTTTAAAGCATTATGTAGAGTGG

CAAATCGGTTATGATGTCCCCATTAAAGATAAAGAA---AAATTTGAGCTCACTACCCTA

AAAGATGAAAAATATCATTTTTTAGGGGCTAATAATAAAATAAAAACCCTTTATGAATTG

AGCGAAATGATTTATTACGCTAAGCGATTGGGTTTAATCAGT---------TTAGAAAAT

TTAGAAAATACTTTAAAATATTTAGAAAAACAAAAACAATTCATAGAAGATAGTTTTATG

ATCACAAGAGAAAGATTTAGATCGCATCAATTTGGTGGCATGGATTTTGAACTTTCACGC

ATTTCTTATCCTTTACTCATTCATTCTTTCAACGATAATCAATTAAGTGAAATCGTTATT

AGAGAGCAACAATACGGCTCTAAAACCCAAGCCATG---CTATATTTTTGCTTTTCTATT

CTGGAATTAAAAACCGCTACCCCTTTATTAAATAGAACGGCTGCACTCAAAGAACATGCC

CTTTTAACTATCCATGAAACTAACGCTCTTGTATTTTTAGAAATGCTTAAAATTTTTGGC

CTTTTAAGCCAAGCGCACCATAACGATGTGTTAAAGATTTTAGAAAAAATACTTCAAAAT

>C-Mx-2006-356

GTGAGTTTGATTAAGATTGATAATGATAAAAAAGTGATTGAGGTTTCTATTCCTTTAACT

------------TCCATTTCAGGCAAAGCGCGTGTGAAAATCAGACATGCCTTTAGCGAT

TATGGCATTTCAACAGCGACTAGAAAAATCCCTTTCAGTTTAAAGCATTATGTAGAGTGG

CAAATCGGTTATGATGTCCCCATTAAAGATAAAGAA---AAATTTGAGCTCACTACCCTA

AAAGATGAAAAATATCATTTTTTAGGGGCTAATAATAAAATAAAAACCCTTTATGAATTG

AGCGAAATGATTTATTACGCTAAGCGATTGGGTTTAATCAGT---------TTAGAAAAT

TTAGAAAATACTTTAAAATATTTAGAAAAACAAAAACAATTCATAGAAGATAGTTTTATG

ATCACAAGAGAAAGATTTAGATCGCATCAATTTGGTGGCATGGATTTTGAACTTTCACGC

ATTTCTTATCCTTTACTCATTCATTCTTTCAACGATAATCAATTAAGTGAAATCGTTATT

AGAGAGCAACAATACGGCTCTAAAACCCAAGCCATG---CTATATTTTTGCTTTTCTATT

CTGGAATTAAAAACCGCTACCCCTTTATTAAATAGAACGGCTGCACTCAAAGAACATGCC

CTTTTAACTATCCATGAAACTAACGCTCTTGTATTTTTAGAAATGCTTAAAATTTTTGGC

CTTTTAAGCCAAGCGCACCATAACGATGTGTTAAAGATTTTAGAAAAAATACTTCAAAAT

>HP11004

GTGAATTTGATTAAGATTGATAATAATAAAAAAGTAATTGAGATTTCTGTTCCTCTAACT

------------TCAATTTCAGGCAAAGCGCGTGTGAAAATCAGACATGCCTTTAGCGAT

TATGGCATTTCAACAGCGACTAGAAAAATCCCTTTTAGTTTAAAGCATTATGTAGAGTGG

CAAATCGGTTATGATGTCCCTATTAAAGATAAAGAA---AAATTTGAACTCACTACTTTA

AAAGATGAAAAATATCATTTTTTAGGGGCTAATAATAAAGTAAAGACTCTTTATGAATTG

AGCGAAATGATTTATTACGCTAAGCGATTGGGTTTAATCAGT---------TTAGAAAAT

TTAGAAAATACTTTAAAATATTTAGAAAAACAAAAACAATTTATAGAAGATAATTTTATG

ATTACAAGAGAAAGATTTAGATCGCATCAATTTGGTGGCATGGATTTTGAACTCTCACGC

ATTTCTTATCCTTTACTCATTCATTCTTTTAATGATAATCAGTTGAGTGAAATTGTTATT

AGGGAACAACAATACGGCTCTAAAACCCAAGCCATG---CTGTATTTTTGCTTTTCTATT

TTGGAGTTAAAAACCGCTACCCCCTTATTAAACAGAACGGCTACACTCAAAGAGCATGCT

CTTTTGATTATCCATAAAACCAACGCTCTTGTGTTTTTAGAAATGCTTAAAATTTTTGGA

CTTTTAAGCCAAGCGCACCATAACGATGTGTTAAAGATTTTAGAAAAAATACTTCAAAAT

>HP13026

GTGAGTTTGATTAAAGTTAATGATGATAAAAAAGTGATTGAGGTTTCTATTCCTTTAACT

------------TCCATTTCAGGCAAAGTTCGTGTGAAAATTAGGCATGCCTTTAGCGAT

TATGGCATTTCAACAGCGACTAGAAAAATCCCTTTCAGTTTAAAACATTATGTAGAGTGG

CAAATCGGTTATGATGTCCCTATTAAAGATAAAGAA---AAATTTGAGCTCACTACTTTA

AAAGATGAAAAATATCATTTTTTAGGGGCTAATAATAAAGTAAAAACCCTTTATGAATTG

AGCGAAATGATTTATTACGCTAAGCGATTGGGTTTAATCAGT---------TTAGAAAAT

TTAGAAAATACTTTAAAATATTTAGAAAAACAAAAACAATTTATAGAAGATAATTTTATG

ATTACAAGAGAAAGATTTAGATCGCATCAATTTGGAGGCATGGATTTTGAACTTTCACGC

ATTTCTTATCCTTTACTCATTCATTCTTTTAATGATAACCAATTGAGTGAAATCGTTATT

AGAGAGCAACAATATGGCTCTAAAACCCAAGCCATG---CTGTATTTTTGCTTTTCTATT

CTGGAATTAAAAACCGCTACCCCCTTATTAAATAGGACCGCTGCACTCAAAGAACATGCT

CTTTTAACTATCCATAAAACCAACGCTCCCATGTTTTTAGAAATGCTTAAAATTTTTGGA

CTTTTAAGCCAAGCACACCATAACGATGTGTTAAAGATTTTAGAAAAAATACTTGAAAAT

>LIM-002

GTGAGTTTGATTAAGATTGATAATAATAAAAAAGTAATTGAGGTTTCTATTCCTTTAACT

------------TCCATTTCAGGCAAAGTGCGTGTGAAAATCAGACATGCCTTTAGCGAT

TATGGCATTTCAACAGCGACTAGAAAAATCCCTTTTAGTTTAAAGCATTATGTAGAGTGG

CAAATCGGTTATGATGTCCCTATTAAAGATAAAGAA---AAATTTGAGCTCACTACTTTA

AAAGATGAAAAATATCATTTTTTAGGGGCTAATAATAAAGTAAAAACCCTTTATGAATTG

AGTGAAATAATTTATTACGCTAAGCGATTGGGTTTAATCGGT---------TTAGAAAAT

TTAGAAAATACTTTAAAATATTTAGAAAAACAAAAACAATTCATAGAATATAATTTTACG

ATTACAAGAGAAAGATTTAGATCGCATCAATTTGGGGGCATGGATTTTGAACTCTCACGC

ATTTCTTATCCCTTACTCATTCACTCTTTTAATGATAATCAGTTGAGCGAAATTGTTATT

AGAGAGCAACAATATGGCTCTAAAACCCAAGCCATG---CTGTATTTTTGCTTTTCTATT

TTGGAGTTAAAAACCGCTACCCCTTTATTAAATAGAACGGCTGCACTCAAAGAACATGCC

CTTTTAACTATCCATAAAACCAACGCTCTTATGTTTTTAGAAATGCTTAAAATTTTTGGA

CTTTTAAGCCAAGCGCACCATAACGATGTGTTAAAGATTTTAGAAAAAATACTTCAAAAT

>D1801434

GTGAGTTTGATTAAAATTAACCATGATGAAAAAGTGATTGAGATTTCTATTCCTTTAACT

------------TCAATTTCAGGCAAAGTGCGTGTGAAAATCAGACATGCCTTTAGCGAT

TATGGTGTTTCAACAGCGACTAGAAAAATCCCTTTTAGTTTAAAGCATTATGTAGAGTGG

CAGATCGGTTATGATGTCCCCATTAAAGATAAAGAA---AAATTTGAACTCACTACTTTA

AAAGATGAAAAATATCATTTTTTAGGAGCTAATAATAAAGTGAAAACCCTTTATGAATTG

AGCGAAATGATTTATTACGCTAAGCAATTAGGTTTAATCAGT---------TTAGAAAAT

TTAGAAAATACTTTAAAATATTTAGAAAAACAAAAACAATTTATAGAAGATAATTTTATG

ATTACAAGAGAAAGATTCAGATCGCATCAATTTGGTGGCATGGATTTTGAACTTTCACGC

ATTTCTTATCCTTTGCTCATTCATTCTTTTAATGATAATCAGTTGAGCGAAATTGTTATT

AGAGAACAACAATATGGTTCTAAAACCCAAGCCATG---CTGTATTTTTGCTTTTCTATT

TTGGAGTTAAAAACCGCTACTCCCTTATTAAACAGAACGGCTACGCTCAAAGAACATGCC

CTTTTAACTATCCATAAAACCAACGCTCTCATGTTTTTAGAAATGCTTAAAATTTTTGGA

CTTTTAAGCCAAGCACACCATAACGATGTGTTAAGGATTTTAGAAAAAATACTTCAAAAT

>2004-37

GTGAGTTTGATTAGGATTGATAATAATAAAAAAGTAATTGGGGTTTCTATTCCTTTAACT

------------TCCATTTCAGGCAAAGTGCGTGTGAAAATCAGACATGCCTTTAGCGAT

TATGGTATTTCAACAGCGACTAGAAAAATCCCTTTTAGCTTAAAACATTATGTAGAGTGG

CAAATCGGTTATGATGTCCCCATTAAAGATAAAGAA---AAATTTGAACTCACTACCCTA

AAAGATGAAAAATATCATTTTTTAGGGGCTAATAATAAAGTAAAAACCCTTTATGAATTG

AGTGAGATAATCTATTACGCTAAGCAATTAAATTTAATCAGT---------TTAGAAAAT

TTAGAAAATACTTTAAAATATTTAGAAAAACAAAAACAATTTATAGAAGATAATTTTATG

ATCACAAGAGAAAGATTTAGATTGCATCAATTTGGTGGCATGGATTTTGAGCTCTCACGC

ATTTCTTATCCTTTGCTCATTCATTCTTTTAATGATAATGAGTTGAGCGAAATCGTTATT

AGAGAACAACAATATGGCTCTAAAACCCAAGCCATG---CTGTATTTTTGCTTTTCTATT

TTGGAGTTAAAAACCGCTACCCCCTTATTAAATAGAACCGCTACGCTCAAAGAGCATGCC

CTTTTAACTATCCATAAAACCAACGCTCTTATGTTTTTAGAAATGCTTAAAATTTTTGGA

CTTTTAAGCCAAGCGCACCATAACGATGTGTTAAAGATTTTAGAAAAAATACTTCAAAAT

>MGms44

GTGAGTTTGATTAAGATTGATAATAATAAAAAAGTGATTGAGGTTTCTATTCCTTTAACT

------------TCCATTTCAGGCAAAGTGCGTGTGAAAATCAGACATGCCTTTAGCGAT

TATGGCATTTCAACAGCGACTAGAAAAATCCCTTTTAGTTTAAAGCATTATGTAGAGTGG

CAAATCGGTTATGATGTCCCCATTAAAGATAAAGAA---AAATTTGAACTCACTACCCTA

AAAGATGAAAAATATCATTTTTTAGGGGCTAATAATAAAAGAAAAACCCTTTATGAATTG

AGCGAAATAATTTATTACGCTAAGCGATTGGGTTTAATCAGT---------TTAGAAAAT

TTAGAAAATACTTTAAAATATTTAGAAAAACAAAAACAATTTATAGAAGATAATTTTATG

ATTACAAGAGAAAGATTTAGATCGCATCAATTTGGTGGCATGGATTTTGAACTTTCACGC

ATTTCTTATCCTTTGCTCATTCATTCTTTCAACGATAATCAATTAAGTGAAATCGTTATT

AGAGAGCAACAATATGGCTCTAAAACCCAAGCCATG---CTGTATTTTTGCTTTTCTATT

TTGGAATTAAAAACCGCTACCCCCTTATTAAATAGAACGGCTGCACTCAAAGAACATGCT

CTTTTGATTATCCATAAAACCAACGCTCTTGTGTTTTTAGAAATGCTTAAAATTTTTGGA

CTTTTAAGCCAAGCGCATCATAACGATGTGTTAAAGATTTTAGAAAAAATACTTGAAAAT

>PIMM-FM-UNAM-52A2

GTGAGTTTGATTAAGATTGATAATAATAAAAAAGTGATTGAGGTTTCTATTCCTTTAACT

------------TCCATTTCAGGCAAAGTGCGTGTGAAAATCAGACATGCCTTTAGCGAT

TATGGCATTTCAACAGCGACTAGAAAAATCCCTTTTAGTTTAAAGCATTATGTAGAGTGG

CAAATCGGTTATGATGTCCCCATTAAAGATAAAGAA---AAATTTGAACTCACTACCCTA

AAAGATGAAAAATATCATTTTTTAGGGGCTAATAATAAAAGAAAAACCCTTTATGAATTG

AGCGAAATAATTTATTACGCTAAGCGATTGGGTTTAATCAGT---------TTAGAAAAT

TTAGAAAATACTTTAAAATATTTAGAAAAACAAAAACAATTTATAGAAGATAATTTTATG

ATTACAAGAGAAAGATTTAGATCGCATCAATTTGGTGGCATGGATTTTGAACTTTCACGC

ATTTCTTATCCTTTGCTCATTCATTCTTTCAACGATAATCAATTAAGTGAAATCGTTATT

AGAGAGCAACAATATGGCTCTAAAACCCAAGCCATG---CTGTATTTTTGCTTTTCTATT

TTGGAATTAAAAACCGCTACCCCCTTATTAAATAGAACGGCTGCACTCAAAGAACATGCT

CTTTTGATTATCCATAAAACCAACGCTCTTGTGTTTTTAGAAATGCTTAAAATTTTTGGA

CTTTTAAGCCAAGCGCATCATAACGATGTGTTAAAGATTTTAGAAAAAATACTTGAAAAT

>1198/04

GTGAGTTTGATTGAGATTGATAATAATAAAAAAGTAATTGAGATTTCTATTCCTTTAACT

------------TCAATTTCAGGCAAAGTGCGTGTGAAAATCAGACATGCCTTTAGCGAT

TATGGTACTTCAACAGCGACTAGAAAAATCCCTTTTAGCTTAAAACATTATGTAGAGTGG

CAAATCGGTTATGATGTCCCCATTAAAGATAAAGAA---AAATTTGAGCTCACTACCCTA

AAAGATGAAAAATATCATTTTTTAGGGGCTAATAATAAAGTAAAAACCCTTTATGAATTG

AGTGAGATAATCTATTACGCTAAGCAATTAAATTTAATCAGT---------TTAGAAAAT

TTAGAAAATACTTTAAAATATTTAGAAAAACAAAAACAATTTATAGAAGATAATTTCACG

ATTACAAGAGAAAGATTTAGATCGCATCAATTTGGTGGCATGGATTTTGAACTCTCACGC

ATTTCTTATCCTTTACTCATTCATTCTTTTAATGATAATCAATTGAGTGAAATCGTTATT

AGAGAGCAACAATATGGCTCTAAAACCCAAGCCATG---CTGTATTTTTGCTTTTCTATT

TTGGAGTTAAAAACCACTACCCCCTTATTAAATAGAACGGCTGCACTCAAAGAACATGCC

CTTTTAACTATCCATAAAACCAACGCTCTTATGTTTTTAGAAATGCTTAAAATTTTTGGA

CTTTTAAGCCAAGCGCACCATAACGATGTGTTAAAGATTTTAGAAAAAATACTTCAAAAT

>B400

GTGAGTTTGATTAGGATTGATAATAATAAAAAAGTAATTGGGGTTTCTATTCCTTTAACT

------------TCCATTTCAGGCAAAGTGCGTGTGAAAATCAGGCATGCCTTTAGCGAT

TATGGTATTTCAACAGCGACCAGAAAAATCCCTTTTAGTTTAAAACATTATGTAGAGTGG

CAAATCGGTTATGATGTCCCCATTAAAGATAAAGAA---AAATTTGAACTCACTACTTTA

AAAGATAAAAAATATCATTTTTTAGGGGCTAATAATAAAGTAAAAACCCTTTATGAATTG

AGCGAAATAATTTATTACGCTAAGCGATTGGGTTTAATCAGT---------TTAGAAAAT

TTAGAAAATACTTTAAAATATTTAGAAAAGCAAAAACAATTCATAGAAGATAATTTTATG

ATTACAAGAGAAAGATTTAGATCGCATCAATTTGGTGGCATGGATTTTGAACTTTCACGC

ATTTCTTATCTTTTGCTCATTCATTCTTTTAATGATAATGAGTTGAGCGAAATCGTTATT

AGAGAACAACAATATGGCTCTAAAACCCAAGCCATG---CTGTATTTTTGCTTTTCTATT

TTGGAGTTAAAAACCGCTACCCCTTTATTAAATAGAACGGCTGCACTCAAAGAACATGCT

CTTTTAACTATCCATAAAACCAACGCTCTTATGTTTTTAGAAATGCTTAAAATTTTTGGA

CTTTTAAGCCAAGCGCACCATAACGATGTGTTAAAGATTTTAGAAAAAATACTTCAAAAT

>ZH51

GTGAGTTTGATTAGGATTGATAATAATAAAAAAGTAATTGGGGTTTCTATTCCTTTAACT

------------TCAATTTCAGGCAAAGTGCGTGTGAAAATCAGACATGCCTTTAGCGAT

TATGGCATTTCAACAGCGACTAGAAAAATCCCTTTTAGTTTAAAGCATTATGTAGAGTGG

CAAATCGGTTATGATGTCCCCATTAAAGATAAAGAA---AAATTTAAACTCACTACTTTA

AAAGATGAAAAATATCATTTTTTAGGGGCTAATAATAAAGTAAAAACCCTTTATGAATTG

AGTGAGATAATCTATTACGCTAAGCAATTAAATTTAATCAGT---------TTAGAAAAT

TTAGAAAATACTTTAAAATATTTAGAAAAACAAAAACAATTTATAGAAGATAATTTTATG

ATTACAAGAGAAAGATTTAGATTACATCAATTTGGTGGCATGGATTTTGAACTCTCACGC

ATTTCTTACCCTTTGCTCATTCATTCTTTTAATGATAATCAATTGAGTGAAATCGTTATT

AGAGAACAACAATATGGCTCTAAAACCCAAGCCATG---CTGTATTTTTGCTTTTCTATT

TTGGAGTTAAAAACCGCTACTCCCTTATTAAATAGAACGGCTGCACTCAAAGAACATGCT

CTTTTGATTATCCATAAAACCAACGCTCTCATGTTTTTAGAAATGCTTAAAATTTTTGGA

CTTTTAAGCCAAGCACACCATAACGATGTGTTAAAGATTTTAGAAAAAATACTTCAAAAT

>ZH94

GTGAGTTTGATTAAAGTTAGTGGTGATAAAAAAGCGATTGAGGTTTCTATTCCTTTAACT

------------TCAATTTCAGGCAAAGTGCGTGTGAAAATCAGACATGCCTTTAGCGAT

TATGGTATTTCAACAGCGACCAGAAAAATCCCTTTTAGTTTAAAGCATTATGTAGAGTGG

CAGATCGGTTATGATGTCCCCATTAAAGATAAAGAA---AAATTTGAACTCACTACTTTA

AAAGATGAAAAATATCATTTTTTAGGGGCTAATAATAAAGTAAAGACTCTTTATGAATTG

AGCGAAATGATTTATTACGCTAAGCAATTAGGTTTAATCAGT---------TTAGAAAAT

TTAGAAAATACTTTAAAATATTTAGAAAAACAAAAACAATTTATAGAAGATAATTTTATG

ATTACAAGAGAAAGATTTAGATCGCATCAATTTGGTGGCATGGATTTTGAACTTTCACGC

ATCTCTTATCCTTTACTCATTCATTCTTTTAATGATAATCAGTTGAGCGAAATCGTTATT

AGAGAGCAACAATATGGCTCTAAAACCCAAGCCATG---CTGTATTTTTGCTTTTCTATT

TTGGAGTTAAAAACCGCTACCCCCTTATTAAACAGAACGGCTGCACTCAAAGAACATGCC

CTTTTAACTATCCATAAAACCAACGCTCTTATGTTTTTAGAAATGCTTAAAATTTTTGGA

CTTTTAAGCCAAGTGCACCATAGCGATGTGTTAAAGATTTTAGAAAAAATACTTCAAAAT

>38185

GTGAATTTGATTAAGATTGATAATAATAAAAAAGTAATTGAGATTTCTGTTCCTTTAACT

------------TCAATTTCAGGCAAAGTGCGTGTGAAAATCAGACATGCCTTTAGCGAT

TATGGCATTTCAACAGCGACTAGAAAAATCCCTTTTAGTTTAAAGCATTATGTAGAGTGG

CAAATCGGTTATGATGTCCCCATTAAAGATAAAGAA---AAATTTAAACTCACTACTTTA

AAAGATGAAAAATATCATTTTTTAGGGGCTAATAATAAAGTAAAGACTCTTTATGAATTG

AGTGAGATAATCTATTACGCTAAGCAATTAAATTTAATCAGT---------TTAGAAAAT

TTAGAAAATACTTTAAAATATTTAGAAAAACAAAAACAATTTATAGAAGATAATTTTATG

ATTACAAGAGAAAGATTTAGATCGCATCAATTTGGTGGCATGGATTTTGAACTCTCACGC

ATTTCTTATCCTTTGCTCATTCATTCTTTTAATGATAATCAGTTGAGCGAAATCGTTATT

AGAGAACAACAATATGGCTCTAAAACCCAAGCCATG---CTGTATTTTTGCTTTTCTATT

TTGGAGTTAAAAACCGCTACCCCTTTATTAAATAGAACGGCTGCACTCAAAGAACATGCC

CTTTTAACTATCCATAAAACCAACGCTCTTGTGTTTTTAGAAATGCTTAAAATTTTTGGA

CTTTTAAGCCAAGCGCACCATAGCGATGTGTTAAAGATTTTAGAAAAAATACTTCAAAAT

>KH0198

GTGAGTTTGATTAGGATTGATAATAATAAAAAAGTAATTGGGGTTTCTATTCCTTTAACT

------------TCAATTTCAGGCAAAGTGCGTGTGAAAATCAGACATGCCTTTAGCGAT

TATGGTATTTCAACAGCGACCAGAAAAATCCCTTTTAGCTTAAAACATTATGTAGAGTGG

CAAATCGGTTATGATGTCCCCATTAAAGATAAAGAA---AAATTTGAGCTCACTACCCTA

AAAGATGAAAAATATCATTTTTTAGGGGCTAATAATAAAGTGAAAACTCTTTATGAATTA

AGCGAAATGATTTATTACGCTAAGCAATTAGGTTTAATTGGT---------TTAGAAAAT

TTAGAAAATACTTTAAAATATTTAGAAAAACAAAAACAATTTATAGAAGATAATTTCACG

ATTACAAGAGAAAGATTTAGATCGCATCAATTTGGTGGCATGGATTTTGAACTCTCACGC

ATTTCTTATCCCTTACTCATTCATTCTTTTAATGATAATCAGTTGAGCGAAATTGTTATT

AGAGAGCAACAATATGGTTCTAAAACCCAAGCCATG---CTGTATTTTTGCTTTTCTATT

TTGGAATTAAAAACCGCTACTCCCTTATTAAATAGAACGGCTGCACTCAAAGAACATGCC

CTTTTAACTATCCATAAAACCAACGCTCTTATGTTTTTAGAAATGCTTAAAATTTCTGGA

CTTTTAAGCCAAGTGCACCATAGCGATGTGTTAAAGATTTTAGAAAAAATACTTCAAAAT

>HP15033

GTGAGTTTGATTAGGATTGATAATAATAAAAAAGTAATTGGGGTTTCTATTCCTTTAACT

------------TCAATTTCAGGCAAAGTGCGTGTGAAAATCAGACATGCCTTTAGCGAT

TATGGTATTTCAACAGCGACCAGAAAAATCCCTTTTAGCTTAAAACATTATGTAGAGTGG

CAAATCGGTTATGATGTCCCCATTAAAGATAAAGAA---AAATTTGAGCTCACTACCCTA

AAAGATGAAAAATATCATTTTTTAGGGGCTAATAATAAAGTGAAAACTCTTTATGAATTA

AGCGAAATGATTTATTACGCTAAGCAATTAGGTTTAATTGGT---------TTAGAAAAT

TTAGAAAATACTTTAAAATATTTAGAAAAACAAAAACAATTTATAGAAGATAATTTCACG

ATTACAAGAGAAAGATTTAGATCGCATCAATTTGGTGGCATGGATTTTGAACTCTCACGC

ATTTCTTATCCCTTACTCATTCATTCTTTTAATGATAATCAGTTGAGCGAAATTGTTATT

AGAGAGCAACAATATGGTTCTAAAACCCAAGCCATG---CTGTATTTTTGCTTTTCTATT

TTGGAATTAAAAACCGCTACTCCCTTATTAAATAGAACGGCTGCACTCAAAGAACATGCC

CTTTTAACTATCCATAAAACCAACGCTCTTATGTTTTTAGAAATGCTTAAAATTTCTGGA

CTTTTAAGCCAAGTGCACCATAGCGATGTGTTAAAGATTTTAGAAAAAATACTTCAAAAT

>ZH54

GTGAGTTTGATTAGGATTGATAATAATAAAAAAGTAATTGGGGTTTCTATTCCTTTAACT

------------TCAATTTCAGGCAAAGTGCGTGTGAAAATCAGACATGCCTTTAGCGAT

TATGGTATTTCAACAGCGACTAGAAAAATCCCTTTTAGTTTAAAACATTATATAGAGTGG

CAAATCGGTTATGATGCCCCCATTAAAGATAAAGAA---AAATTTGAACTCACTACTTTA

AAAGATGAAAAATATCATTTTTTAGGGGCTAATAATAAAGTGAAAACTCTTTATGAATTA

AGCGAAATGATTTATTACGCTAAGCAATTAGATTTAATCAGT---------TTAGAAAAT

TTAGAAAATACTTTAAAATATTTAGAAAAACAAAAACAATTTATAGAAGATAATTTTATG

ATTACAAGAGAAAGATTTAGATTGCATCAATTTGGTGGCATGGATTTTGAACTCTCACGC

ATTTCTTATCCTTTACTCATTCATTCTTTTAATGATAATCAATTGAGCGAAATTATTATT

AGAGAGCAACAATACGGCTCTAAAACCCAAGCCATG---CTGTATTTTTGCTTTTCTATT

TTGGAGTTAAAAACCGCTACCCCTTTATTAAATAGAACGGCTGCACTCAAAGAACATGCC

CTTTTAACCATCCATAAAACCAACGCTCTCATGTTTTTAGAAATGCTTAAAATTTTTGGA

CTTTTAAGCCAAGTGCACCATAACGATGTGTTAAAGATTTTAGAAAAAATACTTCAAAAT

>132A

GTGAGTTTGATTAAAGTTGACTATGATAAAAAAGTGATTGAGGTTTCTATTCCTTTAACT

------------TCAATTTCAGGCAAAGTGCGTGTGAAAATCAGACATGCCTTTAGCGAT

TATGGTATTTCAACAGCGACTAGAAAAATCCCTTTTAGTTTAAAACATTATATAGAGTGG

CAGATCGGTTATGATGTCCCCATTAAAGATAAAGAA---AAATTTGAACTCACTGCTTTA

AAAGATAAAAAATACCATTTTTTAGGGGCTAATAATAAAGTAAAAACTCTTTATGAATTG

AGCGAAATGATTTATTACGCTAAGCAATTAGGTTTAATCAGT---------TTAGAAAAT

TTAGAAAATACTTTAAAATATTTAGAAAAACAAAAACAATTTATAGAAGATAATTTTATG

ATTACAAGAGAAAGATTTAGATCTCATCAATTTGGCGGCATGGATTTTGAACTTTCACGC

ATTTCTTATCCCTTACTCATTCATTCTTTTAATGATAATCAGTTGAGCGAAATAGTTATT

AGAGAACAACAATATGGCTCTAAAACCCAAGCCATG---CTGTATTTTTGCTTTTCTATT

TTGGAGTTAAAAACTGCTACCCCCTTATTAAATAGAACCGCTGCACTCAAAGAACATGCT

TTTTTAACCATCCATAAAACCAACGCTCTCATGTTTTTAGAAATGCTTAAAATTTTTGGA

CTTTTAAGCCAAGCGCACCATAACGATGTGGTGAAGATTTTAGAAAAAATACTTCAAAAT

>132

GTGAGTTTGATTAAAGTTGACTATGATAAAAAAGTGATTGAGGTTTCTATTCCTTTAACT

------------TCAATTTCAGGCAAAGTGCGTGTGAAAATCAGACATGCCTTTAGCGAT

TATGGTATTTCAACAGCGACTAGAAAAATCCCTTTTAGTTTAAAACATTATATAGAGTGG

CAGATCGGTTATGATGTCCCCATTAAAGATAAAGAA---AAATTTGAACTCACTGCTTTA

AAAGATAAAAAATACCATTTTTTAGGGGCTAATAATAAAGTAAAAACTCTTTATGAATTG

AGCGAAATGATTTATTACGCTAAGCAATTAGGTTTAATCAGT---------TTAGAAAAT

TTAGAAAATACCTTAAAATATTTAGAAAAACAAAAACAATTTATAGAAGATAATTTTATG

ATTACAAGAGAAAGATTTAGATCTCATCAATTTGGCGGCATGGATTTTGAACTTTCACGC

ATTTCTTATCCCTTACTCATTCATTCTTTTAATGATAATCAGTTGAGCGAAATAGTTATT

AGAGAACAACAATATGGCTCTAAAACCCAAGCCATG---CTGTATTTTTGCTTTTCTATT

TTGGAGTTAAAAACTGCTACCCCCTTATTAAATAGAACCGCTGCACTCAAAGAACATGCT

TTTTTAACCATCCATAAAACCAACGCTCTCATGTTTTTAGAAATGCTTAAAATTTTTGGA

CTTTTAAGCCAAGCGCACCATAACGATGTGGTGAAGATTTTAGAAAAAATACTTCAAAAT

>KH0071

GTGAGTTTGATTAGGATTGATGATAGTAAAAAAGTAATTGAGGTTTCTATTCCTTTAACT

------------TCAATTTCAGGCAAAGTGCGTGTGAAAATCAGGCATGCCTTTAGCGAT

TATGGTATTTCAACAGCGACTAGAAAAATCCCTTTTAGTTTAAAGCATTATGTAGAGTGG

CAAATCGGTTATGATGTCCCCATTGAAGATAAAGAA---AAATTTGAGCTCACTACCCTA

AAAGATGAAAAATATCATTTTTTAGGGGCTAATAATAAAGTAAAAACTCTTTATGAATTG

AGTGAAATAATCTATTACGCTAAGCAATTAAATTTAATCAGT---------TTAGAAAAT

TTAGAAAATACTTTAAAATATTTAGAAAAACAAAAACAATTTATAGAAGATAATTTCACG

ATTACAAGAGAAAGATTTAGATCGCATCAATTTGGGGGCATGGATTTTGAACTTTCACGC

ATTTCTTATCCCTTACTCATTCATTCTTTTAATGATAATCAATTGAGCGAAATCGTTATT

AGAGAGCAACAATACGGCTCTAAAACCCAAGCCATG---CTGTATTTTTGCTTTTCTATT

TTGGAATTAAAAACCGCTACTCCCTTATTAAATAGAACGGCTGCACTCAAAGAACATGCC

CTTTTAACTATCCATAAAACCAACGCTCTTGTGTTTTTAGAAATGCTTAAAATTTTTGGA

CTTTTAAGCCAAGCGCACCATAACGATGTGTTAAAGATTTTAGAAAAAATACTTCAAAAT

>KH0025

GTGAGTTTGATTAAAGTTAATGATGATAAAAAAGTAATTGAGGTTTCTATTCCTTTAACT

------------TCAATTTCAGGCAAAGTGCGTGTGAAAATCAGACATGCCTTTAGCGAT

TATGGTATTTCAACAGCGACTAGAAAAATCCCTTTTAGTTTAAAACATTATGTAGAGTGG

CAAATCGGTTATGATGTCCCCATTAAAGATAAGGAA---AAATTTGAACTCACTGCTTTA

AAAGATGAAAAATACCATTTTTTAGGAGCTAATGATAAAGTGAAAACTCTTTATGAATTG

AGCGAAACGATTTATTACGCTAAGCAATTAGGTTTAATCAGT---------TTAGAAAAT

TTAGAAAATACTTTAAAATATTTAGAAAAACAAAAACAATTTATAGAAGATAATTTTATG

ATTACAAGAGAAAGATTTAGAACGCATCAATTTGGTGGCATGGATTTTGAACTTTCACGC

ATTTCTTATCCTTTACTCATTCATTCTTTTGATGATAATCAGTTGAGTGAAATTGTTATT

AGAGAGCAACAATACGACTCTAAAACCCAAGCCATG---CTGTATTTTTGCTTTTCTATT

TTGGAATTAAAAACCGCTACTCCCTTATTAAATAGAACGGCTGCACTCAAAGAACATGCC

CTTTTAACTATCCATAAAACCAACGCTCTTGTGTTTTTAGAAATGCTTAAAATTTTTGGA

CTTTTAAGCCAAGCGCACCATAACGATGTGTTAAAGATTTTAGAAAAAATACTTCAAAAT

>456

GTGAGTTTGATTAAAGTTAGTGGTGATAAAAAAGTGATTGAGGTTTCTATTCCTTTAACT

------------TCAATTTCAGGAAAAGTGCGTGTGAAAATCAGACATGCCTTTAGCGAT

TATGGTGTTTCAACAGCGACTAGAAAAATCCCTTTTAGTTTAAAACATTATGTAGAGTGG

CAGATTGGTTATGATGTCCCCATTAAAGATAAAGAA---AAATTTGAACTCACTACTTTA

AAAGATGAAAAATATCATTTTTTAGGGGCTAATAATAAAGTGAAAACTCTTTATGAATTG

AGCGAAATGATTTATTACGCTAAGCAATTAGGTTTAATCAGT---------TTAGAAAAT

TTAGAAAATACTTTAAAAGATTTAGAAAAACAAAAACAATTTATAGAAGATAATTTTATG

ATTACAAGAGAAAGATTTAGATTGCATCAATTTGGTGGCATGGATTTTGAACTCTCACGC

ATTTCTTATCCTTTACTCATTCATTCTTTTGATGATAATCAGTTGAGCGAAATCGTTATT

AGAGAACAACAATATGGCTCTAAAACCCAAGCCATG---CTGTATTTTTGCTTTTCTATT

TTGGAATTAAAAACCGCTACCCCCTTATTAAATAGAACGGCTGCTCTCAAAGAACATGCC

CTTTTAACTATCCATAAAACCAACGCTCTCATGTTTTTAGAAATGCTTAAAATTTTTGGA

CTTTTAAGCCAAGCGCACCATAACGATGTGTTAAAGATTTTAGAAAAAATACTTCAAAAT

>ZH09

GTGAGTTTGATTAAAATTAACCATGATGAAAAAGTGATTGAGGTTTCTATTCCTTTAACT

------------TCAAATTCAGGCAAAGTGCGTGTGAAAATCAGACATGCCTTTAGCGAT

TATGGTATTTCAACAGCGACTAGAAAAATCCCTTTTAGTTTAAAACATTATGTAGAGTGG

CAAATCGGTTATGATGTCCCTATTAAAGATAAAGAA---AAATTTGAACTCACTACTTTA

AAAGATGAAAAATATCATTTTTTAGGGGCTAATAATAAAGTAAAAACTCTTTATGAATTA

AGTGAAATGATTTATTACGCTAAGCAATTAGGTTTAATCAGT---------TTAGAAAAT

TTAGAAAATACTTTAAAATATTTAGAAAAACAAAAACAATTTATAGAAGATAATTTTATG

ATCACAAGAGAAAGATTTAGATCGCATCAATTTGGTGGCATGGATTTTGAACTCTCACGC

ATTTCTTATCCTTTACTCATTCATTCTTTTAATGATAATCAGTTGAGCGAAATTGTTATT

AGAGAGCAACAATACGGCTCTAAAACCCAAGCCATG---CTGTATTTTTGCTTTTCTATT

TTGGAGTTAAAAACCGCTACCCCCTTATTAAACAGAACGGCTGCACTTAAAGAACATGCC

CTTTTAACTATCCATAAAACCAACGCTCTCATGTTTTTAGAAATGCTTAAAATTTTTGGA

CTTTTAAGCCAAGCGCACCATAGTGATGTGTTAAAGATTTTAGAAAAAATACTTCAAAAT

>52

GTGAGTTTGATTAAAGTTAGTAGTGATAAAAAAGCGATTGAGGTTTCTATTCCTTTAACT

------------TCAATTTCAGGCAAAGTGCGTGTGAAAATCAGACATGCCTTTAGCGAT

TATGGTGTTTCAACAGCGACTAGAAAAATCCCTTTTAGTTTAAAACATTATGTAGAGTGG

CAGATCGGTTATGATGTCCCTATTAAAGATAAAGAA---AAATTTGAACTCACTACTTTA

AAAGATGAAAAATATCATTTTTTAGGGGCTAATAATAAAGTAAAAACTCTTTATGAATTA

AGCGAAATGATTTATTACGCTAAGCAGTTAGGTTTAATCAGT---------TTAGAAAAT

TTAGAAAATACTTTAAAATATTTAGAAAAACAAAAACAATTTATAGAAGATAATTTTATG

ATCACAAGAGAAAGATTTAGATCCCATCAATTTGGGGGCATGGATTTTGAACTCTCACGC

ATTTCTTATCCTTTGCTCATTCATTCTTTTAATGATAATCAGTTGAGCGAAATTGTTATT

AGGGAACAACAATATGGCTCTAAAACCCAAGCCATG---CTGTATTTTTGCTTTTCTATT

TTGGAGTTAAAAACCGCTACCCCCTTATTAAATAGAACGGCTATGCTCAAAGAGCATGCT

CTTTTGATTATCCATAAAACCAACGCTCTCATGTTTTTAGAAATGCTTAAAATTTTTGGA

CTTTTAAGCCAAGCGCACCATAACGATGTGTTAAAGATTTTAGAAAAAATACTTCAAAAT

>ECF139-B065

GTGAATTTGATTAAGATTGATAATAATAAAAAAGTAATTGAGATTTCTGTTCCTTTAACT

------------TCAATTTCAGGCAAAGTGCGTGTGAAAATCAGACATGCCTTTAGCGAT

TATGGCATTTCAACAGCGACTAGAAAAATCCCTTTTAGTTTAAAGCATTATGTAGAGTGG

CAAATCGGTTATGATGTCCCCATTAAAGATAAAGAA---AAATTTAAACTCACTACTTTA

AAAGATGAAAAATATCATTTTTTAGGGGCTAATAATAAAGTAAAGACTCTTTATGAATTG

AGTGAGATAATCTACTACGCTAAGCAATTAAATTTAATCAGT---------TTAGAAAAT

TTAGAAAATACTTTAAAATATTTAGAAAAACAAAAACAATTTATAGAAGATAATTTCACG

ATTACAAGAGAAAGATTTAGATCGCATCAATTTGGGGGCATGGATTTTGAACTTTCACGC

ATTTCTTATCCCTTACTCATTCATTCTTTTAATGATAATCAATTGAGCGAAATCGTTATT

AGAGAGCAACAATACGGCTCTAAAACCCAAGCCATG---CTGTATTTTTGCTTTTCTATT

TTGGAGTTAAAAACCGCTACCCCCTTATTAAATAGAACCGCTACGCTCAAAGAGCATGCC

CTTTTAACTATCCATAAAACCAACGCTCTTATGTTTTTAGAAATGCTTAAAATTTTTGGA

CTTTTAAGCCAAGCGCACCATAACGATGTGTTAAAGATTTTAGAAAAAATACTTCAAAAT

>3120

GTGAGTTTGATTAGGATTGATAATAATAAAAAAGTAATTGGGGTTTCTATTCCTTTAACT

------------TCAATTTCAGGCAAAGTGCGTGTGAAAATCAGACATGCCTTTAGCGAT

TATGGTATTTCAACAGCGACTAGAAAAATCCCTTTTAGCTTAAAACATTATGTAGAGTGG

CAAATCGGTTATGATGTCCCCATTAAAGATAAAGAA---AAATTTGAGCTCACTACCCTA

AAAGATGAAAAATATCATTTTTTAGGGGCTAATAATAAAGTAAAAACCCTTTATGAATTG

AGTGAGATAATCTATTACGCTAAGCAATTAAATTTAATCAGT---------TTAGAAAAT

TTAGAAAATACTTTAAAATATTTAGAAAAACAAAAACAATTCATAGAAGATAGTTTTATG

ATCACAAGAGAAAGATTTAGATCGCATCAATTTGGGGGCATGGATTTTGAACTTTCACGC

ATCTCTTATCCCTTACTCATTCATTCTTTTAATGATAATCAGTTGAGTGAAATCGTTATT

AGAGAGCAACAATACGGCTCTAAAGTCCAAGCCATG---CTGTATTTTTGCTTTTCTATT

TTGGAATTAAAAACCGCTACCCCTTTATTAAATAGAACCGCTACGCTCAAAGAGCATGCC

CTTTTAACTATCCATAAAACCAACGCTCTTATGTTTTTAGAAATGCTTAAAATTTTTGGA

CTTTTAAGCCAAGTGCACCATAACGATGTGTTAAAGATTTTAGAAAAAATACTTCAAAAT

>C-Mx-2011-152

GTGAGTTTGATTAAAGTTAATGATGATAAAAAAGTAATTGAGGTTTCTATTCCTTTAACT

------------TCCATTTCAGGCAAAGTTCGTGTGAAAATCAGACATGCCTTTAGCGAT

TATGGCATTTCAACAGCGACTAGAAAAATCCCTTTCAGTTTAAAGCATTATGTAGAGTGG

CAAATCGGTTATGATGTCCCCATTAAAGATAAAGAA---AAATTTGAACTCACTACCCTA

AAAGATGAAAAATATCATTTTTTAGGGGCTAATAATAAAATAAAAACCCTTTATGAATTG

AGCGAAATGATTTATTACGCTAAGCGATTGGGTTTAATCAGT---------TTAGAAAAT

TTAGAAAATACTTTAAAATATTTAGAAAAACAAAAACAATTCATAGAAGATAGTTTTATG

ATCACAAGAGAAAGATTTAGATCGCATCAATTTGGTGGCATGGATTTTGAACTTTCACGC

ATCTCTTACCCTTTACTCATTCATTCTTTTAATGATAATCAGTTGAGTGAAATCGTTATT

AGAGAGCAACAATACGGCTCTAAAACCCAAGCCATG---CTGTATTTTTGCTTTTCTATT

CTGGAATTAAAAACCGCTACACCCTTATTAAATAGAACGGCTGCACTCAAAGAACACGCC

CTTTTAACTATCCATAAAACCAACGCTCCCATGTTTTTAGAAATGCTTAAAATTTTTGGA

CTTTTAAGCCAAGCGCACCATAACGATGTGTTAAAGATTTTAGAAAAAATACTTCAAAAT

>C-Mx-2008-31

GTGAGTTTGATTAAAGTTAATGATGATAAAAAAGTAATTGAGGTTTCTATTCCTTTAACT

------------TCCATTTCAGGCAAAGTTCGTGTGAAAATCAGACATGCCTTTAGCGAT

TATGGCATTTCAACAGCGACTAGAAAAATCCCTTTCAGTTTAAAGCATTATGTAGAGTGG

CAAATCGGTTATGATGTCCCCATTAAAGATAAAGAA---AAATTTGAACTCACTACCCTA

AAAGATGAAAAATATCATTTTTTAGGGGCTAATAATAAAATAAAAACCCTTTATGAATTG

AGCGAAATGATTTATTACGCTAAGCGATTGGGTTTAATCAGT---------TTAGAAAAT

TTAGAAAATACTTTAAAATATTTAGAAAAACAAAAACAATTCATAGAAGATAGTTTTATG

ATCACAAGAGAAAGATTTAGATCGCATCAATTTGGTGGCATGGATTTTGAACTTTCACGC

ATCTCTTACCCTTTACTCATTCATTCTTTTAATGATAATCAGTTGAGTGAAATCGTTATT

AGAGAGCAACAATACGGCTCTAAAACCCAAGCCATG---CTGTATTTTTGCTTTTCTATT

CTGGAATTAAAAACCGCTACACCCTTATTAAATAGAACGGCTGCACTCAAAGAACACGCC

CTTTTAACTATCCATAAAACCAACGCTCCCATGTTTTTAGAAATGCTTAAAATTTTTGGA

CTTTTAAGCCAAGCGCACCATAACGATGTGTTAAAGATTTTAGAAAAAATACTTCAAAAT

>HP15012

GTGAGTTTGATTAGGATTGATGATAGTAAAAAAGCGATTGAGGTTTCCATTCCTTTAACT

------------TTAATTTCAGGCAAAGTGCGTGTGAAAATCAGGCATGCCTTTAGCGAT

TATGGTATTTCAACAGCGACTAGAAAAATCCCTTTTAGTTTAAAACATTATGTAGAGTGG

CAGATCGGTTATGATGTCCCCATTAAAGATAAAGAA---AAATTTGAACTCACTACTTTA

AAAGATGAAAAATATCATTTTTTAGGAGCTAATAATAAAGTAAAAACTCTTTATGAATTG

AGTGAAATGATTTATTACGCTAAGCAATTAGGTTTAATCAGT---------TTAGAAAAT

TTAGAAAATACTTTAAAATATTTAGAAAAACAAAAACAATTTATAGAAGATAATTTTATG

ATTACAAGAGAAAGATTTAGAACGCATCAATTTGGTGGCATGGATTTTGAACTCTCACGC

ATTTCTTATCCTTTACTCATTCATTCTTTTGATGATAATCAATTGAGCGAAATTGTTATT

AGAGAACAACAATACGGCTCTAAAACCCAAGCCATG---CTGTATTTTTGCTTTTCTATT

TTGGAGTTAAAAACCGCTACCCCCTTATTAAACAGAACCGCTACGCTCAAAGAGCATGCT

CTTTTGATTATCCATAAAACCAACGCTCCCATGTTTTTAGAAATGCTTAAAATTTTTGGA

CTTTTAAGCCAAGCACACCATAACGATGTGTTAAAGATTTTAGAAAAAATACTTCAAAAT

>ZH78

GTGAGTTTGATTAAGATTGATAATGATAAAAAAGTGATTGAGGTTTCTATTCCTTTAACT

------------TCCATTTCAGGCAAAGTGCGTGTGAAAATCAGACATGCTTTTAGCGAT

TATGGCATTTCAACAGCGACCAGAAAAATCCCTTTTAGTTTAAAACATTATATAGAGTGG

CAGATCGGTTATGATGTCCCCATTAAAGATAAAGAA---AAATTGGAACTCACTACTTTA

AAAGATGAAAAATATCATTTTTTAGGGGCTAATAATAAAGTAAAGACTCTTTATGAATTG

AGCGAAATGATTGATTACGCTAAGCAATTAAATTTAATCAGT---------TTAGAAAAT

TTAGAAAATACTTTAAAATATTTAGAAAAACAAAAACAATTTATAGAAGATAATTTTATG

ATCACAAGAGAAAGATTTAGATCGCATCAATTTGGTGGCATGGATTTTGAACTCTCACGC

ATTTCTTATCCTTTGCTCATTCATTCTTTTAATGATAATCAATTGAGCGAAATCGTTATT

AGAGAGCAACAATATGGCTCTAAAACTCAAGCCATG---CTGTATTTTTGCTTTTCTATT

TTGGAGTTAAAAACCGCTACCCCCTTATTAAACAGAACGGCTGCACTTAAAGAACATGCC

CTTTTAACTATCCATAAAACCAACGCTCTCATGTTTTTAGAAATGCTTAAAATTTTTGGA

CTTTTAAGCCAAGCGCACCATAGTGATGTGTTAAAGATTTTAGAAAAAATACTTCAAAAT

>G-Mx-2010-13

GTGAGTTTGATTAGGATTGATAATAATAAAAAAGTAATTGGGGTTTCTATTCCTTTAACT

------------TCAATTTCAGGCAAAGTGCGTGTGAAAATCAGACATGCCTTTAGCGAT

TATGGTATTTCAACAGCGACTAGAAAAATCCCTTTTAGCTTAAAACATTATGTAGAGTGG

CAAATCGGTTATGATGTCCCCATTAAAGATAAAGAA---AAATTTGAACTCACTACCCTA

AAAGATGAAAAATATCATTTTTTAGGGGCTAATAATAAAGTAAAAACCCTTTATGAATTG

AGTGAGATAATCTATTACGCTAAGCAATTAAATTTAATCAGT---------TTAGAAAAT

TTAGAAAATACTTTAAAATATTTAGAAAAACAAAAACAATTTATAGAAGATAATTTCACG

ATTACAAGAGAAAGATTTAGATCGCATCAATTTGGTGGCATGGATTTTGAACTCTCACGC

ATTTCTTATCCTTTACTCATTCATTCTTTTAATGATAATCAATTGAGTGAAATCGTTATT

AGAGAGCAACAATATGGCTCTAAAACCCAAGCCATG---CTGTATTTTTGCTTTTCTATT

TTGGAGTTAAAAACCGCTACCCCCTTATTAAATAGAACGGCTGCACTCAAAGAACAGGCT

CTTTTGATTATCCATAAAACCAACGCTCTCATGTTTTTAGAAATGCTTAAAATTTTTGGA

CTTTTAAGCCAAGCACACCATAACGATGTGTTAAAGATTTTAGAAAAAATACTTCAAAAT

>ZH138

GTGAGTTTGATTAAGATTGATAATGATAAAAAAGTGATTGAGGTTTCTATTCCTTTAACT

------------TCCATTTCAGGCAAAGTGCGTGTGAAAATCAGACATGCCTTTAGCGAT

TATGGTATTTCAACAGCGACCAGAAAAATCCCTTTTAGCTTAAAACATTATGTAGAGTGG

CAAATCGGTTATGATGTCCCCATTAAAGATAAAGAA---AAATTTGAGCTCACTACCCTA

AAAGATGAAAAATATCATTTTTTAGGGGCTAATAATAAAGTAAAAACCCTTTATGAATTG

AGTGAGATAATCTATTACGCTAAGCAATTAAATTTAATCAGT---------TTAGAAAAT

TTAGAAAATACTTTAAAATATTTAGAAAAACAAAAACAATTTATAGAAGATAATTTCACG

ATTACAAGAGAAAGATTTAGATCGCATCAATTTGGTGGCATGGATTTTGAACTTTCACGC

ATTTCTTATCCTTTACTCATTCATTCTTTTAATGATAATCAATTGAGTGAAATCGTTATT

AGAGAGCAACAATATGGCTCTAAAACCCAAGCCATG---CTGTATTTTTGCTTTTCTATT

TTGGAGTTAAAAACCGCTACCCCTTTATTAAATAGAACGGCTGCACTCAAAGAACAGGCT

CTTTTAACCATCCATAAAACCAACGCTCTTATGTTTTTAGAAATGCTTAAAATTTTTGGA

CTTTTAAGCCAAGCGCACCATAGCGATGTGTTAAAGATTTTAGAAAAAATACTTCAAAAT

>3843

GTGAGTTTGATTAAAGTCAGTGATGATAAAAAAGCGATTGAGGTTTCTATTCCTTTAACT

------------TCAATTTCAGGCAAAGTGCGTGTGAAAATCAGACATGCCTTTAGCGAT

TATGGTATTTCAACAGCGACTAGAAAAATCCCTTTTAGTTTAAAACATTATGTAGAGTGG

CAAATCGGTTATGATGTCCCTATTAAAGATAAAGAA---AAATTGGAGCTCACTACCCTA

AAAGATGAAAAATATCATTTTTTAGGGGCTAATAATAAAGTAAAGACTCTTTATGAATTA

AGCGAAATGATTGATTACGCTAAGCAATTAGGTTTAATTGGT---------TTAGAAAAT

TTAGAAAATACTTTAAAATATTTAGAAAAACAAAAACAATTTATAGAAGATAATTTCACG

ATTACAAGAGAAAGATTTAGATCGCATCAATTTGGTGGCATGGATTTTGAACTTTCACGC

ATTTCTTATCCTTTGCTCATTCATTCTTTTAATGATGATCAGTTGAGCGAAATTATTATT

AGAGAGCAACAATACGGCTCTAAAACCCAAGCCATG---CTGTATTTTTGCTTTTCTATT

TTGGAATTAAAAACCGCTACTCCCTTATTAAATAGAACGGCTGCACTCAAAGAACATGCC

CTTTTAACTATCCATAAAACCAACGCTCTTATGTTTTTAGAAATGCTTAAAATTTTTGGA

CTTTTAAGTCAAGTGCACCATAACGATGTGTTAAAGATTTTAGAAAAAATACTTCAAAAT

>KH0065

GTGAGTTTGATTAAAATTAACCATGATGAAAAAGTGATTGAGGTTTCTATTCCTTTAACT

------------TCAATTTCAGGCAAAGTGCGTGTGAAAATCAGACATGCCTTTAGCGAT

TATGGTATTTCAACAGCGACTAGAAAAATCCCTTTTAGTTTAAAGCATTATGTAGAGTGG

CAGATCGGTTATGATGTCCCCATTAAAGATAAAGAA---AAATTTGAACTCACTACTTTA

AAAGATGAAAAATATCATTTTTTAGGGGCTAATAATAAAGTAAAAACTCTTTATGAATTG

AGCGAAATGATTTATTACGCTAAGCAATTAGGTTTAATCAGT---------TTAGAAAAT

TTAGAAAATACTTTAAAATATTTAGAAAAACAAAAACAATTTATAGAAGATAATTTTATG

ATTACAAGAGAAAGATTTAGATCGCATCAATTTGGTGGCATGGATTTTGAACTCTCACGC

ATTTCTTATCCTTTACTCATTCATTCTTTTAATGATAATGAGTTGAGCGAAATAGTTATT

AGAGAACAACAATATGGCTCTAAAACCCAAGCCATG---CTGTATTTTTGCTTTTCTATT

TTGGAATTAAAAACCGCTACTCCCTTATTAAACAGAACCACTACGCTCAAAGAACATGCC

CTTTTAACTATCCATAAAACCAACGCTCTCATGTTTTTAGAAATGCTTAAAATTTTTGGA

CTTTTAAGCCAAGCGCACCATAACGATGTGTTAAAGATTTTAGAAAAAATACTTCAAAAT

>23:2_single

GTGAGTTTGATTAGGATTGATGATAGTAAAAAAGCGATTGAGGTTTCCATTCCTTTAACT

------------TCCATTTCAGGCAAAGTGCGTGTGAAAATCAGACATGCCTTTAGCGAT

TATGGCATTTCAACAGCGACCAGAAAAATCCCTTTTAGTTTAAAACATTATGTAGAGTGG

CAAATCGGTTATGATGTCCCTATTAAAGATAAAGAA---AAATTTGAATTCACTACTTTA

AAAGATGAAAAATATCATTTTTTAGGGGCTAATGATAAAGTAAAAACTCTTTATGAATTG

AGTGAAATGATTTATTACGCTAAGCAATTAGGTTTAATCAGT---------TTAGAAAAT

TTAGAAAATACTTTAAAATATTTAGAAAAACAAAAACAATTTATAGAAGATAATTTTATG

ATTACAAGAGAAAGATTTAGATCGCATCAATTTGGTGGCATGGATTTTGAACTCTCACGC

ATTTCTTATCCTTTGCTCATTCATTCTTTTAATGATAATCAGTTGAGCGAAATTGTTATT

AGAGAGCAACAATATGGCTCTAAAACCCAAGCCATG---CTGTATTTTTGCTTTTCTATT

TTGGAGTTAAAAACCGCTACCCCCTTATTAAACAGAACGGCTATGCTCAAAGAGCATGCT

CTTTTGATTATCCATAAAACCAACGCTCCCATGTTTTTAGAAATGCTTAAAATTTTTGGG

CTTTTAAGCCAAGCGCACCATGACGATGTGTTAAAGATTTTAGAAAAAATACTTCAAAAT

>476-C-EK5

GTGAGTTTGATTAAAGTCAGTGATGATAAAAAAGCGATTGAGGTTTCTATTCCTTTAACT

------------TCAATTTCAGGCAAAGTGCGTGTGAAAATCAGACATGCCTTTAGCGAT

TATGGTATTTCAACAGCGACTAGAAAAATCCCTTTTAGTTTAAAACATTATGTAGAGTGG

CAGATCGGTTATGATGTCCCCATTAAAGATAAAGAA---AAATTTGAACTCACTACTTTA

AAAGATGAAAAATATCATTTTTTAGGGGCTAATAATAAAGTGAAAACTCTTTATGAATTG

AGTGAAATGATTTATTACGCTAAGCAATTAGGTTTAATCAGT---------TTAGAAAAT

TTAGAAAATACTTTAAAATATTTAGAAAAACAAAAACAATTTATAGAAGATAATTTTATG

ATTACAAGAGAAAGATTTAGATCGCATCAATTTGGTGGCATGGATTTTGAACTTTCACGC

ATTTCTTATCCTTTACTCATTCATTCTTTTAATGATAATCAATTGAGTGAAATCGTTATT

AGAGAGCAACAATACGACTCTAAAACCCAAGCCATG---CTGTATTTTTGCTTTTCTATT

TTGGAATTAAAAACCGCTACTCCCTTATTAAATAGAACGGCTGCACTCAAAGAACATGCC

CTTTTAACTATCCATAAAACCAACGCTCTTGTGTTTTTAGAAATGCTTAAAATTTTTGGA

CTTTTAAGTCAAGCGCACCATAACGATGTGTTAAAGATTTTAGAAAAAATACTTCAAAAT

>476-A-EK5

GTGAGTTTGATTAAAGTCAGTGATGATAAAAAAGCGATTGAGGTTTCTATTCCTTTAACT

------------TCAATTTCAGGCAAAGTGCGTGTGAAAATCAGACATGCCTTTAGCGAT

TATGGTATTTCAACAGCGACTAGAAAAATCCCTTTTAGTTTAAAACATTATGTAGAGTGG

CAGATCGGTTATGATGTCCCCATTAAAGATAAAGAA---AAATTTGAACTCACTACTTTA

AAAGATGAAAAATATCATTTTTTAGGGGCTAATAATAAAGTGAAAACTCTTTATGAATTG

AGTGAAATGATTTATTACGCTAAGCAATTAGGTTTAATCAGT---------TTAGAAAAT

TTAGAAAATACTTTAAAATATTTAGAAAAACAAAAACAATTTATAGAAGATAATTTTATG

ATTACAAGAGAAAGATTTAGATCGCATCAATTTGGTGGCATGGATTTTGAACTTTCACGC

ATTTCTTATCCTTTACTCATTCATTCTTTTAATGATAATCAATTGAGTGAAATCGTTATT

AGAGAGCAACAATACGACTCTAAAACCCAAGCCATG---CTGTATTTTTGCTTTTCTATT

TTGGAATTAAAAACCGCTACTCCCTTATTAAATAGAACGGCTGCACTCAAAGAACATGCC

CTTTTAACTATCCATAAAACCAACGCTCTTGTGTTTTTAGAAATGCTTAAAATTTTTGGA

CTTTTAAGTCAAGCGCACCATAACGATGTGTTAAAGATTTTAGAAAAAATACTTCAAAAT

>476-A2-EK2

GTGAGTTTGATTAAAGTCAGTGATGATAAAAAAGCGATTGAGGTTTCTATTCCTTTAACT

------------TCAATTTCAGGCAAAGTGCGTGTGAAAATCAGACATGCCTTTAGCGAT

TATGGTATTTCAACAGCGACTAGAAAAATCCCTTTTAGTTTAAAACATTATGTAGAGTGG

CAGATCGGTTATGATGTCCCCATTAAAGATAAAGAA---AAATTTGAACTCACTACTTTA

AAAGATGAAAAATATCATTTTTTAGGGGCTAATAATAAAGTGAAAACTCTTTATGAATTG

AGTGAAATGATTTATTACGCTAAGCAATTAGGTTTAATCAGT---------TTAGAAAAT

TTAGAAAATACTTTAAAATATTTAGAAAAACAAAAACAATTTATAGAAGATAATTTTATG

ATTACAAGAGAAAGATTTAGATCGCATCAATTTGGTGGCATGGATTTTGAACTTTCACGC

ATTTCTTATCCTTTACTCATTCATTCTTTTAATGATAATCAATTGAGTGAAATCGTTATT

AGAGAGCAACAATACGACTCTAAAACCCAAGCCATG---CTGTATTTTTGCTTTTCTATT

TTGGAATTAAAAACCGCTACTCCCTTATTAAATAGAACGGCTGCACTCAAAGAACATGCC

CTTTTAACTATCCATAAAACCAACGCTCTTGTGTTTTTAGAAATGCTTAAAATTTTTGGA

CTTTTAAGTCAAGCGCACCATAACGATGTGTTAAAGATTTTAGAAAAAATACTTCAAAAT

>55:5

GTGAGTTTGATTAGGATTGATAATAATAAAAAAGTAATTGGGGTTTCCATTCCCTTAACT

------------TCAATTTCAGGCAAAGCGCGTGTGAAAATCAGACATGCCTTTAGCGAT

TGTGGTATTTCAACAGCGACTAGAAAAATCCCTTTTAGTTTAAAGCATTATGTAGAGTGG

CAAATCGGTTATGATGTCCCCATTAAAGATAAAGAA---AAATTTGAGCTCACTACTTTA

AAAGATGAAAAATATCATTTTTTAGGGGCTAATAATAAAGCAAAGACTCTTTATGAATTG

AGCGAAATGATTGATTACGCTAAGCGATTGGGTTTAATCAGT---------TTAGAAAAT

TTAGAAAATACTTTAAAATATTTAGAAAAACAAAAACAATTCATAGAAGATAATTTTATG

ATTACAAGAGAAAGATTTAGATCGCATCAATTTGGGGGCATGGATTTTGAACTTTCACGC

ATTTCTTATCCCTTACTCATTCATTCTTTTAATGATAATCAATTGAGCGAAATCGTTATT

AGAGAGCAACAATACGGCTCTAAAACCCAAGCCATG---CTGTATTTTTGCTTTTCTATT

TTGGAATTAAAAACCGCTACTCCCTTATTAAATAGAACGGCTGCACTCAAAGAACATGCC

CTTTTAACTATCCATAAAACCAACGCTCTTATGTTTTTAGAAATGCTTAAAATTTTTGGA

CTTTTAAGCCAAGCGCACCATAACGATGTGTTAAAGATTTTAGAAAAAATACTTCAAAAT

>B35

GTGAGTTTGATTAAGATTGATAATGATAAAAAAGTGATTGAGGTTTCTATTCCTTTAACT

------------TCCATTTCAGGCAAAGTGCGTGTGAAAATCAGACATGCCTTTAGCGAT

TATGGTATTTCAACAGCGACTAGAAAAATCCCTTTTAGCTTAAAACATTATGTAGAGTGG

CAAATCGGTTATGATGTCCCCATTAAAGATAAAGAA---AAATTTGAGCTCACTACCCTA

AAAGATGAAAAATATCATTTTTTAGGGGCTAATAATAAAGTAAAAACTCTTTATGAATTG

AGTGAGATAATCTATTACGCTAAGCAATTAGGTTTAATCAGT---------TTAGAAAAT

TTAGAAAATACTTTAAAATATTTAGAAAAACAAAAACAATTCATAGAAGATAATTTCATG

ATTACAAGAGAAAGATTTAGATCGCATCAATTTGGGGGCATGGGTTTTGAACTTTCACGC

ATTTCTTACCCTTTACTCATTCATTCTTTTAATGATGATCAATTGAGCGAAATCGTTATT

AGGGAACAACAATATGGTTCTAAAACCCAAGCCATG---CTGTATTTTTGCTTTTCTATT

TTGGAGTTAAAAACCGCTATCCCCTTATTAAACAGAACGGCTGCACTCAAAGAACATGCT

CTTTTGATTATTCATAAAACCAACGCTCTCATGTTTTTAGAAATGCTTAAAATTTTTGGA

CTTTTAAGCCAAGCACACCATAATGATGTGTTAAAGATTTTAGAAAAAATACTTCAAAAT

>MHP22

GTGAGTTTGATTAAGATTGATAATAATAAAAAAGTAATTGAGGTTTCTATTCCTTTAACT

------------TCCATTTCAGGCAAAGCGCGTGTGAAAATCAGACATGCCTTTAGCGAT

TATGGCATTTCAACAGCGACTAGAAAAATCCCTTTCAGTTTAAAGCATTATGTAGAGTGG

CAAATCGGTTATGATGTCCCCATTAAAGATAAAGAA---AAATTTGAGCTCACTACTTTA

AAAGATGAAAAATATCATTTTTTAGGGGCTAATAATAAAGTAAAAACCCTTTATGAATTG

AGCGAAATAATTTATTATGCTAAGCGATTGGGTTTAATCAGT---------TTAGAAAAT

TTAGAAAATACTTTAAAATATTTAGAAAAACAAAAACAATTTATAGAAGATAATTTTATG

ATTACAAGAGAAAGATTTAGATCGCATCAATTTGGAGGCATGGATTTTGAACTCTCACGC

ATTTCTTATCCTTTACTCATTCATTCTTTCAACGATAATCAATTAAGTGAAATCGTTATT

AGAGAGCAACAATATGGCTCTAAAACCCAAGCCATG---CTGTATTTTTGCTTTTCTATT

TTGGAATTAAAAACCGCTACCCCTTTATTAAATAGAACGGCTGCACTCAAAGAACATGCT

CTTTTAATTATCTATAAAACTAACGCTCTTGTATTTTTAGAAATGCTTAAAATTTTTGGA

CTTTTAAGCCAAGCGCATCATAACGATGTGTTAAAGATTTTAGAAAAAATACTTGAAAAT

>ZH121

GTGAGTTTGATTAGGATTGATGATAGTAAAAAAGCGATTGAGGTTTCCATTCCTTTAACT

------------TCAATTTCAGGCAAAGTGCGTGTGAAAATCAGACATGCCTTTAGCGAT

TATGGCATTTCAACAGCGACCAGAAAAATCCCTTTTAGTTTAAAACATTATGTAGAGTGG

CAAATCGGTTATAATGTCCCCATTAAAGATAAAGAA---AAATTTGAACTCACTACTTTA

AAAGATGAAAAATATCATTTTTTAGGGGCTAATGATAAAGTAAAAACTCTTTATGAATTA

AGCGAAATGATTTATTACGCTAAACAATTAGGTTTAATCAGT---------TTAGAAAAT

TTAGAAAATACTTTAAAATATTTAGAAAAACAAAAACAATTTATAGAAGATAATTTTATG

ATTACAAGAGAAAGATTTAGATTACATCAATTTGGTGGCATGGATTTTGAACTTTCACGC

ATTTCTTATCCTTTACTCATTCATTCTTTTAATGATAATGAGTTGAGCGAAATCGTTATT

AGAGAGCAACAATATGGCTCTAAAACCCAGGCCATG---CTGTATTTTTGCTTTTCTATT

TTGGAATTAAAAACCGCTACTCCCTTATTAAATAGAACGGCTGCACTCAAAGAACATGCC

CTTTTAACTATCCATAAAACCAACGCTCTTATGTTTTTAGAAATGCTTAAAATTTTTGGA

CTTTTAAGCCAAGTGCACCGTAACGATGTGTTAAAGATTTTAGAAAAAATACTTCAAAAT

>ZH83

GTGAGTTTGATTAGGATTGATAATAATAAAAAAGTAATTGGGGTTTCTATTCCTTTAACT

------------TCAATTTCAGGCAAAGTGCGTGTGAAAATCAGACATGCCTTTAGCGAT

TATGGTATTTCAACAGCGACTAGAAAAATCCCTTTTAGCTTAAAACATTATGTAGAGTGG

CAAATCGGTTATGATGTCCCCATTAAAGATAAAGAA---AAATTGGAGCTCACTACCCTA

AAAGATGAAAAATATCATTTTTTAGGGGCTAATAATAAAGTAAAAACCCTTTATGAATTG

AGTGAGATAATCTATTACGCTAAGCAATTAAATTTAATCAGT---------TTAGAAAAT

TTAGAAAATACTTTAAAATATTTAGAAAAACAAAAACAATTTATAGAAGATAATTTCACG

ATTACAAGAGAAAGATTTAGATCGCATCAATTTGGTGGCATGGATTTTGAACTTTCACGC

ATTTCTTATCCTTTACTCATTCATTCTTTTAATGATAATCAATTGAGTGAAATCGTTATT

AGAGAGCAACAATATGGCTCTAAAACCCAAGCCATG---CTGTATTTTTGCTTTTCTATT

TTGGAATTAAAAACCACTACCCCCTTATTAAATAGAACGGCTACACTCAAAGAACATGCC

CTTTTAACTATCCATAAAACCAACGCTCTCATGTTTTTAGAAATGCTTAAAATTTTTGGA

CTTTTAAGCCAAGCGCACCATAACGATGTGTTAAAGATTTTAGAAAAAATACTTCAAAAT

>ZH101

GTGAGTTTGATTAAAGTTAATGATGATAAAAAAGTGATTGAGATTTCTATTCCTTTAACT

------------TCAATTTCAGGCAAAGTGCGTGTGAAAATCAGACATGCCTTTAGCGAT

TATGGTATTTCAACAGCGACCAGAAAAATCCCTTTTAGCTTAAAACATTATGTAGAGTGG

CAAATCGGTTATGATGTCCCCATTAAAGATAAAGAA---AAATTTGAGCTCACTACCCTA

AAAGATGAAAAATATCATTTTTTAGGGGCTAATAATAAAGTAAAAACCCTTTATGAATTG

AGTGAGATAATCTATTACGCTAAGCAATTAAATTTAATCAGT---------TTAGAAAAT

TTAGAAAATACTTTAAAATATTCAGAAAAACAAAAACAATTTATAGAAGATAATTTTATG

ATTACAAGAGAAAGATTTAGATCGCATCAATTTGGTGGAATGGATTTTGAACTCTCACGC

ATTTCTTATCCTTTGCTCATTCATTCTTTTAATGATAATCAGTTGAGCGAAATCGTTATT

AGAGAGCAACAATATGGCTCTAAAACCCAAGCCATG---CTGTATTTTTGCTTTTCTATT

TTGGAATTAAAAACCGCTACCCCCTTATTAAATAGAACGGCTGCACTCAAAGAACATGCC

CTTTTAACCATCCATAAAACCAACGCTCTTATGTTTTTAGAAATGCTTAAAATTTTTGGA

CTTTTAAGCCAAGCGCACCATAACGATGTGTTAAAGATTTTAGAAAAAATACTTCAAAAT

>B497A

GTGAGTTTGATTAGGATTGATAATAATAAAAAAGTAATTGGGGTTTCTATTCCTTTAACT

------------TCAATTTCAGGCAAAGTGCGTGTGAAAATCAGACATGCCTTTAGCGAT

TATGGCATTTCAACAGCGACTAGAAAAATCCCTTTTAGTTTAAAGCATTATGTAGAGTGG

CAAATCGGTTATGATGTCCCCATTAAAGATAAAGAA---AAATTTGAACTCACTACTTTA

AAAGATGAAAAATATCATTTTTTAGGGGTTAACAATAAAGTAAAGACTCTTTATGAATTG

AGTGAGATAATCTATTACGCTAAGCAATTAAATTTAATCAGT---------TTAGAAAAT

TTAGAAAATACTTTAAAATATTTAGAAAAACAAAAACAATTTATAGAAGATAATTTCACG

ATTACAAGAGAAAGATTTAGATCGCATCAATTTGGTGGCGTGGATTTTGAACTTTCACGC

ATTTCTTATCCTTTACTCATTCATTCTTTTAATGATAATCAATTGAGTGAAATCGTTATT

AGAGAGCAACAATATGGCTCTAAAACCCAAGCCATG---CTGTATTTTTGCTTTTCTATT

TTAGAGTTAAAAACCGCTACTCCCTTATTGAATAGAACGGCTGCACTCAAAGAACATGCC

CTTTTAACTATCCATAAAACCAACGCTCTTGTGTTTTTAGAAATGCTTAAAATTTTTGGA

CTTTTAAGCCAAGCGCACCATAACGATGTGTTAAAGATTTTAGAAAAAATACTTCAAAAT

>ZH139

GTGAATTTGATTAAGATTGATAATAATAAAAAAGTAATTGAGATTTCTGTTCCTTTAACT

------------TCCATTTCAGGCAAAGTGCGTGTGAAAATCAGACATGCCTTTAGCGAT

TATGGCATTTCAACAGCGACCAGAAAAATCCCTTTTAGCTTAAAGCATTATGTAGAGTGG

CAAATCGGCTATGATGTCCCCATTAAAGATAAAGAA---AAATTTGAACTCACTACCCTA

AAAGATGAAAAATATCATTTTTTAGGGGCTAATAATAAAGTAAAAACCCTTTATGAATTG

AGTGAAATAATCTATTACGCTAAGCAATTAGGTTTAATCAGT---------TTAGAAAAT

TTAGAAAATATTTTAAAATATTTAGAAAAACAAAAACAATTTATAGAAGATAATTTCACG

ATTACAAGAGAAAGATTTAGATCGCATCAATTTGGTGGCATGGATTTTGAACTTTCACGC

ATTTCTTATCCTTTACTCATTCATTCTTTTAATGATAATCAATTGAGTGAAATCGTTATT

AGAGAGCAACAATATGGCTCTAAAACTCAAGCCATG---CTGTATTTTTGCTTTTCTATT

TTGGAATTAAAAACCGCTACCCCCTTATTAAATAGAACGGCTGCACTCAAAGAACATGCT

CTTTTAACCATCAATAAAACCAACGCTCTTGTGTTTTTAGAAATGCTTAAAATTTTTGGA

CTTTTAAGCCAAGCGCACCATAACGATGTGTTAAAGATTTTAGAAAAAATACTTCAAAAT

>B

GTGAGTTTGATTAGGATTGATAATAATAAAAAAGTAATTGGGGTTTCTATTCCTTTAACT

------------TCAATTTCAGGCAAAGTGCGTGTGAAAATCAGACATGCCTTTAGCGAT

TATGGTATTTCAACAGCGACCAGAAAAATCCCTTTTAGCTTAAAACATTATGTAGAGTGG

CAAATCGGTTATGATGTCCCCATTAAAGATAAAGAA---AAATTTGAGCTCACTACTTTA

AAAGATGAAAAATATCATTTTTTAGGGGCTAATAATAAAGTGAAAACTCTTTATGAATTA

AGCGAAATGATTTATTACGCTAAGCAATTAGGTTTAATTGGT---------TTAGAAAAT

TTAGAAAATACTTTAAAATATTTAGAAAAACAAAAACAATTTATAGAAGATAATTTCACG

ATTACAAGAGAAAGATTTAGATCGCATCAATTTGGTGGCATGGATTTTGAACTTTCACGC

ATTTCTTATCCCTTACTCATTCATTCTTTTAATGATAATCAATTGAGCGAAATCGTTATT

AGAGAACAACAATACGGCTCTAAGACACAAGCCATG---CTGTATTTTTGCTTTTCTATT

TTGGAGTTAAAAACCGCTACTCCCTTATTAAATAGAACCGCTACGCTCAAAGAACATGCC

CTTTTGATTGTCCATCAAACTAACGCTCTTGTGTTTTTAGAAATGCTTAAAATTTTTGGG

CTTTTAAGCCAAGCGCACCATAACGATGTGTTAAAGATTTTAGAAAAAATACTTCAAAAT

>MHP30

GTGAGTTTGATTAGGATTGATAATAATAAAAAAGTAATTGGGGTTTCTATTCCTTTAACT

------------TCAATTTCAGGCAAAGTGCGTGTGAAAATCAGACATGCCTTTAGCGAT

TATGGTATTTCAACAGCGACTAGAAAAATCCCTTTTAGCTTAAAACATTATGTAGAGTGG

CAAATCGGTTATGATGTCCCCATTAAAGATAAAGAA---AAATTTGAACTCACTACTTTA

AAAGATGAAAAATATCATTTTTTAGGGGCTAATAATAAAGTAAAGACTCTTTATGAATTG

AGTGAGATAATCTATTACGCTAAGCAATTAAATTTAATCAGT---------TTAGAAAAT

TTAGAAAATACTTTAAAATATTTAGAAAAACAAAAACAATTTATAGAAGATAATTTCACG

ATTACAAGAGAAAGATTTAGATCGCATCAATTTGGTGGCATGGATTTTGAACTTTCACGC

ATTTCTTATCCTTTACTCATTCATTCTTTTAATGATAATCAGTTGAGCGAAATAGTTATT

AGAGAACAACAATATGGCTCTAAAACCCAAGCCATG---CTGTATTTTTGCTTTTCTATT

TTGGAATTAAAAACCGCTACTCCCTTATTAAATAGAACGGCTATGCTCAAAGAGCATGCC

CTTTTAACTATCCATAAAACCAACGCTCCCATGTTTTTAGAAATGCTTAAAATTTTTGGA

CTTTTAAGCCAAGCGCACCATAACGATGTGTTAAAGATTTTAGAAAAAATACTTCAAAAT

>2017-105

GTGAGTTTGATTAAAGTTAATGATGATAAAAAAGCGATTGAGGTTTCTATTCCTTTAACT

------------TCCATTTCAGGCAAAGTGCGTGTGAAAATCAGACATGCCTTTAGCGAT

TATGGCATTTCAACAGCGACTAGAAAAATCCCTTTTAGTTTAAAGCATTATGTAGAGTGG

CAAATCGGTTATGATGTCCCTATTAAAGATAAAGAA---AAATTTGAACTCACTACCCTA

AAAGATGAAAAATATCATTTTTTAGGGGCTAATAATAAAGTAAAAACCCTTTATGAATTG

AGCGAAATAATTTATTACGCTAAGCAATTAGGTTTAATCAGT---------TTAGAAAAT

TTAGAAAATACTTTAAAATATTTAGAAAAGCAAAAACAATTCATAGAAGATAATTTTATG

ATCACAAGAGAAAGATTTAGATCCCATCAATTTGGTGGCATGGATTTTGAACTTTCACGC

ATTTCTTATCCTTTACTCATTCATTCTTTTAATGATAATCAGTTGAGCGAAATCGTTATT

AGAGAGCAACAATACGGTTCTAAAACCCAAGCCATG---CTGTATTTTTGCTTTTCTATT

CTGGAATTAAAAACCGCTACCCCTTTATTAAATAGAACGGCTGCACTCAAAGAACATGCT

CTTTTAACTATCCATAAAACCAACGCTCCCATGTTTTTAGAAATGCTTAAAATTTTTGGG

CTTTTAAGCCAAGCACACCATAACGATGTGTTAAAGATTTTAGAAAAAATACTTGAAAAT

>KH41

GTGAGTTTGATTAAAGTTGACTATGATAAAAAAGTGATTGAGGTTTCTATTCCTTTAACT

------------TCAATTTCAGGCAAAGTGCGTGTGAAAATCAGACATGCCTTTAGCGAT

TATGGTATTTCAACAGCGACTAGAAAAATCCCTTTTAGTTTAAAACATTATGTAGAGTGG

CAGATCGGTTATGATGTCCCCATTAAAGATAAAGAA---AAATTTGAACTCACTGCTTTA

AAAGATAAAAAATACCATTTTTTAGGGGCTAATAATAAAGTGAAAACTCTTTATGAATTG

AGCGAAACGATTTATTACGCTAAGCAATTAGGTTTAATCAGT---------TTAGAAAAT

TTAGAAAATACTTTAAAATATTTAGAAGAACAAAAACAATTTATAGAAGATAATTTTATG

ATTACAAGAGAAAGATTTAGATCGCATCAATTTGGTGGCATGGATTTTGAACTTTCACGC

ATTTCTTATCCTTTACTCATTCATTCTTTTAACGATAATCAATTGAGTGAAATCGTTATT

AGAGAACAACAATATGGCTCTAAAACCCAAGCCATG---CTGTATTTTTGCTTTTCTATT

TTGGAGTTAAAAACCGCTACTCCCTTATTAAATAGAACGGCTGCACTCAAAGAGCATGCC

CTTTTAACTATCCATAAAACCAACGCTCTTATGTTTTTAGAAATGCTTAAAATTTTTGGG

ATTTTAAGCCAAGCGCACCATAACGATGTGTTAAAGATTTTAGAAAAAATACTTCAAAAT

>2065

GTGAGTTTGATTGAGATTGATAATAATAAAAAAGTGATTGAGGTTTCTATTCCTTTAACT

------------TCCATTTCAGGCAAAGTGCGTGTGAAAATCAGACATGCCTTTAGCGAT

TATGGCATTTCAACAGCGACCAGAAAAATCCCTTTTAGTTTAAAACATTATGTAGAGTGG

CAAATCGGTTATGATGTCCCCATTAAAGATAAAGAA---AAATTTGAGCTCACTACTTTA

AAAGATGAAAAATATCATTTTTTAGGGGCTAACAATAAAGTAAAAACCCTTTATGAATTG

AGTGAGATAATCTATTACGCTAAGCAATTAAATTTAATCAGT---------TTAGAAAAT

TTAGAAAATACTTTAAAATATTTAGAAAAACAAAAACAATTCATAGAAGATAATTTCATG

ATTACAAGAGAAAGATTTAGATCGCATCAATTTGGTGGCATGGATTTTGAACTTTCACGC

ATTTCTTATCCTTTGCTCATTCATTCTTTTAATGATAATCAATTGAGTGAAATCGTTATT

AGAGAGCAACAATATGGCTCTAAAACCCAAGCCATG---CTGTATTTTTGCTTTTCTATT

TTGGAGTTAAAAACCGCTACCCCTTTATTAAATAGAACGGCTGCACTCAAAGAACAGGCT

CTTTTAACCATCAATAAAACCAACGCTCTTGTGTTTTTAGAAATGCTTAAAATTTTTGGA

CTTTTAAGCCAAGCGCACCATAGCGATGTGTTAAAGATTTTAGAAAAAATACTTCAAAAT

>1095

GTGAGTTTGATTGAGATTGATAATAATAAAAAAGTGATTGAGGTTTCTATTCCTTTAACT

------------TCCATTTCAGGCAAAGTGCGTGTGAAAATCAGACATGCCTTTAGCGAT

TATGGCATTTCAACAGCGACCAGAAAAATCCCTTTTAGTTTAAAACATTATGTAGAGTGG

CAAATCGGTTATGATGTCCCCATTAAAGATAAAGAA---AAATTTGAGCTCACTACTTTA

AAAGATGAAAAATATCATTTTTTAGGGGCTAACAATAAAGTAAAAACCCTTTATGAATTG

AGTGAGATAATCTATTACGCTAAGCAATTAAATTTAATCAGT---------TTAGAAAAT

TTAGAAAATACTTTAAAATATTTAGAAAAACAAAAACAATTCATAGAAGATAATTTCATG

ATTACAAGAGAAAGATTTAGATCGCATCAATTTGGTGGCATGGATTTTGAACTTTCACGC

ATTTCTTATCCTTTGCTCATTCATTCTTTTAATGATAATCAATTGAGTGAAATCGTTATT

AGAGAGCAACAATATGGCTCTAAAACCCAAGCCATG---CTGTATTTTTGCTTTTCTATT

TTGGAGTTAAAAACCGCTACCCCTTTATTAAATAGAACGGCTGCACTCAAAGAACAGGCT

CTTTTAACCATCAATAAAACCAACGCTCTTGTGTTTTTAGAAATGCTTAAAATTTTTGGA

CTTTTAAGCCAAGCGCACCATAGCGATGTGTTAAAGATTTTAGAAAAAATACTTCAAAAT

>B366

GTGAGTTTGATTAAAGTTAGTGGTGATAAAAAAGCGATTGAGGTTTCTATTCCTTTAACT

------------TCCATTTCAGGCAAAGTGCGTGTGAAAATCAGACATGCCTTTAGCGAT

TATGGTATTTCAACAGCGACCAGAAAAATCCCTTTTAGTTTAAAACATTATGTAGAGTGG

CAAATCGGTTATGATGTCCCCATTAAAGATAAAGAA---AAATTTGAGCTCACTACTTTA

AAAGATGAAAAATATCATTATTTAGGGGCTAATAATAAAGTAAAAACCCTTTATGAATTG

AGCGAAATGATTTATTACGCTAAGCAATTAAATTTAATCAGT---------TTAGAAAAT

TTAGAAAATACTTTAAAATATTTAGAAAAACAAAAACAATTTATAGAAGATAATTTTATG

ATTACAAGAGAAAGATTTAGATCGCATCAATTTGGTGGCATGGATTTTGAACTTTCACGC

ATCTCTTATCCTTTACTCATTCATTCTTTTAATGATAATCAGTTGAGCGAAATCGTTATT

AGAGAGCAACAATATGGCTCTAAAACCCAAGCCATG---CTGTATTTTTGCTTTTCTATT

TTGGAGTTAAAAACCGCTACCCCCTTATTAAACAGAACGGCTGCACTCAAAGAACATGCC

CTTTTAACTATCCATAAAACCAACGCTCTTATGTTTTTAGAAATGCTTAAAATTTTTGGA

CTTTTAAGCCAAGCGCACCATAGCGATGTGTTAAAGATTTTAGAAAAAATACTTCAAAAT

>ZH97

GTGAGTTTGATTAAAGTTAGTGGTGATAAAAAAGTGATTGAGGTTTCTATTCCTTTAACT

------------TCAATTTCAGGCAAAGTGCGTGTGAAAATCAGACATGCCTTTAGCGAT

TATGGCATTTCAACAGCGACCAGAAAAATCCCTTTTAGCTTAAAACATTATGTAGAGTGG

CAAATCGGTTATGATGTCCCCATTAAAGATAAAGAA---AAATTTGAGCTCACTACCCTA

AAAGATGAAAAATATCATTTTTTAGGGGCTAATAATAAAGTAAAAACCCTTTATGAATTG

AGTGAGATAATCTATTACGCTAAGCAATTAAATTTAATCAGT---------TTAGAAAAT

TTAGAAAATACTTTAAAATATTTAGAAAAACAAAAACAATTCATAGAAGATAATTTTATG

ATTACAAGAGAAAGATTTAGATCGCATCAATTTGGTGGCATGGATTTTGAACTTTCACGC

ATTTCTTATCCTTTACTCATTCATTCTTTCAATGATAATCAATTGAGTGAAATCGTTATT

AGAGAGCAACAATATGGCTCTAAAACCCAAGCCATG---CTGTATTTTTGCTTTTCTATT

TTGGAATTAAAAACCGCTACCCCCTTATTAAATAGAACGGCTGCACTCAAAGAACATGCT

CTTTTAACCATCAATAAAACCAACGCTCTTATGTTTTTAGAAATGCTTAAAATTTTTGGA

CTTTTAAGCCAAGCGCACCATAACGATGTGTTAAAGATTTTAGAAAAAATACTTCAAAAT

>ZH67

GTGAGTTTGATTAAGATTGATAATAATAAAAAAGTAATTGAGGTTTCTATTCCTTTAACT

------------TCAATTTCAGGCAAAGTGCGTGTGAAAATCAGACATGCCTTTAGCGAT

TATGGCATTTCAACAGCGACTAGAAAAATCCCTTTTAGTTTAAAACATTATGTAGAGTGG

CAAATCGGTTATGATGTCCCCATTAAAGATAAAGAA---AAATTGGAGCTCACTACCCTA

AAAGATGAAAAATATCATTTTTTAGGGGCTAATAATAAAGTAAAGACTCTTTATGAATTG

AGCGAAATGATTTATTACGCTAAGCGATTGGGTTTAATCAGT---------TTAGAAAAT

TTAGAAAATGCTTTAAAATATTTAGAAAAACAAAAACAATTTATAGAAGATAATTTTATG

ATTACAAGAGAAAGATTTAGATCGCATCAATTTGGTGGCATGGATTTTGAACTCTCACGC

ATTTCTTATCCCTTACTTATTCATTCTTTTAATGATAATCAGTTGAGCGAAATTGTTATT

AGAGAGCAACAATATGGCTCTAAAACCCAAGCCATG---CTGTATTTTTGCTTTTCTATT

TTGGAGTTAAAAACCGCTACCCCCTTATTAAACAGAACGGCTATGCTCAAAGAGCATGCT

CTTTTGATTATCCATAAAACCAACGCTCTCATGTTTTTAGAAATGCTTAAAATTTTTGGA

CTTTTAAGCCAAGTGCACCATAGCGATGTGTTAAAGATTTTAGAAAAAATACTTCAAAAT

>ZH29

GTGAGTTTAATTAGGATTGATGATAGTAAAAAAGCGATTGAGGTTTCTATTCCTTTAACT

------------TCAATTTCAGGCAAAGTGCGTGTGAAAATCAGACATGCCTTTAGCGAT

TATGGTATTTCAACAGCGACCAGAAAAATCCCTTTCAGTTTAAAGCATTATGTAGAGTGG

CAAATCGGTTATGATGTCCCCATTAAAGATAAAGAA---AAATTTGAGCTCACTACCCTA

AAAGATGAAAAATATCATTTTTTAGGGGCTAATAATAAAGTAAAAACTCTTTATGAATTG

AGTGAAATAATCTATTACGCTAAGCAATTAAATTTAATCAGT---------TTAGAAAAT

TTAGAAAATACTTTAAAATATTTAGAAAAACAAAAACAATTTATAGAAGATAATTTTATG

ATTACAAGAGAAAGATTTAGATTACATCAATTTGGTGGCATGGATTTTGAACTCTCACGC

ATTTCTTATCCCTTACTCATTCATTCTTTTAATGATAATCAGTTGAGCGAAATCGTTATT

AGAGAGCAACAATACGACTCTAAAACCCAAGCCATG---CTGTATTTTTGCTTTTCTATT

TTGGAATTAAAAACCGCTACTCCCTTATTAAATAGAACGGCTGCACTCAAAGAACATGCC

CTTTTAACTATCCATAAAACCAACGCTCTTATGTTTTTAGAAATGCTTAAAATTTTTGGA

CTTTTAAGTCAAGCGCACCATAACGATGTGTTAAAGATTTTAGAAAAAATACTTCAAAAT

>228/99

GTGAGTTTGATTGAGATTGATAATAATAAAAAAGTAATTGAGATTTCTATTCCTTTAACT

------------TCAATTTCAGGCAAAGTGCGTGTGAAAATCAGGCATGCCTTTAGCGAT

TATGGTATTTCAACAGCGACTAGAAAAATCCCTTTTAGCTTAAAACATTATGTAGAGTGG

CAAATCGGTTATGATGTCCCCATTAAAGATAAAGAA---AAATTTGAGCTCACTACTTTA

AAAGATGAAAAATATCATTTTTTAGGGGCTAACAATAAAGTAAAAACCCTTTATGAATTG

AGTGAGATAATCTATTATGCTAAGCAATTAAATTTAATCAGT---------TTAGAAAAT

TTAGAAAATACTTTAAAATATTTAGAAAAACAAAAACAATTCATAGAAGATAATTTTATG

ATCACAAGAGAAAGATTTAGATTACATCAATTTGGTGGCATGGATTTTGAACTCTCACGC

ATTTCTTATCCTTTGCTCATTCATTCTTTTAATGATAATCAGTTGAGCGAAATCGTTATT

AGAGAGCAACAATACGGCTCTAAAACCCAAGCCATG---CTGTATTTTTGCTTTTCTATT

TTGGAATTAAAAACCGCTACCCCCTTATTAAATAGAGCGGCTACACTCAAAGAACATGCT

TTTTTAACCATCCATAAAACCAACGCTCTTATGTTTTTAGAAATGCTTAAAATTTTTGGA

CTTTTAAGCCAAGCGCACCATAACGATGTGTTAAAGATTTTAGAAAAAATACTTCAAAAT

>2017-54

GTGAGTTTGATTAAGATTGATAATGATAAAAAAGTAATTGAGGTTTCTATTCCTTTAACT

------------TCCATTTCAGGCAAAGTGCGTGTGAAAATCAGACATGCCTTTAGCGAT

TATGGTATTTCAACAGCGACTAGAAAAATCCCTTTTAGTTTAAAACATTATATAGAGTGG

CAGATCGGTTATGATGTCCCCATTAAAGATAAAGAA---AAATTTGAACTCACTACTTTA

AAAGATGAAAAATATCATTTTTTAGGGGCTAATAATAAAGTGAAAACTCTTTATGAATTA

AGCGAAATAATTGATTACGCTAAGCAATTAGGTTTAATCAGT---------TTAGAAAAT

TTAGAAAATACTTTAAAATATTTAGAAAAGCAAAAACAATTCATAGAAGATAGTTTTATG

ATCACAAGAGAAAGATTTAGATCGCATCAATTTGGTGGCATGGATTTTGAACTTTCACGC

ATTTCTTATCCCTTACTCATTCATTCTTTTAATGATAATCAATTGAGTGAAATCGTTATT

AGAGAGCAACAATACGGCTCTAAAACCCAAGCCATG---CTGTATTTTTGCTTTTCTATT

TTGGAATTAAAAACCGCTACCCCTTTATTAAATAGAACGGCTGCACTCAAAGAACATGCT

TTTTTAATTATCCATAAAACCAACGCTCTCATGTTTTTAGAAATGCTTAAAATTTTTGGA

CTTTTAAGCCAAGCGCACCATAACGATGTGTTAAAGATTTTAGAAAAAATACTTCAAAAT

>2017-107

GTGAGTTTGATTAAGATTGATAATGATAAAAAAGTAATTGAGGTTTCTATTCCTTTAACT

------------TCCATTTCAGGCAAAGTGCGTGTGAAAATCAGACATGCCTTTAGCGAT

TATGGTATTTCAACAGCGACTAGAAAAATCCCTTTTAGTTTAAAACATTATATAGAGTGG

CAGATCGGTTATGATGTCCCCATTAAAGATAAAGAA---AAATTTGAACTCACTACTTTA

AAAGATGAAAAATATCATTTTTTAGGGGCTAATAATAAAGTGAAAACTCTTTATGAATTA

AGCGAAATAATTGATTACGCTAAGCAATTAGGTTTAATCAGT---------TTAGAAAAT

TTAGAAAATACTTTAAAATATTTAGAAAAGCAAAAACAATTCATAGAAGATAGTTTTATG

ATCACAAGAGAAAGATTTAGATCGCATCAATTTGGTGGCATGGATTTTGAACTTTCACGC

ATTTCTTATCCCTTACTCATTCATTCTTTTAATGATAATCAATTGAGTGAAATCGTTATT

AGAGAGCAACAATACGGCTCTAAAACCCAAGCCATG---CTGTATTTTTGCTTTTCTATT

TTGGAATTAAAAACCGCTACCCCTTTATTAAATAGAACGGCTGCACTCAAAGAACATGCT

TTTTTAATTATCCATAAAACCAACGCTCTCATGTTTTTAGAAATGCTTAAAATTTTTGGA

CTTTTAAGCCAAGCGCACCATAACGATGTGTTAAAGATTTTAGAAAAAATACTTCAAAAT

>19B6

GTGAGTTTGATTAGGATTGATGATAGTAAAAAAGTAATTGAGGTTTCTATTCCTTTAACT

------------TCAATTTCAGGCAAAGTGCGTGTGAAAATCAGGCATGCCTTTAGCGAT

TATGGTATTTCAACAGCGACTAGAAAAATCCCTTTTAGTTTAAAGCATTATGTAGAGTGG

CAAATCGGTTATGATGTCCCCATTGAAGATAAAGAA---AAATTTGAGCTCACTACCCTA

AAAGATGAAAAATATCATTTTTTAGGGGCTAATAATAAAGTAAAAACTCTTTATGAATTA

AGCGAAATGATTTATTACGCTAAGCAATTAGGTTTAATCAGT---------TTAGAAAAT

TTAGAAAATACTTTAAAATATTTAGAAAAACAAAAACAATTTATAGAAGATAATTTCACG

ATTACAAGAGAAAGATTTAGATCGCATCAATTTGGGGGCATGGATTTTGAACTTTCACGC

ATTTCTTATCCCTTACTCATTCATTCTTTTAATGATAATCAATTGAGCGAAATCGTTATT

AGAGAACAACAATACGGCTCTAAGACACAAGCCATG---CTGTATTTTTGCTTTTCTATT

TTGGAGTTAAAAACCGCTACTCCCTTATTAAATAGAACCGCTACGCTCAAAGAACATGCC

CTTTTGATTGTCCATCAAACTAACGCTCTTGTGTTTTTAGAAATGCTTAAAATTTTTGGG

CTTTTAAGCCAAGCGCACCATAACGATGTGTTAAAGATTTTAGAAAAAATACTTCAAAAT

>KH23

GTGAGTTTGATTAGGATTGATGATAGTAAAAAAGTAATTGAGGTTTCTATTCCTTTAACT

------------TCAATTTCAGGCAAAGTGCGTGTGAAAATCAGGCATGCCTTTAGCGAT

TATGGTATTTCAACAGCGACTAGAAAAATCCCTTTTAGTTTAAAGCATTATGTAGAGTGG

CAAATCGGTTATGATGTCCCCATTGAAGATAAAGAA---AAATTTGAGCTCACTACCCTA

AAAGATGAAAAATATCATTTTTTAGGGGCTAATAATAAAGTAAAAACTCTTTATGAATTA

AGCGAAATGATTTATTACGCTAAGCAATTAGGTTTAATCAGT---------TTAGAAAAT

TTAGAAAATACTTTAAAATATTTAGAAAAACAAAAACAATTTATAGAAGATAATTTCACG

ATTACAAGAGAAAGATTTAGATCGCATCAATTTGGGGGCATGGATTTTGAACTTTCACGC

ATTTCTTATCCCTTACTCATTCATTCTTTTAATGATAATCAATTGAGCGAAATCGTTATT

AGAGAACAACAATACGGCTCTAAGACACAAGCCATG---CTGTATTTTTGCTTTTCTATT

TTGGAGTTAAAAACCGCTACTCCCTTATTAAATAGAACCGCTACGCTCAAAGAACATGCC

CTTTTGATTGTCCATCAAACTAACGCTCTTGTGTTTTTAGAAATGCTTAAAATTTTTGGG

CTTTTAAGCCAAGCGCACCATAACGATGTGTTAAAGATTTTAGAAAAAATACTTCAAAAT

>HP04057

GTGAGTTTGATTAGGATTGATGATAGTAAAAAAGCGATTGAGGTTTCCATTCCTTTAACT

------------TCCATTTCAGGCAAAGTGCGTGTGAAAATCAGACATGCCTTTAGCGAT

TATGGTATTTCAACAGCGACTAGAAAAATCCCTTTTAGCTTAAAACATTATGTAGAGTGG

CAAATCGGTTATGATGTCCCCATTAAAGATAAAGAA---AAATTGGAGCTCACTACCCTA

AAAGATGAAAAATATCATTTTTTAGGGGCTAATAATAAAGTAAAAACCCTTTATGAATTG

AGTGAGATAATCTATTACGCTAAGCAATTAAATTTAATCAGT---------TTAGAAAAT

TTAGAAAATACTTTAAAATATTTAGAAAAACAAAAACAATTTATAGAAGATAATTTCACG

ATTACAAGAGAAAGATTTAGATCGCATCAATTTGGTGGCATGGATTTTGAACTTTCACGC

ATTTCTTATCCTTTACTCATTCATTCTTTTAATGATAATCAATTGAGTGAAATCGTTATT

AGAGAGCAACAATATGGCTCTAAAACCCAAGCCATG---CTGTATTTTTGCTTTTCTATT

TTGGAATTAAAAACCGCTACTCCCTTATTAAATAGAACGGCTGCACTCAAAGAACATGCC

CTTTTAACTATCCATAAAACCAACGCTCTCATGTTTTTAGAAATGCTTAAAATTTTTGGA

CTTTTAAGCCAAGTGCACCATAACGATGTGTTAAAGATTTTAGAAAAAATACTTCAAAAT

>KH12

GTGAGTTTGATTAGGATTGATAATAATAAAAAAGCGATTGAGGTTTCTATTCCTTTAACT

------------TCCATTTCAGGCAAAGTGCGTGTGAAAATCAGACATGCCTTTAGCGAT

TATGGTATTTCAACAGCGACTAGAAAAATCCCTTTTAGCTTAAAACATTATGTAGAGTGG

CAAATCGGTTATGATGTCCCCATTAAAGATAAAGAA---AAATTTGAGCTCACTACCCTA

AAAGATGAAAAATATCATTTTTTAGGGGCTAATAATAAAATGAAAACCCTTTATGAATTG

AGTGAGATAATCTATTACGCTAAGCAATTAAATTTAATCAGT---------TTAGAAAAT

TTAGAAAATACTTTAAAATATTTAGAAAAACAAAAACAATTTATAGAAGATAATTTCACG

ATTACAAGAGAAAGATTTAGATCGCATCAATTTGGTGGCATGGATTTTGAACTCTCACGC

ATTTCTTATCCTTTACTCATTCATTCTTTTAATGATAATCAATTGAGTGAAATCGTTATT

AGAGAGCAACAATATGGCTCTAAAACCCAAGCCATG---CTGTATTTTTGCTTTTCTATT

TTGGAATTAAAAACCGCTACCCCCTTATTAAATAGAACGGCTGCACTCAAAGAACATGCT

TTTTTAACCATCAATAAAACCAACGCTCTTATGTTTTTAGAAATGCTTAAAATTTTTGGA

CTTTTAAGCCAAGTGCACCATAACGATGTGTTAAAGATTTTAGAAAAAATACTTCAAAAT

>MHP16

GTGAGTTTGATTAGGATTGATGATAGTAAAAAAGCGATTGAGGTTTCTATTCCTTTAACT

------------TCAATTTCAGGCAAAGTGCGTGTGAAAATCAGACATGCCTTTAGCGAT

TATGGCATTTCAACAGCGACTAGAAAAATCCCTTTTAGTTTAAAACATTATGTAGAGTGG

CAAATCGGTTATGATGTCCCCATTAAAGATAAAGAA---AAATTTGAACTCACTACCCTA

AAAGATGAAAAATATCATTTTTTAGGGGCTAATAATAAAATAAAAACCCTTTATGAATTG

AGCGAAATAATTTATTACGCTAAGCGATTGGGTTTAATCAGT---------TTAGAAAAT

TTAGAAAATACTTTAAAATATTTAGAAAAACAAAAACAATTCATAGAAGATAGTTTTATG

ATCACAAGAGAAAGATTTAGATCGCATCAATTTGGGGGCATGGATTTTGAACTTTCACGC

ATTTCTTATCCCTTACTCATTCATTCTTTTAATGATAATCAATTGAGCGAAATCGTTATT

AGAGAGCAACAATACGGCTCTAAAACCCAAGCCATG---CTGTATTTTTGCTTTTCTATT

TTGGAGTTAAAAACCGCTACCCCCTTATTAAACAGAACGGCTATGCTCAAAGAGCATGCT

CTTTTGATTATCCATAAAACCAACGCTCTCATGTTTTTAGAAATGCTTAAAATTTTTGGA

CTTTTAAGCCAAGCGCACCATAACGATGTGTTAAAGATTTTAGAAAAAATACTTCAAAAT

>GC52-HL

GTGAGTTTGATTAGGATTGATGATAGTAAAAAAGCGATTGAGGTTTCTATTCCTTTAACT

------------TCAATTTCAGGCAAAGTGCGTGTGAAAATCAGGCATGCCTTTAGCGAT

TATGGCATTTCAACAGCGACTAGAAAAATCCCTTTTAGTTTAAAACATTATGTAGAGTGG

CAAATCGGTTATGATGTCCCCATTAAAGATAAAGAA---AAATTTGAACTCACTACCCTA

AAAGATGAAAAATATCATTTTTTAGGGGCTAATAATAAAATAAAAACCCTTTATGAATTG

AGCGAAATAATTTATTACGCTAAGCGATTGGGTTTAATCAGT---------TTAGAAAAT

TTAGAAAATACTTTAAAATATTTAGAAAAACAAAAACAATTCATAGAAGATAGTTTTATG

ATCACAAGAGAAAGATTTAGATCGCATCAATTTGGGGGCATGGATTTTGAACTTTCACGC

ATTTCTTATCCCTTACTCATTCATTCTTTTAATGATAATCAATTGAGCGAAATCGTTATT

AGAGAGCAACAATACGGCTCTAAAACCCAAGCCATG---CTGTATTTTTGCTTTTCTATT

TTGGAGTTAAAAACCGCTACCCCCTTATTAAACAGAACGGCTATGCTCAAAGAGCATGCC

CTTTTGATTATCCATAAAACCAACGCTCTCATGTTTTTAGAAATGCTTAAAATTTTTGGA

CTTTTAAGCCAAGCGCACCATAACGATGTGTTAAAGATTTTAGAAAAAATACTTCAAAAT

>2007

GTGAGTTTGATTAGGATTGATGATAGTAAAAAAGCGATTGAGGTTTCTATTCCTTTAACT

------------TCAATTTCAGGCAAAGTGCGTGTGAAAATCAGACATGCCTTTAGCGAT

TATGGCATTTCAACAGCGACTAGAAAAATCCCTTTTAGTTTAAAACATTATGTAGAGTGG

CAAATCGGTTATGATGTCCCCATTAAAGATAAAGAA---AAATTTGAACTCACTACCCTA

AAAGATGAAAAATATCATTTTTTAGGGGCTAATAATAAAATAAAAACCCTTTATGAATTG

AGCGAAATAATTTATTACGCTAAGCGATTGGGTTTAATCAGT---------TTAGAAAAT

TTAGAAAATACTTTAAAATATTTAGAAAAACAAAAACAATTCATAGAAGATAGTTTTATG

ATCACAAGAGAAAGATTTAGATCGCATCAATTTGGGGGCATGGATTTTGAACTTTCACGC

ATTTCTTATCCCTTACTCATTCATTCTTTTAATGATAATCAATTGAGCGAAATCGTTATT

AGAGAGCAACAATACGGCTCTAAAACCCAAGCCATG---CTGTATTTTTGCTTTTCTATT

TTGGAGTTAAAAACCGCTACCCCCTTATTAAACAGAACGGCTATGCTCAAAGAGCATGCT

CTTTTGATTATCCATAAAACCAACGCTCTCATGTTTTTAGAAATGCTTAAAATTTTTGGA

CTTTTAAGCCAAGCGCACCATAACGATGTGTTAAAGATTTTAGAAAAAATACTTCAAAAT

>ZH46

GTGAGTTTGATTAAGATTGATAATGATAAAAAAGTGATTGAGGTTTCTATTCCTTTAACT

------------TCCATTTCAGGCAAAGTGCGTGTGAAAATCAGACATGCCTTTAGCGAT

TATGGTATTTCAACAGCGACTAGAAAAATCCCTTTTAGTTTAAAACATTATGTAGAGTGG

CAAATCGGTTATGATGTCCCCATTAAAGATAAAGAA---AAATTTGAGCTCACTACTTTA

AAAGATGAAAAATATCATTTTTTAGGGGCTAATAATAAAGTAAAAACCCTTTATGAATTG

AGCGAAATAATTTATTACGCTAAGCGATTGGGTTTAATCAGT---------TTAGAAAAT

TTAGAAAATACTTTAAAATATTTAGAAAAACAAAAACAATTCATAGAAGATAGTTTTATA

ATCACAAGAGAAAGATTTAGATCCCATCAATTTGGTGGCATGGATTTTGAACTTTCACGC

ATTTCTTATCCCTTACTCATTCATTCTTTCAACGATAATCAGTTGAGCGAAATTGTTATT

AGGGAACAACAATACGGCTCTAAAACCCAAGCCATG---CTGTATTTTTGTTTTTCTATT

TTGGAGTTAAAAACCACTACCCCCTTATTAAATAGAACGGCTGCACTCAAAGAACATGCC

CTTTTAACTATCCATAAAACCAACGCTCTTGTGTTTTTAGAAATGCTTAAAATTTTTGGA

TTTTTAAGCCAAGCGCACCATAACGATGTGTTAAAGATTTTAGAAAAAATACTTCAAAAT

>HP13024

GTGAGTTTGATTAAAGTTAATGATGATAAAAAAGTGATTGAGGTTTCTATTCCTTTAACT

------------TCAATTTCAGGCAAAGTGCGTGTGAAAATCAGACATGCCTTTAGCGAT

TATGGTATTTCAACAGCGACTAGAAAAATCCCTTTTAGCTTAAAACATTATGTAGAGTGG

CAAATCGGTTATGATGTCCCCATTAAAGATAAAGAA---AAATTTGAGCTCACTACCCTA

AAAGATGAAAAATATCATTTTTTAGGGGCTAATAATAAAGTAAAAACTCTTTATGAATTG

AGTGAAATAATTGATTACGCTAAGCAATTAGGTTTAATCAGT---------TTAGAAAAT

TTAGAAAATACTTTAAAATATTTAGAAAAACAAAAACAATTTATAGAAGATAATTTTATG

ATTACAAGAGAAAGATTTAGATCGCATCAATTTGGTGGCATGGATTTTGAACTCTCACGC

ATTTCTTATCCTTTACTCATTCATTCTTTTGATGATAATCAATTGAGCGAAATCGTTATT

AGGGAACAACAATATGGCTCTAAAACCCAAGCCATG---CTGTATTTTTGCTTTTCTATT

TTGGAATTAAAAACCGCTACCCCCTTATTAAACAGAACGGCTATGCTCAAAGAGCATGCC

CTTTTGATTATCCATAAAACCAACGCTCTCATGTTTTTAGAAATGCTTAAAATTTTTGGA

CTTTTAAGCCAAGCGCACCATAACGATGTGTTAAAGATTTTAGAAAAAATACTTCAAAAT

>ZH131

GTGAGTTTGATTGAGATTGATAATAATAAAAAAGTAATTGAGATTTCTATTCCTTTAACT

------------TCAATTTCAGGCAAAGTGCGTGTGAAAATCAGACATGCCTTTAGCGAT

TATGGTATTTCAACAGCGACTAGAAAAATCCCTTTTAGCTTAAAACATTATGTAGAGTGG

CAAATCGGTTATGATGTCCCCATTAAAGATAAAGAA---AAATTTGAGCTCACTACCCTA

AAAGATGAAAAATATCATTTTTTAGGGGCTAATAATAAAGTAAAAACCCTTTATGAATTG

AGTGAGATAATCTATTACGCTAAGCAATTAAATTTAATCAGT---------TTAGAAAAT

TTAGAAAATACTTTAAAATATTTAGAAAAACAAAAACAATTCATAGAAGATAATTTTATG

ATTACAAGAGAAAGATTTAGATTGCATCAATTTGGTAGCATGGATTTTGAACTCTCACGT

ATTTCTTATCCTTTACTCATTCATTCTTTTAATGATAATCAGTTGAGCGAAATCGTTATT

AGAGAGCAACAATACGGCTCTAAAACCCAAGCCATG---CTGTATTTTTGCTTTTCTATT

CTGGAATTAAAAACCGCTACCCCTTTATTAAATAGAACGGCTGCACTCAAAGAACATGCT

TTTTTAATTATCCATAAAACCAACGCTCTCATGTTTTTAGAAATGCTTAAAATTTTTGGG

CTTTTAAGCCAAGCGCACCATAACGATGTGTTAAAGATTTTAGAAAAAATACTTCAAAAT

>MHP31

GTGAGTTTGATTAAAGTTAATGATAATAAAAAAGTGATTGAGGTTTCTATTCCTTTAACT

------------TCCATTTCAGGCAAAGTTCGTGTGAAAATCAGGCACGCCTTTAGCGAT

TATGGCATTTCAACAGCGACTAGAAAAATCCCTTTCAGTTTAAAGCATTATGTAGAGTGG

CAAATCGGTTATGATGTCCCCATTAAAGATAAAGAA---AAATTTGAACTCACTACCCTA

AAAGATGAAAAATATCATTTTTTAGGGGCTAATAATAAAGTAAAAACCCTTTATGAATTG

AGCGAAATAATTGATTACGCTAAGCGATTGGGTTTAATCAGT---------TTAGAAAAT

TTAGAAAATACTTTAAAATATTTAGAAAAACAAAAACAATTTATAGAAGATAATTTTATG

ATTACAAGAGAAAGATTTAGATCGCATCAATTTGGTGGCATGGATTTTGAACTTTCACGC

ATTTCTTATCCTTTACTCATTCATTCTTTTAATGATAACCAGTTGAGTGAAATCGTTATT

AGAGAGCAACAATATGGCTCTAAAACCCAAGCCATG---CTGTATTTTTGCTTTTCTATT

CTGGAATTAAAAACCGCTACACCCTTATTAAATAGAACGGCTGCACTCAAAGAACATGCC

CTTTTAACTATCCATAAAACCAACGCTCCCATGTTTTTAGAAATGCTTAAAATTTTTGGA

CTTTTAAGCCAAGCGCACCATAACGATGTGTTAAAGATTTTAGAAAAAATACTTCAAAAT

>31235

GTGAGTTTGATTAGGATTGATGATAGTAAAAAAGCGATTGAGGTTTCCATTCCTTTAACT

------------TCAATTTCAGGCAAAGCGCGTGTGAAAATCAGACATGCCTTTAGCGAT

TATGGCATTTCAACAGCGACCAGAAAAATCCCTTTTAGTTTAAAGCATTATGTAGAGTGG

CAAATCGGTTATGATGTCCCTATTAAAGATAAAGAA---AAATTTAAACTCACTACTTTA

AAAGATGAAAAATACCATTTTTTAGGGGCTAATAATAAAGTAAAAACTCTTTATGAATTG

AGCGAAATGATTTATTACGCTAATCAATTAGGTTTAATCAGT---------TTAGAAAAT

TTAGAAAATACTTTAAAATATTTAGAAAAACAAAAACAATTTATAGAAGATAATTTTATG

ATCATAAGAGAAAGATTCAGATCGCATCAATTTGGTGGCATGGATTTTGAACTCTCACGC

ATTTCTTATCCTTTGCTCATTCATTCTTTTGATGATAATCAATTGAGCGAAATTGTTATT

AGAGAACAACAATATGGCTCTAAAACCCAAGCCATG---CTCTATTTTTGCTTTTCTATT

CTGGAGTTAAAAACCGCTACCCCTTTATTAAATAGAACCGCTACACTCAAAGAACATGCT

TTTTTAACCATCCATAAAACCAACGCTCTTATGTTTTTAGAAATGCTTAAAATTTTTGGA

CTTTTAAGCCAAGCGCACCATAGCGATGTGTTAAAGATTTTAGAAAAAATACTTCAAAAT

>HP99316

GTGAGTTTGATTAAAGTTAGTGGTGATAAAAAAGTGATTGAGGTTTCTATTCCTTTAACT

------------TCAATTTCAGGCAAAGTGCGTGTGAAAATCAGACATGCCTTTAGCGAT

TATGGTATTTCAACAGCGACTAGAAAAATCCCTTTTAGTTTAAAACATTATGTAGAGTGG

CAGATCGGTTATGATGTCCCCATTAAAGATAAAGAA---AAATTTGAACTCACTACTTTA

AAAGATGAAAAATATCATTTTTTAGGGGCTAATAATAAAGTGAAAACTCTTTATGAATTA

AGTGAAATGATTTATTACGCTAAGCAATTAGATTTAATCAGT---------TTAGAAAAT

TTAGAAAATACTTTAAAATATTTAGAAAAACAAAAACAATTTATAGAAGATAATTTTATG

ATTACAAGAGAAAGATTTAGATTGCATCAATTTGGTGGCATGGATTTTGAACTCTCACGC

ATTTCTTATCCTTTGCTCATTCATTCTTTTAATGATAATCAATTGAGCGAAATTGTTATT

AGGGAACAACAATATGGTTCTAAAACCCAAGCCATG---CTGTATTTTTGCTTTTCTATT

TTGGAGTTAAAAACCGCTACCCCCTTATTAAACAGAACCGCTACGCTCAAAGAACATGCC

CTTTTAACTATCCATAAAACCAACGCTCTCATGTTTTTAGAAATGCTTAAAATTTTTGGA

CTTTTAAGCCAAGCGCACCATAACGATGTGTTAAAGATTTTAGAAAAAATACTTCAAAAT

>M-Mx-2005-152

GTGAGTTTGATTAGGATTGATAATAATAAAAAAGTAATTGGGGTTTCTATTCCTTTAACT

------------TCAATTTCAGGCAAAGTGCGTGTGAAAATCAGACATGCCTTTAGCGAT

TATGGTATTTCAACAGCGACCAGAAAAATCCCTTTTAGCTTAAAACATTATGTAGAGTGG

CAAATCGGTTATGATGTCCCCATTAAAGATAAAGAA---AAATTTGAGCTCACTACTTTA

AAAGATGAAAAATATCATTTTTTAGGGGCTAATAATAAAGTAAAAACCCTTTATGAATTG

AGTGAGATAATCTATTACGCTAAGCAATTAAATTTAATCAGT---------TTAGAAAAT

TTAGAAAATACTTTAAAATATTTAGAAAAACAAAAACAATTTATAGAAGATAATTTCACG

ATTACAAGAGAAAGATTTAGATTACATCAATTTGGTGGCATGGATTTTGAACTCTCACGC

ATTTCTTATCCTTTGCTCATTCATTCTTTTAATGATAATCAGTTGAGCGAAATCGTTATT

AGAGAACAACAATATGGCTCTAAAACCCAAGCCATG---CTGTATTTTTGCTTTTCTATT

CTGGAATTAAAAACCGCTACCCCTTTATTAAATAGAACGGCTGCACTCAAAGAACATGCT

CTTTTGATTATCCATAAAACCAACGCTCTTGTGTTTTTAGAAATGCTTAAAATTTTTGGA

CTTTTAAGCCAAGCGCACCATAACGATGTGTTAAAGATTTTAGAAAAAATACTTCAAAAT

>Nic53-A

GTGAGTTTGATTAGGATTGATGATAGTAAAAAAGCGATTGAGGTTTCTGTTCCTTTAACT

------------TCCATTTCAGGCAAAGTGCGTGTGAAAATCAGACATGCCTTTAGCGAT

TATGGCATTTCAACAGCGACTAGAAAAATCCCTTTTAGTTTAAAACATTATGTAGAGTGG

CAAATCGGTTATGATGTCCCCATTAAAGATAAAGAA---AAATTTGAACTCACTACCCTA

AAAGATGAAAAATATCATTTTTTAGGGGCTAATAATAAAATAAAAACCCTTTATGAATTG

AGCGAAATAATTTATTACGCTAAGCGATTGGGTTTAATCAGT---------TTAGAAAAT

TTAGAAAATACTTTAAAATATTTAGAAAAACAAAAACAATTCATAGAAGATAATTTTATG

ATCACAAGAGAAAGATTTAGATCGCATCAATTTGGGGGCATGGATTTTGAACTTTCACGC

ATTTCTTATCCCTTACTCATTCATTCTTTTAATGATAATCAATTGAGCGAAATCGTTATT

AGAGAGCAACAATACGGCTCTAAAACCCAAGCCATG---CTGTATTTTTGCTTTTCTATT

TTGGAGTTAAAAACCGCTACCCCCTTATTAAACAGAACGGCTATGCTCAAAGAGCATGCT

CTTTTGATTATCCATAAAACCAACGCTCTTGTGTTTTTAGAAATGCTTAAAATTTTTGGA

CTTTTAAGCCAAGCGCACCATAACGATGTGTTAAAGATTTTAGAAAAAATACTTCAAAAT

>G-Mx-2006-152

GTGAGTTTGATTAGGATTGATAATAATAAAAAAGTAATTGGGGTTTCTATTCCTTTAACT

------------TCAATTTCAGGCAAAGTGCGTGTGAAAATCAGACATGCCTTTAGCGAT

TATGGTATTTCAACAGCGACCAGAAAAATCCCTTTTAGCTTAAAACATTATGTAGAGTGG

CAAATCGGTTATGATGTCCCCATTAAAGATAAAGAA---AAATTTGAGCTCACTACTTTA

AAAGATGAAAAATATCATTTTTTAGGGGCTAATAATAAAGTAAAAACCCTTTATGAATTG

AGTGAGATAATCTATTACGCTAAGCAATTAAATTTAATCAGT---------TTAGAAAAT

TTAGAAAATACTTTAAAATATTTAGAAAAACAAAAACAATTTATAGAAGATAATTTCACG

ATTACAAGAGAAAGATTTAGATTACATCAATTTGGTGGCATGGATTTTGAACTCTCACGC

ATTTCTTATCCTTTGCTCATTCATTCTTTTAATGATAATCAGTTGAGCGAAATCGTTATT

AGAGAACAACAATATGGCTCTAAAACCCAAGCCATG---CTGTATTTTTGCTTTTCTATT

CTGGAATTAAAAACCGCTACCCCTTTATTAAATAGAACGGCTGCACTCAAAGAACATGCT

CTTTTGATTATCCATAAAACCAACGCTCTTGTGTTTTTAGAAATGCTTAAAATTTTTGGA

CTTTTAAGCCAAGCGCACCATAACGATGTGTTAAAGATTTTAGAAAAAATACTTCAAAAT

>173-A-EK1

GTGAATTTGATTAAGATTGATAATAATAAAAAAGTAATTGAGATTTCTGTTCCTTTAACT

------------TCAATTTCAGGCAAAGTGCGTGTGAAAATCAGACATGCCTTTAGCGAT

TATGGCATTTCAACAGCGACTAGAAAAATCCCTTTTAGTTTAAAGCATTATGTAGAGTGG

CAAATCGGTTATGATGTCCCTATTAAAGATAAAGAA---AAATTTGAACTCACTACTTTA

AAAGATGAAAAATATCATTTTTTAGGGGCTAATAATAAAGTAAAGACTCTTTATGAATTG

AGTGAGATAATCTATTACGCTAAGCAATTAAATTTAATCAGT---------TTAGAAAAT

TTAGAAAATACTTTAAAATATTTAGAAAAACAAAAACAATTTATAGAAGATAATTTTATG

ATTACAAGAGAAAGATTTAGATTACATCAATTTGGTGGCATGGATTTTGAACTCTCACGC

ATTTCTTATCCCTTACTCATTTATTCTTTTAATGATAATCAGTTGAGCGAAATTGTTATT

AGAGAACAACAATATGGCTCTAAAACCCAAGCCATG---CTGTATTTTTGCTTTTCTATT

TTGGAGTTAAAAACTGCTACCCCCTTATTAAACAGAACGGCTGCACTCAAAGAACATGCC

CTTTTAACTATCCATAAAACCAACGCTCCCATGTTTTTAGAAATGCTTAAAATTTTTGGA

CTTTTAAGCCAAGCGCACCATAACGATGTGTTAAAGATTTTAGAAAAAATACTTCAAAAT

>Nic52-C

GTGAGTTTGATTAAAGTTAATGATGATAAAAAAGTGATTGAGGTTTCTATTCCTTTAACT

------------TCCATTTCAGGCAAAGTTCGTGTGAAAATCAGACATGCCTTTAGCGAT

TATGGCATTTCAACAGCGACTAGAAAAATCCCTTTCAGTTTAAAGCATTATGTAGAGTGG

CAAATCGGTTATGATGTCCCCATTAAAGATAAAGAA---AAATTTGAACTCACTACCCTA

AAAGATGAAAAATATCATTTTTTAGGGGCTAATAATAAAATAAAAACCCTTTATGAATTG

AGCGAAATGATTTATTACGCTAAGCGATTGGGTTTAATCAGT---------TTAGAAAAT

TTAGAAAATACTTTAAAATATTTAGAAAAACAAAAACAATTCATAGAAGATAGTTTTATG

ATCACAAGAGAAAGATTTAGATCGCATCAATTTGGTGGCATGGATTTTGAACTTTCACGC

ATCTCTTACCCTTTACTCATTCATTCTTTTAATGATAATCAGTTGAGCGAAATCGTTATT

AGAGAGCAACAATACGGCTCTAAAACCCAAGCCATG---CTATATTTTTGCTTTTCTATT

CTGGAATTAAAAACCGCTACCCCCTTATTAAATAGAACCGCTGCCCTCAAAGAACATGCC

CTTTTAACTATCCATGAAACTAACGCTCTTGTGTTTTTAGAAATGCTTAAAATTTTTGGC

CTTTTAAGCCAAGCGCACCATAACGATGTGTTAAAGATTTTAGAAAAAATACTTGAAAAT

>25b2

GTGAGTTTGATTAAAGTTGACTATGATAAAAAAGTGATTGAGGTTTCTATTCCTTTAACT

------------TCCATTTCAGGCAAAGTGCGTGTGAAAATCAGACATGCCTTTAGCGAT

TATGGTATTTCAACAGCGACTAGAAAAATCCCTTTTAGTTTAAAGCATTATGTAGAGTGG

CAAATCGGTTATGATGTCCCCATTAAAGATAAAGAA---AAATTGGAGCTCACTACCCTA

AAAGATGAAAAATATCATTTTTTAGGGGCTAATAATAAAGTAAAAACTCTTTATGAATTG

AGCGAAATGATTTATTACGCTAAGCAATTAGGTTTAATCAGT---------TTAGAAAAT

TTAGAAAATACTTTAAAATATTTAGAAAAACAAAAACAATTTATAGAAGATAATTTTATG

ATTACAAGAGAAAGATTCAGATCGCATCAATTTGGTGGCATGGATTTTGAACTTTCACGC

ATTTCTTATCCTTTACTCATTCATTCTTTTAACGATAATCAGTTGAGCGAAATCGTTATT

AGAGAGCAACAATACGACTCTAAGACCCAAGCTATG---CTGTATTTTTGCTTTTCTATT

TTGGAGTTAAAAACCGCTACTCCCTTATTAAACAGAACCGCTACGCTCAAAGAGCATGCT

CTTTTGATTATCCATAAAACCAACGCTCTCATGTTTTTAGAAATGCTTAAAATTTTTGGG

CTTTTAAGCCAAGCGCACCATAACGATGTGTTAAAGATTTTAGAAAAAATACTTCAAAAT

>59a9

GTGAGTTTGATTAAAGTTGACTATGATAAAAAAGTGATTGAGGTTTCTATTCCTTTAACT

------------TCCATTTCAGGCAAAGTGCGTGTGAAAATCAGACATGCCTTTAGCGAT

TATGGTATTTCAACAGCGACTAGAAAAATCCCTTTTAGTTTAAAGCATTATGTAGAGTGG

CAAATCGGTTATGATGTCCCCATTAAAGATAAAGAA---AAATTGGAGCTCACTACCCTA

AAAGATGAAAAATATCATTTTTTAGGGGCTAATAATAAAGTAAAAACTCTTTATGAATTG

AGCGAAATGATTTATTACGCTAAGCAATTAGGTTTAATCAGT---------TTAGAAAAT

TTAGAAAATACTTTAAAATATTTAGAAAAACAAAAACAATTTATAGAAGATAATTTTATG

ATTACAAGAGAAAGATTCAGATCGCATCAATTTGGTGGCATGGATTTTGAACTTTCACGC

ATTTCTTATCCTTTACTCATTCATTCTTTTAACGATAATCAGTTGAGCGAAATCGTTATT

AGAGAGCAACAATACGACTCTAAGACCCAAGCTATG---CTGTATTTTTGCTTTTCTATT

TTGGAGTTAAAAACCGCTACTCCCTTATTAAACAGAACCGCTACGCTCAAAGAGCATGCT

CTTTTGATTATCCATAAAACCAACGCTCTCATGTTTTTAGAAATGCTTAAAATTTTTGGG

CTTTTAAGCCAAGCGCACCATAACGATGTGTTAAAGATTTTAGAAAAAATACTTCAAAAT

>MCms931

GTGAGTTTGATTAGGATTGATAATAATAAAAAAGTAATTGGGGTTTCTATTCCTTTAACT

------------TCAATTTCAGGCAAAGCGCGTGTGAAAATCAGACATGCCTTTAGCGAT

TATGGCATTTCAACAGCGACCAGAAAAATCCCTTTTAGTTTAAAGCATTATGTAGAGTGG

CAAATCGGTTATGATGTCCCTATTAAAGATAAAGAA---AAATTTAAACTCACTACTTTA

AAAGATGAAAAATACCATTTTTTAGGGGCCAATAATAAAGTAAAAACTCTTTATGAATTG

AGTGAAATAATTTATTACGCTAAGCAATTAAATTTAATCAGT---------TTAGAAAAT

TTAGAAAATACTTTAAAATATTTAGAAAAACAAAAACAATTCATAGAAGATAGTTTTATG

ATCACAAGAGAAAGATTTAGATCGCATCAATTTGGGGGCATGGATTTTGAACTCTCACGC

ATTTCTTATCCTTTACTCATTCATTCTTTTAATGATAATGAGTTGAGCGAAATCGTTATT

AGAGAACAACAATACGGCTCTAAAACCCAAGCCATG---CTGTATTTTTGCTTTTCTATT

TTGGAGTTAAAAACCGCTACCCCCTTATTAAATAGAACGGCTGCACTCAAAGAGCACGCT

CTTTTAACTATCCATAAAACTAACGCTCTTATGTTTTTAGGAATGCTTAAAATTTTTGGA

CTTTTAAGCCAAGCGCACCATAACGATGTGTTAAAGATTTTAGAAAAAATACTTCAAAAT

>C-Mx-2010-2

GTGAGTTTGATTAGGATTGATAATAATAAAAAAGTAATTGGGGTTTCTATTCCTTTAACT

------------TCAATTTCAGGCAAAGCGCGTGTGAAAATCAGACATGCCTTTAGCGAT

TATGGCATTTCAACAGCGACCAGAAAAATCCCTTTTAGTTTAAAGCATTATGTAGAGTGG

CAAATCGGTTATGATGTCCCTATTAAAGATAAAGAA---AAATTTAAACTCACTACTTTA

AAAGATGAAAAATACCATTTTTTAGGGGCCAATAATAAAGTAAAAACTCTTTATGAATTG

AGTGAAATAATTTATTACGCTAAGCAATTAAATTTAATCAGT---------TTAGAAAAT

TTAGAAAATACTTTAAAATATTTAGAAAAACAAAAACAATTCATAGAAGATAGTTTTATG

ATCACAAGAGAAAGATTTAGATCGCATCAATTTGGGGGCATGGATTTTGAACTCTCACGC

ATTTCTTATCCTTTACTCATTCATTCTTTTAATGATAATGAGTTGAGCGAAATCGTTATT

AGAGAACAACAATACGGCTCTAAAACCCAAGCCATG---CTGTATTTTTGCTTTTCTATT

TTGGAGTTAAAAACCGCTACCCCCTTATTAAATAGAACGGCTGCACTCAAAGAGCACGCT

CTTTTAACTATCCATAAAACTAACGCTCTTATGTTTTTAGGAATGCTTAAAATTTTTGGA

CTTTTAAGCCAAGCGCACCATAACGATGTGTTAAAGATTTTAGAAAAAATACTTCAAAAT

>499/02

GTGAGTTTGATTAGGATTGATGATAGTAAAAAAGCGATTGAGGTTTCTATTCCTTTAACT

------------TCAATTTCAGGCAAAGTGCGTGTGAAAATCAGACATGCCTTTAGCGAT

TATGGTATTTCAACAGCGACTAGAAAAATCCCTTTTAGTTTAAAACATTATGTAGAGTGG

CAAATCGGTTATGATGTCCCCATTAAAGATAAAGAA---AAATTTGAGCTCACTACCCTA

AAAGATGAAAAATATCATTTTTTAGGGGCTAATAATAAAGTAAAAACCCTTTATGAATTG

AGTGAGATAATCTATTACGCTAAGCGATTTGGTTTAATCAGT---------TTAGAAAAT

TTAGAAAATACTTTAAAATATTTAGAAAAACAAAAACAATTTATAGAAGATAATTTCACG

ATTACAAGAGAAAGATTTAGATCGCATCAATTTGGTGGCATGGATTTTGAACTTTCACGC

ATTTCTTATCCCTTACTCATTCATTCTTTTAATGATAATCAGTTGAGCGAAATCGTTATT

AGAGAGCAACAATACGGCTCTAAAACCCAAGCCATG---CTGTATTTTTGCTTTTCTATT

CTGGAGTTAAAAACCGCTACACCCTTATTAAATAGGACTGTTGCCCTCAAAGAACATGCC

CTTTTAACTATCCATAAAACCAACGCTCTTGTGTTTTTAGAAATGCTTAAAATTTTTGGA

CTTTTAAGCCAAGCGCACCATAACGATGTGTTAAAGATTTTAGAAAAAATACTTCAAAAT

>B274

GTGAGTTTGATTAAGATTGATAATGATAAAAAAGTGATTGAGGTTTCTATTCCTTTAACT

------------TCCATTTCAGGCAAAGCGCGTGTGAAAAACAGACATGCCTTTAGCGAT

TATGGCATTTCAACAGCGACCAGAAAAATCCCTTTTAGTTTAAAGCATTATGTAGAGTGG

CAGATCGGTTATGATGTCCCCATTAAAGATAAAGAA---AAATTTGAACTCACTACTTTA

AAAGATGAAAAATATCATTTTTTAGGGTCTAATGATAAAGTAAAAACTCTTTATGAATTG

AGTGAAATGATTTATTACACTAAGCAATTAGGTTTAATCAGT---------TTAGAAAAT

TTAGAAAATACTTTAAAATATTTAGAAAAACAAAAACAATTTATAGAAGATAATTTTATG

ATTACAAGAGAAAGATTTAGATCGCATCAATTTGGTGGCGTGGATTTTGAACTTTCACGC

ATCTCTTATCCTTTACTCATTCATTCTTTTAATGATAATCAGTTGAGCGAAATCGTTATT

AGAGAGCAACAATATGGCTCTAAAACCCAAGCCATG---CTGTATTTTTGCTTTTCTATT

TTGGAATTAAAAACCGCTACTCCCTTATTAAATAGAACCGCTACACTCAAAGAACATGCC

CTTTTAACTATCCATAAAACCAACGCTCTTGTGTTTTTAGAAATGCTTAAAATTTTTGGA

CTTTTAAGCCAAGCGTACCATAACGATGTGTTAAAGATTTTAGAAAAAATACTTCAAAAT

>565-99

GTGAGTTTGATTAGGATTGATAATAATAAAAAAGTAATTGGGGTTTCTATTCCTTTAACT

------------TCAATTTCAGGCAAAGTGCGTGTGAAAATCAGACATGCCTTTAGCGAT

TATGGTATTTCAACAGCGACTAGAAAAATCCCTTTTAGCTTAAAACATTATGTAGAGTGG

CAAATCGGTTATGATGTCCCCATTAAAGATAAAGAA---AAATTGGAGCTCACTACCCTA

AAAGATGAAAAATATCATTTTTTAGGGGCTAATAATAAAGTAAAAACCCTTTATGAATTG

AGTGAGATAATCTATTACGCTAAGCAATTAAATTTAATCAGT---------TTAGAAAAT

TTAGAAAATACTTTAAAATATTTAGAAAAACAAAAACAATTTATAGAAGATAATTTCACG

ATTACAAGAGAAAGATTTAGATCGCATCAATTTGGTGGCATGGATTTTGAACTTTCACGC

ATTTCTTATCCTTTACTCATTCATTCTTTTAATGATAATCAATTGAGTGAAATCGTTATT

AGAGAGCAACAATATGGCTCTAAAACCCAAGCCATG---CTGTATTTTTGCTTTTCTATT

TTGGAATTAAAAACCACTACCCCCTTATTAAATAGAACGGCTACACTCAAAGAACATGCC

CTTTTAACTATCCATAAAACCAACGCTCTTGTGTTTTTAGAAATGCTTAAAATTTTTGGA

CTTTTAAGCCAAGCGCACCATAACGATGTGTTAAAGATTTTAGAAAAAATACTTCAAAAT

>HP14021

GTGAGTTTGATTGAGATTGATGATGATAAAAAAGTAATTGAGGTTTCTATTCCTTTAACT

------------TCAATTTCAGGCAAAGTGCGTGTGAAAATCAGACATGCCTTTAGCGAT

TATGGTATTTCAACAGCGACCAGAAAAATCCCTTTTAGTTTAAAGCATTATGTAGAGTGG

CAAATCGGTTATGATGTCCCCATTAAAGATAAAGAA---AAATTGGAGCTCACTACTTTA

AAAGATGAAAAATATCATTTTTTAGGGGCTAATAATAAAGTAAAAACTCTTTATGAATTG

AGCGAAATGATTTATTACGCTAAGCAATTAGGTTTAATCAGT---------TTAGAAAAT

TTAGAAAATACTTTAAAATATTTAGAAAAACAAAAACAATTTATAGAAGATAATTTTATG

ATTACAAGAGAAAGATTTAGATCGCATCAATTTGATGGCATGGATTTTGAACTTTCACGC

ATCTCTTACCCTTTACTCATTCATTCTTTTAACGATAATCAATTGAGTGAAATCGTTATT

AGAGAACAACAATACGGCTCTAAAACCCAAGCCATG---CTGTATTTTTGCTTTTCTGTT

TTGGAATTAAAAACCACTACCCCCTTATTAAACAGAACCGCTGCACTCAAAGAACATGCT

TTTTTAACCATCCATAAAACCAACGCTCTTGTGTTTTTAGAAATGCTTAAAATTTTTGGA

CTTTTAAGCCAAGCGCACCATAACGATGTGTTAAAGATTTTAGAAAAAATACTTCAAAAT

>MHP08

GTGAGTTTGATTAGGATTGATGATAGTAAAAAAGCGATTGAGGTTTCTATTCCTTTAACT

------------TCAATTTCAGGCAAAGTGCGTGTGAAAATCAGACATGCCTTTAGCGAT

TATGGTATTTCAACAGCGACTAGAAAAATCCCTTTTAGCTTAAAACATTATGTAGAGTGG

CAAATCGGTTATGATGTCCCCATTAAAGATAAAGAA---AAATTTGAGCTCACTACCCTA

AAAGATGAAAAATATCATTTTTTAGGGGCTAATAATAAAGTAAAAACCCTTTATGAATTG

AGTGAGATAATCTATTACGCTAAGCAATTAAATTTAATCAGT---------TTAGAAAAT

TTAGAAAATACTTTAAAATATTTAGAAAAACAAAAACAATTCATAGAAGATAATTTCACG

ATTACAAGAGAAAGATTTAGATCGCATCAATTTGGGGGCATGGATTTTGAACTCTCACGC

ATTTCTTATCCCTTACTCATTCATTCTTTTAATGATAATCAGTTGAGCGAAATTGTTATT

AGAGAACAACAATATGGCTCTAAAACCCAAGCCATG---CTGTATTTTTGCTTTTCTATT

TTGGAGTTAAAAACCGCTACCCCCTTATTAAACAGAACGGCTATGCTCAAAGAGCATGCT

CTTTTGATTATCCATAAAACCAACGCTCTCATGTTTTTAGAGATGCTTAAAATTTTTGGG

CTTTTAAGCCAAGCGCACCATAACGATGTGTTAAAGATTTTAGAAAAAATACTTCAAAAT

>MHP09C

GTGAGTTTGATTAGGATTGATGATAGTAAAAAAGCGATTGAGGTTTCTATTCCTTTAACT

------------TCAATTTCAGGCAAAGTGCGTGTGAAAATCAGACATGCCTTTAGCGAT

TATGGTATTTCAACAGCGACTAGAAAAATCCCTTTTAGCTTAAAACATTATGTAGAGTGG

CAAATCGGTTATGATGTCCCCATTAAAGATAAAGAA---AAATTTGAGCTCACTACCCTA

AAAGATGAAAAATATCATTTTTTAGGGGCTAATAATAAAGTAAAAACCCTTTATGAATTG

AGTGAGATAATCTATTACGCTAAGCAATTAAATTTAATCAGT---------TTAGAAAAT

TTAGAAAATACTTTAAAATATTTAGAAAAACAAAAACAATTCATAGAAGATAATTTCACG

ATTACAAGAGAAAGATTTAGATCGCATCAATTTGGGGGCATGGATTTTGAACTCTCACGC

ATTTCTTATCCCTTACTCATTCATTCTTTTAATGATAATCAGTTGAGCGAAATTGTTATT

AGAGAACAACAATATGGCTCTAAAACCCAAGCCATG---CTGTATTTTTGCTTTTCTATT

TTGGAGTTAAAAACCGCTACCCCCTTATTAAACAGAACGGCTATGCTCAAAGAGCATGCT

CTTTTGATTATCCATAAAACCAACGCTCTCATGTTTTTAGAGATGCTTAAAATTTTTGGG

CTTTTAAGCCAAGCGCACCATAACGATGTGTTAAAGATTTTAGAAAAAATACTTCAAAAT

>MHP20

GTGAGTTTGATTAGGATTGATGATAGTAAAAAAGCGATTGAGGTTTCTATTCCTTTAACT

------------TCAATTTCAGGCAAAGTGCGTGTGAAAATCAGACATGCCTTTAGCGAT

TATGGTATTTCAACAGCGACTAGAAAAATCCCTTTTAGCTTAAAACATTATGTAGAGTGG

CAAATCGGTTATGATGTCCCCATTAAAGATAAAGAA---AAATTTGAGCTCACTACCCTA

AAAGATGAAAAATATCATTTTTTAGGGGCTAATAATAAAGTAAAAACCCTTTATGAATTG

AGTGAGATAATCTATTACGCTAAGCAATTAAATTTAATCAGT---------TTAGAAAAT

TTAGAAAATACTTTAAAATATTTAGAAAAACAAAAACAATTCATAGAAGATAATTTCACG

ATTACAAGAGAAAGATTTAGATCGCATCAATTTGGGGGCATGGATTTTGAACTCTCACGC

ATTTCTTATCCCTTACTCATTCATTCTTTTAATGATAATCAGTTGAGCGAAATTGTTATT

AGAGAACAACAATATGGCTCTAAAACCCAAGCCATG---CTGTATTTTTGCTTTTCTATT

TTGGAGTTAAAAACCGCTACCCCCTTATTAAACAGAACGGCTATGCTCAAAGAGCATGCT

CTTTTGATTATCCATAAAACCAACGCTCTCATGTTTTTAGAGATGCTTAAAATTTTTGGG

CTTTTAAGCCAAGCGCACCATAACGATGTGTTAAAGATTTTAGAAAAAATACTTCAAAAT

>ZH108

GTGAGTTTGATTAAAGTTAGTGGTGATAAAAAAGTGATTGAGGTTTCTATTCCTTTAACT

------------TCAATTTCAGGCAAAGTGCGTGTGAAAATCAGACATGCCTTTAGCGAT

TATGGTATTTCAACAGCGACTAGAAAAATCCCTTTTAGTTTAAAACATTATATAGAGTGG

CAGATCGGTTATGATGTCCCTATTAAAGATAAAGAA---AAATTTGAACTCACTACTTTA

AAAGATGAAAAATATCATTTTTTAGGGGCTAATGATAAAGTAAAAACTCTTTATGAATTA

AGCGAAATGATTTATTACGCTAAACAATTAGGTTTAATCAGT---------TTAGAAAAT

TTAGAAAATACTTTAAAATATTTAGAAAAACAAAAACAATTTATAGAAGATAATTTTATG

ATTACAAGAGAAAGATTTAGATTACATCAATTTGGTGGCATGGATTTTGAACTCTCATGC

ATTTCTTATCCCTTACTCATTCATTCTTTTAATGATAATGAGTTGAGCGAAATCGTTATT

AGAGAGCAACAATATGGCTCTAAAACCCAGGCCATG---CTGTATTTTTGCTTTTCTATT

TTGGAATTAAAAACCGCTACTCCCTTATTAAATAGAACGGCTGCACTCAAAGAACATGCC

CTTTTAACTATCCATAAAACCAACGCTCTTGTGTTTTTAGAAATGCTTAAAATTTTTGGA

CTTTTAAGCCAAGCACACCATAACGATGTGTTAAAGATTTTAGAAAAAATACTTCAAAAT

>HP98123

GTGAGTTTGATTAAGATTGATAATAATAAAAAAGCGATTGAGGTTTCTATTCCTTTAACT

------------TCCATTTCAGGCAAAGCGCGTGTGAAAATCAGACATGCCTTTAGCGAT

TATGGCATTTCAACAGCGACTAGAAAAATCCCTTTTAGTTTAAAGCATTATGTAGAGTGG

CAAATCGGTTATGATGTCCCCATTAAAGACAAAGAA---AAATTGGAGCTCACTACTTTA

AAAGATGAAAAATACCATTTTTTAGGGGCTAATAATAAAGTAAAAACTCTTTATGAATTG

AGTGAAATGATTGATTACGCTAAGCAATTAGGTTTAATCGGT---------TTAGAAAAT

TTAGAAAATACTTTAAAATATTTAGAAAAACAAAAACAATTTATAGAAGATAATTTTATG

ATTACAAGAGAAAGATTTAGAACGCATCAATTTGGTGGCATGGATTTTGAACTTTCACGC

ATTTCTTACCCTTTACTCATTCATTCTTTCAATGATAATCAATTGAGTGAAATTGTTATT

AGAGAGCAACAATACGGTTCTAAAACCCAAGCCATG---CTGTATTTTTGCTTTTCTATT

TTGGAATTAAAAACCGCTACTCCCTTATTAAATAGAACGGCTGCACTCAAAGAACATGCC

CTTTTAACTATCCATAAAACCAATGCTCTTGTGTTTTTAGAAATGCTTAAAATTTTTGGA

CTTTTAAGCCAAGTGCACCATAACGATGTGTTAAAGATTTTAGAAAAAATACTTCAAAAT

>HP99244

GTGAGTTTGATTAAAGTTGACTATGATAAAAAAGTGATTGAGGTTTCTATTCCTTTAACT

------------TCAATTTCAGGCAAAGTGCGTGTGAAAATCAGACATGCCTTTAGCGAT

TATGGTATTTCAACAGCGACTAGAAAAATTCCTTTTAGTTTAAAACATTATATAGAGTGG

CAGATCGGTTATGATGTCCCCATTAAAGATAAAGAA---AAATTTGAACTCACTGCTTTA

AAAGATAAAAAATATCATTTTTTAGGGGCTAATAATAAAGTAAAAACTCTTTATGAATTA

AGTGAAATGATTTATTACGCTAAGCAATTAGGTTTAATCAGT---------TTAGAAAAT

TTAGAAAATACTTTAAAATATTTAGAAAAACAAAAACAATTTATAGAAGATAATTTTATG

ATTACAAGAGAAAGATTTAGATCGCATCAATTTGGCGGCATGGATTTTGAACTTTCACGC

ATTTCTTATCCCTTACTCATTCATTCTTTTAACGATAATCAGTTGAGCGAAATAGTTATT

AGAGAACAACAATATGGCTCTAAAACCCAAGCCATG---CTGTATTTTTGCTTTTCTATT

TTGGAGTTAAAAACTGCTACCCCCTTATTAAATAGAACCGCTGCACTCAAAGAACATGCT

TTTTTAACCATCCATAAAACCAACGCTCTCATGTTTTTAGAAACGCTTAAAATTTTTGGG

CTTTTAAGCCAAGCGCACCATAACGATGCGTTAAAGATTTTAGAAAAAATACTTCAAAAT

>B29

GTGAGTTTGATTAGGATTGATGATAGTAAAAAAGCGATTGAGGTTTCTATTCCTTTAACT

------------TCAATTTCAGGCAAAGTGCGTGTGAAAATCAGACATGCCTTTAGCGAT

TATGGTATTTCAACAGCGACTAGAAAAATCCCTTTTAGTTTAAAGCATTATGTAGAGTGG

CAGATCGGTTATGATGTCCCCATTAAAGATAAAGAA---AAATTTGAGCTCACTACCCTA

AAAGATGAAAAATATCATTTTTTAGGGGCTAATAATAAAGTAAAAACCCTTTATGAATTG

AGTGAGATAATCTATTACGCTAAGCAATTAAATTTAATCAGT---------TTAGAAAAT

TTAGAAAATACTTTAAAATATTTAGAAAAACAAAAACAATTTATAGAAGATAATTTTATG

ATTACAAGAGAAAGATTTAGATTACATCAATTTGGTGGCATGGGTTTTGAACTCTCACGC

ATTTCTTATCCCTTACTCATTCATTCTTTCAATGATAATCAGTTGAGCGAAATCGTTATT

AGAGAACAACAATACGGCTCTAAAACCCAAGCCATG---CTGTATTTTTGCTTTTCTATT

TTGGAATTAAAAACCGCTACCCCCTTATTAAACAGAACGGCTGCACTCAAAGAACATGCC

CTTTTAACTATCCATAAAACCAACGCTCTTATGTTTTTAGAAATGCTTAAAATTTTTGGG

CTTTTAAGCCAAGTGCACCATAACGATGTGTTAAAGATTTTAGAAAAAATACTTCAAAAT

>18:2

GTGAGTTTGATTAAAGTTAGTGGTGATAAAAAAGCGATTGAGGTTTCTATTCCTTTAACT

------------TCAATTTCAGGCAAAGTGCGTGTGAAAATCAGACATGCCTTTAGCGAT

TATGGTATTTCAACAGCGACTAGAACAATCCCTTTTAGTTTAAAACATTATGTAGAGTGG

CAGATCGGTTATGATGTCCCTATTAAAGATAAAGAA---AAATTTGAACTCACTACTTTA

AAAGATGAAAAATATCATTTTTTAGGGGCTAATAATAAAGTAAAAACTCTTTATGAATTG

AGTGAAATGATTTATTACGCTAAGCAATTAGGTTTAATCAGT---------TTAGAAAAT

TTAGAAAATACTTTAAAATATTTAGAAAAACAAAAACAATTTATAGAAGATAATTTTATG

ATTACAAGAGAAAGATTTAGATCGCATCAATTTGGTGGCATGGATTTTGAACTCTCACGC

ATTTCTTATCCTTTACTCATTCATTCTTTTAATGATAATCAGTTGAGCGAAATAGTTATT

AGAGAACAACAATATGGCTCTAAAACCCAAGCCATG---CTGTATTTTTGCGTTTCTATT

TTGGAGTTAAAAACCGCTACTCCCTTATTAAATAGAACGGCTATGATCAAAGAACATGCC

CTTTTAACTATCCATAAAACCAACGCTCTCATGTTTTTAGAAATGCTTAAAATTTTTGGA

CTTTTAAGCCAAGCGCACCATAACGATGTGTTAAAGATTCTAGAAAAAATACTTCAAAAT

>GCT_43

GTGAGTTTGATTAGGATTGATGATAGTAAAAAAGCGATTGAGGTTTCTATTCCTTTAACT

------------TCAATTTCAGGCAAAGTGCGTGTGAAAATCAGACATGCCTTTAGCGAT

TATGGCATTTCAACAGCGACCAGAAAAATCCCTTTTAGTTTAAAGCATTATGTAGAGTGG

CAAATCGGTTATGATGTCCCCATTAAAGATAAAGAA---AAATTTAAACTCACTACTTTA

AAAGATGAAAAATACCATTTTTTAGGGGCCAATAATAAAGTAAAAACTCTTTATGAATTG

AGTGAAATAATTTATTACGCTAAGCAATTAAATTTAATCAGT---------TTAGAAAAT

TTAGAAAATACTTTAAAATATTTAGAAAAACAAAAACAATTTATAGAAGATAATTTCACG

ATTACAAGAGAAAGATTTAGATCGCATCAATTTGGGGGCATGGATTTTGAACTTTCACGC

ATCTCTTATCCTTTACTCATTCATTCTTTTAATGATAATCAGTTGAGCGAAATCGTTATT

AGAGAGCAACAATACGGCTCTAAAACCCAAGCCATG---CTGTATTTTTGCTTTTCTATT

CTGGAGTTAAAAACCGCTACACCCTTATTAAATAGGACCGCTGCCCTCAAAGAACATGCC

CTTTTAACTATCCATAAAACCAACGCTCTTGTGTTTTTAGAAATGCTTAAAATTTTTGGA

CTTTTAAGTCAAGCGCACCATAACGATGTGTTAAAGATTTTAGAAAAAATACTTGAAAAT

>ZH01

GTGAGTTTGATTAAAGTTAGTGGTGATAAAAAAGCGATTGAGGTTTCTGTTCCTTTAACT

------------TCAATTTCAGGCAAAGTGCGTGTGAAAATCAGACATGCCTTTAGCGAT

TATGGTATTTCAACAGCGACTAGAAAAATCCCTTTTAGTTTAAAACATTATGTAGAGTGG

CAAATCGGTTATGATGTCCCCATTAAAGATAAAGAA---AAATTTAAACTCACTACTTTA

AAAGATGAAAAATACCATTTTTTAGGGGCTAATGATAAAGTAAAGACTCTTTATGAATTG

AGTGAGATAATCTATTACGCTAAGCAATTAAATTTAATCAGT---------TTAGAAAAT

TTAGAAAATACTTTAAAATATTTAGAAAAACAAAAACAATTTATAGAAGATAATTTCACG

ATTACAAGAGAAAGATTTAGATCGCATCAATTTGGTGGCATGGATTTTGAACTTTCACGC

ATTTCTTATCCTTTACTCATTCATTCTTTTAATGATAATCAATTGAGTGAAATCGTTATT

AGAGAGCAACAATATGGCTCTAAAACCCAAGCCATG---CTGTATTTTTGCTTTTCTATT

TTGGAATTAAAAACCGCTACTCCCTTATTAAATAGAACGGCTGCACTCAAAGAACATGCC

CTTTTAACTATCCATAAAACCAACGCTCTTATGTTTTTAGAAATGCTTAAAATTTTTGGA

CTTTTAAGCCAAGCGCACCATAACGATGTGTTAAAGATTTTAGAAAAAATACTTCAAAAT

>HP11042

GTGAGTTTGATTAAGATTGATGATGATAAAAAAGTGATTGAGGTTTCTATTCCTTTAACT

------------TCAATTTCAGGCAAAGTGCGTGTGAAAATCAGACATGCCTTTAGCGAT

TATGGCATTTCAACAGCGACTAGAAAAATCCCTTTTAGTTTAAAACATTATGTAGAGTGG

CAAATCGGTTATGATGTCCCCATTAAAGATAAAGAA---AAATTGGAGCTCACTACTTTA

AAAGATGAAAAATATCATTTTTTAGGGGTTAATAATAAAGTAAAAACTCTTTATGAATTG

AGCGAAATAATCTATTACGCTAAGCAATTAAATTTAATCAGT---------TTAGAAAAT

TTAGAAAATACTTTAAAATATTTAGAAAAACAAAAACAATTTATAGAAGATAATTTTATG

ATTACAAGAGAAAGATTTAGATCGCATCAATTTGGTGGCATGGATTTTGAACTTTCACGC

ATTTCTTACCCTTTACTCATTCATTCTTTCAACGATAATCAGTTGAGCGAAATCGTTATT

AGAGAGCAACAATACGGCTCTAAAACCCAAGCCATG---CTGTATTTTTGCTTTTCTATT

TTGGAGTTAAAAACCGCTACCCCCTTATTAAATAGAACGGCCACACTCAAAGAACATGCT

CTTTTAACTATCCACAAAGCCAACGCTCTTATGTTTTTAGAAATGCTTAAAATTTTTGGG

CTTTTAAGCCAAGCGCACCATAACGATGTGTTAAAGATTTTAGAAAAAATACTTCAAAAT

>KH22

GTGAGTTTGATTAAAATTAACCATGATAAAAAAGTGATTGAGGTTTCTATTCCTTTAACT

------------TCAATTTCAGGCAAAGTGCGTGTGAAAATCAGACATGCCTTTAGCGAT

TATGGTGTTTCAACAGCGACTAGAAAAATCCCTTTTAGTTTAAAGCATTATGTAGAGTGG

CAAATCGGTTATGATGTCCCCATTAAAGATAAAGAA---AAATTTGAACTCACTGCTTTA

AAAGATGAAAAATATCATTTTTTAGGAGCTAATAATAAAGTGAAAACTCTTTATGAATTG

AGCGAAACGATTTATTACGCTAAGCAATTAGGTTTAATCAGT---------TTAGAAAAT

TTAGAAAATACTTTAAAATATTTAGAAAAACAAAAACAATTTATAGAAGATAATTTTATG

ATTGCAAGAGAAAGATTTAGATCGCATCAATTTGGTGGCATGGATTTTGAACTCTCACGC

ATTTCTTATCCTTTACTCATTCATTCTTTTAACGATAATCAGTTGAGTGAAATAGTTATT

AGAGAACAACAATACGGCTCTAAGACTCAAGCCATG---CTGTATTTTTGCTTTTCTATT

TTGGAGTTAAAAACCGCTACCCCCTTATTAAATAGAACCGCTACACTCAAAGAACATGCT

TTTTTAACCATCCATAAAACCAACGCTCTCATGTTTTTAGAAATGCTTAAAATTTTTGGA

CTTTTAAGCCAAGCGCACCATAACGATGTGTTAAAGATTTTAGAAAAAATACTTCAAAAT

>HP15060

GTGAGTTTGATTAAGATTGATAATAATAAAAAAGTAATTGGGGTTTCTATTCCTTTAACT

------------TCAATTTCAGGCAAAGTGCGTGTGAAAATCAGACATGCCTTTAGCGAT

TATGGTATTTCAACAGCGACTAGAAAAATCCCTTTTAGCTTAAAACATTATGTAGAGTGG

CAAATCGGTTATGATGTCCCCATTAAAGATAAAGAA---AAATTTGAGCTCACTACCCTA

AAAGATGAAAAATATCATTTTTTAGGGGCTAATAATAAAGTAAAAACCCTTTATGAATTG

AGTGAGATAATCTATTACGCTAAGCAATTAAATTTAATCAGT---------TTAGAAAAT

TTAGAAAATACTTTAAAATATTTAGAAAAACAAAAACAATTTATAGAAGATAATTTCACG

ATTACAAGAGAAAGATTTAGATTACATCAATTTGGTGGCATGGATTTTGAACTTTCACGC

ATTTCTTATCCTTTACTCATTCATTCTTTCAATGATAATCAATTGAGTGAAATCGTTATT

AGAGAGCAACAATATGGCTCTAAAACCCAAGCCATG---CTGTATTTTTGCTTTTCTATT

TTGGAATTAAAAACCGCTACCCCCTTATTAAATAGAACGGCTACACTCAAAGAACATGCC

CTTTTAACTATCCATAAAACCAACGCTCTTATGTTTTTAGAAATGCTTAAAATTTTTGGA

CTTTTAAGCCAAGCGCACCATAGCGATGTGTTAAAGATTTTAGAAAAAATACTTCAAAAT

>ZH31

GTGAGTTTGATTAGGATTGATAATAATAAAAAAGTAATTGGGGTTTCTATTCCTTTAACT

------------TCAATTTCAGGCAAAGTGCGTGTGAAAATCAGACATGCCTTTAGCGAT

TATGGTATTTCAACAGCGACTAGAAAAATCCCTTTTAGCTTAAAACATTATGTAGAGTGG

CAAATCGGTTATGATGTCCCCATTAAAGATAAAGAA---AAATTTGAGCTCACTACCCTA

AAAGATGAAAAATATCATTTTTTAGGGGCTAATAATAAAGTAAAAACCCTTTATGAATTG

AGTGAGATAATCTATTACGCTAAGCAATTAAATTTAATCAGT---------TTAGAAAAT

TTAGAAAATACTTTAAAATATTTAGAAAAACAAAAACAATTTATAGAAGATAATTTTATG

ATTACAAGAGAAAGATTTAGATTACATCAATTTGGTGGCATGGATTTTGAACTCTCACGC

ATTTCTTATCCTTTGCTCATTCATTCTTTTAATGATAATCAGTTGAGTGAAATCGTTATT

AGAGAACAACAATACGGCTCTAAAACCCAAGCCATG---CTGTATTTTTGCTTTTCTATT

TTGGAGTTAAAAACCGCTACCCCCTTATTAAACAGAACGGCTGCGCTCAAAGAACATGCT

CTTTTAATTATCCATGAAACTAACGCTCTTGTGTTTTTAGAAATGCTTAAAATTTTTGGA

CTTTTAAGCCAAGCACACCATAACGATGTGTTAAAGATTTTAGAAAAAATACTTGAAAAT

>E14

GTGAGTTTGATTAAAATTAACCATGATAAAAAAGTGATTGAGATTTCCATTCCTTTAACT

------------TCAATTTCAGGCAAAGTGCGTGTGAAAATCAGACATGCCTTTAGCGAT

TATGGTGTTTCAACAGCGACTAGAAAAATCCCTTTTAGTTTAAAGCATTATGTAGAGTGG

CAGATCGGTTATGATGTCCCCATTAAAGATAAAGAA---AAATTTGAACTCACTACTTTA

AAAGATGAAAAATATCATTTTTTAGGGGCTAATAATAAAACAAAAACTCTTTATGAATTA

AGCGAAATGATTTATTACGCTAAGCAATTAGGTTTAATCAGT---------TTAGAAAAT

TTAGAAAATACTTTAAAATATTTAGAGAAACAAAAACAATTTATAGAAGATAGTTTTATG

ATTACAAGAGAAAGATTTAAATCGCATCAATTTGGTGGCATGGATTTTGAACTTTCACGC

ATTTCTTATCCTTTGCTCATTCATTCTTTTAATGATAATCAATTGAGCGAAATTGTTATT

AGGGAACAACAATATGGTTCTAAAACCCAAGCCATG---CTGTATTTTTGCTTTTCTATT

TTGGAGTTAAAAACCGCTACCCCCTTATTAAATAGAACCGCTACGCTCAAAGAACATGCC

CTTTTAACTATCCATAAAACCAACGCTCTCATGTTTTTAGAAATGCTTAAAATTTTTGGA

CTTTTAAGCCAAGCGCACCATAGCGATGTGTTAAAGATTTTAGAAAAAATACTTCAAAAT

>HP15050

GTGAGTTTGATTAAAATTAACCATGATAAAAAAGTGATTGAGGTTTCTATTCCTTTAACT

------------TCAATTTCAGGCAAAGTGCGTGTGAAAATCAGACATGCCTTTAGCGAT

TATGGCATTTCAACAGCGACTAGAAAAATCCCTTTTAGTTTAAAACATTATGTAGAGTGG

CAAATCGGTTATGATGTCCCCATTAAAGATAAAGAA---AAATTTGAACTCACTACTTTA

AAAGATGAAAAATATCATTTTTTAGGGGCTAATAATAAAGTAAAGACTCTTTATGAATTG

AGCGAAATGATTTATTACGCTAAGCGATTGGGTTTAATCAGT---------TTAGAAAAT

TTAGAAAATACTTTAAAATATTTAGAAAAACAAAAACAATTTATAGAAGATAATTTTATG

ATTACAAGAGAAAGATTTAGATCGCATCAATTTGGTGGCATGGATTTTGAACTCTCACGT

ATTTCTTATCCCTTACTTATTCATTCTTTTAATGATAATCAGTTGAGCGAAATTGTTATT

AGAGAGCAACAATATGGCTCTAAAACCCAAGCCATG---CTGTATTTTTGCTTTTCTATT

TTGGAGTTAAAAACCGCTACCCCCTTATTAAACAGAACGGCTATGCTCAAAGAGCATGCT

CTTTTGATTATCCATAAAACCAACGCTCTCATGTTTTTAGAAATGCTTAAAATTTTTGGA

CTTTTAAGCCAAGTGCACCATAGCGATGTGTTAAAGATTTTAGAAAAAATACTTCAAAAT

>2010

GTGAGTTTGATTAGGATTGATGATAGTAAAAAAGCGATTGAGGTTTCTATTCCTTTAACT

------------TCCATTTCAGGCAAAGCGCGTGTGAAAATCAGACATGCCTTTAGCGAT

TATGGCATTTCAACAGCGACTAGAAAAATCCCTTTTAGTTTAAAACATTATGTAGAGTGG

CAAATCGGTTATGATGTCCCCATTAAAGATAAAGAA---AAATTTGAACTCACTACCCTA

AAAGATGAAAAATATCATTTTTTAGGGGCTAATAATAAAATAAAAACCCTTTATGAATTG

AGCGAAATAATTTATTACGCTAAGCGATTGGGTTTAATCAGT---------TTAGAAAAT

TTAGAAAATACTTTAAAATATTTAGAAAAACAAAAACAATTCATAGAAGATAGTTTTATG

ATCACAAGAGAAAGATTTAGATCGCATCAATTTGGGGGCATGGATTTTGAACTTTCACGC

ATTTCTTATCCCTTACTCATTCATTCTTTTAATGATAATCAATTGAGCGAAATCGTTATT

AGAGAGCAACAATACGGCTCTAAAACCCAAGCCATG---CTGTATTTTTGCTTTTCTATT

TTGGAGTTAAAAACCGCTACCCCCTTATTAAACAGAACGGCTATGCTCAAAGAGCATGCT

CTTTTGATTATCCATAAAACCAACGCTCTCATGTTTTTAGAAATGCTTAAAATTTTTGGA

CTTTTAAGCCAAGCGCACCATAACGATGTGTTAAAGATTTTAGAAAAAATACTTCAAAAT

>MHP35

GTGAGTTTGATTAAAGTTAATGATGATAAAAAAGTGATTGAGGTTTCTATTCCTTTAACT

------------TCCATTTCAGGCAAAGTTCGTGTGAAAATCAGGCATGCCTTTAGCGAT

TATGGCATTTCAACAGCGACTAGAAAAATCCCTTTTAGTTTAAAGCATTATGTAGAGTGG

CAAATCGGTTATGATGTCCCCATTAAAGATAAAGAA---AAATTTGAACTCACTACCCTA

AAAGATGAAAAATATCATTTTTTAGGGGCTAATAATAAAGTAAAAACCCTTTATGAATTG

AGCGAAATAATTGATTACGCTAAGCGATTGGGTTTAATCAGT---------TTAGAAAAT

TTAGAAAATACTTTAAAATATTTAGAAAAACAAAAACAATTTATAGAAGATAATTTTATG

ATTACAAGAGAAAGATTTAGATCGCATCAATTTGGTGGCATGGATTTTGAACTTTCACGC

ATTTCTTATCCTTTACTCATTCATTCTTTTAATGATAACCAATTGAGTGAAATCGTTATT

AGAGAGCAACAATATGGCTCTAAAACCCAAGCCATG---CTGTATTTTTGCTTTTCTATT

CTGGAATTAAAAACCGCTACACCCTTATTAAATAGAACGGCTGCACTCAAAGAACATGCC

CTTTTAACTATCCATAAAACCAACGCTCCCATGTTTTTAGAAATGCTTAAAATTTTTGGA

CTTTTAAGCCAAGCGCACCATAACGATGTGTTAAAGATTTTAGAAAAAATACTTCAAAAT

>207/99

GTGAGTTTGATTAGGATTGATGATAGTAAAAAAGCGATTGATGTTTCTATTCCTTTAACT

------------TCAATTTCAGGCAAAGTGCGTGTGAAAATCAGACATGCCTTTAGCGAT

TATGGTATTTCAACAGCGACTAGAAAAATCCCTTTTAGTTTAAAACATTATGTAGAGTGG

CAGATCGGTTATGATGTCCCTATTAAAGATAAAGAA---AAATTTGAACTCACTACTTTA

AAAGATGAAAAATATCATTTTTTAGGGGCTAATAATAAAGTAAAAACCCTTTATGAATTG

AGTGAGATAATCTATTACGCTAAGCAATTAAATTTAATCAGT---------TTAGAAAAT

TTAGAAAATACTTTAAAATATTTAGAAAAACAAAAACAATTTATAGAAGATAATTTCACG

ATTACAAGAGAAAGATTTAGATCGCATCAATTTGGTGGCATGGATTTTGAACTTTCACGC

ATTTCTTATCCTTTACTCATTCATTCTTTTAATGATAATCAATTGAGTGAAATCGTTATT

AGAGAGCAACAATATGGCTCTAAAACCCAAGCTATG---CTGTATTTTTGCTTTTCTATT

TTGGAGTTAAAAACCGCTACCCCTTTATTAAATAGAACGGCTGCACTCAAAGAACAGGCT

CTTTTAACCATCCATAAAACCAACGCTCTTATGTTTTTAGAAATGCTTAAAATTTTTGGA

CTTTTAAGCCAAGCGCACCATAGCGATGTGTTAAAGATTTTAGAAAAAATACTTCAAAAT

>ZH136

GTGAGTTTGATTAGGATTGATGATAGTAAAAAAGCGATTGAGGTTTCTATTCCTTTAACT

------------TCAATTTCAGGCAAAGTGCGTGTGAAAATCAGACATGCCTTTAGCGAT

TATGGCATTTCAACAGCGACTAGAAAAATCCCTTTTAGCTTAAAACATTATGTAGAGTGG

CAAATCGGTTATGATGTCCCCATTAAAGATAAAGAA---AAATTTAAACTCACTACTTTA

AAAGATGAAAAATATCATTTTTTAGGGGCTAATAATAAAGTAAAGACTCTTTATGAATTG

AGTGAGATAATCTATTACGCTAAGCAATTAAATTTAATCAGT---------TTAGAAAAT

TTAGAAAATACTTTAAAATATTTAGAAAAACAAAAACAATTTATAGAAGATAATTTCACG

ATTACAAGAGAAAGATTTAGATCGCATCAATTTGGTGGCATGGATTTTGAACTCTCACGC

ATTTCTTATCCTTTACTCATTCATTCTTTCAATGATAATCAGTTGAGTGAAATTGTTATT

AGGGAACAACAATACGGCTCTAAAACCCAAGCCATG---CTGTATTTTTGCTTTTCTATT

TTGGAATTAAAAACCGCTACCCCCTTATTAAATAGAACGGCTGCACTCAAAGAACATGCC

CTTTTAACTATCCATAAAACCAACGCTCTTATGTTTTTAGAAATGCTTAAAATTTTTGGA

CTTTTAAGCCAAGTGCACCATAGCGATGTGTTAAAGATTTTAGAAAAAATACTTCAAAAT

>2019-3

GTGAGTTTGATTAGGATTGATGATAGTAAAAAAGCGATTGAGGTTTCTATTCCTTTAACT

------------TCCATTTCAGGCAAAGCGCGTGTGAAAATCAGACATGCCTTTAGCGAT

TATGGTATTTCAACAGCGACTAGAAAAATCCCTTTTAGTTTAAAACATTATGTAGAGTGG

CAGATCGGTTATGATGTCCCCATTAAAGATAAAGAA---AAATTGGAGCTCACTACTTTA

AAAGATGAAAAATACCATTTTTTAGGAGCTAATAATAAAGTAAAAACTCTTTATGAATTA

AGCGAAATGATTTATTACGCTAAGCAATTAAATTTAATCAGT---------TTAGAAAAT

TTAGAAAATACTTTAAAATATTTAGAAAAACAAAAACAATTTATAGAAGATAGTTTTATG

ATTACAAGAGAAAGATTTAGATTACATCAATTTGGTGGCATGGATTTTGAACTTTCACGC

ATTTCTTATCCTTTACTCATTCATTCTTTTAATGATAATCAGTTGAGTGAAATCGTTATT

AGAGAGCAACAATACGGCTCTAAAACCCAAGCCATG---CTGTATTTTTGCTTTTCTATT

TTGGAGTTAAAAACCGCTACCCCTTTATTAAATAGAACGGCTGCACTCAAAGAACATGCC

CTTTTAACTATCCATAAAACCAACGCTCTTATGTTTTTAGAAATGCTTAAAATTTTTGGA

CTTTTAAGCCAAGCACACCATAGCGATGTGTTAAAGATTTTAGAAAAAATACTTCAAAAT

>HP01330

GTGAGTTTGATTAAAATTAACCATGATGAAAAAGTGATTGAGGTTTCTATTCCTTTAACT

------------TCAAATTCAGGCAAAGTGCGTGTGAAAATCAGACATGCCTTTAGCGAT

TATGGTATTTCAACAGCGACTAGAAAAATCCCTTTTAGTTTAAAGCATTATGTAGAGTGG

CAGATCGGTTATGATGTCCCCATTAAAGATAAAGAA---AAATTTGAACTCACTACTTTA

AAAGATGAAAAATATCATTTTTTAGGGGCTAATAATAAAGTAAAAACTCTTTATGAATTG

AGCGAAATGATTTATTACGCTAAGCAATTAGGTTTAATCAGT---------TTAGAAAAT

TTAGAAAATACTTTAAAATATTTAGAAAAACAAAAACAATTTATAGAAGATAATTTTATG

ATCACAAGAGAAAGATTTAGATCGCATCAATTTGGTGGCATGGATTTTGAACTCTCACGC

ATTTCTTATCCTTTACTCATTCATTCTTTTGATGATAATCAATTGAGCGAAATAGTTATT

AGAGAACAACAATATGGCTCTAAAACCCAAGCCATG---CTGTATTTTTGCTTTTCTATT

TTGGAATTAAAAACCGCTACTCCCTTATTAAACAGAACGGCTATGCTCAAAGAACATGCC

CTTTTGATTATCCATAAAACCAACGCTCCCATGTTTTTAGAAATGCTTAAAATTTTTGGA

CTTTTAAGCCAAGCACACCATAACGATGTGTTAAAGATTTTAGAAAAAATACTTCAAAAT

>KH0125

GTGAGTTTGATTAAAGTTAGTGGTGATAAAAAAGCGATTGAGGTTTCCATTCCTTTAACT

------------TCAATTTCAGGCAAAGTGCGTGTGAAAATCAGACATGCTTTTAGCGAT

TATGGTATTTCAACAGCGACTAGAAAAATCCCTTTTAGTTTAAAACATTATGTAGAGTGG

CAGATCGGTTATGATGTCCCCATTAAAGATAAAGAA---AAATTTGAACTCACTACTTTA

AAAGATGAAAAATATCATTTTTTAGGGGCTAATAATAAAGTAAAAACTCTTTATGAATTG

AGTGAAATAATCTATTACGCTAAGCAATTAAATTTAATCAGT---------TTAGAAAAT

TTAGAAAATACTTTAAAATATTTAGAAAAACAAAAACAATTTATAGAAGATAATTTCACG

ATTACAAGAGAAAGATTTAGATCGCATCAATTTGGGGGCATGGATTTTGAACTTTCACGC

ATTTCTTATCCCTTACTCATTCATTCTTTTAATGATAATCAATTGAGCGAAATCGTTATT

AGAGAGCAACAATACGGCTCTAAAACCCAAGCCATG---CTGTATTTTTGCTTTTCTATT

TTGGAATTAAAAACCGCTACTCCCTTATTAAATAGAACGGCTGCACTCAAAGAACATGCC

CTTTTAACTATCCATAAAACCAACGCTCTTGTGTTTTTAGAAATGCTTAAAATTTTTGGA

CTTTTAAGCCAAGCGCACCATAACGATGTGTTAAAGATTTTAGAAAAAATACTTCAAAAT

>KH0092

GTGAGTTTGATTAAAGTTAGTGGTGATAAAAAAGCGATTGAGGTTTCCATTCCTTTAACT

------------TCAATTTCAGGCAAAGTGCGTGTGAAAATCAGACATGCTTTTAGCGAT

TATGGTATTTCAACAGCGACTAGAAAAATCCCTTTTAGTTTAAAACATTATGTAGAGTGG

CAGATCGGTTATGATGTCCCCATTAAAGATAAAGAA---AAATTTGAACTCACTACTTTA

AAAGATGAAAAATATCATTTTTTAGGGGCTAATAATAAAGTAAAAACTCTTTATGAATTG

AGTGAAATAATCTATTACGCTAAGCAATTAAATTTAATCAGT---------TTAGAAAAT

TTAGAAAATACTTTAAAATATTTAGAAAAACAAAAACAATTTATAGAAGATAATTTCACG

ATTACAAGAGAAAGATTTAGATCGCATCAATTTGGGGGCATGGATTTTGAACTTTCACGC

ATTTCTTATCCCTTACTCATTCATTCTTTTAATGATAATCAATTGAGCGAAATCGTTATT

AGAGAGCAACAATACGGCTCTAAAACCCAAGCCATG---CTGTATTTTTGCTTTTCTATT

TTGGAATTAAAAACCGCTACTCCCTTATTAAATAGAACGGCTGCACTCAAAGAACATGCC

CTTTTAACTATCCATAAAACCAACGCTCTTGTGTTTTTAGAAATGCTTAAAATTTTTGGA

CTTTTAAGCCAAGCGCACCATAACGATGTGTTAAAGATTTTAGAAAAAATACTTCAAAAT

>HP14012

GTGAGTTTGATTAAAGTTAGTGGTGATAAAAAAGCGATTGAGGTTTCCATTCCTTTAACT

------------TCAATTTCAGGCAAAGTGCGTGTGAAAATCAGACATGCTTTTAGCGAT

TATGGTATTTCAACAGCGACTAGAAAAATCCCTTTTAGTTTAAAACATTATGTAGAGTGG

CAGATCGGTTATGATGTCCCCATTAAAGATAAAGAA---AAATTTGAACTCACTACTTTA

AAAGATGAAAAATATCATTTTTTAGGGGCTAATAATAAAGTAAAAACTCTTTATGAATTG

AGTGAAATAATCTATTACGCTAAGCAATTAAATTTAATCAGT---------TTAGAAAAT

TTAGAAAATACTTTAAAATATTTAGAAAAACAAAAACAATTTATAGAAGATAATTTCACG

ATTACAAGAGAAAGATTTAGATCGCATCAATTTGGGGGCATGGATTTTGAACTTTCACGC

ATTTCTTATCCCTTACTCATTCATTCTTTTAATGATAATCAATTGAGCGAAATCGTTATT

AGAGAGCAACAATACGGCTCTAAAACCCAAGCCATG---CTGTATTTTTGCTTTTCTATT

TTGGAATTAAAAACCGCTACTCCCTTATTAAATAGAACGGCTGCACTCAAAGAACATGCC

CTTTTAACTATCCATAAAACCAACGCTCTTGTGTTTTTAGAAATGCTTAAAATTTTTGGA

CTTTTAAGCCAAGCGCACCATAACGATGTGTTAAAGATTTTAGAAAAAATACTTCAAAAT

>HP99689

GTGAGTTTGATTAGGATTGATAATAGTAAAAAAGCGATTGAGGTTTCCATTCCTTTAACT

------------TCAATTTCAGGCAAAGTGCGTGTAAAAATCAGACATGCCTTTAGCGAT

TATGGCATTTCAACAGCGACCAGAAAAATCCCTTTTAGTTTAAAACATTATGTAGAGTGG

CAAATCGGTTATGATGTCCCCATTAAAGATAAAGAA---AAATTTGAACTCACTACTTTA

AAAGATGAAAAATATCATTTTTTAGGGGCTAATAATAAAGTAAAAACCCTTTATGAATTG

AGTGAGATAATCTATTACGCTAAGCAATTAAATTTAATCAGT---------TTAGAAAAT

TTAGAAAATACTTTAAAATATTTAGAAAAACAAAAACAATTCATAGAAGATAGTTTTATG

ATTACAAGAGAAAGATTTAGATTACATCAATTTGGTGGCATGGATTTTGAACTCTCACGC

ATTTCTTATCCTTTGCTCATTCATTCTTTTAATGATAATCAGTTGAGCGAAATCGTTATT

AGAGAACAACAATATGGCTCTAAAACCCAAGCCATG---CTGTATTTTTGCTTTTCTATT

TTGGAATTAAAAACCGCTACCCCCTTATTAAATAGAACGGCTGCACTCAAAGAACATGCT

TTTTTAACCATCAATAAAACCAACGCTCTTATGTTTTTAGAAATGCTTAAAATTTTTGGA

CTTTTAAGCCAAGTGCACCATAACGATGTGTTAAAGATTTTAGAAAAAATACTTCAAAAT

>43a2

GTGAGTTTGATTAAAATTAACCATGATAAAAAAATAATTGAGGTTTCTATTCCTTTAACT

------------TCAATTTCAGGCAAAGTGCGTGTGAAAATCAGACATGCCTTTAGCGAT

TATGGTATTTCAACAGCGACTAGAAAAATCCCTTTTAGTTTAAAACATTATGTAGAGTGG

CAGATCGGTTATGATGTCCCCATTAAAGATAAAGAA---AAATTTGAGCTCACTACCCTA

AAAGATGAAAAATATCATTTTTTAGGGGCTAATAATAAAGTAAAAACTCTTTATGAATTA

AGCGAAATGATTTATTACGCTAAGCAATTAGGTTTAATTAGT---------TTAGAAAAT

TTAGAAAATACTTTAAAATATTTAGAAAAACAAAAACAATTTATAGAAGATAATTTCACG

ATTACAAGAGAAAGATTTAGATCGCATCAATTTGGGGGCATGGATTTTGAACTTTCACGC

ATTTCTTATCCCTTACTCATTCATTCTTTTAATGATAATCAATTGAGCGAAATCGTTATT

AGAGAACAACAATACGGCTCTAAGACACAAGCCATG---CTGTATTTTTGCTTTTCTATT

TTGGAGTTAAAAACCGCTACTCCCTTATTAAATAGAACCGCTACGCTCAAAGAACATGCC

CTTTTGATTGTCCATCAAACTAACGCTCTTGTGTTTTTAGAAATGCTTAAAATTTTTGGG

CTTTTAAGCCAAGCGCACCATAACGATGTGTTAAAGATTTTAGAAAAAATACTTCAAAAT

>HP15054

GTGAGTTTGATTAAAATTAACCATGATAAAAAAATAATTGAGGTTTCTATTCCTTTAACT

------------TCAATTTCAGGCAAAGTGCGTGTGAAAATCAGACATGCCTTTAGCGAT

TATGGTATTTCAACAGCGACTAGAAAAATCCCTTTTAGTTTAAAACATTATGTAGAGTGG

CAGATCGGTTATGATGTCCCCATTAAAGATAAAGAA---AAATTTGAGCTCACTACCCTA

AAAGATGAAAAATATCATTTTTTAGGGGCTAATAATAAAGTAAAAACTCTTTATGAATTA

AGCGAAATGATTTATTACGCTAAGCAATTAGGTTTAATTAGT---------TTAGAAAAT

TTAGAAAATACTTTAAAATATTTAGAAAAACAAAAACAATTTATAGAAGATAATTTCACG

ATTACAAGAGAAAGATTTAGATCGCATCAATTTGGGGGCATGGATTTTGAACTTTCACGC

ATTTCTTATCCCTTACTCATTCATTCTTTTAATGATAATCAATTGAGCGAAATCGTTATT

AGAGAACAACAATACGGCTCTAAGACACAAGCCATG---CTGTATTTTTGCTTTTCTATT

TTGGAGTTAAAAACCGCTACTCCCTTATTAAATAGAACCGCTACGCTCAAAGAACATGCC

CTTTTGATTGTCCATCAAACTAACGCTCTTGTGTTTTTAGAAATGCTTAAAATTTTTGGG

CTTTTAAGCCAAGCGCACCATAACGATGTGTTAAAGATTTTAGAAAAAATACTTCAAAAT

>3125

GTGAGTTTGATTAAAGTTAATGATGATAAAAAAGCGATTGAGGTTTCTATTCCTTTAACT

------------TCCATTTCAGGCAAAGTGCGTGTGAAAATCAGGCATGCCTTTAGCGAT

TATGGTATTTCAACAGCGACCAGAAAAATCCCTTTTAGTTTAAAGCATTATGTAGAGTGG

CAAATCGGGTATGATGTCCCCATTAAAGATAAAGAA---AAATTTGAACTCACTACCCTA

AAAGATGAAAAATATCATTTTTTAGGGGCTAATAATAAAGTAAAAACTCTTTATGAATTG

AGTGAAATGATTTATTACGCTAAGCAATTAGGTTTAATCAGT---------TTAGAAAAT

TTAGAAAATACTTTAAAATATTTAGAAAAGCAAAAACAATTTATAGAAGATAGTTTTATG

ATTACAAGAGAAAGATTTAGATCGCATCAATTTGGTGGCATGGATTTTGAACTCTCACGC

ATTTCTTATCCTTTGCTCATTCATTCTTTTAATGATAATCAATTGAGTGAAATCGTTATT

AGAGAGCAACAATATGGCTCTAAAACCCAAGCCATG---CTGTATTTTTGCTTTTCTATT

TTGGAGTTAAAAACCGCTACCCCCTTATTAAATAGAACGGCTACGCTCAAAGAACATGCT

TTTTTAATTATCCATAAAACCAACGCTCTCATGTTTTTAGAAATGCTTAAAATTTTTGGA

CTTTTAAGCCAAGCGCACCATAGCGATGTGTTAAAGATTTTAGAAAAAATACTTCAAAAT

>UM131

GTGAGTTTGATTAAAGTTGACTATGATAAAAAAGTGATTGAGGTTTCTATTCCTTTAACT

------------TCAATTTCAGGCAAAGTGCGTGTGAAAATCAGACATGCCTTTAGCGAT

TATGGTATTTCAACAGCGACTAGAAAAATTCCTTTTAGTTTAAAACATTATATAGAGTGG

CAGATCGGTTATGATGTCCCCATTAAAGATAAAGAA---AAATTTGAACTCACTGCTTTA

AAAGATAAAAAATATCATTTTTTAGGGGCTAATAATAAAGTAAAAACTCTTTATGAATTA

AGTGAAATGATTTATTACGCTAAGCAATTAGGTTTAATCAGT---------TTAGAAAAT

TTAGAAAATACTTTAAAATATTTAGAAAAACAAAAACAATTTATAGAAGATAATTTTATG

ATTACAAGAGAAAGATTTAGATCGCATCAATTTGGCGGCATGGATTTTGAACTCTCACGC

ATTTCTTATCCTTTACTCATTCATTCTTTTAACGATAATCAATTGAGTGAAATAGTTATT

AGAGAACAACAATACGGCTCTAAGACCCAAGCCATG---CTGTATTTTTGCTTTTCTATT

TTGGAGTTAAAAACCGCTACCCCCTTATTAAATAGAACCGCTACACTCAAAGAACATGCC

CTTTTGATTATCCATCAAACTAACGCTCTCATGTTTTTAGAAATGTTTAAAATTTTTGGG

CTTTTAAGCCAAGCGCACCATAACGATGTGTTAAAGATTTTAGAAAAAATACTTCAAAAT

>MHP11

GTGAGTTTGATTAGGATTGATAATAATAAAAAAGTGATTGAGGTTTCCATTCCTTTAACT

------------TCAATTTCAGGCAAAGTGCGTGTGAAAATCAGACATGCCTTTAGCGAT

TATGGCATTTCAACAGCGACCAGAAAAATCCCTTTTAGTTTAAAACATTATGTAGAGTGG

CAAATCGGTTATGATGTCCTCATTAAAGATAAAGAA---AAATTTGAACTCACTACTTTA

AAAGATGAAAAATATCATTTTTTAGGGGCTAATAATAAAGTAAAAACCCTTTATGAATTG

AGTGAAATGATTTATTACGCTAAGCAATTAGGTTTAATCAGT---------TTAGAAAAT

TTAGAAAATACTTTAAAATATTTAGAAAAACAAAAACAATTTATAGAAGATAATTTTATG

ATCACAAGAGAAAGATTTAGATTGCATCAATTTGGTGGCATGGGTTTTGAACTTTCACGC

ATTTCTTATCCTTTACTCATTCATTCTTTTAATGATAATGAGTTGAGCGAAATAGTTATT

AGAGAACAACAATATGGCTCTAAAACCCAAGCCATG---CTGTATTTTTGCTTTTCTATT

TTGGAATTAAAAACCGCTACTCCCTTATTAAATAGAACGGCTATGCTCAAAGAGCATGCT

CTTTTGATTATCCATAAAACCAACGCTCCCATGTTTTTAGAAATGCTTAAAATTTTTGGA

CTTTTAAGCCAAGCGCACCATAACGATGTGTTAAAGATTTTAGAAAAAATACTTCAAAAT

>KH26

GTGAGTTTGATTAAAATTAACCATGATGAAAAAGTGATTGAGGTTTCCATTCCTTTAACT

------------TCAATTTCAGGCAAAGTGCGTGTGAAAATCAGACATGCCTTTAGCGAT

TATGGTGTTTCAACAGCGACTAGAAAAATCCCTTTTAGCTTAAAGCATTATGTAGAGTGG

CAAATCGGTTATGATGTCCCCATTAAAGATAAAGAA---AAATTTGAACTCACTACTTTA

AAAGATGAAAAATATCATTTTTTAGGGGCTAATAATAAAGTAAAAACTCTTTATGAATTG

AGCGAAATGATTTATTACGCTAAGCAATTAGGTTTAATCAGT---------TTAGAAAAT

TTAGAAAATACTTTAAAATATTTAGAAAAACAAAAACAATTTATAGAAGATAATTTTATG

ATTACAAGAGAAAGATTTAGATCGCATCAATTTGGTGGCATGGATTTTGAACTTTCACGC

ATTTCTTACCCTTTACTCATTCATTCTTTCAACGATAATCAGTTGAGCGAAATCGTTATT

AGAGAGCAACAATATGGCTCTAAAACTCAAGCCATG---CTGTATTTTTGCTTTTCTATT

TTGGAATTAAAAACCGCTACTCCCTTATTAAATAGAACGGCTATGCTCAAAGAACATGCT

TTTTTAACCATCAATAAAACCAACGCTCTCATGTTTTTAGAAATGCTTAAAATTTTTGGA

CTTTTAAGCCAAGCGCACCATAAAGATGTGTTAAAGATTTTAGAAAAAATACTTCAAAAT

>MHP34

GTGAGTTTGATTAAAGTTAATGATGATAAAAAAGTGATTGAGGTTTCTATTCCTTTAACT

------------TCCATTTCAGGCAAAGTTCGTGTGAAAATCAGACATGCCTTTAGCGAT

TATGGTATTTCAACAGCGACTAGAAAAATCCCTTTCAGTTTAAAGCATTATGTAGAGTGG

CAAATCGGTTATGATGTCCCCATTAAAGATAAAGAA---AAATTTGAACTCACTACCCTA

AAAGATGAAAAATATCATTTTTTAGGGGCTAATAATAAAGTAAAGACTCTTTATGAATTG

AGCGAAATGATTGATTACGCTAAGCGATTGGGTTTAATCAGT---------TTAGAAAAT

TTAGAAAATACTTTAAAATATTTAGAAAAACAAAAACAATTCATAGAAGATAATTTTATG

ATTACAAGAGAAAGATTTAGATCGCATCAATTTGGGGGCATGGATTTTGAACTTTCACGC

ATTTCTTATCCTTTACTCATTTATTCTTTTAATGATAATCAGTTGAGCGAAATCGTTATT

AGAGAGCAACAATACGGCTCTAAAACCCAAGCCATG---CTGTATTTTTGCTTTTCTATT

CTGGAATTAAAAACCGCTACCCCTTTATTAAATAGAACGGCTGCTCTCAAAGAACATGCT

CTTTTAACTATCCATAAAACCAACGCTCTTGTGTTTTTAGAAATGCTTAAAATTTTTGGC

CTTTTAAGCCAAGCGCACCATAACGATGTGTTAAAGATTTTAGAAAAAATACTTGAAAAT

>KH6

GTGAGTTTGATTAAAGTTAGTGGTGATAAAAAAGTGATTGAGGTTTCTATTCCTTTAACT

------------TCAATTTCAGGCAAAGTGCGTGTGAAAATCAGGCATGCCTTTAGCGAT

TATGGTCTTTCAACAGCGACTAGAAAAATCCCTTTTAGTTTAAAACATTATGTAGAGTGG

CAGATCGGTTATGATGTCCCCATTAAAGATAAAGAA---AAATTTGAACTCACTACTTTA

AAAGATGAAAAATATCATTTTTTAGGGGCTAATAATAAAGTAAAAACTCTTTATGAATTA

AGTGAAATGATTTATTACGCTAAGCAATTAGGTTTAATCAGT---------TTAGAAAAT

TTAGAAAATACTTTAAAATATTTAGAAAAACAAAAACAATTTATAGAAGATAATTTTATG

ATTACAAGAGAAAGATTTAGATCGCATCAATTTGGTGACATGGATTTTGAACTTTCACGC

ATTTCTTATCCTTTACTCATTCATTCTTTTAATGATAATCAATTGAGTGAAATCGTTATT

AGAGAACAACAATATGGCTCTAAAACCCAAGCCATG---CTGTATTTTTGCTTTTCTATT

TTGGAGTTAAAAACCGCTACCCCCTTATTAAATAGAACCGCTACACTCAAAGAACATGCT

CTTTTGATTATCCATAAAACCAACGCTCTCATGTTTTTAGAAATGCTTAAAATTTTTGGA

CTTTTAAGCCAAGCGCACCATAGCGATGTGTTAAAGATTTTAGAAAAAATACTTCAAAAT

>Nic39-A

GTGAGTTTGATTAAAGTTAATGATGATAAAAAAGTGATTGAGGTTTCTATTCCTTTAACT

------------TCCATTTCAGGCAAAGTTCGTGTGAAAATCAGACATGCCTTTAGCGAT

TATGGCATTTCAACAGCGACTAGAAAAATCCCTTTCAGTTTAAAGCATTATGTAGAGTGG

CAAATCGGTTATGATGTCCCCATTAAAGATAAAGAA---AAATTTGAACTCACTACCCTA

AAAGATGAAAAATATCATTTTTTAGGGGCTAATAATAAAATAAAAACCCTTTATGAATTG

AGCGAAATGATTTATTACGCTAAGCGATTGGGTTTAATCAGT---------TTAGAAAAT

TTAGAAAATACTTTAAAATATTTAGAAAAACAAAAACAATTCATAGAAGATAGTTTTATG

ATCACAAGAGAAAGATTTAGATCGCATCAATTTGGTGGCATGGATTTTGAACTTTCACGC

ATCTCTTACCCTTTACTCATTCATTCTTTTAATGATAATCAGTTGAGCGAAATCGTTATT

AGAGAGCAACAATACGGCTCTAAAACCCAAGCCATG---CTATATTTTTGCTTTTCTATT

CTGGAATTAAAAACCACTACCCCCTTATTAAATAGAACGGCTGCACTCAAAGAACATGCC

CTTTTAACTATCCATAAAACCAACGCTCTTGTGTTTTTAGAAATGCTTAAAATTTTTGGA

CTTTTAAGCAAAGCGCACCATAACGATGTGTTAAAGATTTTAGAAAAAATACTTGAAAAT

>HP15058

GTGAGTTTGATTAAAGTTAGTGGTGATAAAAAAGTGATTGAGGTTTCTATTCCTTTAACT

------------TCAATTTCAGGCAAAGCGCGTGTGAAAATCAGACATGCCTTTAGCGAT

TATGGTATTTCAACAGCGACTAGAAAAATCCCTTTTAGTTTAAAGCATTATGTAGAGTGG

CAGATCGGTTATGATGTCCCCATTAAAGATAAAGAA---AAATTTGAACTCACTACTTTA

AAAGATGAAAAATATCATTTTTTAGGGGCTAATAATAAAGTGAAAACTCTTTATGAATTA

AGCGAAATGATTTATTACGCTAAGCAATTAGGTTTAATCAGT---------TTAGAAAAT

TTAGAAAATACTTTAAAATATTTAGAAAAACAAAAACAATTTATAGAAGATAATTTCACG

ATTACAAGAGAAAGATTTAGATCGCATCAATTTGGTGACATGGATTTTGAACTTTCACGC

ATTTCTTATCCTTTACTCATTCATTCTTTTAATGATAATCAATTGAGTGAAATCGTTATT

AGAGAGCAACAATATGGCTCTAAAACCCAAGCCATG---CTGTATTTTTGCTTTTCTATT

TTGGAATTAAAAACCGCTACTCCCTTATTAAATAGAACGGCTGCACTCAAAGAACATGCC

CTTTTAACTATCCATAAAACCAACGCTCTTATGTTTTTAGAAATGCTTAAAATTTTTGGA

CTTTTAAGTCAAGTGCACCATAACGATGTGTTAAAGATTTTAGAAAAAATATTTCAAAAT

>MGms167

GTGAGTTTGATTAAAGTTAATGATGATAAAAAAGCGATTGAGGTTTCTATTCCTTTAACT

------------TCCATTTCAGGCAAAGTGCGTGTGAAAATCAGACATGCCTTTAGCGAT

TATGGCATTTCAACAGCGACCAGAAAAATCCCTTTTAGTTTAAAGCATTATGTAGAGTGG

CAAATCGGTTATGATGTCCCTATTAAAGATAAAGAA---AAATTTAAACTCACTACTTTA

AAAGATGAAAAATACCATTTTTTAGGGGCCAATAATAAAGTAAAAACTCTTTATGAATTG

AGTGAAATAATTTATTACGCTAAGCAATTAAATTTAATCAGT---------TTAGAAAAT

TTAGAAAATACTTTAAAATATTTAGAAAAACAAAAACAATTCATAGAAGATAGTTTTATG

ATCACAAGAGAAAGATTTAGATCGCATCAATTTGGGGGCATGGATTTTGAACTTTCACGC

ATCTCTTATCCCTTACTCATTCATTCTTTCAATGATAATCAGTTGAGCGAAATCGTTATT

AGAGAGCAACAATACGGCTCTAAAACCCAAGCCATG---CTGTATTTTTGCTTTTCTATT

TTGGAATTAAAAACCGCTACTCCCTTATTAAACAGAACGGCTGCACTCAAAGAACATGCC

CTTTTAACTATCCATAAAACCAACGCTCTTGTGTTTTTAGAAATGCTTAAAATTTTTGGA

CTTTTAAGCCAAGCACACCATAACGATGTGTTAAAGATTTTAGAAAAAATACTTCAAAAT

>G-Mx-2005-104

GTGAGTTTGATTAAAGTTAATGATGATAAAAAAGCGATTGAGGTTTCTATTCCTTTAACT

------------TCCATTTCAGGCAAAGTGCGTGTGAAAATCAGACATGCCTTTAGCGAT

TATGGCATTTCAACAGCGACCAGAAAAATCCCTTTTAGTTTAAAGCATTATGTAGAGTGG

CAAATCGGTTATGATGTCCCTATTAAAGATAAAGAA---AAATTTAAACTCACTACTTTA

AAAGATGAAAAATACCATTTTTTAGGGGCCAATAATAAAGTAAAAACTCTTTATGAATTG

AGTGAAATAATTTATTACGCTAAGCAATTAAATTTAATCAGT---------TTAGAAAAT

TTAGAAAATACTTTAAAATATTTAGAAAAACAAAAACAATTCATAGAAGATAGTTTTATG

ATCACAAGAGAAAGATTTAGATCGCATCAATTTGGGGGCATGGATTTTGAACTTTCACGC

ATCTCTTATCCCTTACTCATTCATTCTTTCAATGATAATCAGTTGAGCGAAATCGTTATT

AGAGAGCAACAATACGGCTCTAAAACCCAAGCCATG---CTGTATTTTTGCTTTTCTATT

TTGGAATTAAAAACCGCTACTCCCTTATTAAACAGAACGGCTGCACTCAAAGAACATGCC

CTTTTAACTATCCATAAAACCAACGCTCTTGTGTTTTTAGAAATGCTTAAAATTTTTGGA

CTTTTAAGCCAAGCACACCATAACGATGTGTTAAAGATTTTAGAAAAAATACTTCAAAAT

>26083

GTGAGTTTGATTAAGATTGATAATGATAAAAAAGCGATTGAGGTTTCTATTCCTTTAACT

------------TCCATTTCAGGCAAAGCACGTGTGAAAATCAGACATGCCTTTAGCGAT

TATGGCATTTCAACAGCGACTAGAAAAATCCCTTTTAGTTTAAAACATTATGTAGAGTGG

CAAATCGGTTATGATGTCCCCATTAAAGATAAAGAA---AAATTTGAACTCACTACCCTA

AAAGATGAAAAATATCATTTTTTAGGGGCTAATAATAAAATAAAAACCCTTTATGAATTG

AGCGAAATAATTTATTACGCTAAGCGATTGGGTTTAATCAGT---------TTAGAAAAT

TTAGAAAATACTTTAAAATATTTAGAAAAACAAAAACAATTCATAGAAGATAGTTTTATG

ATCACAAGAGAAAGATTTAGATCGCATCAATTTGGGGGCATGGATTTTGAACTTTCACGC

ATTTCTTATCCCTTACTCATTCATTCTTTTAATGATAATCAATTGAGCGAAATCGTTATT

AGAGAGCAACAATACGGCTCTAAAACCCAAGCCATG---CTGTATTTTTGCTTTTCTATT

TTGGAGTTAAAAACCGCTACCCCCTTATTAAACAGAACGGCTATGCTCAAAGAGCATGCT

CTTTTGATTATCCATAAAACCAACGCTCTCATGTTTTTAGAAATGCTTAAAATTTTTGGA

CTTTTAAGCCAAGTGCACCATAACGATGTGTTAAAGATTTTAGAAAAAATACTTCAAAAT

>NCTC13345

GTGAGTTTGATTAAAGTTAATGATGATAAAAAAGCGATTGAGGTTTCTATTCCTTTAACT

------------TCCATTTCAGGCAAAGTGCGTGTGAAAATCAGACATGCCTTTAGCGAT

TATGGCATTTCAACAGCGACCAGAAAAATCCCTTTTAGTTTAAAGCATTATGTAGAGTGG

CAAATCGGTTATGATGTCCCCATTAAAGATAAAGAA---AAATTGGAACTCACTACCCTA

AAAGATGAAAAATATCATTTTTTAGGGGCTAACAATAAAGTAAAGACCCTTTATGAATTG

AGTGAAATAATTTATTACGCTAAGCAATTAAATTTAATCGGT---------TTAGAAAAT

TTAGAAAATACTTTAAAATATTTAGAAAAACAAAAACAATTTATAGAAGATAATTTTATG

ATTACAAGAGAAAGATTTAGATCCCATCAATTTGGTGGCATGGATTTTGAACTCTCACGC

ATCTCTTACCCTTTACTCATTCATTCTTTCAACGATAATCAGTTGAGCGAAATCGTTATT

AGAGAGCAACAATACGGCTCTAAAACCCAAGCCATG---CTGTATTTTTGCTTTTCTATT

TTGGAGTTAAAAACCGCTACCCCCTTATTAAATAGGACGGCTGCACTCAAAGAACACGCT

CTTTTAACTATCCATAAAACCAACGCTCTTGTGTTTTTAGAAATGCTTAAAATTTTTGGA

CTTTTAAGCCAAGCGCACCATAACGATGTGTTAAAGATTTTAGAAAAAATACTTCAAAAT

>59

GTGAGTTTGATTAAGATTGATAATAATAAAAAAGTAATTGGGGTTTCTATTCCTTTAACT

------------TCAATTTCAGGCAAAGTGCGTGTGAAAATCAGACATGCCTTTAGCGAT

TATGGCATTTCAACAGCGACTAGAAAAATCCCTTTTAGTTTAAAGCATTATGTAGAGTGG

CAAATCGGTTATGATGTCCCCATTAAAGATAAAGAA---AAATTTAAACTCACTACTTTA

AAAGATGAAAAATATCATTTTTTAGGGGCTAATAATAAAGTAAAGACTCTTTATGAATTG

AGTGAGATAATCTATTACGCTAAGCAATTAAATTTAATCAGT---------TTAGAAAAT

TTAGAAAATACTTTAAAATATTTAGAAAAACAAAAACAATTTATAGAAGATAGTTTCACG

ATTACAAGAGAAAGATTTAGATCGCATCAATTTGGTGGCATGGATTTTGAACTTTCACGC

ATTTCTTATCCTTTACTCATTCATTCTTTTAATGATAATCAGTTGAGTGAAATCGTTATT

AGAGAGCAACAATACGGCTCTAAAACCCAAGCCATG---CTGTATTTTTGCTTTTCTATT

TTGGAATTAAAAACCGCTACTCCCTTATTAAATAGAACGGCTGCACTCAAAGAACATGCT

TTTTTAACCATCAATAAAACCAACGCTCTTATGTTTTTAGAAATGCTTAAAATTTTTGGA

CTTTTAAGCCAAGCGCACCATAACGATGTGTTAAAGATTTTAGAAAAAGTACTTCAAAAT

>HP08073

GTGAGTTTGATTAAAGTTAGTGGTGATAAAAAAGTGATTGAGGTTTCTATTCCTTTAACT

------------TCCATTTCAGGCAAAGTGCGTGTGAAAATCAGACATGCCTTTAGCGAT

TATGGTATTTCAACAGCGACTAGAAAAATCCCTTTTAGCTTAAAACATTATGTAGAGTGG

CAAATCGGTTATGATGTCCCCATTAAAGATAAAGAA---AAATTTGAGCTCACTACCCTA

AAAGATGAAAAATATCATTTTTTAGGGGCTAATAATAAAGTAAAAACCCTTTATGAATTG

AGTGAGATAATCTATTACGCTAAGCAATTAAATTTAATCAGT---------TTAGAAAAT

TTAGAAAATACTTTAAAATATTTAGAAAAACAAAAACAATTCATAGAAGATAGTTTTATG

ATTACAAGAGAAAGATTTAGATTACATCAATTTGGTGGCATGGATTTTGAACTCTCACGC

ATTTCTTATCCTTTGCTCATTCATTCTTTTAATGATAATCAGTTGAGCGAAATCGTTATT

AGAGAACAACAATACGGCTCTAAAACCCAAGCCATG---CTGTATTTTTGCTTTTCTATT

TTGGAGTTAAAAACCGCTACCCCCTTATTAAATAGAACGGCTGCACTCAAAGAACATGCC

CTTTTAACTATCCATAAAACCAACGCTCTCATGTTTTTAGAAATGCTTAAAATTTTTGGA

CTTTTAAGCCAAGCGCACCATAGCGATGTGTTAAAGATTTTAGAAAAAATACTTCAAAAT

>B25

GTGAGTTTGATTAGGATTGATGATGATAAAAAAGCGATTGAGGTTTTTATTCCTTTAACT

------------TCAATTTCAGGCAAAGCGCGTGTGAAAATCAGACATGCCTTTAGCGAT

TATGGTATTTCAACAGCGACTAGAAAAATCCCTTTTAGTTTAAAACATTATGTAGAGTGG

CAAATCGGTTATGATGTCCCCATTAAAGATAAAGAA---AAATTTGAACTCACTACTTTA

AAAGATGAAAAATATCATTTTTTAGGGGCTAATAATAAAGTAAAAACCCTTTATGAATTA

AGCGAAATGATTGATTACGCTAAGCAATTAGGTTTAATCAGT---------TTAGAAAAT

TTAGAAAATACTTTAAAATATTTAGAAAAACAAAAACAATTTATAGAAGATAATTTCACG

ATTACAAGAGAAAGATTTAGATTACATCAATTTGGTGGCATGGATTTTGAACTTTCACGC

ATTTCTTATCCTTTACTCATTCATTCTTTCAATGATAATCAATTGAGTGAAATCGTTATT

AGAGAGCAACAATATGGCTCTAAAACCCAAGCCATG---CTGTATTTTTGCTTTTCTATT

TTGGAGTTAAAAACCGCTACTCCCTTATTAAATAGAACGGCTGCACTCAAAGAACATGCC

CTTTTAACTATCCATAAAACCAACGCTCTTATGTTTTTAGAAATGCTTAAAATTTTTGGA

CTTTTAAGCCAAGCGCACCATAACGATGTGTTAAAGATTTTAGAAAAAATACTTCAAAAT

>MHP18

GTGAGTTTGATTAGGATTGATAATAATAAAAAAGCGATTGAGGTTTCTATTCCTTTAACT

------------TCAATTTCAGGCAAAGCGCGTGTGAAAATCAGACATGCCTTTAGCGAT

TATGGCATTTCAACAGCGACCAGAAAAATCCCTTTTAGTTTAAAGCATTATGTAGAGTGG

CAAATCGGTTATGATGTCCCTATTAAAGATAAAGAA---AAATTTAAACTCACTACTTTA

AAAGATGAAAAATACCATTTTTTAGGGGCCAATAATAAAGTAAAAACTCTTTATGAATTG

AGTGAAATAATTTATTACGCTAAGCAATTAAATTTAATCAGT---------TTAGAAAAT

TTAGAAAATACTTTAAAATATTTAGAAAAACAAAAACAATTCATAGAAGATAGTTTTATG

ATCACAAGAGAAAGATTTAGATTACATCAATTTGGGGGCATGGATTTTGAACTTTCACGC

ATTTCTTATCCTTTACTCATTCATTTTTTCAATGATAATCAGTTGAGCGAAATCGTTATT

AGAGAGCAACAATACGGCTCTAAAACCCAAGCCATG---CTGTATTTTTGCTTTTCTATT

TTGGAATTAAAAACCGCTACCCCCTTATTAAATAGAACGGCTGCACTCAAAGAACATGCC

CTTTTAACTATCCATAAAACCAACGCTCTTATGTTTTTAGAAATGCTTAAAATTTTTGGA

CTTTTAAGCCAAGCGCACTATAACGATGTGTTAAAGATTTTAGAAAAAATACTTCAAAAT

>22317

GTGAGTTTGATTAGGATTGATAATAATAAAAAAGTAATTGGGGTTTCTATTCCTTTAACT

------------TCAATTTCAGGCAAAGCGCGTGTGAAAATCAGACATGCCTTTAGCGAT

TATGGCATTTCAACAGCGACCAGAAAAATCCCTTTTAGTTTAAAGCATTATGTAGAGTGG

CAAATCGGTTATGATGTCCCTATTAAAGATAAAGAA---AAATTTAAACTCACTACTTTA

AAAGATGAAAAATACCATTTTTTAGGGGCCAATAATAAAGTAAAAACTCTTTATGAATTG

AGTGAAATAATTTATTACGCTAAGCAATTAAATTTAATCAGT---------TTAGAAAAT

TTAGAAAATACTTTAAAATATTTAGAAAAACAAAAACAATTCATAGAAGATAGTTTTATG

ATCACAAGAGAAAGATTTAGATCGCATCAATTTGGGGGCATGGATTTTGAACTTTCACGC

ATCTCTTATCCCTTACTCATTCATTCTTTTAATGATAATCAGTTGAGCGAAATTGTTATT

AGGGAACAACAATATGGCTCTAAAACCCAAGCCATG---CTGTATTTTTGCTTTTCTATT

TTGGAATTAAAAACCGCTACTCCCTTATTAAATAGAACGGCTGCACTCAAAGAACATGCT

TTTTTAATTATCCATAAAACTAACGCTCTTGTGTTTTTAGAAATGCTTAAAATTTTTGGG

CTTTTAAGCCAAGTGCACCATAACGATGTGTTAAAGATTTTAGAAAAAATACTTCAAAAT

>SV449_1

GTGAGTTTGATTAAGATTGATAATAATAAAAAAGTAATTGAGGTTTCTATTCCTTTAACT

------------TCCATTTCAGGCAAAGTGCGTGTGAAAATCAGACATGCCTTTAGCGAT

TATGGCATTTCAACAGCGACTAGAAAAATCCCTTTTAGTTTAAAGCATTATGTAGAGTGG

CAAATCGGTTATGATGTCCTTATTAAAGATAAAGAA---AAATTTGAGCTCACTACTTTA

AAAGATGAAAAATATCATTTTTTAGGGGCTAATAATAAAGTAAAAACCCTTTATGAATTG

AGTGAAATAATTTATTACGCTAAGCGATTGGGTTTAATCGGT---------TTAGAAAAT

TTAGAAAATACTTTAAAATATTTAGAAAAACAAAAACAATTCATAGAAGATAATTTCACG

ATTACAAGAGAAAGATTTAGATCGCATCAATTTGGTGGCATGGATTTTGAACTTTCACGC

ATTTCTTATCCTTTACTCATTCATTCTTTTAATGATAATCAGTTGAGTGAAATTGTTATT

AGGGAACAACAATACGGCTCTAAAACCCAAGCCATG---CTGTATTTTTGCTTTTCTATT

TTGGAGTTAAAAACCGCTACCCCCTTATTAAACAGAACGGCTGCACTCAAAGAGCATGCT

CTTTTGATTATCCATAAAACCAACACTCTTGTGTTTTTAGAAATGCTTAAAATTTTTGGA

CTTTTAAGCCAAGCACACCATAACGATGTGTTAAAGATTTTAGAAAAAATACTTCAAAAT

>40A6

GTGAGTTTGATTAAAGTTGACTATGATAAAAAAGTGATTGAGGTTTCTATTCCTTTAACT

------------TCAATTTCAGGCAAAGTGCGTGTGAAAATCAGACATGCCTTTAGCGAT

TATGGCATTTCAACAGCGACTAGAAAAATCCCTTTTAGTTTAAAACATTATGTAGAGTGG

CAAATCGGTTATGATGTCCCCATTAAAGATAAAGAA---AAATTGGAGCTCACTACTTTA

AAAGATGAAAAATATCATTTTTTAGGGGCTAATAATAAAGTAAAGACTCTTTATGAATTG

AGCGAAATAATTTATTACGCTAAGCAATTAAATTTAATCAGT---------TTAGAAAAT

TTAGAAAATACTTTAAAATATTTAGAAAAACAAAAACAATTCATAGAAGATAATTTCACG

ATTACAAGAGAAAAATTTAGATCGCATCAATTTGGTGGCATGGATTTTGAACTTTCACGC

ATCTCTTACCCTTTACTCATTCATTCTTTCAATGATAATCAATTGAGTGAAATCGTTATT

AGAGAGCAACAATACGGCTCTAAAACCCAAGCCATG---CTGTATTTTTGCTTTTCTATT

TTGGAATTAAAAACCGCTACCCCCTTACTAAATAGAACGGCTGCACTCAAAGAACATGCC

CTTTTAACTATCCATAAAACCAACGCTCTTGTGTTTTTAGAAATGCTTAAAATTTTTGGA

CTTTTAAGCCAAGCACACCATAACGATGTGTTAAAGATTTTAGAAAAAATACTTCAAAAT

>KH36

GTGAGTTTGATTAAAGTTGACTATGATAAAAAAGTGATTGAGGTTTCTATTCCTTTAACT

------------TCAATTTCAGGCAAAGTGCGTGTGAAAATCAGACATGCCTTTAGCGAT

TATGGCATTTCAACAGCGACTAGAAAAATCCCTTTTAGTTTAAAACATTATGTAGAGTGG

CAAATCGGTTATGATGTCCCCATTAAAGATAAAGAA---AAATTGGAGCTCACTACTTTA

AAAGATGAAAAATATCATTTTTTAGGGGCTAATAATAAAGTAAAGACTCTTTATGAATTG

AGCGAAATAATTTATTACGCTAAGCAATTAAATTTAATCAGT---------TTAGAAAAT

TTAGAAAATACTTTAAAATATTTAGAAAAACAAAAACAATTCATAGAAGATAATTTCACG

ATTACAAGAGAAAAATTTAGATCGCATCAATTTGGTGGCATGGATTTTGAACTTTCACGC

ATCTCTTACCCTTTACTCATTCATTCTTTCAATGATAATCAATTGAGTGAAATCGTTATT

AGAGAGCAACAATACGGCTCTAAAACCCAAGCCATG---CTGTATTTTTGCTTTTCTATT

TTGGAATTAAAAACCGCTACCCCCTTACTAAATAGAACGGCTGCACTCAAAGAACATGCC

CTTTTAACTATCCATAAAACCAACGCTCTTGTGTTTTTAGAAATGCTTAAAATTTTTGGA

CTTTTAAGCCAAGCACACCATAACGATGTGTTAAAGATTTTAGAAAAAATACTTCAAAAT

>HP08074

GTGAGTTTGATTAAGATTGATAATAATAAAAAAGTAATTGAGGTTTCTATTCCTTTAACT

------------TCCATTTCAGGCAAAGTGCGTGTGAAAATCAGACATGCTTTTAGCGAT

TATGGCATTTCAACAGCGACTAGAAAAATCCCTTTTAGTTTAAAGCATTATGTAGAGTGG

CAAATCGGTTATGATGTCCCCATTAAAGACAAAGAA---AAATTTGAGCTCACTACCCTA

AAAGATGAAAAATATCATTTTTTAGGGGCTAATAATAAAGTAAAAACTCTTTATGAGTTG

AGTGAGATAATTTATTACGCTAAGCAATTAAATTTAATCAGT---------TTAGAAAAT

TTAGAAAATACTTTAAAATATTTAGAAAAACAAAAACAATTCATAGAAGATAGTTTCACG

ATTACAAGAGAAAGATTTAGATCGCATCAATTTGGTGGCATGGATTTTGAACTCTCACAC

ATTTCTTATCCCTTACTCATTCATTCTTTCAACGATAATCAGTTGAGTGAAATCGTTATT

AGAGAGCAACAATACGGCTCTAAAACCCAAGCCATG---CTGTATTTTTGCTTTTCTATT

CTGGAGTTAAAAACCGCTACCCCCTTATTAAATAGAACGGCTGCACTTAAAGAACATGCT

ATTTTGATTATCCATAAAACCAACGCTCTTGTGTTTTTAGAAATGCTTAAAATTTTTGGA

CTTTTAAGCCAAGTGCACCATAACGATGTGTTAAAGATTTTAGAAAAAATACTTCAAAAT

>ZH13

GTGAGTTTGATTAAAGTTAGTGGTGATAAAAAAGCGATTGAGGTTTCTATTCCTTTAACT

------------TCAATTTCAGGCAAAGTGCGTGTGAAAATCAGACATGCCTTTAGCGAT

TATGGTGTTTCAACAGCGACTAGAAAAATCCCTTTTAGTTTAAAACATTATGTAGAGTGG

CAGATCGGTTATGATGTCCCCATTAAAGATAAAGAA---AAATTTGAACTCACTACTTTA

AAAGATGAAAAATATCATTTTTTAGGGGCTAATAATAAAGTAAAAACTCTTTATGAATTG

AGCGAAATGATTGATTACGCTAAGCAATTAGATTTAATCAGT---------TTAGAAAAT

TTAGAAAATACTTTAAAATATTTAGAAAAACAAAAACAATTTATAGAAGATAATTTTATG

ATTACAAGAGAAAGATTTAGATCGCATCAATTTGGTGGCATGGATTTTGAACTCTCACGC

ATTTCTTATCCTTTGCTCATTCATTCTTTTAATGATAATCAGTTGAGCGAAATTGTTATT

AGGGAACAACAATATGGTTCTAAAACCCAAGCCATG---CTGTATTTTTGCTTTTCTATT

TTGGAGTTAAAAACCGCTACCCCTTTATTAAATAGAACGGCTGCACTCAAAGAACATGCC

CTTTTGATTATCCATAAAACCAACGCTCTCATGTTTTTAGAAATGCTTAAAATTTTTGGA

CTTTTAAGCCAAGCACACCATAACGATGTGTTAAAGATTTTAGAAAAAATACTTCAAAAT

>ZH104

GTGAGTTTGATTAGGATTGATGATAGTAAAAAAGCGATTGAGGTTTCTATTCCTTTAACT

------------TCAATTTCAGGCAAAGTGCGTGTGAAAATCAGACATGCCTTTAGCGAT

TATGGCATTTCAACAGCAACTAGAAAAATCCCTTTTAGTTTAAAGCATTATGTAGAGTGG

CAAATCGGTTATGATGTCCCCATTAAAGATAAAGAA---AAATTTGAACTCACTACTTTA

AAAGATGAAAAATATTATTTTTTAGGGGCTAATAATAAAGTAAAGACTCTTTATGAATTG

AGTGAGATAATCTATTACGCTAAGCAATTAAATTTAATCAGT---------TTAGAAAAT

TTAGAAAATACTTTAAAATATTTAGAAAAACAAAAACAATTTATAGAAGATAATTTCACG

ATTACAAGAGAAAGATTTAGATCGCATCAATTTGGGGGCATGGATTTTGAACTTTCACGC

ATCTCTTATCCCTTACTCATTCATTCTTTTAATGATAATCAGTTGAGTGAAATCGTTATT

AGAGAGCAACAATATGGCTCTAAAGTCCAAGCCATG---CTGTATTTTTGCTTTTCTATT

TTGGAATTAAAAACCGCTACTCCCTTATTAAATAGAACGGCTGCACTCAAAGAACATGCT

TTTTTAACTATCCATAAAACCAACGCTCTTGTGTTTTTAGAAATGCTTAAAATTTTTGGA

CTTTTAAGCCAAGCGCACCATAACGATGTGTTAAAGATTTTAGAAAAAATACTTCAAAAT

>ZH125

GTGAGTTTGATTAGGATTGATAATAATAAAAAAGTAATTGGGGTTTCTATTCCTTTAACT

------------TCAATTTCAGGCAAAGTGCGTGTGAAAATCAGACATGCCTTTAGCGAT

TATGGTATTTCAACAGCGACCAGAAAAATCCCTTTTAGCTTAAAACATTATGTAGAGTGG

CAAATCGGTTATGATGTCCCCATTAAAGATAAAGAA---AAATTTGAGCTCACTACTTTA

AAAGATGAAAAATATCATTTTTTAGGGGCTAATAATAAAGTAAAAACTCTTTATGAATTA

AGCGAAATGATTTATTACGCTAAGCAATTAAATTTAATCAGT---------TTAGAAAAT

TTAGAAAATACTTTAAAATATTTAGAAAAACAAAAACAATTTATAGAAGATAGTTTTATG

ATTACAAGAGAAGGATTTAGATCGCATCAATTTGGTGGCATGGATTTTGAACTCTCACGC

ATTTCTTATCCTTTACTCATTCATTCTTTTAATGATAATCAGTTGAGCGAAATTGTTATT

AGAGAACAACAATATGGCTCTAAAACCCAAGCCATG---CTGTATTTTTGCTTTTCTATT

CTGGAATTAAAAACCGCTACCCCCTTATTAAATAGAACGGCTATGCTCAAAGAGCATGCT

CTTTTGATTATCCATAAAACCAACGCTCCCATGTTTTTAGAAATGCTTAAAATTTTTGGA

CTTTTAAGCCAAGCGCACCATAGCGATGTGTTAAAGATTTTAGAAAAAATACTTCAAAAT

>C-Mx-2006-664

GTGAGTTTGATTAAAGTTAATGATGATAAAAAAGCGATTGAGGTTTCTATTCCTTTAACT

------------TCCATTTCAGGCAAAGTGCGTGTGAAAATCAGGCATGCCTTTAGCGAT

TATGGCATTTCAACAGCGACTAGAAAAATCCCTTTTAGTTTAAAACATTATGTAGAGTGG

CAAATCGGTTATGATGTCCCCATTAAAGATAAAGAA---AAATTGGAGCTCACTACCCTA

AAAGATGAAAAATATCATTTTTTAGGGGCTAATAATAAAGTAAAAACCCTTTATGAATTG

AGTGAGATAATCTATTACGCTAAGCAATTAAATTTAATCAGT---------TTAGAAAAT

TTAGAAAATACTTTAAAATATTTAGAAAAACAAAAACAATTTATAGAAGATAATTTCACG

ATTACAAGAGAAAGATTTAGATCGCATCAATTTGGTGGCATGGATTTTGAACTTTCACGC

ATTTCTTACCCTTTACTCATTCATTCTTTCAATGATAATCAGTTGAGCGAAATCGTTATT

AGAGAGCAACAATACGGCTCTAAAACCCAAGCCATG---CTGTATTTTTGCTTTTCTATT

TTGGAATTAAAAACCGCTACTCCCTTATTAAACAGAACGGCTGCACTCAAAGAACATGCC

CTTTTAACTATCCATAAAACCAACGCTCTTGTGTTTTTAGAAATGCTTAAAATTTTTGGA

CTTTTAAGCCAAGCACACCATAACGATGTGTTAAAGATTTTAGAAAAAATACTTCAAAAT

>Nic50-A

GTGAGTTTGATTAAAGTTAATGATGATAAAAAAGTGATTGAGGTTTCTATTTCTTTAACT

------------TCCATTTCAGGCAAAGTTCGTGTGAAAATCAGACATGCCTTTAGCGAT

TATGGCATTTCAACAGCGACTAGAAAAATCCCTTTCAGTTTAAAGCATTATGTAGAGTGG

CAAATCGGTTATGATGTCCCTATTAAAGATAAAGAA---AAATTTGAGCTCACTACTTTA

AAAGATGAAAAATATCATTTTTTAGGAGCTAATAATAAAGTAAAAACCCTTTATGAATTA

AGCGAAATAATTGATTACGCTAAGCAATTAGGTTTAATCAGT---------TTAGAAAAT

TTAGAAAATACTTTAAAATATTTAGAAAAACAAAAACAATTTATAGAAGATAATTTTATG

ATCACAAGAGAAAGATTTAGATCCCATCAATTTGGTGGCATGGATTTTGAACTTTCACGC

ATTTCTTATCCTTTACTCATTCATTCTTTCAACGATAATCAGTTGAGCGAAATCGTTATT

AGAGAGCAACAATACGGCTCTAAAACCCAAGCCATG---CTATATTTTTGCTTTTCTATT

CTGGAATTAAAAACCGCTACCCCCTTATTAAATAGAACCGCTGCCCTCAAAGAACATGCC

CTTTTAACTATCCATAAAACCAACGCTCTTATGTTTTTAGAAATGCTTAAAATTTTTGGA

CTTTTAAGCCAAGTGCACCATAACGATGTGTTAAAGATTTTAGAAAAAATACTTGAAAAT

>KH0176

GTGAGTTTGATTAAAGTCAGTGATGATAAAAAAGCGATTGAGGTTTCTATTCCTTTAACT

------------TCAATTTCAGGCAAAGTGCGTGTGAAAATCAGACATGCCTTTAGCGAT

TATGGTATTTCAACAGCGACTAGAAAAATCCCTTTTAGTTTAAAACATTATGTAGAGTGG

CAAATCGGCTATGATGTCCCCATTAAAGATAAAGAA---AAATTTGAGCTCACTACCCTA

AAAGATGAAAAATATCATTTTTTAGGGGCTAATAATAAAGTAAAAACCCTTTATGAATTG

AGTGAGATAATCTATTACGCTAAGCAATTAAATTTAATCAGT---------TTAGAAAAT

TTAGAAAATACTTTAAAATATTTAGAAAAACAAAAACAATTTATAGAAGATAATTTCACG

ATTACAAGAGAAAGATTTAGATCGCATCAATTTGGTGGCATGGATTTTGAACTCTCACGC

ATTTCTTATCCCTTACTCATTCATTCTTTTAATGATAATCAGTTGAGTGAAATCGTTATT

AGAGAGCAACAATATGGCTCTAAAACCCAAGCCATG---CTGTATTTTTGCTTTTCTATT

TTGGAATTAAAAACCACTACCCCCTTATTAAATAGAACGGCTGCACTCAAAGAACATGCC

CTTTTAACTATCCATAAAACCAACGCTCTTATGTTTTTAGAAATGCTTAAAATTTTTGGA

CTTTTAAGCCAAGCACACCATAGCGATGTGTTAAAGATTTTAGAAAAAATACTTCAAAAT

>MHP36

GTGAGTTTGATTAGGATTGATGATAGTAAAAAAGCGATTGAGGTTTCTATTCCTTTAACT

------------TCAATTTCAGGCAAAGTGCGTGTGAAAATCAGACATGCCTTTAGCGAT

TATGGCATTTCAACAGCGACTAGAAAAATCCCTTTTAGTTTAAAACATTATGTAGAGTGG

CAAATCGGTTATGATGTCCCCATTAAAGATAAAGAA---AAATTTGAACTCACTACCCTA

AAAGATGAAAAATATCATTTTTTAGGGGCTAATAATAAAATAAAAACCCTTTATGAATTG

AGCGAAATAATTTATTACGCTAAGCGATTGGGTTTAATCAGT---------TTAGAAAAT

TTAGAAAATACTTTAAAATATTTAGAAAAACAAAAACAATTCATAGAAGATAGTTTTATG

ATCACAAGAGAAAGATTTAGATCGCATCAATTTGGGGGCATGGATTTTGAACTTTCACGC

ATTTCTTATCCCTTACTCATTCATTCTTTTAATGATAATCAATTGAGCGAAATCGTTATT

AGAGAGCAACAATACGGCTCTAAAACCCAAGCCATG---CTGTATTTTTGCTTTTCTATT

TTGGAGTTAAAAACCGCTACCCCCTTATTAAACAGAACGGCTATGCTCAAAGAGCATGCT

CTTTTGATTATCCATAAAACCAACGCTCTCATGTTTTTAGAAATGCTTAAAATTTTTGGA

CTTTTAAGCCAAGCGCACCATAACTATGTGTTAAAGATTTTAGAAAAAATACTTCAAAAT

>45

GTGAGTTTGATTAGGATTGATGATAGTAAAAAAGCGATTGAGGTTTCCATTCCTTTAACT

------------TCAATTTCAGGCAAAGTGCGTGTGAAAATCAGGCATGCCTTTAGCGAT

TATGGCATTTCAACAGCGACCAGAAAAATCCCTTTTAGCTTAAAGCATTATGTAGAGTGG

CAAATCGGTTATGATGTCCCCATTAAAGATAAAGAA---AAATTTGAATTCACTACTTTA

AAAGATGAAAAATATCATTTTTTAGGGGCTAATAATAAAGTAAAAACCCTTTATGAATTG

AGTGAGATAATCTATTACGCTAAGCAATTAAATTTAATCAGT---------TTAGAAAAT

TTAGAAAATACTTTAAAATATTTAGAAAAACAAAAACAATTCATAGAAGATAGTTTTATG

ATTACAAGAGAAAGATTTAGATCGCATCAATTTGGTGGCATGGATTTTGAACTTTCACGC

ATTTCTTACCCTTTACTCATTCATTCTTTCAACGATAATCAATTGAGTGAAGTCGTTATT

AGAGAGCAACAATACTGCTCTAAAACCCAAGCCATG---CTGTATTTTTGCTTTTCTGTT

TTGGAGTTAAAAACCGCTACCCCCTTATTAAACAGAACGGCTGCACTCAAAGAACATGCC

CTTTTAACTATCCATAAAACCAACGCTCCCATGTTTTTAGAAATGCTTAAAATTTTTGGA

CTTTTAAGCCAAGCGCACCATAACGATGTGTTAAAGATTTTAGAAAAAATACTTCAAAAT

>MHP37

GTGAGTTTGATTAGGATTGATGATAGTAAAAAAGCGATTGAGGTTTCTATTCCTTTAACT

------------TCAATTTCAGGCAAAGTGCGTGTGAAAATCAGACATGCCTTTAGCGAT

TATGGTATTTCAACAGCGACTAGAAAAATCCCTTTTAGTTTAAAACATTATGTAGAGTGG

CAGATCGGTTATGATGTCCCTATTAAAGATAAAGAA---AAATTTGAACTCACTACTTTA

AAAGATGAAAAATATCATTTTTTAGGGGCTAATAATAAAGTAAAAACCCTTTATGAATTG

AGTGAGATAATCTATTACGCTAAGCAATTAAATTTAATCAGT---------TTAGAAAAT

TTAGAAAATACTTTAAAATATTTAGAAAAACAAAAACAATTTATAGAAGATAATTTCACG

ATTACAAGAGAAAGATTTAGATCGCATCAATTTGGTGGCATGGATTTTGAACTCTCACGC

ATTTCTTATCCTTTACTCATTCATTCTTTTAACGATAATCAATTGAGTGAAATCGTTATT

AGAGAACAACAATACGGCTCTAAAACCCAAGCCATG---CTGTATTTTTGCTTTTCTATT

TTGGAGTTAAAAACCGCTACCCCCTTATTAAACAGAACGGCTATGCTCAAAGAGCATGCT

CTTTTGATTATCCATAAAACCAACGCTCCCATGTTTTTAGAAATGCTTAAAATTTTTGGA

CTTTTAAGCCAAGCGCACCATAACGATGTGTTAAAGATTTTAGAAAAGATACTTCAAAAT

>26-A-EK1

GTGAGTTTGATTAAAGTTAGTGGTGATAAAAAAGTGATTGAGGTTTCCATTCCTTTAACT

------------TCAATTTCAGGCAAAGTGCGTGTGAAAATCAGACATGCCTTTAGCGAT

TATGGTATTTCAACAGCGACTAGAAAAATCCCTTTTAGTTTAAAACATTATATAGAGTGG

CAGATCGGTTATGATGTCCCCATTAAAGATAAAGAA---AAATTTGAACTCACTACTTTA

AAAGATGAAAAATATCATTTTTTAGGGGCTAATGATAAAGTAAAAACTCTTTATGAATTA

AGCGAAATGATTGATTACGCTAAGCAATTAGGTTTAATCAGT---------TTAGAAAAT

TTAGAAAATACTTTAAAATATTTAGAAAAACAAAAACAATTCATAGAAGATAATTTCACG

ATTACAAGAGAAAGATTTAGATTACATCAATTTGGTGGCATGGATTTTGAACTCTCACGC

ATTTCTTATCCTTTGCTCATTCATTCTTTTAATGATAATCAGTTGAGCGAAATTGTTATT

AGGGAACAACAATATGGCTCTAAAACCCAAGCCATG---CTGTATTTTTGCTTTTCTATT

TTGGAATTAAAAACCGCTACTCCCTTATTAAACAGAACGGCTGCACTCAAAGAACATGCC

CTTTTAACTATCCATAAAACCAACGCTCCCATGTTTTTAGAAATGCTTAAAATTTTTGGA

CTTTTAAGCCAAGCGCACCATAACGATGTGTTAAAGATTTTAGAAAAAATACTTCAAAAT

>C-Mx-2010-3

GTGAGTTTGATTAGGATTGATAATAATAAAAAAGTAATTGGGGTTTCTATTCCTTTAACT

------------TCAATTTCAGGCAAAGTGTGTGTGAAAATCAGACATGCCTTTAGCGAT

TATGGTATTTCAACAGCGACCAGAAAAATCCCTTTTAGCTTAAAACATTATGTAGAGTGG

CAAATCGGTTATGATGTCCCCATTAAAGATAAAGAA---AAATTTGAGCTCACTACTTTA

AAAGATGAAAAATATCATTTTTTAGGGGCTAATAATAAAGTAAAAACCCTTTATGAATTG

AGTGAGATAATCTATTACGCTAAGCAATTAAATTTAATCAGT---------TTAGAAAAT

TTAGAAAATACTTTAAAATATTTAGAAAAACAAAAACAATTTATAGAAGATAATTTCACG

ATTACAAGAGAAAGATTTAGATTACATCAATTTGGTGGCATGGATTTTGAACTCTCACGC

ATTTCTTATCCTTTGCTCATTCATTCTTTTAATGATAATCAGTTGAGCGAAATCGTTATT

AGAGAACAACAATATGGCTCTAAAACCCAAGCCATG---CTGTATTTTTGCTTTTCTATT

CTGGAATTAAAAACCGCTACCCCTTTATTAAATAGAACGGCTGCACTCAAAGAACATGCT

CTTTTGATTATCCATAAAACCAACGCTCTTGTGTTTTTAGAAATGCTTAAAATTTTTGGA

CTTTTAAGCCAAGCGCACCATAACGATGTGTTAAAGATTTTAGAAAAAATACTTCAAAAT

>C-Mx-2010-5

GTGAGTTTGATTAGGATTGATAATAATAAAAAAGTAATTGGGGTTTCTATTCCTTTAACT

------------TCAATTTCAGGCAAAGTGTGTGTGAAAATCAGACATGCCTTTAGCGAT

TATGGTATTTCAACAGCGACCAGAAAAATCCCTTTTAGCTTAAAACATTATGTAGAGTGG

CAAATCGGTTATGATGTCCCCATTAAAGATAAAGAA---AAATTTGAGCTCACTACTTTA

AAAGATGAAAAATATCATTTTTTAGGGGCTAATAATAAAGTAAAAACCCTTTATGAATTG

AGTGAGATAATCTATTACGCTAAGCAATTAAATTTAATCAGT---------TTAGAAAAT

TTAGAAAATACTTTAAAATATTTAGAAAAACAAAAACAATTTATAGAAGATAATTTCACG

ATTACAAGAGAAAGATTTAGATTACATCAATTTGGTGGCATGGATTTTGAACTCTCACGC

ATTTCTTATCCTTTGCTCATTCATTCTTTTAATGATAATCAGTTGAGCGAAATCGTTATT

AGAGAACAACAATATGGCTCTAAAACCCAAGCCATG---CTGTATTTTTGCTTTTCTATT

CTGGAATTAAAAACCGCTACCCCTTTATTAAATAGAACGGCTGCACTCAAAGAACATGCT

CTTTTGATTATCCATAAAACCAACGCTCTTGTGTTTTTAGAAATGCTTAAAATTTTTGGA

CTTTTAAGCCAAGCGCACCATAACGATGTGTTAAAGATTTTAGAAAAAATACTTCAAAAT

>ZH3

GTGAGTTTGATTAGGATTGATAATAATAAAAAAGTAATTGGGGTTTCTATTCCTTTAACT

------------TCCATTTCAGGCAAAGTGCGTGTGAAAATCAGACATGCCTTTAGCGAT

TATGGCATTTCAACAGCGACTAGAAAAATCCCTTTTAGTTTAAAGCATTATGTAGAGTGG

CAAATCGGTTATGATGTCCCCATTAAAGATAAAGAA---AAATTTGAACTCACTACCCTA

AAAGATGAAAAATATCATTTTTTAGGGGCTAATAATAAAAGAAAAACCCTTTATGAATTG

AGTGAAATAATTTATTACGCTAAGCGATTAGGTTTAATCAGT---------TTAGAAAAT

TTAGAAAATACTTTAAAATATTTAGAAAAACAAAAACAATTTATAGAAGATAATTTTATG

ATTACAAGAGAAAGATTTAGATCGCATCAATTTGGTGGCATGGATTTTGAACTCTCACAC

ATTTCTTACCCTTTACTCATTCATTCTTTCAATGATAATCAATTGAGTGAAATCGTTATT

AGAGAGCAACAATATGGCTCTAAAACCCAAGCCATG---CTGTATTTTTGCTTTTCTATT

TTGGAATTAAAAACCGCTACTCCCTTATTAAATAGAACGGCTGCACTCAAAGAACATACT

CTTTTAACCATCAATAAAACCAACGCTCTTGTGTTTTTAGAAATGCTTAAAATTTTTGGA

CTTTTAAGTCAAGTGCACCATAACGATGTGTTAAAGATTTTAGAAAAAATACTTCAAAAT

>HP06059

GTGAGTTTGATTAAAGTTAATGATGATAAAAAAGTGATTGAGGTTTCTATTCCTTTAACT

------------TCCATTTCAGGCAAAGTTCGTGTGAAAATCAGACATGCCTTTAGCGAT

TATGGCATTTCAACAGCGACTAGAAAAATCCCTTTCAGTTTAAAGCATTATGTAGAGTGG

CAAATTGGTTATGATGTCCCCATTAAAGATAAAGAA---AAATTTGAACTCACTACCCTA

AAAGATGAAAAATATCATTTTTTAGGGGCTAATAATAAAATAAAAACCCTTTATGAATTG

AGCGAAATAATTTATTACGCTAAGCGATTGGATTTAATCAGT---------TTAGAAAAT

TTAGAAAATACTTTAAAATATTTAGAAAAACAAAAACAATTCATAGAAGATAGTTTTATG

ATCACAAGAGAAAGATTTAGATCGCATCAATTTGGTGGCATGGATTTTGAACTTTCACGC

ATTTCTTATCCTTTACTCATTCATTCTTTCAACGATAATCAGTTGAGCGAAATCGTTATT

AGAGAGCAACAATACGGCTCTAAAACCCAAGCCATG---CTATATTTTTGCTTTTCTATT

CTGGAATTAAAAACCGCTACCCCCTTATTAAATAGAACGGCTACACTCAAAGAACATGCC

CTTTTAACTATCCATAAAACCAACGCTCTTATGTTTTTAGAAATGCTTAAAATTTTTGGA

CTTTTAAGCCAAGCGCACCATAACGATGTGTTAAAGATTTTAGAAAAAATACTTGAAAAT

>22

GTGAGTTTGATTAGGATTGATGATAGTAAAAAAGCGATTGAGGTTTCCATTCCTTTAACT

------------TCAATTTCAGGCAAAGCGCGTGTGAAAATCAGACATGCCTTTAGCGAT

TATGGCATTTCAACAGCGACTAGAAAAATCCCTTTTAGCTTAAAACATTATGTAGAGTGG

CAAATCGGTTATGATGTCCCCATTAAAGATAAAGAA---AAATTTGAACTCACTACTTTA

AAAGATGAAAAATATCATTTTTTAGGGGCTAATAATAAAGTAAAAACCCTTTATGAATTG

AGTGAGATAATCTATTACGCTAAGCAATTAGATTTAATCAGT---------TTAGAAAAT

TTAGAAAATACTTTAAAATATTTAGAAAAACAAAAACAATTCATAGAAGATAGTTTTATG

ATTACAAGAGAAAGATTTAGATTACATCAATTTGGTGGCATGGATTTTGAACTCTCACGC

ATTTCTTATCCTTTGCTCATTCATTCTTTTAATGATAATCAGTTGAGCGAAATCGTTATT

AGAGAACAACAATATGGCTCTAAAACCCAAGCCATG---CTGTATTTTTGCTTTTCTATT

CTGGAATTAAAAACCGCTACCCCTTTATTAAATAGAACGGCTGCACTCAAAGAACATGCC

CTTTTAACTATCCATAAAACCAACGCTCTTATGTTTTTAGAAATGCTTAAAATTTTTGGA

CTTTTAAGCCAAGTGCACCATAGCGATGTGTTAAAGATTTTAGAAAAAATACTTCAAAAT

>G-Mx-2006-53

GTGAGTTTGATTAGGATTGATGATAGTAAAAAAGCGATTGAGGTTTCTATTCCTTTAACT

------------TCAATTTCAGGCAAAGCGCGTGTGAAAATCAGACATGCCTTTAGCGAT

TATGGTATTTCAACAGCGACCAAAAAAATCCCTTTTAGTTTAAAACATTATATAGAGTGG

CAGATCGGTTATGATGTCCCCATTAAAGATAAAGAA---AAATTTGAACTCACTACTTTA

AAAGATGAAAAATATCATTTTTTAGGGGCTAATAATAAAGTAAAAACCCTTTATGAATTG

AGTGAGATAATCTATTACGCTAAGCAATTAAATTTAATCAGT---------TTAGAAAAT

TTAGAAAATACTTTAAAATATTTAGAAAAACAAAAACAATTTATAGAAGATAATTTCACG

ATTACAAGAGAAAGATTTAGATCGCATCAATTTGGTGGCATGGATTTTGAACTTTCACGC

ATTTCTTATCCTTTACTCATTCATTCTTTTAATGATAATCAATTGAGTGAAATCGTTATT

AGAGAGCAACAATATGGCTCTAAAACCCAAGCCATG---CTGTATTTTTGCTTTTCTATT

TTGGAGTTAAAAACCGCTACCCCTTTATTAAATAGAACGGCTGCACTCAAAGAACAGGCT

CTTTTAACCATCCATAAAACCAACGCTCTTATGTTTTTAGAAATGCTTAAAATTTTTGGA

CTTTTAAGCCAAGCGCACCATAGCGATGTGTTAAAGATTTTAGAAAAAATACTTCAAAAT

>SV340_2

GTGAGTTTGATTAGGATTGATGATAGTAAAAAAGCGATTGAGGTTTCTGTTCCTTTAACT

------------TCCATTTCAGGCAAAGCGCGTGTGAAAATCAGACATGCCTTTAGCGAT

TATGGCATTTCAACAGCGACTAGAAAAATCCCTTTTAGTTTAAAACATTATGTAGAGTGG

CAAATCGGTTATGATGTCCCCATTAAAGATAAAGAA---AAATTTGAACTCACTACCCTA

AAAGATGAAAAATATCATTTTTTAGGGGCTAATAATAAAATAAAAACCCTTTATGAATTG

AGCGAAATAATTGATTACGCTAAGCGATTGGGTTTAATCAGT---------TTAGAAAAT

TTAGAAAATACTTTAAAATATTTAGAAAAACAAAAACAATTCATAGGAGATAATTTTATG

ATCACAAGAGAAAGATTTAGATCGCATCAATTTGGTGGCATGGATTTTGAACTTTCACGC

ATCTCTTATCCCTTACTCATTCATTCTTTTAATGATAATCAATTGAGCGAAATCGTTATT

AGAGAGCAACAATACGGCTCTAAAACCCAAGCCATG---CTATATTTTTGCTTTTCTATT

CTGGAATTAAAAACCGCTACCCCCTTATTAAATAGAACCGCTGCCCTCAAAGAACATGCC

CTTTTAACTATCCATAAAACCAACGCTCTTATGTTTTTAGAAATGCTTAAAATTTTTGGA

CTTTTAAGCCAAGCGCACCATAACGATGTGTTAAAGATTTTAGAAAAAATACTTGAAAAT

>ZH117

GTGAATTTGATTAAGATTGATAATGATAAAAAAGTGATTGAGGTTTCTATTCCTTTAACT

------------TCAATTTCAGGCAAAGCGCGTGTGAAAATCAGACATGCCTTTAGCGAT

TATGGCATTTCAACAGCGACCAGAAAAATCCCTTTTAGTTTAAAGCATTATGTAGAGTGG

CAAATCGGTTATGATGTCCCTATTAAAGATAAAGAA---AAATTTGAGCTCACTACCCTA

AAAGATGAAAAATATCATTTTTTAGGGGCTAATAATAAAGTAAAAACCCTTTATGAATTG

AGTGAAATAATTGATTACGCTAAGCGATTGGGTTTAATCGGT---------TTAGAAAAT

TTAGAAAATACTTTAAAATATTTAGAAAAACAAAAACAATTCATAGAAGATAATTTCACG

ATTACAAGAGAAAGATTTAGATCGCATCAATTTGGTGGCATGGATTTTGAACTTTCACGC

ATTTCTTACCCTTTACTCATTCATTCTTTCAATGATAATCAGTTGAGCGAAATCGTTATT

AGAGAGCAACAATACGGCTCTAAAACCCAAGCCATG---CTGTATTTTTGCTTTTCTATT

TTGGAATTAAAAACCGCTACTCCCTTATTAAACAGAACGGCTGCACTCAAAGAACATGCC

CTTTTAACTATCCATAAAACCAACGCTCTTGTGTTTTTAGAAATGCTTAAAATTTTTGGA

CTTTTAAGCCAAGCACACCATAACGATGTGTTAAAGATTTTAGAAAAAATACTTCAAAAT

>29C8

GTGAGTTTGATTAAAGTTAGTGGTGATAAAAAAGCGATTGAGGTTTCTATTCCCTTAACT

------------TCAATTTCAGGTAAAGTGCGTGTGAAAATCAGACATGCCTTTAGCGAT

TATGGTGTTTCAACAGCGACTAGAAAAATCCCTTTTAGTTTAAAGCATTATGTAGAGTGG

CAAATCGGTTATGATGTCCCCATTAAAGATAAAGAA---AAATTTGAGCTCACTACCCTA

AAAGATGAAAAATATCATTTTTTAGGGGCTAATAATAAAGTAAAAACTCTTTATGAATTG

AGCGAGATCATTTACTATGCCAAGCAATTAGATTTAATCAGT---------TTAGAAAAT

TTAGAAAATACTTTAAAATATTTAGAAAAACAAAAACAATTTATAGAAGATAATTTTATG

ATTACAAGAGAAAGATTTAGATCGCATCAATTTGGTGGCATGGATTTTGAACTTTCACGC

ATTTCTTATCCTTTACTCATTCATTCTTTTAATGATAATCAATTGAGCGAAATTGTTATT

AGAGAACAACAATACGGCTCTAAAACCCAAGCCATG---CTGTATTTTTGCTTTTCTATT

TTGGAGTTAAAAACCGCTACTCCCTTATTAAATAGAACGGCTGCTCTCAAAGAACATGCC

CTTTTAACTATCCATAAAACCAACGCTCCCATGTTTTTAGAAATGCTTAAAATTTTTGGA

CTTTTAAGCCAAGCGCACCATGACGATGTGTTAAAGATTTTAGAAAAGATACTTCAAAAT

>119A2

GTGAGTTTGATTAAAGTTAGTGGTGATAAAAAAGCGATTGAGGTTTCTATTCCCTTAACT

------------TCAATTTCAGGTAAAGTGCGTGTGAAAATCAGACATGCCTTTAGCGAT

TATGGTGTTTCAACAGCGACTAGAAAAATCCCTTTTAGTTTAAAGCATTATGTAGAGTGG

CAAATCGGTTATGATGTCCCCATTAAAGATAAAGAA---AAATTTGAGCTCACTACCCTA

AAAGATGAAAAATATCATTTTTTAGGGGCTAATAATAAAGTAAAAACTCTTTATGAATTG

AGCGAGATCATTTACTATGCCAAGCAATTAGATTTAATCAGT---------TTAGAAAAT

TTAGAAAATACTTTAAAATATTTAGAAAAACAAAAACAATTTATAGAAGATAATTTTATG

ATTACAAGAGAAAGATTTAGATCGCATCAATTTGGTGGCATGGATTTTGAACTTTCACGC

ATTTCTTATCCTTTACTCATTCATTCTTTTAATGATAATCAATTGAGCGAAATTGTTATT

AGAGAACAACAATACGGCTCTAAAACCCAAGCCATG---CTGTATTTTTGCTTTTCTATT

TTGGAGTTAAAAACCGCTACTCCCTTATTAAATAGAACGGCTGCTCTCAAAGAACATGCC

CTTTTAACTATCCATAAAACCAACGCTCCCATGTTTTTAGAAATGCTTAAAATTTTTGGA

CTTTTAAGCCAAGCGCACCATGACGATGTGTTAAAGATTTTAGAAAAGATACTTCAAAAT

>125C7

GTGAGTTTGATTAAAGTTAGTGGTGATAAAAAAGCGATTGAGGTTTCTATTCCCTTAACT

------------TCAATTTCAGGTAAAGTGCGTGTGAAAATCAGACATGCCTTTAGCGAT

TATGGTGTTTCAACAGCGACTAGAAAAATCCCTTTTAGTTTAAAGCATTATGTAGAGTGG

CAAATCGGTTATGATGTCCCCATTAAAGATAAAGAA---AAATTTGAGCTCACTACCCTA

AAAGATGAAAAATATCATTTTTTAGGGGCTAATAATAAAGTAAAAACTCTTTATGAATTG

AGCGAGATCATTTACTATGCCAAGCAATTAGATTTAATCAGT---------TTAGAAAAT

TTAGAAAATACTTTAAAATATTTAGAAAAACAAAAACAATTTATAGAAGATAATTTTATG

ATTACAAGAGAAAGATTTAGATCGCATCAATTTGGTGGCATGGATTTTGAACTTTCACGC

ATTTCTTATCCTTTACTCATTCATTCTTTTAATGATAATCAATTGAGCGAAATTGTTATT

AGAGAACAACAATACGGCTCTAAAACCCAAGCCATG---CTGTATTTTTGCTTTTCTATT

TTGGAGTTAAAAACCGCTACTCCCTTATTAAATAGAACGGCTGCTCTCAAAGAACATGCC

CTTTTAACTATCCATAAAACCAACGCTCCCATGTTTTTAGAAATGCTTAAAATTTTTGGA

CTTTTAAGCCAAGCGCACCATGACGATGTGTTAAAGATTTTAGAAAAGATACTTCAAAAT

>8C10

GTGAGTTTGATTAAAGTTAGTGGTGATAAAAAAGCGATTGAGGTTTCTATTCCCTTAACT

------------TCAATTTCAGGTAAAGTGCGTGTGAAAATCAGACATGCCTTTAGCGAT

TATGGTGTTTCAACAGCGACTAGAAAAATCCCTTTTAGTTTAAAGCATTATGTAGAGTGG

CAAATCGGTTATGATGTCCCCATTAAAGATAAAGAA---AAATTTGAGCTCACTACCCTA

AAAGATGAAAAATATCATTTTTTAGGGGCTAATAATAAAGTAAAAACTCTTTATGAATTG

AGCGAGATCATTTACTATGCCAAGCAATTAGATTTAATCAGT---------TTAGAAAAT

TTAGAAAATACTTTAAAATATTTAGAAAAACAAAAACAATTTATAGAAGATAATTTTATG

ATTACAAGAGAAAGATTTAGATCGCATCAATTTGGTGGCATGGATTTTGAACTTTCACGC

ATTTCTTATCCTTTACTCATTCATTCTTTTAATGATAATCAATTGAGCGAAATTGTTATT

AGAGAACAACAATACGGCTCTAAAACCCAAGCCATG---CTGTATTTTTGCTTTTCTATT

TTGGAGTTAAAAACCGCTACTCCCTTATTAAATAGAACGGCTGCTCTCAAAGAACATGCC

CTTTTAACTATCCATAAAACCAACGCTCCCATGTTTTTAGAAATGCTTAAAATTTTTGGA

CTTTTAAGCCAAGCGCACCATGACGATGTGTTAAAGATTTTAGAAAAGATACTTCAAAAT

>103A4

GTGAGTTTGATTAAAGTTAGTGGTGATAAAAAAGCGATTGAGGTTTCTATTCCCTTAACT

------------TCAATTTCAGGTAAAGTGCGTGTGAAAATCAGACATGCCTTTAGCGAT

TATGGTGTTTCAACAGCGACTAGAAAAATCCCTTTTAGTTTAAAGCATTATGTAGAGTGG

CAAATCGGTTATGATGTCCCCATTAAAGATAAAGAA---AAATTTGAGCTCACTACCCTA

AAAGATGAAAAATATCATTTTTTAGGGGCTAATAATAAAGTAAAAACTCTTTATGAATTG

AGCGAGATCATTTACTATGCCAAGCAATTAGATTTAATCAGT---------TTAGAAAAT

TTAGAAAATACTTTAAAATATTTAGAAAAACAAAAACAATTTATAGAAGATAATTTTATG

ATTACAAGAGAAAGATTTAGATCGCATCAATTTGGTGGCATGGATTTTGAACTTTCACGC

ATTTCTTATCCTTTACTCATTCATTCTTTTAATGATAATCAATTGAGCGAAATTGTTATT

AGAGAACAACAATACGGCTCTAAAACCCAAGCCATG---CTGTATTTTTGCTTTTCTATT

TTGGAGTTAAAAACCGCTACTCCCTTATTAAATAGAACGGCTGCTCTCAAAGAACATGCC

CTTTTAACTATCCATAAAACCAACGCTCCCATGTTTTTAGAAATGCTTAAAATTTTTGGA

CTTTTAAGCCAAGCGCACCATGACGATGTGTTAAAGATTTTAGAAAAGATACTTCAAAAT

>8A3

GTGAGTTTGATTAAAGTTAGTGGTGATAAAAAAGCGATTGAGGTTTCTATTCCCTTAACT

------------TCAATTTCAGGTAAAGTGCGTGTGAAAATCAGACATGCCTTTAGCGAT

TATGGTGTTTCAACAGCGACTAGAAAAATCCCTTTTAGTTTAAAGCATTATGTAGAGTGG

CAAATCGGTTATGATGTCCCCATTAAAGATAAAGAA---AAATTTGAGCTCACTACCCTA

AAAGATGAAAAATATCATTTTTTAGGGGCTAATAATAAAGTAAAAACTCTTTATGAATTG

AGCGAGATCATTTACTATGCCAAGCAATTAGATTTAATCAGT---------TTAGAAAAT

TTAGAAAATACTTTAAAATATTTAGAAAAACAAAAACAATTTATAGAAGATAATTTTATG

ATTACAAGAGAAAGATTTAGATCGCATCAATTTGGTGGCATGGATTTTGAACTTTCACGC

ATTTCTTATCCTTTACTCATTCATTCTTTTAATGATAATCAATTGAGCGAAATTGTTATT

AGAGAACAACAATACGGCTCTAAAACCCAAGCCATG---CTGTATTTTTGCTTTTCTATT

TTGGAGTTAAAAACCGCTACTCCCTTATTAAATAGAACGGCTGCTCTCAAAGAACATGCC

CTTTTAACTATCCATAAAACCAACGCTCCCATGTTTTTAGAAATGCTTAAAATTTTTGGA

CTTTTAAGCCAAGCGCACCATGACGATGTGTTAAAGATTTTAGAAAAGATACTTCAAAAT

>78A3

GTGAGTTTGATTAAAGTTAGTGGTGATAAAAAAGCGATTGAGGTTTCTATTCCCTTAACT

------------TCAATTTCAGGTAAAGTGCGTGTGAAAATCAGACATGCCTTTAGCGAT

TATGGTGTTTCAACAGCGACTAGAAAAATCCCTTTTAGTTTAAAGCATTATGTAGAGTGG

CAAATCGGTTATGATGTCCCCATTAAAGATAAAGAA---AAATTTGAGCTCACTACCCTA

AAAGATGAAAAATATCATTTTTTAGGGGCTAATAATAAAGTAAAAACTCTTTATGAATTG

AGCGAGATCATTTACTATGCCAAGCAATTAGATTTAATCAGT---------TTAGAAAAT

TTAGAAAATACTTTAAAATATTTAGAAAAACAAAAACAATTTATAGAAGATAATTTTATG

ATTACAAGAGAAAGATTTAGATCGCATCAATTTGGTGGCATGGATTTTGAACTTTCACGC

ATTTCTTATCCTTTACTCATTCATTCTTTTAATGATAATCAATTGAGCGAAATTGTTATT

AGAGAACAACAATACGGCTCTAAAACCCAAGCCATG---CTGTATTTTTGCTTTTCTATT

TTGGAGTTAAAAACCGCTACTCCCTTATTAAATAGAACGGCTGCTCTCAAAGAACATGCC

CTTTTAACTATCCATAAAACCAACGCTCCCATGTTTTTAGAAATGCTTAAAATTTTTGGA

CTTTTAAGCCAAGCGCACCATGACGATGTGTTAAAGATTTTAGAAAAGATACTTCAAAAT

>125A3

GTGAGTTTGATTAAAGTTAGTGGTGATAAAAAAGCGATTGAGGTTTCTATTCCCTTAACT

------------TCAATTTCAGGTAAAGTGCGTGTGAAAATCAGACATGCCTTTAGCGAT

TATGGTGTTTCAACAGCGACTAGAAAAATCCCTTTTAGTTTAAAGCATTATGTAGAGTGG

CAAATCGGTTATGATGTCCCCATTAAAGATAAAGAA---AAATTTGAGCTCACTACCCTA

AAAGATGAAAAATATCATTTTTTAGGGGCTAATAATAAAGTAAAAACTCTTTATGAATTG

AGCGAGATCATTTACTATGCCAAGCAATTAGATTTAATCAGT---------TTAGAAAAT

TTAGAAAATACTTTAAAATATTTAGAAAAACAAAAACAATTTATAGAAGATAATTTTATG

ATTACAAGAGAAAGATTTAGATCGCATCAATTTGGTGGCATGGATTTTGAACTTTCACGC

ATTTCTTATCCTTTACTCATTCATTCTTTTAATGATAATCAATTGAGCGAAATTGTTATT

AGAGAACAACAATACGGCTCTAAAACCCAAGCCATG---CTGTATTTTTGCTTTTCTATT

TTGGAGTTAAAAACCGCTACTCCCTTATTAAATAGAACGGCTGCTCTCAAAGAACATGCC

CTTTTAACTATCCATAAAACCAACGCTCCCATGTTTTTAGAAATGCTTAAAATTTTTGGA

CTTTTAAGCCAAGCGCACCATGACGATGTGTTAAAGATTTTAGAAAAGATACTTCAAAAT

>48A2

GTGAGTTTGATTAAAGTTAGTGGTGATAAAAAAGCGATTGAGGTTTCTATTCCCTTAACT

------------TCAATTTCAGGTAAAGTGCGTGTGAAAATCAGACATGCCTTTAGCGAT

TATGGTGTTTCAACAGCGACTAGAAAAATCCCTTTTAGTTTAAAGCATTATGTAGAGTGG

CAAATCGGTTATGATGTCCCCATTAAAGATAAAGAA---AAATTTGAGCTCACTACCCTA

AAAGATGAAAAATATCATTTTTTAGGGGCTAATAATAAAGTAAAAACTCTTTATGAATTG

AGCGAGATCATTTACTATGCCAAGCAATTAGATTTAATCAGT---------TTAGAAAAT

TTAGAAAATACTTTAAAATATTTAGAAAAACAAAAACAATTTATAGAAGATAATTTTATG

ATTACAAGAGAAAGATTTAGATCGCATCAATTTGGTGGCATGGATTTTGAACTTTCACGC

ATTTCTTATCCTTTACTCATTCATTCTTTTAATGATAATCAATTGAGCGAAATTGTTATT

AGAGAACAACAATACGGCTCTAAAACCCAAGCCATG---CTGTATTTTTGCTTTTCTATT

TTGGAGTTAAAAACCGCTACTCCCTTATTAAATAGAACGGCTGCTCTCAAAGAACATGCC

CTTTTAACTATCCATAAAACCAACGCTCCCATGTTTTTAGAAATGCTTAAAATTTTTGGA

CTTTTAAGCCAAGCGCACCATGACGATGTGTTAAAGATTTTAGAAAAGATACTTCAAAAT

>48C8

GTGAGTTTGATTAAAGTTAGTGGTGATAAAAAAGCGATTGAGGTTTCTATTCCCTTAACT

------------TCAATTTCAGGTAAAGTGCGTGTGAAAATCAGACATGCCTTTAGCGAT

TATGGTGTTTCAACAGCGACTAGAAAAATCCCTTTTAGTTTAAAGCATTATGTAGAGTGG

CAAATCGGTTATGATGTCCCCATTAAAGATAAAGAA---AAATTTGAGCTCACTACCCTA

AAAGATGAAAAATATCATTTTTTAGGGGCTAATAATAAAGTAAAAACTCTTTATGAATTG

AGCGAGATCATTTACTATGCCAAGCAATTAGATTTAATCAGT---------TTAGAAAAT

TTAGAAAATACTTTAAAATATTTAGAAAAACAAAAACAATTTATAGAAGATAATTTTATG

ATTACAAGAGAAAGATTTAGATCGCATCAATTTGGTGGCATGGATTTTGAACTTTCACGC

ATTTCTTATCCTTTACTCATTCATTCTTTTAATGATAATCAATTGAGCGAAATTGTTATT

AGAGAACAACAATACGGCTCTAAAACCCAAGCCATG---CTGTATTTTTGCTTTTCTATT

TTGGAGTTAAAAACCGCTACTCCCTTATTAAATAGAACGGCTGCTCTCAAAGAACATGCC

CTTTTAACTATCCATAAAACCAACGCTCCCATGTTTTTAGAAATGCTTAAAATTTTTGGA

CTTTTAAGCCAAGCGCACCATGACGATGTGTTAAAGATTTTAGAAAAGATACTTCAAAAT

>78C8

GTGAGTTTGATTAAAGTTAGTGGTGATAAAAAAGCGATTGAGGTTTCTATTCCCTTAACT

------------TCAATTTCAGGTAAAGTGCGTGTGAAAATCAGACATGCCTTTAGCGAT

TATGGTGTTTCAACAGCGACTAGAAAAATCCCTTTTAGTTTAAAGCATTATGTAGAGTGG

CAAATCGGTTATGATGTCCCCATTAAAGATAAAGAA---AAATTTGAGCTCACTACCCTA

AAAGATGAAAAATATCATTTTTTAGGGGCTAATAATAAAGTAAAAACTCTTTATGAATTG

AGCGAGATCATTTACTATGCCAAGCAATTAGATTTAATCAGT---------TTAGAAAAT

TTAGAAAATACTTTAAAATATTTAGAAAAACAAAAACAATTTATAGAAGATAATTTTATG

ATTACAAGAGAAAGATTTAGATCGCATCAATTTGGTGGCATGGATTTTGAACTTTCACGC

ATTTCTTATCCTTTACTCATTCATTCTTTTAATGATAATCAATTGAGCGAAATTGTTATT

AGAGAACAACAATACGGCTCTAAAACCCAAGCCATG---CTGTATTTTTGCTTTTCTATT

TTGGAGTTAAAAACCGCTACTCCCTTATTAAATAGAACGGCTGCTCTCAAAGAACATGCC

CTTTTAACTATCCATAAAACCAACGCTCCCATGTTTTTAGAAATGCTTAAAATTTTTGGA

CTTTTAAGCCAAGCGCACCATGACGATGTGTTAAAGATTTTAGAAAAGATACTTCAAAAT

>87A3

GTGAGTTTGATTAAAGTTAGTGGTGATAAAAAAGCGATTGAGGTTTCTATTCCCTTAACT

------------TCAATTTCAGGTAAAGTGCGTGTGAAAATCAGACATGCCTTTAGCGAT

TATGGTGTTTCAACAGCGACTAGAAAAATCCCTTTTAGTTTAAAGCATTATGTAGAGTGG

CAAATCGGTTATGATGTCCCCATTAAAGATAAAGAA---AAATTTGAGCTCACTACCCTA

AAAGATGAAAAATATCATTTTTTAGGGGCTAATAATAAAGTAAAAACTCTTTATGAATTG

AGCGAGATCATTTACTATGCCAAGCAATTAGATTTAATCAGT---------TTAGAAAAT

TTAGAAAATACTTTAAAATATTTAGAAAAACAAAAACAATTTATAGAAGATAATTTTATG

ATTACAAGAGAAAGATTTAGATCGCATCAATTTGGTGGCATGGATTTTGAACTTTCACGC

ATTTCTTATCCTTTACTCATTCATTCTTTTAATGATAATCAATTGAGCGAAATTGTTATT

AGAGAACAACAATACGGCTCTAAAACCCAAGCCATG---CTGTATTTTTGCTTTTCTATT

TTGGAGTTAAAAACCGCTACTCCCTTATTAAATAGAACGGCTGCTCTCAAAGAACATGCC

CTTTTAACTATCCATAAAACCAACGCTCCCATGTTTTTAGAAATGCTTAAAATTTTTGGA

CTTTTAAGCCAAGCGCACCATGACGATGTGTTAAAGATTTTAGAAAAGATACTTCAAAAT

>H1

GTGAGTTTGATTAAAGTTAGTGGTGATAAAAAAGCGATTGAGGTTTCTATTCCCTTAACT

------------TCAATTTCAGGTAAAGTGCGTGTGAAAATCAGACATGCCTTTAGCGAT

TATGGTGTTTCAACAGCGACTAGAAAAATCCCTTTTAGTTTAAAGCATTATGTAGAGTGG

CAAATCGGTTATGATGTCCCCATTAAAGATAAAGAA---AAATTTGAGCTCACTACCCTA

AAAGATGAAAAATATCATTTTTTAGGGGCTAATAATAAAGTAAAAACTCTTTATGAATTG

AGCGAGATCATTTACTATGCCAAGCAATTAGATTTAATCAGT---------TTAGAAAAT

TTAGAAAATACTTTAAAATATTTAGAAAAACAAAAACAATTTATAGAAGATAATTTTATG

ATTACAAGAGAAAGATTTAGATCGCATCAATTTGGTGGCATGGATTTTGAACTTTCACGC

ATTTCTTATCCTTTACTCATTCATTCTTTTAATGATAATCAATTGAGCGAAATTGTTATT

AGAGAACAACAATACGGCTCTAAAACCCAAGCCATG---CTGTATTTTTGCTTTTCTATT

TTGGAGTTAAAAACCGCTACTCCCTTATTAAATAGAACGGCTGCTCTCAAAGAACATGCC

CTTTTAACTATCCATAAAACCAACGCTCCCATGTTTTTAGAAATGCTTAAAATTTTTGGA

CTTTTAAGCCAAGCGCACCATGACGATGTGTTAAAGATTTTAGAAAAGATACTTCAAAAT

>119C10

GTGAGTTTGATTAAAGTTAGTGGTGATAAAAAAGCGATTGAGGTTTCTATTCCCTTAACT

------------TCAATTTCAGGTAAAGTGCGTGTGAAAATCAGACATGCCTTTAGCGAT

TATGGTGTTTCAACAGCGACTAGAAAAATCCCTTTTAGTTTAAAGCATTATGTAGAGTGG

CAAATCGGTTATGATGTCCCCATTAAAGATAAAGAA---AAATTTGAGCTCACTACCCTA

AAAGATGAAAAATATCATTTTTTAGGGGCTAATAATAAAGTAAAAACTCTTTATGAATTG

AGCGAGATCATTTACTATGCCAAGCAATTAGATTTAATCAGT---------TTAGAAAAT

TTAGAAAATACTTTAAAATATTTAGAAAAACAAAAACAATTTATAGAAGATAATTTTATG

ATTACAAGAGAAAGATTTAGATCGCATCAATTTGGTGGCATGGATTTTGAACTTTCACGC

ATTTCTTATCCTTTACTCATTCATTCTTTTAATGATAATCAATTGAGCGAAATTGTTATT

AGAGAACAACAATACGGCTCTAAAACCCAAGCCATG---CTGTATTTTTGCTTTTCTATT

TTGGAGTTAAAAACCGCTACTCCCTTATTAAATAGAACGGCTGCTCTCAAAGAACATGCC

CTTTTAACTATCCATAAAACCAACGCTCCCATGTTTTTAGAAATGCTTAAAATTTTTGGA

CTTTTAAGCCAAGCGCACCATGACGATGTGTTAAAGATTTTAGAAAAGATACTTCAAAAT

>29A2

GTGAGTTTGATTAAAGTTAGTGGTGATAAAAAAGCGATTGAGGTTTCTATTCCCTTAACT

------------TCAATTTCAGGTAAAGTGCGTGTGAAAATCAGACATGCCTTTAGCGAT

TATGGTGTTTCAACAGCGACTAGAAAAATCCCTTTTAGTTTAAAGCATTATGTAGAGTGG

CAAATCGGTTATGATGTCCCCATTAAAGATAAAGAA---AAATTTGAGCTCACTACCCTA

AAAGATGAAAAATATCATTTTTTAGGGGCTAATAATAAAGTAAAAACTCTTTATGAATTG

AGCGAGATCATTTACTATGCCAAGCAATTAGATTTAATCAGT---------TTAGAAAAT

TTAGAAAATACTTTAAAATATTTAGAAAAACAAAAACAATTTATAGAAGATAATTTTATG

ATTACAAGAGAAAGATTTAGATCGCATCAATTTGGTGGCATGGATTTTGAACTTTCACGC

ATTTCTTATCCTTTACTCATTCATTCTTTTAATGATAATCAATTGAGCGAAATTGTTATT

AGAGAACAACAATACGGCTCTAAAACCCAAGCCATG---CTGTATTTTTGCTTTTCTATT

TTGGAGTTAAAAACCGCTACTCCCTTATTAAATAGAACGGCTGCTCTCAAAGAACATGCC

CTTTTAACTATCCATAAAACCAACGCTCCCATGTTTTTAGAAATGCTTAAAATTTTTGGA

CTTTTAAGCCAAGCGCACCATGACGATGTGTTAAAGATTTTAGAAAAGATACTTCAAAAT

>81A1

GTGAGTTTGATTAAAGTTAGTGGTGATAAAAAAGCGATTGAGGTTTCTATTCCCTTAACT

------------TCAATTTCAGGTAAAGTGCGTGTGAAAATCAGACATGCCTTTAGCGAT

TATGGTGTTTCAACAGCGACTAGAAAAATCCCTTTTAGTTTAAAGCATTATGTAGAGTGG

CAAATCGGTTATGATGTCCCCATTAAAGATAAAGAA---AAATTTGAGCTCACTACCCTA

AAAGATGAAAAATATCATTTTTTAGGGGCTAATAATAAAGTAAAAACTCTTTATGAATTG

AGCGAGATCATTTACTATGCCAAGCAATTAGATTTAATCAGT---------TTAGAAAAT

TTAGAAAATACTTTAAAATATTTAGAAAAACAAAAACAATTTATAGAAGATAATTTTATG

ATTACAAGAGAAAGATTTAGATCGCATCAATTTGGTGGCATGGATTTTGAACTTTCACGC

ATTTCTTATCCTTTACTCATTCATTCTTTTAATGATAATCAATTGAGCGAAATTGTTATT

AGAGAACAACAATACGGCTCTAAAACCCAAGCCATG---CTGTATTTTTGCTTTTCTATT

TTGGAGTTAAAAACCGCTACTCCCTTATTAAATAGAACGGCTGCTCTCAAAGAACATGCC

CTTTTAACTATCCATAAAACCAACGCTCCCATGTTTTTAGAAATGCTTAAAATTTTTGGA

CTTTTAAGCCAAGCGCACCATGACGATGTGTTAAAGATTTTAGAAAAGATACTTCAAAAT

>12C8

GTGAGTTTGATTAAAGTTAGTGGTGATAAAAAAGCGATTGAGGTTTCTATTCCCTTAACT

------------TCAATTTCAGGTAAAGTGCGTGTGAAAATCAGACATGCCTTTAGCGAT

TATGGTGTTTCAACAGCGACTAGAAAAATCCCTTTTAGTTTAAAGCATTATGTAGAGTGG

CAAATCGGTTATGATGTCCCCATTAAAGATAAAGAA---AAATTTGAGCTCACTACCCTA

AAAGATGAAAAATATCATTTTTTAGGGGCTAATAATAAAGTAAAAACTCTTTATGAATTG

AGCGAGATCATTTACTATGCCAAGCAATTAGATTTAATCAGT---------TTAGAAAAT

TTAGAAAATACTTTAAAATATTTAGAAAAACAAAAACAATTTATAGAAGATAATTTTATG

ATTACAAGAGAAAGATTTAGATCGCATCAATTTGGTGGCATGGATTTTGAACTTTCACGC

ATTTCTTATCCTTTACTCATTCATTCTTTTAATGATAATCAATTGAGCGAAATTGTTATT

AGAGAACAACAATACGGCTCTAAAACCCAAGCCATG---CTGTATTTTTGCTTTTCTATT

TTGGAGTTAAAAACCGCTACTCCCTTATTAAATAGAACGGCTGCTCTCAAAGAACATGCC

CTTTTAACTATCCATAAAACCAACGCTCCCATGTTTTTAGAAATGCTTAAAATTTTTGGA

CTTTTAAGCCAAGCGCACCATGACGATGTGTTAAAGATTTTAGAAAAGATACTTCAAAAT

>87C7

GTGAGTTTGATTAAAGTTAGTGGTGATAAAAAAGCGATTGAGGTTTCTATTCCCTTAACT

------------TCAATTTCAGGTAAAGTGCGTGTGAAAATCAGACATGCCTTTAGCGAT

TATGGTGTTTCAACAGCGACTAGAAAAATCCCTTTTAGTTTAAAGCATTATGTAGAGTGG

CAAATCGGTTATGATGTCCCCATTAAAGATAAAGAA---AAATTTGAGCTCACTACCCTA

AAAGATGAAAAATATCATTTTTTAGGGGCTAATAATAAAGTAAAAACTCTTTATGAATTG

AGCGAGATCATTTACTATGCCAAGCAATTAGATTTAATCAGT---------TTAGAAAAT

TTAGAAAATACTTTAAAATATTTAGAAAAACAAAAACAATTTATAGAAGATAATTTTATG

ATTACAAGAGAAAGATTTAGATCGCATCAATTTGGTGGCATGGATTTTGAACTTTCACGC

ATTTCTTATCCTTTACTCATTCATTCTTTTAATGATAATCAATTGAGCGAAATTGTTATT

AGAGAACAACAATACGGCTCTAAAACCCAAGCCATG---CTGTATTTTTGCTTTTCTATT

TTGGAGTTAAAAACCGCTACTCCCTTATTAAATAGAACGGCTGCTCTCAAAGAACATGCC

CTTTTAACTATCCATAAAACCAACGCTCCCATGTTTTTAGAAATGCTTAAAATTTTTGGA

CTTTTAAGCCAAGCGCACCATGACGATGTGTTAAAGATTTTAGAAAAGATACTTCAAAAT

>12A3

GTGAGTTTGATTAAAGTTAGTGGTGATAAAAAAGCGATTGAGGTTTCTATTCCCTTAACT

------------TCAATTTCAGGTAAAGTGCGTGTGAAAATCAGACATGCCTTTAGCGAT

TATGGTGTTTCAACAGCGACTAGAAAAATCCCTTTTAGTTTAAAGCATTATGTAGAGTGG

CAAATCGGTTATGATGTCCCCATTAAAGATAAAGAA---AAATTTGAGCTCACTACCCTA

AAAGATGAAAAATATCATTTTTTAGGGGCTAATAATAAAGTAAAAACTCTTTATGAATTG

AGCGAGATCATTTACTATGCCAAGCAATTAGATTTAATCAGT---------TTAGAAAAT

TTAGAAAATACTTTAAAATATTTAGAAAAACAAAAACAATTTATAGAAGATAATTTTATG

ATTACAAGAGAAAGATTTAGATCGCATCAATTTGGTGGCATGGATTTTGAACTTTCACGC

ATTTCTTATCCTTTACTCATTCATTCTTTTAATGATAATCAATTGAGCGAAATTGTTATT

AGAGAACAACAATACGGCTCTAAAACCCAAGCCATG---CTGTATTTTTGCTTTTCTATT

TTGGAGTTAAAAACCGCTACTCCCTTATTAAATAGAACGGCTGCTCTCAAAGAACATGCC

CTTTTAACTATCCATAAAACCAACGCTCCCATGTTTTTAGAAATGCTTAAAATTTTTGGA

CTTTTAAGCCAAGCGCACCATGACGATGTGTTAAAGATTTTAGAAAAGATACTTCAAAAT

>81C9

GTGAGTTTGATTAAAGTTAGTGGTGATAAAAAAGCGATTGAGGTTTCTATTCCCTTAACT

------------TCAATTTCAGGTAAAGTGCGTGTGAAAATCAGACATGCCTTTAGCGAT

TATGGTGTTTCAACAGCGACTAGAAAAATCCCTTTTAGTTTAAAGCATTATGTAGAGTGG

CAAATCGGTTATGATGTCCCCATTAAAGATAAAGAA---AAATTTGAGCTCACTACCCTA

AAAGATGAAAAATATCATTTTTTAGGGGCTAATAATAAAGTAAAAACTCTTTATGAATTG

AGCGAGATCATTTACTATGCCAAGCAATTAGATTTAATCAGT---------TTAGAAAAT

TTAGAAAATACTTTAAAATATTTAGAAAAACAAAAACAATTTATAGAAGATAATTTTATG

ATTACAAGAGAAAGATTTAGATCGCATCAATTTGGTGGCATGGATTTTGAACTTTCACGC

ATTTCTTATCCTTTACTCATTCATTCTTTTAATGATAATCAATTGAGCGAAATTGTTATT

AGAGAACAACAATACGGCTCTAAAACCCAAGCCATG---CTGTATTTTTGCTTTTCTATT

TTGGAGTTAAAAACCGCTACTCCCTTATTAAATAGAACGGCTGCTCTCAAAGAACATGCC

CTTTTAACTATCCATAAAACCAACGCTCCCATGTTTTTAGAAATGCTTAAAATTTTTGGA

CTTTTAAGCCAAGCGCACCATGACGATGTGTTAAAGATTTTAGAAAAGATACTTCAAAAT

>103C8

GTGAGTTTGATTAAAGTTAGTGGTGATAAAAAAGCGATTGAGGTTTCTATTCCCTTAACT

------------TCAATTTCAGGTAAAGTGCGTGTGAAAATCAGACATGCCTTTAGCGAT

TATGGTGTTTCAACAGCGACTAGAAAAATCCCTTTTAGTTTAAAGCATTATGTAGAGTGG

CAAATCGGTTATGATGTCCCCATTAAAGATAAAGAA---AAATTTGAGCTCACTACCCTA

AAAGATGAAAAATATCATTTTTTAGGGGCTAATAATAAAGTAAAAACTCTTTATGAATTG

AGCGAGATCATTTACTATGCCAAGCAATTAGATTTAATCAGT---------TTAGAAAAT

TTAGAAAATACTTTAAAATATTTAGAAAAACAAAAACAATTTATAGAAGATAATTTTATG

ATTACAAGAGAAAGATTTAGATCGCATCAATTTGGTGGCATGGATTTTGAACTTTCACGC

ATTTCTTATCCTTTACTCATTCATTCTTTTAATGATAATCAATTGAGCGAAATTGTTATT

AGAGAACAACAATACGGCTCTAAAACCCAAGCCATG---CTGTATTTTTGCTTTTCTATT

TTGGAGTTAAAAACCGCTACTCCCTTATTAAATAGAACGGCTGCTCTCAAAGAACATGCC

CTTTTAACTATCCATAAAACCAACGCTCCCATGTTTTTAGAAATGCTTAAAATTTTTGGA

CTTTTAAGCCAAGCGCACCATGACGATGTGTTAAAGATTTTAGAAAAGATACTTCAAAAT

>HP11043

GTGAGTTTGATTAGGATTGATGATAGTAAAAAAGCGATTGAGGTTTCTATTCCTTTAACT

------------TCAATTTCAGGCAAAGTGCGTGTGAAAATCAGACATGCCTTTAGCGAT

TATGGTATTTCAACAGCGACCAGAAAAATCCCTTTTAGCTTAAAACATTATGTAGAGTGG

CAAATCGGTTATGATGTCCCCATTAAAGATAAAGAA---AAATTTGAGCTCACTACCCTA

AAAGATGAAAAATATCATTTTTTAGGGGCTAATAATAAAGTGAAAACTCTTTATGAATTA

AGCGAAATGATTTATTACGCTAAGCAATTAGGTTTAATTGGT---------TTAGAAAAT

TTAGAAAATACTTTAAAATATTTAGAAAAACAAAAACAATTTATAGAAGATAATTTCACG

ATTACAAGAGAAAGATTTAGATCGCATCAATTTGGTGGCATGGATTTTGAACTCTCACGC

ATTTCTTATCCTTTGCTCATTCATTCTTTTAATGATAATGAGTTGAGCGAAATAGTTATT

AGAGAACAACAATATGGCTCTAAAACCCAAGCCATG---CTGTATTTTTGCTTTTCTATT

TTGGAGTTAAAAACCGCTACTCCCTTATTAAATAGAACGGCTGCACTCAAAGAACATGCC

CTTTTGATTGTCCGTCAAACTAACGCTTCCATGTTTTTAGAAATGCTTAAAATTTTTGGA

CTTTTAAGCCAAGCGCACCATAACGATGTGTTAAAGATTTTAGAAAAAATACTTCAAAAT

>2004-51

GTGAGTTTGATTAAAGTTAATGATGATAAAAAAGCGATTGAGGTTTCTATTCCTTTAACT

------------TCCATTTCAGGCAAAGCGCGTGTGAAAATCAGACATGCCTTTAGCGAT

TATGGCATTTCAACAGCGACTAGAAAAATCCCTTTCAGTTTAAAGCATTATGTAGAGTGG

CAAATCGGTTATGATGCCCCCATTAAAGATAAAGAA---AAATTTGAACTCACTACCCTA

AAAGATGAAAAATATCATTTTTTAGGAGCTAATAATAAAGTAAAAACCCTTTATGAATTG

AGCGAAATAATTTATTACGCTAAGCGATTGGGTTTAATCAGT---------TTAGAAAAT

TTAGAAAATACTTTAAAATATTTAGAAAAACAAAAACAATTCATAGAAGATAATTTTATG

ATTACAAGAGAAAGATTTAGATCGCATCAATTTGGGGGCATGGATTTTGAACTTTCACGC

ATTTCTTATCCTTTGCTCATTCATTCTTTTAATGATAATCAGTTGAGCGAAATCGTTATT

AGAGAACAACAATACGGCTCTAAAACCCAAGCCATG---CTGTATTTTTGCTTTTCTATT

TTGGAATTAAAAACCGCTACCCCTTTATTAAATAGAACGGCTGCCCTCAAAGAACACGCT

CTTTTAATTATCCATAAAACCAACGCTCTTGTGTTTTTAGAAATGCTTAAAATTTTTGGT

CTTTTAAGCCAAGCGCATCATAACGATGTGTTAAAGATTTTAGAAAAAATACTTCAAAAT

>GC43-HL

GTGAGTTTGATTAAAGCTAGTGGTGATAAAAAAGTGATCGAGGTTTCCATTCCTTTAACT

------------TCAATTTCAGGCAAAGTGCGTGTGAAAATCAGGCATGCCTTTAGCGAT

TATGGTATTTCAACAGCGACTAGAAAAATCCCTTTTAGTTTAAAACATTATGTAGAGTGG

CAGATCGGTTATGATGTCCCCATTAAAGATAAAGAA---AAATTTGAACTCACTACTTTA

AAAGATGAAAAATATCATTTTTTAGGGGCTAACAATAAAGTAAAAACTCTTTATGAATTA

AGCGAAATGATTTATTACGCTAAGCAATTGGGTTTAATCAGT---------TTAGAAAAT

TTAGAAAATACTTTAAAATATTTAGAAAAACAAAAACAATTTATAGAAGATAATTTTATG

ATTACAAGAGAAAGACTTAGATCGCATCAATTTGGTGGAATGGATTTTGAACTCTCACGC

ATTTCTTATCCTTTGCTCATTCATTCTTTCAACGATAACCAATTGAGCGAAATAGTTATT

AGAGAACAACAATATGGCTCTAAAACCCAAGCCATG---CCGTATTTTTGCTTTTCTATT

TTGGAATTAAAAACCGCTACCCCTTTATTAAATAGAACCGCTACACTCAAAGAACATGCT

TTTTTAACTATCCATAAAACCAACGCTCTTGTGTTTTTAGAAATGCTTAAAATTTTTGGA

CTTTTAAGCCAAGCGCACCATAACGATGTGTTAAAGATTTTAGAAAAAATACTTCAAAAT

>ZH35

GTGAGTTTGATTAAAATTAACCATGATAAGAAAGTGATCGAGATTTCTATTCCTTTAACT

------------TCAATTTCAGGCAAAGTGCGTGTGAAAATCAGACATGCCTTTAGCGAT

TATGGTATTTCAACAGCGACTAGAACAATCCCTTTTAGTTTAAAACATTATGTAGAGTGG

CAAATCGGTTATGATGTCCCCATTAAAGATAAAGAA---AAATTTGAACTCACTACTTTA

AAAGATGAAAAATATCATTTTTTAGGGGCTAATAATAAAGTAAAGACTCTTTATGAATTG

AGCGAAATGATTTATTACGCTAAGCGATTGGGTTTAATCAGT---------TTAGAAAAT

TTAGAAAATACTTTAAAATATTTAGAAAAACAAAAACAATTTATAGAAGATAATTTTATG

ATTACAAGAGAAAGATTTAGATCGCATCAATTTGGTGGCATGGATTTTGAACTCTCACGC

ATTTCTTATCCCTTACTTATTCATTCTTTTAATGATAATCAGTTGAGCGAAATTGTTATT

AGAGAGCAACAATATGGCTCTAAAACCCAAGCCATG---CTGTATTTTTGCTTTTCTATT

TTGGAGTTAAAAACCGCTACCCCCTTATTAAACAGAACGGCTATGCTCAAAGAGCATGCT

CTTTTGATTATCCATAAAACCAACGCTCTCATGTTTTTAGAAATGCTTAAAATTTTTGGA

CTTTTAAGCCAAGTGCACCATAGCGATGTGTTAAAGATTTTAGAAAAAATACTTCAAAAT

>KH4

GTGAGTTTGATTAAAGTTGACTATGATAAAAAAGTGATTAAGGTTTCTATTCCTCTAACT

------------TCAATTTCAGGCAAAGTGCGTGTGAAAATCAGACATGCCTTTAGCGAT

TATGGTATTTCAACAGCGACTAGAAAAATCCCTTTTAGTTTAAAACATTATGTAGAGTGG

CAGATCGGTTATGATGTCCCCATTAAAGATAAGGAA---AAATTTGAACTCACTGCTTTA

AAAGATAAAAAATATCATTTTTTAGGGGCTAATAATAAGGTAAAAACTCTTTATGAATTG

AGCGAAATGATTTATTACGCTAAGCAATTAGGTTTAATCAGT---------TTAGAAAAT

TTAGAAAATACTTTAAAATATTTAGAAAAACAAAAACAATTTATAGAAGATAATTTTATG

ATTGCAAGAGAAGGATTTAGATCGCATCAATTTGGTGGCATGGATTTTGAACTCTCACGC

ATTTCTTATCCTTTACTCATTCATTCTTTTAACGATAATCAGTTGAGTGAAATAGTTATT

AGAGAACAACAATACGGCTCTAAGACTCAAGCCATG---CTGTATTTTTGCTTTTCTATT

TTGGAGTTAAAAACCGCTACCCCCTTATTAAATAGAACCGCTACACTCAAAGAACATGCT

TTTTTAACCATCCATAAAACCAACGCTCTCATGTTTTTAGAAATGCTTAAAATTTTTGGA

CTTTTAAGCCAAGCGCACCATAACGATGTGTTAAAGATTTTAGAAAAAATACTTCAAAAT

>KH10

GTGAGTTTGATTAAAGTTGACTATGATAAAAAAGTGATTGAGGTTTCTATTCCTTTAACT

------------TCAATTTCAGGCAAAGTGCGTGTGAAGATCAGACATGCCTTTAGCGAT

TATGGTATTTCAACAGCGACTAGAAAAATCCCTTTTAGTTTAAAGCATTATGTAGAGTGG

CAGATCGGTTATGATGTCCCCATTAAAGATAAAGAA---AAATTTGAACTCACTGCTTTA

AAAGATAAAAAATACCATTTTTTAGGGGCTAATAATAAAGTAAAAACTCTTTATGAATTG

AGCGAAATGATTTATTACGCTAAGCAATTAGGTTTAATCAGT---------TTAGAAAAT

TTAGAAAATACTTTAAAATATTTAGAAAAACAAAAACAATTTATAGAAGATAATTTTATG

ATTACAAGAGAAAGATTTAGATCGCATCAATTTGGTGGCATGGATTTTGAACTCTCACAC

ATTTCTTATCCTTTACTCATTCATTCTTTTAACGATAATCAATTGAGTGAAATAGTTATT

AGAGAACAACAATATGGCTCTAAGACCCAAGCCATG---CTGTATTTTTGCTTTTCTATT

TTGGAGTTAAAAACCGCTACCCCCTTATTAAATAGAACCGCTGCACTCAAAGAACATGCT

CTTTTGATTATTCATCAAACTAACGCTTCCATGTTTTTAGAAATGCTTAAAATTTTTGGA

CTTTTAAGTCAAGCGCACCATAACGATGTGGTGAAGATTTTAGAAAAAATACTTCAAAAT

>GC65-HL

GTGAGTTTGATTAGGATTGATGATAGTAAAAAAGCGATTGAGGTTTCTATTCCTTTAACT

------------TCAATTTCAGGCAAAGCACGTGTGAAAATCAGGCATGCCTTTAGCGAT

TATGGCATTTCAACAGCGACTAGAAAAATCCCTTTTAGTTTAAAACATTATATAGAGTGG

CAAATCGGTTATGATGCCCCCATTAAAGATAAAGAA---AAATTTAAACTCACTACTTTA

AAAGATGAAAAATATCATTTTTTAGGGGCTAATAATAAAGTAAAGACTCTTTATGAATTG

AGTGAGATAATCTATTACGCTAAGCAATTAAATTTAATCAGT---------TTAGAAAAT

TTAGAAAATACTTTAAAATATTTAGAAAAACAAAAACAATTTATAGAAGATAATTTTATG

ATTACAAGAGAAAGATTTAGATTACATCAATTTGGTGGCATGGATTTTGAACTCTCACGC

ATCTCTTATCCTTTACTCATTCATTCTTTTAATGATAATCAGTTGAGCGAAATTATTATT

AGAGAACAACAATATGGCTCTAAAACCCAAGCCATG---CTGTATTTTTGCTTTTCTATT

TTGGAATTAAAAACCGCTACCCCTTTATTAAATAGAACGGCTGCACTCAAAGAACATGCT

TTTTTAACCATCCATAAAACCAACGCTCTCATGTTTTTAGAAATGCTTAAAATTTTTGGA

CTTTTAAGCCAAGCGCACCATAACGATGTGTTAAAGATTTTAGAAAAAATACTTCAAAAT

>KH18

GTGAGTTTGATTAGGATTGATGATAGTAAAAAAGTAATTGAGGTTTCTATTCCTTTAACT

------------TCAATTTCAGGCAAAGCGCGTGTGAAAATCAGACATGCCTTTAGCGAT

TATGGTATTTCAACAGCGACTAGAAAAATCCCTTTTAGTTTAAAGCATTATGTAGAGTGG

CAAATCGGTTATGATGTCCCCATTAAAGATAAAGAA---AAATTGGAGCTCACTACCCTA

AAAGATGAAAAATATCATTTTTTAGGGGCTAATAATAAAGTAAAAACCCTTTATGAATTA

AGCGAAATGATTTATTACGCTAAGCAATTAAATTTAATCAGT---------TTAGAAAAT

TTAGAAAATACTTTAAAATATTTAGAAAAACAAAAACAATTTATAGAAGATAATTTCATG

ATTACAAGAGAAGGATTCAGATCGCATCAATTTGGTGGCATGGATTTTGAACTTTCACGC

ATTTCTTATCCCTTACTCATTCATTCTTTTAATGATAATCAATTGAGCGAAATCGTTATT

AGAGAACAACAATACGGCTCTAAGACACAAGCCATG---CTGTATTTTTGCTTTTCTATT

TTGGAGTTAAAAACCGCTACTCCCTTATTAAATAGAACCGCTACGCTCAAAGAACATGCC

CTTTTGATTGTCCATCAAACTAACGCTCTTGTGTTTTTAGAAATGCTTAAAATTTTTGGG

CTTTTAAGCCAAGCGCACCATAACGATGTGTTAAAGATTTTAGAAAAAATACTTCAAAAT

>Nic44-A

GTGAGTTTGATTAAAGTTAATGATGATAAAAAAGTAATTGAGGTTTCTATTCCTTTAACT

------------TCCATTTCAGGCAAAACGCGTGTGAAAATCAGACACGCCTTTAGCGAT

TATGGTATTTCAACAGCGACTAGAAAAATCCCTTTCAGTTTAAAGCATTATGTAGAGTGG

CAAATCGGCTATGATGTCCCCATTAAAGATAAAGAA---AAATTTGAACTCACTACTTTA

AAAGATGAAAAATATCATTTTTTAGGGGCTAATAATAAAGTAAAAACCCTTTATGAATTG

AGCGAAATAATTTATTACGCTAAGCGATTGGGTTTAATCAGT---------TTAGAAAAT

TTAGAAAATACTTTAAAATATTTAGAAAAACAAAAACAATTCATAGAAGATAGTTTTATG

ATTACAAGAGAAAGATTTAGATCGCATCAATTTGGGGGCATGGATTTTGAACTTTCACGC

ATTTCTTACCCTTTACTCATTCATTCTTTTAATGATAATCAGTTGAGCGAAATCGTTATT

AGAGAGCAACAATACGGCTCTAAAACCCAAGCCATG---CTATATTTTTGCTTTTCTATT

TTGGAGTTAAAAACCGCTACACCCTTATTAAATAGGACCGCTGCCCTCAAAGAACACGCT

CTTTTAACTATCCATAAAACCAACGCTCCCATGTTTTTAGAAATGCTTAAAATTTTTGGC

CTCTTAAGCCAAGCGCACCATAACGATGTGTTAAAGATTTTAAAAAAAATACTTGAAAAT

>ZH132

GTGAGTTTGATTAAAGTCAGTGATGATAAAAAAGCGATTGAGGTTTCTATTCCTTTAACT

------------TCAATTTCAGGCAAAGTGCGTGTGAAAATCAGACATGCCTTTAGCGAT

TATGGTATTTCAACAGCGACTAGAAAAATCCCTTTTAGTTTAAAACATTATGTAGAGTGG

CAAATCGGTTATGATGTCCCTATTAAAGATAAAGAA---AAATTTGAGCTCACTACTTTA

AAAGATGAAAAATATCATTTTTTAGGGGCTAACAATAAAGTAAAAACCCTTTATGAATTG

AGTGAGATAATCTATTATGCTAAGCAATTAAATTTAATCAGT---------TTAGAAAAT

TTAGAAAATACTTTAAAATATTTAGAAAAACAAAAACAATTTATAGAAGATAATTTCACG

ATTACAAGAGAAAGATTTAGATCGCATCAATTTGGTGGCATGGATTTTGAACTTTCACGC

ATTTCTTATCCTTTACTCATTCATTCTTTCAATGATAATCAATTGAGTGAAATCGTTATT

AGAGAGCAACAATATGGCTCTAAAACCCAAGCCATG---CTGTATTTTTGCTTTTCTATT

TTGGAATTAAAAACCGCTACCCCCTTATTAAATAGAACGGCTGCACTCAAAGAACATGCT

CTTTTAACCATCAATAAAACCAACGCTCTTATGTTTTTAGAAATGCTTAAAATTTTTGGA

CTTTTAAGCCAAGCACACCATAGCGATGTGTTAAAGATTTTAGAAAAAATACTTCAAAAT

>HP12068

GTGAGTTTGATTAAGATTGATAATAATAAAAAAGTAATTGAGATTTCTATTCCTTTAACT

------------TCAATTTCAGGCAAAGCGCGTGTGAAAATCAGACATGCCTTTAGCGAT

TATGGTATTTCAACAGCGACCAGAAAAATCCCTTTTAGTTTAAAACATTATGTAGAGTGG

CAAATCGGTTATGATGTCCCCATTAAAGATAAAGAA---AAATTTGAACTCACTACTTTA

AAAGATGAAAAATATCATTTTTTAGGGGCTAATAATAAAGTAAAAACCCTTTATGAATTG

AGTGAGATAATCTATTACGCTAAGCAATTAAATTTAATCAGT---------TTAGAAAAT

TTAGAAAATACTTTAAAATATTTAGAAAAACAAAAACAATTTATAGAAGATAATTTCACG

ATTACAAGAGAAAGATTTAGATTACATCAATTTGGTGGCATGGATTTTGAACTTTCACGC

ATTTCTTATCCTTTACTCATTCATTCTTTCAATGATAATCAATTGAGTGAAATCGTTATT

AGAGAGCAACAATATGGCTCTAAAACCCAAGCCATG---CTGTATTTTTGCTTTTCTATT

TTGGAATTAAAAACCGCTACCCCCTTATTAAATAGAACGGCTGCACTCAAAGAACATGCT

TTTTTAATTATCCATAAAACTAACGCTCTTGTGTTTTTAGAAATGCTTAAAATTTTTGGA

CTTTTAAGCCAAGCGCACCATAGCGATGTGTTAAAGATTTTAGAAAAAATACTTCAAAAT

>HP16001

GTGAGTTTGATTAAAGTTAGTGGTGATAAAAAAGTGATTGAGGTTTCTATTCCTTTAACT

------------TCAATTTCAGGCAAAGTGCGTGTGAAAATCAGACATGCCTTTAGCGAT

TATGGTGTTTCAACAGCGACTAGAAAAATCCCTTTTAGTTTAAAACATTATGTAGAGTGG

CAGATCGGTTATGATGTCCCCATTAAAGATAAAGAA---AAATTTGAACTCACTACTTTA

AAAGATGAAAAATATCATTTTTTAGGGGCTAATAATAAAGTAAAAACTCTTTATGAATTG

AGCGAAATGATTTATTACGCTAAGCAATTAGGTTTAATCAGT---------TTAGAAAAT

TTAGAAAATACTTTAAAATATTTAGAAAAACAAAAACAATTTATAGAAGATAATTTCACG

ATTACAAGAGAAAGATTTAGATCGCATCAATTTGGTGGCATGGATTTTGAACTCTCACGC

ATTTCTTATCCTTTACTCATTCATTCTTTTAGTGATAATCAATTGAGCGAAATAGTTATT

AGAGAGCAACAATACGGCTCTAAAACCCAAGCCATG---CTGTATTTTTGCTTTTCTATT

TTGGAATTAAAAACCGCTACCCCCTTATTAAATAGAACGACTATGCTCAAAGAGCATGCC

CTGTTGATTATCCATAAAACCAACGCTCCCATGTTTTTAGAAATGCTTAAAATTTTTGGA

CTTTTAAGCCAAGCGCACCATAACGATGTGTTAAAGATTTTAGAAAAAATACTTCAAAAT

>HP15059

GTGAGTTTGATTAAAGTTAATGATGATAAAAAAGTGATTGAGGTTTCTATTCCTTTAACT

------------TCCATTTCAGGCAAAGTTCGTGTGAAAATTAGGCATGCCTTTAGCGAT

TATGGCATTTCAACAGCGACTAGAAAAATCCCTTTCAGTTTAAAACATTATGTAGAGTGG

CAAATCGGTTATGATGTCCCTATTAAAGATAAAGAA---AAATTTGAGCTCACTACTTTA

AAAGATGAAAAATATCATTTTTTAGGGGCTAATAATAAAGTAAAAACCCTTTATGAATTG

AGCGAAATAATTGATTACGCTAAGCAATTAGGTTTAATCAGT---------TTAGAAAAT

TTAGAAAATACTTTAAAATATTTAGAAGAACAAAAACAATTCATAGAAGATAGTTTTATG

ATTACAAGAGAAAGATTTAGATCGCATCAATTTGGTGGCATGGATTTTGAACTTTCACGC

ATTTCTTATCCTTTACTCATTCATTCTTTTAATGATAACCAATTGAGTGAAATCGTTATT

AGAGAGCAACAATATGGCTCTAAAACCCAAGCCATG---CTGTATTTTTGCTTTTCTATT

CTGGAATTAAAAACCGCTACCCCCTTATTAAATAGGACCGCTGCACTCAAAGAACATGCT

CTTTTAACTATCCATAAAACCAACGCTCCCATGTTTTTAGAAATGCTTAAAATTTTTGGA

CTTTTAAGCCAAGCACACCATAACGATGTGTTAAAGATTTTAGAAAAAATACTTGAAAAT

>HP07019

GTGAGTTTGATTAGGATTGATAATAATAAAAAAGTAATTGGGGTTTCTATTCCTTTAACT

------------TCAATTTCAGGCAAAGTGCGTGTGAAGATCGGACATGCCTTTAGTGAT

TATGGTATTTCAACAGCGACTAGAAAAATCCCTTTTAGTTTAAAACATTATATAGAGTGG

CAGATCGGTTATGATGTCCCCATTAAAGATAAAGAA---AAATTTGAACTCACTACTTTA

AAAGATAAAAAATACCATTTTTTAGGGGCTAATAATAAAGTAAAAACTCTTTATGAATTG

AGCGAAACGATTTATTACGCTAAGCAATTAGGTTTAATCAGT---------TTAGAAAAT

TTAGAAAATACTTTAAAATATTTAGAAAAACAAAAACAATTTATAGAAGATAATTTCACG

ATTACAAGAGAAAGATTTAGATCGCATCAATTTGGTGGCATGGATTTTGAACTCTCACGC

ATTTCTTATCCTTTGCTCATTCATTCTTTTAATGATAATCAGTTGAGCGAAATCGTTATT

AGAGAACAACAATATGGCTCTAAAACCCAAGCCATG---CTGTATTTTTGCTTTTCTATT

CTGGAATTAAAAACCGCTACCCCTTTATTAAATAGAACGGCTGCACTCAAAGAACATACT

CTTTTGATTATCCATAAAACCAACGCTCTTGTGTTTTTAGAAATGCTTAAAATTTTTGGA

CTTTTAAGCCAAGCGCACCATAGCGATGTGTTAAAGATTTTAGAAAAAATACTTCAAAAT

>MGms203

GTGAGTTTGATTAGGATTGATGATAGTAAAAAAGCGATTGAGATTTCCATTCCTTTAACT

------------TCAATTTCAGGCAAAGTGCGTGTGAAAATCAGACATGCCTTTAGCGAT

TATGGTATTTCAACAGCGACTAGAAAAATCCCTTTTAGTTTAAAACATTATATAGAGTGG

CAGATCGGTTATGATGTCCCCATTAAAGATAAAGAA---AAATTTGAGCTCACTACCCTA

AAAGATGAAAAATATCATTTTTTAGGGGCTAATAATAAAGTAAAGACTCTTTATGAATTG

AGCGAAATGATTTATTACGCTAAGCAATTAAGTTTAATCGGT---------TTAGAAAAT

TTAGAAAATACTTTAAAATATTTAGAAAAACAAAAACAATTTATAGAAGATAATTTTATG

ATTACAAGAGAAAGATTTAGATTACATCAATTTGGTGGCATGGATTTTGAACTCTCACGC

ATTTCTTATCCTTTGCTCATTCATTCTTTTAATGATAATCAGTTGAGCGAAATCGTTATT

AGAGAACAACAATACGGCTCTAAAACCCAAGCCATG---CTGTATTTTTGCTTTTCTATT

TTGGAGTTAAAAACTGCTACCCCCTTATTAAATAGAACGGCTGCACTCAAAGAACAGGCT

CTTTTAACCATCAATAAAACCAACGCTCTTATGTTTTTAGAAATGCTTAAAATTTTTGGA

CTTTTAAGCCAAGCGCACCATAGCGATGTGTTAAAGATTTTAGAAAAAATACTTCAAAAT

>ZH59

GTGAGTTTGATTAGGATTGATGATAGTAAAAAAGCGATTGAGGTTTCTATTCCTTTAACT

------------TCAATTTCAGGCAAAGTGCGTGTGAAAATCAGACATGCCTTTAGCGAT

TATGGTATTTCAACAGCGACTAGAAAAATCCCTTTTAGCTTAAAACATTATGTAGAGTGG

CAAATCGGTTATGATGTCCCTATTAAAGATAAAGAA---AAATTTGAGCTCACTACCCTA

AAAGATGAAAAATATCATTTTTTAGGGGCTAATAATAAAGTAAAGACTCTTTATGAATTG

AGTGAGATAATCTATTACGCTAAGCAATTAAATTTAATCAGT---------TTAGAAAAT

TTAGAAAATACTTTAAAATATTTAGAAAAACAAAAACAATTTATAGAAGATAATTTCACG

ATTACAAGAGAAAGATTTAGATTGCATCAATTTGGTGGCATGGATTTTGAACTTTCACGC

ATTTCTTATCCTTTACTCATTCATTCTTTCAACGATAATCAATTGAGCGAAATCGTTATT

AGAGAGCAACAATACGGCTCTAAAACCCAAGCCATG---CTGTATTTTTGCTTTTCTATT

CTGGAGTTAAAAACCGCTACTCCCTTATTAAATAGAACCGCTACACTCAAAGAACATGCT

TTTTTAATCATCCATAAAACCAACGCTCTCATGTTTTTAGAAATGCTTAAAATTTTTGGA

CTTTTAAGCCAAGCGCACCATAACGATGTGTTAAAGATTTTAGAAAAAATACTTCAAAAT

>ZH90

GTGAGTTTGATTAGGATTGATAATAATAAAAAAGTAATTGGGGTTTCTATTCCTTTAACT

------------TCAATTTCAGGCAAAGTGCGTGTGAAAATCAGACATGCCTTTAGCGAT

TATGGTATTTCAACAGCGACTAGAAAAATCCCTTTTAGCTTAAAACATTATGTAGAGTGG

CAAATCGGTTATGATGTCCCTATTAAAGATAAAGAA---AAATTTGAGCTCACTACCCTA

AAAGATGAAAAATATCATTTTTTAGGGGCTAATAATAAAGTGAAAACCCTTTATGAATTG

AGTGAGATAATCTATTACGCTAAGCAATTAAATTTAATCAGT---------TTAGAAAAT

TTAGAAAATACTTTAAAATATTTAGAAAAACAAAAACAATTTATAGAAGATAATTTTATA

------AGAGAAAGATTTAGATCGCATCAATTTGGTGGCATGGATTTTGAACTCTCACGC

ATTTCTTATCCCTTACTCATTCATTCTTTTAATGATAATCAGTTGAGCGAAATTGTTATT

AGAGAGCAACAATATGGTTCTAAAACCCAAGCCATG---CTGTATTTTTGCTTTTCTATT

TTGGAATTAAAAACCGCTACTCCCTTATTAAATAGAACGGCTGCACTCAAAGAACATGCC

CTTTTAACTATCCATAAAACCAACGCTCTTGTGTTTTTAGAAATGCTTAAAATTTTTGGA

CTTTTAAGCCAAGTGCACCATAACGATGTGTTAAAGATTTTAGAAAAAATACTTCAAAAT

>22393

GTGAGTTTGATTAGGATTGATAATAATAAAAAAGTAATTGAGATTTCTGTTCCTTTAACT

------------TCAATTTCAGGCAAAGTGCGTGTGAAAATCAGGCATGCCTTTAGCGAT

TATGGCATTTCAACAGCGACTAGAAAAATCCCTTTCAGTTTAAAGCATTATGTAGAGTGG

CAAATCGGTTATGATGTCCCCATTAAAGATAAAGAA---AAATTTGAACTCACTACCCTA

AAAGATGAAAAATATCATTTTTTAGGGGCTAATAATAAAGTAAAAACCCTTTATGAATTG

AGTGAGATAATCTATTACGCTAAGCAATTAAATTTAATCAGT---------TTAGAAAAT

TTAGAAAATACTTTAAAATATTTAGAAAAACAAAAACAATTTATAGAAGATAATTTT---

---ATAAGAGAAAGATTTAGATTACATCAATTTGGTGGCATGGATTTTGAACTCTCACGC

ATCTCTTATCCTTTACTCATTCATTCTTTTAATGATAATCAGTTGAGCGAAATCGTTATT

AGAGAGCAACAATACGGCTCTAAAACCCAAGCCATG---CTGTATTTTTGCTTTTCTATT

TTGGAATTAAAAACCGCTACTCCCTTATTAAATAGAACGGCTGCACTCAAAGAACATGCC

CTTTTAACTATCCATAAAACCAACGCTCTTATGTTTTTAGAAATGCTTAAAATTTTTGGA

CTTTTAAGCCAAGCGCACCATAGCGATGTGTTAAAGATTTTAGAAAAAATACTTCAAAAT

>66

GTGAGTTTGATTAAAGTTAGTGGTGATAAAAAAGTGATTGAGGTTTCTATTCCTTTAACT

------------TCAATTTCAGGCAAAGCGCGTGTGAAAATCAGACATGCCTTTAGCGAT

TATAGCATTTCAACAGCGACTAGAAAAATCCCTTTTAGTTTAAAACATTATGTAGAGTGG

CAGATCGGTTATGATGTCCCTATTAAAGATAAAGAA---AAATTTGAGCTCACTACTTTA

AAAGATGAAAAATATCATTTTTTAGGGGCTAATGATAAAGTAAAAACTCTTTATGAATTG

AGTGAAATGATTGATTACGCTAAGCAATTAGGTTTAATCAGT---------TTAGAAAAT

TTAGAAAATACTTTAAAATATTTAGAAAAACAAAAACAATTTATAGAAGATAATTTTATG

ATTACAAGAGAAAGATTTAGATCGCATCAATTTGGTGGCATGGATTTTGAACTCTCACGC

ATTTCTTATCCTTTGCTCATTCATTCTTTTGATGATAATCAGTTGAGCGAAATCGTTATT

AGAGAGCAACAATATGGTTCTAAAATCCAAGCCATG---CTGTATTTTTGCTTTTCTATT

TTGGAATTAAAAACCGCTACTCCCTTATTAAATAGAACCGCTACGCTCAAAGAGCATGCC

CTTTTAACCATCAATAAAACCAACGCTCTTGTGTTTTTAGAAATGCTTAAAATTTTTGGA

CTTTTAAGCCAAGCGCACCATAACGATGTGTTAAAGATTTTAGAAAAAATACTTCAAAAT

>MHP23

GTGAGTTTGATTAAGATTGATAATAATAAAAAAGCGATTGAGGTTTCTATTCCTTTAACT

------------TCCATTTCAGGCAAAGCGCGTGTGAAAATCAGACATGCCTTTAGCGAT

TATGGCATTTCAACAGCGACTAGAAAAATCCCTTTTAGTTTAAAGCATTATGTAGAGTGG

CAAATCGGTTATGATGTCCCCATTAAAGATAAAGAA---AAATTTGAACTCACTACCCTA

AAAGATGAAAAATATCATTTTTTAGGGGCTAATAATAAAGTAAAGACTCTTTATGAATTG

GGTGAAATGATTTATTACGCTAAGCGATTGGGTTTAATCAGT---------TTAGAAAAT

TTAGAAAATACTTTAAAATATTTAGAAAAACAAAAACAATTCATAGAAGATAATTTT---

---ATAAGAGAAAGATTTAGATCGCATCAATTTGGTGGCATGGATTTTGAACTCTCACGC

ATTTCTTATCCTTTACTCATTCATTCTTTTAATGATAACCAATTGAGTGAAATCGTTATT

AGAGAGCAACAATATGGCTCTAAAACCCAAGCCATG---CTGTATTTTTGCTTTTCTATT

CTGGAATTAAAAACCGCTACCCCCTTATTAAATAGAACGGCTGCACTCAAAGAACATGCC

CTTTTAACTATCCATAAAACCAACGCTCCCATGTTTTTAGAAATGCTTAAAATTTTTGGA

CTTTTAAGCCAAGCGCACCATAACGATGTGTTAAAGATTTTAGAAAAAATACTTCAAAAT

>B362

GTGAGTTTGATTAAAGTTAGTGGTGATAAAAAAGCGATTGAGGTTTCCATTCCTTTAACT

------------TCAATTTCAGGCAAAGCGCGTGTGAAAATCAGACATGCCTTTAGCGAT

TATGGTATTTCAACAGCGACTAGAAAAATCCCTTTTAGTTTAAAACATTATATAGAGTGG

CAGATCGGTTATGATGTCCCCATTAAAGATAAAGAA---AAATTTGAACTCACTACTTTA

AAAGATGAAAAATATCATTTTTTAGGGGCTAATAATAAAGTGAAAACTCTTTATGAATTG

AGCGAAATGATTTATTACACTAAGCAATTAGGTTTAATCAGT---------TTAGAAAAT

TTAGAAAATACTTTAAAATATTTAGAAAAACAAAAACAATTTATAGAAGATAATTTTATG

ATTACAAGAGAAAGATTTAGATCGCATCAATTTGGTGGCGTGGATTTTGAACTTTCACGC

ATCTCTTATCCTTTACTCATTCATTCTTTTAATGATAATCAGTTGAGCGAAATCGTTATT

AGAGAGCAACAATATGGCTCTAAAACCCAAGCCATG---CTGTATTTTTGCTTTTCTATT

TTGGAATTAAAAACCGCTACTCCCTTATTAAATAGAACCGCTACACTCAAAGAACATGCC

CTTTTAACTATCCATAAAACCAACGCTCTTGTGTTTTTAGAAATGCTTAAAATTTTTGGA

CTTTTAAGCCAAGCGTACCATAACGATGTGTTAAAGATTTTAGAAAAAATACTTCAAAAT

>LIM-005

GTGAGTTTGATTAGGATTGATGATAGTAAAAAAGCGATTGAGGTTTCTGTTCCTTTAACT

------------TCCATTTCAGGCAAAGCGCGTGTGAAAATCAGACATGCCTTTAGCGAT

TATGGCATTTCAACAGCGACTAGAAAAATCCCTTTTAGTTTAAAACATTATGTAGAGTGG

CAAATCGGTTATGATGTCCCCATTAAAGATAAAGAA---AAATTTGAACTCACTACCCTA

AAAGATGAAAAATATCATTTTTTAGGGGCTAATAATAAAATAAAAACCCTTTATGAATTG

AGCGAAATAATTTATTACGCTAAGCGATTGGGTTTAATCAGT---------TTAGAAAAT

TTAGAAAATACTTTAAAATATTTAGAAAAACAAAAACAATTCATAGAAGATAGTTTTATG

ATCACAAGAGAAAGATTTAGATCGCATCAATTTGGGGGCATGGATTTTGAACTTTCACGC

ATTTCTTATCCCTTACTCATTCATTCTTTTAATGATAATCAATTGAGCGAAATCGTTATT

AGAGAGCAACAATACGGCTCTAAAACCCAAGCCATG---CTGTATTTTTGCTTTTCTATT

TTGGAGTTAAAAACCGCTACCCCTTTATTAAATAGAACCGCTGCACTCAAAGAACATGCT

TTTTTAATTATCCATAAAACTAACGCTCTTGTGTTTTTAGAAATGCTTAAAATTTTTGGA

CTTTTAAGCCAAGTGCACCATAACGATGTGTTAAAGATTTTAGAAAAAATACTTCAAAAT

>KH44

GTGAGTTTGATTAAAGTTGACTATGATAAAAAAGTGATTGATGTTTCTATTCCTTTAACT

------------TCAATTTCAGGCAAAGTGCGTGTGAAAATCAGACATGCCTTTAGTGAT

TATGGTATTTCAACAGCGACTAGAAAAATCCCTTTTAGTTTAAAGCATTATGTAGAGTGG

CAAATCGGTTATGATGTCCCCATTAAAGATAAAGAA---AAATTTAAACTCACTACTTTA

AAAGATGAAAAATATCATTTTTTAGGGGCTAATAATAAAGTAAAGACTCTTTATGAATTG

AGTGAGATAATCTATTACGCTAAGCAATTAAATTTAATCAGT---------TTAGAAAAT

TTAGAAAATACTTTAAAATATTTAGAAAAACAAAAACAATTTATAGAAAATAATTTTATG

ATTACAAGAGAAAGATTTAGATTACATCAATTTGGTGGCATGGATTTTGAACTCTCACGC

ATTTCTTATCCCTTACTCATTCATTCTTTCAACGATAATCAGTTGAGCGAAATCGTTATT

AGAGAGCAACAATACGGCTCTAAAACCCAAGCCATG---CTGTATTTTTGCTTTTCTATT

TTGGAGTTAAAAACCGCTACCCCCTTATTAAATAGAACGGCTGCACTCAAAGAACATGCC

CTTTTAACTATCCATAAAACCAACGCTCTCATGTTTTTAGAAATGCTTAAAATTTTTGGA

CTTTTAAGCCAAGCGCACCATAGCGATGTGTTAAAGATTTTAGAAAAAATACTTCAAAAT

>ZH55

GTGAGTTTGATTAAAGTTAATGATGATAAAAAAGCGATTGAGGTTTCTATTCCTTTAACT

------------TCAATTTCAGGCAAAGTGCGTGTGAAAATCAGACATGCCTTTAGCGAT

TATGGCATTTCAACAGCGACCAGAAAAATCCCTTTCAGTTTAAAGCATTATGTAGAGTGG

CAAATCGGTTATGATGTCCCCATTAAAGATAAAGAA---AAATTTGAACTCACTACCCTA

AAAGATGAAAAATATCATTTTTTAGGGGCTAATAATAAAGTAAAAACTCTTTATGAATTG

AGCGAAATGATTTATTACGCTAAGCGATTGGGTTTAATCAGT---------TTAGAAAAT

TTAGAAAATACTTTAAAATATTTAGAAAAACAAAAACAATTCATAGAAGATAGTTTTATG

ATCACAAGAGAAAGATTTAGATCGCATCAATTTGGGGGCATGGATTTTGAACTTTCACGC

ATCTCTTATCCCTTACTCATTCATTCTTTTAATGATAATCAGTTGAGTGAAATTGTTATT

AGAGAACAACAATATGGCTCTAAAACCCAAGCCATG---CTGTATTTTTGCTTTTCTATT

TTGGAATTAAAAACCGCTACCCCCTTATTAAACAGAACGGCTATGCTCAAAGAACATGCT

TTTTTAACCATCCATAAAACCAACGCTCCCATGTTTTTAGAAATGCTTAAAATTTTTGGA

CTTTTAAGCCAAGCGCACCATGACGATGTGTTAAAGATTCTAGAAAAAATACTTCAAAAT

>HP05044

GTGAGTTTGATTAGGATTGATGATGGTAAAAAAGCGATTGAGGTTTCTATTCCTTTAACT

------------TCAATTTCAGGCAAAGCGCGTGTGAAAATCAGACATGCCTTTAGCGAT

TATGGTATTTCAACAGCGACTAGAAAAATCCCTTTTAGCTTAAAACATTATGTAGAGTGG

CAAATCGGTTATGATGTCCCCATTAAAGATAAAGAA---AAATTTGAACTCACTACTTTA

AAAGATGAAAAATATCATTTTTTAGGGGCTAATAATAAAGTAAAAACTCTTTATGAATTG

AGTGAAATGATTGATTACGCTAAGCACTTAGGTTTAATCAGT---------TTAGAAAAT

TTAGAAAATACTTTAAAATATTTAGAAAAACAAAAACAATTTATAGAAGATAATTTTATG

ACTACAAGAAAAAGATTTAGATCGCATCAATTTGGTGGCATGGATTTTGAACTCTCACGC

ATTTCTTATCCTTTACTCATTCATTCTTTTAATGATAATCAGTTGAGCGAAATCGTTATT

AGAGAACAACAATATGGCTCTAAAACCCAAGCCATG---CTGTATTTTTGCTTTTCTATT

CTGGAATTAAAAACCGCTACCCCTTTATTAAATAGAACGGCTGCACTCAAAGAACATGCT

CTTTTGATTATCCATAAAACCAACGCTCTCATGTTTTTAGAAATGCTTAAAATTTTTGGA

CTTTTAAGCCAAGCGCACCATAGCGATGTGTTAAAGATTTTAGAAAAAATACTTCAAAAT

>MHP01

GTGAGTTTGATTAGGATTGATAATAATAAAAAAGTGATTGAGGTTTCCATTCCTTTAACT

------------TCCATTTCAGGCAAAGTTCGTGTGAAAATCAGGCATGCCTTTAGCGAT

TATGGCATTTCAACAGCGACCAGAAAAATCCCTTTTAGTTTAAAACATTATGTAGAGTGG

CAAATCGGTTATGATGTCCCCATTAAAGATAAAGAA---AAATTTGAACTCACTACCCTA

AAAGATGAAAAATATCATTTTTTAGGGGCTAATAATAAAGTAAAAACCCTTTATGAATTG

AGTGAGATAATCTATTACGCTAAGCAATTAAATTTAATCAGT---------TTAGAAAAT

TTAGAAAATACTTTAAAATATTTAGAAAAACAAAAACAATTTATAGAAGATAATTTTATA

------AGAGAAAGATTTAGATCGCATCAATTTGGTGGCATGGATTTTGAACTTTCACGC

ATTTCTTATCCTTTACTCATTCATTTTTTCAATGATAATCAGTTGAGCGAAATCGTTATT

AGAGAGCAACAATACGGCTCTAAAACCCAAGCCATG---CTATATTTTTGCTTTTCTATT

CTGGAATTAAAAACCGCTACCCCCTTATTAAATAGAACGGCTGCCCTCAAAGAACATGCC

CTTTTAACTATCCATAAAGCCAACGCTCTTGTGTTTTTAGAAATGCTTAAAATTTTTGGA

CTTTTAAGCCAAGCGCACCATAACGATGTGTTAAAGATTTTAGAAAAAATACTTCAAAAT

>MHP38

GTGAGTTTGATTAAAGTTAATGATGATAAAAAAGCGATTGAGGTTTCTATTCCTTTAACT

------------TCCATTTCAGGCAAAGTGCGTGTGAAAATCAGACATGCCTTTAGCGAT

TATGGTATTTCAACAGCGACTAGAAAAATCCCTTTTAGCTTAAAACATTATGTAGAGTGG

CAAATCGGTTATGATGTCCCCATTAAAGATAAAGAA---AAATTTGAGCTCACTACTTTA

AAAGATGAAAAATATCATTTTTTAGGGGCTAATAATAAAGTAAAAACTCTTTATGAATTG

AGTGAAATAATCTATTACGCTAAGCAATTAAATTTAATCAGT---------TTAGAAAAT

TTAGAAAATACTTTAAAATATTTAGAAAAACAAAAACAATTTATAGAAGATAATTTCACG

ATTACAAGAGAAAGATTTAGATCGCATCAATTTGGTGGCATGGATTTTGAACTTTCACGC

ATTTCTTATCCTTTACTCATTCATTCTTTTAATGATAATCAGTTGAGTGAAATCGTTATT

AGAGAGCAACAATATGGCTCTAAAACCCAAGCCATG---CTGTATTTTTGCTTTTCTATT

TTGGAGTTAAAAACCGCTACCCCCTTATTAAACAGAACGGCTATGCTCAAAGAGCATGCC

CTTTTAACTATCCATAAAACTAACGCTCTTGTGTTTTTAGAAATGCTTAAAATTTTTGGA

CTTTTAAGCCAAGTGCACCATAACGATGTGTTAAAGATTTTAGAAAAAATACTTCAAAAT

>B130A

GTGAGTTTGATTAAAGTTAGTGGTGATAAAAAAGTGATTGAAGTTTCTATTCCTTTAACT

------------TCAATTTCAGGCAAAGTGCGTGTGAAAATCAGACATGCCTTTAGCGAT

TATGGTATTTCAACAGCGACTAGAAAAATCCCTTTTAGTTTAAAACATTATATAGAGTGG

CAGATCGGTTATGATGTCCCCATTAAAGATAAAGAA---AAATTTGAACTCACTACTTTA

AAAGATGAAAAATATCATTTTTTAGGGGCTAATAATAAAGTAAAGACTCTTTATGAATTG

AGTGAGATAATCTATTACGCTAAGCAATTAAATTTAATCAGT---------TTAGAAAAT

TTAGAAAATACTTTAAAATATTTAGAAAAACAAAAACAATTTATAGAAGATAGTTTCACG

ATTACAAGAGAAAGATTTAGATCGCATCAATTTGGTGGCATGGATTTTGAACTTTCACGC

ATTTCTTACCCTTTACTCATTCATTCTTTTAATGATAATCAGTTGAGCGAAATCGTTATT

AGAGAGCAACAATACGGCTCTAAAACCCAAGCCATG---CTGTATTTTTGCTTTTCTATT

TTGGAATTAAAAACCGCTACTCCCTTATTAAATAGAACGGCTGCACTCAAAGAACATGCT

CTTTTGATTATCCATAAAACCAACGCTCTTATGTTTTTAGAAATGCTTAAAATTTTTGGA

CTTTTAAGTCAAGTGCACCATAACGATGTGTTAAAGATTTTAGAAAAAATACTTCAAAAT

>MHP17

GTGAGTTTGATTAAAGTTAATGATGATAAAAAAGCGATTGAGGTTTCTATTCCTTTAACT

------------TCCACTTCAGGCAAAGTGCGTGTGAAAATCAGACATGCCTTTAGCGAT

TATGGCATTTCAACAGCGACTAGAAAAATCCCTTTTAGTTTAAAGCATTATGTAGAGTGG

CAAATCGGTTATGATGTCCCCATTAAAGATAAAGAA---AAATTTGAACTCACTACCCTA

AAAGATGAAAAATATCATTTTTTAGGGGCTAATAATAAAGTAAAAACCCTTTATGAATTG

AGCGAAATAATTGATTACGCTAAGCGATTGGGTTTAATCAGT---------TTAGAAAAT

TTAGAAAATACTTTAAAATATTTAGAAAAACAAAAACAATTCATAGAAGATAGTTTTATG

ATCACAAGAGAAAGATTTAGATCGCATCAATTTGGGGGCATGGATTTTGAACTTTCACGC

ATTTCTTATCCTTTACTCATTCATTCTTTTAATGATAACCAATTGAGTGAAATCGTTATT

AGAGAGCAACAATATGGCTCTAAAACCCAAGCCATG---CTGTATTTTTGCTTTTCTATT

CTGGAATTAAAAACCGCTACACCCTTATTAAATAGAACGGCTGCACTCAAAGAACATGCC

CTTTTAACTATCCATAAAACCAACGCTCTTGTGTTTTTAGAAATGCTTAAAATTTTTGGA

CTTTTAAGCCAAGCGCACCATAACGATGTGTTAAAGATTTTAGAAAAAATACTTCAAAAT

>GC31-B

GTGAGTTTGATTAAAATTAACCATGATGAAAAAGTGATTGAGGTTTCTATTCCTTTAACT

------------TCAAATTCAGGCAAAGTGCGTGTGAAAATCAGACATGCCTTTAGCGAT

TATGGTATTTCAACAGCGACTAGAAAAATCCCTTTTAGTTTAAAACATTATGTAGAGTGG

CAGATCGGTTATGATGTCCCCATTAAAGATAAAGAA---AAATTTGAACTCACTACTTTA

AAAGATGAAAAATATCATTTTTTAGGGGCTAATAATAAAGTAAAAACTCTTTATGAATTG

AGCGAAATGATTTATTACGCTAAGCAATTAGGTTTAATCAGT---------TTAGAAAAT

TTAGAAAATATTTTAAAATATTTAGAAAAACAAAAACAATTTATAGAAGATAATTTTATG

ATTACAAGAGAAAGATTTAGATCGCATCAATTTGGTGGCATGGATTTTGAACTCTCACGC

ATTTCTTATCCTTTGCTCATTCATTCTTTTAATGATAATGAGTTGAGCGAAATCGTTATT

AGAGAGCAACAATATGGCTCTAAAACCCAGGCCATG---CTGTATTTTTGCTTTTCTATT

TTGGAATTAAAAACCGCTACTCCCTTATTAAATAGAACGGCTGCACTCAAAGAACATGCC

CTTTTAACTATCCATAAAACCAACGCTCCCATGTTTTTAGAAATGCTTAAAATTTTTGGA

CTTTTAAGTCAAGCACACCATGATGATGTGTTAGAGATTTTAGAAAAAATACTTCAAAAT

>G-Mx-2005-108

GTGAGTTTGATTAAGATTGATAATGATAAAAAAGCGATTGAGGTTTCTATTCCTTTAACT

------------TCCATTTCAGGCAAAGCACGTGTGAAAATCAGACATGCCTTTAGCGAT

TATGGCATTTCAACAGCGACTAGAAAAATCCCTTTTAGTTTAAAGCATTATGTAGAGTGG

CAAATCGGTTATGATGTCCCTATTAAAGATAAAGAA---AAATTTGAGCTCACTACTTTA

AAAGATGAAAAATATCATTTTTTAGGGGCTAATAATAAAGTAAAAACTCTTTATGAATTA

AGCGAAATAATTGATTACGCTAAGCGATTGGGTTTAATCGGT---------TTAGAAAAT

TTAGAAAATACTTTAAAATATTTAGAAAAACAAAAACAATTCATAGAAGATAATTTTACG

ATTACAAGAGAAAGATTTAGATCGCATCAATTTGGTGGCATGGATTTTGAACTTTCACGC

ATTTCTTACCCTTTACTCATTCATTCTTTCAATGATAATCAGTTGAGCGAAATCGTTATT

AGAGAGCAACAATACGGCTCTAAAACCCAAGCCATG---CTGTATTTTTGCTTTTCTATT

TTGGAATTAAAAACCACTACCCCCTTATTAAATAGAACGGCTGCACTCAAAGAACATGCC

CTTTTAACTATCCATAAAACCAACGCTCTTATGTTTTTAGAAATGCTTAAAATTTTTGGA

CTTTTAAGCCAAGCACACCATAGCGATGTGTTAAAGATTTTAGAAAAAATACTTCAAAAT

>44A4

GTGAGTTTGATTAAAGTTGACTATGATAAAAAAGTGATTGAGGTCTCTATTCCTTTAACT

------------TCAATTTCAGGCAAAGTGCGTGTGAAGATCAGACATGCCTTTAGCGAT

TATGGTATTTCAACAGCGACTAGAAAAATCCCTTTTAGTTTAAAACATTATGTAGAGTGG

CAAATCGGTTATGATGTCCCCATTAAAGATAAAGAA---AAATTTGAACTCACTGCTTTA

AAAGATAAAAAATACCATTTTTTAGGGGCTAATAATAAAGTAAAAACTCTTTATGAATTG

AGCGAAATGATTTATTACGCTAAGCAATTAGGTTTAATCAGT---------TTAGAAAAT

TTAGAAAATACTTTAAAATATTTAGAAAAACAAAAACAATTTATAGAAGATAATTTTATG

ATTACAAGAGAAAAATTCAGATCGCATCAATTTGGTGGCATGGATTTTGAACTCTCACGC

ATTTCTTATCCTTTACTCATTCATTCTTTTAACGATAATCAGTTGAGCGAAATTGTTATT

AGAGAACAACAATATGGTTCTAAAACCCAAGCCATG---CTGTATTTTTGCTTTTCTATT

TTGGAGTTAAAAACCGCTACCCCCTTATTAAACAGAACGGCTATGCTCAAAGAGTATGCT

CTTTTGATTATCCATAAAACCAACGCTCCCATGTTTTTAGAAATGCTTAAAATTTTTGGG

CTTTTAAGCCAAGCGCATCATAACGATGTGTTAAAGATTTTAGAAAAAATACTTCAAAAT

>HP06038

GTGAGTTTGATTAGGATTGATAATAATAAAAAAGTAATTGGGGTTTCTATTCCTTTAACT

------------TCAATTTCAGGCAAAGTGCGTGTGAAAATCAGACATGCCTTTAGCGAT

TATGGTATTTCAACAGCGACTAGAAAAATCCCTTTTAGCTTAAAACATTATGTAGAGTGG

CAAATCGGTTATGATGTCCCCATTAAAGATAAAGAA---AAATTTGAGCTCACTACCCTA

AAAGATGAAAAATATCATTTTTTAGGGGCTAATAATAAAGTAAAAACCCTTTATGAATTG

AGCGAAATGATTTATTACGCTAAGCAATTAAATTTAATCAGT---------TTAGAAAAT

TTAGAAAATACTTTAAAATATTTAGAAAAACAAAAACAATTTATAGAAGATAATTTCACG

ATTACAAGAGAAAGATTTAGATCGCATCAATTTGGTGGCATGGATTTTGAACTCTCACAC

ATTTCTTATCCTTTGCTCATTCATTCTTTTAATGATAATCAGTTGAGTGAAATAGTTATT

AGAGAACAACAATATGGCTCTAAAACCCAAGCCATG---CTCTATTTTTGCTTTTCTATT

TTGGAATTAAAAACCGCTACCCCCTTATTAAACAGAACGGCTATGCTCAAAGAGCATGCT

CTTTTGATTATCCATAAAACCAACGCTCCCATGTTTTTAGAAATGCTTAAAATTTTTGGA

CTTTTAAGCCAAGTGTACCATAACGATGTGTTAAAGATTTTAGAAAAAATACTTCAAAAT

>MG2003-98

GTGAGTTTGATTAAAGTTAATGATGATAAAAAAGTGATTGAGGTTTCTATTCCTTTAACT

------------TCCATTTCAGGCAAAGTTCGTGTGAAAATTAGGCATGCCTTTAGTGAT

TATGGTATTTCAACAGCGACTAGAAAAATCCCTTTCAGTTTAAAGCATTATGTAGAGTGG

CAAATCGGTTATGATGTCCCCATTAAAGATAAAGAA---AAATTTGAACTCACTACTTTA

AAAGATGAAAAATATCATTTTTTAGGGGCTAATAATAGAGTAAAGACTCTTTATGAATTG

AGCGAAATAATTGATTACGCTAAGCGATTGGGTTTAATCAGT---------TTAGAAAAT

TTAGAAAATACTTTAAAATATTTAGAAAAACAAAAACAATTCATAGAAGATAGTTTTATA

ATTACAAGAGAAAGATTTAGATCGCATCAATTTGGTGGCATGGATTTTGAACTTTCACGC

ATCTCTTATCCTTTACTCATTCATTCTTTTAATGATAATCAGTTGAGCGAAATCGTTATT

AGAGAGCAACAATACGGCTCTAAAACCCAAGCTATG---CTGTATTTTTGCTTTTCTATT

CTGGAATTAAAAACCGCTACCCCTTTATTAAATAGGACCGCTGCCCTCAAAGAACATGCC

CTTTTAACTATCCACAAAACTAACGCTCTTGTGTTTTTAGAAATGCTTAAAATTTTTGGA

CTTTTAAGCCAAGCGCACCATAACGATGTGTTAAAGATTTTAGAAAAAATACTTCAAAAT

>Nic46-A

GTGAGTTTGATTAAGATTGATAATAATAAAAAAGCGATTGAGGTTTCTATTCCTTTAACT

------------TCAATTTCAGGCAAAGCGCGTGTGAAAATCAGACATGCTTTTAGCGAT

TATGGTATTTCAACAGCGACTAGAAAAATCCCTTTTAGTTTAAAACATTATGTAGAGTGG

CAGATCGGTTACGATGTCCCCATTAAAGATAAAGAA---AAATTGGAGCTCACTACCCTA

AAAGATGAAAAATATCATTTTTTAGGGGCTAATAATAAAGTAAAAACCCTTTATGAATTG

AGTGAGATAATCTATTACGCTAAGCAATTAAATTTAATCAGT---------TTAGAAAAT

TTAGAAAATACTTTAAAATATTTAGAAAAACAAAAACAATTTATAGAAGATAATTTCACG

ATTACAAGAGAAAGATTTAGATCGCATCAATTTGGTGGCATGGATTTTGAACTCTCACGC

ATTTCTTATCCTTTGCTCATTCATTCTTTTAATGATAATGAGTTGAGCGAAATCGTTATT

AGAGAACAACAATATGGCTCTAAAACCCAAGCCATG---CTGTATTTTTGCTTTTCTATT

TTGGAGTTAAAAACCGCTACCCCTTTATTAAATAGAACCGCTACACTCAAAGAACATGCT

TTTTTAACTATCCATAAAACCAACGCTCTTGTGTTTTTAGAAATGCTTAAAATTTTTGGA

CTTTTAAGCCAAGTGCACCATAACGATGTGTTAAAGATTTTAGAAAAAATACTTCAAAAT

>HP99216

GTGAGTTTGATTAAAGTTAATGATGATAAAAAAGTGATTGAGGTTTCTATTCCTTTAACT

------------TCCATTTCAGGCAAAGTTCGTGTGAAAATCAGACATGCCTTTAGCGAT

TATGGCATTTCAACAGCGACTAGAAAAATCCCTTTCAGTTTAAAGCATTATGTAGAGTGG

CAAATCGGTTATGATGTCCCCATTAAAGATAAAGAA---AAATTTGAACTCACTACCCTA

AAAGATGAAAAATATCATTTTTTAGGGGCTAATAATAAAATAAAAACCCTTTATGAATTG

AGCGAAATAATTGATTACGCTAAGCGATTGGATTTAATCAGT---------TTAGAAAAT

TTAGAAAATACTTTAAAATATTTAGAAAAACAAAAACAATTCATAGAAGATAGTTTTATG

ATTACAAGAGAAAGATTTAGATCGCATCAATTTGGTGGCATGGATTTTGAACTTTCACGC

ATTTCTTATCCTTTACTCATTCATTCTTTCAACGATAATCAGTTGAGCGAAATCGTTATT

AGAGAGCAACAATACGGCTCTAAAACCCAAGCCATG---CTATATTTTTGCTTTTCTATT

CTGGAATTAAAAACCGCTACCCCCTTATTAAATAGAACCGCTGCCCTCAAAGAACATGCC

CTTTTAACTATCCATAAAACCAACGCTCTTATGTTTTTAGAAATGCTTAAAATTTTTGGC

CTTTTAAGCCAAGCGCACCATAACGATGTGTTAAAGATTTTAGAAAAAATACTTGAAAAT

>Nic42-A

GTGAGTTTGATTAAAGTTAATGATGATAAAAAAGCGATTGAGGTTTCTATTCCTTTAACT

------------TCCATTTCAGGCAAAGCACATGTGAAAATCAGGCATGCCTTTAGCGAT

TATGGTATTTCAACAGCGACTAGAAAAATCCCTTTTAGTTTAAAGCATTATGTAGAGTGG

CAAATCGGTTATGATGTCCCTATTAAAGATAAAGAA---AAATTTGAACTCACTACTTTA

AAAGATGAAAAATATCATTTTTTAGGGGCTAATAATCAAGTAAAAACCCTTTATGAATTG

AGCGAAATAATTGATTACGCTAAGCGATTGGGTTTAATCAGT---------TTAGAAAAT

TTAGAAAATACTTTAAAATATTTAGAAAAACAAAAACAATTCATAGAAGATAATTTTATG

ATTACAAGAGAAAGATTTAGATCGCATCAATTTGGTGGCATGGATTTTGAACTTTCACGC

ATTTCTTATCCTTTACTCATTCATTCTTTTAATGATAATCAATTGAGTGAAATCGTTATT

AGAGAGCAACAATACGGCTCTAAAACCCAAGCCATG---CTGTATTTTTGCTTTTCTATT

TTGGAATTAAAAACCGCTACCCCCTTATTAAATAGAACGGCTGCTCTCAAAGAACATGCC

CTTTTAACTATCCATAAAACCAACGCTCTTATGTTTTTAGAAATGCTTAAAATTTTTGGA

CTTTTAAGCCAAGCGCACCATAACGATGTGTTAAAGATTTTAGAAAAAATACTTCAAAAT

>MHP33

GTGAGTTTGATTAGGATTGATGATAGTAAAAAAGCGATTGAGGTTTCTATTCCTTTAACT

------------TCCATTTCAGGCAAAGTTCGTGTGAAAATTAGGCATGCCTTTAGCGAT

TATGGTATTTCAACAGCGACTAGAAAAATCCCTTTTAGTTTAAAGCATTATGTAGAGTGG

CAAATCGGTTATGATGTCCCCATTAAAGATAAAGAA---AAATTTGAACTCACTACCCTA

AAAGATGAAAAATATCATTTTTTAGGGGCTAATAATAAAGTAAAAACCCTTTATGAATTG

AGCGAAATAATTGATTACGCTAAGCGATTGGGTTTAATCAGT---------TTAGAAAAT

TTAGAAAATACTTTAAAATATTTAGAAAAACAAAAACAATTCATAGAAGATAATTTTATG

ATCACAAGAGAAAGATTTAGATCGCATCAATTTGGTGGCATGGATTTTGAACTTTCACGC

ATTTCTTATCCTTTACTCATTCATTCTTTTGATGATAATCAGTTGAGCGAAATCGTTATT

GGAGAGCAACAATATGGCTCTAAAACCCAAGCCATG---CTGTATTTTTGCTTTTCTATT

TTGGAATTAAAAACCGCTACTCCCTTATTAAACAGAACGGCTACGCTCAAAGAACATGCT

CTTTTGATTATCCATAAAACCAACGCTCTTGTGTTTTTAGAAATGCTTAAAATTTTTGGA

CTTTTAAGCCAAGTGCACCATAACGATGTGTTAAAGATTTTAGAAAAAATACTTCAAAAT

>KH0149

GTGAGTTTGATTAAAATTAACCATGATAAAAAAGTGATTGAGGTTTCTATTCCTTTAACT

------------TCAATTTCAGGCAAAGTGCGTGTGAAAATCAGACATGCCTTTAGTGAT

TATGGTATTTCAACAGCGACTAGAACAATCCCTTTTAGTTTAAAACATTATGTAGAGTGG

CAGATCGGTTATGATGTCCCCATTAAAGATAAAGAA---AAATTTGAACTCACTACTTTA

AGAGATGAAAAATATCATTTTTTAGGGGCTAATAATAAAGTAAAAACTCTTTATGAATTG

AGCGAAATGATTTATTACGCCAAGCAATTAGGTTTAATCAGT---------TTAGAAAAT

TTAGAAAATACTTTAAAATATTTAGAAAAACAAAAACAATTTATAGAAGATAATTTTATG

ATTACAAGAGAAAGATTTAGATCGCATCAATTTGGTGGCATGGATTTTGAACTTTCACGC

ATTTCTTATCCTTTACTCATTCATTCTTTTAATGATAATCAATTGAGCGAAATTGTTATT

AGAGAACAACAATATGGTTCTAAAACCCAAGCTATG---CTGTATTTTTGCTTTTCTATT

TTGGAATTAAAAACCGCTCCTCCCTTATTAAACAGAACGGCTACGCTCAAAGAACATGCT

CTTTTGATTATCCATAAAACCAACGCTCCCATGTTTTTAGAAATGCTTAAAATTTTTGGA

CTTTTAAGCCAAGCGCACCATGACGATGTGTTAAAGATTTTAGAAAAAATACTTCAAAAT

>2017-79

GTGAGTTTGATTAAAGTTAATGATGATAAAAAAGCGATTGAGGTTTCTATTCCTTTAACT

------------TCCATTTCAGGCAAAACGCGTGTGAAAATCAGACACGCCTTTAGCGAT

TATGGTATTTCAACAGCGACTAGAAAAATCCCTTTCAGTTTAAAGCATTATGTAGAGTGG

CAAATCGGTTATGATGTCCCCATTAAAGATAAAGAA---AAATTTGAACTCACTACCCTA

AAAGATGAAAAATATCATTTTTTAGGGGCTAATAATAGAGTAAAGACTCTTTATGAATTG

AGCGAAATAATTTATTACGCTAAGCGATTGGGTTTAATCAGT---------TTAGAAAAT

TTAGAAAATACTTTAAAATATTTAGAAAAACAAAAACAATTCATAGAAGATAATTTTATG

ATTACAAGAGAAAGATTTAGATCGCATCAATTTGGTGGCATGGATTTTGAACTTTCACGC

ATCTCTTATCCTTTACTCATTCATTCTTTTAATGATAACCAATTGAGCGAAATCGTTATT

AGAGAGCAACAATACGGCTCTAAAACCCAAGCCATG---CTGTATTTTTGCTTTTCTATT

TTGGAGTTAAAAACCGCTACCCCCTTATTAAATAGGACCGCTGCCCTCAAAGAACATGCT

CTTTTAACTATCCATGAAACTAACGCTCTTGTGTTTTTAGAAATGCTTAAAATTTTTGGC

CTCTTAAGCCAAGCACACCATAACGATGTGTTAAAGATTTTAAAAAAAATACTTGAAAAT

>2017-177

GTGAGTTTGATTAAAGTTAATGATGATAAAAAAGCGATTGAGGTTTCTATTCCTTTAACT

------------TCCATTTCAGGCAAAACGCGTGTGAAAATCAGACACGCCTTTAGCGAT

TATGGTATTTCAACAGCGACTAGAAAAATCCCTTTCAGTTTAAAGCATTATGTAGAGTGG

CAAATCGGTTATGATGTCCCCATTAAAGATAAAGAA---AAATTTGAACTCACTACCCTA

AAAGATGAAAAATATCATTTTTTAGGGGCTAATAATAGAGTAAAGACTCTTTATGAATTG

AGCGAAATAATTTATTACGCTAAGCGATTGGGTTTAATCAGT---------TTAGAAAAT

TTAGAAAATACTTTAAAATATTTAGAAAAACAAAAACAATTCATAGAAGATAATTTTATG

ATTACAAGAGAAAGATTTAGATCGCATCAATTTGGTGGCATGGATTTTGAACTTTCACGC

ATCTCTTATCCTTTACTCATTCATTCTTTTAATGATAACCAATTGAGCGAAATCGTTATT

AGAGAGCAACAATACGGCTCTAAAACCCAAGCCATG---CTGTATTTTTGCTTTTCTATT

TTGGAGTTAAAAACCGCTACCCCCTTATTAAATAGGACCGCTGCCCTCAAAGAACATGCT

CTTTTAACTATCCATGAAACTAACGCTCTTGTGTTTTTAGAAATGCTTAAAATTTTTGGC

CTCTTAAGCCAAGCACACCATAACGATGTGTTAAAGATTTTAAAAAAAATACTTGAAAAT

>3824

GTGAGTTTGATTAAAGTTAATGATGATAAAAAAGCGATTGAGGTTTCTATTCCTTTAACT

------------TCCATTTCAGGTAAAGCGCGTGTGAAAATCAGGCATGCCTTTAGCGAT

TATGGCATTTCAACAGCGACCAGAAAAATTCCTTTTAGTTTAAAGCATTATGTAGAGTGG

CAAATCGGCTATGATGTCCCCATTAAAGATAAAGAA---AAATTTGAACTCACTACCCTA

AAAGATGAAAAATATCATTTTTTAGGGGCTAACAATAAAGTAAAGACTCTTTATGAATTG

AGCGAAATGATTTATTACGCTAAGCGATTGGGTTTAATCAGT---------TTAGAAAAT

TTAGAAAATACTTTAAAATATTTAGAAAAACAAAAACAATTTATAGAAGATAATTTTATG

ATTACAAGAGAAAGATTTAGATCGCATCAATTTGGTGGCATGGATTTTGAACTTTCACGC

ATTTCTTATCCCTTACTCATTCATTCTTTTAATGATAATCAATTGAGTGAAATCGTTATT

AGAGAGCAACAATACGGCTCTAAAGTCCAAGCCATG---CTGTATTTTTGCTTTTCTATT

TTGGAGTTAAAAACCGCTACCCCCTTATTAAATAGAACCGCTGCACTCAAAGAACATGCT

CTTTTAATTATCCATAAAACCAACGCTCCCATGTTTTTAGAAATGCTTAAAATTTTTGGA

CTTTTAAGCCAAGTGCACCATAACGATGTGTTAAAGATTTTAGAAAAAATACTTGAAAAT

>HP15044

GTGAGTTTGATTAAAGTCAGTGATGATAAAAAAGCGATTGAGGTTTCCATTCCTTTAACT

------------TCAATTTCAGGCAAAGTGCGTGTGAAAATCAGACATGCCTTTAGCGAT

TATGGCATTTCAACAGCGACCAGAAAAATCCCTTTTAGCTTAAAACATTATGTAGAGTGG

CAAATCGGTTATGATGTCCCCATTAAAGATAAAGAA---AAATTGGAACTCACTACCCTA

AAAGATGAAAAATATCATTTTTTAGGGGCTAATAATAAAGTAAAAACCCTTTATGAATTG

AGTGAGATAATCTATTACGCTAAGCAATTAAATTTAATCAGT---------TTAGAAAAT

TTAGAAAATACTTTAAAATATTTAGAAAAACAAAAACAATTTATAGAAGATAATTTCACG

ATTACAAGAGAAAGATTTAGATCGCATCAATTTGGTGGCGTGGATTTTGAACTTTCACGC

ATCTCTTATCCTTTACTCATTCATTCTTTTAATGATAATCAGTTGAGCGAAATCGTTATT

AGAGAGCAACAATATGGCTCTAAAACCCAAGCCATG---CTGTATTTTTGCTTTTCTATT

TTGGAATTAAAAACCGCTACTCCCTTATTAAATAGAACGGCTGCACTCAAAGAACATGCC

CTTTTAACTATCCATAAAACCAACGCTCTTGTGTTTTTAGAAATGCTTAAAATTTTTGGA

CTTTTAAGCCAAGCGCACCATAACGATGTGTTAAAGATTTTAGAAAAAATACTTCAAAAT

>38:5

GTGAGTTTGATTAAAGCTAGTGGTGATAAAAAAGTGATTGAGGTTTCTATTCCTTTAACT

------------TCAATTTCAGGCAAAGTGCGTGTGAAAATCAGACATGCCTTTAGCGAT

TATGGTATTTCAACAGCGACTAGAAAAATCCCTTTTAGTTTAAAACATTATGTAGAGTGG

CAGATCGGTTATGATGTCCCTATTAAAGATAAAGAA---AAATTTGAACTCACTACTTTA

AAAGATGAAAAATATCATTTTTTAGGGGCTAATAATAAAGCAAAGACTCTTTATGAATTG

AGCGAAATGATTGATTACGCTAAGCAATTAGGTTTAATCAGT---------TTAGAAAAT

TTAGAAAATACTTTAAAATATTTAGAAAAACAAAAACAATTTATAGAAGATAATTTTATG

ATTACAAGAGAAAGATTTAGATCGCATCAATTTGGTGGCATGGATTTTGAACTCTCACGC

ATTTCTTATCCCTTACTCATTCATTCTTTTAATGATAATCAGTTGAGCGAAATAGTTATT

AGAGAGCAACAATATGGCTCTAAAACCCAAGCCATG---CTGTATTTTTGCTTTTCTATT

TTGGAGTTAAAAACCGCTACCCCCTTATTAAATAGAACCGCTACACTCAAAGAACATGCT

TTTTTAACCATCCATAAAACCAACGCTCCCATGTTTTTAGAAATGCTTAAAATTTTTGGA

CTTTTAAGCCAAGCGCACCATAACGATGTGTTAAGGATTTTAGAAAAAATACTTCAAAAT

>CA2

GTGAGTTTGATTAAAGTTAATAATAATAAAAAAGTGATTGAGGTTTCTATTCCTTTAACT

------------TCAATTTCAGGCAAAGTGCGTGTGAAAATCAGACATGCCTTTAGCGAT

TATGGCATTTCAACAGCGACTAGAAAAATCCCTTTTAGTTTAAAACATTATGTAGAGTGG

CAAATCGGTTATGATGTCCCCATTAAAGATAAAGAA---AAATTTGAACTCACTACTTTA

AAAGATGAAAAATATCATTTTTTAGGGGCTAATAATAAAGTAAAAACCCTTTATGAATTG

AGTGAGATAATCTATTACGCTAAGCAATTAAATTTAATCAGT---------TTAGAAAAT

TTAGAAAATACTTTAAAATATTTAGAAAAACAAAAACAATTTATAGAAGATAATTTTATG

ATTACAAGAGAAAGATTTAGATCGCATCAATTTGGTGGTATGGATTTTGAACTTTCACAC

ATTTCTTATCCTTTACTCATTCATTCTTTTAATGATAATCAATTGAGCGAAATTGTTATT

AGAGAACAACAATATGGTTCTAAAACCCAAGCTATG---CTGTATTTTTGCTTTTCTATT

TTGGAATTAAAAACCGCTACTCCCTTATTAAACAGAACGGCTATGCGCAAAGAACATGCT

CTTTTGATTATCCATAAAACCAACGCTCCCATGTTTTTAGAAATGCTTAAAATTTTTGGA

CTTTTAAGCCAAGCGCACCATGACGATGTGTTAAAGATTTTAGAAAAAATACTTCAAAAT

>B136A

GTGAGTTTGATTAAAGTTAGTGGTGATAAAAAAGCGATTGAGGTTTCTATTCCTTTAACT

------------TCAATTTCAGGCAAAGTGCGTGTGAAAATCAGACATGCCTTTAGCGAT

TATGGTGTTTCAACAGCGACTAGAAAAATCCCTTTCAGTTTAAAACATTATGTAGAGTGG

CAGATCGGTTATGATGTCCCCATTAAAGATAAAGAA---AAATTTGAACTCACTACTTTA

AAAGATGAAAAATATCATTTTTTAGGGGCTAATGATAAAGTAAAAACTCTTTATGAATTG

AGCGAAATGATTTATTACGCTAAGCAATTAGGTTTAATCAGT---------TTAGAAAAT

TTAGAAAATACTTTAAAATATTTAGAAAAACAAAAACAATTTATAGAAGATAATTTTATG

ATTACAAGAGAAAGATTTAGATTGCATCAATTTGGTGGCATGGATTTTGAACTCTCACGC

ATTTCTTATCCTTTGCTCATTCATTCTTTTAATGATAATCAGTTGAGCGAAATAGTTATT

AGAGAACAACAATACGGCTCTAAAACCCAAGCCATG---CTGTATTTTTGCTTTTCTATT

TTGGAATTAAAAACCGCTACCCCCTTATTAAATAGAACGGCTATGCTCAAAGAACATGCT

TTTTTGATTATCCATAAAACCAACGCTCCCATGTTTTTAGAAATGCTTAAAATTTTTGGA

CTTTTAAGCCAAGCGCACCATAGCGATGTGTTAAAGATTTTAGAAAAAATACTTCAAAAT

>UM408

GTGAGTTTGATTAAAATTAACCATGATGAAAAAGTGATTGAGGTTTCTATTCCTTTAACT

------------TCAATTTCAGGCAAAGTGCGTGTGAAAATCAGACATGCCTTTAGCGAT

TATGGTATTTCAACAGCGACTAGAAAAATCCCTTTTAGTTTAAAGCATTATGTAGAGTGG

CAAATCGGTTATGATGTCCCCATTAAAGATAAAGAA---AAATTTGAACTCACTACTTTA

AAAGATGAAAAATACCATTTTTTAGGGGCTAATAATAAAGTAAAAACTCTTTATGAATTG

AGCGAAATGATTTATTACGCTAAGCAATTAGGTTTAATCAGT---------TTAGAAAAT

TTAGAAAATACTTTAAAATATTTAGAAAAACAAAAACAATTTATAGAAGATAATTTTATG

ATTACAAGAGAAAAATTCAGATCGCATCAATTTGGGGGCATGGATTTTGAACTCTCACGC

ATTTCTTATCCTTTACTCATTCATTCTTTTAACGATAATCAGTTGAGCGAAATTGTTATT

AGAGAACAACAATATGGTTCTAAAACCCAAGCCATG---CTGTATTTTTGCTTTTCTATT

TTGGAGTTAAAAACCGCTACCCCCTTATTAAACAGAACGGCTATGCTCAAAGAGTATGCT

CTTTTGATTATCCATAAAACCAACGCTCCCATGTTTTTAGAAATGCTTAAAATTTTTGGG

CTTTTAAGCCAAGCACACCATAGCGATGTGTTAAAGATTTTAGAAAAAATACTTCAAAAT

>20A8

GTGAGTTTGATTAAAATTAACCATGATGAAAAAGTGATTGAGGTTTCTATTCCTTTAACT

------------TCAATTTCAGGCAAAGTGCGTGTGAAAATCAGACATGCCTTTAGCGAT

TATGGTATTTCAACAGCGACTAGAAAAATCCCTTTTAGTTTAAAGCATTATGTAGAGTGG

CAAATCGGTTATGATGTCCCCATTAAAGATAAAGAA---AAATTTGAACTCACTGCTTTA

AAAGATGAAAAATACCATTTTTTAGGGGCTAATAATAAAGTAAAAACTCTTTATGAATTG

AGCGAAATGATTTATTACGCTAAGCAATTAGGTTTAATCAGT---------TTAGAAAAT

TTAGAAAATACTTTAAAATATTTAGAAAAACAAAAACAATTTATAGAAGATAATTTTATG

ATTACAAGAGAAAGATTTAGATCGCATCAATTTGGGGGCATGGATTTTGAACTCTCACGC

ATTTCTTATCCTTTACTCATTCATTCTTTTAACGATAATCAGTTGAGCGAAATTGTTATT

AGAGAACAACAATATGGTTCTAAAACCCAAGCCATG---CTGTATTTTTGCTTTTCTATT

TTGGAGTTAAAAACCGCTACCCCCTTATTAAACAGAACGGCTATGCTCAAAGAGTATGCT

CTTTTGATTATCCATAAAACCAACGCTCCCATGTTTTTAGAAATGCTTAAAATTTTTGGG

CTTTTAAGCCAAGCGCACCATAGCGATGTGTTAAAGATTTTAGAAAAAATACTTCAAAAT

>ZH135

------------------AGTGGTAATAAAAAAGCGATTGAGGTTTCTATTCCTTTAACT

------------TCAATTTCAGGCAAAGTGCGTGTGAAAATCAGACATGCTTTTAGCGAT

TATGGTATTTCAACAGCGACTAGAAAAATCCCTTTTAGTTTAAAACATTATGTAAAGTGG

CAAATCGGTTATGATGTCCCCATTAAAGATAAAGAA---AAATTTGAATTCACTACTTTA

AAAGATGAAAAATATCATTTTTTAGGGGCTAATAATAAAGTAAAAACTCTTTATGAATTG

AGCGAAATGATTTATTACGCTAATCAATTAGGTTTAATCAGT---------TTAGAAAAT

TTAGAAAATACTTTAAAATATTTAGAAAAACAAAAACAATTTATAGAAGATAATTTTATG

ATTACAAGAGAAAGATTTAGATCGCATCAATTTGGTGGCATGGATTTTGAACTCTCACGC

ATTTCTTATCCTTTACTCATTCATTCTTTTAATGATAATCAGTTGAGTGAAATTGTTATT

AGGGAACAACAATACGGCTCTAAAACCCAAGCCATG---CTGTATTTTTGCTTTTCTATT

TTGGAGTTAAAAACCGCTACCCCCTTATTAAACAGAACGGCTACACTCAAAGAACATGCC

CTTTTAACTATCCATAAAACCAACGCTCTTATGTTTTTAGAAATGCTTAAAATTTTTGGA

CTTTTAAGCCAAGCGCACCATAACGATGTGTTAAAGGTTTTAGAAAAAATACTTCAAAAT

>KH17

GTGAGTTTGATTAAAATTAACCATGATAAAAAAGTGATTGAGGTTTCTATTCCTTTAACT

------------TCAATTTCAGGCAAAGTGCGTGTGAAAATCAGACATGCCTTTAGCGAT

TATGGTATTTCAACAGCGACTAGAAAAATCCCTTTTAGTTTAAAACATTATGTAGAGTGG

CAGATCGGTTATGATGTCCCCATTAAAGATAAAGAA---AAATTTGAACTCACTGCTTTA

AAAGATAAAAAATATCATTTTTTAGGGGCTAATAATAAAGTAAAAACTCTTTATGAATTG

AGCGAAACGATTTATTACGCTAAGCAATTAGGTTTAATCAGT---------TTAGAAAAT

TTAGAAAATACTTTAAAATATTTAGAAAAACAAAAACAATTTATAGAAGATAATTTTATG

ATTACAAGAGAAAGATTTAGATCGCATCAATTTGGTGGTATGGATTTTGAACTTTCACGC

ATTTCTTATCCTTTACTCATTCATTCTTTTAACGATAATCAATTGAGTGAAATAGTTATT

AGAGAACAACAATACGGCTCTAAGACTCAAGCCATG---CTGTATTTTTGCTTTTCTATT

TTGGAGTTAAAAACCGCTACCCCCTTATTAAACAGAACGGCTGCACTCAAAGAGTATGCT

CTTTTGATTATCCATAAAACCAACGCTCCCATGTTTTTAGAAATGTTTAAAATTTTTGGA

CTTTTAAGCCAAGCGCACCATAACGATGTGTTAAAGATTTTAGAAAAAATACTTCAAAAT

>B44

GTGAGTTTGATTAAAGCTAGTGGTGATAAAAAAGTGATTGAGGTTTCCATTCCTTTAACT

------------TCAATTTCAGGCAAGGTGCGTGTGAAAATCAGACATGCCTTTAGCGAT

TATGGTATTTCAACAGCGACCAGAAAAATCCCTTTTAGTTTAAAACATTATGTAGAGTGG

CAAATCGGTTATGATGTCCCCATTAAAGATAAAGAA---AAATTGGAACTCACTACTTTA

AAAGATGAAAAATATCATTTTTTAGGGGCTAATAATAAAGTAAAGACTCTTTATGAATTG

AGCGAAATGATTTATTACGCTAAGCGATTGGGTTTAATCAGT---------TTAGAAAAT

TTAGAAAATACTTTAAAATATTTAGAAAAACAAAAACAATTTATAGAAGATAATTTTATG

ATTACAAGAGAAAGATTTAGATCGCATCAATTTGGTGGCATGGATTTTGAACTCTCATAC

ATTTCTTATCCTTTACTCATTCATTCTTTTAGTGATAATCAATTGAGCGAAATCGTTATT

AGAGAGCAACAATATGGCTCTAAAACCCAAGCCATG---CTGTATTTTTGCTTTTCTATT

TTGGAATTAAAAACCGCTACTCCCTTATTAAATAGAACGGCTATGCTCAAAGAACATGCC

CTTTTAACTATCCATAAAACCAACGCTCCCATGTTTTTAGAAATGCTTAAAATTTTTGGA

CTTTTAAGCCAAGCACACCATAACGATGTGTTAAAGATTTTAGAAAAAATACTTCAAAAT

>MCms1055

GTGAGTTTGATTAAGATTGATGATGATAAAAAAGCGATTGAGGTTTCTATTCCTTTAACT

------------TCAATTTCAGGCAAAGCGCGTGTGAAAATCAGACATGCCTTTAGCGAT

TATGGCATTTCAACAGCGACCAGAAAAATCCCTTTTAGTTTAAAGCATTATGTAGAGTGG

CAAATCGGTTATGATGTCCCTATTAAAGATAAAGAA---AAATTTAAACTCACTACTTTA

AAAGATGAAAAATACCATTTTTTAGGGGCCAATAATAAAGTAAAAACTCTTTATGAATTG

AGTGAAATAATTTATTACGCTAAGCAATTAAATTTAATCAGT---------TTAGAAAAT

TTAGAAAATACTTTAAAATATTTAGAAAAACAAAAACAATTCATAGAAGATAGTTTTATG

ATCACAAGAGAAAGATTTAGATCGCATCAATTTGGGGGCATGGATTTTGAACTTTCACGC

ATCTCTTATCCCTTACTCATTCATTCTTTTGATGATAATCAGTTGAGCGAAATTGTTATT

AGAGAACAACAATATGGCTCTAAAACCCAAGCCATG---CTGTATTTTTGCTTTTCTATT

TTGGAGTTAAAAACTGCTACTCCCTTATTAAACAGAACCGCTACGCTCAAAGAACATGCT

TTTTTAACCATCAATAAAACCAACGCTCTTGTGTTTTTAGAAATGCTTAAAGTTTTTGGC

CTCTTAAGCCAAGCGCACCATAACGATGTGTTAAAGATTTTAGAAAAAATACTTCAAAAT

>C-Mx-2010-100

GTGAGTTTGATTAAGATTGATGATGATAAAAAAGCGATTGAGGTTTCTATTCCTTTAACT

------------TCAATTTCAGGCAAAGCGCGTGTGAAAATCAGACATGCCTTTAGCGAT

TATGGCATTTCAACAGCGACCAGAAAAATCCCTTTTAGTTTAAAGCATTATGTAGAGTGG

CAAATCGGTTATGATGTCCCTATTAAAGATAAAGAA---AAATTTAAACTCACTACTTTA

AAAGATGAAAAATACCATTTTTTAGGGGCCAATAATAAAGTAAAAACTCTTTATGAATTG

AGTGAAATAATTTATTACGCTAAGCAATTAAATTTAATCAGT---------TTAGAAAAT

TTAGAAAATACTTTAAAATATTTAGAAAAACAAAAACAATTCATAGAAGATAGTTTTATG

ATCACAAGAGAAAGATTTAGATCGCATCAATTTGGGGGCATGGATTTTGAACTTTCACGC

ATCTCTTATCCCTTACTCATTCATTCTTTTGATGATAATCAGTTGAGCGAAATTGTTATT

AGAGAACAACAATATGGCTCTAAAACCCAAGCCATG---CTGTATTTTTGCTTTTCTATT

TTGGAGTTAAAAACTGCTACTCCCTTATTAAACAGAACCGCTACGCTCAAAGAACATGCT

TTTTTAACCATCAATAAAACCAACGCTCTTGTGTTTTTAGAAATGCTTAAAGTTTTTGGC

CTCTTAAGCCAAGCGCACCATAACGATGTGTTAAAGATTTTAGAAAAAATACTTCAAAAT

>55:1

GTGAGTTTGATTAGGATTGATAATAATAAAAAAGTAATTGGGGTTTCCATTCCTTTAACT

------------TCAATTTCAGGCAAAGCGCGTGTGAAAATCAGACATGCCTTTAGCGAT

TGTGGTATTTCAACAGCGACTAGAAAAATCCCTTTTAGTTTAAAGCATTATGTAGAGTGG

CAAATCGGTTATGATGTCCCCATTAAAGATAAAGAA---AAATTTGAGCTCACTACTTTA

AAAGATGAAAAATATCATTTTTTAGGGGCTAATAATAAAGCAAAGACTCTTTATGAATTG

AGCGAAATGATTGATTACGCTAAGCGATTGGGTTTAATCAGT---------TTAGAAAAT

TTAGAAAATACTTTAAAATATTTAGAAAAACAAAAACAATTTATAGAAGATAATTTTATG

ATTACAAGAGAAAGATTTAGATCGCATCAATTTGGTGGCATGGATTTTGAACTCTCACGC

ATTTCTTATCCTTTGCTCATTCATTCTTTTAATGATAATGAGTTGAGCGAAATCGTTATT

AGAGAGCAACAATACGGCTCTAAAACCCAAGCCATG---CTGTATTTTTGCTTTTCTATT

TTGGAGTTAAAAACCGCTACTCCCTTATTAAATAGAACGGCTGCACTCAAAGAACATGCC

CTTTTGATTATCCATGAAACCAACGCTCCCATGTTTTTAGAAATGCTTAAAATTTTTGGA

CTTTTAAGCCAAGCGCACCATAACGATGTGTTAAAGATTTTAGAAAAAATACTTCAAAAT

>CG22385

GTGAGTTTGATTAAAGTTAATGATGATAAAAAAGTAATTGAGATTTCTATTCCTTTAACT

------------TCTATTTCAGGCAAAGCGCGTGTGAAAATCAGACATGCCTTTAGCGAT

TATGGCATTTCAACAGCGACTAGAAAAATCCCTTTTAGTTTAAAACATTATGTAGAGTGG

CAAATCGGTTATGATGTCCCCATTAAAGATAAAGAA---AAATTTGAACTCACTACTTTA

AAAGATGAAAAATATCATTTTTTAGGGGCTAATAATAAAGTAAAAACTCTTTATGAATTG

AGCGAAATGATTTATTACGCTAAGCAATTAGGTTTAATCAGT---------TTAGAAAAT

TTAGAAAATACTTTAAAATATTTAGAAAAACAAAAACAATTTATAGAAGATAATTTCACG

ATTACAAGAGAAAGATTTAGATTACATCAATTTGGTGGCATGGATTTTGAACTTTCACGC

ATTTCTTATCCTTTACTCATTCGTTCTTTCAACGATAATCAGTTGAGCGAAATTATTATT

AGAGAGCAACAATATGGCTCTAAAACCCAAGCCATG---CTGTATTTTTGCTTTTCTATT

TTGGAGTTAAAAACCGCTACCCCTTTATTAAATAGAACGGCTGCACTCAAAGAACACGCT

CTTTTGATTATCCATAAAACCAACGCTCTCATGTTTTTAGAAATGCTTAAAATTTTTGGA

CTTTTAAGCCAAGTGCACCATAACGATGTGTTAAAGATTTTAGAAAAAATACTTCAAAAT

>22385

GTGAGTTTGATTAAAGTTAATGATGATAAAAAAGTAATTGAGATTTCTATTCCTTTAACT

------------TCTATTTCAGGCAAAGCGCGTGTGAAAATCAGACATGCCTTTAGCGAT

TATGGCATTTCAACAGCGACTAGAAAAATCCCTTTTAGTTTAAAACATTATGTAGAGTGG

CAAATCGGTTATGATGTCCCCATTAAAGATAAAGAA---AAATTTGAACTCACTACTTTA

AAAGATGAAAAATATCATTTTTTAGGGGCTAATAATAAAGTAAAAACTCTTTATGAATTG

AGCGAAATGATTTATTACGCTAAGCAATTAGGTTTAATCAGT---------TTAGAAAAT

TTAGAAAATACTTTAAAATATTTAGAAAAACAAAAACAATTTATAGAAGATAATTTCACG

ATTACAAGAGAAAGATTTAGATTACATCAATTTGGTGGCATGGATTTTGAACTTTCACGC

ATTTCTTATCCTTTACTCATTCGTTCTTTCAACGATAATCAGTTGAGCGAAATTATTATT

AGAGAGCAACAATATGGCTCTAAAACCCAAGCCATG---CTGTATTTTTGCTTTTCTATT

TTGGAGTTAAAAACCGCTACCCCTTTATTAAATAGAACGGCTGCACTCAAAGAACACGCT

CTTTTGATTATCCATAAAACCAACGCTCTCATGTTTTTAGAAATGCTTAAAATTTTTGGA

CTTTTAAGCCAAGTGCACCATAACGATGTGTTAAAGATTTTAGAAAAAATACTTCAAAAT

>SV355_2

GTGAGTTTGATTAAAGTTAATGATGATAAAAAAGCGATTGAGGTTTCTATTCCTTTAACT

------------TCCATTTCAGGCAAAGTTCGTGTGAAAATCAGACATGCCTTTAGCGAT

TATGGCATTTCAACAGCGACTAGAAAAATCCCTTTCAGTTTAAAACATTATGTAGAGTGG

CAAATCGGCTATGATGTCCCCATTAAAGATAAAGAA---AAATTTGAACTCACTACCCTA

AAAGATGAAAAATATCATTTTTTAGGGGCTAATAATAAAATAAAAACCCTTTATGAATTG

AGCGAAATAATTGATTACGCTAAGCGATTGGGTTTAATCAGT---------TTAGAAAAT

TTAGAAAATACTTTAAAATATTTAGAAAAACAAAAACAATTCATAGAAGATAGTTTTATG

ATCACAAGAGAAAGATTTAGATCGCATCAATTTGGTGGCATGGATTTTGAACTTTCACGC

ATCTCTTACCCTTTACTCATTCATTCTTTTAATGATAATCAGTTGAGCGAAATCGTTATT

AGAGAGCAACAATACGGCTCTAAAACCCAAGCCATG---CTATATTTTTGCTTTTCTATT

TTGGAGTTAAAAACCGCTACCCCTTTATTAAATAGAACCGCTGCCCTCAAAGAACATGCC

CTTTTAACTATCCATGAAACTAACGCTCTTGTATTTTTAGAAATGCTTAAAATTTTTGGA

CTTTTAAGCCAAGCGCACCATAACGATGTGTTAAAGATTTTAGAAAAAATACTTCAAAAT

>2019-1

GTGAGTTTGATTAGGATTGATGATAGTAAAAAAGCGATTGAGGTTTCTATTCCTTTAACT

------------TCCATTTCAGGCAAAGCGCGTGTGAAAATCAGACATGCCTTTAGCGAT

TATGGTATTTCAACAGCGACTAGAAAAATCCCTTTTAGTTTAAAACATTATGTAGAGTGG

CAGATCGGTTATGATGTCCCCATTAAAGATAAAGAA---AAATTGGAGCTCACTACTTTA

AAAGATGAAAAATACCATTTTTTAGGAGCTAATAATAAAGTAAAAACTCTTTATGAATTA

AGCGAAATGATTTATTACGCTAAGCAATTAAATTTAATCAGT---------TTAGAAAAT

TTAGAAAATACTTTAAAATATTTAGAAAAACAAAAACAATTTATAGAAGATAGTTTTATG

ATTACAAGAGAAAGATTTAGATTACATCAATTTGGTGGCATGGATTTTGAACTTTCACGC

ATTTCTTATCCTTTACTCATTCATTCTTTTAATGATAATCAGTTGAGTGAAATCGTTATT

AGAGAGCAACAATACGGCTCTAAAACCCAAGCCATG---CTGTATTTTTGCTTTTCTATT

TTGGAATTAAAAACCGCTACCCCTTTATTAAATAGAACCGCTACACTCAAAGAACATGCT

TTTTTAACTATCCATAAAACCAACGCTCTTGTGTTTTTAGAAATGCTTAAAATTTTTGGA

CTTTTAAGCCAAGCGCACCATAGCGATGTGTTAAAGATTTTAGAAAAAATACTTCAAAAT

>2019-8

GTGAGTTTGATTAGGATTGATGATAGTAAAAAAGCGATTGAGGTTTCTATTCCTTTAACT

------------TCCATTTCAGGCAAAGCGCGTGTGAAAATCAGACATGCCTTTAGCGAT

TATGGTATTTCAACAGCGACTAGAAAAATCCCTTTTAGTTTAAAACATTATGTAGAGTGG

CAGATCGGTTATGATGTCCCCATTAAAGATAAAGAA---AAATTGGAGCTCACTACTTTA

AAAGATGAAAAATACCATTTTTTAGGAGCTAATAATAAAGTAAAAACTCTTTATGAATTA

AGCGAAATGATTTATTACGCTAAGCAATTAAATTTAATCAGT---------TTAGAAAAT

TTAGAAAATACTTTAAAATATTTAGAAAAACAAAAACAATTTATAGAAGATAGTTTTATG

ATTACAAGAGAAAGATTTAGATTACATCAATTTGGTGGCATGGATTTTGAACTTTCACGC

ATTTCTTATCCTTTACTCATTCATTCTTTTAATGATAATCAGTTGAGTGAAATCGTTATT

AGAGAGCAACAATACGGCTCTAAAACCCAAGCCATG---CTGTATTTTTGCTTTTCTATT

TTGGAATTAAAAACCGCTACCCCTTTATTAAATAGAACCGCTACACTCAAAGAACATGCT

TTTTTAACTATCCATAAAACCAACGCTCTTGTGTTTTTAGAAATGCTTAAAATTTTTGGA

CTTTTAAGCCAAGCGCACCATAGCGATGTGTTAAAGATTTTAGAAAAAATACTTCAAAAT

>C-Mx-2011-145

GTGAGTTTGATTAAAGTTAATGATGATAAAAAAGCGATTGAGGTTTCTATTCCTTTAACT

------------TCCATTTCAGGCAAAGCGCGTGTGAAAATCAGACACGCCTTTAGCGAT

TATGGTATTTCAACAGCGACTAGAAAAATCCCTTTCAGTTTAAAACATTATGTAGAGTGG

CAAATCGGTTATGATGTCCCCATTGAAGATAAAGAA---AAATTTGAGCTCACTACTTTA

AAAGATGAAAAATATCATTTTTTAGGGGCTAATAATAAAGTAAAAACCCTTTATGAATTG

AGCGAAATGATTGATTACGCTAAGCGATTGGGTTTAATCAGT---------TTAGAAAAT

TTAGAAAATACTTTAAAATATTTAGAAAAGCAAAAACAATTCATAGAAGATAATTTTATG

ATTACAAGAGAAAGATTTAGATCGCATCAATTTGGGGGCATGGATTTTGAACTTTCACGC

ATTTCTTATCCTTTACTCATTCATTCTTTTAATGATAATCAATTGAGCGAAATCGTTATT

AGAGAGCAACAATACGGCTCTAAAACCCAAGCCATG---CTATATTTTTGCTTTTCTATT

TTGGAATTAAAAACCGCTACCCCCTTATTAAATAGAACGGCTGCACTCAAAGAACATGCC

CTTTTAACTATCCATAAAACTAATGCTCTTGTGTTTTTAGAAATGCTTAAAATTTTTGGA

CTTTTAAGCCAAGTGCATCATAATGATGTGTTAAAGATTTTAGAAAAAATACTTGAAAAT

>Nic49-A

GTGAGTTTGATTAAGATTGATAATGATAAAAAAGTGATTGAGGTTTCTATTCCTTTAACT

------------TCCATTTCAGGCAAAGTGCGTGTGAAAATCAGGCATGCCTTTAGCGAT

TATGGCATTTCAACAGCGACCAGAAAAATCCCTTTTAGCTTAAAGCATTATGTAGAGTGG

CAAATCGGTTATGATGTCCCTATTAAAGATAAAGAA---AAATTTGAACTCACTACTTTA

AAAGATGAAAAATATCATTTTTTAGGGGCTAATAATAAAGTAAAAACCCTTTATGAATTG

AGCGAAATAATTGATTACGCTAAGCGATTGGGTTTAATCAGT---------TTAGAAAAT

TTAGAAAATACTTTAAAATATTTAGAAAAACAAAAACAATTCATAGAAGATAGTTTTATA

ATCACAAGAGAAAGATTTAGATCGCATCAATTTGGTGGCATGGATTTTGAACTTTCACGC

ATTTCTTATCTTTTACTCATTCATTCTTTTAATGATAATCAATTGAGTGAAATCGTTATT

AGAGAGCAACAATATGGCTCTAAAACCCAAGCCATG---CTGTATTTTTGCTTTTCTATT

CTAGAATTAAAAACCGCTACCCCCTTATTAAACAGAACGGCTATGCTCAAAGAGCATGCT

CTTTTGATTATCCATAAAACCAACGCTCTCATGTTTTTAGAAATGCTTAAAATTTTTGGG

CTTTTAAGCCAAGCGCACCATAACGATGTGTTAAAGATTTTAGAAAAAATACTTCAAAAT

>MG2003-107

GTGAGTTTGATTAAAGTTAATGATGATAAAAAAGTGATTGAGGTTTCTATTCCTTTAACT

------------TCCATTTCAGGCAAAGTTCGTGTGAAAATTAGGCATGCCTTTAGTGAT

TATGGTATTTCAACAGCGACTAGAAAAATCCCTTTCAGTTTAAAGCATTATGTAGAGTGG

CAAATCGGTTATGATGTCCCCATTAAAGATAAAGAA---AAATTTGAACTCACTACTTTA

AAAGATGAAAAATATCATTTTTTAGGGGCTAATAATAGAGTAAAGACTCTTTATGAATTG

AGCGAAATAATTGATTACGCTAAGCGATTGGGTTTAATCAGT---------TTAGAAAAT

TTAGAAAATACTTTAAAATATTTAGAAAAACAAAAACAATTCATAGAAGATAGTTTTATA

ATTACAAGAGAAAGATTTAGATCGCATCAATTTGGTGGCATGGATTTTGAACTTTCACGC

ATCTCTTATCCTTTACTCATTCATTCTTTTAATGATAATCAGTTGAGCGAAATCGTTATT

AGAGAGCAACAATACGGCTCTAAAACCCAAGCTATG---CTGTATTTTTGCTTTTCTATT

CTGGAATTAAAAACCGCTACCCCTTTATTAAATAGGACCGCTGCCCTCAAAGAACATGCC

CTTTTAACTATCCACAAAACTAACGCTCTTGTGTTTTTAGAAATGCTTAAAATTTTTGGG

CTTTTAAGCCAAGCGCACCACAACGATGTGTTAAAGATTTTAGAAAAAATACTTGAAAAT

>KH0070

GTGAGTTTGATTAAAGTCGACTATGATAAAAAAGTGGTTGAGGTTTCTATTCCTTTAACT

------------TCAATTTCAGGCAAAGTGCGTGTGAAGATCAGACATGCCTTTAGCGAT

TATGGTATTTCAACAGCGACTAGAAAAATCCCTTTTAGTTTAAAGCATTATGTAGAGTGG

CAGATCGGTTATGATGTCCCCATTAAAGATAAAGAA---AAATTTGAACTCACTACTTTA

AAAGATGAAAAATATCATTTTTTAGGGGCTAATAATAAAGTAAAAACTCTTTATGAATTG

AGCGAAATGATTGATTACGCTAAGCAATTAGGTTTAATCAGT---------TTAGAAAAT

TTAGAAAATACTTTAAAATATTTAGAAAAACAAAAACAATTTATAGAAGATAATTTCACG

ATTACAAGAGAAAGATTTAGATCGCATCAATTTGGGGGCATGGATTTTGAACTTTCACGC

ATTTCTTATCCTTTACTCATTCATTCTTTTAATGATAATCAGTTGAGCGAAATCGTTATT

AGAGAACAACAATATGGCTCTAAAACCCAAGTCATG---CTGTATTTTTGCTTTTCAATT

TTGGAGTTAAAAACCGCTACCCCCTTATTAAACAGAACCGCTACACTCAAAGAACATGCC

CTTTTGATTATCCATAAAACTAACGCTCTCATGTTTTTAGAAATGCTTAAAATTTTTGGA

CTTTTAAGCCAAGCACACCATAGCGATGCGTTAAAGATTTTAGAAAAAATACTTCAAAAT

>2004-101

GTGAGTTTGATTAAAGTTAATGATGATAAAAAAGTGATTGAGGTTTCTATTCCTTTAACT

------------TCCATTTCAGGCAAAGTTCGTGTGAAAATTAGGCATGCCTTTAGTGAT

TATGGTATTTCAACAGCGACTAGAAAAATCCCTTTCAGTTTAAAGCATTATGTAGAGTGG

CAAATCGGTTATGATGTCCCCATTAAAGATAAAGAA---AAATTTGAACTCACTACTTTA

AAAGATGAAAAATATCATTTTTTAGGGGCTAATAATAGAGTAAAGACTCTTTATGAATTG

AGCGAAATAATTGATTACGCTAAGCGATTGGGTTTAATCAGT---------TTAGAAAAT

TTAGAAAATACTTTAAAATATTTAGAAAAACAAAAACAATTCATAGAAGATAGTTTTATA

ATTACAAGAGAAAGATTTAGATCGCATCAATTTGGTGGCATGGATTTTGAACTTTCACGC

ATCTCTTATCCTTTACTCATTCATTCTTTTAATGATAATCAGTTGAGCGAAATCGTTATT

AGAGAGCAACAATACGGCTCTAAAACCCAAGCTATG---CTGTATTTTTGCTTTTCTATT

CTGGAATTAAAAACCGCTACCCCTTTATTAAATAGGACCGCTGCCCTCAAAGAACATGCC

CTTTTAACTATCCACAAAACTAACGCTCTTGTGTTTTTAGAAATGCTTAAAATTTTTGGA

CTTTTAAGCCAAGCGCACCACAACGATGTGTTAAAGATTTTAGAAAAAATACTTGAAAAT

>G-Mx-2006-513

GTGAGTTTGATTAAAGTTAATGATGATAAAAAAGTGATTGAGGTTTCTATTCCTTTAACT

------------TCCATTTCAGGCAAAGTTCGTGTGAAAATTAGGCATGCCTTTAGTGAT

TATGGTATTTCAACAGCGACTAGAAAAATCCCTTTCAGTTTAAAGCATTATGTAGAGTGG

CAAATCGGTTATGATGTCCCCATTAAAGATAAAGAA---AAATTTGAACTCACTACTTTA

AAAGATGAAAAATATCATTTTTTAGGGGCTAATAATAGAGTAAAGACTCTTTATGAATTG

AGCGAAATAATTGATTACGCTAAGCGATTGGGTTTAATCAGT---------TTAGAAAAT

TTAGAAAATACTTTAAAATATTTAGAAAAACAAAAACAATTCATAGAAGATAGTTTTATA

ATTACAAGAGAAAGATTTAGATCGCATCAATTTGGTGGCATGGATTTTGAACTTTCACGC

ATCTCTTATCCTTTACTCATTCATTCTTTTAATGATAATCAGTTGAGCGAAATCGTTATT

AGAGAGCAACAATACGGCTCTAAAACCCAAGCTATG---CTGTATTTTTGCTTTTCTATT

CTGGAATTAAAAACCGCTACCCCTTTATTAAATAGGACCGCTGCCCTCAAAGAACATGCC

CTTTTAACTATCCACAAAACTAACGCTCTTGTGTTTTTAGAAATGCTTAAAATTTTTGGG

CTTTTAAGCCAAGCGCACCACAACGATGTGTTAAAGATTTTAGAAAAAATACTTGAAAAT

>2017-192

GTGAGTTTGATTAAAGTTAATGATGATAAAAAAGTGATTGAGGTTTCTATTCCTTTAACT

------------TCCATTTCAGGCAAAGTTCGTGTGAAAATTAGGCATGCCTTTAGTGAT

TATGGTATTTCAACAGCGACTAGAAAAATCCCTTTCAGTTTAAAGCATTATGTAGAGTGG

CAAATCGGTTATGATGTCCCCATTAAAGATAAAGAA---AAATTTGAACTCACTACTTTA

AAAGATGAAAAATATCATTTTTTAGGGGCTAATAATAGAGTAAAGACTCTTTATGAATTG

AGCGAAATAATTGATTACGCTAAGCGATTGGGTTTAATCAGT---------TTAGAAAAT

TTAGAAAATACTTTAAAATATTTAGAAAAACAAAAACAATTCATAGAAGATAGTTTTATA

ATTACAAGAGAAAGATTTAGATCGCATCAATTTGGTGGCATGGATTTTGAACTTTCACGC

ATCTCTTATCCTTTACTCATTCATTCTTTTAATGATAATCAGTTGAGCGAAATCGTTATT

AGAGAGCAACAATACGGCTCTAAAACCCAAGCTATG---CTGTATTTTTGCTTTTCTATT

CTGGAATTAAAAACCGCTACCCCTTTATTAAATAGGACCGCTGCCCTCAAAGAACATGCC

CTTTTAACTATCCACAAAACTAACGCTCTTGTGTTTTTAGAAATGCTTAAAATTTTTGGA

CTTTTAAGCCAAGCGCACCACAACGATGTGTTAAAGATTTTAGAAAAAATACTTGAAAAT

>MM2006-106

GTGAGTTTGATTAAAGTTAATGATGATAAAAAAGTGATTGAGGTTTCTATTCCTTTAACT

------------TCCATTTCAGGCAAAGTTCGTGTGAAAATTAGGCATGCCTTTAGTGAT

TATGGTATTTCAACAGCGACTAGAAAAATCCCTTTCAGTTTAAAACATTATGTAGAGTGG

CAAATCGGTTATGATGTCCCCATTAAAGATAAAGAA---AAATTTGAACTCACTACTTTA

AAAGATGAAAAATATCATTTTTTAGGGGCTAATAATAGAGTAAAGACTCTTTATGAATTG

AGCGAAATAATTGATTACGCTAAGCGATTGGGTTTAATCAGT---------TTAGAAAAT

TTAGAAAATACTTTAAAATATTTAGAAAAACAAAAACAATTCATAGAAGATAGTTTTATA

ATTACAAGAGAAAGATTTAGATCGCATCAATTTGGTGGCATGGATTTTGAACTTTCACGC

ATCTCTTATCCTTTACTCATTCATTCTTTTAATGATAATCAGTTGAGCGAAATCGTTATT

AGAGAGCAACAATACGGCTCTAAAACCCAAGCTATG---CTGTATTTTTGCTTTTCTATT

CTGGAATTAAAAACCGCTACCCCTTTATTAAATAGGACCGCTGCCCTCAAAGAACATGCC

CTTTTAACTATCCACAAAACTAACGCTCTTGTGTTTTTAGAAATGCTTAAAATTTTTGGA

CTTTTAAGCCAAGCGCACCACAACGATGTGTTAAAGATTTTAGAAAAAATACTTGAAAAT

>MM2006-103

GTGAGTTTGATTAAAGTTAATGATGATAAAAAAGTGATTGAGGTTTCTATTCCTTTAACT

------------TCCATTTCAGGCAAAGTTCGTGTGAAAATTAGGCATGCCTTTAGTGAT

TATGGTATTTCAACAGCGACTAGAAAAATCCCTTTCAGTTTAAAACATTATGTAGAGTGG

CAAATCGGTTATGATGTCCCCATTAAAGATAAAGAA---AAATTTGAACTCACTACTTTA

AAAGATGAAAAATATCATTTTTTAGGGGCTAATAATAGAGTAAAGACTCTTTATGAATTG

AGCGAAATAATTGATTACGCTAAGCGATTGGGTTTAATCAGT---------TTAGAAAAT

TTAGAAAATACTTTAAAATATTTAGAAAAACAAAAACAATTCATAGAAGATAGTTTTATA

ATTACAAGAGAAAGATTTAGATCGCATCAATTTGGTGGCATGGATTTTGAACTTTCACGC

ATCTCTTATCCTTTACTCATTCATTCTTTTAATGATAATCAGTTGAGCGAAATCGTTATT

AGAGAGCAACAATACGGCTCTAAAACCCAAGCTATG---CTGTATTTTTGCTTTTCTATT

CTGGAATTAAAAACCGCTACCCCTTTATTAAATAGGACCGCTGCCCTCAAAGAACATGCC

CTTTTAACTATCCACAAAACTAACGCTCTTGTGTTTTTAGAAATGCTTAAAATTTTTGGA

CTTTTAAGCCAAGCGCACCACAACGATGTGTTAAAGATTTTAGAAAAAATACTTGAAAAT

>2004-102

GTGAGTTTGATTAAAGTTAATGATGATAAAAAAGTGATTGAGGTTTCTATTCCTTTAACT

------------TCCATTTCAGGCAAAGTTCGTGTGAAAATTAGGCATGCCTTTAGTGAT

TATGGTATTTCAACAGCGACTAGAAAAATCCCTTTCAGTTTAAAGCATTATGTAGAGTGG

CAAATCGGTTATGATGTCCCCATTAAAGATAAAGAA---AAATTTGAACTCACTACTTTA

AAAGATGAAAAATATCATTTTTTAGGGGCTAATAATAGAGTAAAGACTCTTTATGAATTG

AGCGAAATAATTGATTACGCTAAGCGATTGGGTTTAATCAGT---------TTAGAAAAT

TTAGAAAATACTTTAAAATATTTAGAAAAACAAAAACAATTCATAGAAGATAGTTTTATA

ATTACAAGAGAAAGATTTAGATCGCATCAATTTGGTGGCATGGATTTTGAACTTTCACGC

ATCTCTTATCCTTTACTCATTCATTCTTTTAATGATAATCAGTTGAGCGAAATCGTTATT

AGAGAGCAACAATACGGCTCTAAAACCCAAGCTATG---CTGTATTTTTGCTTTTCTATT

CTGGAATTAAAAACCGCTACCCCTTTATTAAATAGGACCGCTGCCCTCAAAGAACATGCC

CTTTTAACTATCCACAAAACTAACGCTCTTGTGTTTTTAGAAATGCTTAAAATTTTTGGA

CTTTTAAGCCAAGCGCACCACAACGATGTGTTAAAGATTTTAGAAAAAATACTTGAAAAT

>NCTC13094

GTGAGTTTGATTAAAGTTAATGATGATAAAAAAGTAATTGAGATTTCTATTCCTTTAACT

------------TCTATTTCAGGCAAAGCGCGTGTGAAAATCAGACATGCCTTTAGCGAT

TATGGCATTTCAACAGCGACTAGAAAAATCCCTTTTAGTTTAAAACATTATGTAGAGTGG

CAAATCGGTTATGATGTCCCCATTAAAGATAAAGAA---AAATTTGAACTCACTACTTTA

AAAGATGAAAAATATCATTTTTTAGGGGCTAATAATAAAGTAAAAACCCTTTATGAATTG

AGTGAGATAATCTATTACGCTAAGCAATTAAATTTAATCAGT---------TTAGAAAAT

TTAGAAAATACTTTAAAATATTTAGAAAAACAAAAACAATTTATAGAAGATAATTTCACG

ATTACAAGAGAAAGATTCAGATCGCATCAATTTGGTGGCATGGATTTTGAACTTTCACGC

ATTTCTTATCCTTTACTCATTCATTCTTTTAATGATAATCAGTTGAGTGAAATCGTTATT

AGAGAGCAACAATACGGCTCTAAAGTCCAAGCCATG---CTGTATTTTTGCTTTTCTATT

TTGGAATTAAAAACCGCTACCCCTTTATTAAATAGAACCGCTACACTCAAAGAACATGCT

TTTTTAACTATCCATAAAACCAACGCTCTTGTGTTTTTAGAAATGCTTAAAATTTTTGGA

CTTTTAAGCCAAGCGCACCATAACGATGTGTTAAAGATTTTAGAAAAAATACTTCAAAAT

>HP99647

GTGAGTTTGATTAGGATTGATGATAGTAAAAAAGTAATTGAGGTTTCTATTCCTTTAACT

------------TCAATTTCAGGCAAAGCGCGTGTGAAAATCAGGCATGCCTTTAGCGAT

TATGGTATTTCAACAGCGACTAGAAAAATCCCTTTTAGTTTAAAACATTATGTAGAGTGG

CAAATCGGTTATGATGTCCCTATTAAAGATAAAGAA---AAATTGGAGCTCACTACCCCA

AAAGATGAAAAATATCATTTTTTAGGGGCTAATAATAAAGTAAAGACTCTTTATGAATTG

AGTGAAATAATCTATTACGCTAAGCAATTAAATTTAATCAGT---------TTAGAAAAT

TTAGAAAATACTTTAAAATATTTAGAAAAACAAAAACAATTTATAGAAGATAATTTCACG

ATTACAAGAGAAAGATTTAGATCGCATCAATTTGGTGGCATGGATTTTGAACTCTCACGC

ATTTCTTATCCCTTACTCATTCATTCTTTTAATGATAATCAATTGAGCGAAATCGTTATT

AGAGAGCAACAATACGGCTCTAAAACCCAAGCCATG---CTGTATTTTTACTTTTCTATT

TTGGAATTAAAAACCGCTACCCCTTTATTAAATAGAACCGCTACGCTCAAAGAACATGCT

CTTTTAACTATCCATAAAACCAACGCTCTCATGTTTTTAGAAATGCTTAAAATTTTTGGA

CTTTTAAGCCAAGCGCACCATAACGATGTGTTAAAGATTTTAGAAAAAATACTTCAAAAT

>CHL14

GTGAGTTTGATTAAAGTTAGTGGTGATAAAAAAGCGATTGAGGTTTCTATTCCCTTAACT

------------TCAATTTCAGGTAAAGTGCGTGTGAAAATCAGACATGCCTTTAGTGAT

TATGGTATTTCAACAGCGACTAGAACAATCCCTTTTAGTTTAAAACATTATGTAGAGTGG

CAGATCGGTTATGATGTCCCCATTAAAGATAAAGAA---AAATTTGAACTCACTACTTTA

AAAGATGAAAAATATCATTTTTTAGGGGCTAATAATAAAGTAAAAACTCTTTATGAATTG

AGCGAGATCATTTATTATGCCAAACAATTAGGTTTAATCAGT---------TTAGAAAAT

TTAGAAAATACTTTAAAATATTTAGAAAAACAAAAACAATTTATAGAAGATAATTTTATG

ATTACAAGAGAAAGATTTAGATCGCATCAATTTGGTGGCATGGATTTTGAACTTTCACGC

ATTTCTTATCCTTTACTCATTCATTCTTTTGATGATAATCAATTGAGCGAAATTGTTATT

AGAGAGCAACAATATGGTTCTAAAACCCAAGCTATG---CTGTATTTTTGCTTTTCTATT

TTGGAGTTAAAAACCGCTACTCCCTTATTAAACAGAACGGCTACGCCCAAAGAACATGCT

CTTTTGATTATCCATAAAACCAACGCTCCCATGTTTTTAGAAATGCTTAAAATTTTTGGA

CTTTTAAGCCAAGCGCACCATGACGATGTGTTAAAGATTTTAGAAAAAATACTTCAAAAT

>KH0032

GTGAGTTTGATTAGGATTGATGATAGTAAAAAAGCGATTGAGGTTTCTATTCCTTTAACT

------------TCAATTTCAGGCAAAGTGCGTGTGAAAATCAGACATGCCTTTAGCGAT

TATGGTATTTCAACAGCGACTAGAAAAATCCCTTTTAGTTTAAAGCATTATGTAGAGTGG

CAAATCGGTTATGATGTCCCCATTAAAGATAAAGAA---AAATTTGAACTCACTACTTTA

AAAGATAAAAAATATCATTTTTTAGGGGCTAATAATAAAGTAAAAACTCTTTATGAATTG

AGCGAGATCATTTACTATGCCAAGCAATTAGATTTAATCAGT---------TTAGAAAAT

TTAGAAAATACTTTAAAATATTTAGAAAAACAAAAACAATTTATAGAAGATAATTTTATG

ATTACAAGAGAAAGATTTAGATCACATCAATTTGGTGGCATGGATTTTGAACTTTCATGC

ATTTCTTATCCTTTACTCATTCATTCTTTTAATGATAATCAATTGAGCGAAATTGTTATT

AGAGAACAACAATATGGTTCTAAAACCCAAGCTATG---CTGTATTTTTGCTTTTCTATT

TTAGAATTAAAAACCGCTACTCCCTTATTAAACAGAACGGCCACACTCAAAGAACATGCT

CTTTTGATTATCCATAAAACCAACGCTCCCATGTTTTTAGAAATGCTTAAAATTTTTGGA

CTTTTAAGTCAAGCGCACCATGACGATGTGTTAAAGATTTTAGAAAAAATACTTCAAAAT

>HP13021

GTGAGTTTGATTAAAATTAACCATGATGAAAAAGTGATTGAGGTTTCTATTCCTTTAACT

------------TCAATTTCAGGCAAAGTGCGTGTGAAAATCAGACATGCCTTTAGCGAT

TATGGTATTTCAACAGCGACTAGAAAAATCCCTTTTAGCTTAAAACATTATGTAGAGTGG

CAAATCGGTTATGATGTCCCCATTAAAGATAAAGAA---AAATTTGAGCTCACTACCCTA

AAAGATGAAAAATATCATTTTTTAGGGGCTAATAATAAAGTAAAAACCCTTTATGAATTG

AGTGAGATAATCTATTACGCTAAGCAATTAAATTTAATCAGT---------TTAGAAAAT

TTAGAAAATAATTTAAAATATTTAGAAAAACAAAAACAATTTATAGAAGATAATTTTATG

ATTACAAGAGAAAGATTTAGATTACATCAATTTGGTGGCATGGATTTTGAACTTTCACGC

ATTTCTTATCCTTTGCTCATTCATTCTTTTAATGATAATCAATTGAGTGAAATCGTTATT

AGAGAGCAACAATACGGCTCTAAAACCCAAGCCATG---CTGTATTTTTGCTTTTCTATT

TTGGAATTAAAAACCGCTACCCCCTTATTAAACAGAACGGCTGCACTCAAAGAACATGCC

CTTTTAACTATCCATAAAACCAACACTCTTGTGTTTTTAGAAATGCTTAAAATTTTTGGA

CTTTTAAGCCAAGCACACCATAACGATGTGTTAAAGATTTTAGAAAAAACACTTCAAAAT

>CHL54

GTGAGTTTGATTAAAGTTAATAATAATAAAAAAGTGATTGAGGTTTCTATTCTTTTAACT

------------TCAATTTCAGGCAAAGTGCGTGTGAAAATCAGACATGCCTTTAGCGAT

TATGGCATTTCAACAGCGACTAGAAAAATCCCTTTTAGTTTAAAACATTATGTAGAGTGG

CAAATCGGTTATGATGTCCCCATTAAAGATAAAGAA---AAATTTGAACTCACTACTTTA

AAAGATGAAAAATATCATTTTTTAGGGGCTAATAATAAAGTAAAAACCCTTTATGAATTG

AGTGAGATAATCTATTACGCTAAGCAATTAAATTTAATCAGT---------TTAGAAAAT

TTAGAAAATACTTTAAAATATTTAGAAAAACAAAAACAATTCATAGAAGATAATTTTATG

ATTACAAGAGAAAGATTTAGATCGCATCAATTTGGTGGCATGGGTTTTGAACTTTCACGC

ATTTCTTACCCTTTACTCATTTATTCTTTTAATGATAATCAATTGAGCGAAATCGTTATT

AGAGAGCAACAATATGGCTCTAAAACCCAAGCCATG---CTGTATTTTTGCTTTTCTATT

TTGGAATTAAAAACCGCTACCCCCTTATTAAATAGAACGGCTGCCCTCAAAGAACATGCC

CTTTTAACTATCCATAAAACCAACGCTCCCATGTTTTTAAAAATGCTTAAAATTTTTGGA

CTTTTAAGCCAAGCGCACCATGACGATGTGTTAAAGATTTTAGAAAAAATACTTCAAAAT

>UM411

---------------GTTGACTATGATAAAAAAGTGATTGAGGTTTCTGTTCCTTTAACT

------------TCAATTTCAGGCAAAGTGCGTGTGAAGATCAGACATGCCTTTAGCGAT

TATGGTATTTCAACAGCGACTAGAAAAATCCCTTTTAGTTTAAAGCATTATGTAGAGTGG

CAGATCGGTTATGATGTCCCCATTAAAGATAAAGAA---AAATTTGAACTCACTGCTTTA

AAAGATAAAAAATACCATTTTTTAGGGGCTAATAATAAAGTAAAAACTCTTTATGAATTG

AGCGAAATGATTTATTACGCTAAGCAATTAGGTTTAATCAGT---------TTAGAAAAT

TTAGAAAATACTTTAAAATATTTAGAAGAACAAAAACAATTTATAGAAGATAATTTTATG

ATTACAAGAGAAAGATTTAGATCGCATCAATTTGGTGGCATGGATTTTGAACTCTCACGC

ATTTCTTATCCTTTACTCATTCATTCTTTTAACGATAATCAATTGAGTGAAATAGTTATT

AGAGAACAACAATACGGCTCTAAGACACAAGCCATG---TTGTATTTTTGCTTTTCTATT

TTGGAGTTAAAAACCGCTACTCCCTTATTAAATAGAACGGCTGCACTCAAAGAGCATGCT

CTTTTGATTATCCATAAAACCAACGCTCTCATGTTTTTAGAAATGTTTAAAATTTTTGGG

CTTTTAAGCCAAGCGCACCATAACGATATGTTAAAGATTTTAGAAAAAATACTTCAAAAT

>Nic37-A

GTGAGTTTGATTAAAGTTAATGATGATAAAAAAGCGATTGAGGTTTCTATTCCTTTAACT

------------TCCATTTCAGGCAAAGCGCGTGTGAAAATCAGACATGCCTTTAGCGAT

TATGGCATTTCAACAGCGACCAGAAAAATCCCTTTTAGCTTAAAACATTATGTAGAGTGG

CAAATCGGTTATGATGCCCCCATTAAAGATAAAGAA---AAATTTGAACTCACTACCCTA

AAAGATGAAAAATATCATTTTTTAGGGGCTAATAATAAAGTAAAAACCCTTTATGAATTG

AGTGAGATAATCTATTACGCTAAGCAATTAAATTTAATCAGT---------TTAGAAAAT

TTAGAAAATACTTTAAAATATTTAGAAAAACAAAAACAATTCATAGAAGATAATTTCACG

ATCACAAGAGAAAGATTTAGATCGCATCAATTTGGGGGCATGGATTTTGAACTTTCACGC

ATTTCTTATCCTTTACTCATTCATTCTTTCAACGATAATCAGTTGAGCGAAATCGTTATT

AGAGAGCAACAATACGGCTCTAAAACCCAAGCCATG---CTGTATTTTTGCTTTTCTATT

CTGGAATTAAAAACCGCTACCCCCTTATTAAATAGAACGGCTGCACTCAAAGAACATGCC

CTTTTAACTATCCATAAAACCAACGCTCTTGTGTTTTTAGAAATGCTTAAAATTTTTGGA

CTTTTAAGCAAAGCGCACCATAACGATGTGTTAAAGATTTTAGAAAAAATACTTGAAAAT

>HP06058

GTGAGTTTGATTAGGATTGATGATAGTAAAAAAGCGATTGAGGTTTCTATTCCTTTAACT

------------TCAATTTCAGGCAAAGCGCGTGTGAAAATCAGACATGCCTTTAGCGAT

TATGGTATTTCAACAGCGACTAGAAAAATCCCTTTTAGCTTAAAACATTATGTAGAGTGG

CAAATCGGTTATGATGTCCCCATTAAAGATAAAGAA---AAATTTGAACTCACTACTTTA

AAAGATGAAAAATATCATTTTTTAGGGGCTAATAATAAAGTAAAGACTCTTTATGAATTG

AGTGAGATAATCTATTACGCTAAGCAATTAAATTTAATCAGT---------TTAGAAAAT

TTAGAAAATACTTTAAAATATTTAGAAAAACAAAAACAATTTATAGAAGATAATTTCACG

ATTACAAGAGAAAGATTTAGATCGCATCAATTTGGTGGCATGGATTTTGAACTCTCACGC

ATTTCTTATCCTTTACTCATTCATTCTTTTAATGATAATCAATTGAGCGAAATAGTTATT

AGAGAGCAACAATATGGTTCTAAAACCCAAGCCATG---CTGTATTTTTGCTTTTCTATT

TTGGAGTTAAAAACCGCTACCCCCTTATTAAACAGAACGGCTATGCTCAAAGAGCATGCC

CTTTTGATTATCCATAAAACCAACGCTCCCATGTTTTTAGAAATGCTTAAAATTTTTGGA

CTTTTAAGTCAAGCGCACCATAACGGCGTGTTAAAGATTTTAGAAAAAATACTTCAAAAT

>CA22339

GTGAGTTTGATTAGGATTGATGATAGTAAAAAAGCGATTGAGATTTCCATTCCTTTAACT

------------TCAATTTCAGGCAAAGTGCGTGTGAAAATCAGACATGCCTTTAGCGAT

TATGGTATTTCAACAGCGACTAGAAAAATCCCTTTTAGTTTAAAACATTATATAGAGTGG

CAGATCGGTTATGATGTCCCCATTAAAGATAAAGAA---AAATTTGAGCTCACTACCCTA

AAAGATGAAAAATATCATTTTTTAGGGGCTAATAATAAAGTAAAGACTCTTTATGAATTG

AGCGAAATGATTTATTACGCTAAGCAATTAAGTTTAATCGGT---------TTAGAAAAT

TTAGAAAATACTTTAAAATATTTAGAAAAACAAAAACAATTTATAGAAGATAATTTTATG

ATTACAAGAGAAAGATTTAGATTACATCAATTTGGTGACATGGATTTTGAACTCTCACGC

ATTTCTTATCCTTTGCTCATTCATTCTTTTAATGATAATCAGTTGAGCGAAATCGTTATT

AGAGAACAACAATACGGCTCTAAAACCCAAGCCATG---CTGTATTTTTGCTTTTCTATT

TTGGAGTTAAAAACTGCTACCCCCTTATTAAATAGAACGGCTGCACTCAAAGAACAGGCT

CTTTTAACCATCAATAAAACCAACGCTCTTATGTTTTTAGAAATGCTTAAAATTTTTGGA

CTTTTAAGCCAAGCGCACCATAGCGATGTGTTAAAGATTTTAGAAAAAATACTTCAAAAT

>22346

GTGAGTTTGATTAGGATTGATGATAGTAAAAAAGCGATTGAGATTTCCATTCCTTTAACT

------------TCAATTTCAGGCAAAGTGCGTGTGAAAATCAGACATGCCTTTAGCGAT

TATGGTATTTCAACAGCGACTAGAAAAATCCCTTTTAGTTTAAAACATTATATAGAGTGG

CAGATCGGTTATGATGTCCCCATTAAAGATAAAGAA---AAATTTGAGCTCACTACCCTA

AAAGATGAAAAATATCATTTTTTAGGGGCTAATAATAAAGTAAAGACTCTTTATGAATTG

AGCGAAATGATTTATTACGCTAAGCAATTAAGTTTAATCGGT---------TTAGAAAAT

TTAGAAAATACTTTAAAATATTTAGAAAAACAAAAACAATTTATAGAAGATAATTTTATG

ATTACAAGAGAAAGATTTAGATTACATCAATTTGGTGACATGGATTTTGAACTCTCACGC

ATTTCTTATCCTTTGCTCATTCATTCTTTTAATGATAATCAGTTGAGCGAAATCGTTATT

AGAGAACAACAATACGGCTCTAAAACCCAAGCCATG---CTGTATTTTTGCTTTTCTATT

TTGGAGTTAAAAACTGCTACCCCCTTATTAAATAGAACGGCTGCACTCAAAGAACAGGCT

CTTTTAACCATCAATAAAACCAACGCTCTTATGTTTTTAGAAATGCTTAAAATTTTTGGA

CTTTTAAGCCAAGCGCACCATAGCGATGTGTTAAAGATTTTAGAAAAAATACTTCAAAAT

>KH39

GTGAGTTTGATTAAAGTTGACTATGATAAAAAAGTGATTGAGGTTTCTATTCCTTTAACT

------------TCAATTTCAGGCAAAGTGCGTGTGAAGATCAGACATGCCTTTAGCGAT

TATGGTATTTCAACAGCGACTAGAAAAATCCCTTTTAGTTTAAAACATTATGTAGAGTGG

CAAATCGGTTATGATGTCCCCATTAAAGATAAGGAA---AAATTTGAACTCACTGCTTTA

AAAGATAAAAAATATCATTTTTTAGGGGCTAATAATAAAGTAAAAACTCTTTATGAATTG

AGCGAAATGATTTATTACGCTAAGCAATTAGGTTTAATCAGT---------TTAGAAAAT

TTAGAAAATACTTTAAAATATTTAGAAAAACAAAAACAATTTATAGAAGATAATTTTATG

ATTACAAGAGAAAGATTTAGATCGCATCAATTTGGTGGCATGGATTTTGAACTTTCACAC

ATTTCTTATCCTTTACTCATTCATTCTTTTAACGATAATCAATTGAGTGAAATAGTTATT

AGAGAACAACAATATGGCTCTAAGACCCAAGCCATG---CTGTATTTTTGCTTTTCTATT

TTGGAGTTAAAAACCGCTACCCCCTTATTAAATAGAACGGCTGCACTCAAAGAACATGCC

CTTTTGATTGTCCGTCAAACTAACGCTTCCATGTTTTTAGAAATGCTTAAAATTTTTGGA

CTTTTAAGCCAAGTGCACCATAACGATGTGTTAAAGATTTTAGAAAAAATACTTCAAAAT

>MSL190

GTGAGTTTGATTAGGATTGATGATAGTAAAAAAGCGATTGAGGTTTCTATTCCTTTAACT

------------TCAATTTCAGGCAAAGCGCGTGTGAAAATCAGACATGCCTTTAGCGAT

TATGGTATTTCAACAGCGACTAGAAAAATCCCTTTTAGCTTAAAACATTATGTAGAGTGG

CAAATCGGTTATGATGTCCCCATTAAAGATAAAGAA---AAATTTGAGCTCACTACCCTA

AAAGATGAAAAATATCATTTTTTAGGGGCTAATAATAAAGTGAAAACTCTTTATGAATTA

AGCGAAATGATTTATTACGCTAAGCAATTAGGTTTAATCAGT---------TTAGAAAAT

TTAGAAAATACTTTAAAATATTTAGAAAAACAAAAACAATTTATAGAAGATAATTTTATA

------AGAGAAAGATTTAGATTACATCAATTTGGTGGCATGGATTTTGAACTTTCACGC

ATTTCTTATCCCTTACTCATTCATTCTTTTAATGATAATCAATTGAGCGAAATCGTTATT

AGAGAGCAACAATACGGCTCTAAAACCCAAGCCATG---CTGTATTTTTGCTTTTCTATT

TTGGAATTAAAAACCGCTACTCCCTTATTAAATAGAACGGCTGCACTCAAAGAACATGCC

CTTTTAACTATCCATAAAACCAACGCTCTTGTATTTTTAGAAATGCTTAAAATTTTTGGA

CTTTTAAGCCAAGTGCACCATAACGATGTGTTAAAGATTTTAGAAAAAATACTTCAAAAT

>36:3

GTGAGTTTGATTAGGATTGATGATAGTAAAAAAGCGATTGAGGTTTCTATTCCTTTAACT

------------TCAATTTCAGGCAAAGTGCGTGTGAAAATCAGACATACCTTTAGCGAT

TATAGCATTTCAACAGCGACCAGAAAAATCCCTTTTAGTTTAAAACATTATGTAGAGTGG

CAAATCGGTTATGATGTCCCCATTAAAGATAAAGAA---AAATTTAAACTCACTACTTTA

AAAGATGAAAAATATCATTTTTTAGGGGCTAATAATAAAGTAAAGACTCTTTATGAATTG

AGTGAGATAATCTATTACGCTAAGCAATTAAATTTAATCAGT---------TTAGAAAAT

TTAGAAAATACTTTAAAATATTTAGAAAAACAAAAACAATTTATAGAAGATAATTTCACG

ATTACAAGAGAAAGATTTAGATCGCATCAATTTGGTGGCATGGATTTTGAACTTTCACGC

ATTTCTTATCCTTTACTCATTCATTCTTTTAATGATAATCAATTGAGTGAAATCGTTATT

AGGGAACAACAATATGGCTCTAAAACCCAAGCCATG---CTGTATTTTTGCTTTTCTATT

TTGGAGTTAAAAACCGCTACCCCCTTATTAAACAGAACGGCTATGCTCAAAGAGCATGCT

CTTTTGATTATCCATAAAACCAACGCTCTCATGTTTTTAGAAATGCTTAAAATTTTTGGA

CTTTTAAGCCAAGCGCACCATAGCGATGTGTTAAAGATTTTAGAAAAAATACTTCAAAAT

>KH25

GTGAGTTTGATTAAAGTCAGTGATGATAAAAAAGCGATTGAGGTTTCCATTCCTTTAACT

------------TCAATTTCAGGCAAAGTGCGTGTGAAAATCAGACATGCCTTTAGCGAT

TATGGTCTTTCAACAGCGACTAGAAAAATCCCTTTTAGTTTAAAACATTATATAGAGTGG

CAGATCGGTTATGATGTCCCCATTAAAGATAAAGAA---AAATTTGAACTCACTACCCTA

AAAGATGAAAAATATCATTTTTTAGGGGCTAATAATAAAGTAAAAACCCTTTATGAATTG

AGTGAGATAATCTATTACGCTAAGCAATTAAATTTAATCAGT---------TTAGAAAAT

TTAGAAAATACTTTAAAATATTTAGAAAAACAAAAACAATTTATAGAAGATAATTTCACG

ATTACAAGAGAAAGATTTAGATCGCATCAATTTGGTGGCGTGGATTTTGAACTTTCACGC

ATTTCTTATCCTTTGCTCATTCATTCTTTTAATGATAATCAGTTGAGTAAAATTGTTATT

AGAGAACAACAATATGGCTCTAAAACCCAAGCCATG---CTGTATTTTTGCTTTTCTATT

TTGGAGTTAAAAACCGCTACCCCCTTATTAAACAGAACGGCTGCACTCAAAGAACATGCC

CTTTTAACTATCCATAAAACCAACGCTCCCATGTTTTTAGAAATGCTTAAAATTTTTGGA

CTTTTAAGCCAAGCGCACCATAACGATGTGTTAAAGATTTTAGAAAAAATACTTCAAAAT

>638

GTGAGTTTGATTAAAATTAACCATGATGAAAAAGTGATTGAGGTTTCTATTCCTTTAACT

------------TCAAATTCAGGCAAAGTGCGTGTTAAAATCAGACATGCCTTTAGCGAT

TATGGTATTTCAACAGCGACTAGAAAAATCCCTTTTAGTTTAAAACATTATGTAGAGTGG

CAGATCGGTTATGATGTCCCCATTAAAGATAAAGAA---AAATTTGAACTCACTACTTTA

AAAGATGAAAAATATCATTTTTTAGGGGCCAATAATAAAGTGAAAACTCTTTATGAATTG

AGCGAAATGATTTATTACGCTAAGCAATTAAATTTAATCAGT---------TTAGAAAAT

TTAGAAAATACTTTAAAATATTTAGAAAAACAAAAACAATTTATAGAAGATAGTTTTATG

ATTACAAGAGAAAGATTTAGATCGCATCAATTTGGGGGCATGGATTTTGAACTCTCACGC

ATTTCTTATCCTTTACTCATTCATTCTTTTAATGATAATCAGTTGAGCGAAATTGTTATT

AGGGAACAACAATACGGCTCTAAAACCCAAGCCATG---CTGTATTTTTGCTTTTCTATT

TTGGAGTTAAAAACCGCTACCCCCTTATTAAACAGAACGGCTATGCTCAAAGAGCATGCC

CTTTTGATTATCCATAAAACCAACGCTCCCATGTTTTTAGAAATGCTTAAAATTTTTGGA

CTTTTAAGCCAAGCACACCATAACGATGTGTTAAAGATTTTAGAAAAAATACTTCAAAAT

>20-A-EK1

GTGAGTTTGATTAAAGTTAGTGGTGATAAAAAAGCGATTGAGGTTTCCATTCCTTTAACT

------------TCAATTTCAGGCAAAGTGCGTGTGAAAATCAGACATGCCTTTAGCGAT

TATGGTATTTCAACAGCGACTAGAAAAATCCCTTTCAGTTTAAAACATTATATAGAGTGG

CAGATCGGTTATGATGTCCCCATTAAAGATAAAGAA---AAATTTGAACTCACTACTTTA

AAAGATGAAAAATATCATTTTTTAGGGGCTAATAATAAAGTGAAAACTCTTTATGAATTG

AGCGAAATGATTTATTACACTAAGCAATTAGGTTTAATCAGT---------TTAGAAAAT

TTAGAAAATACTTTAAAATATTTAGAAAAACAAAAACAATTTATAGAAGATAATTTTATG

ATTACAAGAGAAAGATTTAGATTACATCAATTTGGTGGCATGGATTTTGAACTCTCACGC

ATTTCTTATCCTTTGCTCATTCATTCTTTTAATGATAATCAGTTGAGCGAAATAGTTATT

AGAGAACAACAATATGGCTCTAAAACCCAAGCCATG---CTGTATTTTTGCTTTTCTATT

TTGGAGTTAAAAACCGCTACCCCCTTATTAAACAGAACGGCTATGCTCAAAGAGCATGCT

CTTTTGATTATCCATAAAACCAACGCTCTTGTGTTTTTAGAAATGCTTAAAATTTTTGGG

CTTTTAAGCCAAGCGCACCATAACGGTGTGTTAAAGATTTTAGAAAAAATACTTCAAAAT

>KH0153

GTGAGTTTGATTAAAGTTAGTGGTGATAAAAAAGCGATTGAGGTTTCTATTCCCTTAACT

------------TCAATTTCAGGTAAAGTGCGTGTGAAAATCAGACATGCCTTTAGCGAT

TATGGTGTTTCAACAGCGACTAGAAAAATCCCTTTTAGTTTAAAGCATTATGTAGAGTGG

CAGATCGGTTATGATGTCCCCATTAAAGATAAAGAA---AAATTTGAACTCACTACTTTA

AAAGATGAAAAATATCATTTTTTAGGGGCTAATAATAAAGTAAAAACTCTTTATGAATTG

AGCGAGATCATTTACTATGCCAAGCAATTAGGTTTAATCAGT---------TTAGAAAAT

TTGGAAAATACTTTAAAATATTTAGAAAAACAAAAACAATTTATAGAAGATAATTTTATG

ATTACAAGAGAAAGATTTAGATCGCATCAATTTGGTGGCATGGATTTTGAACTTTCACGC

ATTTCTTATCCTTTACTCATTCATTCTTTTAATGATAATCAATTGAGCGAAATTGTTATT

AGAGAACAACAATATGGTTCTAAAACCCAAGCCATG---CTGTATTTTTGCTTTTCTATT

TTGGAGTTAAAAACCGTTACTCCCTTATTAAACAGAACGGCTATGCACAAAGAACATGCT

CTTTTGATTATCCATAAAACCAACGCTCCCATGTTTTTAGAAATGCTTAAAATTTTTGGA

CTTTTAAGCCAAGCGCACCATAACGATGTGTTAAAGATTTTAGAAAAAATACTTCAAAAT

>D1801426

GTGAGTTTGATTAGGATTGATAATAATAAAAAAGTAATTGGGGTTTCTATTCCTTTAACT

------------TCAATTTCAGGCAAAGTGCGTGTGAAAATCAGACATGCCTTTAGCGAT

TATGGTATTTCAACAGCGACTAGAAAAATCCCTTTTAGTTTAAAACATTATGTAGAGTGG

CAAATCGGTTATGATGTCCCCATTAAAGATAAAGAA---AAATTTGAACTCACTACTTTA

AAAGATAAAAAATATCATTTTTTAGGGGCTAATAATAAAGTAAAAACTCTTTATGAATTG

AGCGAGATTATTTACTATGCCAAGCAATTAGATTTAATCAGT---------TTAGAAAAT

TTAGAAAATACTTTAAAATATTTAGAAAAACAAAAACAATTTATAGAAGATAATTTTATG

ATTACAAGAGAAAGATTTAGATCACATCAATTTGGTGGCATGGATTTTGAACTTTCACAC

ATTTCTTATCCTTTACTCATTCATTCTTTTAATAATAATCAATTGAGCGAAATTGTTATT

AGAGAACAACAATATGGTTCTAAAACCCAAGCTATG---CTGTATTTTTGCTTTTCTATT

TTGGAATTAAAAACCGCTACTCCCTTATTAAACAGAACGGCTACGCTCAAAGAACATGCT

CTTTTGATTATCCATAAAACCAACGCTCCCATGTTTTTAAAAATGCTTAAAATTTTTGGA

CTTTTAAGCCAAGCGCACCATGACGATGTGTTAAAGATTTTAGAAAAAATACTTCAAAAT

>HP14016

GTGAGTTTGATTAAGATTGATAATAATAAAAAAGTAATTGAGATTTCTATTCCTTTAACT

------------TCAATTTCAGGCAAAGCGCGTGTGAAAATCAGACATGCCTTTAGCGAT

TATGGTATTTCAACAGCGACCAGAAAAATCCCTTTTAGTTTAAAACATTATGTAGAGTGG

CAAATCGGTTATGATGTCCCCATTAAAGATAAAGAA---AAATTTGAACTCACTACCCTA

AAAGATGAAAAATATCATTTTTTATGGGCTAATAATAAAGTAAAAACCCTTTATGAATTG

AGTGAGATAATCTATTACGCTAAGCAATTAAATTTAATCAGT---------TTAGAAAAT

TTAGAAAATACTTTAAAATATTTAGAAAAACAAAAACAATTTATAGAAGATAATTTCACG

ATTACAAGAGAAAGATTTAGATTACATCAATTTGGTGGCATGGATTTTGAACTTTCACGC

ATTTCTTATCCTTTACTCATTCATTCTTTCAATGATAATCAATTGAGTGAAATCGTTATT

AGAGAGCAACAATATGGCTCTAAAACCCAAGCCATG---CTGTATTTTTGCTTTTCTATT

TTGGAATTAAAAACCGCTACCCCCTTATTAAATAGAACGGCTACACTCAAAGAACATGCC

CTTTTAACTATCCATAAAACCAACGCTCTTATGTTTTTAGAAATGCTTAAAATTTTTGGA

CTTTTAAGCCAAGTGCACCGTAGCGATGTGTTAAAGATTTTAGAAAAAATACTTCAAAAT

>CC33C

GTGAGTTTGATTAAAGTTAATGATGATAAAAAAGCGATTGAGGTTTCTATTCCTTTAACT

------------TCCATTTCAGGCAAAGTTCGTGTGAAAATCAGACATGCCTTTAGCGAT

TATGGCATTTCAACAGCGACTAGAAAAGTCCCTTTCAGTTTAAAGCATTATGTAGAGTGG

CAAATCGGTTATGATGTCCCCATTAAAGATAAAGAA---AAATTTGAACTCACTACCCTA

AAAGATGAAAAATATCATTTTTTAGGGGCTAATAATAAAATAAAAACCCTTTATGAATTG

AGCGAAATAATTGATTACGCTAAGCGATTGGGTTTAATCAGT---------TTAGAAAAT

TTAGAAAATACTTTAAAATATTTAGAAAAACAAAAACAATTCATAGAAGATAGTTTTATG

ATCACAAGAGAAAGATTTAGATCGCATCAATTTGGTGGCATGGATTTTGAACTTTCACGC

ATCTCTTACCCTTTACTCATTCATTCTTTTAATGATAATCAGTTGAGCGAAATCGTTATT

AGAGAGCAACAATACGGCTCTAAAACCCAAGCCATG---CTATATTTTTGCTTTTCTATT

CTGGAATTAAAAACCGCTACCCCTTTATTAAATAGAACCGCTGCCCTCAAAGAACATGCC

CTTTTAACTATCCATGAAACTAACGCTCTTGTGTTTTTAGAAATGCTTAAAATTTTTGGC

CTTTTAAGCCAAGCGCACCATAACGATGTGTTAAAGATTTTAGAAAAAATACTTGAAAAT

>30950

GTGAGTTTGATTAGGATTGATGATAGTAAAAAAGCGATTGAGGTTTCTATTCCTTTAACT

------------TCAATTTCAGGCAAAGTGCGTGTGAAAATCAGACATGCCTTTAGCGAT

TATGGCATTTCAACAGCGACCAGAAAAATCCCTTTTAGTTTAAAACATTATATAGAGTGG

CAAATCGGTTATGATGTCCCCATTGAAGATAAAGAA---AAATTTGAACTCACTACTTTA

AAAGATGAAAAATATCATTTTTTAGGGGCTAATAATAAAGTAAAAACTCTTTATGAATTG

AGTGAGATAATCTATTACGCTAAGCAATTAAATTTAATCAGT---------TTAGAAAAT

TTAGAAAATACTTTAAAATATTTAGAAAAACAAAAACAATTTATAGAAGATAATTTTATG

ATCACAAGAGAAAGATTTAGATTGCATCAATTTGGTGGCATGGATTTTGAACTCTCACGC

ATTTCTTATCCCTTACTCATTCATTCTTTTAATGATAATCAATTGAGCGAAATCGTTATT

AGAGAGCAACAATATGGTTCTAAAACCCAAGCCATG---CTGTATTTTTGCTTTTCTGTT

TTGGAGTTAAAAACCGCTACCCCCTTATTAAATAGAACCGCTACACTCAAAGAACATGCT

TTTTTGATTATCCATAAAACCAACGCTCTTGTGTTTTTAGAAATGCTTAAAATTTTTGGG

CTTTTAAGCCAAGTGCACCATAACGATGTGTTAAAGATTTTAGAAAAAATACTTCAAAAT

>J182

GTGAGTTTGATTAAAGCTAGTGGTGATAAAAAAGCGATTGAGGTTTCTATTCCTTTAACT

------------TCAATTTCAGGCAAAGTGCGTGTGAAAATCAGACATGCCTTTAGCGAT

TATGGTATTTCAACAGCGACTAGAACAATCCCTTTTAGTTTAAAACATTATGTAGAGTGG

CAGATCGGTTATGATGTCCCCATTAAAGATAAAGAA---AAATTTGAACTCACTACTTTA

AAAGATGAAAAATATCATTTTTTAGGGGCTAATAATAAAGTAAAAACTCTTTATGAATTG

AGCGAAATGATTTATTACGCTAAGCAATTAGGTTTAATCAGT---------TTAGAAAAT

TTAGAAAATACTTTAAAATATTTAGAAAAACAAAAACAATTTATAGAAGATAATTTTATG

ATTACAAGAGAAAGATTTAGATTGCATCAATTTGGTGGCATGGATTTTGAACTCTCACGC

ATTTCTTATCCTTTGCTCATTCATTCTTTTAATGATAATCAGTTGAGCGAAATCGTTATT

AGAGAACAACAATATGGCTCTAAAACCCAAGCCATG---CTGTATTTTTGCTTTTCTATT

TTGGAATTAAAAACCGCTACCCCCTTATTAAATAGAACGGCTATGCTCAAAGAACATGCC

CTTTTAACTATCCATAAAACCAACGCTCCCATGTTTTTAGAAATGCTTAAAATTTTTGGA

CTTTTAAGCCAAGTGCACCATAGCGATGTGTTAAAGATTTTAGAAAAAATACTTCAAAAT

>Nic55-C

GTGAGTTTGATTAAAGTTAATGATGATAAAAAAGCGATTGAGGTTTCTATTCCTTTAACT

------------TCCATTTCAGGCAAAGCGCGTGTGAAAATCAGACATGCCTTTAGCGAT

TATGGCATTTCAACAGCGACCAGAAAAATCCCTTTTAGCTTAAAACATTATGTAGAGTGG

CAAATCGGTTATGATGCCCCCATTAAAGATAAAGAA---AAATTTGAACTCACTACCCTA

AAAGATGAAAAATATCATTTTTTAGGGGCTAATAATAAAGTAAAAACCCTTTATGAATTG

AGCGAAATAATTGATTACGCTAAGCGATTGGGTTTAATCAGT---------TTAGAAAAT

TTAGAAAATACTTTAAAATATTTAGAAAAACAAAAACAATTCATAGAAGATAATTTCACG

ATCACAAGAGAAAGATTTAGATCGCATCAATTTGGGGGCATGGATTTTGAACTTTCACGC

ATTTCTTATCCTTTACTCATTCATTCTTTCAACGATAATCAGTTGAGCGAAATCGTTATT

AGAGAGCAACAATACGGCTCTAAAACCCAAGCCATG---CTGTATTTTTGCTTTTCTATT

CTGGAATTAAAAACCGCTACCCCCTTATTAAATAGAACGGCTGCACTCAAAGAACATGCC

CTTTTAACTATCCATAAAACCAACGCTCTTGTGTTTTTAGAAATGCTTAAAATTTTTGGA

CTTTTAAGCCAAGCGCACCATAACGATGTGTTAAAGATTTTAGAAAAAATACTTGAAAAT

>Nic36-A

GTGAGTTTGATTAAAGTTAATGATGATAAAAAAGCGATTGAGGTTTCTATTCCTTTAACT

------------TCCATTTCAGGCAAAGCGCGTGTGAAAATCAGACATGCCTTTAGCGAT

TATGGCATTTCAACAGCGACCAGAAAAATCCCTTTTAGCTTAAAACATTATGTAGAGTGG

CAAATCGGTTATGATGCCCCCATTAAAGATAAAGAA---AAATTTGAACTCACTACCCTA

AAAGATGAAAAATATCATTTTTTAGGGGCTAATAATAAAGTAAAAACCCTTTATGAATTG

AGCGAAATAATTGATTACGCTAAGCGATTGGGTTTAATCAGT---------TTAGAAAAT

TTAGAAAATACTTTAAAATATTTAGAAAAACAAAAACAATTCATAGAAGATAATTTCACG

ATCACAAGAGAAAGATTTAGATCGCATCAATTTGGGGGCATGGATTTTGAACTTTCACGC

ATTTCTTATCCTTTACTCATTCATTCTTTCAACGATAATCAGTTGAGCGAAATCGTTATT

AGAGAGCAACAATACGGCTCTAAAACCCAAGCCATG---CTGTATTTTTGCTTTTCTATT

CTGGAATTAAAAACCGCTACCCCCTTATTAAATAGAACGGCTGCACTCAAAGAACATGCC

CTTTTAACTATCCATAAAACCAACGCTCTTGTGTTTTTAGAAATGCTTAAAATTTTTGGC

CTTTTAAGCCAAGCACACCATAACGATGTGTTAAAGATTTTAGAAAAAATACTTGAAAAT

>Nic48-A

GTGAGTTTGATTAAAGTTAATGATGATAAAAAAGCGATTGAGGTTTCTATTCCTTTAACT

------------TCCATTTCAGGCAAAGCGCGTGTGAAAATCAGACATGCCTTTAGCGAT

TATGGCATTTCAACAGCGACCAGAAAAATCCCTTTTAGCTTAAAACATTATGTAGAGTGG

CAAATCGGTTATGATGCCCCCATTAAAGATAAAGAA---AAATTTGAACTCACTACCCTA

AAAGATGAAAAATATCATTTTTTAGGGGCTAATAATAAAGTAAAAACCCTTTATGAATTG

AGCGAAATAATTGATTACGCTAAGCGATTGGGTTTAATCAGT---------TTAGAAAAT

TTAGAAAATACTTTAAAATATTTAGAAAAACAAAAACAATTCATAGAAGATAATTTCACG

ATCACAAGAGAAAGATTTAGATCGCATCAATTTGGGGGCATGGATTTTGAACTTTCACGC

ATTTCTTATCCTTTACTCATTCATTCTTTCAACGATAATCAGTTGAGCGAAATCGTTATT

AGAGAGCAACAATACGGCTCTAAAACCCAAGCCATG---CTGTATTTTTGCTTTTCTATT

CTGGAATTAAAAACCGCTACCCCCTTATTAAATAGAACGGCTGCACTCAAAGAACATGCC

CTTTTAACTATCCATAAAACCAACGCTCTTGTGTTTTTAGAAATGCTTAAAATTTTTGGA

CTTTTAAGCCAAGCGCACCATAACGATGTGTTAAAGATTTTAGAAAAAATACTTGAAAAT

>B126

GTGAGTTTGATTAAAGTTAATGATGATAAAAAAGCGATTGAGGTTTCTATTCCTTTAACT

------------TCCATTTCAGGCAAAGCGCGTGTGAAAATCAGACATGCCTTTAGCGAT

TATGGTATTTCAACAGCGACTAGAAAAATCCCTTTTAGCTTAAAACATTATGTAGAGTGG

CAAATCGGTTATGATGTCCCCATTAAAGATAAAGAA---AAATTTGAGCTCACTACCCTA

AAAGATGAAAAATATCATTTTTTAGGGGCTAATAATAAAGTAAAAACCCTTTATGAATTG

AGTGAGATAATCTATTACGCTAAGCAATTAAATTTAATCAGT---------TTAGAAAAT

TTAGAAAATACTTTAAAATATTTAGAAAAACAAAAACAATTTATAGAAGATAATTTTATG

ATTACAAGAGAAAGATTTAGATTACATCAATTTGGGGGCATGGATTTTGAACTTTCACGC

ATTTCTTATCCTTTACTCATTCATTCTTTTGATGATAATCAATTGAGCGAAATCGTTATT

AGAGAGCAACAATACGGCTCTAAAACCCAAGCCATG---CTGTATTTTTGCTTTTCTATT

TTGGAGTTAAAAACCGCTACTCCCTTATTAAATAGAACCGCTACACTCAAAGAATATGCT

TTTTTAACTATCCATAAAACCAACGTTCTTATGTTTTTAGAAATGCTTAAAATTTTTGGA

CTTTTAAGCCAAGCGCACCATAACGATGTGTTAAAGATTTTAGAAAAAATACTTGAAAAT

>CHL8

GTGAGTTTGATTAAAGTTAGTGGTGATAAAAAAGCGATTGAGGTTTCTATTCCCTTAACT

------------TCAATTTCAGGTAAAGTGCGTGTGAAAATCAGACATGCCTTTAGCGAT

TATGGTGTTTCAACAGCGACTAGAAAAATCCCTTTTAGTTTAAAACATTATGTAGAGTGG

CAAATCGGTTATGATGTCCCCATTAAAGATAAAGAA---AAATTTGAACTCACTACTTTA

AAAGATGAAAAATATCATTTTTTAGGGGCTAATAGTAAAACAAAAACTCTTTATGAATTG

AGCGAGATCATTTACTATGCCAAGCAATTAGATTTAATCAGT---------TTAGAAAAT

TTAGAAAATACTTTAAAATATTTAGAAAAACAAAAACAATTTATAGAAGATAATTTTATG

ATTACAAGAGAAAGATTTAGATCGCATCAATTTGGTGGCATGGATTTTGAACTTTCACGC

ATTTCTTATCCTTTACTCATTCATTCTTTTAATGATAATCAATTGAGCGAAATTGTTATT

AGAGAACAACAATATGGTTCTAAAACCCAAGCCATG---CTGTATTTTTGCTTTTCTATT

TTGGAATTAAAAACCGCTACTCCCTTATTAAACAGAACGGCTACGCTCAAAGAACATGCT

CTTTTGATTATCCATAAAACTAACGCCCTCATGTTCTTAAAAATGCTTAAAATTTTTGGA

CTTTTAAGCCAAGCGCACCATGACGATGTGTTAAAGATTTTAGAAAAAATACTTCAAAAT

>MHP05

GTGAGTTTGATTAAAGTTAATGATGATAAAAAAGCGATTGAGGTTTCTATTCCTTTAACT

------------TCCATTTCAGGCAAAGCGCGTGTGAAAATCAGGCATGCCTTTAGCGAT

TATGGTATTTCAACAGCGACTAGAAAAATCCCTTTTAGTTTAAAGCATTATGTAGAGTGG

CAAATCGGTTATGATGTCCCCATTGAAGATAAAGAA---AAATTTGAACTCACTACCCTA

AAAGATGAAAAATATCATTTTTTAGGGGCTAATAATAAAGTAAAAACCCTTTATGAATTG

AGCGAAATAATTGATTACGCTAAGCGATTGGGTTTAATCAGT---------TTAGAAAAT

TTAGAAAATACTTTAAAATATTTAGAAAAACAAAAACAATTCATAGAATATAATTTTATG

ATTACAAGAGAAAGATTTAGATCGCATCAATTTGGGGGCATGGATTTTGAACTTTCACGC

ATTTCTTATCCTTTACTCATTCATTCTTTCAACGATAATCAATTAAGTGAAATCGTTATT

AGAGAGCAACAATACGGCTCTAAAACCCAAGCCATG---CTGTATTTTTGCTTTTCTATT

CTGGAATTAAAAACCGCTACCCCTTTATTAAATAGAACCGCTGCCCTCAAAGAACATGCC

CTTTTAACTATCCATAAAACCAACGCTCTTGTGTTTTTAGAAATGCTTAAAATTTTTGGC

CTTTTAAGCCAAGCACACCATAACGATGTGTTAAAGATTTTAGAAAAAATACTTCAAAAT

>M-Mx-2005-115

GTGAGTTTGATTAAGATTGATGATGATAAAAAAGCAATTGAGGTTTCTATTCCTTTAACT

------------TCAATTTCAGGCAAAGCACGTGTGAAAATCAGACATGCCTTTAGCGAT

TATGGCATTTCAACAGCGACCAGAAAAATCCCTTTTAGTTTAAAGCATTATGTAGAGTGG

CAAATCGGTTATGATGTCCCTATTAAAGATAAAGAA---AAATTTAAACTCACTACTTTA

AAAGATGAAAAATACCATTTTTTAGGGGCCAATAATAAAGTAAAAACTCTTTATGAATTG

AGTGAAATAATTTATTACGCTAAGCAATTAAATTTAATCAGT---------TTAGAAAAT

TTAGAAAATACTTTAAAATATTTAGAAAAACAAAAACAATTCATAGAAGATAGTTTTATG

ATCACAAGAGAAAGATTTAGATCGCATCAATTTGGGGGCATGGATTTTGAACTTTCACGC

ATCTCTTATCCCTTACTCATTCATTCTTTTAATGATAATCAGTTGAGTGAAATCGTTATT

AGAGAGCAACAATACGGCTCTAAAGTCCAAGCCATG---CTGTATTTTTGCTTTTCTATT

TTGGAATTAAAAACCGCTACCCCTTTATTAAATAGAACCGCTACACTCAAAGAACATGCT

TTTTTAACTATCCATAAAACCAACGCTCTTGTGTTTTTAGAAATGCTTAAAATTTTTGGA

CTTTTAAGCCAAGTGCACCATAACGATGTGTTAAAGATTTTAGAAAAAATACTTCAAAAT

>ZH109

GTGAGTTTGATTAAAGTTAGTGGTGATAAAAAAGCGATTGAGGTTTCTATTCCTTTAACT

------------TCAATTTCAGGCAAAGTGCGTGTGAAAATCAGACATGCCTTTAGCGAT

TATGGTATTTCAACAGCGACTAGAAAAATCCCTTTTAGTTTAAAACATTATATAGAGTGG

CAGATCGGTTATGATGTCCCTATTAAAGATAAAGAA---AAATTTGAACTCACTACTTTA

AAAGATGAAAAATATCATTTTTTAGGGGCTAATAATAAAGTAAAAACCCTTTATGAATTG

AGTGAGATAATCTATTACGCTAAGCAATTAAATTTAATCAGT---------TTAGAAAAT

TTAGAAAATACTTTAAAATATTTAGAAAAACAAAAACAATTTATAGAAGATAATTTCACG

ATTACAAGAGAAAGATTTAGATCGCATCAATTTGGTGGCATGGATTTTGAACTTTCACAC

ATTTCTTATCCTTTACTCATTCATTCTTTTAATGATAATCAATTGAGCGAAATCGTTATT

AGAGAGCAACAATATGGCTCTAAAACCCAAGCCATG---CTGTATTTTTGCTTTTCTATT

TTGGAGTTAAAAACCGCTACCCCCTTATTAAATAGAACCGCTACACTCAAAGAACATTCC

CTTTTAACTATCCATAAAACCAACGCTCTTATGTTTTTAGAAATGCTTAAAATTTTTGGA

CTTTTAAGTCAAGTGCACCATAACGATGTGTTAAAGATTTTAGAAAAAATACTTCAAAAT

>2004-42

GTGAGTTTGATTAGGATTGATGATAGTAAAAAAGCGATTGAGGTTTCTATTCCTTTAACT

------------TCAATTTCAGGCAAAGTGCGTGTGAAAATCAGACATGCCTTTAGCGAT

TATGGCATTTCAACAGCGACTAGAAAAATCCCTTTTAGTTTAAAGCATTATGTAGAGTGG

CAAATCGGTTATGATGTCCCCATTAAAGATAAAGAA---AAATTTGAACTCACTACTTTA

AAAGATGAAAAATATCATTTTTTAGGGGCTAATAATAAAGTAAAAACTCTTTATGAATTA

AGCGAAATAATTGATTACGCTAAGCAATTAAATTTAATCAGT---------TTAGAAAAT

TTAGAAAATACTTTAAAATATTTAGAAAAACAAAAACAATTCATAGAAGATAGTTTCACG

ATTACAAGAGAAAGATTTAGATCGCATCAATTTGGTGGCATGGATTTTGAACTCTCACGC

ATTTCTTATCCTTTACTCATTCATTCTTTTAACGATAATCAATTGAGTGAAATCGTTATT

AGAGAACAACAATACGGCTCTAAAACCCAAGCCATG---CTGTATTTTTGCTTTTCTATT

TTGGAGTTAAAAACCGCTACCCCCTTATTAAACAGAACGGCTATGCTCAAAGAGCATGCT

CTTTTGATTATCCATAAAACCAACGCTCCCATGTTTTTAGAAATGCTTAAAATTTTTGGA

CTTTTAAGCCAAGCGCACCATAACGATGTGTTAAAGATTTTAGAAAAGATACTTCAAAAT

>2004-39

GTGAGTTTGATTAGGATTGATGATAGTAAAAAAGCGATTGAGGTTTCTATTCCTTTAACT

------------TCAATTTCAGGCAAAGTGCGTGTGAAAATCAGACATGCCTTTAGCGAT

TATGGCATTTCAACAGCGACTAGAAAAATCCCTTTTAGTTTAAAGCATTATGTAGAGTGG

CAAATCGGTTATGATGTCCCCATTAAAGATAAAGAA---AAATTTGAACTCACTACTTTA

AAAGATGAAAAATATCATTTTTTAGGGGCTAATAATAAAGTAAAAACTCTTTATGAATTA

AGCGAAATAATTGATTACGCTAAGCAATTAAATTTAATCAGT---------TTAGAAAAT

TTAGAAAATACTTTAAAATATTTAGAAAAACAAAAACAATTCATAGAAGATAGTTTCACG

ATTACAAGAGAAAGATTTAGATCGCATCAATTTGGTGGCATGGATTTTGAACTCTCACGC

ATTTCTTATCCTTTACTCATTCATTCTTTTAACGATAATCAATTGAGTGAAATCGTTATT

AGAGAACAACAATACGGCTCTAAAACCCAAGCCATG---CTGTATTTTTGCTTTTCTATT

TTGGAGTTAAAAACCGCTACCCCCTTATTAAACAGAACGGCTATGCTCAAAGAGCATGCT

CTTTTGATTATCCATAAAACCAACGCTCCCATGTTTTTAGAAATGCTTAAAATTTTTGGA

CTTTTAAGCCAAGCGCACCATAACGATGTGTTAAAGATTTTAGAAAAGATACTTCAAAAT

>2017-78

GTGAGTTTGATTAGGATTGATGATAGTAAAAAAGCGATTGAGGTTTCTATTCCTTTAACT

------------TCAATTTCAGGCAAAGTGCGTGTGAAAATCAGACATGCCTTTAGCGAT

TATGGCATTTCAACAGCGACTAGAAAAATCCCTTTTAGTTTAAAGCATTATGTAGAGTGG

CAAATCGGTTATGATGTCCCCATTAAAGATAAAGAA---AAATTTGAACTCACTACTTTA

AAAGATGAAAAATATCATTTTTTAGGGGCTAATAATAAAGTAAAAACTCTTTATGAATTA

AGCGAAATAATTGATTACGCTAAGCAATTAAATTTAATCAGT---------TTAGAAAAT

TTAGAAAATACTTTAAAATATTTAGAAAAACAAAAACAATTCATAGAAGATAGTTTCACG

ATTACAAGAGAAAGATTTAGATCGCATCAATTTGGTGGCATGGATTTTGAACTCTCACGC

ATTTCTTATCCTTTACTCATTCATTCTTTTAACGATAATCAATTGAGTGAAATCGTTATT

AGAGAACAACAATACGGCTCTAAAACCCAAGCCATG---CTGTATTTTTGCTTTTCTATT

TTGGAGTTAAAAACCGCTACCCCCTTATTAAACAGAACGGCTATGCTCAAAGAGCATGCT

CTTTTGATTATCCATAAAACCAACGCTCCCATGTTTTTAGAAATGCTTAAAATTTTTGGA

CTTTTAAGCCAAGCGCACCATAACGATGTGTTAAAGATTTTAGAAAAGATACTTCAAAAT

>B547F

GTGAGTTTGATTGAGATTGATAATAATAAAAAAGTAATTGAGATTTCTATTCCTTTAACT

------------TTAATTTCAGGCAAAGTGCGTGTGAAAATCAGACATGCCTTTAGCGAT

TATGGTATTTCAACAGCGACTAGAAAAATCCCTTTTAGTTTAAAGCATTATGTAGAGTGG

CAAATCGGTTATGATGTCCCCATTAAAGATAAAGAA---AAATTTGAGCTCACTACCCTA

AAAGATGAAAAATATCATTTTTTAGGGGCTAATAATAAAGTAAAGACTCTTTATGAATTG

AGTGAGATAATCTATTACGCTAAGCAATTAAATTTAATCAGT---------TTAGAAAAT

TTAGAAAATACTTTAAAATATTTAGAAAAACAAAAACAATTTATAGAAGATAATTTTATA

------AGAGAAAGATTTAGATCGCATCAATTTGGTGGCATGGATTTTGAACTCTCACGC

ATTTCTTATCCTTTACTCATTCATTCTTTTAATGATAATCAATTGAGTGAAATCGTTATT

AGAGAACAACAATATGGCTCTAAAACCCAAGCCATG---CTGTATTTTTGCTTTTCTATT

TTGGAGTTAAAAACCGCTACCCCCTTATTAAACAGAACGGCTATGCTCAAAGAGCATGCT

CTTTTGATTATCCATAAAACCAACGCTCTTATGTTTTTAGAAATGCTTAAAATTTTTGGA

CTTTTAAGCCAAGCGCACCATAACGATGTGTTAAAGATTTTAGAAAAAATACTTCAAAAT

>JSS185-B120

GTGAGTTTGATTGAGATTGATAATAATAAAAAAGTAATTGAGATTTCTATTCCTTTAACT

------------TTAATTTCAGGCAAAGTGCGTGTGAAAATCAGACATGCCTTTAGCGAT

TATGGTATTTCAACAGCGACTAGAAAAATCCCTTTTAGTTTAAAGCATTATGTAGAGTGG

CAAATCGGTTATGATGTCCCCATTAAAGATAAAGAA---AAATTTGAGCTCACTACCCTA

AAAGATGAAAAATATCATTTTTTAGGGGCTAATAATAAAGTAAAGACTCTTTATGAATTG

AGTGAGATAATCTATTACGCTAAGCAATTAAATTTAATCAGT---------TTAGAAAAT

TTAGAAAATACTTTAAAATATTTAGAAAAACAAAAACAATTTATAGAAGATAATTTTATA

------AGAGAAAGATTTAGATCGCATCAATTTGGTGGCATGGATTTTGAACTCTCACGC

ATTTCTTATCCTTTACTCATTCATTCTTTTAATGATAATCAATTGAGTGAAATCGTTATT

AGAGAACAACAATATGGCTCTAAAACCCAAGCCATG---CTGTATTTTTGCTTTTCTATT

TTGGAGTTAAAAACCGCTACCCCCTTATTAAACAGAACGGCTATGCTCAAAGAGCATGCT

CTTTTGATTATCCATAAAACCAACGCTCTTATGTTTTTAGAAATGCTTAAAATTTTTGGA

CTTTTAAGCCAAGCGCACCATAACGATGTGTTAAAGATTTTAGAAAAAATACTTCAAAAT

>2029

GTGAGTTTGATTAAAGTTAATGATGATAAAAAAGTAATTGGGGTTTCTATTCCTTTAACT

------------TCAATTTCAGGCAAAGCGCGTGTGAAAATCAGACATGCCTTTAGCGAT

TATGGTATTTCAACAGCGACTAGAAAAATCCCTTTTAGCTTAAAACATTATGTAGAGTGG

CAAATCGGTTATGATGTCCCCATTAAAGATAAAGAA---AAATTTGAGCTCACTACCCTA

AAAGATGAAAAATATCATTTTTTAGGGGCTAATAATAAAGTAAAAACCCTTTATGAATTG

AGTGAGATAATCTATTACGCTAAGCAATTAAATTTAATCAGT---------TTAGAAAAT

TTAGAAAATACTTTAAAATATTTAGAAAAACAAAAACAATTTATAGAAGATAATTTCACG

ATTACAAGAGAAAGATTTAGATCGCATCAATTTGGTGGCATGGATTTTGAACTTTCACGC

ATTTCTTATCCTTTACTCATTCATTCTTTTAATGATAATCAATTGAGCGAAATCGTTATT

AGAGAGCAACAATACGGCTCTAAAGTTCAAGCCATG---CTGTATTTTTGCTTTTCTATT

TTGGAATTAAAAACCGCTACCCCTTTATTAAATAGAACCGCTACACTCAAAGAACATGCT

TTTTTAACTATCCATAAAACCAACGCTCTTATGTTTTTAGAAATGCTTAAAATTTTTGGA

CTTTTAAGCCAAGCGCACCATAGCGATGTGTTAAAGATTTTAGAAAAAATACTTCAAAAT

>MM2005-72

GTGAGTTTGATTAAAGTTAATGATGATAAAAAAGTGATTGAGGTTTCTATTCCTTTAACT

------------TCCATTTCAGGCAAAGTTCGTGTGAAAATTAGGCATGCCTTTAGTGAT

TATAGTATTTCAACAGCGACTAGAAAAATCCCTTTCAGTTTAAAGCATTATGTAGAGTGG

CAAATCGGTTATGATGTCCCCATTAAAGATAAAGAA---AAATTTGAACTCACTACTTTA

AAAGATGAAAAATATCATTTTTTAGGGGCTAATAATAGAGTAAAGACTCTTTATGAATTG

AGCGAAATAATTGATTACGCTAAGCGATTGGGTTTAATCAGT---------TTAGAAAAT

TTAGAAAATACTTTAAAATATTTAGAAAAACAAAAACAATTCATAGAAGATAGTTTTATA

ATTACAAGAGAAAGATTTAGATCGCATCAATTTGGTGGCATGGATTTTGAACTTTCACGC

ATCTCTTATCCTTTACTCATTCATTCTTTTAATGATAATCAGTTGAGCGAAATCGTTATT

AGAGAGCAACAATACGGCTCTAAAACCCAAGCTATG---CTGTATTTTTGCTTTTCTATT

CTGGAATTAAAAACTGCTACCCCTTTATTAAATAGGACCGCTGCCCTCAAAGAACATGCC

CTTTTAACTATCCACAAAACTAACGCTCTTGTGTTTTTAGAAATGCTTAAAATTTTTGGA

CTTTTAAGCCAAGCGCACCACAACGATGTGTTAAAGATTTTAGAAAAAATACTTGAAAAT

>HP04042

GTGAGTTTGATTAAAGTTAGTGGTGATAAAAAAGCGATTGAGGTTTCTATTCCTTTAACT

------------TCAATTTCAGGCAAAGCGCGTGTGAAAATCAGACATGCCTTTAGCGAT

TATGGTATTTCAACAGCGACTAGAAAAATCCCTTTTAGTTTAAAACATTATGTAGAGTGG

CAGATCGGTTATGATGTCCCCATTAAAGATAAAGAA---AAATTGGAGCTCACTACTTTA

AAAGATGAAAAATATCATTTTTTAGGGGCTAATAATAAAGTGAAAACTCTTTATGAATTG

AGCGAAATGATTGATTACGCTAAGCGATTGGGTTTAATCAGT---------TTAGAAAAT

TTAGAAAATACTTTAAAATATTTAGAAAAACAAAAACAATTTATAGAAGATAGTTTTATG

ATCACAAGAGAAAGATTTAGATCGCATCAATTTGGGGGCATGGATTTTGAACTTTCACGC

ATTTCTTATCCTTTACTCATTCATTCTTTTGATGATAATCAATTGAGCGAAATAGTTATT

AGAGAACAACAATATGGCTCTAAAACCCAAGCCATG---CTGTATTTTTGCTTTTCTATT

TTGGAGTTAAAAACCGCTACCCCCTTATTAAACAGAACGGCTATGCTCAAAGAGCATGCC

CTTTTGATTATCCATAAAACCAACGCTCTTATGTTTTTAGAAATGCTTAAAATTTTTGGA

CTTTTAAGCCAAGCGCACCATAGCGATGTGTTAAAGATTTTAGAAAAAATACTTCAAAAT

>HP00192

GTGAGTTTGATTAAAGTTAGTGGTGATAAAAAAGCGATTGAGGTTTCTATTCCTTTAACT

------------TCAATTTCAAGCAAAGTGCGTGTGAAAATCAGACATGCCTTTAGCGAT

TATGGGATTTCAACAGCGACTAGAAAAATCCCTTTTAGTTTAAAACATTATGTAGAGTGG

CAGATCGGTTATGATGTCCCTATCAAAGATAAAGAA---AAATTTGAACTCACTACTTTA

AAAGATGAAAAATATCATTTTTTAGGGGCTAATAATAAAGTAAAAACTCTTTATGAATTA

AGCGAAATGATTGATTACGCTAAGCAATTAGGTTTAATCAGT---------TTAAAAAAT

TTAGAAAATACTTTAAAATATTTAGAAAAACAAAAACAATTCATAGAAGATAGTTTCACG

ATTACAAGAGAAAGATTTAGATCGCATCAATTTGGTGGCATGGATTTTGAACTCTCACGC

ATTTCTTATCCTTTACTCATTCATTCTTTTAACGATAATCAATTGAGTGAAATCGTTATT

AGAGAACAACAATACGGCTCTAAAACCCAAGCCATG---CTGTATTTTTGCTTTTCTATT

TTGGAGTTAAAAACCGCTACTCCCTTATTAAATAGAACGGCTGCACTCAAAGAGCACGCT

CTTTTGATTATCCATAAAACCAACGCTCTTGTGTTTTTAGAAATGCTTAAAATTTTTGGA

CTTTTAAGCCAAGCACACCATAACGATGTGTTAAAGATTTTAGAAAAAATACTTCAAAAT

>37A5

GTGAGTTTGATTAAAATTAACCATGATAAAAAAATGATTGAGGTTTCTATTCCTTTAACT

------------TCAATTTCAGGCAAAGTGCGTGTGAAGATCAGACATGCCTTTAGCGAT

TATGGTATTTCAACAGCGACTAGAAAAATCCCTTTTAGTTTAAAACATTATGTAGAGTGG

CAGATCGGTTATGATGTCCCCATTAAAGATAAGGAA---AAATTTGAGCTCACTACCCTA

AAAGATGAAAAATATCATTTTTTAGGGGCTAATAATAAAGTAAAAACCCTTTATGAATTG

AGTGAGATAATCTATTACGCTAAGCAATTAAATTTAATCAGT---------TTAGAAAAT

TTAGAAAATACTTTAAAATATTTAGAAAAACAAAAACAATTTATAGAAGATAATTTCACG

ATTACAAGAGAAAGATTTAGAACGCATCAATTTGGTGGCATGGATTTTGAACTTTCACGC

ATTTCTTATCCTTTACTCATTCATTCTTTTAATGATAATGAGTTGAGCGAAATAGTTATT

AGAGAACAACAATACGGCTCTAAGACCCAAGCCATG---CTGTATTTTTGCTTTTCTATT

TTGGAGTTAAAAACTGCTACTCCCTTATTAAATAGAACCGCTACGCTCAAAGAGCATGCT

CTTTTGATTATCCATAAAACCAACGCTCTCATGTTTTTAGAAATGCTTAAATTTTTTGGA

CTTTTAAGCCAAGCGCACCATAGCGATGTGTTAAAGATTTTAGAAAAAATACTTCAAAAT

>21-A-EK1

GTGAGTTTGATTAAAGCTAGTGGTGATAAAAAAGCGATTGAGGTTTCTATTCCTTTAACT

------------TCAATTTCAGGCAAAGCGCGTGTGAAAATCAGACATGCCTTTAGCGAT

TATGGTATTTCAACAGCGACTAGAAAAATCCCTTTTAGCTTAAAACATTATGTAGAGTGG

CAGATCGGTTATGATGTCCCCATTAAAGATAAAGAA---AAATTTGAGCTCACTACCCTA

AAAGATGAAAAATATCATTTTTTAGGGGCTAATAATAAAGTAAAAACCCTTTATGAATTA

AGCGAAATGATTTATTACGCTAAGCAATTAAATTTAATCAGT---------TTAGAAAAT

TTAGAAAATACTTTAAAATATTTAGAAAAACAAAAACAATTTATAGAAGATAATTTT---

---ATAAGAGAAAGATTTAGATCGCATCAATTTGGTGGCATGGATTTTGAACTTTCACGC

ATTTCTTATCCCTTACTCATTCATTCTTTTAATGATAATCAGTTGAGCGAAATTGTTATT

AGAGAGCAACAATATGGCTCTAAAACCCAAGCCATG---CTGTATTTTTGCTTTTCTATT

TTGGAATTAAAAACCGCTACCCCTTTATTAAATAGAACGGCTGCACTCAAAGAACATGCC

CTTTTAACTATCCATAAAACCAACGCTCTTATGTTTTTAGAAATGCTTAAAATTTTTGGA

CTTTTAAGCCAAGCACACCATAACGATGTGTTAAAGATTTTAGAAAAAATACTTCAAAAT

>21-F-EK1

GTGAGTTTGATTAAAGCTAGTGGTGATAAAAAAGCGATTGAGGTTTCTATTCCTTTAACT

------------TCAATTTCAGGCAAAGCGCGTGTGAAAATCAGACATGCCTTTAGCGAT

TATGGTATTTCAACAGCGACTAGAAAAATCCCTTTTAGCTTAAAACATTATGTAGAGTGG

CAGATCGGTTATGATGTCCCCATTAAAGATAAAGAA---AAATTTGAGCTCACTACCCTA

AAAGATGAAAAATATCATTTTTTAGGGGCTAATAATAAAGTAAAAACCCTTTATGAATTA

AGCGAAATGATTTATTACGCTAAGCAATTAAATTTAATCAGT---------TTAGAAAAT

TTAGAAAATACTTTAAAATATTTAGAAAAACAAAAACAATTTATAGAAGATAATTTT---

---ATAAGAGAAAGATTTAGATCGCATCAATTTGGTGGCATGGATTTTGAACTTTCACGC

ATTTCTTATCCCTTACTCATTCATTCTTTTAATGATAATCAGTTGAGCGAAATTGTTATT

AGAGAGCAACAATATGGCTCTAAAACCCAAGCCATG---CTGTATTTTTGCTTTTCTATT

TTGGAATTAAAAACCGCTACCCCTTTATTAAATAGAACGGCTGCACTCAAAGAACATGCC

CTTTTAACTATCCATAAAACCAACGCTCTTATGTTTTTAGAAATGCTTAAAATTTTTGGA

CTTTTAAGCCAAGCACACCATAACGATGTGTTAAAGATTTTAGAAAAAATACTTCAAAAT

>MHP40

GTGAGTTTGATTAAAGTTAATGATGATAAAAAAGCGATTGAGGTTTCTATTCCTTTAACT

------------TCAATTTCAGGCAAAGCGCGTGTGAAAATCAGACATGCCTTTAGCGAT

TATGGCATTTCAACAGCGACTAGAAAAATCCCTTTTAGTTTAAAGCATTATGTAGAGTGG

CAAATCGGTTATGATGTCCCCATTAAAGATAAAGAA---AAATTTGAACTCACTACCCTA

AAAGATGAAAAATATCATTTTTTAGGGGCTAATAATAAAATAAAAACCCTTTATGAATTG

AGCGAAATAATTGATTACGCTAAGCAATTAGGTTTAATCAGT---------TTAGAAAAT

TTAGAAAATACTTTAAAATATTTAGAAAAACAAAAACAATTTATAGAAGATAGTTTTATG

ATTACAAGAGAAAGATTTAGATCGCATCAATTTGGTGGCATGGATTTTGAACTCTCACGC

ATCTCTTACCCTTTACTCATTCATTCTTTTAATGATAATCAGTTGAGCGAAATCGTTATT

AGAGAGCAACAATACGGCTCTAAAACCCAAGCCATG---CTGTATTTTTGCTTTTCTATT

CTGGAATTAAAAACCGCTACCCCTTTATTAAATAGAACCGCTGCCCTCAAAGAACATGCT

CTTTTAATTATCCATAAAACCAACGCTCCCATGTTTTTAGAAATGCTTAAAATTTTTGGA

CTTTTAAGCCAAGCGTACCATAACGATGTGTTAAAGATTTTAGAAAAAATACTTCAAAAT

>CM22341

GTGAATTTGATTAAAGTTAATGATGATAAAAAAGCGATTGAGGTTTCTATTCCTTTAACT

------------TCCATTTCAGGCAAAGCGCATGTGAAAATCAGACATGCCTTTAGCGAT

TATGGTATTTCAACAGCGACTAGAAAAATCCCTTTCAGTTTAAAGCATTATGTAGAGTGG

CAAATCGGTTATGATGTCCCCATTAAAGATAAAGAA---AAATTTGAACTCACTACCCTA

AAAGATGAAAAATATCATTTTTTAGGGGCTAATAATAAAGTAAAAACCCTTTATGAATTG

AGTGAGATAATCTATTACGCTAAGCAATTAAATTTAATCAGT---------TTAGAAAAT

TTAGAAAATACTTTAAAATATTTAGAAAAACAAAAACAATTCATAGAAGATAGTTTTATG

ATCACAAGAGAAAGATTTAGATCGCATCAATTTGGGGGCATGGATTTTGAACTTTCACGC

ATCTCTTATCCCTTACTCATTCATTCTTTTAATGATAATCAATTGAGTGAAATCGTTATT

AGAGAGCAACAATATGGCTCTAAAACCCAAGCCATG---CTGTATTTTTGCTTTTCTATT

TTGGAGTTAAAAACCGCTACCCCCTTATTAAATAGAACGGCTGCACTCAAAGAACAGGCT

CTTTTGATTATCCATAAAACCAACGCTCTCATGTTTTTAGAAATGCTTAAAATTTTTGGA

CTTTTAAGCCAAGCGCACCATAACGATGTGTTAAAGATTTTAGAAAAAATACTTCAAAAT

>ZH107

GTGAGTTTGATTAAGATTGATAATGATAAAAAAGTGATTGAGGTTTCTATTCCTTTAACT

------------TCAATTTCAGGCAAAGCGCGTGTGAAAATCAGACATGCCTTTAGCGAT

TATGGCATTTCAACAGCGACCAGAAAAATCCCTTTTAGTTTAAAGCATTATGTAGAGTGG

CAAATCGGTTATGATGTCCCCATTAAAGATAAAGAA---AAATTGGAACTCACTACTTTA

AAAGATGAAAAATATCATTTTTTAGGGGCTAATAATAAAGTAAAGACTCTTTATGAATTG

AGCGAAATGATTGATTGCGCTAAGCGATTAGGTTTAATCGGT---------TTAGAAAAT

TTAGAAAATACTTTAAAATATTTAGAAAAACAAAAACAATTTATAGAAGATAATTTCACG

ATTACAAGAGAAAGATTTAGATCGCATCAATTTGGTGGCGTGGATTTTGAACTTTCACGC

ATCTCTTATCCTTTGCTCATTCATTCTTTTAATGATAATCAATTGAGCGAAATCGTTATT

AGAGAGCAACAATACGACTCTAAAACCCAAGCCATG---CTGTATTTTTGCTTTTCTATT

TTGGAATTAAAAACCGCTACTCCCTTATTAAATAGAACGGCTGCACTCAAAGAACATGCC

CTTTTAACTATCCATAAAACCAACGCTCTTATGTTTTTAGAAATGCTTAAAATTTTTGGA

CTTTTAAGTCAAGTGCACCATAACGATGTGTTAAAGATTTTAGAAAAAATACTTCAAAAT

>ZH140

GTGAGTTTGATTAAGATTGATAATGATAAAAAAGTGATTGAGGTTTCTATTCCTTTAACT

------------TCAATTTCAGGCAAAGCGCGTGTGAAAATCAGACATGCCTTTAGCGAT

TATGGCATTTCAACAGCGACCAGAAAAATCCCTTTTAGTTTAAAGCATTATGTAGAGTGG

CAAATCGGTTATGATGTCCCCATTAAAGATAAAGAA---AAATTGGAACTCACTACTTTA

AAAGATGAAAAATATCATTTTTTAGGGGCTAATAATAAAGTAAAGACTCTTTATGAATTG

AGCGAAATGATTGATTGCGCTAAGCGATTAGGTTTAATCGGT---------TTAGAAAAT

TTAGAAAATACTTTAAAATATTTAGAAAAACAAAAACAATTTATAGAAGATAATTTCACG

ATTACAAGAGAAAGATTTAGATCGCATCAATTTGGTGGCGTGGATTTTGAACTTTCACGC

ATCTCTTATCCTTTGCTCATTCATTCTTTTAATGATAATCAATTGAGCGAAATCGTTATT

AGAGAGCAACAATACGACTCTAAAACCCAAGCCATG---CTGTATTTTTGCTTTTCTATT

TTGGAATTAAAAACCGCTACTCCCTTATTAAATAGAACGGCTGCACTCAAAGAACATGCC

CTTTTAACTATCCATAAAACCAACGCTCTTATGTTTTTAGAAATGCTTAAAATTTTTGGA

CTTTTAAGTCAAGTGCACCATAACGATGTGTTAAAGATTTTAGAAAAAATACTTCAAAAT

>UM163S

GTGAGTTTGATTAAAATTAACCATGATGAAAAAGTGATTGAGGTTTCTATTCCCTTAACT

------------TCAATTTCAGGCAAAGTGCGTGTGAAAATCAGACATGCCTTTAGCGAT

TATGGTATTTCAACAGCGACTAGAACAATCCCTTTTAGTTTAAAACATTATGTGGAGTGG

CAGATCGGTTATGATGTCCCTATTAAAGATAAAGAA---AAATTTGAACTCACTACTTTA

AGAGATGAAAAATATCATTTTTTAGGGGCTAATAATAAAGTAAAAACTCTTTATGAATTG

AGCGAGATCATTTACTATGCCAAGCAATTAGGTTTAATCAGT---------TTAGAAAAT

TTAGAAAATACTTTAAAATATTTAGAAAAACAAAAACAATTTATAGAAGATAATTTTATG

ATTACAAGAGAAAGATTTAGATCGCATCAATTTGGTGGCATGGATTTTGAACTTTCACGC

ATTTCTTATCCTTTACTCATTCATTCTTTTAATGATAATCAATTGAGCGAGATTGTTATT

AGAGAACAACAATATGGTTCTAAAACCCAAGCCATG---CTGTATTTTTGCTTTTCTATT

TTGGAGTTAAAAACCGCTACTCCCTTATTAAATAGAACGGCTACGCCCAAAGAACATGCT

CTTTTGATTATCCATAAAACCAACGCTCCCATGTTTTTAGAAATGCTTAAAATTTTTGGA

CATTTAAGCCAAGCACACCATAACGATGTGTTAAAGATTTTAGAAAAGATACTCCAAAAT

>UM163

GTGAGTTTGATTAAAATTAACCATGATGAAAAAGTGATTGAGGTTTCTATTCCCTTAACT

------------TCAATTTCAGGCAAAGTGCGTGTGAAAATCAGACATGCCTTTAGCGAT

TATGGTATTTCAACAGCGACTAGAACAATCCCTTTTAGTTTAAAACATTATGTGGAGTGG

CAGATCGGTTATGATGTCCCTATTAAAGATAAAGAA---AAATTTGAACTCACTACTTTA

AGAGATGAAAAATATCATTTTTTAGGGGCTAATAATAAAGTAAAAACTCTTTATGAATTG

AGCGAGATCATTTACTATGCCAAGCAATTAGGTTTAATCAGT---------TTAGAAAAT

TTAGAAAATACTTTAAAATATTTAGAAAAACAAAAACAATTTATAGAAGATAATTTTATG

ATTACAAGAGAAAGATTTAGATCGCATCAATTTGGTGGCATGGATTTTGAACTTTCACGC

ATTTCTTATCCTTTACTCATTCATTCTTTTAATGATAATCAATTGAGCGAGATTGTTATT

AGAGAACAACAATATGGTTCTAAAACCCAAGCCATG---CTGTATTTTTGCTTTTCTATT

TTGGAGTTAAAAACCGCTACTCCCTTATTAAATAGAACGGCTACGCCCAAAGAACATGCT

CTTTTGATTATCCATAAAACCAACGCTCCCATGTTTTTAGAAATGCTTAAAATTTTTGGA

CATTTAAGCCAAGCACACCATAACGATGTGTTAAAGATTTTAGAAAAGATACTCCAAAAT

>APR133

GTGAGTTTGATTAAAGTTAGTGGTGATAAAAAAGTGATTGAGATTTCTATTCCTTTAACT

------------TCCATTTCAGGCAAAGCGCGTGTGAAAATCAGGCATGCCTTTAGCGAT

TATGGTATTTCAACAGCGACTAGAAAAATCCTTTTCAGTTTAAAGCATTATGTAGAGTGG

CAGATCGGTTATGATGTCCCCATTAAAGATAAAGAA---AAATTTGAACTCACTACTTTA

AAAGATGAAAAATATCATTTTTTAGGGGCTAATAATAAAGTAAAAACTCTTTATGAATTG

AGCGAAATGATTGATTACGCTAAGCAATTAGGTTTAATCAGT---------TTAGAAAAT

TTAGAAAATACTTTAAAATATTTAGAAAAACAAAAACAATTCATAGAAGATAGTTTTATG

ATCACAAGAGAAAGATTTAGATCGCATCAATTTGGGGGCATGGATTTTGAACTTTCACGC

ATCTCTTACCCTTTACTCATTCATTCTTTTAATGATAATCAGTTGAGTGAAATCGTTATT

AGAGAGCAACAATATGGCTCTAAAGTCCAAGCCATG---CTGTATTTTTGCTTTTCTATT

TTGGAATTAAAAACCGCTACTCCCTTATTAAATAGAACGGCTATGCTCAAAGAACATGCC

CTTTTAACTATCCATAAAACCAACGCTCTTGTGTTTTTAGAAATGCTTAAAATTTTTGGA

CTTTTAAGCCAAGCGCACCATAACGATGTGTTAAAGATTTTAGAAAAAATACTTCAAAAT

>HP15034

GTGAGTTTGATTAAAGTTAATGATGATAAAAAAGCGATTGAGGTTTCTATTCCTTTAACT

------------TCCATTTCAGGCAAAGCGCGTGTGAAAATCAGACACGCCTTTAGCGAT

TATGGTATTTCAACAGCGACTAGAAAAATCCCTTTCAGTTTAAAGCATTATGTAGAGTGG

CAAATCGGTTATGATGTCCCCATTAAAGATAAAGAA---AAATTTGAACTCACTACTTTA

AAAGATGAAAAATATCATTTTTTATGGGCTAATAATAAAGTAAAAACCCTTTATGAATTG

AGCGAAATGATTGATTACGCTAAGCGATTGGGTTTAATCAGT---------TTAGAAAAT

TTAGAAAATACTTTAAAATATTTAGAAAAACAAAAACAATTCATAGAAGATAATTTTATG

ATCACAAGAGAAAGATTCAGATCGCATCAATTTGAGGGCATGGATTTTGAACTTTCACGC

ATTTCTTATCCCTTACTCATTCATTCTCTTAATGATAATCAATTGAGCGAAATCGTTATT

AGAGAGCAACAATACGGCTCTAAAACCCAAGCCATG---CTGTATTTTTGCTTTTCTATT

CTGGAGTTAAAAACCGCTACACCCTTATTAAATAGGACCGCTGCACTCAAAGAACATGCC

CTTTTAACTATCCATAAAACCAACGCTCTTGTGTTTTTAGAAATGCTTAAAATTTTTGGA

CTTTTAAGTCAAGCGCACCATAACGATGTGTTAAAGATTTTAGAAAAAATACTTCAAAAT

>ZH49

GTGAGTTTGATTAAAATTAACCATGATGAAAAAGTGATTGAGGTTTCCATTCCTTTAACT

------------TCAATTTCAGGCAAAGTGCGTGTGAAAATCAGACATGCCTTTAGCGAT

TATGGTCTTTCAACAGCGACTAGAAAAATCCCTTTTAGTTTAAAACATTATGTAGAGTGG

CAGATCGGTTATGATGTCCCCATTAAAGATAAAGAA---AAATTTGAACTCACTACTTTA

AAAGATGAAAAATACCATTTTTTAGGGGCTAATAATAAAGTAAAAACTCTTTATGAATTA

AGTGAAATGATTTATTACGCTAAGCAATTAGATTTAATCAGT---------TTAGAAAAT

TTAGAAAATACTTTAAAATATTTAGAAAAACAAAAACAATTTATAGAAGATAATTTT---

---ACAAGAGAAAGATTTAGATCGCATCAATTTGGTGGCATGGATTTTGAACTCTCACGC

ATTTCTTATCCTTTGCTCATTCATTCTTTTAATGATAATCAGTTGAGCGAAATCGTTATT

AGAGAACAACAATATGGCTCTAAAACCCAAGCCATG---CTGTATTTTTGCTTTTCTATT

CTGGAATTAAAAACCGCTACCCCTTTATTAAATAGAACGGCTGCACTCAAAGAACATGCT

CTTTTGATTATCCATAAAACCAACGCTCTCATGTTTTTAGAAATGCTTAAAATTTTTGGA

CTTTTAAGCCAAGTGCACCATAACGATGTGTTAAAGATTTTAGAAAAAATACTTCAAAAT

>LIM-008

---------------------------AAAAAAGTGATTGAGATTTCCATTCCTTTAACT

------------TCAATTTCAGGCAAAGTGCGTGTGAAAATCAGACATGCCTTTAGCGAT

TATGGTATTTCAACAGCGACTAGAAAAATCCCTTTTAGTTTAAAACATTATATAGAGTGG

CAGATCGGTTATGATGTCCCCATTAAAGATAAAGAA---AAATTTGAGCTCACTACCCTA

AAAGATGAAAAATATCATTTTTTAGGGGCTAATAATAAAGTAAAAACCCTTTATGAATTG

AGCGAAATGATTTATTACGCTAAGCAATTAAGTTTAATCAGT---------TTAGAAAAT

TTAGAAAATACTTTAAAATATTTAGAAAAACAAAAACAATTTATAGAAGATAATTTTATG

ATTACAAGAGAAAGATTTAGATTACATCAATTTGGTGGCATGGATTTTGAACTCTCACGC

ATTTCTTATCCTTTGCTCATTCATTCTTTTAATGATAATCAATTGAGTGAAATCGTTATT

AGAGAGCAACAATATGGCTCTAAAACCCAAGCCATG---CTGTATTTTTGCTTTTCTATT

TTGGAGTTAAAAACCGCTACCCCCTTATTAAATAGAACCGCTACGCTCAAAGAGCATGCC

CTTTTAACTATCCATAAAACCAACGCTCTTATGTTTTTAGAAATGCTTAAAATTTTTGGA

CTTTTAAGCCAAGCGCACCATAGCGATGTGTTAAAGATTTTAGAAAAAATACTTCAAAAT

>B147

GTGAGTTTGATTAAAGTCAGTGATGATAAAAAAGCGATTGAGGTTTCTATTCCTTTAACT

------------TCAATTTCAGGCAAAGTGCGTGTGAAAATCAGACATGCCTTTAGCGAT

TATGGTATTTCAACAGCGACTAGAAAAATCCCTTTTAGTTTAAAGCATTATGTAGAGTGG

CAAATCGGTTATGATGTCCCTATTAAAGATAAAGAA---AAATTGGAGCTCACTACCCTA

AAAGATGAAAAATATCATTTTTTTGGGGCTAATAATAAAGTAAAGACTCTTTATGAATTA

AGCGAAATGATTGATTACGCTAAGCAATTAAATTTAATCAGT---------TTAGAAAAT

TTAGAAAATACTTTAAAATATTTAGAAAAACAAAAACAATTTATAGAAGATAATTTTATG

ATTACAAGAGAAAGATTTAGATTACATCAATTTGGTGGCATGGATTTTGAACTCTCACGC

ATCTCTTATCCTTTACTCATTCATTCTTTTAATGATAATCAGTTGAGCGAAATCGTTATT

AGAGAGCAACAATACGGCTCTAAAACCCAAGCCATG---CTGTATTTTTGCTTTTCTATT

TTGGAGTTAAAAACCGCTACCCCCTTATTAAATAGAACGGCTGCACTCAAAGAACATGCT

CTTTTGATTATCCATAAAACCAACGCTCTTATGTTTTTAGAAATGCTTAAAATTTTTGGA

CTTTTAAGTCAAGTGCACCATAACGATGTGTTAAAGATTTTAGAAAAAATACTTCAAAAT

>B712A

GTGAGTTTGATTAAGATTGATAATAATAAAAAAGCGATTGAGGTTTCTATTCCTTCAACT

------------TCAATTTCAGGCAAAACGCGTGTGAAAATCAGACATGCCTTTAGCGAT

TATGGCATTTCAACAGCGACCAGAAAAATCCCTTTTAGTTTAAAGCATTATGTAGAGTGG

CAAATCGGTTATGATGTCCCTATTAAAGATAAAGAA---AAATTTAAACTCACTACTTTA

AAAGATGAAAAATATCATTTTTTAGGGGCTAATAATAAAGTAAAAACCTTTTATGAATTG

AGTGAGATAATCTATTACGCTAAGCAATTAAATTTAATCAGT---------TTAGAAAAT

TTAGAAAATACTTTAAAATATTTAGAAAAACAAAAACAATTCATAGAAGATAGTTTTATG

ATTACAAGAGAAAGATTTAGATTACATCAATTTGGTGGCATGGATTTTGAACTCTCACGC

ATTTCTTATCCTTTGCTCATTCATTCTTTTAATGATAATGAGTTGAGCGAAATAGTTATT

AGAGAGCAACAATATGGCTCTAAAACCCAAGCCATG---CTGTATTTTTGCTTTTCTATT

TTGGAATTAAAAACCGCTACTCCCTTATTAAACAGAACCGCTACGCTCAAAGAACATGCT

TTTTTAATTATCCATAAAACCAACGCTCTCATGTTTTTAGAAATGCTTAAAATTTTTGGA

CTTTTAAGCCAAGCGCACCATAATGATGTGTTAAAGATTTTAGAAAAAATACTTCAAAAT

>KH0158

GTGAGTTTGATTAAAATTAACCATGATGAAAAAGTGATTGAGGTTTCTATTCCCTTAACT

------------TCAATTTCAGGCAAAGTGCGTGTGAAAATCAGACATGCCTTTAGCGAT

TATGGTGTTTCAACAGCGACTAGAAAAATCCCTTTTAGTTTAAAACATTATGTAGAGTGG

CAGATCGGTTATGATGTCCCCATTAAAGATAAAGAA---AAATTTGAACTCACTACTTTA

AAAGATGAAAAATACCATTTTTTAGGGGCTAATAATAAAGTAAAAACTCTTTATGAATTG

AGCGAAATGATTTATTACGCTAAGCAATTAGGTTTAATCAGT---------TTAGAAAAT

TTAGAAAATACTTTAAAATATTTAGAAAAACAAAAACAATTTATAAAAGATAATTTTATG

ATTACAAGAGAAAGATTTAGATCGCATCAATTTGGTGGCATGGCTTTTGAACTTTCACGC

ATTTCTTATCCTTTACTCATTCATTCTTTTAATGATAATCAATTGAGCGAAATTGTTATT

AGAGAGCAACAATATGGTTCTAAAACCCAAGCCATG---CTGTATTTTTGCTTTTCTATT

TTGGAGTTAAAAACCGCTACTCCCTTATTAAATAGAACGGCTACGCCCAAAGAACATGCT

CTTTTGATTATCCATAAAACCAACGCTCCCATGTTTTTAAAAATGCTTAAAATTTTTGGA

CTTTTAAGCCAAGTGCACCATAACGATGTGTTAAAGATTTTAGAAAAAATACTTCAAAAT

>HP14036

GTGAGTTTGATTAAAGTTAGTGGTGATAAAAAAGCGATTGAGATTTCTATTCCTTTAACT

------------TCAATTTCAAGCAAAGTGCGTGTGAAAATCAGACATGCCTTTAGCGAT

TATGGGATTTCAACAGCGACTAGAAAAATCCCTTTTAGTTTAAAACATTATGTAGAGTGG

CAGATCGGTTATGATGTCCCTATCAAAGATAAAGAA---AAATTTGAACTCACTACTTTA

AAAGATGAAAAATATCATTTTTTAGGGGCTAATAATAAAGTAAAAACTCTTTATGAATTA

AGCGAAATGATTGATTACGCTAAGCAATTAGGTTTAATCAGT---------TTAAAAAAT

TTAGAAAATACTTTAAAATATTTAGAAAAACAAAAACAATTCATAGAAGATAGTTTCACG

ATTACAAGAGAAAGATTTAGATCGCATCAATTTGGTGGCATGGATTTTGAACTCTCACGC

ATTTCTTATCCTTTACTCATTCATTCTTTTAACGATAATCAATTGAGTGAAATCGTTATT

AGAGAACAACAATACGGCTCTAAAACCCAAGCCATG---CTGTATTTTTGCTTTTCTATT

TTGGAGTTAAAAACCGCTACTCCCTTATTAAATAGAACGGCTGCACTCAAAGAGCACGCT

CTTTTGATTATCCATAAAACCAACGCTCTTGTGTTTTTAGAAATGCTTAAAATTTTTGGA

CTTTTAAGCCAAGCACACCATAACGATGTGTTAAAGATTTTAGAAAAAATACTTCAAAAT

>PIMM-FM-UNAM-18CaC1

GTGAGTTTGATTAAGATTGATAATAATAAAAAAGTAATTGGGGTTTCTATTCCTTTAACT

------------TCAATTTCAGGCAAAGCGCGTGTGAAAATCAGACATGCCTTTAGCGAT

TATGGTATTTCAACAGCGACCAGAAAAATCCCTTTTAGTTTAAAGCATTATGTAGAGTGG

CAAATCGGTTATGATGTCCCCATTAAAGATAAAGAA---AAATTGGAACTCACTACCCTA

AAAGATGAAAAATATCATTTTTTAGGGGCTAATAATAAAGTAAAAACCCTTTATGAATTG

AGTGAGATAATCTATTACGCTAAGCAATTAAATTTAATCAGT---------TTAGAAAAT

TTAGAAAATACTTTAAAATATTTAGAAAAACAAAAACAATTTATAGAAGATAATTTCACG

ATTACAAGAGAAAGATTTAGATTACATCAATTTGGTGGCATTGATTTTGAACTCTCACGC

ATTTCTTATCCTTTACTCATTCGTTCTTTCAACGATAATCAGTTGAGCGAAATTATTATT

AGAGAGCAACAATATGGCTCTAAAACCCAAGCCATG---CTGTATTTTTGCTTTTCTATT

TTGGAGTTAAAAACCGCTACTCCCTTATTAAATAGAACGGCTGCACTCAAAGAACATGCC

CTTTTAACCATCAATAAAACCAACGCTCTTGTGTTTTTAGAAATGCTTAAAATTTTTGGA

CTTTTAAGCCAAGCGCACCATAACGATGTGTTAAAGATTTTAGAAAAAATACTTCAAAAT

>PIMM-FM-UNAM-34CaC2

GTGAGTTTGATTAAGATTGATAATAATAAAAAAGTAATTGGGGTTTCTATTCCTTTAACT

------------TCAATTTCAGGCAAAGCGCGTGTGAAAATCAGACATGCCTTTAGCGAT

TATGGTATTTCAACAGCGACCAGAAAAATCCCTTTTAGTTTAAAGCATTATGTAGAGTGG

CAAATCGGTTATGATGTCCCCATTAAAGATAAAGAA---AAATTGGAACTCACTACCCTA

AAAGATGAAAAATATCATTTTTTAGGGGCTAATAATAAAGTAAAAACCCTTTATGAATTG

AGTGAGATAATCTATTACGCTAAGCAATTAAATTTAATCAGT---------TTAGAAAAT

TTAGAAAATACTTTAAAATATTTAGAAAAACAAAAACAATTTATAGAAGATAATTTCACG

ATTACAAGAGAAAGATTTAGATTACATCAATTTGGTGGCATTGATTTTGAACTCTCACGC

ATTTCTTATCCTTTACTCATTCGTTCTTTCAACGATAATCAGTTGAGCGAAATTATTATT

AGAGAGCAACAATATGGCTCTAAAACCCAAGCCATG---CTGTATTTTTGCTTTTCTATT

TTGGAGTTAAAAACCGCTACTCCCTTATTAAATAGAACGGCTGCACTCAAAGAACATGCC

CTTTTAACCATCAATAAAACCAACGCTCTTGTGTTTTTAGAAATGCTTAAAATTTTTGGA

CTTTTAAGCCAAGCGCACCATAACGATGTGTTAAAGATTTTAGAAAAAATACTTCAAAAT

>HP_106

GTGAGTTTGATTAAAGTTAGTGGTGATAAAAAAGTGATTGAGGTTTCTATTCCTTTAACT

------------TCCATTTCAGGCAAAGCGCGTGTGAAAATCAGACATGCCTTTAGCGAT

TATGGTATTTCAACAGCGACTAGAAAAATCCCTTTTAGTTTAAAGCATTATGTAGAGTGG

CAGATCGGTTATGATGTCCCCATTAAAGATAAAGAA---AAATTTGAACTCACTACTTTA

AAAGATGAAAAATATCATTTTTTAGGGGCTAATAATAAAGTAAAAACTCTTTATGAATTG

AGCGAAATGATTTATTACGCTAAGCGATTGGGTTTAATCAGT---------TTAGAAAAT

TTAGAAAATACTTTAAAATTTTTAGAAAAACAAAAACAATTTATAGAAGATAATTTTATG

ATTACAAGAGAAAGATTTAGATCGCATCAATTTGGTGGCATGGATTTTGAACTCTCACGC

ATTTCTTATCCTTTGCTCATTCATTCTTTTGATGATAATGAGTTGAGCGAAATAGTTATT

AAGGAACAACAATATGGCTCTAAAACCCAAGCCATG---CTGTATTTTTGCTTTTCTATT

TTGGAGTTAAAAACCGCTACCCCCTTATTAAACAGAACCGCTATGCCCAAAGAACATGCC

CTTTTGATTATCCATGAAACCAACGCTCTTGTGTTTTTAGAAATGCTTAAAATTTTTGGA

CTTTTAAGCCAAGTGCACCATAACGATGTGTTAAAGATTTTAGAAAAAATACTTCAAAAT

>G4

GTGAGTTTGATTAAAGTTAGTGGTGATAAAAAAGTGATTGAGGTTTCTATTCCTTTAACT

------------TCCATTTCAGGCAAAGCGCGTGTGAAAATCAGACATGCCTTTAGCGAT

TATGGTATTTCAACAGCGACTAGAAAAATCCCTTTTAGTTTAAAGCATTATGTAGAGTGG

CAGATCGGTTATGATGTCCCCATTAAAGATAAAGAA---AAATTTGAACTCACTACTTTA

AAAGATGAAAAATATCATTTTTTAGGGGCTAATAATAAAGTAAAAACTCTTTATGAATTG

AGCGAAATGATTTATTACGCTAAGCGATTGGGTTTAATCAGT---------TTAGAAAAT

TTAGAAAATACTTTAAAATTTTTAGAAAAACAAAAACAATTTATAGAAGATAATTTTATG

ATTACAAGAGAAAGATTTAGATCGCATCAATTTGGTGGCATGGATTTTGAACTCTCACGC

ATTTCTTATCCTTTGCTCATTCATTCTTTTGATGATAATGAGTTGAGCGAAATAGTTATT

AAGGAACAACAATATGGCTCTAAAACCCAAGCCATG---CTGTATTTTTGCTTTTCTATT

TTGGAGTTAAAAACCGCTACCCCCTTATTAAACAGAACCGCTATGCCCAAAGAACATGCC

CTTTTGATTATCCATGAAACCAACGCTCTTGTGTTTTTAGAAATGCTTAAAATTTTTGGA

CTTTTAAGCCAAGTGCACCATAACGATGTGTTAAAGATTTTAGAAAAAATACTTCAAAAT

>HP725g

GTGAGTTTGATTAAAGTTAGTGGTGATAAAAAAGTGATTGAGGTTTCTATTCCTTTAACT

------------TCCATTTCAGGCAAAGCGCGTGTGAAAATCAGACATGCCTTTAGCGAT

TATGGTATTTCAACAGCGACTAGAAAAATCCCTTTTAGTTTAAAGCATTATGTAGAGTGG

CAGATCGGTTATGATGTCCCCATTAAAGATAAAGAA---AAATTTGAACTCACTACTTTA

AAAGATGAAAAATATCATTTTTTAGGGGCTAATAATAAAGTAAAAACTCTTTATGAATTG

AGCGAAATGATTTATTACGCTAAGCGATTGGGTTTAATCAGT---------TTAGAAAAT

TTAGAAAATACTTTAAAATTTTTAGAAAAACAAAAACAATTTATAGAAGATAATTTTATG

ATTACAAGAGAAAGATTTAGATCGCATCAATTTGGTGGCATGGATTTTGAACTCTCACGC

ATTTCTTATCCTTTGCTCATTCATTCTTTTGATGATAATGAGTTGAGCGAAATAGTTATT

AAGGAACAACAATATGGCTCTAAAACCCAAGCCATG---CTGTATTTTTGCTTTTCTATT

TTGGAGTTAAAAACCGCTACCCCCTTATTAAACAGAACCGCTATGCCCAAAGAACATGCC

CTTTTGATTATCCATGAAACCAACGCTCTTGTGTTTTTAGAAATGCTTAAAATTTTTGGA

CTTTTAAGCCAAGTGCACCATAACGATGTGTTAAAGATTTTAGAAAAAATACTTCAAAAT

>HP_PWs

GTGAGTTTGATTAAAGTTAGTGGTGATAAAAAAGTGATTGAGGTTTCTATTCCTTTAACT

------------TCCATTTCAGGCAAAGCGCGTGTGAAAATCAGACATGCCTTTAGCGAT

TATGGTATTTCAACAGCGACTAGAAAAATCCCTTTTAGTTTAAAGCATTATGTAGAGTGG

CAGATCGGTTATGATGTCCCCATTAAAGATAAAGAA---AAATTTGAACTCACTACTTTA

AAAGATGAAAAATATCATTTTTTAGGGGCTAATAATAAAGTAAAAACTCTTTATGAATTG

AGCGAAATGATTTATTACGCTAAGCGATTGGGTTTAATCAGT---------TTAGAAAAT

TTAGAAAATACTTTAAAATTTTTAGAAAAACAAAAACAATTTATAGAAGATAATTTTATG

ATTACAAGAGAAAGATTTAGATCGCATCAATTTGGTGGCATGGATTTTGAACTCTCACGC

ATTTCTTATCCTTTGCTCATTCATTCTTTTGATGATAATGAGTTGAGCGAAATAGTTATT

AAGGAACAACAATATGGCTCTAAAACCCAAGCCATG---CTGTATTTTTGCTTTTCTATT

TTGGAGTTAAAAACCGCTACCCCCTTATTAAACAGAACCGCTATGCCCAAAGAACATGCC

CTTTTGATTATCCATGAAACCAACGCTCTTGTGTTTTTAGAAATGCTTAAAATTTTTGGA

CTTTTAAGCCAAGTGCACCATAACGATGTGTTAAAGATTTTAGAAAAAATACTTCAAAAT

>HP_151

GTGAGTTTGATTAAAGTTAGTGGTGATAAAAAAGTGATTGAGGTTTCTATTCCTTTAACT

------------TCCATTTCAGGCAAAGCGCGTGTGAAAATCAGACATGCCTTTAGCGAT

TATGGTATTTCAACAGCGACTAGAAAAATCCCTTTTAGTTTAAAGCATTATGTAGAGTGG

CAGATCGGTTATGATGTCCCCATTAAAGATAAAGAA---AAATTTGAACTCACTACTTTA

AAAGATGAAAAATATCATTTTTTAGGGGCTAATAATAAAGTAAAAACTCTTTATGAATTG

AGCGAAATGATTTATTACGCTAAGCGATTGGGTTTAATCAGT---------TTAGAAAAT

TTAGAAAATACTTTAAAATTTTTAGAAAAACAAAAACAATTTATAGAAGATAATTTTATG

ATTACAAGAGAAAGATTTAGATCGCATCAATTTGGTGGCATGGATTTTGAACTCTCACGC

ATTTCTTATCCTTTGCTCATTCATTCTTTTGATGATAATGAGTTGAGCGAAATAGTTATT

AAGGAACAACAATATGGCTCTAAAACCCAAGCCATG---CTGTATTTTTGCTTTTCTATT

TTGGAGTTAAAAACCGCTACCCCCTTATTAAACAGAACCGCTATGCCCAAAGAACATGCC

CTTTTGATTATCCATGAAACCAACGCTCTTGTGTTTTTAGAAATGCTTAAAATTTTTGGA

CTTTTAAGCCAAGTGCACCATAACGATGTGTTAAAGATTTTAGAAAAAATACTTCAAAAT

>HP_751

GTGAGTTTGATTAAAGTTAGTGGTGATAAAAAAGTGATTGAGGTTTCTATTCCTTTAACT

------------TCCATTTCAGGCAAAGCGCGTGTGAAAATCAGACATGCCTTTAGCGAT

TATGGTATTTCAACAGCGACTAGAAAAATCCCTTTTAGTTTAAAGCATTATGTAGAGTGG

CAGATCGGTTATGATGTCCCCATTAAAGATAAAGAA---AAATTTGAACTCACTACTTTA

AAAGATGAAAAATATCATTTTTTAGGGGCTAATAATAAAGTAAAAACTCTTTATGAATTG

AGCGAAATGATTTATTACGCTAAGCGATTGGGTTTAATCAGT---------TTAGAAAAT

TTAGAAAATACTTTAAAATTTTTAGAAAAACAAAAACAATTTATAGAAGATAATTTTATG

ATTACAAGAGAAAGATTTAGATCGCATCAATTTGGTGGCATGGATTTTGAACTCTCACGC

ATTTCTTATCCTTTGCTCATTCATTCTTTTGATGATAATGAGTTGAGCGAAATAGTTATT

AAGGAACAACAATATGGCTCTAAAACCCAAGCCATG---CTGTATTTTTGCTTTTCTATT

TTGGAGTTAAAAACCGCTACCCCCTTATTAAACAGAACCGCTATGCCCAAAGAACATGCC

CTTTTGATTATCCATGAAACCAACGCTCTTGTGTTTTTAGAAATGCTTAAAATTTTTGGA

CTTTTAAGCCAAGTGCACCATAACGATGTGTTAAAGATTTTAGAAAAAATACTTCAAAAT

>J99

GTGAGTTTGATTAAAGTTAGTGGTGATAAAAAAGTGATTGAGGTTTCTATTCCTTTAACT

------------TCCATTTCAGGCAAAGCGCGTGTGAAAATCAGACATGCCTTTAGCGAT

TATGGTATTTCAACAGCGACTAGAAAAATCCCTTTTAGTTTAAAGCATTATGTAGAGTGG

CAGATCGGTTATGATGTCCCCATTAAAGATAAAGAA---AAATTTGAACTCACTACTTTA

AAAGATGAAAAATATCATTTTTTAGGGGCTAATAATAAAGTAAAAACTCTTTATGAATTG

AGCGAAATGATTTATTACGCTAAGCGATTGGGTTTAATCAGT---------TTAGAAAAT

TTAGAAAATACTTTAAAATTTTTAGAAAAACAAAAACAATTTATAGAAGATAATTTTATG

ATTACAAGAGAAAGATTTAGATCGCATCAATTTGGTGGCATGGATTTTGAACTCTCACGC

ATTTCTTATCCTTTGCTCATTCATTCTTTTGATGATAATGAGTTGAGCGAAATAGTTATT

AAGGAACAACAATATGGCTCTAAAACCCAAGCCATG---CTGTATTTTTGCTTTTCTATT

TTGGAGTTAAAAACCGCTACCCCCTTATTAAACAGAACCGCTATGCCCAAAGAACATGCC

CTTTTGATTATCCATGAAACCAACGCTCTTGTGTTTTTAGAAATGCTTAAAATTTTTGGA

CTTTTAAGCCAAGTGCACCATAACGATGTGTTAAAGATTTTAGAAAAAATACTTCAAAAT

>FDAARGOS_300

GTGAGTTTGATTAAAGTTAGTGGTGATAAAAAAGTGATTGAGGTTTCTATTCCTTTAACT

------------TCCATTTCAGGCAAAGCGCGTGTGAAAATCAGACATGCCTTTAGCGAT

TATGGTATTTCAACAGCGACTAGAAAAATCCCTTTTAGTTTAAAGCATTATGTAGAGTGG

CAGATCGGTTATGATGTCCCCATTAAAGATAAAGAA---AAATTTGAACTCACTACTTTA

AAAGATGAAAAATATCATTTTTTAGGGGCTAATAATAAAGTAAAAACTCTTTATGAATTG

AGCGAAATGATTTATTACGCTAAGCGATTGGGTTTAATCAGT---------TTAGAAAAT

TTAGAAAATACTTTAAAATTTTTAGAAAAACAAAAACAATTTATAGAAGATAATTTTATG

ATTACAAGAGAAAGATTTAGATCGCATCAATTTGGTGGCATGGATTTTGAACTCTCACGC

ATTTCTTATCCTTTGCTCATTCATTCTTTTGATGATAATGAGTTGAGCGAAATAGTTATT

AAGGAACAACAATATGGCTCTAAAACCCAAGCCATG---CTGTATTTTTGCTTTTCTATT

TTGGAGTTAAAAACCGCTACCCCCTTATTAAACAGAACCGCTATGCCCAAAGAACATGCC

CTTTTGATTATCCATGAAACCAACGCTCTTGTGTTTTTAGAAATGCTTAAAATTTTTGGA

CTTTTAAGCCAAGTGCACCATAACGATGTGTTAAAGATTTTAGAAAAAATACTTCAAAAT

>GC67-HL

GTGAGTTTGATTAAAGTTAATGATGATAAAAAAGCAATTGAGGTTTCTATTCCTTTAACT

------------TCCATTTCAGGTAAGGCGCGTGTGAAAATCAGACATGCCTTTAGCGAT

TATGGCATTTCAACAGCGACTAGAAAAATCCCTTTTAGTTTAAAACATTATGTGGAGTGG

CAAATCGGTTATGATGTCCTCATTAAAGATAAAGAA---AAATTTGAACTCACTACCCTA

AAAGATGAAAAATATCATTTTTTAGGGGCTAATAATAAAGTAAAAACCCTTTATGAATTG

AGCGAAATAATTTATTACGCTAAGCGATTGGGTTTAATCAGT---------TTAGAAAAT

TTAGAAAATACTTTAAAATATTTAGAAAAACAAAAACAATTCATAGAAGATAGTTTTATG

ATCACAAGAGAAAGATTTAGATCGCATCAATTTGGGGGCATGGATTTTGAACTTTCACGC

ATTTCTTATCCTTTACTCATTCATTCTTTTAATGATAATCAGTTGAGCGAAATCGTTATT

AGAGAGCAACAATACGGCTCTAAAACCCAAGCCATG---CTGTATTTTTGCTTTTCTATT

TTGGAATTAAAAACCACTACCCCTTTATTAAATAGAACGGCTGCCCTCAAAGAACATGCT

CTTTTAACTATCCATAAAACTAACGCTCCCATGTTTTTAGAAATGCTTAAAATTTTTGGA

CTTTTAAGCCAAGCGCACCATAGCGATGTGTTAAAGATTTTAGAAAAAATACTTCAAAAT

>HP13061

GTGAGTTTGATTAAAATTAACCATGATGAAAAAGTGATTGGGGTTTCTATTCCTTTAACT

------------TCAATTTCAGGCAAAGTGCGTGTGAAAATCAGACATGCCTTTAGCGAT

TATGGTGTTTCAACAGCGACTAGAACAATCCCTTTTAGTTTAAAGCATTATGTAGAGTGG

CAGATCGGTTATGATGTCCCCATTAAAGATAAAGAA---AAATTTGAACTCACTACTTTA

AAAGATGAAGAATATCATTTTTTAGGGGTTAATAATAAAGTAAAAACTCTTTATGAATTG

AGCGAGATCATTTACTATGCCAAGCAATTAGGTTTAATCAGT---------TTAGAAAAT

TTAGAAAATACTTTAAAATATTTAGAAAAACAAAAACAATTTATAGAAGATAATTTTATG

ATTACAAGAGAAAGATTTAGATCGCATCAATTTGGTGGCATGGATTTTGAACTTTCACAC

ATTTCCTATCCTTTACTCATTCATTCTTTTAATGATAATCAATTGAGCGAAATCGTTATT

AGAGAGCAACAATATGGTTCTAAAACCCAAGCTATG---CTGTATTTTTGCTTTTCTATT

TTGGAATTAAAAACCGCTACTCCCTTATTAAACAGAACGGCTGCACTTAAAGAACATGCT

CTTTTAACTATCCATAAAACCAACGCTCCCATGTTTTTAGAAATGCTTAAAATTTTTGGA

CTTTTAAGCCAAGCGCACCATGACGATGTGTTAAAGATTTTAGAAAAAATACTTCAAAAT

>B373

GTGAGTTTGATTAAGATTGATGATAATAAAAAAGTAATTGGGGTTTCTATTCCTTTAACT

------------TCAATTTCAGGCAAAGTGCGTGTGAAAATCAGACATGCCTTTAGCGAT

TATGGTATTTCAACAGCGACCAGAAAAATCCCTTTTAGTTTAAAGCATTATGTAGAGTGG

CAAATCGGTTATGATGTCCCCATTAAAGATAAAGAA---AAATTTGAACTTACTACTTTA

AAAGATGAAAAATATCATTTTTTAGGGGTTAATAATAAAATAAAAACCCTTTATGAATTG

AGTGAAATGATTTATTACGCTAAGCAATTAGTTTTAATCAGT---------TTAGAAAAT

TTAGAAAATACTTTAAAATATTTAGAAAAACAAAAACAATTTATAGAAGATAATTTTATA

------AGAGAAAGATTTAGATCGCATCAATTTGGGGGCATGGATTTTGAACTCTCACGC

ATTTCTTATCCCTTACTCATTCACTCTTTTAATGATAATCAGTTGGGCGAAATCGTTATT

AGAGAGCAACAATACGGCTCTAAAACCCAAGCCATG---CTGTATTTTTGCTTTTCTATT

TTGGAGTTAAAAACCGCTACCCCCTTATTAAACAGAACGGCTGCACTCAAAGAGCATGCC

CTTTTAACTATCCATAAAACCAACGCTCCCATGTTTTTAGAAATGCTTAAAATTTTTGGA

CTTTTAAGCCAAGCGCACCATAACGATGTGTTAAAGATTTTAGAAAAAATACTTCAAAAT

>SV376_1

GTGAGTTTGATTAGGATTGATGATAGTAAAAAAGCGATTGAGGTTTCTGTTCCTTTAACT

------------TCCATTTCAGGCAAAGTGCGTGTGAAAATCAGACATGCCTTTAGCGAT

TATGGCATTTCAACAGCGACTAGAAAAATCCCTTTTAGTTTAAAACATTATGTAGAGTGG

CAAATCGGTTATGATGTCCCCATTAAAGATAAAGAA---AAATTTGAGCTCACTACCCTA

AAAGATGAAAAATATCATTTTTTAGGGGCTAATAATAAAGTAAAAACCCTTTATGAATTG

AGTGAGATAATCTATTACGCTAAGCAATTAAATTTAATCAGT---------TTAGAAAAT

TTAGAAAATACTTTAAAATATTTAGAAAAACAAAAACAATTTATAGAAGATAATTTTATA

------AGAGAAAGATTTAGATCGCATCAATTTGGGGGCATGGATTTTGAACTCTCACGC

ATTTCTTATCCTTTACTCATTCATTCTTTTAATGATAATCAGTTGAGTGAAATCGTTATT

AGAGAGCAACAATACGGCTCTAAAGTCCAAGCCATG---CTGTATTTTTGCTTTTCTATT

TTGGAATTAAAAACCGCTACCCCTTTATTAAATAGAACCGCTACACTCAAAGAACATGCT

TTTTTAACTATCCATAAAACCAACGCTCTTGTGTTTTTAGAAATGCTTAAAATTTTTGGA

CTTTTAAGCCAAGCGCACCATAACGATGTGTTAAAGATTTTAGAAAAAATACTTCAAAAT

>Nic51-C

GTGAGTTTGATTAAAGTTAGTGGTGATAAAAAAGCGATTGAGGTTTCCATTCCTTTAACT

------------TCCATTTCAGGCAAAGTGCGTGTGAAAATCAGGCATGCCTTTAGCGAT

TATGGTATTTCAACAGCGACCAGAAAAATCCCTTTTAGTTTAAAGCATTATGTAGAGTGG

CAAATCGGTTATGATGTCCCCATTAAAGATAAAGAA---AAATTTGAGCTCACTACCCTA

AAAGATGAAAAATATCATTTTTTAGGGGCTAATAATAAAGTAAAAACCCTTTATGAATTG

AGTGAGATAATCTATTACGCTAAGCAATTAAATTTAATCAGT---------TTAGAAAAT

TTAGAAAATACTTTAAAATATTTAGAAAAACAAAAACAATTCATAGAAGATAGTTTCACG

ATTACAAGAGAAAGATTTAGATTACATCAATTTGGTAGCATGGATTTTGAACTCTCACGC

ATTTCTTATCCTTTGCTCATTCATTCTTTTAATGATAATCAGTTGAGCGAAATCGTTATT

AGAGAGCAACAATACGGCTCTAAAACCCAAGCCATG---CTGTATTTTTGCTTTTCTATT

TTGGAATTAAAAACCACTACCCCCTTATTAAATAGAACGGCTGCACTCAAAGAACATGCC

CTTTTAACTATCCATAAAACCAACGCTCTTATGTTTTTAGAAATGCTTAAAATTTTTGGA

CTTTTAAGCCAAGCACACCATAGCGATGTGTTAAAGATTTTAGAAAAAATACTTCAAAAT

>CHL16

GTGAGTTTGATTAAAATTAACCATGATGAAAAAGTGATTGAAATTTCCATTCCTTTAACT

------------TCAATTTCAGGCAAAGTGCGTGTGAAAATCAGACATGCCTTTAGCGAT

TATGGTGTTTCAACAGCGACTAGAACAATCCCTTTTAGTTTAAAGCATTATGTAGAGTGG

CAAATCGGTTATGATGTCCCCATTAAAGATAAAGAA---AAATTTGAACTCACTACTTTA

AAAGATGAAAAATATCATTTTTTAGGGGCTAATAATAAAGTAAAAACGCTTTATGAATTG

AGCGAGATCATTTACTATGCCAAGCAATTAGGTTTAATCAGT---------TTAGAAAAT

TTAGAAAATACTTTAAAATATTTAGAAAAACAAAAACAATTTATAGAAGATAATTTTATG

ATTACAAGAGAAAGATTTAGATCGCATCAATTTGGTGGCATGGATTTTGAACTTTCACAC

ATTTCTTATCCTTTACTCATTCATTCTTTTAATGATAATCAATTGAGCGAAATTGTTATT

AGAGAACAACAATATGGTTCTAAAACCCAAGCCATG---CTGTATTTTTGCTTTTCTATT

TTGGAATTAAAAACCGCTACTCCCTTATTAAACAGAACGGCTACGCTCAAAGAACATGCT

CTTTTGATTATCCATAAAACCAACGCTCCCATGTTTTTAAAAATGCTTAAAATTTTTGGA

CTTTTAAGTCAAGCGCACCATGACGATGTGTTAAAGATTTTAGAAAAGATACTTCAAAAT

>Hp238

GTGAGTTTGATTAAAATTAACCATGATGAAAAAGTGATTGAAATTTCCATTCCTTTAACT

------------TCAATTTCAGGCAAAGTGCGTGTGAAAATCAGACATGCCTTTAGCGAT

TATGGTGTTTCAACAGCGACTAGAACAATCCCTTTTAGTTTAAAGCATTATGTAGAGTGG

CAAATCGGTTATGATGTCCCCATTAAAGATAAAGAA---AAATTTGAACTCACTACTTTA

AAAGATGAAAAATATCATTTTTTAGGGGCTAATAATAAAGTAAAAACGCTTTATGAATTG

AGCGAGATCATTTACTATGCCAAGCAATTAGGTTTAATCAGT---------TTAGAAAAT

TTAGAAAATACTTTAAAATATTTAGAAAAACAAAAACAATTTATAGAAGATAATTTTATG

ATTACAAGAGAAAGATTTAGATCGCATCAATTTGGTGGCATGGATTTTGAACTTTCACAC

ATTTCTTATCCTTTACTCATTCATTCTTTTAATGATAATCAATTGAGCGAAATTGTTATT

AGAGAACAACAATATGGTTCTAAAACCCAAGCCATG---CTGTATTTTTGCTTTTCTATT

TTGGAATTAAAAACCGCTACTCCCTTATTAAACAGAACGGCTACGCTCAAAGAACATGCT

CTTTTGATTATCCATAAAACCAACGCTCCCATGTTTTTAAAAATGCTTAAAATTTTTGGA

CTTTTAAGCCAAGCGCACCATGACGATGTGTTAAAGATTTTAGAAAAGATACTTCAAAAT

>KH0012

GTGAGTTTGATTAGGATTGATAATAATAAAAAAGTAATTGGGGTTTCTATTCCTTTAACT

------------TCAATTTCAGGCAGAGTGCGTGTGAAAATCAGACATGCCTTTAGCGAT

TATGGTATTTCAACAGCGACTAGAAAAATCCCTTTTAGCTTAAAACATTATGTAGAGTGG

CAAATCGGTTATGATGTCCCCATTAAAGATAAAGAA---AAATTTGAGCTCACTACCCTA

AAAGATGAAAAATATCATTTTTTAGGGGCTAATAATAAAATGAAAACTCTTTATGAATTG

AGTGAAATGATTTATTACGCTAAGCAATTAAATTTAATCAGT---------TTAGAAAAT

TTAGAAAATACTTTAAAATATTTAGAAAAACAAAAACAATTTATAGAAGATAATTTTATA

------AGAGAAAGATTTAGATCGCATCAATTTGGGGGCATGGATTTTGAACTTTCACGC

ATTTCTTATCCCTTACTCATTCATTCTTTTAATGATAATCAATTGAGCGAAATCGTTATT

AGAGAGCAACAATACGGCTCTAAAACCCAAGCCATG---CTGTATTTTTGCTTTTCTATT

TTGGAGTTAAAAACCGCTACCCCCTTATTAAACAGAACGGCTACACTCAAAGAGCATGCT

CTTTTGATTATCCATAAAACCAACGCTCTTGTGTTTTTAGAAATGCTTAAAATTTTTGGA

CTTTTAAGCCAAGTGCACCATAACGATGTGTTAAAGATTTTAGAAAAAATATTTCAAAAT

>ZH23

GTGAGTTTGATTAGGATTGATAATAATAAAAAAGTAATTGGGGTTTCTATTCCTTTAACT

------------TCAATTTCAGGCAAAGTGCGTGTGAAAATCAGACATGCCTTTAGCGAT

TATGGTATTTCAACAGTGACCAGAAAAATCCCTTTTAGCTTAAAACATTATGTAGAGTGG

CAGATCGGTTATGATGTCCCCATTAAAGATAAAGAA---AAATTTGAACTCACTACTTTA

AAAGATGAAAAATATCATTTTTTAGGGGCTAACAATAAAGTAAAAACTCTTTATGAATTG

AGCGAAATGATTGATTACGTTAAGCAATTAGGTTTAATCAGT---------TTAGAAAAT

TTAGAAAATACTTTAAAATATTTAGAAAAACAAAAACAATTTATAGAAGATAATTTTATG

ATTACAAGAGAAAGATTTAGATTACATCAATTTGGTGGCATGGATTTTGAACTCTCATGC

ATTTCTTATCCCTTACTCATTCATTCTTTTAATGATAATCAGTTGAACGAAATTGTTATT

AGAGAACAACAATATGGCTCTAAAACCCAAGCCATG---CTGTATTTTTGCTTTTCTATT

TTGGAGTTAAAAACCGCTACTCCCTTATTAAACAGAACCGCTATGCTCAAAGAGCATGCT

TTTTTAACCATCCATAAAACCAACACTCTCATGTTTTTAGAAATGCTTAAAATTTTTGGG

CTTTTAAGCCAAGCACACTATAGCGATGTGTTAAAGATTTTAGAAAAAATACTTCAAAAT

>ZH24

GTGAGTTTGATTAGGATTGATAATAATAAAAAAGTAATTGGGGTTTCTATTCCTTTAACT

------------TCAATTTCAGGCAAAGTGCGTGTGAAAATCAGACATGCCTTTAGCGAT

TATGGTATTTCAACAGTGACCAGAAAAATCCCTTTTAGCTTAAAACATTATGTAGAGTGG

CAGATCGGTTATGATGTCCCCATTAAAGATAAAGAA---AAATTTGAACTCACTACTTTA

AAAGATGAAAAATATCATTTTTTAGGGGCTAACAATAAAGTAAAAACTCTTTATGAATTG

AGCGAAATGATTGATTACGTTAAGCAATTAGGTTTAATCAGT---------TTAGAAAAT

TTAGAAAATACTTTAAAATATTTAGAAAAACAAAAACAATTTATAGAAGATAATTTTATG

ATTACAAGAGAAAGATTTAGATTACATCAATTTGGTGGCATGGATTTTGAACTCTCATGC

ATTTCTTATCCCTTACTCATTCATTCTTTTAATGATAATCAGTTGAACGAAATTGTTATT

AGAGAACAACAATATGGCTCTAAAACCCAAGCCATG---CTGTATTTTTGCTTTTCTATT

TTGGAGTTAAAAACCGCTACTCCCTTATTAAACAGAACCGCTATGCTCAAAGAGCATGCT

TTTTTAACCATCCATAAAACCAACACTCTCATGTTTTTAGAAATGCTTAAAATTTTTGGG

CTTTTAAGCCAAGCACACTATAGCGATGTGTTAAAGATTTTAGAAAAAATACTTCAAAAT

>HP00152

GTGAGTTTGATTAAAGTTAATGATGATAAAAAAGTAATTGAGGTTTCTATTCCTTTAACT

------------TCCATTTCAGGCAAAGCGTGTGTGAAAATCAGACATCCCTTTAGCGAT

TATGGCATTTCAACAGCGACCAGAAAAATCCCTTTTAGTTTAAAGCATTATGTAGAGTGG

CAAATCGGTTATGATGTCCCCATTAAAGATAAAGAA---AAATTTGAACTCACTACCCTA

AAAGATGAAAAATATCATTTTTTAGGGGCTAATAATAAAGTAAAAACCCTTTATGAATTG

AGCGAAATAATTGATTACGCTAAGCGATTGGGTTTAATCAGT---------TTAGAAAAT

TTAGAAAATACTTTAAAATATTTAGAAAAACAAAAACAATTTATAGAAGATAGTTTTATG

ATCACAAGAGAAAGATTTAGATCGCATCAATTTGGGGGCATGGATTTTGAACTTTCACGC

ATTTCTTATCCTTTACTCATTCATTCTTTCAACGATAATCAATTGAGCGAAATCGTTATT

AGAGAGCAACAATACGGCTCTAAAACCCAAGCCATG---CTGTATTTTTGCTTTTCTATT

TTGGAGTTAAAAACCGCTACCCCTTTATTAAATAGGACCGCTGTCCTCAAAGAACACGCT

CTTTTAACTATCCATAAAACTAACGCTCTTGTGTTTTTAGAAATGCTTAAAATTTTTGGA

CTTTTAAGCCAAGCGCACCATAACGATGTGTTAAAGATTTTAGAAAAAATACTTCAAAAT

>HP01306

GTGAGTTTGATTAAAGTTAATGATGATAAAAAAGTAATTGAGGTTTCTATTCCTTTAACT

------------TCCATTTCAGGCAAAGCGTGTGTGAAAATCAGACATCCCTTTAGCGAT

TATGGCATTTCAACAGCGACCAGAAAAATCCCTTTTAGTTTAAAGCATTATGTAGAGTGG

CAAATCGGTTATGATGTCCCCATTAAAGATAAAGAA---AAATTTGAACTCACTACCCTA

AAAGATGAAAAATATCATTTTTTAGGGGCTAATAATAAAGTAAAAACCCTTTATGAATTG

AGCGAAATAATTGATTACGCTAAGCGATTGGGTTTAATCAGT---------TTAGAAAAT

TTAGAAAATACTTTAAAATATTTAGAAAAACAAAAACAATTTATAGAAGATAGTTTTATG

ATCACAAGAGAAAGATTTAGATCGCATCAATTTGGGGGCATGGATTTTGAACTTTCACGC

ATTTCTTATCCTTTACTCATTCATTCTTTCAACGATAATCAATTGAGCGAAATCGTTATT

AGAGAGCAACAATACGGCTCTAAAACCCAAGCCATG---CTGTATTTTTGCTTTTCTATT

TTGGAGTTAAAAACCGCTACCCCTTTATTAAATAGGACCGCTGTCCTCAAAGAACACGCT

CTTTTAACTATCCATAAAACTAACGCTCTTGTGTTTTTAGAAATGCTTAAAATTTTTGGA

CTTTTAAGCCAAGCGCACCATAACGATGTGTTAAAGATTTTAGAAAAAATACTTCAAAAT

>HPJ099

---------------------------AAAAAAGCGATTGAGGTTTCTATTCCTTTAACT

------------TCAATTTCAGGCAAAGTGCGTGTGAAAATCAGACATGCCTTTAGCGAT

TATGGTATTTCAACAGCGACTAGAAAAATCCCTTTTAGTTTAAAACATTATGTAGAGTGG

CAGATCGGTTATGATGCCCCCATTAAAGATAAAGAA---AAATTTGAACTCACTACTTTA

AAAGATGAAAAATATCATTTTTTAGGGGCTAATAATAAAGTAAAAACTCTTTATGAATTG

AGTGAAATGATTTATTACGCTAAGCAATTAGGTTTAATCAGT---------TTAGAAAAT

TTAGAAAATACTTTAAAATATTTAGAAAAACAAAAACAATTTATAGAAGATAATTTTATG

ATTACAAGAGAAAGATTTAGATCGCATCAATTTGGTGGCATGGATTTTGAACTCTCACGC

ATTTCTTATCCTTTACTCATTCATTCTTTTAGTGATAATCAATTGAGCGAAATAGTTATT

AGAGAGCAACAATACGGCTCTAAAACCCAAGCCATG---CTGTATTTTTGCTTTTCTATT

TTGGAATTAAAAACCGCTACCCCCTTATTAAACAGAACCGCTACACTCAAAGAACATGCT

TTTTTAACCATCCATAAAACCAACGCTCCCATGTTTTTAGAAATGCTTAAAATTTTTGGG

CTTTTAAGCCAAGTGCACCATAACGATGTGTTAAAGATTTTAGAAAAAATACTTCAAAAT

>B657-A4

GTGAGTTTGATTAGGATTGATAATAATAAAAAAGTAATTGGGGTTTCCATTCCTTTAACT

------------TCAATTTCAGGCAAAGCGCGTGTGAAAATCAGACGTGCCTTTAGCGAT

TATGGCATTTCAACAGCGACCAGAAAAATCCCTTTTAGCTTAAAACATTATGTAGAGTGG

CAAATCGGTTATGATGTCCCCATTAAAGATAAAGAA---AAATTTGAACTCACTACTTTA

AAAGATGAAAAATATCATTTTTTAGGGGCTAACAATAAAGTAAAGACTCTTTATGAATTG

AGTGAAATGATTGATTACGCTAAGCGATTAAATTTAATCAGT---------TTAGAAAAT

TTAGAAAATACTTTAAAATATTTAGAAAAACAAAAACAATTTATAGAAGATAGTTTCACG

ATTACAAGAGAAAGATTTAGATCGCATCAATTTGGGGGCATGGGTTTTGAACTCTCACGC

ATTTCTTATCCCTTACTCATTCATTCTTTTAATGATAATCAGTTGGGCGAAATCGTTATT

AGAGAGCAACAATACGGCTCTAAAACCCAAGCCATG---CTGTATTTTTGCTTTTCTATT

TTGGAATTAAAAACCGCTACTCCCTTATTAAATAGAACGGCTATGCTCAAAGAACATGCC

CTTTTAACTATCCATAAAACCAACGCTCTTATGTTTTTAGAAATGTTTAAAATTTTTGGC

CTTTTAAGCCAAGCGCACCATAATGATGTGTTAAAGATTTTAGAAAAAATACTTCAAAAT

>B657-C1

GTGAGTTTGATTAGGATTGATAATAATAAAAAAGTAATTGGGGTTTCCATTCCTTTAACT

------------TCAATTTCAGGCAAAGCGCGTGTGAAAATCAGACGTGCCTTTAGCGAT

TATGGCATTTCAACAGCGACCAGAAAAATCCCTTTTAGCTTAAAACATTATGTAGAGTGG

CAAATCGGTTATGATGTCCCCATTAAAGATAAAGAA---AAATTTGAACTCACTACTTTA

AAAGATGAAAAATATCATTTTTTAGGGGCTAACAATAAAGTAAAGACTCTTTATGAATTG

AGTGAAATGATTGATTACGCTAAGCGATTAAATTTAATCAGT---------TTAGAAAAT

TTAGAAAATACTTTAAAATATTTAGAAAAACAAAAACAATTTATAGAAGATAGTTTCACG

ATTACAAGAGAAAGATTTAGATCGCATCAATTTGGGGGCATGGGTTTTGAACTCTCACGC

ATTTCTTATCCCTTACTCATTCATTCTTTTAATGATAATCAGTTGGGCGAAATCGTTATT

AGAGAGCAACAATACGGCTCTAAAACCCAAGCCATG---CTGTATTTTTGCTTTTCTATT

TTGGAATTAAAAACCGCTACTCCCTTATTAAATAGAACGGCTATGCTCAAAGAACATGCC

CTTTTAACTATCCATAAAACCAACGCTCTTATGTTTTTAGAAATGTTTAAAATTTTTGGC

CTTTTAAGCCAAGCGCACCATAATGATGTGTTAAAGATTTTAGAAAAAATACTTCAAAAT

>B657-A1

GTGAGTTTGATTAGGATTGATAATAATAAAAAAGTAATTGGGGTTTCCATTCCTTTAACT

------------TCAATTTCAGGCAAAGCGCGTGTGAAAATCAGACGTGCCTTTAGCGAT

TATGGCATTTCAACAGCGACCAGAAAAATCCCTTTTAGCTTAAAACATTATGTAGAGTGG

CAAATCGGTTATGATGTCCCCATTAAAGATAAAGAA---AAATTTGAACTCACTACTTTA

AAAGATGAAAAATATCATTTTTTAGGGGCTAACAATAAAGTAAAGACTCTTTATGAATTG

AGTGAAATGATTGATTACGCTAAGCGATTAAATTTAATCAGT---------TTAGAAAAT

TTAGAAAATACTTTAAAATATTTAGAAAAACAAAAACAATTTATAGAAGATAGTTTCACG

ATTACAAGAGAAAGATTTAGATCGCATCAATTTGGGGGCATGGGTTTTGAACTCTCACGC

ATTTCTTATCCCTTACTCATTCATTCTTTTAATGATAATCAGTTGGGCGAAATCGTTATT

AGAGAGCAACAATACGGCTCTAAAACCCAAGCCATG---CTGTATTTTTGCTTTTCTATT

TTGGAATTAAAAACCGCTACTCCCTTATTAAATAGAACGGCTATGCTCAAAGAACATGCC

CTTTTAACTATCCATAAAACCAACGCTCTTATGTTTTTAGAAATGTTTAAAATTTTTGGC

CTTTTAAGCCAAGCGCACCATAATGATGTGTTAAAGATTTTAGAAAAAATACTTCAAAAT

>ZH85

GTGAGTTTGATTAGGATTGATGATAGTAAAAAAGCGATTGAGGTTTCTATTCCTTTAACT

------------TCAATTTCAGGCAAAGCGCGTGTGAAAATCAGACATGCCTTTAGCGAT

TATGGCATTTCAACAGCGACCAGAAAAATCCCTTTTAGTTTAAAACATTATATAGAGTGG

CAAATCGGTTATGATGTCCCCATTAAAGATAAAGAA---AAATTGGAGCTCACTACCCTA

AAAGATGAAAAATATCATTTTTTAGGGGCTAATAATAAAGTAAAAACCCTTTATGAATTG

AGTGAAATAATTGATTACGCTAAGCAATTAAATTTAATCAGT---------TTAGAAAAT

TTAGAAAATACTTTAAAATATTTAGAAAAACAAAAACAATTTATAGAAGATAATTTTATG

ATTACAAGAGAAAGATTTAGATCGCATCAATTTGGTGGCATGGATTTTGAACTCTCACGC

ATTTCTTATCCTTTGCTCATTCATTCTTTTAATGATAATGAGTTGAGCGAAATTGTTATT

AGGGAACAACAATATGGTTCTAATACCCAAGCCATG---CTGTATTTTTGCTTTTCTATT

TTGGAGTTAAAAACCGCTACCCCCTTATTAAACAGAACGGCTATGCTCAAAGAGCATGCC

CTTTTAACTATCCATAAAACCAACGCTCCCATGTTTTTAGAAATGCTTAAAATTTTTGGA

CTTTTAAGCCAAGTGCACCATAACGATGTGTTAAAGATTTTAGAAAAAATACTTCAAAAT

>ZH19

GTGAGTTTGATTAGGATTGATGATAGTAAAAAAGCGATTGAGGTTTCTATTCCTTTAACT

------------TCAATTTCAGGCAAAGCGCGTGTGAAAATCAGACATGCCTTTAGCGAT

TATGGCATTTCAACAGCGACCAGAAAAATCCCTTTTAGTTTAAAACATTATATAGAGTGG

CAAATCGGTTATGATGTCCCCATTAAAGATAAAGAA---AAATTGGAGCTCACTACCCTA

AAAGATGAAAAATATCATTTTTTAGGGGCTAATAATAAAGTAAAAACCCTTTATGAATTG

AGTGAAATAATTGATTACGCTAAGCAATTAAATTTAATCAGT---------TTAGAAAAT

TTAGAAAATACTTTAAAATATTTAGAAAAACAAAAACAATTTATAGAAGATAATTTTATG

ATTACAAGAGAAAGATTTAGATCGCATCAATTTGGTGGCATGGATTTTGAACTCTCACGC

ATTTCTTATCCTTTGCTCATTCATTCTTTTAATGATAATGAGTTGAGCGAAATTGTTATT

AGGGAACAACAATATGGTTCTAATACCCAAGCCATG---CTGTATTTTTGCTTTTCTATT

TTGGAGTTAAAAACCGCTACCCCCTTATTAAACAGAACGGCTATGCTCAAAGAGCATGCC

CTTTTAACTATCCATAAAACCAACGCTCCCATGTTTTTAGAAATGCTTAAAATTTTTGGA

CTTTTAAGCCAAGTGCACCATAACGATGTGTTAAAGATTTTAGAAAAAATACTTCAAAAT

>32

GTGAGTTTGATTAAAGTTAGTGGTGATAAAAAAGCGATTGAGGTTTCTATTCCTTTAACT

------------TCAATTTCAGGCAAAGCACGTGTGAAAATCAGGCATGCCTTTAGCGAT

TATAGCATTTCAACAGCGACTAGAAAAATCCCTTTTAGTTTAAAACATTATGTAGAGTGG

CAAATCGGTTATGATGTCCCCATTAAAGATAAAGAA---AAATTTAAACTCACTACTTTA

AAAGATGAAAAATATCATTTTTTAGGGTCTAATAATAAAATAAAAACACTTTATGAATTG

AGCGAAATGATTTATTACGCTAATCAATTAAATTTAATCAGT---------TTAGAAAAT

TTAGAAAATACTTTAAAATATTTAGAAAAACAAAAACAATTTATAGAAGATAATTTTATG

ATTACAAGAGAAAGATTTAGATTACATCAATTTGGTGGCATGGATTTTGAACTTTCACGC

ATTTCTTACCCTTTACTCATTCATTCTTTTAATGATAATCAATTGAGCGAAATCGTTATT

AGAGAGCAACAATATGGCTCTAAAACCCAAGCCATG---CTGTATTTTTGCTTTTCTATT

TTGGAGTTAAAAACCGCCACCCCCTTATTAAACAGAACCGCTACACTCAAAGAACATGCT

TTTTTAACCATCCATAAAACCAACGCTCTCATGTTTTTAGAAATGCTTAAAATTTTTGGA

CTTTTAAGCCAAGCGCACCATAACGATGTGTTAAAGATTTTAGAAAAAATACTTCAAAAT

>Taiwan-47

GTGAGTTTGATTAAAGTTAGTGGTGATAAAAAAGCGATTGAGGTTTCTATTCCCTTAACT

------------TCAATTTCAGGCAAAGTGCGTGTGAAAATCAGACATGCCTTTAGCGAT

TATGGTGTTTCAACAGCGACTAGAAAAATCCCTTTCAGTTTAAAGCATTATGTGGAGTGG

CAGATCGGTTATGATGTCCCCATTAAAGATAAAGAA---AAATTTGAACTCACTACTTTA

AGAGATGAAAAATATTATTTTTTAGGGGCTAATAATAAAGTAAAAACTCTTTATGAATTG

AGCGAAATGATTTATTACGCCAAGCAATTAGGTTTAATCAGT---------TTAGAAAAT

TTAGAAAATACTTTAAAATATTTAGAAAAACAAAAACAATTTATAGAAGATAATTTTATG

ATTACAAGAGAAAGATTTAGATCGCATCAATTTGGTGGCATGGATTTTGAACTTTCACGC

ATTTCTTATCCTTTACTCATTCATCCTTTTAATGATAATCAATTGAGCGAAATTGTTATT

AGAGAGCAACAATACGGCTCTAAAACCCAAGCCATG---CTGTATTTTTGCTTTTCTATT

TTGGAGTTAAAAACCGCTACTCCCTTATTAAACAGAACGGCTACGCCCAAAGAACATGCT

CTTTTGATTATCCATAAAACCAACGCTCCCATGTTTTTAAAAATGCTTAAAATTTTTGGA

CTTTTAAGCCAAGCACACCATAACGATGTGTTAAAGATTTTAGAAAAGATATTTCAAAAT

>HP15004

GTGAGTTTGATTAAAGTTAGTGGTGATAAAAAAGCGATTGAGGTTTCTATTCCCTTAACT

------------TCAATTTCAGGCAAAGTGCGTGTGAAAATCAGACATGCCTTTAGCGAT

TATGGTGTTTCAACAGCGACTAGAAAAATCCCTTTTAGTTTAAAACATTATGTAGAGTGG

CAAATCGGTTATGATGTCCCCATTAAAGATAAAGAA---AAATTTGAACTCACTACTTTA

AAAGATAAAAAATATCATTTTTTAGGGGCTAATAATAAAGTAAAAACTCTTTATGAATTG

AGCGAGATTATTTACTATGCCAAGCAATTAGATTTAATCAGT---------TTAGAAAAT

TTAGAAAATACTTTAAAATATTTAGAAAAACAAAAACAATTTATAGAAGATAATTTTATG

ATCACAAGAGAAAGATTCAGATCGCATCAATTTGGTGGCATGGCTTTTGAACTCTCACGC

ATTTCTTATCCTTTACTCATTCATTCTTTTAATGATAATCAATTGAGCGAAATTGTTATT

AGAGAACAACAATATGGTTCTAAAACCCAAGCTATG---CTGTATTTTTGCTTTTCTATT

TTGGAATTAAAAACCGCTACTCCCTTATTAAACAGAACGGCTACGCTCAAAGAACATGCT

CTTTTGATTATCTATAAAACCAACGCTCCCATGTTTTTAGAAATGCTTAAAATTTTTGGA

CTTTTAAGCCAAGCGCACCATAACGATGTGTTAAAGATTTTAGAAAAAATACTTCAAAAT

>KH0095

GTGAGTTTGATTAAAGTTAGTGGTGATAAAAAAGCGATTGAGATTTCTATTCCCTTAACT

------------TCAATTTCAGGTAAAGTGCGTGTGAAAATCAGACATGCCTTTAGCGAT

TATGGTGTTTCAACAGCGACTAGAAAAATCCCTTTTAGTTTAAAACATTATGTAGAGTGG

CAAATCGGTTATGATGTCCCCATTAAAGATAAAGAA---AAATTTGAACTCACTACTTTA

AAAGATGAAAAATATCATTTTTTAGGGGCTAATAATAAAGTAAAAACTCTTTATGAATTG

AGCGAGATTATTTACTATGCCAAGCAATTAAGTTTAATCAGT---------TTAGAAAAT

TTAGAAAATACTTTAAAATATTTAGAAAAACAAAAACAATTTATAGAAGATAATTTTATG

ATTACAAGAGAAAGATTTAGATCACATCAATTTGGTGGCATGGATTTTGAACTTTCACGC

ATTTCTTATCCTTTACTCATTCATTCTTTTGATGATAATCAATTGAGCGAAATTGTTATT

AGAGAGCAACAATATGGTTCTAAAACCCAAGCCATG---CTGTATTTTTGCTTTTCTATT

TTGGAGTTAAAAACCGCTACTCCCTTATTAAACAGAACGGCTACGCCCAAAGAACATGCT

CTTTTGATTATTCATAAAACCAACGCTCCCATGTTTTTAAAAATGCTTAAAATTTTTGGA

CTTTTAAGCCAAACGCACCATGACGATGTGTTAAAGATTTTAGAAAAGATACTTCAAAAT

>22327

GTGAGTTTGATTAAAGCTAGTGGTGATAAAAAAGTGATCGAGGTTTCCATTCCTTTAACT

------------TCCATTTCAGGCAAAGTGCGTGTGAAAATCAGACATGCCTTTAGCGAT

TATGGTATTTCAACAGCGACTAGAAAAATCCCTTTTAGTTTAAAGCATTATGTAGAGTGG

CAGATCGGTTATGATGTCCCCATTAAAGATAAAGAA---AAATTTGAACTCACTACTTTA

AAAGATGAAAAATATCATTTTTTAGGGGCTAATAATAAAGTAAAGACTCTTTATGAATTG

AGTGAGATAATCTATTACGCTAAGCAATTAAATTTAATCAGT---------TTAGAAAAT

TTAGAAAATACTTTAAAATATTTAGAAAAACAAAAACAATTTATAGAAGATAATTTCACG

ATTACAAGAGAAAGATTTAGATCGTATCAATTTGGAGGCATGGGTTTTGAACTCTCACGC

ATCTCTTATCCTTTACTCATTCATTCTTTTAATGATAATCAATTGAGCGAAATTGTTATT

AGGGAACAACAATATGGCTCTAAAACCCAAGTCATG---CTGTATTTTTGCTTTTCTATT

TTGGAATTAAAAACCGCTACCCCTTTATTAAATAGAACCGCTACACTCAAAGAACATGCT

TTTTTAACTATCCATAAAACCAACGCTCTTGTGTTTTTAGAAATGCTTAAAATTTTTGGA

CTTTTAAGCCAAGCGCACCATAACGATGTGTTAAAGATTTTAGAAAAAATACTTCAAAAT

>ZH17

GTGAGTTTGATTAAAGTTAGTGGTGATAAAAAAGCGATTAAGGTTTCTATTCCTTTAACT

------------CCAATTTCAGGCAAAGCGCGTGTGAAAATCAGACATGCCTTTAGCGAT

TATGGTATTTCAACAGCGACTAGAAAAATCCCTTTTAGCTTAAAACATTATGTAGAGTGG

CAAATCGGTTATGATGTCCCCATTAAAGATAAAGAA---AAATTTGAACTCACTACTTTA

AAAGATGAAAAATATCATTTTTTAGGGGCTAATAATAAAGCAAAGACTCTTTATGAATTG

AGCGAAATGATTGATTACGCTAAGCAATTAGGTTTAATCAGT---------TTAAAAAAT

TTAGAAAATACTTTAAAATATTTAGAAAAACAAAAACAATTTATAGAAGATAATTTTATG

ATCACAAGAGAAAGATTTAGATCGCATCAATTTGGTGGCATGGATTTTGAACTCTCACGC

ATTTCTTATCCCTTACTCATTCATTCTTTTAATGATAATGAGTTGAGTGAAATCGTTATT

AGAGAGCAACAATATGGCTCTAAAACCCAAGCCATG---CTGTATTTTTGCTTTTCTATT

TTGGAATTAAAAACCGCTACCCCCTTATTAAATAGAACCGCTATGCTCAAAGAGCATGCT

CTTTTGATTATCCATAAAACCAACGCTCTCATGTTTTTAGAAATGCTTAAAATTTTTGGA

CTTTTAAGCCAAGCGCACCATAACGATGTGTTAAAGATTTTAGAAAAAATACTTCAAAAT

>2003-368

GTGAGTTTGATTAAAGTTAATGATGATAAAAAAGTGATTGAGGTTTCTATTCCTTTAACT

------------TCCATTTCAGGCAAAGTTCGTGTGAAAATTAGGCATGCCTTTAGTGAT

TGTGGTATTTCAACAGCGACTAGAAAAATCCCTTTCAGTTTAAAGCATTATGTAGAGTGG

CAAATCGGTTATGATGTCCCCATTAAAGATAAAGAA---AAATTTGAACTCACTACTTTA

AAAGATGAAAAATATCATTTTTTAGGGGCTAATAATAGAGTAAAGACTCTTTATGAATTG

AGCGAAATAATTGATTACGCTAAGCGATTGGGTTTAATCAGT---------TTAGAAAAT

TTAGAAAATACTTTAAAATATTTAGAAAAACAAAAACAATTCATAGAAGATAGTTTTATA

ATTACAAGAGAAAGATTTAGATCGCATCAATTTGGTGGCATGGATTTTGAACTTTCACGC

ATCTCTTATCCTTTACTCATTCATTCTTTTAATGATAATCAGTTGAGCGAAATCGTTATT

AGAGAGCAACAATACGGCTCTAAAACCCAAGCTATG---CTGTATTTTTGCTTTTCTATT

CTGGAATTAAAAACCGCTACTCCCTTATTAAATAGAACGGCTATGCTCAAAGAACATGCT

CTTTTAACTATCCATAAAACCAACGCTCTTGTGTTTTTAGAAATGCTTAAAATTTTTGGC

CTTTTAAGCCAAGCGCACCATAACGATGTGTTAAAGATTTTAGAAAAAATACTTGAAAAT

>22003

GTGAGTTTGATTAAAGTTAATGATGATAAAAAAGCGATTGAGGTTTCTATTCCTTTAACT

------------TCCATTTCAGGCAAAGTGCGTGTGAAAATCAGGCATGCCTTTAGCGAT

TATGGCATTTCAACAGCGACCAGAAAAATCCCTTTTAGCTTAAAGCATTATGTAGAGTGG

CAAATCGGTTATGATGTCCCCATTAAAGATAAAGAA---AAATTTAAACTCACTACTTTA

AAAGATGAAAAATATCATTTTTTAGGGGCTAATAATAAAGTAAAGACTCTTTATGAATTG

AGTGAGATAATCTATTACGCTAAGCAATTAAATTTAATCAGT---------TTAGAAAAT

TTAGAAAATACTTTAAAATATTTAGAAAAACAAAAACAATTTATAGAAGATAATTTTATG

ATTACAAGAGAAAGATTTAGATTACATCAATTTGGTAGCATGGATTTTGAACTTTCACGC

ATTTCTTACCCTTTACTCATTCATTCTTTTAATGATAATCAGTTGAGTGAAATCGTTATT

AGAGAGCAACAATACGGCTCTAAAGTCCAAGCCATG---CTGTATTTTTGCTTTTCTATT

TTGGAATTAAAAACCGCTACCCCTTTATTAAATAGAACCGCTACACTCAAAGAACATGCT

TTTTTAACTATCCATAAAACCAACGCTCTTGTGTTTTTAGAAATGCTTAAAATTTTTGGA

CTTTTAAGCCAAGCGCACCATAACGATGTGTTAAAGATTTTAGAAAAAATACTTGAAAAT

>GC69-HL

GTGAGTTTGATTAGGATTGATGATAGTAAAAAAGCGATTGAGATTTCTATTCCTTTAACT

------------TCAATTTCAGGCAAAGTGCGTGTGAAAATCAGACATGCCTTTAGCGAT

TATGGTATTTCAACAGCGACCAAAAAAATCCCTTTTAGTTTAAAACATTATATAGAGTGG

CAGATCGGTTATGATGTCCCCATTAAAGATAAAGAA---AAATTTGAACTCACTACTTTA

AAAGATGAAAAATATCATTTTTTAGGGGCTAATAATAAAGTAAAGACTCTTTATGAATTG

AGCGAAATGATTTATTACGCTAAGCAATTAGGTTTAATCAGT---------TTAGAAAAT

TTAGAAAATACTTTAAAATATTTAGAAAAACAAAAACAATTTATAGAAGATAATTTTATG

ATTACAAGAGAAAGATTTAGATCGCATCAATTTGGGGGCATGGATTTTGAACTTTCACGC

ATCTCTTATCCTTTACTCATTCATTCTTTTAATGATAATCAGTTGAGCGAAATCGTTATT

AGAGAGCAACAATATGGCTCTAAAACCCAAGCCATG---CTGTATTTTTGCTTTTCTATT

TTGGAGTTAAAAACCGCTACCCCCTTATTAAACAGAACGGCTGCACTCAAAGAACATGCC

CTTTTAACTATCCATAAAACCAACGCTCTTATGTTTTTAGAAATGCTTAAAATTTTTGGA

CTTTTAAGCCAAGCGCACCATAACGAGTGT------------------------------

>E

GTGAGTTTGATTAAAGTTAGTGGTGATAAAAAAGCGATTGAGGTTTCTATTCCCTTAACT

------------TCAATTTCAGGCAAAGTGCGTGTGAAAATCAGACATACCTTTAGCGAT

TATGGTATTTCAACAGCGACTAGAAAAATCCCTTTCAGTTTAAAGCATTATGTGGAGTGG

CAGATCGGTTATGATGTCCCCATTAAAGATAAAGAA---AAATTGGAACTCACTACCCTA

AAAGATGAAAAATATCATTTTTTAGGGGCTAATAATAAAGTAAAAACCCTTTATGAATTG

AGCGAAATAATTGATTACGCTAAGCAATTAGGTTTAATCAGT---------TTAGAAAAT

TTAGAAAATACTTTAAAATATTTAGAAAAACAAAAACAATTTATAGAAGATAATTTCACG

ATTACAAGAGAAAGATTTAGATCGCATCAATTTGGGGGCATGGATTTTGAACTTTCACGC

ATTTCTTATCCTTTACTCATTCATTCTTTTAATGATAATCAATTGAGTGAAATCGTTATT

AGAGAGCAACAATACGGCTCTAAAACCCAAGCCATG---CTGTATTTTTGCTTTTCTATT

TTGGAATTAAAAACCGCTACCCCCTTATTAAACAGAACCACTACACTCAAAGAATATGCT

TTTTTAACCATCCATAAAACCAACGCTCTCATGTTTTTAGAAATGCTTAAAATTTTTGGA

CTTTTAAGCCAAGCGCACCATAACGATGTGTTAAAGATTTTAGAAAAAATACTTCAAAAT

>HP12078

GTGAGTTTGATTAAAATTAACCATGATGAAAAAGTGATTGAGATTTCTATTCCCTTAACT

------------TCAATTTCAGGCAAAGTGCGTGTGAAAATCAGACATGCCTTTAGCGAT

TATGGTGTTTCAACAGCGACTAGAACAATCCCTTTTAGTTTAAAGCATTATGTAGAGTGG

CAAATCGGTTATGATGTCCCCATTAAAGATAAAGAA---AAATTTGAACTCACTACTTTA

AAAGATAAAAAATATCATTTTTTAGGGGCTAATAATAAAGTAAAAACTCTTTATGAATTG

AGCGAGATTATTTACTATGCCAAGCAATTAGATTTAATCAGT---------TTAGAAAAT

TTAGAAAATACTTTAAAATATTTAGAAAAACAAAAACAATTTATAGAAGATAATTTTATG

ATTACAAGAGAAAGATTTAGATCGCATCAATTTGGTGGCATGGATTTTGAACTTTCACAC

ATTTCCTATCCTTTACTCATTCATTCTTTTAATGATAATCAATTGAGCGAAATAGTTATT

AGAGAGCAACAATATGGTTCTAAAACCCAAGCTATG---CTGTATTTTTGCTTTTCTATT

TTGGAGTTAAAAACTGCTACCCCCTTATTAAATAGAACCGCTACGCTCAAAGAACATGCC

CTTTTAACTATCCATAAAACCAACACTCTCATGTTTTTAGAAATGCTTAAAATTTTTGGA

CTTTTAAGCCAAGCGCACCATAGCGATGTGTTAAAGATTTTAGAAAAGATACTTCAA---

>428

GTGAGTTTGATTAAAGTTAGTGGTGATAAAAAAGCGATTGAGGTTTCTATTCCCTTAACT

------------TCAATTTCAGGTAAAGTGCGTGTGAAAATCAGACATGCCTTTAGCGAT

TATGGTGTTTCAACAGCGACTAGAAAAATCCCTTTTAGTTTAAAGCATTATGTAGAGTGG

CAAATCGGTTATGATGTCCCCATTAAAGATAAAGAA---AAATTTGAACTCACTACTTTA

AAAGATGAAAAATATCATTTTTTAGGGGCTAATAGTAAAACAAAAACTCTTTATGAATTG

AGCGAGATCATTTACTATGCCAAGCAATTAGATTTAATCAGT---------TTAGAAAAT

TTAGAAAATACTTTAAAATATTTAGAAAAACAAAAACAATTTATAGAAGATAATTTTATG

ATTACAAGAGAAAGATTTAGATCGCATCAATTTGGTGGCATGGATTTTGAACTTTCACGC

ATTTCTTATCCTTTACTCATTCATTCTTTTAATGATAATCAATTGAGCGAAATTGTTATT

AGAGAACAACAATATGGTTCTAAAACCCAAGCTATG---CTGTATTTTTGCTTTTCTATT

TTGGAATTAAAAACCGCTACTCCCTTATTAAACAGAACGGCTACGCTCAAAGAACATGCT

CTTTTGATTATCCATAAAACCAACGCTCCCATGTTTTTAGAAATGCTTAAAATTTTTGAA

CTTTTAAGCCAAGCGCACCATGACGATGTGTTAAAGATTTTAGAAAAAATACTTCAAAAT

>HP15039

GTGAGTTTGATTAAAGTTAATGATGATAAAAAAGTAATTGAGGTTTCTATTCCTTTAACT

------------TCCATTTCAGGCAAAGCGTGTGTGAAAATCAGACATCCCTTTAGCGAT

TATGGCATTTCAACAGCGACCAGAAAAATCCCTTTTAGTTTAAAGCATTATGTAGAGTGG

CAAATCGGTTATGATGTCCCCATTAAAGATAAAGAA---AAATTTGAACTCACTACCCTA

AAAGATGAAAAATATCATTTTTTAGGGGCTAATAATAAAGTAAAAACCCTTTATGAATTG

AGCGAAATAATTGATTACGCTAAGCGATTGGGTTTAATCAGT---------TTAGAAAAT

TTAGAAAATACTTTAAAATATTTAGAAAAACAAAAACAATTTATAGAAGATAGTTTTATG

ATCACAAGAGAAAGATTTAGATCGCATCAATTTGGGGGCATGGATTTTGAACTTTCACGC

ATTTCTTATCCTTTACTCATTCATTCTTTCAACGATAATCAATTGAGCGAAATCGTTATT

AGAGAGCAACAATACGGCTCTAAAACCCAAGCCATG---CTGTATTTTTGCTTTTCTATT

TTGGAGTTAAAAACCGCTACCCCTTTATTAAATAGGACCGCTGTCCTCAAAGAACACGCT

CTTTTAACTATCCATAAAACTAACGCTCTTGTGTTTTTAGAAATGCTTAAAATTTTTGGA

CTTTTAAGCCAAGTGCACCATAACGATGTGTTAAAGATTTTAGAAAAAATACTTCAAAAT

>UM443S

GTGAGTTTGATTAAAGTTAGTGGTGATAAAAAAGCGATTGAGGTTTCTATTCCCTTAACT

------------TCAATTTCAGGTAAAGTGCGTGTGAAAATCAGACATGCCTTTAGCGAT

TATGGTGTTTCAACAGCGACTAGAAAAATCCCTTTTAGTTTAAAACATTATGTAGAGTGG

CAAATCGGTTATGATGTCCCCATTAAAGATAAAGAA---AAATTTGAACTCACTACTTTA

AAAGATGAAAAATATCATTTTTTAGGGGCTAATAGTAAAACAAAAACTCTTTATGAATTG

AGCGAGATTATTTACTATGCCAAGCAATTAAGTTTAATCAGT---------TTAGAAAAT

TTAGAAAATACTTTAAAATATTTAGAAAAACAAAAACAATTTATAGAAGATAATTTTATG

ATTACAAGAGAAAGATTTAGATCACATCAATTTGGTGGCATGGACTTTGAACTTTCACGC

ATTTCTTATCCTTTACTCATTCATTCTTTTGATGATAATCAATTGAGCGAAATTGTTATT

AGAGAGCAACAATATGGTTCTAAAACCCAAGCCATG---CTGTATTTTTGCTTTTCTATT

TTGGAGTTAAAAACCGCTACTCCCTTATTAAACAGAACGGCTACGCCCAAAGAACATGCT

CTTTTGATTATTCATAAAACCAACGCTCCCATGTTTTTAAAAATGCTTAAAATTTTTGGA

CTTTTAAGCCAAACGCACCATGACGATGTGTTAAAGATTTTAGAAAAGATACTTCAAAAT

>UM443R

GTGAGTTTGATTAAAGTTAGTGGTGATAAAAAAGCGATTGAGGTTTCTATTCCCTTAACT

------------TCAATTTCAGGTAAAGTGCGTGTGAAAATCAGACATGCCTTTAGCGAT

TATGGTGTTTCAACAGCGACTAGAAAAATCCCTTTTAGTTTAAAACATTATGTAGAGTGG

CAAATCGGTTATGATGTCCCCATTAAAGATAAAGAA---AAATTTGAACTCACTACTTTA

AAAGATGAAAAATATCATTTTTTAGGGGCTAATAGTAAAACAAAAACTCTTTATGAATTG

AGCGAGATTATTTACTATGCCAAGCAATTAAGTTTAATCAGT---------TTAGAAAAT

TTAGAAAATACTTTAAAATATTTAGAAAAACAAAAACAATTTATAGAAGATAATTTTATG

ATTACAAGAGAAAGATTTAGATCACATCAATTTGGTGGCATGGACTTTGAACTTTCACGC

ATTTCTTATCCTTTACTCATTCATTCTTTTGATGATAATCAATTGAGCGAAATTGTTATT

AGAGAGCAACAATATGGTTCTAAAACCCAAGCCATG---CTGTATTTTTGCTTTTCTATT

TTGGAGTTAAAAACCGCTACTCCCTTATTAAACAGAACGGCTACGCCCAAAGAACATGCT

CTTTTGATTATTCATAAAACCAACGCTCCCATGTTTTTAAAAATGCTTAAAATTTTTGGA

CTTTTAAGCCAAACGCACCATGACGATGTGTTAAAGATTTTAGAAAAGATACTTCAAAAT

>HP15051

GTGAGTTTGATTAAAATTAACCATGATAAAAAAGTGATTGAAACTTCCATTCCTTTAACT

------------TCAATTTCAGGCAAAGTGCGTGTGAAAATCAGACATGCCTTTAGCGAT

TATGGTATTTCAACAGCGACTAGAACAATCCCTTTTAGTTTAAAACATTATGTAGAGTGG

CAGATCGGTTATGATGTCCCCATTAAAGATAAAGAA---AAATTTGAACTCACTACTTTA

AAAGATGAAAAATATCATTTTTTAGGGGCTAATAATAAAGTAAAAACTCTTTATGAATTG

AGTGAGATTATTTACTATGCCAAGCAATTAGATTTAATCAGT---------TTAGAAAAT

TTAGAAAATACTTTAAAATATTTAGAAAAACAAAAACAATTTATAGAAGATAATTTTATG

ATTACAAGAGAAAGATTTAGATCACATCAATTTGGTGGCATGGATTTTGAACTTTCACAC

ATTTCTTATCCTTTACTCATTCATTCTTTTAATGATAATCAATTGAGCGAAATTGTTATT

AGAGAACAACAATATGGTTCTAAAACCCAAGCTATG---CTGTATTTTTGCTTTTCTATT

TTGGAATTAAAAACCGCTCCTCCCTTATTAAACAGAACGGCTACGCTCAAAGAACATGCT

CTTTTGATTATCCATAAAACCAACGCTCCCATGTTTTTAGAAATGCTTAAAATTTTTGGA

CTTTTAAGCCAAGCACATCATGACGATGTGTTAAAGATTTTAGAAAAGATACTTCAAAAT

>47:5

GTGAGTTTGATTAAAGCTAGTGGTGATAAAAAAGTGATTGAGGTTTCCATTCCTTTAACT

------------TCAATTTCAGGCAAAGTGCGTGTGAAAATCAGACATGCCTTTAGCGAT

TATGGTATTTCAACAGCGACTAGAAAAATCCCTTTTAGTTTAAAACATTATGTAGAGTGG

CAGATCGGTTATGATGGCCCCATTAAAGATAAAGAA---AAATTTGAACTCACTACTTTA

AAAGATGAAAAATATCATTTTTTAGGGGCTAATAATAAAGTAAAAACTCTTTATGAATTG

AGTGAAATGATTGATTACGCTAAGCAATTAGGTTTAATCAGT---------TTAGAAAAT

TTAGAAAATACTTTAAAATATTTAGAAAAACAAAAACAATTTATAGAAGATAATTTTATG

ATCACAAGAGAAAGATTTAGATCGCATCAATTTGGTGGCATGGATTTTGAACTCTCACGC

ATTTCTTATCCCTTACTCATTTATTCTTTTAATGATAATCAGTTGAGCGAAATTGTTATT

AGAGAACAACAATATGGCTCTAAAACCCAAGCCATG---CTGTATTTTTGCTTTTCTATT

TTGGAGTTAAAAACCGCTACCCCCTTATTAAACAGAACGGCTGAGCTCAAAGAGCATGCT

CTTTTGATCATCCATGAAGCCAACGCTCCCATGTTTTTAGAAATGCTTAAAATTTTTGGA

CTTTTAAGCCAAGCGCACCATAACGATGTGTTAAAGATTTTAGAAAAAATACTTCAAAAT

>KH0040

GTGAGTTTGATTAAAGTTAGTGGTGATAAAAAAGCGATTGAGGTTTCTATTCCCTTAACT

------------TCAATTTCAGGTAAAGTGCGTGTGAAAATCAGACATGCCTTTAGCGAT

TATGGTGTTTCAACAGCGACTAGAAAAATCCCTTTTAGTTTAAAGCATTATGTAGAGTGG

CAAATCGGTTATGATGTCCCCATTAAAGATAAAGAA---AAATTTGAACTCACTACTTTA

AAAGATGAAAAATATCATTTTTTAGGGGCTAATAATAAAGTAAAAACTCTTTATGAATTG

AGCGAAATGATTTATTACGCCAAGCAATTAGGTTTAATCAGT---------TTAGAAAAT

TTAGAAAATACTTTAAAATATTTAGAAAAACAAAAACAATTTATAGAAGATAATTTTATG

ATTACAAGAGAAAGATTTAGATCGCATCAATTTGGTGGCATGGATTTTGAACTCTCACGC

ATTTCTTATCCTTTACTCATTCATTCTTTTAATGATAATCAATTGAGCGAAATTGTTATT

AGAGAACAACAATATGGTTCTAAAACCCAAGCTATG---CTGTATTTTTGCTTTTCTATT

TTGGAATTAAAAACCGCTACTTCCTTATTAAACAGAACGGCTACGCCCAAAGAACATGCC

CTTTTGATTATCCGTAAAGCCAACGCTCCCATGTTTTTAGAAATGCTTAAAATTTTTGGA

CTTTTAAGCCAAGCGCACCATGACGATGTGTTAAAGATTTTAGAAAAAATACTTCAAAAT

>GC30-HL

GTGAGTTTGATTAAAGTTAGTGGTGATAAAAAAGTGATTGAGGTTTCTATTCCTTTAACT

------------TCAATTTCAGGCAAAGCGCGTGTGAAAATCAGGCATGCCTTTAGCGAT

TATGGCATTTCAACAGCGACTAGAAAAATCCCTTTTAGTTTAAAACATTATGTAGAGTGG

CAGATCGGTTATGATGTCCCCATTAAAGATAAAGAA---AAATTTGAACTCACTACTTTA

AAAGATGAAAAATATCATTTTTTAGGGGCTAATAATAAAAGAAAAACCCTTTATGAATTG

AGCGAAATAATTGATTACGCTAAGCAATTAAATTTAATCAGT---------TTAGAAAAT

TTAGAAAATACTTTAAAATATTTAGAAAAACAAAAACAATTCATAGAAGATAGTTTTATG

ATTACAAGAGAAAGATTTAGATCGTATCAATTTGGTGGCATGGATTTTGAACTTTCACGC

ATTTCTTATCCCTTACTCATTCATTCTTTTAATGATAATCAGTTGAGCGAAATCGTTATT

AGAGAGCTACAATACGGCTCTAAAACCCAAGCCATG---CTGTATTTTTGCTTTTCTATT

TTGGAGTTAAAAACCGCTACCCCCTTATTAAACAGAACGGCTGCACTCAAAGAACATGCC

CTTTTAACTATCCATAAAACCAACGCTCTTGTGTTTTTAGAAATGCTTAAAATTTTTGGG

CTTTTAAGCCAAGCACACCATAACGATGTGTTAAAGATTTTAGAAAAAATACTTCAAAAT

>GC26-HL

GTGAGTTTGATTAAAGTTAGTGGTGATAAAAAAGTGATTGAGGTTTCTATTCCTTTAACT

------------TCAATTTCAGGCAAAGCGCGTGTGAAAATCAGGCATGCCTTTAGCGAT

TATGGCATTTCAACAGCGACTAGAAAAATCCCTTTTAGTTTAAAACATTATGTAGAGTGG

CAGATCGGTTATGATGTCCCCATTAAAGATAAAGAA---AAATTTGAACTCACTACTTTA

AAAGATGAAAAATATCATTTTTTAGGGGCTAATAATAAAAGAAAAACCCTTTATGAATTG

AGCGAAATAATTGATTACGCTAAGCAATTAAATTTAATCAGT---------TTAGAAAAT

TTAGAAAATACTTTAAAATATTTAGAAAAACAAAAACAATTCATAGAAGATAGTTTTATG

ATTACAAGAGAAAGATTTAGATCGTATCAATTTGGTGGCATGGATTTTGAACTTTCACGC

ATTTCTTATCCCTTACTCATTCATTCTTTTAATGATAATCAGTTGAGCGAAATCGTTATT

AGAGAGCTACAATACGGCTCTAAAACCCAAGCCATG---CTGTATTTTTGCTTTTCTATT

TTGGAGTTAAAAACCGCTACCCCCTTATTAAACAGAACGGCTGCACTCAAAGAACATGCC

CTTTTAACTATCCATAAAACCAACGCTCTTGTGTTTTTAGAAATGCTTAAAATTTTTGGG

CTTTTAAGCCAAGCACACCATAACGATGTGTTAAAGATTTTAGAAAAAATACTTCAAAAT

>MHP43

------------------------------AAAGTGATTGAGGTTTCTATTCCTTTAACT

------------TCCATTTCAGGCAAAGTTCGTGTGAAAATCAGACATGCCTTTAGCGAT

TATGGCATTTCAACAGCGACTAGAAAAATCCCTTTCAGTTTAAAGCATTATGTAGAGTGG

CAAATCGGTTATGATGTCCCCATTAAAGATAAAGAA---AAATTTGAACTCACTACCCTA

AAAGATGAAAAATATCATTTTTTAGGGGCTAATAATAAAAGAAAAACCCTTTATGAATTG

AGCGAAATGATTGATTACGCTAAGCGATTGGGTTTAATCAGT---------TTAGAAAAT

TTAGAAAATACTTTAAAATATTTAGAAAAACAAAAACAATTTATAGAAGATAATTTTATG

ATTACAAGAGAAAGATTTAGATCGCATCAATTTGGTGGCATGGATTTTGAACTTTCACGC

ATTTCTTATCCTTTACTCATTCATTCTTTTAATGATAACCAATTGAGTGAAATTGTTATT

AGAGAGCAACAATATGGCTCTAAAACCCAAGCCATG---CTGTATTTTTGCTTTTCTATT

CTGGAATTAAAAACCGCTACACCTTTATTAAATAGAACCGCTGCACTCAAAGAACATGCT

CTTTTAACTATCCATAAAACCAACGCTCTTGTGTTTTTAGAAATGCTTAAAATTTTTGGA

CTCTTAAGCCAAGCGCATCATAACGATGTGTTAAAGATTTTAGAAAAAATACTTGAAAAT

>UM119

GTGAGTTTGATTAAAGTTAGTGGTGATAAAAAAGCGATTGAGGTTTCTATTCCCTTAACT

------------TCAATTTCAGGTAAAGTGCGTGTGAAAATCAGACATGCTTTTAGCGAT

TATGGTGTTTCAACAGCGACTAGAAAAATCCCTTTTAGTTTAAAACATTATGTAGAGTGG

CAAATCGGTTATGATGTCCCCATTAAAGATAAAGAA---AAATTTGAACTCACTACTTTA

AAAGATGAAAAATATCATTTTTTAGGGGCTAATAGTAAAACAAAAACTCTTTATGAATTG

AGCGAGATCATTTACTATGCCAAGCAATTAGATTTAATCAGT---------TTAGAAAAT

TTAGAAAATACTTTAAAATATTTAGAAAAACAAAAACAATTTATAGAAGATAATTTTATG

ATTACAAGAGAAAGATTTAGATCGCATCAATTTGGTGGCATGGATTTTGAACTTTCACGC

ATTTCTTATCCTTTACTCATTCATTCTTTTAATGATAATCAATTGAGCGAAATTGTTATT

AGAGAACAACAATATGGTTCTAAAACCCAAGCCATG---CTATATTTTTGCTTTTCTATT

TTGGAGTTAAAAACCGCTACTCCCTTATTAAACAGAACGGCTACGCTCAAAGAACATGCT

CTTTTGATTATCCATAAAACCAACGCTCCCATGTTTTTAAAAATGCTTAAAATTTTTGGA

CTTTTAAGCCAAGTGCACCATGACGATGTGTTAAAGATTTTAGAAAAAATACTTCAAAAT

>KH0182

GTGAGTTTGATTAAAATTAACCATGATAAAAAAGTGATTGAGGTTTCTATTCCTTTAACT

------------TCAATTTCAGGCAAAGTGCGTGTGAAAATCAGACATGCCTTTAGTGAT

TATGGTATTTCAACAGCGACTAGAACAATCCCTTTTAGTTTAAAACATTATGTAGAGTGG

CAGATCGGTTATGATGTCCCCATTAAAGATAAAGAA---AAATTTGAACTCACTACTTTA

AGAGATGAAAAATATCATTTTTTAGGGGCTAATAATAAAGTAAAAACTCTTTATGAATTG

AGCGAAATGATTTATTACGCCAAGCAATTAGGTTTAATCAGT---------TTAGAAAAT

TTAGAAAATACTTTAAAATATTTAGAAAAACAAAAACAATTTATAGAAGATAATTTTATG

ATTACAAGAGAAAGATTTAGATCGCATCAATTTGGTGGCATGGATTTTGAACTCTCACGC

ATTTCTTATCCTTTACTCATTCATTCTTTTAATGATAATCAATTGAGCGAAATTGTTATT

AGAGAACAACAATATGGTTCTAAAACCCAAGCTATG---CTGTATTTTTGCTTTTCTATT

TTGGAATTAAAAACCGCTACTTCCTTATTAAACAGAACGGCTACGCCCAAAGAACATGCC

CTTTTGATTATCCGTAAAGCCAACGCTCCCATGTTTTTAGAAATGCTTAAAATTTTTGGA

CTTTTAAGCCAAGCGCACCATGACGATGTGTTAAAGATTTTAGAAAAAATACTTCAAAAT

>HP14052

GTGAGTTTGATTAAAATTAACCATGATGAAAAAGTGATTGGGATTTCTATTCCTTTAACT

------------TCAATTTCAGGCAAAGTGCGTGTGAAAATCAGACATGCCTTTAGCGAT

TATGGTGTTTCAACAGCGACTAGAACAATCCCTTTTAGTTTAAAGCATTATGTAGAGTGG

CAGATCGGTTATGATGTCCCCATTAAAGATAAAGAA---AAATTTGAACTTACTACTTTA

AAAGATGAAAAATATCATTTTTTAGGGGCTAATAATAAAGTAAAAACTCTTTATGAATTG

AGCGAGATCATTTATTATGCCAAACAATTAGGTTTAATCAGT---------TTAGAAAAT

TTAGAAAATACTTTAAAATATTTAGAAAAACAAAAACAATTTATAGAAGATAATTTTATG

ATTACAAGAGAAAGATTTAGATCGCATCAATTTGGTGGCATGGCTTTTGAACTTTCACGC

ATTTCTTATCCTTTACTCATTCATTCTTTTAATGATAATCAATTGAGCGAAATTGTTATT

AGAGAACAACAATATGGTTCTAAAACCCAAGCCATG---CTGTATTTTTGCTTTTCTATT

TTGGAGTTAAAAACCGCTACTCCCTTATTAAACAGAACGGCTACGCTCAAAGAACATGCT

TTTTTGATTATCCATAAAACCAACGCTCCCATGTTTTTAGAAATGCTTAAAATTTTTGGA

CTTTTAAGCCAAGCGCACCATGACGATGTGTTAAAGATTTTAGAAAAAATACTTCAAAAT

>KH9

GTGAGTTTGATTAGGATTGATGATAGTAAAAAAGTAATTGAGGTTTCTATTCCTTTAACT

------------TCAATTTCAGGCAAAGTGCGTGTGAAAATCAGGCATGCCTTTAGCGAT

TATGGTATTTCAACAGCGACTAGAAAAATCCCTTTTAGTTTAAAACATTATGTAGAGTGG

CAGATCGGTTATGATGTCCCCATTAAAGATAAAGAA---AAATTTGAACTCACTACCCTA

AAAGATGAAAAATATCATTTTTTAGGGGCTAATAATAAAGTAAAAACTCTTTATGAATTA

AGCGAAATGATTTATTACGCTAAGCAATTAGGTTTAATTGGT---------TTAGAAAAT

TTAGAAAATACTTTAAAATATTTAGAAAAACAAAAACAATTTATAGAAGATAATTTCACG

ATTACAAGAGAAAGATTTAGATCGCATCAATTTGGCGGCATGGATTTTGAACTTTCACAC

ATTTCTTATCCTTTACTCATTCATTCTTTTGATGATAATCAGTTGAGTGAAATTGTTATT

AGAGAGCAACAATATGGCTCTAAAACCCAAGCCATG---TTGTATTTTTGCTTTTCTATT

TTGGAGTTAAAAACCGCTACCCCCTTATTAAATAGAACCGCTACACTCAAAGAACATGCT

CTTTTGATTATCCATAAAACCAACGCTCTTGTGTTTTTAGAAATGCTTAAAATTTTTGGA

CTTTTAAGCCAAGCACACCATAACGATGTGTTAAGA------------------------

>UM171S

GTGAGTTTGATTAAAATTAACCATGATGAAAAAGTGATTGAGGTTTCTATTCCCTTAACT

------------TCAATTTCAGGCAAAGTGCGTGTGAAAATCAGACATGCCTTTAGCGAT

TATGGTGTTTCAACAGCGACTAGAAAAATCCCTTTTAGTTTAAAACATTATGTAGAGTGG

CAAATCGGTTATGATGTTCCTATTAAAGATAAAGAA---AAATTTGAACTCACTACTTTA

AAAGATGAAAAATATCATTTTTTAGGGGCTAATAATAAAGTAAAAACTCTTTATGAATTG

AGCGAAATGATTTATTACGCCAAGCAATTAGGTTTAATCAGT---------TTAGGAAAT

TTAGAAAATACTTTAAAATATTTAGAAAAACAAAAACAATTTATAGAAGATAATTTTATG

ATTACAAGAGAAAGATTTAGATCGCATCAATTTGGTGGCATGGATTTTGAACTTTCACAC

ATTTCTTATCCTTTACTCATTCATTCTTTTAATGATAATCAATTGAGCGAGATTGTTATT

AGAGAACAACAATATGGTTCTAAAACCCAAGCCATG---CTGTATTTTTGCTTTTCTATT

TTGGAGTTAAAAACCGCTACTCCCTTATTAAACAGAACGGCTACGCTCAAAGAACATGCT

CTTTTGATTATCTATAAAACCAACGCTCCCATGTTTTTAAAAATGCTTAAAATTTTTGGA

CTTTTAAGCCAAGTGCACCATGACGATGTGTTAAAGATTTTAGAAAAAATACTTCAAAAT

>B528A

GTGAGTTTGATTAGGATTGATGATAGTAAAAAAGCGATTGAGGTTTCTATTCCTTTAACT

------------TCAATTTCAGGCAAAGCGCGTGTGAAAATCAGACATGCCTTTAGCGAT

TATGGTATTTCAACAGCGACCAGAAAAATCCCTTTTAGTTTAAAACATTATGTAGAGTGG

CAAATCGGTTATGATGTCCCCATTAAAGATAAAGAA---AAATTGGAGCTCACTACTTTA

AAAGATGAAAAATATCATTTTTTAGGGGCTAATAATAAAGTGAAAACCCTTTATGAATTG

AGCGAAATGATTGATTACGCTAAGCGATTGGGTTTAATCAGT---------TTAGAAAAT

TTAGAAAATACTTTAAAATATTTAGAAAAACAAAAACAATTTATAGAAGATAATTTTATA

------AGAGAAAGATTTAGATCGCATCAATTTGGTGGCATGGATTTTGAACTTTCACGC

ATTTCTTATCCTTTACTCATTCATTCTTTTAATGATAATCAATTGAGTGAAATCGTTATT

AGAGAGCAACAATATGGCTCTAAAACCCAAGCCATG---CTGTATTTTTGCTTTTCTATT

TTGGAATTAAAAACCGCTACTCCCTTATTAAATAGAACGGCTGCACTCAAAGAACATGCC

CTTTTAACTATCCATAAAACCAACGCTCTTGTGTTTTTAGAAATGCTTAAAATTTTTGGA

CTTTTAAGCCAAGTGCACCATAACGATGTGTTAAAGATTTTAGAAAAAATATTTCAAAAT

>HP13012

GTGAGTTTGATTAAAGTTAGTGGTGATAAAAAAGCGATTGAGGTTTCTATTCCCTTAACT

------------TCAATTTCAGGCAAAATGCGTGTGAAAATCAGACATGCCTTTAGCGAT

TATGGTATTTCAACAGCGACTAGAAAAATCCCTTTTAGTTTAAAGCATTATGTAGAGTGG

CAGATCGGTTATGATGTCCCCATTAAAGATAAAGAA---AAATTTGAACTCACTACTTTA

AAAGATGAAAAATATCATTTTTTAGGGGCTAATAATAAAATAAAAACTCTTTATGAATTG

AGCGAGATCATTTACTATGCCAAGCAATTAGGTTTAATCAGT---------TTAGAAAAT

TTAGAAAATACTTTAAAATATTTAGAAAAACAAAAACAATTTATAGAAGATAATTTTATG

ATTACAAGAGAAAGATTTAGATCGCATCAATTTGGTGGCATGGATTTTGAACTTTCACGC

ATTTCTTATCCTTTACTCATTCATTCTTTTAATGATAATCAGTTGAGCGAGATTATTATT

AGAGAGCAACAATATGGTTCTAAAACCCAAGCCATG---CTGTATTTTTGCTTTTCTATT

TTGGAGTTAAAAACCGCTACTCCCTTATTAAACAGAACGGCTACGCCCAAAGAACATGCT

CTTTTGATTATCCATAAAACCAACGCTCCCATGTTTTTAAAAATGCTTAAAATTTTTGGA

CTTTTAAGCCAAGCACACCATGACGATGTGTTAAAGATTTTAAGAAAAATACTTCAAAAT

>2004-137

GTGAGTTTGATTAAAGTTAATGATGATAAAAAAGTGATTGAGGTTTCTATTCCTTTAACT

------------TCCATTTCAGGCAAAGTTCGTGTGAAAATTAGGCATGCCTTTAGTGAT

TATGGTATTTCAACAGCGACTAGAAAAATCCCTTTCAGTTTAAAGCATTATGTAGAGTGG

CAAATCGGTTATGATGTCCCCATTAAAGATAAAGAA---AAATTTGAACTCACTACTTTA

AAAGATGAAAAATATCATTTTTTAGGGGCTAATAATAGAGTAAAGACTCTTTATGAATTG

AGCGAAATAATTGATTACGCTAAGCGATTGGGTTTAATCAGT---------TTAGAAAAT

TTAGAAAATACTTTAAAATATTTAGAAAAACAAAAACAATTCATAGAAGATAGTTTTATA

ATTACAAGAGAAAGATTTAGATCGCATCAATTTGGTGGCATGGATTTTGAACTTTCACGC

ATCTCTTATCCTTTACTCATTCATTCTTTTAATGATAATCAGTTGAGCGAAATCGTTATT

AGAGAGCAACAATACGGCTCTAAAACCCAAGCTATG---CTGTATTTTTGCTTTTCTATT

TTGGAATTAAAAACCACTACCCCCTTATTAAATAGAACGGCTGCACTCAAAGAAAATGCT

CTTTTGATTATCTATAAAACCAACGCTCTTGTGTTTTTAGAAATGCTTAAAATTTTTGGA

CTTTTAAGCCAAGCGCACCATAACGATGTGTTAAAGATTTTAGAAAAAATACTTCAAAAT

>ZH79

GTGAGTTTGATTAAAGTTAGTGGTGATAAAAAAGCGATTGAGGTTTCTATTCCTTTAGCT

------------TCAATTTCAGGCAAAGTGCGTGTGAAAATCAGACATGCCTTTAGCGAT

TATGGTGTTTCAACAGCGACTAGAAAAATCCCTTTTAGTTTAAAACATTATGTAGAGTGG

CAGATCGGTTATGATGCCCCCATTAAAGATAAAGAA---AAATTTGAACTCACTACTTTA

AAAGATGAAAAATATCATTTTTCAGGGGCTAATGGTAAAACAAAAACTCTTTATGAATTG

AGCGAGATCATTTATTATGCTAAACAATTAGGTTTAATCAGT---------TTAAAAAAT

TTAGAAAATACTTTAAAATATTTAGAAAAACAAAAACAATTTATAGAAGATAATTTTATG

ATTACAAGAGAAAGATTTAGATTACATCAATTTGGTGGCATGGATTTTGAACTCTCACGC

ATTTCTTATCCTTTACTCATTCATTCTTTTAATGATAATCAGTTGAGCGAAATCGTTATT

AGAGAGCAACAATATGGCTCTAAAACCCAAGCCATG---CTCTATTTTTGCTTTTCTATT

TTGGAGTTAAAAACCGCTACCCCTTTATTAAATAGAACGGCTGCACTCAAAGAACATGCT

TTTTTAATTATCCATAAAACCAACGCTCTTGTGTTTTTAGAAATGCTTAAAATTTTTGGG

CTTTTAAGCCAAGCGCACCATAACGATGTGTTAAAGATTTTAGAAAAAATACTTCAAAAT

>MG2011-41

GTGAGTTTGATTAGGATTGATGATGATAAAAAAGCGATTGAGGTTTCTATTCCTTTAACT

------------TCAATTTCAGGCAAAGTGCGTGTGAAAATCAGACATGCCTTTAGCGAT

TATGGCATTTCAACAGCGACTAGAAAAATCCCTTTTAGTTTAAAGCATTATGTAGAGTGG

CAAATCGGTTATGATGTCCCCATTAAAGATAAAGAA---AAATTTGAACTCACTACTTTA

AAAGATGAAAAATATCATTTTTTAGGGGCTAATAATAAAGTAAAAACCCTTTATGAATTG

AGCGAAATAATTTATTACGCTAAGCAATTAGGTTTAATCAGT---------TTAGAAAAA

TTAGAAAATACTTTAAAATATTTAGAAAAACAAAAACAATTTATAGAAGATAATTTCATG

ATTACAAGAGAAAGATTTAGATCGCATCAATTTGGTGGCATGGATTTTGAACTTTCACGC

ATTTCTTATCCTTTACTCATTCATTCTTTTAATGATAATCAATTGAGTGAAATCGTTATT

AGAGAGCAACAATATGGCTCTAAAACCCAAGCCATG---CTGTATTTTTGCTTTTCTATT

TTGGAATTAAAAACCGCTACCCCCTTATTAAACAGAACGGCTGCACTCAAAGAACATGCC

CTTTTAACTATCCATAAAACCAACGCTCTCATGTTTTTAGAAATGCTTAAAATTTTTGGA

CTTTTAAGCCAAGCGCACCATAACGAGTGT------------------------------

>G-Mx-2011-41

GTGAGTTTGATTAGGATTGATGATGATAAAAAAGCGATTGAGGTTTCTATTCCTTTAACT

------------TCAATTTCAGGCAAAGTGCGTGTGAAAATCAGACATGCCTTTAGCGAT

TATGGCATTTCAACAGCGACTAGAAAAATCCCTTTTAGTTTAAAGCATTATGTAGAGTGG

CAAATCGGTTATGATGTCCCCATTAAAGATAAAGAA---AAATTTGAACTCACTACTTTA

AAAGATGAAAAATATCATTTTTTAGGGGCTAATAATAAAGTAAAAACCCTTTATGAATTG

AGCGAAATAATTTATTACGCTAAGCAATTAGGTTTAATCAGT---------TTAGAAAAA

TTAGAAAATACTTTAAAATATTTAGAAAAACAAAAACAATTTATAGAAGATAATTTCATG

ATTACAAGAGAAAGATTTAGATCGCATCAATTTGGTGGCATGGATTTTGAACTTTCACGC

ATTTCTTATCCTTTACTCATTCATTCTTTTAATGATAATCAATTGAGTGAAATCGTTATT

AGAGAGCAACAATATGGCTCTAAAACCCAAGCCATG---CTGTATTTTTGCTTTTCTATT

TTGGAATTAAAAACCGCTACCCCCTTATTAAACAGAACGGCTGCACTCAAAGAACATGCC

CTTTTAACTATCCATAAAACCAACGCTCTCATGTTTTTAGAAATGCTTAAAATTTTTGGA

CTTTTAAGCCAAGCGCACCATAACGAGTGT------------------------------

>PNG84A

GTGAGTTTGATTAAAGTTAGTGGTGATAAAAAAGTGATTGAGGTTTCTATTCCTTTAACT

------------TCAATTTCAGGCAAAGTGCGTGTGAAAATCAGAAATGCCTTTAGCGAC

TATGATATTTCAACAGCGACCAGAAAAATCCCTCTCAGTTTAAAACATTATGTAGAGTGG

CAAATCGGTTATGATGTTCCCATTAAAGATAAAGAA---AAATTTGAACTCACTACTTTA

AAAGATGAAAAATATCATTTTTTAGGGGCTAATAATAAGGTAAAAACTCTTTATGAATTA

AGCGAAATAATTTATTACGCTAAGCAATTAGGTTTAATCAGT---------TTAGAAAAT

TTAGAAAATACTTTAAAATATTTAGAAAAACAAAAACAATTTATAGAAGATAATTTTATG

ATTACAAGAGAAAGATTTAGATCGCATCAATTTGGCGGCATGGATTTTAAATTTTCACAT

ATTTCTTATCCTTTACTCATTCATTCTTTTAACGATAATCAGTTGAGCGAAATGGTTATT

AGAGAACAACAATATGGCTCTAAAACCCAAGCCATG---CTGTATTTTTGCTTTTCTGTT

TTGGAGTTAAAAACCGCTACCCCCTTATTAAACAGAACGGCTGCACTCAAAGAACATGCT

CTTTTGATTATCCATAAAACCAACGCTCTCATGTTTTTAGAAACGCTTAAAATTTTTGGA

CTTTTAAGCCAAGCGCACCATAACGATGTGTTAAAGATTTTAGAAAAAATACTTCAAAAT

>UM196

GTGAGTTTGATTAAAATTAACCATGATGAAAAAGTGATTGAGGTTTTTATTCCTTTAACT

------------TCAATTTCAGGCAAAGTGCGTGTGAAAATCAGACATGCCTTTAGCGAT

TATGGTATTTCAACAGCGACTAGAACAATCCCTTTTAGTTTAAAACATTATGTAGAGTGG

CAAATCGGTTATGATGTCCCCATTAAAGATAAAGAA---AAATTTGAACTCACTACTTTA

AAAGATGAAAAATATCATTTTTTAGAGGCTAATAATAAAGTAAAAACTCTTTATGAATTG

AGCGAGATCATTTACTATGCCAAGCAATTAGGTTTAATCAGT---------TTAGAAAAT

TTAGAAAATACTTTAAAATATTTAGAAAAACAAAAACAATTTATAGAAGATAATTTTATG

ATTACAAGAGAAAGATTTAGATCGCATCAATTTGGTGGCATGGATTTTGAACTTTCACGC

ATTTCTTATCCTTTACTCATTCATTCTTTTAATGATAATCAATTGAGCGAAATTGTTATT

AGAGAACAACAATATGGTTCTAAAACCCAAGCTATG---CTGTATTTTTGCTTTTCTATT

TTGGAGTTAAAAACCGCTACTCCCTTATTAAACAGAACGGCTACGCGCAAAGAACATGCT

CTTTTGATTATCCATAAAACCAACGCTCCCATGTTTTTAGAAATGCTTAAAATTTTTGGA

CTTTTAAGCCAAGCGCACCATGACGATGTGTTAAAGATTTTAGAAAAAATACTTCAAAAT

>Nic38-A

GTGAGTTTGATTAAAGTTAATGATGATAAAAAAGCGATTGAGGTTTCTATTCCTTTAACT

------------TCCATTTCAGGCAAAGCGCGTGTGAAAATCAGACATGCCTTTAGCGAT

TATGGCATTTCAACAGCGACCAGAAAAATCCCTTTTAGCTTAAAACATTATGTAGAGTGG

CAAATCGGTTATGATGCCCCCATTAAAGATAAAGAA---AAATTTGAACTCACTACCCTA

AAAGATGAAAAATATCATTTTTTAGGGGCTAATAATAAAGTAAAAACCCTTTATGAATTG

AGCGAAATAATTGATTACGCTAAGCGATTGGGTTTAATCAGT---------TTAGAAAAT

TTAGAAAATACTTTAAAATATTTAGAAAAACAAAAACAATTTATAGAATATAATTTCACG

ATTACAAGAGAAAGATTTAGATCGCATCAATTTGGGGGCATGGATTTTGAACTTTCACGC

ATTTCTTATCCTTTACTCATTCATTCTTTCAACGATAATCAGTTGAGCGAAATCGTTATT

AGAGAGCAACAATACGGCTCTAAAACCCAAGCCATG---CTGTATTTTTGCTTTTCTATT

CTGGAATTAAAAACCGCTACCCCCTTATTAAATAGAACGGCTGCACTCAAAGAACATGCC

CTTTTAACTATCCATAAAACCAACGCTCTTGTGTTTTTAGAAATGCTTAAAATTTTTGGA

CTTTTAAGCAAAGCGCACCATAACGATGTGTTAAAGATTTTAGAAAAAATACTTGAAAAT

>BMG112

------------------------GGTAAAAAAGCGATTGAGGTTTCTATTCCTTTAACT

------------TCAATTTCAGGCAAAGCGCGTGTGAAAATCAGACATGCCTTTAGCGAT

TATGGTATTTCAACAGCGACTAGAAAAATCCCTTTTAGTTTAAAACATTATGTAGAGTGG

CAGATCGGTTATGATGTCCCCATTAAAGATAAAGAA---AAATTTGAACTCACTACTTTA

AAAGATGAAAAATATCATTTTTTAGGGGTTAATAATAAAGTGAAAACTCTTTATGAATTA

AGCGAAATGATTTATTACGCTAAGCAATTAAATTTAATCAGT---------TTAGAAAAT

TTAGAAAATACTTTAAAATATTTAGAAAAACAAAAACAATTTATAGAAGATAATTTCACG

ATTACAAGAGAAAGATTTAGATCGCATCAATTTGGTGGCATGGATTTTGAACTCTCACGC

ATTTCTTATCCTTTACTCATTCATTCTTTTAATGATAATCAATTGAGTGAAATCGTTATT

AGAGAGCAACAATATGGCTCTAAAACCCAAGCCATG---CTGTATTTTTGCTTTTCTATT

TTGGAGTTAAAAACCGCTACCCCCTTATTAAATAGAACGGCTGCACTCAAAGAACAGGCT

CTTTTGATTATCCATAAAACCAACGCTCTCATGTTTTTAGAAATGCTTAAAATTTTTGGA

CTTTTAAGCCAAGCGCACCATAACGATGTGTTAAAGATTTTAGAAAAAATACTTCAAAAT

>PIMM-FM-UNAM-53C1

GTGAGTTTGATTAAAGTTAGTGGTGATAAAAAAGCGATTGAGGTTTCCATTCCTTTAACT

------------TCAATTTCAGGCAAAGTGCGTGTGAAAATCAGACATGCCTTTAGCGAT

TATGGTATTTCAACAGCGACTAGAAAAATCCCTTTTAGTTTAAAACATTATATAGAGTGG

CAGATCGGTTATGATGTCCCCATTAAAGATAAAGAA---AAATTTGAACTCACTACTTTA

AAAGATGAAAAATATCATTTTTTAGGGGCTAATAATAAAGTAAAAACTCTTTATGAATTA

AGCGAAATAATTGATTACGCTAAGCAATTAAATTTAATCAGT---------TTAGAAAAT

TTAGAAAATACTTTAAAATATTTAGAAAAACAAAAACAATTCATAGAAGATAGTTTCACG

ATTACAAGAGAAAGATTTAGATCGCATCAATTTGGTGGCATGGATTTTGAACTCTCACGC

ATTTCTTATCCTTTACTCATTCATTCTTTTAACGATAATCAATTGAGTGAAATCGTTATT

AGAGAACAACAATACGGCTCTAAAACCCAAGCCATG---CTGTATTTTTGCTTTTCTATT

TTGGAGTTAAAAACCGCTACCCCCTTATTAAACAGAACGGCTATGCTCAAAGAGCATGCT

CTTTTGATTATCCATAAAACCAACGCTCCCATGTTTTTAGAAATGCTTAAAATTTTTGGA

CTTTTAAGCCAAGCGCACCATAACGATGTGTTAAAGATTTTAGAAAAGATACTTCAAAAT

>ZH80

GTGAGTTTGATTAAGATTGATAATGATAAAAAAGCGATTGAGGTTTCTATTCCTTTAACT

------------TCAATTTCAGGCAAAGCGCGTGTGAAAATCAGACATGCCTTTAGCGAT

TATGGCATTTCAACAGCGACTAGAAAAATCCCTTTTAGTTTAAAACATTATATAGAGTGG

CAGATCGGTTATGATGTCCCCATTAAAGATAAAGAA---AAATTTGAGCTCACTACCCTA

AAAGATGAAAAATATCATTTTTTAGGGGCTAATAATAAAGTAAAAACCCTTTATGAATTG

AGTGAGATAATCTATTACGCTAAGCAATTAAATTTAATCAGT---------TTAGAAAAT

TTAGAAAATACTTTAAAATATTTAGAAAAACAAAAACAATTTATAGAAGATAATTTT---

---ATAAGAGAAAGATTTAGATCGCATCAATTTGGTGGCGTGGATTTTGAACTTTCACGC

ATCTCTTATCCTTTACTCATTCATTCTTTTAATGATAATCAGTTGAGCGAAATTGTTATT

AGAGAGCAACAATATGGCTCTAAAACCCAAGCCATG---CTGTATTTTTGCTTTTCTATT

TTGGAGTTAAAAACCGCTACCCCCTTATTAAATAGAACGGCCGCACTCAAAGAACATGCC

ATTTTAACTATCCATAAAGCTAACGCTCCCATGTTTTTAGAAATGCTTAAAATTTTTGGG

CTTTTAAGCCAAGTGCACCATAACGATGTGTTAAAGATTTTAGAAAAAATACTTCAAAAT

>UM233S

GTGAGTTTGATTAAAGTTAGTGGTGATAAAAAAGCGATTGAGGTTTCTATTCCCTTAACT

------------TCAATTTCAGGTAAAGTGCGTGTGAAAATCAGACATGCCTTTAGCGAT

TATGGTGTTTCAACAGCGACTAGAACAATCCCTTTTAGTTTAAAGCATTATGTAGAGTGG

CAGATCGGTTATGATGTCCCCATTAAAGATAAAGAA---AAATTTGAACTTACTACTTTA

AAAGATGAAAAATATCATTTTTTAGGGGCTAATAGTAAAACAAAAACTCTTTATGAATTG

AGCGAGATCATTTACTATGCCAAGCAATTAAGTTTAATCAGT---------TTAGAAAAT

TTAGAAAATACTTTAAAATATTTAGAAAAACAAAAACAATTTATAGAAGATAATTTTATG

ATTACAAGAGAAAGATTTAGATCACATCAATTTGGTGGCATGGATTTTGAACTTTCACGC

ATTTCTTATCCTTTACTCATTCATTCTTTTGATGATAATCAATTGAGCGAAATTGTTATT

AGAGAGCAACAATATGGTTCTAAAACCCAAGCCATA---CTGTATTTTTGCTTTTCTATT

TTGGAGTTAAAAACCGCTACTCCCTTATTAAACAGAACGGCTACGCTTAAAGAACATGCT

CTTTTGATTATCCATAAAACCAACGCTCCCATGTTTTTAAAAATGCTTAAAATTTTTGGA

CTTTTAAGCCAAGTGCACCATGACGATGTGTTAAAGATTTTAGAAAAAATACTTCAAAAT

>UM233R

GTGAGTTTGATTAAAGTTAGTGGTGATAAAAAAGCGATTGAGGTTTCTATTCCCTTAACT

------------TCAATTTCAGGTAAAGTGCGTGTGAAAATCAGACATGCCTTTAGCGAT

TATGGTGTTTCAACAGCGACTAGAACAATCCCTTTTAGTTTAAAGCATTATGTAGAGTGG

CAGATCGGTTATGATGTCCCCATTAAAGATAAAGAA---AAATTTGAACTTACTACTTTA

AAAGATGAAAAATATCATTTTTTAGGGGCTAATAGTAAAACAAAAACTCTTTATGAATTG

AGCGAGATCATTTACTATGCCAAGCAATTAAGTTTAATCAGT---------TTAGAAAAT

TTAGAAAATACTTTAAAATATTTAGAAAAACAAAAACAATTTATAGAAGATAATTTTATG

ATTACAAGAGAAAGATTTAGATCACATCAATTTGGTGGCATGGATTTTGAACTTTCACGC

ATTTCTTATCCTTTACTCATTCATTCTTTTGATGATAATCAATTGAGCGAAATTGTTATT

AGAGAGCAACAATATGGTTCTAAAACCCAAGCCATA---CTGTATTTTTGCTTTTCTATT

TTGGAGTTAAAAACCGCTACTCCCTTATTAAACAGAACGGCTACGCTTAAAGAACATGCT

CTTTTGATTATCCATAAAACCAACGCTCCCATGTTTTTAAAAATGCTTAAAATTTTTGGA

CTTTTAAGCCAAGTGCACCATGACGATGTGTTAAAGATTTTAGAAAAAATACTTCAAAAT

>UM137S

GTGAGTTTGATTAAAGTTAGTGGTGATAAAAAAGCGATTGAGGTTTCTATTCCCTTAACT

------------TCAATTTCAGGTAAAGTGCGTGTGAAAATCAGACATGCCTTTAGCGAT

TATGGTGTTTCAACAGCGACTAGAACAATCCCTTTTAGTTTAAAGCATTATGTAGAGTGG

CAGATCGGTTATGATGTCCCCATTAAAGATAAAGAA---AAATTTGAACTTACTACTTTA

AAAGATGAAAAATATCATTTTTTAGGGGCTAATAGTAAAACAAAAACTCTTTATGAATTG

AGCGAGATCATTTACTATGCCAAGCAATTAAGTTTAATCAGT---------TTAGAAAAT

TTAGAAAATACTTTAAAATATTTAGAAAAACAAAAACAATTTATAGAAGATAATTTTATG

ATTACAAGAGAAAGATTTAGATCACATCAATTTGGTGGCATGGATTTTGAACTTTCACGC

ATTTCTTATCCTTTACTCATTCATTCTTTTGATGATAATCAATTGAGCGAAATTGTTATT

AGAGAGCAACAATATGGTTCTAAAACCCAAGCCATA---CTGTATTTTTGCTTTTCTATT

TTGGAGTTAAAAACCGCTACTCCCTTATTAAACAGAACGGCTACGCTTAAAGAACATGCT

CTTTTGATTATCCATAAAACCAACGCTCCCATGTTTTTAAAAATGCTTAAAATTTTTGGA

CTTTTAAGCCAAGTGCACCATGACGATGTGTTAAAGATTTTAGAAAAAATACTTCAAAAT

>UM137R

GTGAGTTTGATTAAAGTTAGTGGTGATAAAAAAGCGATTGAGGTTTCTATTCCCTTAACT

------------TCAATTTCAGGTAAAGTGCGTGTGAAAATCAGACATGCCTTTAGCGAT

TATGGTGTTTCAACAGCGACTAGAACAATCCCTTTTAGTTTAAAGCATTATGTAGAGTGG

CAGATCGGTTATGATGTCCCCATTAAAGATAAAGAA---AAATTTGAACTTACTACTTTA

AAAGATGAAAAATATCATTTTTTAGGGGCTAATAGTAAAACAAAAACTCTTTATGAATTG

AGCGAGATCATTTACTATGCCAAGCAATTAAGTTTAATCAGT---------TTAGAAAAT

TTAGAAAATACTTTAAAATATTTAGAAAAACAAAAACAATTTATAGAAGATAATTTTATG

ATTACAAGAGAAAGATTTAGATCACATCAATTTGGTGGCATGGATTTTGAACTTTCACGC

ATTTCTTATCCTTTACTCATTCATTCTTTTGATGATAATCAATTGAGCGAAATTGTTATT

AGAGAGCAACAATATGGTTCTAAAACCCAAGCCATA---CTGTATTTTTGCTTTTCTATT

TTGGAGTTAAAAACCGCTACTCCCTTATTAAACAGAACGGCTACGCTTAAAGAACATGCT

CTTTTGATTATCCATAAAACCAACGCTCCCATGTTTTTAAAAATGCTTAAAATTTTTGGA

CTTTTAAGCCAAGTGCACCATGACGATGTGTTAAAGATTTTAGAAAAAATACTTCAAAAT

>HP11032

GTGAGTTTGATTAAAGTTAGTGGTGATAAAAAAGTAATTGGGGTTTCCATTCCTTTAACT

------------TCAATTTCAGGCAAAGCGCGTGTGAAAATCAGACATGCCTTTAGCGAT

TGTGGTATTTCAACAGCGACTAGAAAAATCCCTTTTAGTTTAAAGCATTATGTAGAGTGG

CAGATCGGTTATGATGTCCCCATTAAAGATAAAGAA---AAATTTGAACTCACTACTTTA

AAAGATGAAAAATATCATTTTTTAGGGGCTAATAATAAAGTAAAAACTCTTTATGAATTG

AGTGAAATGATTGATTACGCTAAGCAATTAGGTTTAATCAGT---------TTAGAAAAT

TTAGAAAATACTTTAAAATATTTAGAAAAACAAAAACAATTTATAGAAGATAATTTTATG

ATTACAAGAGAAAGATTTAGATCGCATCAATTTGGTGGCATGGATTTTGAACTCTCACGC

ATTTCTTATCCCTTACTCATTCATTCTTTTAATGATAATGAGTTGAGCGAAATAGTTATT

AGAGAGCAACAATACGGCTCTAAAACCCAAGCCATG---CTGTATTTTTGCGTTTCTATT

TTGGAATTAAAAACCGCTACTCCCTTATTAAAAAGAACCGCTGCCCTCAAAGAACATGCT

CTTTTGATTATCCATAAAACCAACGCTCCCATGTTTTTGGAAATGCTTAAAATTTTTGGA

CTTTTAAGCCAAGCGCACCATAACGATGTGTTAAAGATTTTAGAAAAAATACTTCAAAAT

>CHL5

GTGAGTTTGATTAAAATTAACCATGATGAAAAAGTGATTGGGATTTTTATTCCTTTAACT

------------TCAATTTCAGGCAAAGTGCGTGTGAAAATCAGACATGCCTTTAGTGAT

TATGGTATTTCAACAGCGACTAGAACAATCCCTTTTAGTTTAAAACATTATGTAGAGTGG

CAGATCGGTTATGATGTCCCCATTAAAGATAAAGAA---AAATTTGAACTCACTACTTTA

AAAGATGAAAAATATCATTTTTTAGGGGCCAATAATAAAGTAAAAACTCTTTATGAATTG

AGCGAGATCATTTACTATGCCAAACAATTAGGTTTAATCAGT---------TTAGAAAAT

TTAGAAAATACTTTAAAATATTTAGAAAAACAAAAACAATTTATAGAAGATAATTTTATG

ATTACAAGAGAAAGATTTAGATCGCATCAATTTGGTGGCATGGATTTTGAACTTTCACGC

ATTTCTTATCCTTTACTCATTCATTCTTTTAATGATAATCAATTGAGCGAAATTGTTATT

AGAGAGCAACAATATGGTTCTAAAACCCAAGCTATG---CTGTATTTTTGCTTTTCTATT

TTGGAGTTAAAAACCGCTCCTCCCTTATTAAACAGAACGGCTATGCGCAAAGAACATGCT

CTTTTGATTATCCATAAAACCAACGCTCTCATGTTTTTAGAAATGCTTAAAATTTTTGGA

CTTTTAAGCCAAGCGCACCATGACGATGTGTTAAAGATTTTAGAAAAAATACTTCAAAAT

>HP42K

GTGAGTTTGATTAGGATTGATAATAATAAAAAAGTAATTGGGGTTTCCATTCCCTTAACT

------------TCAATTTCAGGCAAAGTGCGTGTGAAAATCAGACATGCCTTTAGCGAT

TATGGCATTTCAACAGCGACCAGAAAAATCCCTTTTAGTTTAAAACATTATGTAGAGTGG

CAAATCGGTTATGATGTCCCTATTAAAGATAAAGAA---AAATTTGAATTCACTACTTTA

AAAGATGAAAAATATCATTTTTTAGGGGATAATAATAAAGTAAAAACTCTTTATGAATTG

AGTGAAATGATTGATTACGCTAAGCAATTAAATTTAATCAGT---------TTAGAAAAT

TTAGAAAATACTTTAAAATATTTAGAAAAACAAAAACAATTTATAGAAGATAATTTTATA

------AGAGAAAGATTTAGATCGCATCAATTTGGTGGCATGGATTTTGAACTCTCACGC

ATTTCTTATCCTTTGCTCATTCATTCTTTTAATAATAATCAATTGAGTGAAATCGTTATT

AGAGAGCAACAATATGGCTCTAAAACCCAAGCCATG---CTGTATTTTTGCTTTTCTATT

TTGGAATTAAAAACCGCTACCCCCTTATTAAATAGAACCGCTACACTCAAAGAACATGCC

CTTTTAACTATCCATAAAACCAACGCTCTTGTGTTTTTAGAAATGCTTAAAATTTTTGGA

CTTTTAAGCCAAGTGCACCATAACGATGTGTTAAAGATTTTAGAAAAAATACTTCAAAAT

>ASHA-003

GTGAGTTTGATTAAAGTTAATGATGATAAAAAAGCGATTGAGGTTTCTATTCTTTTAACT

------------TCCATTTCAGGCAAAGCACGTGTGAAAATCAGACATGCCTTTAGCGAT

TATGGCATTTCAACAGCGACTAGAAAAATCCCTTTCAGTTTAAAACATTATGTAGAGTGG

CAAATCGGTTATGATGTCCCCATTGAAGATAAAGAA---AAATTTGAGCTCACTACCCTA

AAAGATGAAAAATATCATTTTTTAGGGGCTAATAATAAAGTAAAAACCCTTTATGAATTG

AGCGAAATAATTGATTACGCTAAGCGATTGGGTTTAATCAGT---------TTAGAAAAT

TTAGAAAATACTTTAAAATATTTAGAAAAACAAAAACAATTTATAGAAGATAGTTTTATG

ATTACAAGAGAAAGATTTAGATCGCATCAATTTGGTGGCATGGATTTTGAACTTTCACGC

ATTTCTTACCCTTTACTCATTCATTCTTTTAATGATAACCAATTGAGCGAAATCGTTATT

AGAGAGCAACAATATGGCTCTAAAACCCAAGCCATG---CTGTATTTTTGCTTTTCTATT

CTGGAATTAAAAACCGCTACACCCTTATTAAATAGAACGGCTGCACTCAAAGAACATGCT

CTTTTAATTATCCATAAAACCAACGCTCTTGTGTTTTTAGAAATGCTTAAAATTTTTGGT

CTCTTAAGCCAAGCGCATCATAACGATGTGTTAAAGATTTTAGAAAAAATACTTGAAAAT

>M-Mx-2006-276

GTGAGTTTGATTAGGATTGATGATAGTAAAAAAGCGATTGAGGTTTATGTTCCTTTAACT

------------TCCATTTCAGGCAAAGTGCGTGTGAAAATCAGACATGCCTTTAGCGAT

TATGGCGTTTCAACAGCGACTAGAAAAATCCCTTTTAGTTTAAAGCATTATGTAGAGTGG

CAAATCGGTTATAATGCCCCCATTAAAGATAAAGAA---AAATTTGAGCTCACTACCCTA

AAAGATGAAAAATATCATTTTTTAGGGGCTAATAATAAAGTGAAAACTCTTTATGAATTA

AGCGAAATAATTGATTACGCTAAGCAATTAGGTTTAATCAGT---------TTAGAAAAT

TTAGAAAATACTTTAAAATATTTAGAAAAACAAAAACAATTTATAGAAGATAATTTCACG

ATTACAAGAGAAAGATTTAGATTACATCAATTTGGTGGCATGGATTTTGAACTTTCACGC

ATTTCTTATCCTTTACTCATTCGTTCTTTCAACGATAATCAGTTGAGCGAAATTATTATT

AGAGAGCAACAATATGGCTCTAAAACCCAAGCCATG---CTGTATTTTTGCTTTTCTATT

TTGGAGTTAAAAACCGCTACCCCTTTATTAAATAGAACGGCTGCACTCAAAGAACATGCT

TTTTTAATTATCCATAAAACCAACGCTCTCATGTTTTTAGAAATGCTTAAAATTTTTGGG

CTTTTAAGCCAAGCGCACCATAACGATGTGTTAAAGATTTTAGAAAAAATACTTCAAAAT

>1089/03

------------------------------AAAGCGATTGAGGTTTCTATTCCTTTAACT

------------TCAATTTCAGGCAAAGTGCGTGTGAAAATCAGGCATGCCTTTAGCGAT

TATGGCATTTCAACAGCGACTAGAAAAATCCCTTTCAGTTTAAAGCATTATGTAGAGTGG

CAAATCGGCTATGATGTCCCCATTAAAGATAAAGAA---AAATTGGAACTCACTACCCTA

AAAGATGAAAAATATCATTTTTTAGGGGCTAATAATAAAGTAAAAACCCTTTATGAATTG

AGCGAAATAATTTATTACGCTAAGCGATTGGGTTTAATCAGT---------TTAGAAAAT

TTAGAAAATACTTTAAAATATTTAGAAAAACAAAAACAATTCATAGAAGATAATTTTATG

ATTACAAGAGAAAGATTTAGATCGCATCAATTTGGGGGCATGGATTTTGAACTTTCACGC

ATCTCTTATCCCTTACTCATTCATTCTTTCAACGATAACCAATTGAGCGAAATCGTTATT

AGAGAGCAACAATACGGCTCTAAAGTCCAAGCCATG---CTGTATTTTTGCTTTTCTATT

TTGGAATTAAAAACCGCTACCCCTTTATTAAATAGAACCGCTACACTCAAAGAACATGCC

CTTTTAACTATCCATAAAACCAACGCTCTTATGTTTTTAGAAATGCTTAAAATTTTTGGG

CTTTTAAGCCAAGCACACCATAGCGATGTGTTAAAGATTTTAGAAAAAATACTTCAAAAT

>1152/04

------------------------------AAAGCGATTGAGGTTTCTATTCCTTTAACT

------------TCAATTTCAGGCAAAGTGCGTGTGAAAATCAGGCATGCCTTTAGCGAT

TATGGCATTTCAACAGCGACTAGAAAAATCCCTTTCAGTTTAAAGCATTATGTAGAGTGG

CAAATCGGCTATGATGTCCCCATTAAAGATAAAGAA---AAATTGGAACTCACTACCCTA

AAAGATGAAAAATATCATTTTTTAGGGGCTAATAATAAAGTAAAAACCCTTTATGAATTG

AGCGAAATAATTTATTACGCTAAGCGATTGGGTTTAATCAGT---------TTAGAAAAT

TTAGAAAATACTTTAAAATATTTAGAAAAACAAAAACAATTCATAGAAGATAATTTTATG

ATTACAAGAGAAAGATTTAGATCGCATCAATTTGGGGGCATGGATTTTGAACTTTCACGC

ATCTCTTATCCCTTACTCATTCATTCTTTCAACGATAACCAATTGAGCGAAATCGTTATT

AGAGAGCAACAATACGGCTCTAAAGTCCAAGCCATG---CTGTATTTTTGCTTTTCTATT

TTGGAATTAAAAACCGCTACCCCTTTATTAAATAGAACCGCTACACTCAAAGAACATGCC

CTTTTAACTATCCATAAAACCAACGCTCTTATGTTTTTAGAAATGCTTAAAATTTTTGGG

CTTTTAAGCCAAGCACACCATAGCGATGTGTTAAAGATTTTAGAAAAAATACTTCAAAAT

>MKM6

GTGAGTTTGATTAAAATTAACCATGATGAAAAAGTGATTGAGGTTTCTATTCCTTTAACT

------------TCAATTTCAGGCAAAGTGCGTGTGAAAATCAGACATGCCTTTAGCGAT

TATGGTGTTTCAACAGCGACTAGAACAATCCCTTTTAGTTTAAAACATTATGTAGAGTGG

CAAATCGGTTATGATGTCCCCATTAAAGATAAAGAA---AAATTTGAACTCACTACTTTA

AAAGATAAAAAATATCATTTTTTAGGGGCTAATAATAAAGTAAAAACTCTTTATGAATTG

AGCGAGATCATTTACTATGCCAAGCAATTAGATTTAATCAGT---------TTAGAAAAT

TTAGAAAATACTTTAAAATATTTAGAAAAACAAAAACAATTTATAGAAGATAATTTTATG

ATTACAAGAGAAAGATTTAGATCGCATCAATTTGGTGGCATGGATTTTGAACTCTCACGC

ATTTCTTATCCTTTACTCATTCATTCTTTTAATGATAATCAATTGAGCGAAATTGTTATT

AGAGAACAACAATATGGTTCTAAAACCCAAGCTATG---CTGTATTTTTGCTTTTCTATT

TTGGAATTAAAAACCGCTACTCCCTTATTAAACAGAACGGCTACGCCCAAAGAACATGCT

CTTTTGATTATCCATAAAACCAACGCTCCTATGTTTTTAGAAATGCTTAAAATTTTTGGA

CTTTTAAGCCAAGCGCACCATGACGATGCGTTAAAGATTTTAGAAAAAATACTTCAAAAT

>KH0129

GTGAGTTTGATTAAAATTAACCATGATGAAAAAGTGATTGGGATTTTTATTCCTTTAACT

------------TCAATTTCAGGCAAAGTGCGTGTGAAAATCAGACATGCCTTTAGCGAT

TATGGTGTTTCAACAGCGACTAGAACAATCCCTTTTAGTTTAAAACATTATGTAGAGTGG

CAAATCGGTTATGATGTCCCCATTAAAGATAAAGAA---AAATTTGAACTCACTACTTTA

AAAGATGAAAAATATCATTTTTTAGGGGCTAATAATAAAGTAAAAACTCTTTATGAATTG

AGCGAGATCATTTACTATGCCAAGCAATTAGGTTTAATCAGT---------TTAGAAAAT

TTAGAAAATACTTTAAAATATTTAGAAAAACAAAAACAATTTATAGAAGATAATTTTATG

ATTACAAGAGAAAGATTTAGATCGCATCAATTTGGTGGCATGGCTTTTGAACTTTCACGC

ATTTCTTATCCTTTACTCATTCATTCTTTTAATGATAATCAATTGAGTGAAATTGTTATT

AGAGAACAACAATACGGCTCTAAAACCCAAGCCATG---CTGTATTTTTGCTTTTCTATT

TTGGAATTAAAAACCGCTACTCCCTTATTAAACAGAACGGCTACGCTCAAAGAGCATGCC

CTTTTAACTATCCATAAAACCAACGCTCTTGTGTTTTTAAAAATGCTTAAAATTTTTGGA

CTTTTAAGCCAAGTGCACCATAACGATGTGTTAAAGATTTTAAGAAAAATACTTCAAAAT

>MKF3

GTGAGTTTGATTAAAATTAACCATGATGAAAAAGTGATTGGGATTTTTATTCCTTTAACT

------------TCAATTTCAGGCAAAGTGCGTGTGAAAATCAGACATGCCTTTAGTGAT

TATGGTATTTCAACAGCGACTAGAACAATCCCTTTTAGTTTAAAACATTATGTAGAGTGG

CAAATCGGTTATGATGTCCCCATTAAAGATAAAGAA---AAATTTGAACTCACTACTTTA

AAAGATAAAAAATATCATTTTTTAGGGGCTAATAATAAAGTAAAAACTCTTTATGAATTG

AGCGAGATTATTTACTATGCCAAGCAATTAGATTTAATCAGT---------TTAGAAAAT

TTAGAAAATACTTTAAAATATTTAGAAAAACAAAAACAATTTATAGAAGATAATTTTATG

ATTACAAGAGAAAGGTTTAGATCGCATCAATTTGGTGGCATGGATTTTGAACTTTCACGC

ATTTCTTATCCTTTACTCATTCATTCTTTTGATGATAATCAATTGAGCGAAATTGTTATT

AGAGAGCAACAATATGGTTCTAAAACCCAAGCTATG---CTGTATTTTTGCTTTTCTATT

TTGGAGTTAAAAACCGCTACTCCCTTATTAAACAGAACGGCTACGCTCAAAGAACATGCC

CTTTTGATTATCCATAAAACCAACGCTCCCATGTTTTTAGAAATGCTTAAAATTTTTGGA

CTTTTAAGCCAAGCACACCATGACGATGTGTTAAAGATTTTAGAAAAAATACTTCAAAAT

>HP14069

GTGAGTTTGATTAAAATTAACCATGATGAAAAAGTGATTGGGATTTTTATTCCTTTAACT

------------TCAATTTCAGGCAAAGTGCGTGTGAAAATCAGACATGCCTTTAGTGAT

TATGGTGTTTCAACAGCGACTAGAACAATCCCTTTTAGTTTAAAGCATTATGTAGAGTGG

CAAATCGGTTATGATGTCCCCATTAAAGATAAAGAA---AAATTTGAACTTACTACTTTA

AAAGATGAAAAATATCATTTTTTAGGGGCTAATAATAAAGTAAAAACTCTTTATGAATTG

AGCGAGATCATTTACTATGCCAAGCAGTTAGGTTTAATCAGT---------TTAGAAAAT

TTAGAAAATACTTTAAAATATCTAGAAAAACAAAAACAATTTATAGAAGATAATTTTATG

ATTACAAGAGAAAGATTTAGATCGCATCAATTTGGTGGCATGGATTTTGAACTTTCACGC

ATTTCTTATCCTTTACTCATTCATTCTTTTAATGATAATCAATTGAGCGAAATTGTTATT

AGAGAGCAACAATATGGTTCTAAAACCCAAGCTATG---CTGTATTTTTGTTTTTCTATT

TTGGAGTTAAAAACCGCTCCTCCCTTATTAAACAGAACGGCTACGCTCAAAGAACATGCC

CTTTTGATTATCCATAAAACCAACGCTCCCATGTTTTTAGAAATGCTTAAAATTTTTGGA

CTTTTAAGCCAAGCGCACCATGACGATGTGTTAAAGATTTTAGAAAAAATACTTCAAAAT

>Col_29-PUJ

GTGAGTTTGATTAGGATTGATGATAGTAAAAAAGCGATTGAGGTTTCCATTCCTTTAACT

------------TCAATTTCAGGCAAAGTGCGTGTGAAAATCAGACATGCCTTTAGCGAT

TATGGTATTTCAACAGCGACTAGAAAAATCCCTTTTAGTTTAAAACATTATGTAGAGTGG

CAGATCGGTTATGATGTCCCCATTAAAGATAAAGAA---AAATTTGAACTCACTACTTTA

AAAGATGAAAAATATCATTTTTTAGGGGCTAATAATAAAGTAAAAACTCTTTATGAATTG

AGCGAAATGATTGATTGCGCTAAGCAATTAGATTTAATCAGT---------TTAGAAAAT

TTAGAAAATACTTTAAAATATTTAGAAAAACAAAAACAATTTATAGAAGATAATTTTATA

------AGAGAAAGATTTAGATTGCATCAATTTGGTGACATGGATTTTGAACTTTCACGC

ATTTCTTATCCTTTACTCATTCATTCTTTTAATGATAATCAGTTGAGTGAAATTGTTATT

AGGGAACAACAATACGGCTCTAAAACCCAAGCCATG---CTGTATTTTTGCTTTTCTATT

TTGGAGTTAAAAACCGCTACTCCCTTATTAAATAGAACGGCTGCACTCAAAGAACATGCC

CTTTTAACTATCCATAAAACCAACGCTCTTATGTTTTTAGAAATGCTTAAAATTTTTGGA

CTTTTAAGCCAAGCGCACCATAACGATGTGTTAAAGATTTTAGAAAAAATACTTCAAAAT

>HP11037

GTGAGTTTGATTAGGATTGATAATGATAAAAAAGTGATTGAGATTTCTGTTCCTTTAACT

------------TCAATTTCAGGCAAAGTGCGTGTGAAAATCAGGCATGCCTTTAGCGAT

TATGGCATTTCAACAGCGACCAGAAAAATCCCTTTTAGTTTAAAGCATTATGTAGAGTGG

CAAATCGGTTATGATGTCCCCATTAAAGATAAAGAA---AAATTTAAACTCACTACTTTA

AAAGATGAAAAATACCATTTTTTAGGGGCTAATAATAAAGTAAAGACTCTTTATGAATTG

AGCGAAATGATTTATTACGCTAAGCAATTAGATTTAATCAGT---------TTAGAAAAT

TTAGAAAATACTTTAAAATATTTAGAAAAACAAAAACAATTTATAGAAGATAATTTTATG

ATTACAAGAGAAAGATTTAGATTGCATCAATTTGGTGGCATGGATTTTGAACTCTCACGC

ATTTCTTATCCTTTGCTCATTCATTCTTTTAATGATAATCAATTGAGCGAAATTGTTATT

AGGGAACAACAATATGGTTCTAAAACCCAAGCCATG---CTGTATTTTTGCTTTTCTATT

TTGGAATTAAAAACCGCTACCCCCTTATTAAATAGAACGGCTGCACTCAAAGAACATGCC

CTTTTAACCATCCATAAAACCAACGCTCTTATGTTTTTAGAAATGCTTAAAATTTTTGGA

CTTTTAAGCCAAGCGCACCATAACGAGTGT------------------------------

>UM163R

GTGAGTTTGATTAAAATTAACCATGATGAAAAAGTGATTGAGGTTTCTATTCCCTTAACT

------------TCAATTTCAGGCAAAGTGCGTGTGAAAATCAGACATGCCTTTAGCGAT

TATGGTATTTCAACAGCGACTAGAACAATCCCTTTTAGTTTAAAACATTATGTGGAGTGG

CAGATCGGTTATGATGTCCCTATTAAAGATAAAGAA---AAATTTGAACTCACTACTTTA

AGAGATGAAAAATATCATTTTTTAGGGGCTAATAATAAAGTAAAAACTCTTTATGAATTG

AGCGAGATCATTTACTATGCCAAGCAATTAGGTTTAATCAGT---------TTAGAAAAT

TTAGAAAATACTTTAAAATATTTAGAAAAACAAAAACAATTTATAGATTTTAATTTTATG

ATTACAAGAGAAAGATTTAGATCGCATCAATTTGGTGGCATGGATTTTGAACTTTCACGC

ATTTCTTATCCTTTACTCATTCATTCTTTTAATGATAATCAATTGAGCGAGATTGTTATT

AGAGAACAACAATATGGTTCTAAAACCCAAGCCATG---CTGTATTTTTGCTTTTCTATT

TTGGAGTTAAAAACCGCTACTCCCTTATTAAATAGAACGGCTACGCCCAAAGAACATGCT

CTTTTGATTATCCATAAAACCAACGCTCCCATGTTTTTAGAAATGCTTAAAATTTTTGGA

CATTTAAGCCAAGCACACCATAACGATGTGTTAAAGATTTTAGAAAAGATACTCCAAAAT

>ZH48

GTGAGTTTGATTGAAATTAACCATGATGAAAAAGTGATTGAGGTTTCTATTCCCTTAACT

------------TCAATTTCAGGCAAAGTGCGTGTGAAAATCAGACATGCCTTTAGTGAT

TATGGTGTTTCAACAGCGACTAGAACAATCCCTTTTAGTTTAAAACATTATGTAGAGTGG

CAAATCGGTTATGATGTCCCCATTAAAGATAAAGAA---AAATTTGAACTCACTACTTTA

AAAGATAAAAAATATCATTTTTTAGGGGCTAATAATAAAGTAAAAACCCTTTATGAATTG

AGCGAGATCATTTACTATGCCAAGCAATTAGATTTAATCAGT---------TTAGAAAAT

TTAGAAAATACTTTAAAATATTTAGAAAAACAAAAACAATTTATAGAAGATAATTTTATG

ATTACAAGAGAAAGATTTAGATCGCATCAATTTGGTGGCATGGATTTTGAACTTTCACAC

ATTTCTTATCCTTTACTCATTCATTCTTTTAATGATAATCAATTGAGCGAAATTGTTATT

AGAGAACAACAATATGGTTCTAAAACCCAAGCTATG---CTGTATTTTTGCTTTTCTATT

TTGGAATTAAAAACCGCTACTCCCTTATTAAACAGAACGGCTACGCTCAAAGAACATGCT

CTTTTGATTATCCATAAAACCAACGCTCCCATGTTTTTAAAAATGCTTAAAATTTTTGGA

CTTTTAAGCCAAGCACACCATGACGATGTGTTAAAGATTTTAGAAAAAATACTTCAAAAT

>HP15027

GTGAGTTTGATTAAAGTTAGTGGTGATAAAAAAGCGATTGAGGTTTCCATTCCTTTAACT

------------TCAATTTCAGGCAAAGTGCGTGTGAAAATCAGACATGCTTTTAGCGAT

TATGGTATTTCAACAGCGACTAGAAAAATCCCTTTTAGTTTAAAACATTATGTAGAGTGG

CAGATCGGTTATGATGTCCCCATTAAAGATAAAGAA---AAATTTGAACTCACTACTTTA

AAAGATGAAAAATATCATTTTTTAGGGGCTAATAGTAAAACAAAAACTCTTTATGAATTG

AGTGAAATAATCTATTACGCTAAGCAATTAAATTTAATCAGT---------TTAGAAAAT

TTAGAAAATACTTTAAAATATTTAGAAAAACAAAAACAATTTATAGAAGATAATTTCACG

ATTACAAGAGAAAGATTTAGATCGCATCAATTTGGGGGCATGGATTTTGAACTTTCACGC

ATTTCTTATCCCTTACTCATTCATTCTTTTAATGATAATCAATTGAGCGAAATCGTTATT

AGAGAGCAACAATACGGCTCTAAAACCCAAGCCATG---CTGTATTTTTGCTTTTCTATT

TTGGAATTAAAAACCGCTACTCCCTTATTAAACAGAACGGCTACGCCCAAAGAACATGCT

CTTTTGATTATCCATAAAACCAACGCTCCCATGTTTTTAGAAATGCTTAAAATTTTTGGA

CTTTTAAGCCAAGTGCACCATGACGATGTGTTAAAGATTTTAGAAAAAATACTTCAAAAT

>F67

GTGAGTTTGATTAAAATTAACCATGATGAAAAAGTGATTGGGATTTTTATTCCTTTAACT

------------TCAATTTCAGGCAAAGTGCGTGTGAAAATCAGACATGCCTTTAGCGAT

TATGGTGTTTCAACAGCGACTAGAACAATCCCTTTTAGTTTAAAACATTATGTAGAGTGG

CAAATCGGTTATGATGTCCCCATTAAAGATAAAGAA---AAATTTGAACTCACTACTTTA

AAAGATGAAAAATATCATTTTTTAGGGGCTAATAATAAAGTAAAAACTCTTTATGAATTG

AGCGAGATCATTTATTATGCCAAACAATTAGGTTTAATCAGT---------TTAGAAAAT

TTAGAAAATATTTTAAAATATTTAGAAAAACAAAAACAATTTATAGAAGATAATTTTATG

ATTACAAGAGAAAGATTTAGATCGCATCAATTTGGTGGCATGGATTTTGAACTTTCACGC

ATTTCTTATCCTTTACTCATTCATTCTTTTGATGATAATCAATTGAGCGAAATAGTTATT

AGAGAGCAACAATATGGTTCTAAAACCCAAGCTATG---CTGTATTTTTGCTTTTCTATT

TTGGAGTTAAAAACCGCTACTCCCTTATTAAACAGAACGGCTATGCGCAAAGAACATGCT

CTTTTGATTATCCATAAAACCAACGCTCCCATGTTTTTAGAAATGCTTAAAATTTTTGGA

CTTTTAAGCCAAGCGCACCATGACGATGTGTTAAAGATTTTAGAAAAAATACTTCAAAAT

>G-Mx-2011-131

GTGAGTTTGATTAAAGTTAATGATGATAAAAAAGTGATTGAGGTTTCTATTCCTTTAACT

------------TCAATTTCAGGCAAAGCGCGTGTGAAAATCAGACATGCCTTTAGCGAT

TATGGCATTTCAACAGCGACCAGAAAAATCCCTTTTAGTTTAAAGCATTATGTAGAGTGG

CAAATCGGTTATGATGTCCCTATTAAAGATAAAGAA---AAATTTAAACTCACTACTTTA

AAAGATGAAAAATACCATTTTTTAGGGGCCAATAATAAAGTAAAAACCCTTTATGAATTA

AGCGAAATAATTGATTACGCTAAGCAATTAAATTTAATCAGT---------TTAGAAAAT

TTAGAAAATACTTTAAAATATTTAGAAAAACAAAAACAATTCATAGAAGATAGTTTCACG

ATTACAAGAGAAAGATTTAGATCGCATCAATTTGGTGGCATGGATTTTGAACTCTCACGC

ATTTCTTATCCTTTACTCATTCATTCTTTTAACGATAATCAATTGAGCGAAATCGTTATT

AGAGAACAACAATACGGCTCTAAAACCCAAGCCATG---CTGTATTTTTGCTTTTCTATT

TTGGAGTTAAAAACCGCTACCCCCTTATTAAACAGAACGGCTATGCTCAAAGAGCATGCT

CTTTTGATTATCCATAAAACCAACGCTCCCATGTTTTTAGAAATGCTTAAAATTTTTGGA

CTTTTAAGCCAAGCGCACCACAACGATGTGTTAAAGATTTTAGAAAAAATACTTGAAAAT

>CHL6

GTGAGTTTGATTGAAATTAACCATGATGAAAAAGTGATTGAAATCCCTATTCCTCTCACT

------------TCAATTTCAGGCAAAGTGCGTGTGAAAATCAGACATGCCTTTAGCGAT

TATGGTGTTTCAACAGCGACTAGAACAATCCCTTTTAGTTTAAAGCATTATGTAGAGTGG

CAGATCGGTTATGATGTCCCCATTGAAAATAAAGAA---AAATTTGAACTCACTACTTTA

AAAGATGAAAAATATCATTTTTTAGGGGCTAATAATAAAGTAAAAACTCTTTATGAATTG

AGCGAAATGATTTATTACGCTAAGCAATTAGGTTTAATCAGT---------TTAGAAAAT

TTAGAAAATACTTTAAAATATTTAGAAAAACAAAAACAATTTATAGAAGATAATTTTATG

ATTACAAGAGAAAGATTCAGATCGCATCAATTTGGTGGCATGGATTTTGAGCTTTCACAC

ATTTCTTACCCTTTACTCATTCATTCTTTTAATGATAATCAATTGAGCGAAATTGTTATT

AGAGAACAACAATATGGTTCTAAAACCCAAGCCATG---CTGTATTTTTGCTTTTCTATT

TTGGAGTTAAAAACCGCTACTCCCTTATTAGACAGAACGGCTACGCTCAAAGAACATGCT

CTTTTGATTATCCATAAAACCAACGCTCCCATGTTTTTAGAAATGCTTAAAATTTTTGGA

CTTTTAAGCCAAGCGCACCATGACGATGTGTTAAAGATTTTAGAAAAAATACTTCAAAAT

>H30A

GTGAGTTTGATTAAAATTAACCATGATGAAAAAGTGATTGGGATTTTTATTCCTTTAACT

------------TCAATTTCAGGCAAAGTGCGTGTGAAAATCAGACATGCCTTTAGCGAT

TATGGTGTTTCAACAGCGACTAGAAAAATCCCTTTTAGTTTAAAACATTATGTAGAGTGG

CAAATCGGTTATGATGTCCCCATTAAAGATAAAGAA---AAATTTGAGCTCACTACCCTA

AAAGATGAAAAATATCATTTTTTAGGGGCTAATAATAAAGTAAAAACTCTTTATGAATTG

AGCGAAATGATTTATTACGCTAAGCAATTAAATTTAATCAGT---------TTAGAAAAT

TTAGAAAATACTTTAAAATATTTAGAAAAACAAAAACAATTTATAGAAGATAATTTTATG

ATTACAAGAGAAAGATTTAGATCGCATCAATTTGGTGGCATGGATTTTGAACTTTCACGC

ATTTCTTATCCTTTACTCATTCATTCTTTTAATGATAATCAATTGAGCGAAATTGTTATT

AGAGAGCAACAATATGGTTCTAAAACCCAAGCCATG---CTGTATTTTTGCTTTTCTATT

TTGGAGTTAAAAACCGCTCCTCCCTTATTAAACAGAACGGCTACGCCCAAAGAACATGCT

CTTTTGATTATCCATAAAACCAACGCTCCCATGTTTTTAGAAATGCTTAAAATTTTTGGA

CTTTTAAGCCAAGCGCACCATGACGATGTGTTAAAGATTTTAGAAAAGATACTTCAAAAT

>H30

GTGAGTTTGATTAAAATTAACCATGATGAAAAAGTGATTGGGATTTTTATTCCTTTAACT

------------TCAATTTCAGGCAAAGTGCGTGTGAAAATCAGACATGCCTTTAGCGAT

TATGGTGTTTCAACAGCGACTAGAAAAATCCCTTTTAGTTTAAAACATTATGTAGAGTGG

CAAATCGGTTATGATGTCCCCATTAAAGATAAAGAA---AAATTTGAGCTCACTACCCTA

AAAGATGAAAAATATCATTTTTTAGGGGCTAATAATAAAGTAAAAACTCTTTATGAATTG

AGCGAAATGATTTATTACGCTAAGCAATTAAATTTAATCAGT---------TTAGAAAAT

TTAGAAAATACTTTAAAATATTTAGAAAAACAAAAACAATTTATAGAAGATAATTTTATG

ATTACAAGAGAAAGATTTAGATCGCATCAATTTGGTGGCATGGATTTTGAACTTTCACGC

ATTTCTTATCCTTTACTCATTCATTCTTTTAATGATAATCAATTGAGCGAAATTGTTATT

AGAGAGCAACAATATGGTTCTAAAACCCAAGCCATG---CTGTATTTTTGCTTTTCTATT

TTGGAGTTAAAAACCGCTCCTCCCTTATTAAACAGAACGGCTACGCCCAAAGAACATGCT

CTTTTGATTATCCATAAAACCAACGCTCCCATGTTTTTAGAAATGCTTAAAATTTTTGGA

CTTTTAAGCCAAGCGCACCATGACGATGTGTTAAAGATTTTAGAAAAGATACTTCAAAAT

>CM22046

GTGAGTTTGATTAAGATTGATGATGATAAAAAAGCGATTGAGGTTTCTATTCCTTTAACT

------------TCCATTTCAGGCAAAGCGCGTGTGAAAATCAGACATGCCTTTAGCGAT

TATGGCATTTCAACAGCGACTAGAAAAATCCCTTTCAGTTTAAAACATTATGTAGAGTGG

CAAATCGGTTATGATGTCCCCATTAAAGATAAAGAA---AAATTTGAACTCACTACCCTA

AAAGATGAAAAATATCATTTTTTAGGGGCTAATAATAAAGTAAAAACCCTTTATGAATTG

AGTGAGATAATCTATTACGCTAAGCAATTAAATTTAATCAGT---------TTAGAAAAT

TTAGAAAATACTTTAAAATATTTAGAAAAACAAAAACAATTTATAGAAGATAATTTT---

---ATAAGAGAAAGATTTAGATTACATCAATTTGGTGACATGGATTTTGAACTTTCACGC

ATTTCTTATCCTTTACTCATTCATTCTTTTAATGATAATCAATTGAGTGAAATCGTTATT

AGAGAGCAACAATATGGCTCTAAAACCCAAGCCATG---CTGTATTTTTGCTTTTCTATT

TTGGAGTTAAAAACCGCTACTCCCTTATTAAATAGAACGGCTGCACTCAAAGAACATGCC

CTTTTAACTATCCATAAAACCAACGCTCTTGTGTTTTTAGAAATGCTTAAAATTTTTGGA

CTTTTAAGCCAAGCGCACCATAGCGATGTGTTAAAGATTTTAGAAAAAATACTTCAAAAT

>B24

GTGAGTTTGATTAAAGTTAGTGGTGATAAAAAAGTGATTGAGTTTTCTATTCCTTTAACT

------------TCAATTTCAGGCAAAGCGCGTGTGAAAATCAGACATGCTTTTAGCGAT

TATGGTATTTCAACAGCGACTGGAAAAATCCCTTTTAGTTTAAAACATTATGTAGAGTGG

CAGATCGGTTATGATGTCCCCATTAAAGATAAAGAA---AAATTTGAACTCACTACTTTA

AAAGATGAAAAATATCATTTTTTAGGGGCTAATGATAAAGTAAAAACCCTTTATGAATTG

AGTGAGATAATCTATTACGCTAAGCAATTAAATTTAATCAGT---------TTAGAAAAT

TTAGAAAATACTTTAAAATATTTAGAAAAACAAAAACAATTTATAGAAGATAATTTCACG

ATTACAAGAGAAAGATTTAGATTACATCAATTTGGTGGCATGGGTTTTGAACTCTCACGC

ATCTCTTATCCTTTACTCATTCATTCTTTTAATGATAATCAGTTGAGCGAAATCGTTATT

AGAGAGCAACAATATGGCTCTAAAACCCAAGCCATG---CTGTATTTTTGCTTTTCTATT

TTGGAGTTAAAAACCGCTACCCCCTTATTAAATAGAACCGCTACACTCAAAGAACATGCT

TTTTTAACCATCCATAAAACCAACGCTCCCATGTTTTTAGAAATGCTTAAAATTTTTGGA

CTTTTAAGCCAAGCGCACCATAACGATGTGTTAAAGATTTTAGAAAAAATACTTCAAAAT

>D1801435

GTGAGTTTGATTAAAATTAACCATGATGAAAAAGTGATTGGGATTTTTATTCCTTTAACT

------------TCAATTTCAGGCAAGGTGCGTGTGAAAATCAGACATGCCTTTAGCGAT

TATGGTATTTCAACAGCGACTAGAACAATCCCTTTTAGTTTAAAACATTATGTAGAGTGG

CAAATCGGTTATGATGTCCCCATTAAAGATAAAGAA---AAATTTGAACTCACTACTTTA

AAAGATGAAAAATATCATTTTTTAGGGGCTAATAATAAAGTAAAAACGCTTTATGAATTG

AGCGAGATCATTTACTATGCCAAGCAATTAGGTTTAATCAGT---------TTAGAAAAT

TTAGAAAATACTTTAAAATATTTAGAAAAACAAAAACAATTTATAGAAGATAATTTTATG

ATTACAAGAGAAAGATTTAGATTACATCAATTTGGTGGCATGGATTTTGAACTCTCACGC

ATTTCTTATCCTTTGCTCATTCATTCTTTTAATGATAATGAGTTGAGCGAAATAGTTATT

AGAGAACAACAATATGGCTCTAAAACCCAAGCCATG---CTGTATTTTTGCTTTTCTATT

TTGGAGTTAAAAACCGCTACTCCCTTATTAAACAGAACGGCTGCACTCAAAGAACATGCC

CTTTTGATTGTCCGTCAAACTAACGCTTCCATGTTTTTAGAAATGCTTAAAATTTTTGGA

CTTTTAAGCCAAGCGCACCATAACGATGTGTTAAAGATTTTAGAAAAAATACTTCAAAAT

>22351

GTGAATTTGATTAAAGTTAATGATGATAAAAAAGCGATTGAGGTTTCTATTCCTTTAACT

------------TCCATTTCAGGCAAAGTGCATGTGAAAATCAGACATGCCTTTAGCGAT

TATGGTATTTCAACAGCGACTAGAAAAATCCCTTTCAGTTTAAAGCATTATGTAGAGTGG

CAAATCGGTTATGATGTCCCCATTAAAGATAAAGAA---AAATTTGAACTCACTACCCTA

AAAGATGAAAAATATCATTTTTTAGGGGCTAATAATAAAGTAAAAACCCTTTATGAATTG

AGTGAGATAATCTATTACGCTAAGCAATTAAATTTAATCAGT---------TTAGAAAAT

TTAGAAAATACTTTAAAATATTTAGAAAAACAAAAACAATTTATAGAAGATAATTTT---

---ACAAGAGAAAGATTTAGATCGCATCAATTTGGTGGCATGGATTTTGAACTCTCACGC

ATTTCTTATCCCTTACTCATTCATTCTTTTAATGATAATCAATTGAGCGAAATCGTTATT

AGAGAGCAACAATACGGCTCTAAAACCCAAGCCATG---CTGTATTTTTGCTTTTCTATT

TTGGAGTTAAAAACCGCTACCCCCTTATTAAACAGAACGGCTATGCTCAAAGAGCATGCT

CTTTTGATTATCCATAAAACCAACGCTCTCATGTTTTTAGAAATGCTTAAAATTTTTGGA

CTTTTAAGCCAAGTGCACCATAACGATGTGTTAAAGATTTTAGAAAAAATACTTCAAAAT

>C

GTGAGTTTGATTAAAATTAACCATGATGAAAAAGTGATTGAGGTTTTTATTCCTTTAACT

------------TCAATTTCAGGCAAAGTGCGTGTGAAAATCAGACATGCCTTTAGCGAT

TATGGTATTTCAACAGCGACTAGAACAATCCCTTTTAGTTTAAAACATTATATAGAGTGG

CAGATCGGTTATGATGTCCCCATTAAAGATAAAGAA---AAATTTGAACTCACTACTTTA

AAAGATGAAAAATATCATTTTTTAGGGGCTAATAATAAAGTAAAAACTCTTTATGAATTG

AGCGAAATGATTTATTACGCCAAGCAATTAGGTTTAATCAGT---------TTAGAAAAT

TTAGAAAATACTTTAAAATATTTAGAAAAACAAAAACAATTTATAGAAGATAATTTTATG

ATTATAAGAGAAAGATTTAGATCGCATCAATTTGGTGGCATGGATTTTGAACTTTCACAC

ATTTCTTATCCTTTACTCATTCATTCTTTTAATGATAATCAATTGAGCGAGATTGTTATT

AGAGAACAACAATATGGTTCTAAAACCCAAGCCATG---CTGTATTTTTGCTTTTCTATT

TTGGAGTTAAAAACCGCTACTCCCTTATTAAATAGAACGGCTACGCCCAAAGAACATGCT

CTTTTGATTATCCATAAAACCAACGCTCCCATGTTTTTAAAAATGCTTAAAATTTTTGGA

CTTTTAAGCCAAGCGCACCATGACGATGTGTTAAAGATTTTAGAAAAGATACTTCAAAAT

>F24

GTGAGTTTGATTAAAATTAACCATGATGAAAAAGTGATTGGGATTTTTATTCCTTTAACT

------------TCAATTTCAGGCAAAGTGCGTGTGAAAATCAGACATGCCTTTAGCGAT

TATGGTGTTTCAACAGCGACTAGAACAATCCCTTTTAGTTTAAAACATTATGTAGAGTGG

CAAATCGGTTATGATGTCCCCATTAAAGATAAAGAA---AAATTTGAACTCACTACTTTA

AAAGATGAAAAATATCATTTTTTAGGGGCTAATAATAAAGTAAAAACTCTTTATGAATTG

AGCGAGATCATTTATTATGCCAAACAATTAGGTTTAATCAGT---------TTAGAAAAT

TTAGAAAATACTTTAAAATATTTAGAAAAACAAAAACAATTTATAGAAGATAATTTTATG

ATTACAAGAGAAAGATTTAGATCGCATCAATTTGGTGGTATGGATTTTGAACTTTCACAC

ATTTCTTATCCTTTACTCATTCATTCTTTTAATGATAATCAATTGAACGAAATTGTTATT

AGAGAACAACAATATGGTTCTAAAACCCAAGCTATG---CTGTATTTTTGCTTTTCTATT

TTGGAATTAAAAACCGCTACTCCCTTATTAAACAGAACGGCTACGCTCAAAGAACATGCT

CTTTTGATTATCCATAAAACCAACGCTCCCATGTTTTTAGAAATGCTTAAAATTTTTGGA

CTTTTAAGCCAAGTGCACCATAACGATGTGTTAAAGATTTTAGAAAAAATACTTCAAAAT

>CHL11

GTGAGTTTGATTAAAGTTAGTGGTGATAAAAAAGCGATTGAGGTTTCTATTCCCTTAACT

------------TCAATTTCAGGCAAAGTGCGTGTGAAAATCAGACATGCCTTTAGCGAT

TATGGTATTTCAACAGCGACTAGAACAATCCCTTTTAGTTTAAAGCATTATGTAGAGTGG

CAGATCGGTTATGATGTCCCCATTAAAGATAAAGAA---AAATTTGAACTCACTACTTTA

AAAGATAAAAAATATCATTTTTTAGGGGCTAATAATAAAGTAAAAACTCTTTATGAATTG

AGCGAGATTATTTACTATGCCAAGCAATTAGATTTAATCAGT---------TTAGAAAAT

TTAGAAAATACTTTAAAATATTTAGAAAAACAAAAACAATTTATAGAAGATAATTTTATG

ATTACAAGAGAAAGATTTAGATCGCATCAATTTGGTGGCATGGATTTTGAACTTTCACGC

ATTTCTTATCCTTTACTCATTCATTCTTTTAATGATAATCAATTGAGCGAAATTGTTATT

AGAGAACAACAATATGGTTCTAAAACCCAAGCCATG---CTGTATTTTTGCTTTTCTACT

TTGGAGTTAAAAACCGCTACTCCCTTATTAAACAGAACGGCTACGCCCAAAGAACATGCT

CTTTTGATTATCCATAAAACCAACGCTCCCATGTTTTTAGAAATGCTTAAAATTTTTGGA

CTTTTAAGCCAAGCGCACCATGACGATGCGTTAAAGATTTTAGAAAAGATACTTCAAAAT

>3136

GTGAGTTTGATTAGGATTGATAATAATAAAAAAGTGATTGAGGTTTCCATTCCTTTAACT

------------TCAATTTCAGGCAAAGTGCGTGTGAAAATCAGACATGCCTTTAGCGAT

TATGGCATTTCAACAGCGACCAGAAAAATCCCTTTTAGTTTAAAACATTATGTAGAGTGG

CAAATCGGTTATGATGTCCCCATTAAAGATAAAGAA---AAATTTGAACTCACTACTTTA

AAAGATGAAAAATATCATTTTTTAGGGGCTAATAATAAAGTAAAAACCCTTTATGAATTG

AGTGAGATAATCTATTACGCTAAGCAATTAAATTTAATCAGT---------TTAGAAAAT

TTAGAAAATACTTTAAAATATTTAGAAAAACAAAAACAATTCATAGAAGATAATTTTATG

ATTACAAGAGAAAGATTTAGATCGCATCAATTTGGTGGCATGGGTTTTGAACTTTCACGC

ATTTCTTACCCTTTACTCATTCATTCTTTTAATGATAATCAATTGAGCGAAATCGTTATT

AGAGAGCAACAATATGGCTCTAAAACCCAAGCCATG---CTGTATTTTTGCTTTTCTATT

TTGGAGTTAAAAACCGCTACCCCTTTATTAAATAGAACCGCTGCACTCAAAGAACATGCT

TTTTTAATTATCCATAAAACCAACGCTCTTGTGTTTTTAGAAATGCTTAAAATTTTTGGA

CTTTTAAGCCAAGCGCACCATAACGAGTGT------------------------------

>CHL39

GTGAGTTTGATTAAAATTAACCATGATGAAAAAGTGATTGAAATTTCCATTCCTTTAACT

------------TCAATTTCAGGCAAAGTGCGTGTGAAAATCAGACATGCCTTTAGCGAT

TATGGTGTTTCAACAGCGACTAGAACAATCCCTTTTAGTTTAAAACATTATGTAGAGTGG

CAGATCGGTTATGATGTCCCCATTAAAGATAAAGAA---AAATTTGAACTCACTACTTTA

AAAGATGAAAAATATCATTTTTTAGGGGCTAATAATAAAGTAAAAACTCTTTATGAATTG

AGCGAGATTATTTACTATGCCAAGCAATTAGATTTAATCAGT---------TTAGAAAAT

TTAGAAAATACTTTAAAATATTTAGAAAAACAAAAACAATTTATAGAAGATAATTTTATG

ATTACAAGAGAAAGATTTAGATCACATCAATTTGGTGGCATGGATTTTGAACTTTCACGC

ATTTCTTATCCTTTACTCATTCATTCTTTTAATGATAATCAATTGAGCGAAATTGTTATT

AGAGAACAACAATATGGTTCTAAAACCCAAGCCATG---CTGTATTTTTGCTTTTCTATT

TTGGAGTTAAAAACCACTACTCCCTTGTTAAACAGAACGGCTACGCTCAAAGAACATGCT

CTTTTGATTATCCATAAAGCCAACGCTCCCATGTTTTTAGAAACGCTTAAAATTTTTGGA

CTTTTAAGCCAAGCGCACCATGACGATGTGTTAAAGATTTTAAAAAAAATACTTCAAAAT

>CHL50

GTGAGTTTGATTAAAGTTAGTGGCGATAAAAAAGTGATTGAGGTTTCTATTCCTTTAACT

------------TCAATTTCAGGCAAAGTGCGTGTGAAAATCAGACATGCCTTTAGCGAT

TATGGTATTTCAACAGCGACTAGAACAATCCCTTTTAGTTTAAAACATTATGTAGAGTGG

CAGATCGGTTATGATGTCCCCATTAAAGATAAAGAA---AAATTTGAACTCACTACTTTA

AAAGATAAAAAATATCATTTTTTAGGGACTAATAATAAAGTAAAAACTCTTTATGAATTG

AGCGAGATCATTTATTATGCCAAACAATTAGGTTTAATCAGT---------TTAGAAAAT

TTAGAAAATACTTTAAAATATTTAGAAAAACAAAAACAATTTATAGAAGATAATTTTATG

ATTACAAGAGAAAGATTTAGATCGCATCAATTTGGTGGCATGGATTTTGAACTTTCACAC

ATTTCTTATCCTTTACTCATTCATTCTTTTAATGATAATCAATTGAGCGAAATTGTTATT

AGAGAGCAACAATATGGTTCTAAAACCCAAGCCATG---TTGTATTTTTGCTTTTCTATT

TTGGAGTTAAAAACCGCTCCTCCCTTATTAAACAGAACGGCTACGCTCAAAGAACATGCT

CTTTTGATTATCCATAAAGCCAACGCTCCCATGTTTTTAAAAATGCTTAAAATTTTTGGA

CTTTTAAGCCAAGCACACCATGACGATGTGTTAAAGATTTTAGAAAAAATACTTCAAAAT

>HPAS14

GTGAGTTTGATTAAAGTTAGTAGTGATAAAAACGAGATTAAAGTTTCTATTCCTTTAACT

------------TCAATTTCAGGCAAAGTGCGTGTGAAAATCAGACATGCCTTTAGCGAT

TATGGTATTTCAATAGCGACCAGAAAAATCCCTTTTAGTTTAAAGCATTATGTAGAGTGG

CAAATCGGTTATGATGTCCCCATTAAAGATAAAGAA---AAATTTGAACTCACTACTCTA

AAAGATGAAAAACATCATTTTTTAGGGGCTAATGGTAAAACAAAAACTCTTTATGAATTG

AGCGAAATAATTTATTACGCTAAGCAATTAGGTTTAATCAGT---------TCAGGAAAT

TTAGAAAATACTTTAAAATATTTAGAAAAACAAAAACAATTTATAGAAGATAATTTTACG

ATTACAAGAGAAAGATTTAGATCGCATCAATTTGGCGGCATGGATTTTGAACTTTCACAC

ATTTCTTATCCTTTACTCATTCATTCTTTTAATGATAATCAGTTGAGTGAAATAGTTATT

AGAGAACAACAATATGGCTCTAAAACTCAAGCCATG---CTGTATTTTTGCTTTTCTATT

TTGGAGTTAAAAACCGCTACTCCTTTATTAAACAGAACGGCTGCGCTCAAAGAACATGCT

CTTTTAACTATCCATAAAACCAACGCTCTTATGTTTTTAGAAATGCTTAAAATTTTTGGA

CTTTTAAGCCAAGCGCACCATAACGATGTGTTAAAGATTTTAGAAAAAATCCTTCAAAAT

>ZH127

GTGAGTTTGATTAAAGTTAATGATGATAAAAAAGTGATTGAGGTTTCTATTCCTTTAACT

------------TCCACTTCAGGCAAAGCGCGTGTGAAAATCAGACATGCCTTTAGCGAT

TATGGCATTTCAACAGCGACTAGAAAAATCCCTTTCAGTTTAAAGCATTATGTAGAGTGG

CAAATCGGTTATGATGTCCCCATTAAAGATAAAGAA---AAATTTGAACTCACTACCCTA

AAAGATGAAAAATATCATTTTTTAGGGGCTAATAATAAAGTAAAGACTCTTTATGAATTG

AGCGAAATAATTGATTACGCTAAGCGATTGGGTTTAATCAGT---------TTAGAAAAT

TTAGAAAATACTTTAAAATATTTAGAAAAACAAAAACAATTTATAGAAGATAATTTTATA

------AGAGAAAGATTTAGATCGCATCAATTTGGTGGCATGGATTTTGAACTTTCACGC

ATTTCTTATCCTTTACTCATTCATTCTTTTAATGATAATCAGTTGAGCGAAATCGTTATT

AGAGAGCAACAATACGGCTCTAAAACCCAAGCCATG---CTGTATTTTTGCTTTTCTATT

CTGGAATTAAAAACCGCTACACCCTTATTAAATAGAACGGCTGCACTCAAAGAACACGCT

CTTTTAACTATCCATAAAACCAACGCTCTTGTGTTTTTAGAAATGCTTAAAATTTTTGGA

CTTTTAAGTCAATCACACCATAACGATGTGTTAAAGATTTTAGAAAAAATACTTGAAAAT

>ZH5

GTGAGTTTGATTAAAGTTAATGATGATAAAAAAGTGATTGAGGTTTCTATTCCTTTAACT

------------TCCACTTCAGGCAAAGCGCGTGTGAAAATCAGACATGCCTTTAGCGAT

TATGGCATTTCAACAGCGACTAGAAAAATCCCTTTCAGTTTAAAGCATTATGTAGAGTGG

CAAATCGGTTATGATGTCCCCATTAAAGATAAAGAA---AAATTTGAACTCACTACCCTA

AAAGATGAAAAATATCATTTTTTAGGGGCTAATAATAAAGTAAAGACTCTTTATGAATTG

AGCGAAATAATTGATTACGCTAAGCGATTGGGTTTAATCAGT---------TTAGAAAAT

TTAGAAAATACTTTAAAATATTTAGAAAAACAAAAACAATTTATAGAAGATAATTTTATA

------AGAGAAAGATTTAGATCGCATCAATTTGGTGGCATGGATTTTGAACTTTCACGC

ATTTCTTATCCTTTACTCATTCATTCTTTTAATGATAATCAGTTGAGCGAAATCGTTATT

AGAGAGCAACAATACGGCTCTAAAACCCAAGCCATG---CTGTATTTTTGCTTTTCTATT

CTGGAATTAAAAACCGCTACACCCTTATTAAATAGAACGGCTGCACTCAAAGAACACGCT

CTTTTAACTATCCATAAAACCAACGCTCTTGTGTTTTTAGAAATGCTTAAAATTTTTGGA

CTTTTAAGTCAATCACACCATAACGATGTGTTAAAGATTTTAGAAAAAATACTTGAAAAT

>KH0075

GTGAGTTTGATTAAAATTAACCATGATGAAAAAGTGATTGGGATTTTTATTCCTTTAACT

------------TCAATTTCAGGCAAAGTGCGTGTGAAAATCAGACATGCCTTTAGCGAT

TATGGTGTTTCAACAGCGACTAGAACAATCCCTTTTAGTTTAAAGCATTATGTAGAGTGG

CAGATCGGTTATGATGTCCCCATTAAAGATAAAGAA---AAATTTGAACTCACTACTTTA

AAAGATGAAAAATATCATTTTTTAGGGGCTAATAGTAAAACAAAAACTCTTTATGAATTG

AGCGAGATCATTTACTATGCCAAGCAATTAGGTTTAATCAGT---------TTAGAAAAT

TTAGAAAATACTTTAAAATATTTAGAAAAACAAAAACAATTTATAGAAGATAATTTTATG

ATTACAAGAGAAAGATTTAGATCGCATCAATTTGGTGGCATGGATTTTGAACTTTCACGC

ATTTCTTATCCTTTACTCATTCATTCTTTTAATGATAATCAATTGAGCGAAATTGTTATT

AGAGAGCAACAATATGGTTCTAAAACCCAAGCCATG---CTGTATTTTTGCTTTTCTATT

TTGGAGTTAAAAACCGCTACTCCCTTATTAAACAGAACGGCTACGCCCAAAGAACATGCT

CTTTTGATTATCCATAAAACCAACGCTCCCATGTTTTTAGAAATGCTTAAAATTTTTGGA

CTTTTAAGCCAAGCGCACCATGACGATGTGTTAAAGATTTTAGAAAAAATACTTCAAAAT

>HP15020

GTGAGTTTGATTAAAATTAACCATGATGAAAAAGTGATTGGGATTTTTATTCCTTTAACT

------------TCAATTTCAGGCAAAGTGCGTGTGAAAATCAGACATGCCTTTAGCGAT

TATGGTGTTTCAACAGCGACTAGAACAATCCCTTTTAGTTTAAAACATTATGTAGAGTGG

CAAATCGGTTATGATGTCCCCATTAAAGATAAAGAA---AAATTTGAACTCACTACTTTA

AAAGATGAAAAATATCATTTTTTAGGGGCTAATAATAAAGTAAAAACTCTTTATGAATTG

AGCGAGATTATTTACTATGCCAAGCAATTAGATTTAATCAGT---------TTGGAAAAT

TTAGAAAATACTTTAAAATATTTAGAAAAACAAAAACAATTTATAGAAGATAATTTTATG

ATTACAAGAGAAAGATTTAGATCACATCAATTTGGTGGCATGGATTTTGAACTTTCACGC

ATTTCTTATCCTTTACTCATTCATTCTTTTAATGATAATCAATTGAGCGAAATTGTTATT

AGAGAACAACAATACGGTTCTAAAACCCAAGCTATG---CTGTATTTTTGCTTTTCTATT

TTGGAATTAAAAACCGCTACTCCCTTATTAAACAGAACGGCTACGCTCAAAGAACATGCT

CTTTTGATTATCCATAAAACCAACGCTCCCATGTTTTTAAAAATGCTTAAAATTTTTGGA

CTTTTAAGCCAAGCGCACCATGATGATGTGTTAAAGATTTTAGAAAAAATACTTCAAAAT

>CHL23

GTGAGTTTGATTAAAATTAACCATGATGAAAAAGTGATTGGGATTTTTATTCCTTTAACT

------------TCAATTTCAGGCAAAGTGCGTGTGAAAATCAGACATGCCTTTAGCGAT

TATGGTGTTTCAACAGCGACTAGAACAATCCCTTTTAGTTTAAAACATTATGTAGAGTGG

CAGATCGGTTATGATGTCCCCATTAAAGATAAAGAA---AAATTTGAACTCACTACTTTA

AAAGATGAAAAATATCATTTTTTAGGGGCTAATAATAAAGTAAAAACTCTTTATGAATTG

AGCGAGATCATTTACTATGCCAAGCAATTAGATTTAATCAGT---------TTAGAAAAT

TTAGAAAATACTTTAAAATATTTAGAAAAACAAAAACAATTTATAGAAGATAATTTTATG

ATTACAAGAGAAAGATTTAGATCGCATCAATTTGGTGGCATGGATTTTGAACTTTCACGC

ATTTCCTATCCTTTACTCATTCATTCTTTTAATGATAATCAATTGAGCGAAATAGTTATT

AGAGAGCAACAATATGGTTCTAAAACCCAAGCTATG---CTGTATTTTTGCTTTTCTATT

TTGGAATTAAAAACCGCTACTCCCTTATTAAACAGAACGGCTACGCTTAAAGAACATGCT

CTTTTGATTATCCATAAAACCAACGCTCCCATGTTTTTAAAAATGCTTAAAATTTTTGGA

CTTTTAAGCCAAGCGCACCATGACGATGTGTTAAAGATTTTAGAAAAAATACTTCAAAAT

>HP15067

GTGAGTTTGATTGAAATTAACCATGATGAAAAAGTGATTGAGGTTTCTATTCCCTTAACT

------------TCAATTTCAGGCAAAGTGCGTGTGAAAATCAGACATGCCTTTAGTGAT

TATGGTGTTTCAACAGCGACTAGAACAATCCCTTTTAGTTTAAAACATTATGTAGAGTGG

CAAATCGGTTATGATGTCCCCATTAAAGATAAAGAA---AAATTTGAACTCGCTACTTTA

AAAGATAAAAAATATCATTTTTTAGGGGCTAATAATAAAGTAAAAACCCTTTATGAATTG

AGCGAGATCATTTACTATGCCAAGCAATTAGATTTAATCAGT---------TTAGAAAAT

TTAGAAAATACTTTAAAATATTTAGAAAAACAAAAACAATTTATAGAAGATAATTTTATG

ATTACAAGAGAAAGATTTAGATCGCATCAATTTGGTGGCATGGATTTTGAACTTTCACAC

ATTTCTTATCCTTTACTCATTCATTCTTTTAATGATAATCAATTGAGCGAAATTGTTATT

AGAGAACAACAATATGGTTCTAAAACCCAAGCTATG---CTGTATTTTTGCTTTTCTATT

TTGGAATTAAAAACCGCTACTCCCTTATTAAACAGAACGGCTACGCTCAAAGAACATGCT

CTTTTGATTATCCATAAAACCAACGCTCCCATGTTTTTAAAAATGCTTAAAATTTTTGGA

CTTTTAAGCCAAGCACACCATGACGATGTGTTAAAGATTTTAGAAAAAATACTTCAAAAT

>KH0042

GTGAGTTTGATTAAAATTAGTGGTGATAAAAAAGCGATTGAGGTTTCTATTCCCTTAACT

------------TCAATTTCAGGCAAAGTGCGTGTGAAAATCAGACATGCCTTTAGCGAT

TATGGTGTTTCAACAGCGACTAGAACAATCCCTTTTAGTTTAAAACATTATGTAGAGTGG

CAGATCGGTTATGATGTCCCCATTAAAGATAAAGAA---AAATTTGAACTCACTACTTTA

AAAGATGAAAAATATCATTTTTTAGGGGCTAATAGTAAAACAAAAACTCTTTATGAATTG

AGCGAGATCATTTACTATGCCAAGCAATTAGGTTTAATCAGT---------TTAGAAAAT

TTAGAAAATACTTTAAAATATTTAGAAAAACAAAAACAATTTATAGAAGATAATTTTATG

ATTACAAGAGAAAGATTTAGATCGCATCAATTTGGTGGCATGGATTTTGAACTTTCACAC

ATTTCTTATCCTTTACTCATTCATTCTTTTAATGATAATCAATTGAGCGAAATTGTTATT

AGAGAACAACAATATGGTTCTAAAACCCAAGCTATG---CTGTATTTTTGCTTTTCTATT

TTGGAATTAAAAACCGCTACTCCCTTATTAAACAGAACGGCTACGCCCAAAGAACATGCT

CTTTTGATTATCCATAAAACCAACGCTCCCATGTTTTTAAAAATGCTTAAAATTTTTGGA

CTTTTAAGCCAAGTGCACCATAACGATGTGTTAAAGATTTTAAGAAAAATACTTCAAAAT

>HP15002

GTGAGTTTGATTAAAATTAGTGGTGATAAAAAAGCGATTGAGGTTTCTATTCCCTTAACT

------------TCAATTTCAGGCAAAGTGCGTGTGAAAATCAGACATGCCTTTAGCGAT

TATGGTGTTTCAACAGCGACTAGAACAATCCCTTTTAGTTTAAAACATTATGTAGAGTGG

CAGATCGGTTATGATGTCCCCATTAAAGATAAAGAA---AAATTTGAACTCACTACTTTA

AAAGATGAAAAATATCATTTTTTAGGGGCTAATAGTAAAACAAAAACTCTTTATGAATTG

AGCGAGATCATTTACTATGCCAAGCAATTAGGTTTAATCAGT---------TTAGAAAAT

TTAGAAAATACTTTAAAATATTTAGAAAAACAAAAACAATTTATAGAAGATAATTTTATG

ATTACAAGAGAAAGATTTAGATCGCATCAATTTGGTGGCATGGATTTTGAACTTTCACAC

ATTTCTTATCCTTTACTCATTCATTCTTTTAATGATAATCAATTGAGCGAAATTGTTATT

AGAGAACAACAATATGGTTCTAAAACCCAAGCTATG---CTGTATTTTTGCTTTTCTATT

TTGGAATTAAAAACCGCTACTCCCTTATTAAACAGAACGGCTACGCCCAAAGAACATGCT

CTTTTGATTATCCATAAAACCAACGCTCCCATGTTTTTAAAAATGCTTAAAATTTTTGGA

CTTTTAAGCCAAGTGCACCATAACGATGTGTTAAAGATTTTAAGAAAAATACTTCAAAAT

>HP15018

GTGAGTTTGATTAGGATTGATGATAGTAAAAAAGCGATTGAGGTTTCCATTCCTTTAACT

------------TCAATTTCAGGCAAAGTGCGTGTGAAAATCAGACATGCCTTTAGCGAT

TATGGCATTTCAACAGCGACCAGAAAAATCCCTTTTAGCTTAAAACATTATGTAGAGTGG

CAAATCGGTTATGATGTCCCCATTAAAGATAAAGAA---AAATTGGAGCTCACTACTTTA

AAAGATGAAAAATATCATTTTTTAGGGGCTAATAATAAAATAAAAACCCTTTATGAATTA

AGCGAAATGATTTATTACGCTAAGCAATTAGATTTAATCAGT---------TTAGAAAAT

TTAGAAAATACTTTAAAATATTTAGAAAAACAAAAACAATTTATAGAAGATAATTTTATA

------AGAGAAAGATTTAGATCGCATCAATTTGGTGGCATGGATTTTGAACTCTCACGC

ATTTCTTATCCTTTGCTCATTCATTCTTTTAATGATAATCAATTGAGCGAAATCGTTATT

AGAGAGCAACAATATGGCTCTAAAACCCAAGCCATG---CTGTATTTTTGCTTTTCTGTT

TTGGAGTTAAAAACCGCTACCCCCTTATTAAACAGAACGGCTACGCCCAAAGAACATGCT

CTTTTGATTATCCATAAAACCAACGCTCCCATGTTTTTAGAAATGCTTAAAATTTTTGGA

CTTTTAAGCCAAGCACACCATGACGATGTGTTAAAGATTTTAGAAAAGGTACTTCAAAAT

>CHL51

GTGAGTTTGATTAAAATTAACCATGATGAAAAAGTGATTGGGATTTTTATTCCTTTAACT

------------TCAATTTCAGGCAAAGTGCGTGTGAAAATCAGACATGCCTTTAGCGAT

TATGGTATTTCAACAGCGACTAGAACAATCCCTTTTAGTTTAAAGCATTATGTAGAGTGG

CAGATCGGTTATGATGTCCCCATTAAAGATAAAGAA---AAATTTGAACTTACTACTTTA

AAAGATGAAAAATATCATTTTTTAGGAGCTAATAATAAAGTAAAAACGCTTTATGAATTG

AGCGAGATCATTTACTATGCCAAGCAATTAGGTTTAATCAGT---------TTAGAAAAT

TTAGAAAATACTTTAAAATATTTAGAAAAACAAAAACAATTTATAGAAGATAATTTTATG

ATTACAAGAGAAAGATTTAGATCACATCAATTTGGTGGCATGGATTTTGAACTTTCACAC

ATTTCTTATCCTTTACTCATTCATTCTTTTAATGATAATCAATTGAGCGAAATTGTTATT

AGAGAACAACAATATGGTTCTAAAACCCAAGCCATG---CTGTATTTTTGCTTTTCTATT

TTGGAATTAAAAACCGCTACTCCCTTATTAAACAGAACGGCTACGCCCAAAGAACATGCT

CTTTTGATTATCCATAAAACCAACGCTCCCATGTTTTTAGAAATGCTTAAAATTTTTGGA

CTTTTAAGCCAAGCGCACCATGACGATGTGTTAAAGATTTTAGAAAAAATACTTCAAAAT

>KH0210

ATGAGTTTGATTAAAATTAACCATGATGAAAAAGTGATTGGGATTTTTATTCCTTTAACT

------------TCAATTTCAGGCAAAGTGCGTGTGAAAATCAGACATGCCTTTAGCGAT

TATGGTGTTTCAACAGCGACTAGAACAATCCCTTTTAGTTTAAAGCATTATGTAGAGTGG

CAGATCGGTTATGATGTCCCCATTAAAGATAAAGAA---AAATTTGAACTCACTACTTTA

AAAGATAAAAAATATCATTTTTTAGGGGCTAATAATAAAGTAAAAACTCTTTATGAATTG

AGCGAAATGATTTATTACGCTAAGCAATTAGATTTAATCAGT---------TTAGAAAAT

TTAGAAAATACTTTAAAATATTTAGAAAAACAAAAACAATTTATAGAAGATAATTTTATG

ATTACAAGAGAAAGATTTAGATCGCATCAATTTGGTGGCATGGATTTTGAACTTTCACGC

ATTTCTTATCCTTTACTCATTCATTCTTTTAATGATAATCAATTGAGCGAAATTGTTATT

AGAGAGCAACAATATGGTTCTAAAACCCAAGCCATG---CTGTATTTTTGCTTTTCTATT

TTGGAGTTAAAAACCGCTACTCCCTTATTAAATAGAACGGCTACGCCCAAAGAACATGCT

CTTTTGATTATCCATAAAACCAACGCTCCCATGTTTTTAAAAATGCTTAAAATTTTTGGA

CTTTTAAGCCAAGTGCACCATAACGATGTGTTAAAGATTTTAGAAAAAATACTTCAAAAT

>CHL10

GTGAGTTTGATTAAAGTTAGTGGTGATAAAAAAGCGATTGAGATTTCTATTCCCTTAACT

------------TCAATTTCAGGTAAAGTGCGTGTGAAAATCAGACATGCCTTTAGCGAT

TATGGTGTTTCAACAGCGACTAGAACAATCCCTTTTAGTTTAAAACATTATGTGGAGTGG

CAAATCGGTTATGATGTCCCCATTAAAGATAAAGAA---AAATTTGAACTCACTACTTTA

AAAGATAAAAAATATCATTTTTTAGGGGCTAATAATAAAGTAAAAACTCTTTATGAATTG

AGCGAGATCATTTACTATGCCAAGCAATTAGATTTAATCAGT---------TTAGAAAAT

TTAGAAAATACTTTAAAATATTTAGAAAAACAAAAACAATTTATAGAGGATAATTTTATG

ATTACAAGAGAAAGATTTAGATCGCATCAATTTGGTGGCATGGATTTTGAACTTTCACGC

ATTTCTTATCCTTTACTCATTCATTCTTTTAATGATAATCAATTGAACGAAATTGTTATT

AGAGAGCAACAATATGGTTCTAAAACCCAAGCCATG---CTGTATTTTTGCTTTTCTATT

TTGGAGTTAAAAACCGCTACTCCCTTATTAAACAGAACGGCTACGCTCAAAGAACATGCT

CTTTTGATTATCTATAAAACCAACGCTCCCATGTTTTTAGAAATGCTTAAAATTTTTGGA

CTTTTAAGCCAAGCGCACCATGACGATGTGTTAAAGATTTTAGAAAAGACACTTCAAAAT

>22360

GTGAATTTGATTAAAGTTAATGATGATAAAAAAGCGATTGAGGTTTCTATTCCTTTAACT

------------TCCATTTCAGGCAAAGCGCATGTGAAAATCAGACATGCCTTTAGCGAT

TATGGTATTTCAACAGCGACTAGAAAAATCCCTTTCAGTTTAAAGCATTATGTAGAGTGG

CAAATCGGTTATGATGTCCCCATTAAAGATAAAGAA---AAATTTGAACTCACTACCCTA

AAAGATGAAAAATATCATTTTTTAGGGGCTAATAATAAAGTAAAAACCCTTTATGAATTG

AGTGAGATAATCTATTACGCTAAGCAATTAAATTTAATCAGT---------TTAGAAAAT

TTAGAAAATACTTTAAAATATTTAGAAAAACAAAAACAATTTATAGAAGATAATTTT---

---ATAAGAGAAAGATTTAGATTACATCAATTTGGTGGCATGGATTTTGAACTCTCACGC

ATCTCTTATCCTTTACTCATTCATTCTTTTAATGATAATCAGTTGAGCGAAATCGTTATT

AGAGAGCAACAATACGGCTCTAAAACCCAAGCCATG---CTGTATTTTTGCTTTTCTATT

TTGGAATTAAAAACCGCTACTCCCTTATTAAATAGAACGGCTGCACTCAAAGAACATGCC

CTTTTAACTATCCATAAAACCAACGCTCTTATGTTTTTAGAAATGCTTAAAATTTTTGGA

CTTTTAAGCCAAGCGCACCATAGCGATGTGTTAAAGATTTTAGAAAAAATACTTCAAAAT

>22019

GTGAATTTGATTAAAGTTAATGATGATAAAAAAGCGATTGAGGTTTCTATTCCTTTAACT

------------TCCATTTCAGGCAAAGCGCATGTGAAAATCAGACATGCCTTTAGCGAT

TATGGTATTTCAACAGCGACTAGAAAAATCCCTTTCAGTTTAAAGCATTATGTAGAGTGG

CAAATCGGTTATGATGTCCCCATTAAAGATAAAGAA---AAATTTGAACTCACTACCCTA

AAAGATGAAAAATATCATTTTTTAGGGGCTAATAATAAAGTAAAAACCCTTTATGAATTG

AGTGAGATAATCTATTACGCTAAGCAATTAAATTTAATCAGT---------TTAGAAAAT

TTAGAAAATACTTTAAAATATTTAGAAAAACAAAAACAATTTATAGAAGATAATTTT---

---ATAAGAGAAAGATTTAGATTACATCAATTTGGTGGCATGGATTTTGAACTCTCACGC

ATCTCTTATCCTTTACTCATTCATTCTTTTAATGATAATCAGTTGAGCGAAATCGTTATT

AGAGAGCAACAATACGGCTCTAAAACCCAAGCCATG---CTGTATTTTTGCTTTTCTATT

TTGGAATTAAAAACCGCTACTCCCTTATTAAATAGAACGGCTGCACTCAAAGAACATGCC

CTTTTAACTATCCATAAAACCAACGCTCTTATGTTTTTAGAAATGCTTAAAATTTTTGGA

CTTTTAAGCCAAGCGCACCATAGCGATGTGTTAAAGATTTTAGAAAAAATACTTCAAAAT

>HP15031

GTGAGTTTGATTAAAATTAACCATGATGAAAAAGTGATTGGGATTTTTATTCCTTTAACT

------------TCAATTTCAGGCAAAATGCGTGTGAAAATCAGACATGCCTTTAGCGAT

TATGGTATTTCAACAGCGACTAGAAAAATCCCTTTTAGTTTAAAGCATTATGTAGAGTGG

CAGATCGGTTATGATGTCCCCATTAAAGATAAAGAA---AAATTTGAACTCACTACTTTA

AAAGATGAAAAATATCATTTTTTAGGGGCTAATGGTAAAACAAAAACTCTTTATGAATTG

AGCGAGATCATTTACTATGCCAAGCAATTAGGTTTAATCAGT---------TTAGAAAAT

TTAGAAAATACTTTAAAATATTTAGAAAAACAAAAACAATTTATAGAAGATAATTTTATG

ATTACAAGAGAAAGATTTAGATCGCATCAATTTGGTGGCATGGATTTTGAACTCTCACGC

ATTTCTTATCCTTTACTCATTCATTCTTTTAATGATAATCAATTGAGCGAAATTGTTATT

AGAGAGCAACAATATGGTTCTAAAACCCAAGCCATG---CTGTATTTTTGCTTTTCTATT

TTGGAGTTAAAAACCGCTACTCCCTTATTAAACAGAACGGCTACGCTCAAAGAACATGCT

CTTTTGATTATCCATAAAACCAACGCTCCCATGTTTTTAAAAATGCTTAAAATTTTTGGA

CTTTTAAGCCAAACGCACCATGACGATGTGTTAAAGATTTTAGAAAAGATACTTCAAAAT

>22046

GTGAGTTTGATTAAGATTGATGATGATAAAAAAGCGATTGAGGTTTCTATTCCTTTAACT

------------TCCATTTCAGGCAAAGCGCGTGTGAAAATCAGACATGCCTTTAGCGAT

TATGGCATTTCAACAGCGACTAGAAAAATCCCTTTCAGTTTAAAACATTATGTAGAGTGG

CAAATCGGTTATGATGTCCCCATTAAAGATAAAGAA---AAATTTGAACTCACTACCCTA

A-AGATGAAAAATATCATTTTTTAGGGGCTAATAATAAAGTAAAAACCCTTTATGAATTG

AGTGAGATAATCTATTACGCTAAGCAATTAAATTTAATCAGT---------TTAGAAAAT

TTAGAAAATACTTTAAAATATTTAGAAAAACAAAAACAATTTATAGAAGATAATTTT---

---ATAAGAGAAAGATTTAGATTACATCAATTTGGTGACATGGATTTTGAACTTTCACGC

ATTTCTTATCCTTTACTCATTCATTCTTTTAATGATAATCAATTGAGTGAAATCGTTATT

AGAGAGCAACAATATGGCTCTAAAACCCAAGCCATG---CTGTATTTTTGCTTTTCTATT

TTGGAGTTAAAAACCGCTACTCCCTTATTAAATAGAACGGCTGCACTCAAAGAACATGCC

CTTTTAACTATCCATAAAACCAACGCTCTTGTGTTTTTAGAAATGCTTAAAATTTTTGGA

CTTTTAAGCCAAGCGCACCATAGCGATGTGTTAAAGATTTTAGAAAAAATACTTCAAAAT

>MHP15

GTGAGTTTGATTAAAGTTAATGATGATAAAAAAGTGATTGAGGTTTCTATTCCTTTAACT

------------TCCATTTCAGGCAAAGCGCGTGTGAAAATCAGACATGCCTTTAGCGAT

TATGGCATTTCAACAGCGACTAGAAAAATCCCTTTCAGTTTAAAGCATTATGTAGAGTGG

CAAATCGGTTATGATGTCCCCATTAAAGATAAAGAA---AAATTTGAACTCACTACCCTA

AAAGATGAAAAATATCATTTTTTAGGGGCTAATAATAAAAGAAAAACCCTTTATGAATTG

AGCGAAATGATTGATTACGCTAAGCGATTGGGTTTAATCAGT---------TTAGAAAAT

TTAGAAAATACTTTAAAATATTTAGAAAAACAAAAACAATTTATAGAAGATAATTTTATA

------AGAGAAAGATTTAGATCGCATCAATTTGGTGGCATGGATTTTGAACTTTCACGC

ATTTCTTATCCTTTACTCATTCATTTTTTCAATGATAATCAGTTGAGCGAAATCGTTATT

AGAGAGCAACAATACGGCTCTAAAACCCAAGCCATG---CTATATTTTTGCTTTTCTATT

CTGGAATTAAAAACCGCTACCCCCTTATTAAATAGAACGGCTGCCCTCAAAGAACATGCC

CTTTTAACTATCCATAAAGCCAACGCTCTTGTGTTTTTAGAAATGCTTAAAATTTTTGGA

CTTTTAAGCCAAGCGCACCATAACGATGTGTTAAAGATTTTAGAAAAAATACTTCAAAAT

>MHP13

GTGAGTTTGATTAAAGTTAATGATGATAAAAAAGTGATTGAGGTTTCTATTCCTTTAACT

------------TCCATTTCAGGCAAAGCGCGTGTGAAAATCAGACATGCCTTTAGCGAT

TATGGCATTTCAACAGCGACTAGAAAAATCCCTTTCAGTTTAAAGCATTATGTAGAGTGG

CAAATCGGTTATGATGTCCCCATTAAAGATAAAGAA---AAATTTGAACTCACTACCCTA

AAAGATGAAAAATATCATTTTTTAGGGGCTAATAATAAAAGAAAAACCCTTTATGAATTG

AGCGAAATGATTGATTACGCTAAGCGATTGGGTTTAATCAGT---------TTAGAAAAT

TTAGAAAATACTTTAAAATATTTAGAAAAACAAAAACAATTTATAGAAGATAATTTTATA

------AGAGAAAGATTTAGATCGCATCAATTTGGTGGCATGGATTTTGAACTTTCACGC

ATTTCTTATCCTTTACTCATTCATTTTTTCAATGATAATCAGTTGAGCGAAATCGTTATT

AGAGAGCAACAATACGGCTCTAAAACCCAAGCCATG---CTATATTTTTGCTTTTCTATT

CTGGAATTAAAAACCGCTACCCCCTTATTAAATAGAACGGCTGCCCTCAAAGAACATGCC

CTTTTAACTATCCATAAAGCCAACGCTCTTGTGTTTTTAGAAATGCTTAAAATTTTTGGA

CTTTTAAGCCAAGCGCACCATAACGATGTGTTAAAGATTTTAGAAAAAATACTTCAAAAT

>MHP12

GTGAGTTTGATTAAAGTTAATGATGATAAAAAAGTGATTGAGGTTTCTATTCCTTTAACT

------------TCCATTTCAGGCAAAGCGCGTGTGAAAATCAGACATGCCTTTAGCGAT

TATGGCATTTCAACAGCGACTAGAAAAATCCCTTTCAGTTTAAAGCATTATGTAGAGTGG

CAAATCGGTTATGATGTCCCCATTAAAGATAAAGAA---AAATTTGAACTCACTACCCTA

AAAGATGAAAAATATCATTTTTTAGGGGCTAATAATAAAAGAAAAACCCTTTATGAATTG

AGCGAAATGATTGATTACGCTAAGCGATTGGGTTTAATCAGT---------TTAGAAAAT

TTAGAAAATACTTTAAAATATTTAGAAAAACAAAAACAATTTATAGAAGATAATTTTATA

------AGAGAAAGATTTAGATCGCATCAATTTGGTGGCATGGATTTTGAACTTTCACGC

ATTTCTTATCCTTTACTCATTCATTTTTTCAATGATAATCAGTTGAGCGAAATCGTTATT

AGAGAGCAACAATACGGCTCTAAAACCCAAGCCATG---CTATATTTTTGCTTTTCTATT

CTGGAATTAAAAACCGCTACCCCCTTATTAAATAGAACGGCTGCCCTCAAAGAACATGCC

CTTTTAACTATCCATAAAGCCAACGCTCTTGTGTTTTTAGAAATGCTTAAAATTTTTGGA

CTTTTAAGCCAAGCGCACCATAACGATGTGTTAAAGATTTTAGAAAAAATACTTCAAAAT

>S380A

GTGAGTTTGATTAAAATTAACCATGATGAAAAAGTGATTGAGGTTTCTATTCCCTTAACT

------------TCAATTTCAGACAAAGTGCGTGTGAAAATCAGACATGCCTTTAGCGAT

TATGGTGTTTCAACAGCGACTAGAAAAATCCCTTTTAGTTTAAAACATTATGTAGAGTGG

CAAATCGGTTATGATGTCCCCATTAAAGATAAAGAA---AAATTTGAACTCACTACTTTA

AAAGATGAAAAATATCATTTTTTAGGGGCTAATAATAAAGTAAAAACTCTTTATGAATTG

AGCGAGATCATTTACTATGCCAAGCAATTAGATTTAATCAGT---------TTAGAAAAT

TTAGAAAATACTTTAAAATATTTAGAAAAACAAAAACAATTTATAGAAGATAATTTTATG

ATTACAAGAGAAAGATTTAGATCGCATCAATTTGGTGGCATGGATTTTGAACTTTCACGC

ATTTCTTATCCTTTACTCATTCATTCTTTTAATGATAATCAATTGAGCGAAATTGTTATT

AGAGAGCAACAATATGGTTCTAAAACCCAAGCCATG---TTGTATTTTTGCTTTTCTATT

TTGGAGTTAAAAACCGCTCCTCCCTTATTAAACAGAACGGCTATGTGCAAAGAACATGCT

CCTTTGATTATCCATAAAGCCAACGCTCCCATGTTTTTAGAAATGCTTAAAATTTTTGGA

CTTTTAAGCCAAACGCACCATAACGATGTGTTAAAGATTTTAGAAAAAATACTTCAAAAT

>KH0120

GTGAGTTTGATTAAAATTAACCATGATGAAAAAGTGATTGGGATTTTTATTCCTTTAACT

------------TCAATTTCAGGCAAAGTGCGTGTGAAAATCAGACATGCCTTTAGCGAT

TATGGTATTTCAACAGCGACTAGAACAATCCCTTTTAGTTTAAAACATTATGTAGAGTGG

CAGATCGGTTATGATGTCCCCATTAAAGATAAAGAA---AAATTTGAACTCACTACTTTA

AAAGATGAAAAATATTATTTTTTAGGGGCTAATAATAAAGTAAAAACTCTTTATGAATTG

AGCGAAATGATTTATTACGCCAAGCAATTAGGTTTAATCAGT---------TTAGAAAAT

TTAGAAAATACTTTAAAATATTTAGAAAAACAAAAACAATTTATAGAAGATAATTTTATG

ATTACAAGAGAAAGATTTAGATCGCATCAATTTGGTGGCATGGATTTTGAACTTTCACGC

ATTTCTTATCCTTTACTCATTCATTCTTTTAATGATAATCAATTGAGCGAAATTGTTATT

AGAGAGCAACAATATGGTTCTAAAACCCAAGCCATG---CTGTATTTTTGCTTTTCTATT

TTGGAGTTAAAAACCGCTACTCCCTTATTAAACAGAACGGCTACGCCCAAAGAACATGCT

CTTTTGATTATCCATAAAACCAACGCTCCCATGTTTTTAGAAATGCTTAAAATTTTTGGA

CTTTTAAGCCAAGCACACCATGACGATGTGTTAAAGATTTTAAAAAAAATACTTCAAGAT

>241

GTGAGTTTGATTAAAATTAACCGTGATGAAAAAGTGATTGGGATTTTTATTCCTTTAACT

------------TCAATTTCAGGCAAAGTGCGTGTGAAAATCAGACATGCCTTTAGTGAT

TATGGTGTTTCAACAGCGACTAGAAAAATCCCTTTTAGTTTAAAACATTATGTAGAGTGG

CAGATCGGTTATGATGTCCCCATTAAAGATAAAGAA---AAATTTGAACTCACTACTTTA

AAAGATAAAAAATATCATTTTTTAGGGGCTAATAATAAAGTAAAAACTCTTTATGAATTG

AGCGAAATGATTTATTACGCTAAGCAATTAGGTTTAATCAGT---------TTAGAAAAT

TTAGAAAATACTTTAAAATATTTAGAAAAACAAAAACAATTTATAGAAGATAATTTTATG

ATTACAAGAGAAAGATTTAGATCGCATCAATTTGGTGGCATGGCTTTTGAACTTTCACGC

ATTTCTTATCCTTTACTCATTCATTCTTTTAATGATAATCAATTGAACGAAATTGTTATT

AGAGAGCAACAATATGGTTCTAAAACCCAAGCCATG---CTGTATTTTTGCTTTTCTATT

TTGGAGTTAAAAACCGCTACTCCCTTATTAAACAGAACGGCTACGCCCAAAGAACATGCT

CTTTTGATTATCCATAAAACCAACGCTCCCATGTTTTTAGAAATGCTTAAAATTTTTGGA

CTTTTAAGCCAAACGCACCATAACGATGCGTTAAAGATTTTAGAAAAGATACTTCAAAAT

>Hpbs1

GTGAGTTTGATTAAAATTAACCATGATGAAAAAGTGATTGAGGTTTTTATTCCTTTAACT

------------TCAATTTCAGGCAAAGTGCGTGTGAAAATCAGACATGCCTTTAGCGAT

TATGGTGTTTCAACAGCGACTAGAACAATCCCTTTTAGTTTAAAACATTATGTAGAGTGG

CAAATCGGTTATGATGTCCCCATTAAAGATAAAGAA---AAATTTGAACTCACTACTTTA

AAAGATAAAAAATATCATTTTTTAGGGGCTAATAATAAAGTAAAAACTCTTTATGAATTG

AGCGAGATCATTTACTATGCCAAGCAATTAGGTTTAATCAGT---------TTAGAAAAT

TTAGAAAATACTTTAAAATATTTAGAAAAACAAAAACAATTTATAGAAGATAATTTTATG

ATTACAAGAGAAAGATTTAGATCGCATCAATTTGGTGGCATGGCTTTTGAACTTTCACAC

ATTTCTTATCCTTTACTCATTCATTCTTTTAATGATAATCAATTGAGCGAAATTGTTATT

AGAGAACAACAATATGGTTCTAAAACCCAAGCCATG---CTGTATTTTTGCTTTTCTATT

TTGGAGTTAAAAACCGCTACTCCCTTATTAAACAGAACGGCTACGCTCAAAGAACATGCT

CCTTTGATTATCCATAAAACCAACGCTCCCATGTTTTTAGAAATGCTTAAAATTTTTGGA

CTTTTAAGCCAAGCGCACCATGACGATGTGTTAAAGATTTTAGAAAAAATACTTCAAAAT

>F20

GTGAGTTTGATTAAAATTAACTATGATGAAAAAGTGATTGGGATTTTTATTCCTTTAACT

------------TCAATTTCAGGCAAAGTGCGTGTGAAAATCAGACATGCCTTTAGCGAT

TATGGTATTTCAACAGCGACTAGATCAATCCCTTTTAGTTTAAAACATTATGTAGAGTGG

CAAATCGGTTATGATGTCCCCATTAAAGATAAAGAA---AAATTTGAACTCACTACTTTA

AAAGATAAAAAATATCATTTTTTAGGGGCTAATAATAAAGTAAAAACTCTTTATGAATTG

AGCGAGATCATTTACTATGCCAAGCAGTTAGGTTTAATCAGT---------TTAGAAAAT

TTAGAAAATACTTTAAAATATTTAGAAAAACAAAAACAATTTATAGAAGATAATTTTATG

ATTACAAGAGAAAGATTTAGATCGCATCAATTTGGTGGCATGGATTTTGAACTTTCACAC

ATTTCTTATCCTTTACTCATTCATTCTTTTAATGATAATCAATTGAGCGAAATTGTTATT

AGAGAGCAACAATATGGTTCTAAAACCCAAGCCATG---CTGTATTTTTGCTTTTCTATT

TTGGAATTAAAAACCGCTACTCCCTTATTAAACAGAACGGCTACGCTTAAAGAACATGCT

CTTTTGATTATCCATAAAACCAACGCTCCCATGTTTTTAGAAATGCTTAAAATTTTTGGA

CTTTTAAGCCAAACGCACCATGACGATGTGTTAAAGATTTTAGAAAAGATACTTCAAAAT

>ZH27

GTGAGTTTGATTAAAGTTAGTGGTGATAAAAAAGTGATTGAGGTTTCTATTCCTTTAACT

------------TCAATTTCAGGCAAAGTGCGTGTGAAAATCAGACATGCCTTTAGCGAT

TATGGGATTTCAACAGCGACTAGAAAAATCCCTTTTAGTTTAAAACATTATATAGAGTGG

CAGATCGGTTATGATGTCCCCATTAAAGATAAAGAA---AAATTTGAACTCACTACTTTA

AAAGATGAAAAATATCATTTTTTAGGGGTTAATGATAAAGTAAAAACTCTTTATGAATTG

AGTGAAATGATTGATTACGCTAAGCAATTAGACTTAATCAGT---------TTAGAAAAT

TTAGAAAATACTTTAAAATATTTAGAAAAACAAAAACAATTTATAGAAGATAATTTTATA

ATCACAAGAGAAAGATTCAGATCGCATCAATTTGGTGGCATGGATTTTGAACTTTCACGC

ATTTCTTATCCCTTACTCATTCATTCTTTTAATGATAATCAATTGAGCGAAATAGTTATT

AGAGAGCAACAATATGGTTCTAAAACCCAAGCCATG---CTGTATTTTTGCTTTTCTATT

TTGGAATTAAAAACCGCTACCCCCTTATTAAATAGAACCGCTATGCTCAAAGAGCATGCT

CTTTTGATTATCCATAAAACCAACGCTCTCATGTTTTTAGAAATGCTTAAAATTTTTGGA

CTTTTAAGCCAAGCGCACCATAACGATGTGTTAAAGATTTTT------------------

>HP15028

GTGAGTTTGATTAAAGTTAATGGTGATAAAAAAGTGATTGAGATTTCTATTCCTTTAACT

------------TCAATTTCAGGCAAAGTGCGTGTGAAAATCAGACATGCCTTTAGCGAT

TATGGTATTTCAACAGCGACTAGAACAATCCCTTTTAGTTTAAAACATTATGTAGAGTGG

CAAATCGGTTATGATGTCCCCATTAAAGATAAAGAA---AAATTTGAACTCACTACTTTA

AAAGATGAAAAATATCATTTTTTAGGGGCTAATAATAAAGTAAAGACTCTTTATGAATTA

AGCGAAATGATTGATTACGCTAAGCAATTAGGTTTAATTGGT---------TTAGACAAT

TTAGAAAATACTTTAAAATATTTAGAAAAACAAAAACAATTTATAGAAGATAATTTT---

---ACAAGAGAAAGATTTAGATCGCATCAATTTGGTGGCATGGATTTTGAACTCTCACGC

ATTTCTTATCCCTTACTCATTCATTCTTTTAATGATAATCAGTTGAGCGAAATTGTTATT

AGGGAACAACAATATGGCTCTAAAACCCAAGCCATG---CTGTATTTTTGCTTTTCTATT

TTGGAGTTAAAAACCGCTACCCCCTTATTAAACAGAACGGCTATGCTCAAAGAGCATGCT

CTTTTGATTATCCATAAAACCAACGCTCTCATGGTTTTAGAAATGCTTAAAACTTTTGGA

CTTTTAAGCCAAGCGCACCATAACGATGTGTTAAAGATTTTAGAAAAAATACTTCAAAAT

>MHP28

GTGAGTTTGATTAAAGTTAATGATGATAAAAAAGCGATTGAGGTTTCTATTCCTTTAACT

------------TCCATTTCAGGCAAAACGCATGTGAAAATCAGACACGCCTTTAGCGAT

TATGGTATTTCAACAGCGACTAGAAAAATCCCTTTCAGTTTAAAGCATTATGTAGAGTGG

CAAATCGGTTATGATGTCCCCATTAAAGATAAAGAA---AAATTTGAGCTCACTACCCTA

AAAGATGAAAAATATCATTTTTTAGGGGCTAATAATAGAGTAAAGACTCTTTATGAATTG

AGCGAAATAATTGATTACGCTAAGCGATTGGGTTTAATCAGT---------TTAGAAAAT

TTAGAAAATACTTTAAAATATTTAGAAAAACAAAAACAATTCATAGAAGATAGTTTTATG

ATCACAAGAGAAAGATTTAGATCGCATCAATTTGGTGGCATGGATTTTGAACTTTCACGC

ATTTCTTATCCTCTACTCATTCATTCTTTCAACGATAATCAATTAAGTGAAATCGTTATT

AGAGAGCAACAATACGGCTCTAAAACCCAAGCCATG---CTATATTTTTGCTTTTCTATT

CTGGAATTAAAAACCGCTACCCCCTTATTAAATAGAACCGCTGCCCTCAAAGAACGCACT

CTTTTAACTATCCATAAAACCAACGCTCCCATGTTTTTAGAAACGCTTAAAATTTTTGGA

CTTTTAAGCCAAGCGCACCATAACGATGTGTTAAAGATTTTAGAAAAAATACTTGAAAAT

>HP11055

GTGAGTTTGATTAAAATTAACCATGATGAAAAAGTGATTGGGATTTTTATTCCTTTAACT

------------TCAATTTCAGGCAAAGTGCGTGTGAAAATCAGACATGCCTTTAGCGAT

TATGGTGTTTCAACAGCGACTAGAACAATCCCTTTTAGTTTAAAGCATTATGTAGAGTGG

CAGATCGGTTATGATGTCCCCATTAAAGATAAAGAA---AAATTTGAACTCACTACTTTA

AAAGATAAAAAATATCATTTTTTAGGGGCTAATAATAAAGTAAAAACTCTTTATGAATTG

AGCGAAATGATTTATTACGCTAAGCAATTAGATTTAATCAGT---------TTAGAAAAT

TTAGAAAATACTTTAAAATATTTAGAAAAACAAAAACAATTTATAGAAGATAATTTTATG

ATTACAAGAGAAAGATTTAGATCGCATCAATTTGGTGGCATGGATTTTGAACTTTCACGC

ATTTCTTATCCTTTACTCATTCATTCTTTTAATGATAATCAATTGAGCGAAATTGTTATT

AGAGAGCAACAATATGGTTCTAAAACCCAAGCCATG---CTGTATTTTTGCTTTTCTATT

TTGGAGTTAAAAACCGCTACTCCCTTATTAAATAGAACGGCTACGCCCAAAGAACATGCT

CTTTTGATTATCCATAAAACCAACGCTCCCATGTTTTTAAAAATGCTTAAAATTTTTGGA

CTTTTAAGCCAAGTGCACCATAACGATGTGTTAAAGATTTTAGAAAAAATACTTCAAAAT

>KH0054

GTGAGTTTGATTAAAATTAACCATGATGAAAAAGTGATTGGGATTTTTATTCCTTTAACT

------------TCAATTTCAGGCAAAGTGCGTGTGAAAATCAGACATGCCTTTAGCGAT

TATGGTGTTTCAACAGCGACTAGAACAATCCCTTTTAGTTTAAAGCATTATGTAGAGTGG

CAGATCGGTTATGATGTCCCCATTAAAGATAAAGAA---AAATTTGAACTCACTACTTTA

AAAGATAAAAAATATCATTTTTTAGGGGCTAATAATAAAGTAAAAACTCTTTATGAATTG

AGCGAAATGATTTATTACGCTAAGCAATTAGATTTAATCAGT---------TTAGAAAAT

TTAGAAAATACTTTAAAATATTTAGAAAAACAAAAACAATTTATAGAAGATAATTTTATG

ATTACAAGAGAAAGATTTAGATCGCATCAATTTGGTGGCATGGATTTTGAACTTTCACGC

ATTTCTTATCCTTTACTCATTCATTCTTTTAATGATAATCAATTGAGCGAAATTGTTATT

AGAGAGCAACAATATGGTTCTAAAACCCAAGCCATG---CTGTATTTTTGCTTTTCTATT

TTGGAGTTAAAAACCGCTACTCCCTTATTAAATAGAACGGCTACGCCCAAAGAACATGCT

CTTTTGATTATCCATAAAACCAACGCTCCCATGTTTTTAAAAATGCTTAAAATTTTTGGA

CTTTTAAGCCAAGTGCACCATAACGATGTGTTAAAGATTTTAGAAAAAATACTTCAAAAT

>ZH98

GTGAGTTTGATTAAAATTAACCATGATGAAAAAGTGATTGGGATTTTTATTCCTTTAACT

------------TCAATTTCAGGCAAAGTGCGTGTGAAAATCAGACATGCCTTTAGCGAT

TATGGTGTTTTAACAGCGACTAGAAAAATCCCTTTTAGTTTAAAGCATTATGTAGAGTGG

CAAATCGGTTATGATGTCCCCATTAAAGATAAAGAA---AAATTTGAACTCACTACTTTA

AAAGATGAAAAATATCATTTTTTAGGGGCTAATAATAAAGTAAAAACTCTTTATGAATTG

AGCGAGATCATTTATTATGCCAAACAATTAGGTTTAATCAGT---------TTAGAAAAT

TTAGAAAATACTTTAAAATATTTAGAAAAACAAAAACAATTTATAGAAGATAATTTTATG

ATTACAAGAGAAAGATTTAGATCGCATCAATTTGGTGGTATGGATTTTGAACTTTCACAC

ATTTCTTATCCTTTACTCATTCATTCTTTTAATGATAATCAATTGAGTGAAATTGTTATT

AGAGAACAACAATATGGTTCTAAAACCCAAGCCATG---CTGTATTTTTGCTTTTCTATT

TTGGAGTTAAAAACCGCTACTCCCTTATTAAACAGAACGGCTACGCTTAAAGAACATGCT

CTTTTGATTATCCATAAAGCCAACGCTCCCATGTTTTTAGAAATGCTTAAAATTTTTGGA

CTTTTAAGCCAAGTGCACCATGACGATGTGTTAAAGATTTTAGAAAAGATACTTCAAAAT

>MHP54

GTAAGTTTGATTAAAGTTAATGATGATAAAAAAGCGATTGAGGTTTCTATTCCTTTAACT

------------TCCACTTCAGGCAAAGCGCGTGTGAAAATCAGACATGCCTTTAGCGAT

TATGGTATTTCAACAGCGACTAGAAAAATCCCTTTCAGTTTAAAGCATTATGTAGAGTGG

CAAATCGGTTATGATGTCCCCATTAAAGATAAAGAA---AAATTTGAACTCACTACCCTA

AAAGATGAAAAATATCATTTTTTAGGGGCTAATAATAAAGTAAAGACTCTTTATGAATTG

AGCGAAATAATTGATTACGCTAAGCGATTGGGTTTAATCAGT---------TTAGAAAAT

TTAGAAAATACTTTAAAATATTTAGAAAAACAAAAACAATTTATAGAAGATAATTTTATA

------AGAGAAAGATTTAGATCGCATCAATTTGGTGGCATGGATTTTGAACTTTCACGC

ATTTCTTATCCTTTACTCATTCATTCTTTTAATGATAATCAGTTGAGCGAAATCGTTATT

AGAGAGCAACAATACGGCTCTAAAACCCAAGCCATG---CTGTATTTTTGCTTTTCTATT

CTGGAATTAAAAACCGCTACCCCCTTATTAAATAGGACCGCTGCCCTCAAAGAACATGCC

CTTTTAACTATCCATAAAACCAACGCTCCCATGTTTTTAGAAATGCTTAAAATTTTTGGA

CTTTTAAGTCAATCACACCATAACGATGTGTTAAAGATTTTAGAAAAAATACTTGAAAAT

>F75

GTGAGTTTGATTAAAATTAACCATGATGAAAAAGTGATTGGGATTTTTATTCCTTTAACT

------------TCAATTTCAGGCAAAGTGCGTGTGAAAATCAGACATGCCTTTAGTGAT

TATGGTATTTCAACAGCGACTAGAACAATCCCTTTTAGTTTAAAACATTATGCAGAGTGG

CAAATCGGTTATGATGTCCCCATTAAAGATAAAGAA---AAATTTGAACTCACTACTTTA

AAAGATAAAAAATATCATTTTTTAGGGGCTAATAATAAAGTAAAAACTCTTTATGAATTG

AGCGAGATCATTTACTATGCCAAGCAGTTAGGTTTAATCAGT---------TTAGAAAAT

TTAGAAAATACTTTAAAATATTTAGAAAAACAAAAACAATTTATAGAAGATAATTTTATG

ATTACAAGAGAAAGATTTAGATCGCATCAATTTGGTGGCATGGATTTTGAACTTTCACAC

ATTTCTTATCCTTTACTCATTCATTCTTTTAATGATAATCAATTGAGCGAAATTGTTATT

AGAGAACAACAATATGGTTCTAAAACCCAAGCTATG---CTGTATTTTTGCTTTTCTATT

TTGGAATTAAAAACCGCTACTCCCTTATTAAACAGAACGGCTACGCTCAAAGAACATGCT

CTTTTGATTATCCATAAAACCAACGCTCCCATGTTTTTAGAAATGCTTAAAATTTTTGGA

CTTTTAAGCCAAACGCACCATGACGATGTGTTAAAGATTTTAGAAAAGATACTTCAAAAT

>K25

GTGAGTTTGATTAAAATTAACCATGATGAAAAAGTGATTGGGATTTTTATTCCTTTAACT

------------TCAATTTCAGGCAAAGTGCGTGTGAAAATCAGACATGCCTTTAGCGAT

TATGGTGTTTCAACAGCGACTAGAACAATCCCTTTTAGTTTAAAACATTATGTAGAGTGG

CAAATCGGTTATGATGTCCCCATTAAAGATAAAGAA---AAATTTGAACTCACTACTTTA

AAAGATAAAAAATATCATTTTTTAGGGGCTAATAATAAAGTAAAAACTCTTTATGAATTG

AGCGAGATTATTTACTATGCCAAGCAATTAGATTTAATCAGT---------TTAGAAAAT

TTAGAAAATACTTTAAAATATTTAGAAAAACAAAAACAATTTATAGAAGATAATTTTATG

ATTACAAGAGAAAGATTTAGATCGCATCAATTTGGTGGTATGGATTTTGAACTTTCACAC

ATTTCTTATCCTTTACTCATTCATTCTTTTAATGATAATCAATTGAGCGAAATTGTTATT

AGAGAGCAACAATATGGTTCTAAAACCCAAGCTATG---CTGTATTTTTGCTTTTCTATT

TTGGAATTAAAAACCGCTACTCCCTTATTAAACAGAACGGCTACGCTCAAAGAACATGCT

CTTTTGATTATCCATAAAACCAACGCTCCCATGTTTTTAGAAATGCTTAAAATTTTTGGA

CTTTTAAGCCAAGCGCACCATGACGATGTGTTAAAGATTTTAGAAAAAATACTTCAAAAT

>A

GTGAGTTTGATTAAAATTAACCATGATGAAAAAGTGATTAAGGTTTCTATTCCTTTAACT

------------TCAATTTCAGGCAAAGTGCGTGTGAAAATCAGACATGCCTTTAGTGAT

TATGGTATTTCAACAGCGACTAGAAAAATCTCTTTTAGTTTAAAGCATTATGTAGAGTGG

CAGATCGGTTATGATGTCCCCATTAAAGATAAAGAA---AAATTTGAACTCACTACTTTA

AAAGATGAAAAATATCATTTTTCAGGGGCTAATGGTAAAACAAAAACTCTTTATGAATTG

AGCGAGATCATTTATTATGCCAAACAATTAGGTTTAATCAGT---------TTAAAAAAT

TTAGAAAATACTTTAAAATATTTAGAAAAACAAAAACAATTTATAGAAGATAATTTTATG

ATTACAAGAGAAAGATTTAGATTACATCAATTTGGTGGCATGGATTTTGAACTCTCACGC

ATTTCTTATCCTTTACTCATTCATTCTTTTAATGATAATCAGTTGAGCGAAATAGTTATT

AGAGAACAACAATATGGTTCTAAAACCCAAGCCATG---CTGTATTTTTGCTTTTCCATT

TTGGAATTAAAAACCGCTACTCCCTTATTAAACAGAACGGCTGCACTCAAAGAACATGCC

CTTTTAACTATCCATAAAACCAACGCTCCCATGTTTTTAAAAATGCTTAAAATTTTTGGA

ATTTTAAGCCAAACGCACCATGACGATGTGTTAGAGATTTTAGAAAAGATACTTCAAAAT

>1177

GTGAGTTTGATTAAAATTAACCATGATGAAAAAGTGATTGGGATTTTTATTCCTTTAACT

------------TCAATTTCAGGCAAAGTGCGTGTGAAAATCAGACATGCCTTTAGCGAT

TATGGTGTTTCAACAGCGACTAGAAAAATCCCTTTTAGTTTAAAACATTATGTGGAGTGG

CAGATCGGTTATGATGTCCCCATTAAAGATAAAGAA---AAATTTGAACTCACTACTTTA

AAAGATGAAAAATATCATTTTTTAGGGGCTAATAATAAAATAAAAACTCTTTATGAATTG

AGCGAGATCATTTACTATGCCAAGCAATTAGGTTTAATCAGT---------TTAGAAAAT

TTAGAAAATACTTTAAAATATTTAGAAAAACAAAAACAATTTATAGAAGATAATTTTATG

ATTACAAGAGAAAGATTTAGATCCCATCAATTTGGTGGCATGGATTTTGAACTTTCACAC

ATTTCTTATCCTTTACTCATTCATTCTTTTAATGATAATCAATTGAGCGAAATTGTTATT

AGAGAGCAACAATATGGTTCTAAAACCCAAGCTATG---CTGTATTTTTGCTTTTCTATT

TTGGAATTAAAAACCGCTACTCCCTTATTAAACAGAACGGCTACGCTTAAAGAACATGCT

CCTTTGATTATCCATAAAACCAACGCTCCCATGTTTTTAGAAATGCTTAAAATTTTTGGA

CTTTTAAGCCAAGCGCACCATGACGATGTGTTAAAGATTTTAAAAAAAATACTTCAAGAT

>CC26084

GTGAATTTGATTAAAGTTAATGATGATAAAAAAGCGATTGAGGTTTCTATTCCTTTAACT

------------TCCATTTCAGGCAAAGCGCATGTGAAAATCAGACATGCCTTTAGCGAT

TATGGTATTTCAACAGCGACTAGAAAAATCCCTTTCAGTTTAAAGCATTATGTAGAGTGG

CAAATCGGTTATGATGTCCCCATTAAAGATAAAGAA---AAATTGGAGCTCACTACCCTA

AAAGATGAAAAATATCATTTTTTAGGGGCTAATAATAAAGTAAAAACCCTTTATGAATTG

AGTGAGATAATCTATTACGCTAAGCAATTAAATTTAATCAGT---------TTAGAAAAT

TTAGAAAATACTTTAAAATATTTAGAAAAACAAAAACAATTTATAGAAGATAATTTT---

---ATAAGAGAAAGATTTAGATTACATCAATTTGGTGGCATGGATTTTGAACTCTCACGC

ATCTCTTATCCTTTACTCATTCATTCTTTTAATGATAATCAGTTGAGCGAAATCGTTATT

AGAGAGCAACAATACGGCTCTAAAACCCAAGCCATG---CTGTATTTTTGCTTTTCTATT

TTGGAATTAAAAACCGCTACTCCCTTATTAAACAGAACGGCTGCACTCAAAGAACATGCC

CTTTTAACTATCCATAAAACCAACGCTCTTATGTTTTTAGAAATGCTTAAAATTTTTGGA

CTTTTAAGCCAAGCGCACCATAGCGATGTGTTAAAGATTTTAGAAAAAATACTTCAAAAT

>26084

GTGAATTTGATTAAAGTTAATGATGATAAAAAAGCGATTGAGGTTTCTATTCCTTTAACT

------------TCCATTTCAGGCAAAGCGCATGTGAAAATCAGACATGCCTTTAGCGAT

TATGGTATTTCAACAGCGACTAGAAAAATCCCTTTCAGTTTAAAGCATTATGTAGAGTGG

CAAATCGGTTATGATGTCCCCATTAAAGATAAAGAA---AAATTGGAGCTCACTACCCTA

AAAGATGAAAAATATCATTTTTTAGGGGCTAATAATAAAGTAAAAACCCTTTATGAATTG

AGTGAGATAATCTATTACGCTAAGCAATTAAATTTAATCAGT---------TTAGAAAAT

TTAGAAAATACTTTAAAATATTTAGAAAAACAAAAACAATTTATAGAAGATAATTTT---

---ATAAGAGAAAGATTTAGATTACATCAATTTGGTGGCATGGATTTTGAACTCTCACGC

ATCTCTTATCCTTTACTCATTCATTCTTTTAATGATAATCAGTTGAGCGAAATCGTTATT

AGAGAGCAACAATACGGCTCTAAAACCCAAGCCATG---CTGTATTTTTGCTTTTCTATT

TTGGAATTAAAAACCGCTACTCCCTTATTAAACAGAACGGCTGCACTCAAAGAACATGCC

CTTTTAACTATCCATAAAACCAACGCTCTTATGTTTTTAGAAATGCTTAAAATTTTTGGA

CTTTTAAGCCAAGCGCACCATAGCGATGTGTTAAAGATTTTAGAAAAAATACTTCAAAAT

>HP13031

GTGAGTTTGATTAAAATTAACCATGATGAAAAAGTGATTGGGATTTTTATTCCTTTAACT

------------TCAATTTCAGGCAAAGTGCGTGTGAAAATCAGACATGCCTTTAGCGAT

TATGGTGTTTCAACAGCGACTAGAACAATCCCTTTTAGTTTAAAGCATTATGTAGAGTGG

CAGATCGGTTATGATGTCCCCATTAAAGATAAAGAA---AAATTTGAACTCACTACTTTA

AAAGATAAAAAATATCATTTTTTAGGGGCTAATAATAAAGTAAAAACTCTTTATGAATTG

AGCGAAATGATTTATTACGCTAAGCAATTAGATTTAATCAGT---------TTAGAAAAT

TTAGAAAATACTTTAAAATATTTAGAAAAACAAAAACAATTTATAGAAGATAATTTTATG

ATTACAAGAGAAAGATTTAGATCGCATCAATTTGGTGGCATGGATTTTGAACTTTCACGC

ATTTCTTATCCTTTACTCATTCATTCTTTTAATGATAATCAATTGAGCGAAATTGTTATT

AGAGAGCAACAATATGGTTCTAAAACCCAAGCCATG---CTGTATTTTTGCTTTTCTATT

TTGGAGTTAAAAACCGCTACTCCCTTATTAAATAGAACGGCTACGCCCAAAGAACATGCT

CTTTTGATTATCCATAAAACCAACGCTCCCATGTTTTTAAAAATGCTTAAAATTTTTGGA

CTTTTAAGCCAAGCGCACTATAACGATGTGTTAAAGATTTTAGAAAAAATACTTCAAAAT

>KH0022

GTGAGTTTGATTAAAATTAACCATGATGAAAAAGTGATTGGGATTTTTATTCCTTTAACT

------------TCAATTTCAGGCAAAGTGCGTGTGAAAATCAGACATGCCTTTAGCGAT

TATGGTGTTTCAACAGCGACTAGAACAATCCCTTTTAGTTTAAAGCATTATGTAGAGTGG

CAAATCGGTTATGATGTCCCCATTAAAGATAAAGAA---AAATTTGAACTCACTACTTTA

AAAGATGAAAAATATCATTTTTTAGGGGCTAATAGTAAAACAAAAACTCTTTATGAATTG

AGCGAGATCATTTACTATGCCAAGCAATTAGATTTAATCAGT---------TTAGAAAAT

TTAGAAAATACTTTAAAATATTTAGAAAAACAAAAACAATTTATAGAAGATAATTTTATG

ATTACAAGAGAAAGATTTAGATCGCATCAATTTGGTGGCATGGATTTTGAACTTTCACGC

ATTTCTTATCCTTTACTCATTCATTCTTTTAATGATAATCAATTGAGCGAAATTGTTATT

AGAGAACAACAATATGGTTCTAAAACTCAAGCTATG---CTGTATTTTTGCTTTTCTATT

TTGGAATTAAAAACCGCTACTCCCTTATTAAACAGAACGGCTACGCCCAAAGAACATGCT

CTTTTGATTATCCATAAAACCAACGCTCCCATGTTTTTAGAAATGCTTAAAATTTTTGGA

CTTTTAAGCCAAGCGCACCATGACGATGTGTTAAAGATTTTAGAAAAAATACTTCAAAAT

>K21

GTGAGTTTGATTAAAATTAACCATGATGAAAAAGTGATTGAGGTTTCTATTCCTTTAACT

------------TCAATTTCAGGCAAAGTGCGTGTGAAAATCAGACATGCCTTTAGTGAT

TATGGTATTTCAACAGCGACTAGAACAATCTCTTTTAGTTTAAAACATTATGTAGAGTGG

CAGATCGGTTATGATGTCCCCATTAAAGATAAAGAA---AAATTTGAACTCACTACTTTA

AAAGATAAAAAATATCATTTTTTAGGGGCTAATAATAAAGTAAAAACTCTTTATGAATTA

AGCGAGATCATTTATTATGCCAAACAATTAGGTTTAATCAGT---------TTAGAAAAT

TTAGAAAATACTTTAAAATATTTAGAAAAACAAAAACAATTTATAGAAGATAATTTTATG

ATTACAAGAGAAAGATTTAGATCGCATCAATTTGGTGGCATGGATTTTGAACTCTCACGC

ATTTCTTACCCTTTACTCATTCATTCTTTTAATGATAATCAATTGAGCGAAATTGTTATT

AGAGAACAACAACATGGTTCTAAAACCCAAGCCATG---CTGTATTTTTGCTTTTCTACT

TTGGAATTAAAAACCGCTACTCCCTTATTAAACAGAACGGCTACGCCCAAAGAACATGCT

CTTTTGATTATCCATCAAACCAACGCTCCCATATTTTTAGAAATGCTTAAAATTTTTGGA

CTTTTAAGCCAAGCGCACCATAACGATGTGTTAAAGATTTTAGAAAAAATACTTCAAGAT

>NCTC13207

GTGAGTTTGATTAAAGTTAATGATGATAAAAAAGTGATTGAGGTTTCTATTCCTTTAACT

------------TCCATTTCAGGCAAAGTTCGTGTGAAAATCAGGCATGCCTTTAGCGAT

TATGGTATTTCAACAGCGACTAGAAAAATCCCTTTTAGTTTAAAGCATTATGTAGAGTGG

CAAATCGGTTATGATGTCCCCATTAAAGATAAAGAA---AAATTTGAACTCACTACTTTA

AAAGATGAAAAATATCATTTTTTAGGGGCTAATAATAAAGTAAAAACCCTTTATGAATTG

AGCGAAATAATTGATTACGCTAAGCGATTGGGTTTAATCAGT---------TTAGAAAAT

TTAGAAAATACTTTAAAATATTTAGAAAAACAAAAACAATTTATAGAAGATAATTTTATA

------AGAGAAAGATTTAGATTACATCAATTTGGTGGCATGGATTTTGAACTTTCACGC

ATTTCTTATCCTTTACTCATTCATTCTTTTAATGATAATCAATTGAGTGAAATCGTTATT

AGAGAGCAACAATACGACTCTAAAACCCAAGCCATG---CTGTATTTTTGCTTTTCTATT

TTGGAATTAAAAACCGCTACTCCCTTATTAAACAGAACGGCTGCACTCAAAGAACATGCC

CTTTTAACTATCCATAAAACCAACACTCTTGTGTTTTTAGAAATGCTTAAAATTTTTGGA

CTTTTAAGCCAAGCACACCATAACGATGTGTTAAAGATTTTAGAAAAAACACTTCAAAAT

>CHL42

GTGAGTTTGATTAAAATTAACCATGATGAAAAAGTGATTGGGATTTTTATTCCTTTAACT

------------TCAATTTCAGGCAAAGTGCGTGTGAAAATCAGACATGCCTTTAGCGAT

TATGGTGTTTCAACAGCGACTAGAACAATCCCTTTTAGTTTAAAGCATTATGTAGAGTGG

CAAATCGGTTATGATGTCCCCATTAAAGATAAAGAA---AAATTTGAACTCACTACTTTA

AAAGATAAAAAATATCATTTTTTAGGGGCTAATAATAAAGTAAAAACTCTTTATGAATTG

AGCGAGATCATTTATTATGCCAAGCAATTAGATTTAATCAGT---------TTAGAAAAT

TTAGAAAATACTTTAAAATATTTAGAAAAACAAAAACAATTTATAGAAGATAATTTTATG

ATTGCAAGAGAAAGATTTAGATCGCATCAATTTGGTGGCATGGATTTTGAACTCTCACGC

ATTTCTTATCCTTTACTCATTCATTCTTTTAATGATAATCAATTGAGCGAAATTGTTATT

AGAGAACAACAATATGGTTCTAAAACCCAAGCTATG---CTGTATTTTTGCTTTTCTATT

TTGGAATTAAAAACCGCTACTCCCTTATTAAACAGAACGGCTACGCTCAAAGAACATGCT

CTTTTGATTATCCATAAAGCCAACGCTCCCATGTTTTTAGAAATGCTTAAAATTTTTGGA

CTTTTAAGCCAAGCGCACCATGACGATGTGTTAAAGATTTTAGAAAAAATACTTCAAAAT

>MKF10

GTGAGTTTGATTGAAATTAACCATGATGAAAAAGTGATTGGGATTTTTATTCCTTTAACT

------------TCAATTTCAGGCAAAGTGCGTGTGAAAATCAGACATGCCTTTAGCGAT

TATGGTGTTTCAACAGCGACTAGAACAATCCCTTTTAGTTTAAAACATTATGTAGAGTGG

CAGATCGGTTATGATGTCCCCATTAAAGATAAAGAA---AAATTTGAACTCACTACTTTA

AAAGATGAAAAATATCATTTTTTAGGGGCTAATAATAAAGTAAAAACTCTTTATGAATTG

AGCGAGATCATTTACTATGCCAAGCAGTTAGGTTTAATCAGT---------TTAGAAAAT

TTAGAAAATACTTTAAAATATTTAGAAAAACAAAAACAATTTATAGAAGATAATTTTATG

ATTACAAGAGAAAGATTTAGATCGCATCAATTTGGTGGCATGGATTTTGAACTTTCACAC

ATTTCTTATCCTTTACTCATTCATTCTTTTAATGATAATCAATTGAGCGAAATAGTTATT

AGAGAACAACAACATGGTTCTAAAACCCAAGCTATG---CTGTATTTTTGCTTTTCTATT

TTGGAATTAAGAACCGCTACTCCCTTATTAAACAGAACGGCTACGCTCAAAGAACATGCC

CTTTTGATTATCCATAAAACTAACGCTCCCATGTTTTTAAAAATGCTTAAAATTTTTGGA

CTTTTAAGCCAAGCACACCATGACGATGTGTTAAAGATTTTAGAAAAAATACTTCAAAAT

>F210

GTGAGTTTGATTGAAATTAACCATGATGAAAAAGTGATTGGGATTTTTATTCCTTTAACT

------------TCAATTTCAGGCAAAGTGCGTGTGAAAATCAGACATGCCTTTAGCGAT

TATGGTGTTTCAACAGCGACTAGAACAATCCCTTTTAGTTTAAAACATTATGTAGAGTGG

CAGATCGGTTATGATGTCCCCATTAAAGATAAAGAA---AAATTTGAACTCACTACTTTA

AAAGATGAAAAATATCATTTTTTAGGGGCTAATAATAAAGTAAAAACTCTTTATGAATTG

AGCGAGATCATTTACTATGCCAAGCAGTTAGGTTTAATCAGT---------TTAGAAAAT

TTAGAAAATACTTTAAAATATTTAGAAAAACAAAAACAATTTATAGAAGATAATTTTATG

ATTACAAGAGAAAGATTTAGATCGCATCAATTTGGTGGCATGGATTTTGAACTTTCACAC

ATTTCTTATCCTTTACTCATTCATTCTTTTAATGATAATCAATTGAGCGAAATAGTTATT

AGAGAACAACAACATGGTTCTAAAACCCAAGCTATG---CTGTATTTTTGCTTTTCTATT

TTGGAATTAAGAACCGCTACTCCCTTATTAAACAGAACGGCTACGCTCAAAGAACATGCC

CTTTTGATTATCCATAAAACTAACGCTCCCATGTTTTTAAAAATGCTTAAAATTTTTGGA

CTTTTAAGCCAAGCACACCATGACGATGTGTTAAAGATTTTAGAAAAAATACTTCAAAAT

>CHL26

GTGAGTTTGATTAAAATTAACCATGATGAAAAAGTGATTGGGATTTTTATTCCTTTAACT

------------TCAATTTCAGGCAAAGTGCGTGTGAAAATCAGACATGCCTTTAGCGAT

TATGGTGTTTCAACAGCGACTAGAACAATCCCTTTTAGTTTAAAGCATTATGTAGAGTGG

CAGATCGGTTATGATGTCCCCATTAAAGATAAAGAA---AAATTTGAACTCACTACTTTA

AAAGATAAAAAATATCATTTTTTAGGGGCTAATAATAAAATAAAAACTCTTTATGAATTG

AGCGAGATCATTTACTATGCCAAGCAATTAGATTTAATCAGT---------TTAGAAAAT

TTAGAAAATACTTTAAAATATTTAGAAAAACAAAAACAATTTATAGAAGATAATTTTATG

ATTACAAGAGAAAGATTTAGATCGCATCAATTTGGTGGCATGGCTTTTGAACTTTCACGC

ATTTCTTATCCTTTACTCATTCATTCTTTTAATGATAATCAATTGAGCGAAATTGTTATT

AGAGAACAACAATATGGTTCTAAAACCCAAGCCATG---CTGTATTTTTGCTTTTCTATT

TTGGAGTTAAAAACCGCTACTCCCTTATTAAACAGAACGGCTACGCTCAAAGAACATGCT

CTTTTGATTATCCATAAAACCAACGCTCCCATGTTTTTAGAAATGCTTAAAATTTTTGGA

CTTTTAAGCCAAGCGCACCATGACGATGTGTTAAAGATTTTAGAAAAAATACTTCAAAAT

>UM246

GTGAGTTTGATTAAAATTAACCATGATGAAAAAGTGATTGGGATTTTTATTCCTTTAACT

------------TCAATTTCAGGCAAAGTGCGTGTGAAAATCAGACATGCCTTTAGCGAT

TATGGTATTTCAACAGCGACTAGAACAATCCCTTTTAGTTTAAAGCATTATGTAGAGTGG

CAGATCGGTTATGATGTCCCCATTAAAGATAAAGAA---AAATTTGAACTCACTACTTTA

AAAGATAAAAAATATCATTTTTTAGGGGCTAATAATAAAGTAAAAACTCTTTATGAATTA

AGCGAAATAATTTATTACGCTAAGCAATTAGGTTTAATCAGT---------TTAGAAAAT

TTAGAAAATACTTTAAAATATTTAGAAAAACAAAAACAATTTATAGAAGATAATTTTATG

ATTACAAGAGAAAGATTTAGATCGCATCAATTTGGTGGCATGGATTTTGAACTCTCACGC

ATTTCTTATCCTTTACTCATTCATTCTTTTAATGATAATCAATTGAGCGAAATTGTTATT

AGAGAGCAACAATATGGTTCTAAAACCCAAGCCATG---CTGTATTTTTGCTTTTCTATT

TTGGAGTTAAAAACCGCTCCTCCCTTATTAAACAGAACGGCTACGCCCAAAGAACATGCT

CTTTTGATTATCCATAAAACCAACGCTCCCATGTTTTTAGAAATGCTTAAAATTTTTGGA

CTTTTAAGCCAAACGCACCATGACGATGTGTTAAAGATTTTAGAAAAAATACTTCAAAAT

>CHL21

GTGAGTTTGATTAAAATTAACCATGATGAAAAAGTGATTGGGATTTTTATTCCTTTAACT

------------TCAATTTCAGGCAAAGTGCGTGTGAAAATCAGACATGCCTTTAGCGAT

TATGGTGTTTCAACAGCGACTAGAACAATCCCTTTTAGTTTAAAACATTATGTAGAGTGG

CAAATCGGTTATGATGTCCCCATTAAAGATAAAGAA---AAATTTGAACTCACTACTTTA

AAAGATAAAAAATATCATTTTTTAGGGGCTAATAATAAAGTAAAAACTCTTTATGAATTG

AGCGAGATCATTTACTATGCCAAGCAATTAGGTTTAATCAGT---------TTAGAAAAT

TTAGAAAATACTTTAAAATATTTAGAAAAACAAAAACAATTTATAGAAGATAATTTTATG

ATTACAAGAGAAAGATTTAGATCGCATCAATTTGGTGGCATGGATTTTGAACTTTCACGC

ATTTCTTATCCTTTACTCATTCATTCTTTTAATGATAATCAATTGAGCGAAATTGTTATT

AGAGAACAACAATATGGTTCTAAAACCCAAGCTATG---CTGTATTTTTGCTTTTCTATT

TTGGAGTTAAAAACCGCTCCTCCCTTATTAAACAGAACGGCTACGCGCAAAGAACATGCT

CTTTTGATTATCCATAAAACCAACGCTCCCATGTTTTTAGAAATGCTTAAAATTTTTGGA

CTTTTAAGCCAAGTGCACCATGACGATGTGTTAAAGATTTTAGAAAAGATACTTCAAAAT

>KH0175

GTGAGTTTGATTAAAATTAACCATGATGAAAAAGTGATTGGGATTTTTATTCCTTTAACT

------------TCAATTTCAGGCAAAGTGCGTGTGAAAATCAGACATGCCTTTAGCGAT

TATGGTGTTTCAACAGCGACTAGAAAAATCCCTTTTAGTTTAAAACATTATGTAGAGTGG

CAGATCGGTTATGATGTCCCCATTAAAGATAAAGAA---AAATTTGAACTCACTACTTTA

AAAGATAAAAAATATCATTTTTTAGGGGCTAATAATAAAGTAAAAACTCTTTATGAATTG

AGCGAGATTATTTACTATGCCAAGCAATTAGATTTAATCAGT---------TTAGAAAAT

TTAGAAAATACTTTAAAATATTTAGAAAAACAAAAACAATTTATAGAAGATAATTTTATG

ATTACAAGAGAAAGATTTAGATCACATCAATTTGGTGGCATGGATTTTGAACTTTCACGC

ATTTCTTATCCTTTACTCATTCATTCTTTTAATGATAATCAATTGAGCGAAATTGTTATT

AGAGAGCAACAATATGGTTCTAAAACCCAAGCCATG---CTGTATTTTTGCTTTTCTATT

TTGGAGTTAAAAACCGCTACTCCCTTATTAAATAGAACGGCTACGCCCAAAGAACATGCT

CTTTTGATTATCCATAAAACCAACGCTCCCATGTTTTTAAAAATGCTTAAAATTTTTGGA

CTTTTAAGCCAAGTGCACCATGACGATGTGTTAAAGATTTTAGAAAAAATACTTCAAAAT

>F17

GTGAGTTTGATTAAAATTAACCATGATGAAAAAGTGATTGGGATTTTTATTCCTTTAACT

------------TCAATTCCAGGCAAAGTGCGTGTGAAAATCAGACATGCCTTTAGCGAT

TATGGTGTTTCAACAGCGACTAGAACAATCCCTTTTAGTTTAAAACATTATGTAGAGTGG

CAAATCGGTTATGATGTCCCCATTAAAGATAAAGAA---AAATTTGAACTCACTACTTTA

AAAGATAAGAAATATCATTTTTTAGGGGCTAATAATAAAGTAAAAACTCTTTATGAATTG

AGCGAGATCATTTACTATGCCAAGCAATTAGGTTTAATCAGT---------TTAGAAAAT

TTAGAAAATACTTTAAAATATTTAGAAAAACAAAAACAATTTATAGAAGATAATTTTATG

ATTACAAGAGAAAGATTTAGATCGCATCAATTTGGTGGCATGGCTTTTGAACTTTCACGC

ATTTCTTATCCTTTACTCATTCATTCTTTTAATGATAATCAATTGAGCGAAATTGTTATT

AGAGAACAACAATATGGTTCTAAAACCCAAGCTATG---CTGTATTTTTGCTTTTCTATT

TTGGAATTAAAAACCGCTACTCCCTTATTAAACAGAACGGCTACGCCCAAAGAACATGCC

CTTTTGATTATCCATAAAACCAACGCTCCCATGTTTTTAGAAATGCTTAAAATTTTTGGA

CTTTTAAGCCAAGCACACCATGACGATGTGTTAAAGATTTTAGAAAAGATACTTCAAAAT

>B455

GTGAGTTTGATTAGGATTGATGATAGTAAAAAAGCGATTGAGGTTTCTATTCCTTTAACT

------------TCAATTTCAGACAAAGCGCGTGTGAAAATCAGGCATGCCTTTAGCGAT

TATGGCATTTCAACAGCGACCAGAAAAATCCCTTTTAGTTTAAAACATTATGTAGAGTGG

CAAATCGGTTATGATGTCCCCATTAAAGATAAAGAA---AAATTTGAGCTCACTACCCTA

AAAGATGAAAAATATCATTTTTTAGGGGCTAATAATAAAGTAAAAACCCTTTATGAATTG

AGTGAGATAATCTATTACGCTAAGCAATTAAATTTAATCAGT---------TTAGAAAAT

TTAGAAAATACTTTAAAATATTTAGAAAAACAAAAACAATTTATAGAAGATAATTTTATA

------AGAGAAAGATTTAGATTACATCAATTTGGTGGCATGGATTTTGAACTTTCACGC

ATTTCTTATCCTTTGCTCATTCATTCTTTTAATGATAATCAGTTGAGCGAAATCGTTATT

AGAGAACAACAATATGGCTCTAAAACCCAAGCCATG---CTGTATTTTTGCTTTTCTATT

TTGGAATTAAAAACCGCTATCCCCTTATTAAATAGAACGGCTGCACTCAAAGAACATACT

CTTTTAACCATCAATAAAACCAACGCTCTTGTGTTTTTAGAAATGCTTAAAATTTTTGGA

CTTTTAAGCCAAGCGCACCATAGCGATGTGTTAAAGATTTTAGAAAAAATACTTCAAAAT

>MHP10

GTGAGTTTGATTAAAATTAACCATGATGAAAAAGTGATTGGGATTTTTATTCCTTTAACT

------------TCAATTTCAGGCAAAGTGCGTGTGAAAATCAGACATGCCTTTAGCGAT

TATGGTGTTTCAACAGCGACTAGAAAAATCCCTTTTAGTTTAAAACATTATGTAGAGTGG

CAAATCGGTTATGATGTCCCCATTAAAGATAAAGAA---AAATTTGAACTCACTACTTTA

AAAGATAAAAAATATCATTTTTTAGGGGCTAATAATAAAGTAAAAACTCTTTATGAATTG

AGCGAGATCATTTACTATGCCAAGCAATTAGGTTTAATCAGT---------TTAGAAAAT

TTAGAAAATACTTTAAAATATTTAGAAAAACAAAAACAATTTATAGAAGATAATTTTATG

ATTACAAGAGAAAGATTTAGATCGCATCAATTTGGTGGCATGGCTTTTGAACTTTCACGC

ATTTCTTATCCTTTACTCATTCATTCTTTTAATGATAATCAATTGAGCGAAATTGTTATT

AGAGAGCAACAATATGGTTCTAAAACCCAAGCTATG---CTGTATTTTTGCTTTTCTATT

TTGGAGTTAAAAACCGCTCCTCCCTTATTAAACAGAACGGCTACGCCCAAAGAACATGCT

CTTTTGATTATCCATAAAACCAACGCTCCCATGTTTTTAGAAATGCTTAAAATTTTTGGA

CTTTTAAGCCAAACGCACCATGACGATGTGTTAAAGATTTTAGAAAAAATACTTCAAAAT

>JMM43

GTGAGTTTGATTAGGATTGATGATAGTAAAAAAGCGATTGAGGTTTCTATTCCTTTAACT

------------TCAATTTCAGGCAAAGCGCGTGTGAAAATCAGACATGCCTTTAGCGAT

TATGGTATTTCAACAGCGACCAGAAAAATCCCTTTCAGTTTAAAACATTATGTAGAGTGG

CAAATCGGTTATGATGTCCCCATTAAAGATAAAGAA---AAATTTGAGCTCACTACCCTA

AAAGATGAAAAATATCATTTTTTAGGGGCTAATAATAAAGTAAAAACCCTTTATGAATTG

AGTGAGATAATCTATTACGCTAAGCAATTAAATTTAATCAGT---------TTAGAAAAT

TTAGAAAATACTTTAAAATATTTAGAAAAACAAAAACAATTTATAGAAGATAATTTCACG

ATTACAAGAGAAAGATTTAGATCGCATCAATTTGGTGGCGTGGATTTTGAACTTTCACGC

ATCTCTTATCCTTTACTCATTCATTCTTTTAATGATAATCAGTTGAGTGAAATCGTTATT

AGAGAGCAACAATACGGCTCTAAAACCCAAGCCATG---CTGTATTTTTGCTTTTCTATT

TTGGAATTAAAAACCGCTACTCCCTTATTAAATAGAACGGCTGCACTCAAAGAACATGCC

CTTTTAACTATCCATAAAACCAACGCTCTTGTGTTTTTAGAAATGCTTAAAATTTTTGGA

CTTTTAAGCCAAGCGCACCATAACGAGTGT------------------------------

>ZH68

GTGAGTTTGATTAAAGTTAATGATGATAAAAAAGTGATTGAGGTTTCTATTCCTTTAACT

------------TCCACTTCAGGCAAAGCGCGTGTGAAAATCAGACATGCCTTTAGCGAT

TATGGCATTTCAACAGCGACTAGAAAAATCCCTTTTAGTTTAAAGCATTATGTAGAGTGG

CAAATCGGTTATGATGTCCCCATTAAAGATAAAGAA---AAATTTGAACTCACTACTTTA

AAAGATGAAAAATATCATTTTTTAGGGGCTAATAATCAAGTAAAAACCCTTTATGAATTG

AGCGAAATAATTGATTACGCTAAGCGATTGGGTTTAATCAGT------------------

TTAGAAAATACTTTAAAATATTTAGAAAAACAAAAACAATTCATAGAAGATAATTTTATG

ATCACAAGAGAAAGATTTAGATCGCATCAATTTGGGGGCATGGATTTTGAACTTTCACGC

ATTTCTTATCCTTTACTCATTCATTTTTTCAATGATAATCAGTTGAGCGAAATCGTTATT

AGAGAGCAACAATACGGCTCTAAAACCCAAGCCATG---CTGTATTTTTGCTTTTCTATT

CTGGAATTAAAAACCGCTACACCCTTATTAAATAGAACGGCTGCACTCAAAGAACACGCC

CTTTTAACTATCCATAAAACCAACGCTCCCATGTTTTTAGAAATGCTTAAAATTTTTGGC

CTTTTAAGCCAAGCGCACCACAACGATGTGTTAAAGATTTTAGAAAAAATACTTGAAAAT

>G272

GTGAGTTTGATTAAAATTAACCATGATGAAAAAGTGATTGGGATTTTTATTCCTTTAACT

------------TCAATTTCAGGCAAAGTGCGTGTGAAAATCAGACATGCCTTTAGCGAT

TATGGTATTTCAACAGCGACTAGAACAATCCCTTTTAGTTTAAAACATTATGTAGAGTGG

CAAATCGGTTATGATGTCCCCATTAAAGATAAAGAA---AAATTTGAACTCACTACTTTA

AAAGATAAAAAATATCATTTTTTAGGGGCTAATAATAGAGTAAAAACTCTTTATGAATTG

AGCGAGATCATTTACTATGCCAAGCAATTAGGTTTAATCAGT---------TTAGAAAAT

TTAGAAAATACTTTAAAATATTTAGAAAAACAAAAACAATTTATAGAAGATAATTTTATG

ATTACAAGAGAAAGATTTAGATCACATCAATTTGGTGGCATGGATTTTGAACTTTCACAC

ATTTCTTATCCTTTACTCATTCATTCTTTTAATGATAATCAATTGAGCGAAATAGTTATT

AGAGAGCAACAATATGGTTCTAAAACCCAAGCCATG---CTGTATTTTTGCTTTTCTATT

TTGGAATTAAAAACCGCTACTCCCTTATTAAACAGAACGGCTACGCCCAAAGAACATGCC

CTTTTGATTATCCATAAAGCCAACGCTCCCATGTTCTTAGAAATGCTTAAAATTTTTGGA

CTTTTAAGCCAAGCACACCATGACGATGTGTTAAAGATTTTAGAAAAAATACTTCAAAAT

>S468A

GTGAGTTTGATTAAAATTAACCATGATGAAAAAGTGATTGGGATTTTTATTCCTTTAACT

------------TCAATTTCAGGCAAAGTGCGTGTGAAAATCAGACATGCCTTTAGCGAT

TATGGTGTTTCAACGGCGACTAGAACAATCCCTTTTAGTTTAGAGCATTATGTAGAGTGG

CAGATCGGTTATGATGTCCCCATTAAAGATAAAGAA---AAATTTGAACTCACTACTTTA

AAAGATGAAAAATATCATTTTTTAGGGGCTAATAATAAAGTAAAAACTCTTTATGAATTG

AGCGAGATCATTCATTATGCCAAACAATTAGGTTTAATCAGT---------TTAGAAAAT

TTAGAAAATACTTTAAAATATTTAGAAAAACAAAAACAATTTATAGAAGATAATTTTATG

ATTACAAGAGAAAGATTTAAATCGCATCAATTTGGTGGCATGGATTTTGAACTTTCACAC

ATTTCCTATCCTTTACTCATTCATTCTTTTAATGATAATCAATTGAGCGAAATAGTTATT

AGAGAGCAACAATATGGTTCTAAAACCCAAGCTATG---CTGTATTTTTGCTTTTCTATT

TTGGAATTAAAAACCGCTACTCCTTTATTAAACAGAACGGCTACGCTCAAAGAACATGCT

CTTTTGATTATCCATAAAACCAACGCTCCCATGTTTTTAGAAATGCTTAAAATTTTTGGA

CTTTTAAGCCAAGCGCACCATGACGATGTGTTAAAGATTTTAGAAAAGACACTTCAAAAT

>MKM5

GTGAGTTTGATTAAAATTAACCATGATGAAAAAGTGATTGGGATTTTTATTCCTTTAACT

------------TCAATTTCAAGTAAAGTGCGTGTGAAAATCAGACATGCCTTTAGTGAT

TATGGTATTTCAACAGCGACTAGAACAATCCCTTTTAGTTTAAAACATTATGCAGAGTGG

CAAATCGGTTATGATGTCCCCATTAAAGATAAAGAA---AAATTTGAACTCACTACTTTA

AAAGATAAAAAATATCATTTTTTAGGGGCTAATAATAAAGTAAAAACTCTTTATGAATTG

AGCGAGATCATTTATTATGCCAAACAATTAGGTTTAATCAGT---------TTAGAAAAT

TTAGAAAATACTTTAAAATATTTAGAAAAACAAAAACAATTTATAGAAGATAATTTTATG

ATTACAAGAGAAAGATTTAGATCGCATCAATTTGGTGGCATGGATTTTGAACTTTCACAC

ATTTCTTATCCTTTACTCATTCATTCTTTTAATGATAATCAATTGAGCGAAATTGTTATT

AGAGAACAACAATATGGTTCTAAAACCCAAGCTATG---CTGTATTTTTGCTTTTCTATT

TTGGAATTAAAAACCGCTACTCCCTTATTAAACAGAACGGCTACGCTCAAAGAACATGCT

CTTTTGATTATCCATAAAACCAACGCTCCCATGTTTTTAGAAATGCTTAAAATTTTTGGA

CTTTTAAGCCAAACGCACCATGACGATGTGTTAAAGATTTTAGAAAAGATACTTCAAAAT

>KH0097

GTGAGTTTGATTGAAATTAACCATGATGAAAAAGTGATTGGGATTTTTATTCCTTTAACT

------------TCAATTTCAGGCAAAATGCGTGTGAAAATCAGACATGCCTTTAGCGAT

TATGGTGTTTCAACAGCGACTAGAACAATCCCTTTTAGTTTAAAGCATTATGTAGAGTGG

CAAATCGGTTATGATGTCCCCATTAAAGATAAAGAA---AAATTTGAACTCACTACTTTA

AAAGATAAAAAATATCATTTTTTAGGGGCTAATAATAAAGTAAAAACTCTTTATGAATTG

AGCGAGATCATTTACTATGCCAAGCAATTAGATTTAATCAGT---------TTAGAAAAT

TTAGAAAATACTTTAAAATATTTAGAAAAACAAAAACAATTTATAGAAGATAATTTTATG

ATTACAAGAGAAAGATTTAGATCACATCAATTTGGTGGCATGGATTTTGAACTTTCACGC

ATTTCTTATCCTTTACTCATTCATTCTTTTAATGATAATCAATTGAGCGAAATTGTTATT
[truncated: 50,756 more chars]
