# Supplementary material for: Helicobacter pylori base-excision restriction enzyme in stomach carcinogenesis
Source: PNAS Nexus. 2025 Aug 5;4(8):pgaf244. doi: 10.1093/pnasnexus/pgaf244 (PMC12366791; doi:10.1093/pnasnexus/pgaf244)
Supplement: pgaf244_Supplementary_Data [file pgaf244_supplementary_data.zip › PNASNEXUS-PNASNEXUS-2024-00952RR-s01_20250825.docx]

**Supporting Information for**

***Helicobacter pylori* base-excision restriction enzyme in stomach carcinogenesis**

Masaki Fukuyo^1^, Noriko Takahashi^2^, Katsuhiro Hanada^3^, Ken Ishikawa^4^, Česlovas Venclovas^5^, Koji Yahara^6^, Hideo Yonezawa^3,7^, Takeshi Terabayashi^8^, Yukako Katsura^9^, Naoki Osada^10^, Atsushi Kaneda^1,11^, M. Constanza Camargo^12^, Charles S. Rabkin^12^, Ikuo Uchiyama^13^, Takako Osaki^2^, Ichizo Kobayashi^2, 13, 14, 15, *, +^

1 Department of Molecular Oncology, Graduate School of Medicine, Chiba University, Chiba 260-8670, Japan

2 Department of Infectious Diseases, Kyorin University School of Medicine, Mitaka 181-8611, Tokyo, Japan

3 Clinical Engineering Research Center, Faculty of Medicine, Oita University, Oita, Japan

4 Department of Cell Biology, Institute of Life Science, Kurume University, Kurume, Japan

5 Institute of Biotechnology, Life Sciences Center, Vilnius University, LT-10257 Vilnius, Lithuania

6 Antimicrobial Resistance Research Center, National Institute of Infectious Diseases, Tokyo, Japan

7 Department of Microbiology, Tokyo Dental College, Tokyo 101-0061, Tokyo, Japan

8 Department of Pharmacology, Faculty of Medicine, Oita University, Yufu, Oita, Japan

9 Center for the Evolutionary Origins of Human Behavior, Kyoto University, Inuyama, Japan

10 Graduate School of Information Science and Technology, Hokkaido University, Sapporo, Japan

11 Health and Disease Omics Center, Chiba University, Japan

12 Division of Cancer Epidemiology and Genetics, National Cancer Institute, Maryland 20878, USA

13 Laboratory of Genome Informatics, National Institute for Basic Biology, Okazaki 444-8585, Japan

14 Research Institute for Micro-nano Technology, Hosei University, Koganei 184-0003, Tokyo, Japan

15 Department of Medical Genome Sciences, University of Tokyo, Minato-ku 108-8639, Tokyo, Japan

+ Dedicated to Ira Herskowitz

* Corresponding author, Ichizo Kobayashi

**Email:** [ikobaya@k.u-tokyo.ac.jp](mailto:ikobaya@k.u-tokyo.ac.jp)

**This PDF file includes:**

Supporting text: SI Figures and Tables Index and SI Materials and Methods

Figures S1 to S11

Tables S1 to S9

Legends for Structure S1 to S2 and Datasets S1 to S3

SI References

**Other supporting materials for this manuscript include the following:**

Structure S1 to S2

Datasets S1 to S3

Supporting Information Text

**SI Figures and Tables Index**

**Fig. S1.** Amino acid alignments of *Hp*PabI in *H. pylori* strains.

**Fig. S2.** *H. pylori* subclusters with association between *Hp*PabI and gastric cancer.

**Fig. S3.** Association between *Hp*PabI and gastric cancer with *cagA* type

**Fig. S4.** 3-mer motif characteristics.

**Fig. S5.** *Hp*PabI-mediated chromosomal breakage during *H. pylori* infection.

**Fig. S6.** Counting k-mer motifs.

**Fig. S7.** 5-mer motif characteristics across cancer studies.

**Fig. S8.** Top and bottom ten 4-mer motifs in STAD.

**Fig. S9.** Mutagenesis by PabI from *Pyrococcus abyssi*.

**Fig. S10.** Assessment of *Hp*PabI structure modeling.

**Fig. S11.** Hypothetical *Hp*PabI tetramer on DNA.

**Fig. S12.** Forest plots of the top ten 4-mer motifs in all cancer studies.

**Fig. S13.** Compositional bias of relevant motifs in bacterial genomes.

**Table S1. Association between *Hp*PabI and gastric cancer in *cagA*+ *Hp*GP only *H. pylori* clusters.**

**Table S2.** Genes with a GTAC mutation in gastric cancer genomes.

**Table S3.** Enrichment analyses of genes with a GTAC mutation before filtering of cancer-related genes.

**Table S4.** Enrichment analyses of genes with a GTAC mutation after filtering of cancer-related genes by COSMIC.

**Table S5.** Number of unique mutations of 4-mers on *H. pylori* genome during infection.

**Table S6.** Ranking of 4-mers for mutation frequency on *H. pylori* genome during infection.

**Table S7.** Amino acid sequence with dN/dS of HpPabI.

**Table S8.** Bacteria and restriction enzymes predicted to cause cancer.

**Table S9.** *Hp*PabI nucleotide alignments used for dN/dS calculation.

**SI Materials and Methods**

**Population structure analysis**

***Hp*GP only**. Protein-coding sequences of HpGP genomes were searched using the amino acid sequences of strain 26695 as queries. Only 1315 genes included in the core genome, which were defined using the CoreAligner program (with -ConsRatio = 0.9 and -NbrConsRatio = 0.6), and encoding >100-aa-length- proteins were analyzed. In search for homologous sequences, tblastn (E-value <1e-10 and sequence identity >80%) was used. If matched sequences did not cover 50% of query sequence length, the sequences were filtered out. The nucleotide sequences were aligned for each gene using MAFFT with amino acid sequence as a template[1]. The regions with many alignment gaps were subsequently filtered out; an alignment column that contains gaps in >30% samples were labeled as gapped sites and if the gapped sites represented >4 aligned sites in a 11-bp-length window, all sites in the window were masked for further analyses.

Following the filtering process, alignments containing fewer than 100 aa sites were excluded, resulting in a final set of 1156 genes. Multiple sequence alignments of all the genes were concatenated and converted to PLINK format for genetic structure analysis [2]. For tri-allelic and tetra-allelic SNPs, we decomposed the sites into multiple pseudo sites, which have the same major allele but different minor alleles. Prior to the inference of the genetic structure, samples with >10% missing genotypes, sites with >20% missing genotype, and sites with a minor allele frequency smaller than 1% were filtered out. After the filtering, linked SNPs were pruned using the function implemented in PLINK using the option “--indep-pairwise 20 1 0.5” and missing genotypes were imputed using the most common genotypes among samples. In addition, when several samples showed pi-hat value greater than 0.9, potentially clonal or duplicated samples were removed to leave only one sample. PCA-UMAP feature extraction was performed using the scikit-learn library. In order to define genetic clusters, k-means++ algorithm [3] was applied in the two-dimensional space. The top five principal components were used as input to UMAP. All samples were initially clustered into six global genetic clusters, using the following clustering parameters with the top five PCA components: numbers of components = 2, nearest neighbors = 100, and minimum distance = 0. For each global genetic clusters, samples were further divided into subclusters using the following parameters: number of components = 2, nearest neighbors = 50, and minimum distance = 0.

***Hp*GP + NCBI.** RefSeq Genome Sequences of *Helicobacter pylori* (1710 in all) were downloaded from NCBI on March 10, 2021. Protein-coding sequences were searched against RefSeq genome sequences of *H. pylori* using the amino acid sequences of strain 26695 as queries. As with the HpGP-only dataset, only core genes (n = 1315) and encoding >100-bp amino-acid proteins were used as queries. In search for homologous sequences, tblastn (E-value <1e-10 and sequence identity >80%) was used. If matched sequences did not cover 50% of query sequence length, the sequences were filtered out. The genes containing the data of <2400 strains were filtered out, leaving 1119 genes. The nucleotide sequences were aligned for each gene using MAFFT with amino acid sequence as a template [1]. The regions with many alignment gaps were subsequently filtered out; an alignment column that contains gaps in >30% samples were labeled as gapped sites and, if the gapped sites represented >4 aligned sites in a 11-bp-length window, all sites in the window were masked for further analyses. This dataset was processed and analyzed using the same pipeline as employed for the *Hp*GP data.

**Assessing association of *Hp*PabI gene with gastric cancer**

The patients were diagnosed as carrying cancer by each strain contributor of *Hp*GP Research Network[4]. The *H. pylori* strain and genome sequence data were unlinked from the personal information. The full diagnosis data will be released later in a publication by *Hp*GP. For our study, a BLAST database was constructed using multi-fasta files that encompassed the protein products annotated in the genome assembly of all strains within the *Hp*GP [4]. We then performed a blastp analysis on this database with default parameters, taking the amino acid sequence of the *Hp*PabI from strain HPAG1 (HpyHII) as the query. Strains of *H. pylori* that displayed a blastp hit with a length surpassing 70% and an amino-acid identity greater than 90% were categorized as *Hp*PabI-positive while those that did not meet these specifications were classified as *Hp*PabI-negative. (In instances where the *Hp*PabI sequence appeared fragmented in a strain's genome, we merged these sequences to re-evaluate both the sequence length and identity.) For *cagA* typing, amino acid sequences of *cagA* from strain 26695 and F32 were taken as the query. Then, resulting *cagA* genes were blasted against EPIYA segment amino acid sequence [5] and when a case matched multiple segments, the segment with the highest amino-acid identity was selected. *cagA* containing B and C segments were considered as Western-type, and *cagA* containing B and D segments were considered as East-Asian-type.

We calculated odds ratios, 95% confidence intervals, and p-values for the association between *Hp*PabI and gastric cancer in each group utilizing the “twoby2” function within the “Epi” package in R 3.6.2 without correction for multiple testing. In case where a count in a cell is  zero, we added 1 for small sample correction.

For reference, amino acid sequence alignments for the blastp hits, *Hp*PabI (in strain HPAG1), PabI, and CcoLI are presented in **Fig. S1**. These alignments were generated using default parameters in MUSCLE incorporated within MEGA [6] version 11.0.13.

**Motif analysis with TCGA (The Cancer Genome Atlas) dataset**

Mutation datasets for various cancer studies within TCGA were retrieved from GDAC Firehose (https://gdac.broadinstitute.org/) as Mutation Annotation Format (MAF) files. Studies lacking mutation data were excluded. Single base-pair substitution mutations were exclusively considered.

To construct mutation motifs of 3, 4, and 5-mer base pairs, we extracted sequences from the hg38 genome. These sequences consisted of one 5′-flanking base, the mutated base, and one 3'-flanking base (3-mer); two 5′-flanking bases, the mutated base, and one 3'-flanking base (4-mer); and two 5′-flanking bases, the mutated base, and two 3'-flanking bases (5-mer), respectively. We counted both the top and bottom strands. When the reverse complement of the bottom strand is identical to the top strand, we consolidated them under the lexicographically smaller one in our dictionary arrangement. To obtain extended mutation motifs ranging from 3 to 7 base pairs (i.e., N***N***N = 3-mer, NN***N***, NN***N***N = 4-mer, NNN***N***, NN***N***NN = 5-mer, NNN***N***N, NNNN***N***, NNN***N***NN, NNNN***N***N, NNNNN***N***, NNN***N***NNN, NNNN***N***NN, NNNNN***N***N, and NNNNNN***N***, where ***N*** represents the mutated base). These sequences included various combinations of 5′-flanking bases and 3'-flanking bases, as specified, for each single base-pair substitution mutation. Detailed examples are provided in **Fig. S4**.

To account for sampling bias and differences in mutation load across cancer types, the relative ratio for each motif was calculated using a normalized average motif ratio, obtained by averaging results from 10,000 random oversamplings of SNPs for each of the 28 cancer studies. The relative ratio was defined as:

$relative ratio= \frac{N\left( motif in study \right)/ N(SNP in study)}{N\left( motif in average in oversampling \right)/ N(oversampled SNP)}$,

where N(X) represents the count of X.

In our exploration of varied k-mer motif distribution patterns across different cancer types, we performed a two-way hierarchical clustering analysis. Specifically, we utilized Ward's clustering method along with Manhattan Distance as the metric to gauge dissimilarity between clusters. This analysis was executed on the relative ratios, organized by both motif and cancer type. Our approach leveraged the 'hclust' function, housed within the 'Heatplus' package of R.

We utilized the 'twoby2' function from the 'Epi' package in R to calculate the relative ratios and their corresponding 95% confidence intervals (CIs) for each cancer type. Subsequently, we employed the 'forestplot' package in R to create visual representations of these data as forest plots.

We calculated the compositional bias (Observed/Expected) as an indicator of restriction avoidance, by computing the expected motif frequencies using a maximum-order Markov-chain model [7].

**Analysis of genes with a GT**A**C mutation on gastric cancer genomes**

The mutation list was annotated with COSMIC Cancer Mutation Census (downloaded on Jul 21st, 2020) and mutations at GT***A***C 4-mer motif were filtered. Gene enrichment analysis was performed using DAVID 2021[8] before and after filtering with COSMIC annotations.

**Analysis of 4-mer mutations on *H. pylori* genomes**

Reference genomes and Illumina sequencing reads were obtained from NCBI BioProject (PRJNA490474). Illumina reads were aligned to the reference complete genome, obtained from PacBio sequencing with strains form same patients, using bwa 0.7.17. Pilon 1.24 [9] was used to polish the genome assembly two - nine rounds until output genome reaches equilibrium. The resulting genomes were summarized into multiple fasta files by patients and then, multiple sequence alignment were performed using Kalign 3.4.0. [10]. Initial phylogenetic trees were generated using RAxML 8.2.12 [11] with the “-f a”, “-m GTRGAMMA”, “-o”, and “-N 100” options. Recombination events were predicted using ClonalFrameML 1.12 [12] with default parameters, and the phylogenetic trees were corrected accordingly. Based on these recombination-corrected phylogenies, ancestral genomes were computed by RAxML with “-f A”, “-m GTRGAMMA” options. Mutations were called using GSAlign 1.0.22 [13] with “-sen”, “-slen 10”, and “-one” options.

***H. pylori cag*PAI mutant.**

The mutant *cagPAI* in *H. pylori* strain P12 was generated through double-crossover homologous recombination, introducing a kanamycin (Km) resistance determinant gene. To achieve this, we amplified the upstream and downstream regions of the target genes using specific pairs of primers. For the upstream region, we used primers cagPAIUF (5′-AGATTACAAAGCCTACCAGCA) and cagPAIURagKm (5′-GGGTACCGAGCTCGAATTCAGTGTTACCTCCATAAGGTAT). Similarly, for the downstream region, primers cagPAIDF (5′-GGGGATCCTCTAGAGTCATGACTAACGAAACCATTAACCA) and cagPAIDR (5′-AGCCTTAGAGTCTTTTTGGA) were employed. The flanking regions of these primers were then attached to joint sequence to Km cassette genes. The Km resistance gene cassette (*aph-A3*) was amplified with the specific primer pair (KmF = 5′-GAGCTCGGTACCCGGGTGA, KmR = 5′-GACTCTAGAGGATCCCCG). Subsequently, the upstream, Km, and the downstream amplicons were combined through overlapping extension PCR, with equal amounts of each PCR product. The resulting up-Km-down fragment was introduced into the P12 strain through natural transformation, leading to the creation of a *cag*PAI mutant. The transformants were then spread on Brucella agar (BD Difco) supplemented with 80 mg/ml of kanamycin. The gene disruption was subsequently confirmed using PCR.

**Cell lines and culture.**

The human gastric cancer cell line, AGS, was cultured in RPMI 1640 (Nacalai Tesque) supplemented with 10% fetal bovine serum (FBS) (Cosmobio) at 37°C with 5% CO_2_. Hela cells were cultured in Dulbecco's modified Eagle's medium (DMEM) (Nacalai Tesque) supplemented with 10% FBS at 37°C with 5% CO_2_.

For *H. pylori* strains, including the wild-type P12 strain and its mutants, cultivation was carried out in Brucella broth (BD Difco) with 10% FBS. Incubation was at 37°C within an anaerobic environment using an anaerobic jar with an Anaero Pack (Mitsubishi Gas Chemical).

**Detection of chromosomal double-strand breakage by pulsed-field gel electrophoresis.**

A method, as previously reported in detail [14]^,^ [15], was employed. In brief, AGS cells were initially seeded at approximately 25% confluence within T-25 flasks. These cells were labelled with 10 µM iododeoxyuridine (IdU) (Sigma-Aldrich, Merck) for 48 hours. The cells were then washed with PBS and suspended in fresh medium devoid of IdU. Next, the cells were infected with an *H. pylori* strain at a multiplicity of infection (MOI) of 50 or 100. After 24 h of incubation, the cells were harvested by trypsinization.

To create plugs, a clamped homogeneous electric field (CHEF) disposable plug mould (Bio-Rad) was employed. This involved mixing a cell suspension with a concentration of 2.5 × 10^6^ cells/ml with an equal volume of 1% (w/v) agarose, resulting in a total of 2.5 × 10 ^5^ cells per plug. Subsequently, the plugs were placed in lysis buffer (comprising 100 mM EDTA, 1% [w/v] sodium lauryl sarcosine, 0.2% [w/v] sodium deoxycholate, and 0.5 mg/ml proteinase K) and incubated at 37°C overnight. Pulsed-field gel electrophoresis was conducted at 13°C for 23 hours in 0.9% (w/v) agarose containing 0.25 × Tris-borate-EDTA (TBE) buffer, using a Rotaphor pulsed-field gel electrophoresis system 6.0 (Analytik Jena). The parameters for electrophoresis were set as follows: voltage ranged from 180 to 120 V logarithmically, angle changed from 120° to 110° linearly, time interval decreased from 30 seconds to 5 seconds logarithmically, and no inversion step was included. Gels were subsequently stained with 0.5 μg/ml ethidium bromide (EtBr) and analyzed using a Typhoon FLA7000 scanner (GE Healthcare Life Science).

Following pulsed-field gel electrophoresis (PFGE), the ethidium bromide (EtBr)-stained gel was subjected to UV light exposure at 2,000 J/m² with a wavelength of 258 nm. Subsequently, the gel was treated with denaturation buffer [0.5 N NaOH, 1.5 M NaCl] for a duration of 1 hour, followed by treatment with neutralization buffer [1 M Tris-Cl (pH 7.6), 1.5 M NaCl] for another 1 hour.　Then, DNA was transferred onto Hybond-N+ membrane (GE Healthcare Life Science) using 20X SSC buffer [3.0 M NaCl, 0.3 M trisodium citrate]. Transferred DNAs were crosslinked with UV light at 1,200 J/m^2^. Following this step, the membranes were blocked with a solution consisting of 2.5% skim milk (DB Difco) in 0.1% Tween-20 (Nacalai Tesque) diluted in PBS for 1 hour. The membranes were subsequently incubated with mouse anti-BrdU antibody (BU-44, 1:5,000, BD Biosciences, Inc.) in the blocking buffer for 3 h. Following the antibody incubation, the membranes underwent three washes with 0.1% Tween-20 in PBS. To detect the IdU-labeled DNA, a horse radish peroxidase (HRP)-conjugated donkey anti-mouse antibody (715-035-151, 1:10,000; Jackson Immuno Research) in PBS containing 0.1% Tween-20 was applied to the membranes and incubated overnight. The membranes were then washed three times with 0.1% Tween-20 in PBS. Finally, the signals were visualized using ECL prime solution (Nacalai Tesque) and analyzed with a LAS4000 system (GE Healthcare Life Science).

Semi-quantitative analysis was conducted with ImageQuant software (GE Healthcare Life Science). To determine the amount of broken DNA, the intensity of DNA within the migrated fraction, denoted as 'Broken DNA,' was calculated relative to the intensity of DNA within the well fraction, denoted as 'Intact DNA.' The resulting values were then used to calculate means and standard errors (SE), derived from data gathered in four independent experiments. P-values were determined by Student *t*-test.

**Immunofluorescent staining.**

Immunofluorescent staining was carried out as previously described[15]. In brief, AGS cells were seeded on glass coverslips at approximately 50% confluence and incubated overnight. Subsequently, *H. pylori* strains were allowed to infect these cells. After 6 h and 24 h of incubation, the cells were fixed with 4% paraformaldehyde in PBS (phosphate-buffered saline) for 15 min at 37°C. Then, the cells were permeabilized with 0.1% Triton X-100 in PBS and g-H2AX was detected with mouse monoclonal anti-g-H2AX antibody (JBW301, 1:400, Merck Millipore). As the secondary antibody, AF555-conjugated donkey anti-mouse antibody (20037, 1:400; Biotium) was utilized in PBS containing 0.15% (w/v) glycine and 0.5% (w/v) bovine serum albumin. Nuclear staining was achieved with DAPI (4’,6-diamidino-2-phenylindole) and fluorescent signals were visualized using a Leica TCS SP8 STED confocal microscope. Based on the previous study, cells with more than ten clear and large foci were judged as positive. Tiny spots corresponding to arrested replication forks were excluded from the count.

**Transfection and silencing.**

The cDNA for *Hp*PabI was synthesized (Eurofins genomics) to conform to human codon usage as follows: 5′- ATGTCACTTATTCGCATCGATAACAACAAGAAAGTGATCGAAGTGTCCATTCCCCTTACGAGCATAAGCGGCAAAGTTCGGGTGAAGATAAGACATGCATTCTCCGATTATGGCATTAGTACAGCTACTAGGAAAATCCCTTTCTCTCTGAAACATTACGTAGAGTGGCAGATAGGGTACGATGTTCCGATCAAGGACAAAGAGAAATTCGAGCTGACAACCCTTAAGGACGAGAAGTACCACTTTCTGGGTGCCAATGACAAGGTGAAAACCCTCTACGAACTGTCAGAGATGATCTACTATGCGAAGCAATTGGGACTGATCTCACTGGAGAACCTGGAAAACACGCTCAAGTATCTGGAAAAGCAGAAGCAGTTCATTGAGGACAACTTTATGATCACCCGTGAAAGGTTCCGATCTCACCAGTTTGGTGGGATGGACTTTGAGCTCAGTCGGATATCCTATCCCTTGCTGATTCACAGCTTTGATGACAATCAGCTTAGCGAAATCGTCATTAGAGAGCAGCAGTATGGAAGCAAAACCCAAGCCATGCTGTACTTCTGCTTCTCTATTCTGGAACTGAAAACCGCTACACCACTCCTGAATCGCACTGCAGCATTGAAGGAACATGCCCTGCTCACTATTCACAAGACAAATGCCCTGATGTTCCTCGAGATGCTGAAGATCTTTGGCTTGCTGTCCCAAGCTCATCACAACGATGTCCTCAAGATCCTGGAGAAAATCCTGCAGAATTGA-3’. The synthesized sequence was inserted between the BamHI and EcoRI sites in pCMV-Tag 2B (Stratagene, Agilent Technologies). This expression vector incorporates a FLAG (DDDDK)-tagged protein and features the cytomegalovirus immediate-early enhancer/promoter.

For transfection experiments, Hela cells were cultured in 6-well plates until they reached 40-50% confluence. APE1 siRNA (On-Target Plus human APEX1 siRNA, L-010237-00-0005, Dharmacon) was transfected into the cells at a concentration of 100 pmol using Lipofectamine RNAi Max reagent (Life Technologies) following the manufacturer's instructions. After 48 h, the cells were transfected with 2 mg of plasmid, pCMV-Tag 2B or pCMV-Tag 2B-*Hp*PabI. After 24 h, the cells were harvested and analyzed by pulsed-field gel electrophoresis or Western blot, as described earlier. The error bars represent standard errors (SEs) calculated from data obtained in four independent experiments. The *p*-values were determined by Student’s *t*-test.

**Mutation testers.**

Due to the high cost and time required for human studies, we utilized a simplified *E. coli* system for analysis. We used the *rpsL* system to test for mutations. The *rpsL* gene in *E. coli* encodes the ribosomal protein S12, which serves as the binding site for streptomycin. Specific mutations in *rpsL* confer resistance to this antibiotic. Since the streptomycin resistance allele (Str^R^) is recessive to the wild-type allele (Str^S^), Str^R^ cells carrying an additional wild-type *rpsL* gene exhibit streptomycin sensitivity. However, if a mutation disrupts the function of the wild-type *rpsL*, the cells become resistant to streptomycin. We acquired *E. coli* T7 Express lysY/Iq [= MiniF *lysY lacIq* (Cam^R^) / *fhuA2 lacZ::T7 gene1 [lon] ompT gal sulA11* Δ(*mcr-73::miniTn10--TetS*)2 [dcm] Δ(*zgb-210::Tn10--TetS*) *endA1* Δ(mcrC-mrr) *114::IS10*] from New England Biolabs. The *rpsL128* gene (Str^R^ mutant allele) from the ribosomal protein gene cluster of MK9544 [16] was transferred to this strain using P1 transduction. To the resulting strain, the ectopic *rpsL*^+^ gene (Str^S^ wild-type) and linked kan (Kan^R^) gene from MK9528 [16] were also transferred using P1 transduction. The *kan* gene was then removed by FLP site-specific recombinase encoded by pCP20 [17], kindly provided by Hirotada Mori.

We introduced pBAD30-cviQIM[18] into the above strain by electroporation. This plasmid carries a Chlorella virus DNA methyltransferase gene recognizing 5’-GTAC in a form inducible with arabinose. We then introduced either pYF46 = pET28a::hpyAXIIR (hpyAXIIR = *Hp*PabI) 19 or its empty version into this strain via electroporation in the presence of arabinose.

We constructed a pKIK5 by inserting *Hp*PabI gene into a protein expression vector, pEU3-NII (TOYOBO). This vector features a T7 promoter and omega sequence, a plant translational signal, instead of a bacterial Shine-Dalgarno sequence and was made as follows. The coding region DNA of *Hp*PabI of HPAG1 was synthesized and inserted into a cloning vector pEX-A2J2 at its EcoRV and BamHI sites by Eurofins Scientific SE to generate pEX-A2J2-R.HpyAXII_HPAG1. We amplified its *Hp*PabI-coding region using PCR with primers KI1711 (ATCATGAGTTTGATTAGGATTGATAAT) and KI1712 (CGGGATCCTTAATTTTGAAGTATTTTTTCT) and the PrimeSTAR DNA polymerase (Takara Bio), which produces blunt-ended products. This DNA fragment was cleaved at its 3’-end with BamHI and ligated to EcoRV (blunt end) and BamHI sites of pEU3-NII to generate pKIK5. We confirmed the DNA sequences of its T7 promoter, omega, and HpPabI gene by Sanger sequencing.

**Measuring mutant frequency.**

**A. Strains with a cognate DNA methyltransferase.** The bacterial strains were streaked on an LB agar (Difco) plate with 50 µg/ml Amp, 50 µg/ml Kan and 0.5% arabinose and then incubated overnight at 37ºC. An isolated colony was inoculated into 5 ml LB broth with the same additives and incubated overnight at 37ºC with shaking at 120 rpm. The culture was then diluted 1:100 into 10 ml of the same medium and shaken at 120 rpm in a water bath until its OD600 reached approximately 0.2. The culture was centrifuged at 7,000 rpm for 10 minutes at 4ºC. Then, the supernatant was discarded and the cells were resuspended in the same medium with Amp and Kan. The suspension was divided into tubes (5 ml each), to which arabinose (to 0.5%) and/or IPTG (to 0.5 mM) were added. The tubes were then shaken at 120 rpm in a water bath at 37ºC.

After 18-20 hours, each culture was serially diluted and spread on two types of LB agar plates: plate A (with 50 µg/ml Amp, 50 µg/ml Kan, and 0.5% arabinose) and plate B (with the same additives as plate A plus 100 µg/ml streptomycin). The plates were incubated overnight at 37ºC. Colonies were counted to calculate the concentration of colony formers on plate B (representing Str^R^ mutant cells) and on plate A (representing viable cells).  The mutant frequency was determined by dividing the former by the latter.

**B. Strains without a cognate DNA methyltransferase.** The bacterial strains were streaked on an LB agar plate supplemented with 75 µg/ml Amp and incubated overnight at 37ºC. Each isolated colony was picked and inoculated into LB broth with 75 µg/ml Amp and then the broth was shaken at 130 rpm in a water bath at 37ºC overnight. The culture was diluted 1:100 in the same medium and shaken at 37ºC until its OD600 reached approximately 0.2. The culture was then diluted 1 to 10^5^ in the same medium, resulting in approximately 10^3^ cells/ml (5 ml per tube). Each culture was serially diluted and spread onto two types of LB agar plates: plate A (with 75 µg/ml Amp) and plate B (with 75 µg/ml Amp and 100 µg/ml Str). The plates were incubated overnight at 37ºC. Colonies were counted to calculate the concentration of colony formers on plate B (representing Str^R^ mutant cells) and on plate A (representing viable cells). The mutant frequency was determined by dividing the former by the latter. In experiments to examine the effects *Hp*PabI induction, IPTG was either added to a final concentration of 0.5 mM or not added to the culture with approximately 10^3^ cells/ml.

**Structural modelling and analysis.**

A dimeric structure model of *Hp*PabI from the *H. pylori* strain HPAG1 was generated using AlphaFold-Multimer v.2 [19]. The model’s quality was evaluated both with AlphaFold’s self-estimates and VoroMQA [20]. The *Hp*PabI model was compared to PDB structures using Dali [21] or MM-align [22].  A putative *Hp*PabI-DNA complex was constructed by first superimposing the *Hp*PabI model onto the structure of the PabI-DNA complex and then deleting the PabI protein. A hypothetical *Hp*PabI-DNA complex was assembled by superimposing the *Hp*PabI model onto the PabI-DNA complex structure, followed by removal of the PabI protein. In other words, DNA from the PabI-DNA complex was copied to the *Hp*PabI model. UCSF Chimera [23] was used for structure visualisation and analysis.

**dN/dS calculation**

Using the amino acid sequences of HPAG1 *Hp*PabI (Hypothetical protein HPAG1_0479: ABF84546.1) as a query, homologous protein-coding sequences of *H. pylori* were searched against RefSeq genome sequences of *H. pylori* (2250 sequences, downloaded on June 23^rd^, 2023). The homologous sequences were identified using tblastn (e-value < 1e-10 and sequence identity > 80%) [24]. Sequences that did not cover 90% of the query sequence length were excluded, resulting in 553 sequences. The nucleotide sequences were aligned using MAFFT with the amino acid sequence as a template [1]. Regions with numerous alignment gaps were filtered out as follows: an alignment column with a gap in >50% of the samples was labelled as a gapped site and, if these gapped sites accounted for >3 aligned sites in a 6-bp window, all the sites in that window were masked in the subsequent analyses.

The dN/dS value of for each codon was estimated using genomegaMap [25] (**Table S7**). We used the independent codon model with an equal frequency of codons. The mean of the prior distribution for theta was set to 0.2. For the *Hp*GP gene, 4,000,000 MCMC iterations, including 200,000 burn-in steps, were conducted. We used the default parameters unless stated otherwise. The names of the 553 strains used are listed in **Datasets S3** while the nucleotide alignments can be found in **Table S9**.

Structure S1 (separate file). A model of *Hp*PabI dimer.

Structure S2 (separate file). A model of HPAG1 dimer with DNA.

Datasets S1 (separate file). (A) Clustering *Hp*GP strains. (B) Clustering “*Hp*GP + NCBI” strains. (C) Collection places in some relevant subgroups (*Hp*GP). (D) Collection places in some relevant subgroups (*Hp*GP + NCBI).

Datasets S2 (separate file). Top 10 motifs for each cancer type with top 10 genera. For each motif, the relevant R and M genes in REBASE and the hits in REBASE Pacbio were counted to give the numbers in all column. The top 10 ranking genera for this number are listed.

Datasets S3 (separate file). Open *H. pylori* genomes used for dN/dS calculation.

**SI References**

1. Katoh K, Kuma K-I, Toh H, Miyata T (2005) MAFFT version 5: improvement in accuracy of multiple sequence alignment. Nucleic Acids Res 33:511–518

2. Purcell S, Neale B, Todd-Brown K, et al (2007) PLINK: a tool set for whole-genome association and population-based linkage analyses. Am J Hum Genet 81:559–575

3. David Arthur SV (2007) k-means++: the advantages of careful seeding. In: SODA ’07: Proceedings of the eighteenth annual ACM-SIAM symposium on Discrete algorithms. pp 1027–1035

4. Thorell K, Muñoz-Ramírez ZY, Wang D, et al (2023) The Helicobacter pylori Genome Project: insights into H. pylori population structure from analysis of a worldwide collection of complete genomes. Nat Commun 14:1–16

5 Yamaoka Y (2010) Mechanisms of disease: Helicobacter pylori virulence factors. Nat Rev Gastroenterol Hepatol 7:629-641

6. Kumar S, Stecher G, Li M, et al (2018) MEGA X: Molecular Evolutionary Genetics Analysis across Computing Platforms. Mol Biol Evol 35:1547–1549

7. Rusinov IS, Ershova AS, Karyagina AS, et al (2018) Comparison of Methods of Detection of Exceptional Sequences in Prokaryotic Genomes. Biochemistry 83:129–139

8. Huang DW, Sherman BT, Lempicki RA (2009) Systematic and integrative analysis of large gene lists using DAVID bioinformatics resources. Nat Protoc 4:44–57

9. Walker BJ, Abeel T, Shea T, et al (2014) Pilon: an integrated tool for comprehensive microbial variant detection and genome assembly improvement. PLoS One 9:e112963

10. Lassmann T (2019) Kalign 3: multiple sequence alignment of large datasets. Bioinformatics 36:1928–1929

11. Stamatakis A (2015) Using RAxML to Infer Phylogenies. Curr Protoc Bioinformatics 51:6.14.1-6.14.14

12. Didelot X, Wilson DJ (2015) ClonalFrameML: efficient inference of recombination in whole bacterial genomes. PLoS Comput Biol 11:e1004041

13. Lin H-N, Hsu W-L (2020) GSAlign: an efficient sequence alignment tool for intra-species genomes. BMC Genomics 21:182

14. Kawashima Y, Yamaguchi N, Teshima R, et al (2017) Detection of DNA double-strand breaks by pulsed-field gel electrophoresis. Genes Cells 22:84–93

15. Teshima R, Hanada K, Akada J, et al (2018) Aggregatibacter actinomycetemcomitans infection causes DNA double-strand breaks in host cells. Genes Cells 23:264–273

16. Le LAT, Chang PY, Ando S, et al (2020) Nutritional conditions and oxygen concentration affect spontaneous occurrence of homologous recombination events but not spontaneous mutagenesis in Escherichia coli. Genes Genet Syst 95:85–93

17. Cherepanov PP, Wackernagel W (1995) Gene disruption in *Escherichia coli*: TcR and KmR cassettes with the option of Flp-catalyzed excision of the antibiotic-resistance determinant. Gene 158:9–14

18. Fukuyo M, Nakano T, Zhang Y, et al (2015) Restriction-modification system with methyl-inhibited base excision and abasic-site cleavage activities. Nucleic Acids Res 43:2841–2852

19. Evans R, O’Neill M, Pritzel A, et al (2022) Protein complex prediction with AlphaFold-Multimer. bioRxiv 2021.10.04.463034

20. Olechnovič K, Venclovas Č (2019) VoroMQA web server for assessing three-dimensional structures of proteins and protein complexes. Nucleic Acids Res 47:W437–W442

21. Holm L (2022) Dali server: structural unification of protein families. Nucleic Acids Res 50:W210–W215

22. Mukherjee S, Zhang Y (2009) MM-align: a quick algorithm for aligning multiple-chain protein complex structures using iterative dynamic programming. Nucleic Acids Res 37:e83

23. Pettersen EF, Goddard TD, Huang CC, et al (2004) UCSF Chimera--a visualization system for exploratory research and analysis. J Comput Chem 25:1605–1612

24. Altschul SF, Madden TL, Schäffer AA, et al (1997) Gapped BLAST and PSI-BLAST: a new generation of protein database search programs. Nucleic Acids Res 25:3389–3402

25. Wilson DJ, CRyPTIC Consortium (2020) GenomegaMap: Within-Species Genome-Wide dN/dS Estimation from over 10,000 Genomes. Mol Biol Evol 37:2450–2460

26. Chen Y, Wu F-H, Wu P-Q, et al (2022) The Role of The Tumor Microbiome in Tumor Development and Its Treatment. Front Immunol 13:935846

27. Inamura K, Hamada T, Bullman S, et al (2022) Cancer as microenvironmental, systemic and environmental diseases: opportunity for transdisciplinary microbiomics science. Gut. https://doi.org/10.1136/gutjnl-2022-327209

28. Mima K, Kosumi K, Baba Y, et al (2021) The microbiome, genetics, and gastrointestinal neoplasms: the evolving field of molecular pathological epidemiology to analyze the tumor-immune-microbiome interaction. Hum Genet 140:725–746

29. Anipindi M, Bitetto D (2022) Diagnostic and Therapeutic Uses of the Microbiome in the Field of Oncology. Cureus 14:e24890

30. Kandalai S, Li H, Zhang N, et al (2023) The human microbiome and cancer: a diagnostic and therapeutic perspective. Cancer Biol Ther 24:2240084

31. Zhou J, Sun S, Luan S, et al (2021) Gut Microbiota for Esophageal Cancer: Role in Carcinogenesis and Clinical Implications. Frontiers in Oncology 11:717242

32. La Rosa GRM, Gattuso G, Pedullà E, et al (2020) Association of oral dysbiosis with oral cancer development. Oncol Lett 19:3045–3058

33. Piao X-M, Byun YJ, Zheng C-M, et al (2023) A New Treatment Landscape for RCC: Association of the Human Microbiome with Improved Outcomes in RCC. Cancers 15:935

34. Mahmood R, Voisin A, Olof H, et al (2023) Host Microbiomes Influence the Effects of Diet on Inflammation and Cancer. Cancers 15:521

35. Sipos A, Ujlaki G, Mikó E, et al (2021) The role of the microbiome in ovarian cancer: mechanistic insights into oncobiosis and to bacterial metabolite signaling. Mol Med 27:33

36. Wahid M, Dar SA, Jawed A, et al (2022) Microbes in gynecologic cancers: Causes or consequences and therapeutic potential. Semin Cancer Biol 86:1179–1189

37. Crocetto F, Boccellino M, Barone B, et al (2020) The Crosstalk between Prostate Cancer and Microbiota Inflammation: Nutraceutical Products Are Useful to Balance This Interplay? Nutrients 12:2648
