# Supplementary material for: Helicobacter pylori base-excision restriction enzyme in stomach carcinogenesis
Source: PNAS Nexus. 2025 Aug 5;4(8):pgaf244. doi: 10.1093/pnasnexus/pgaf244 (PMC12366791; doi:10.1093/pnasnexus/pgaf244)

Fig. S1. Amino acid alignments of *Hp*PabI in *H. pylori* strains.

A total of 1,191 blastp hits were identified from 918 strains using *Hp*PabI (strain HPAG1) as the query sequence. The alignment viewer displays blastp hits, *Hp*PabI, PabI, and CcoLI. Among these hits, sequences with a length exceeding 70% of *Hp*PabI and an amino acid identity greater than 90% were classified as *Hp*PabI-positive.

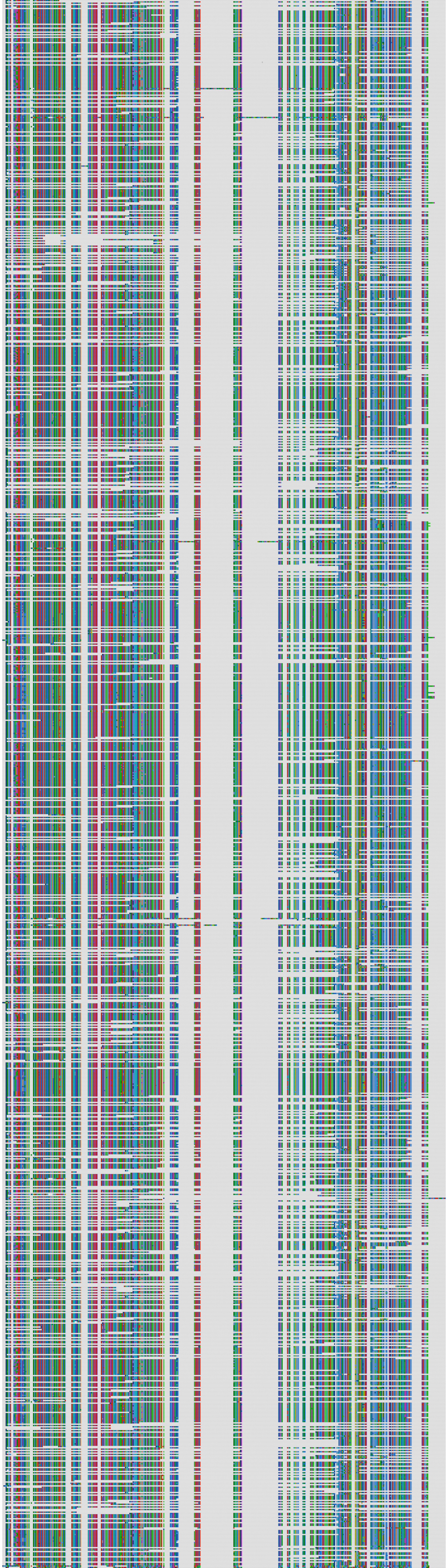

Supplement: pgaf244_Supplementary_Data [file pgaf244_supplementary_data.zip › PNASNEXUS-PNASNEXUS-2024-00952RR-s02.pdf]
