# Supplementary material for: Helicobacter pylori base-excision restriction enzyme in stomach carcinogenesis
Source: PNAS Nexus. 2025 Aug 5;4(8):pgaf244. doi: 10.1093/pnasnexus/pgaf244 (PMC12366791; doi:10.1093/pnasnexus/pgaf244)
Supplement: pgaf244_Supplementary_Data [file pgaf244_supplementary_data.zip › PNASNEXUS-PNASNEXUS-2024-00952RR-s03.pdf]

Fig. S2

(A) *Hp*GP only EU

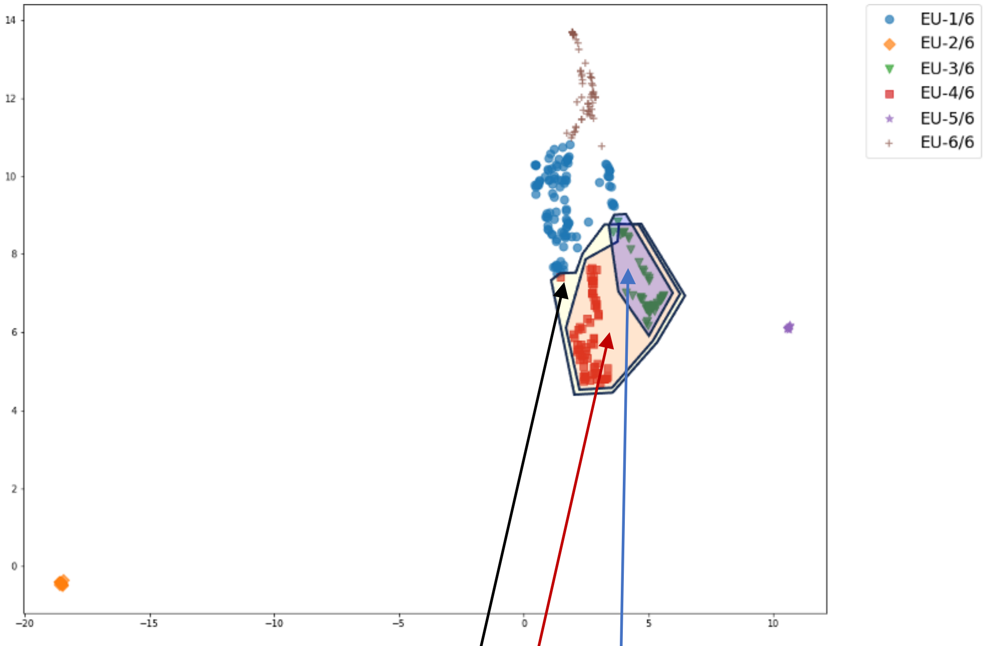

group	OR(cagA+/-)	OR(cagA+)	Country
EU	2.0	-	-
EU-1_3	3.1	3.7	Latvia, Sweden, Lithuania, Iceland
EU-3_5	3.2	3.8	Latvia, Sweden, Lithuania
EU-3_6	-	9.4	Sweden

(B) *Hp*GP only LA

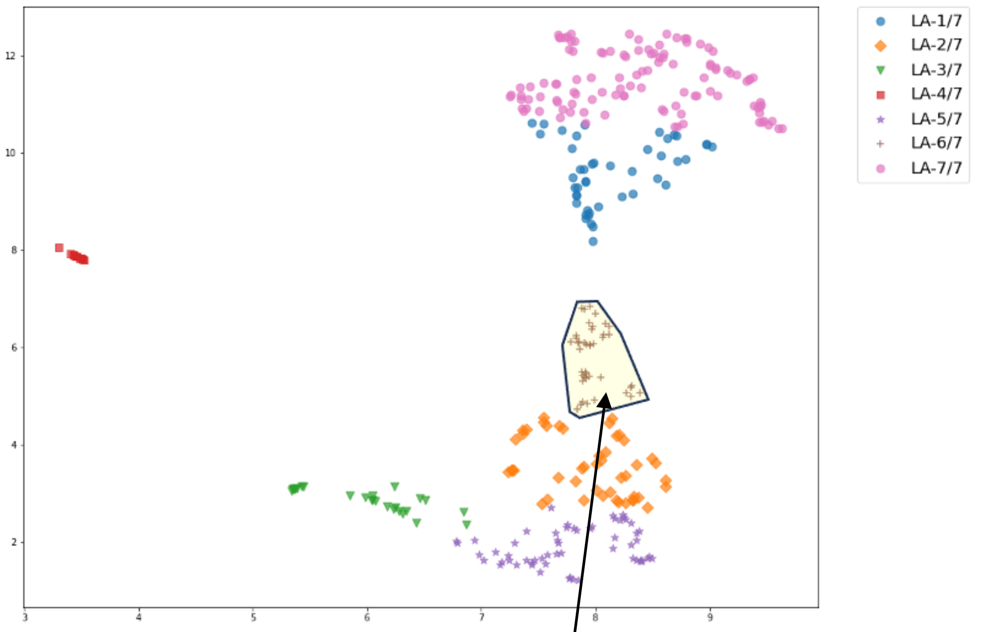

group	OR(cagA+/-)	OR(cagA+)	Country
LA-6_7	-	9.9	Spain

Fig. S2

(C) *Hp*GP +NCBI
EU

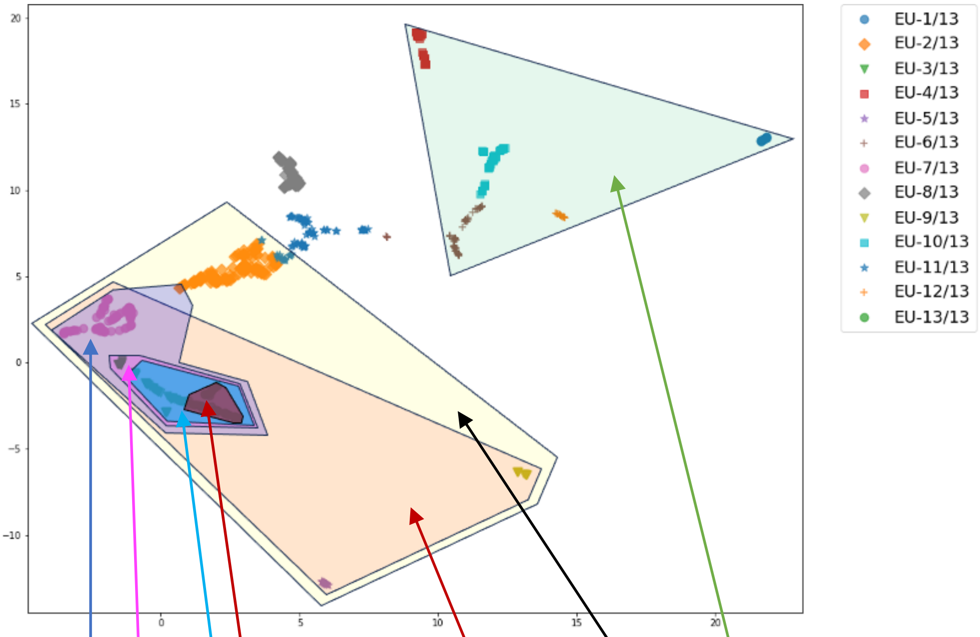

group	OR(cagA+/-)	OR(cagA+)	Country
EU	2.5	2.6	
EU-1/2	2.0	2.0	Australia, Sweden, Switzerland, Latvia, Germany, Lithuania, France, Poland, Italy, Icekabd, Russia
EU-1/3	2.4	2.6	Sweden, Australia, Latvia, Germany, Lithuania, Iceland, Switzerland
EU-2/3	9.9	-	Malaysia, India, Cambodia, Bangladesh, Australia, USA, Myanmar
EU-1/4	2.4	2.5	Sweden, Australia, Latvia, Germany, Lithuania, Switzerland
EU-5/7	5.0	7.2	Sweden, Australia
EU-7/8	4.8	7.4	Sweden, Australia
EU-13/13	11	13	Sweden, Australia

Fig. S2. *H. pylori* subclusters with association between *Hp*PabI and gastric cancer.

(A) *Hp*GP genomes of the EU cluster divided into 6 subclusters (**Table S1A**). Subclusters EU-1_3 (the 1st subcluster in the EU cluster divided into 3), EU-3_5 and EU-3_6 highlighted with colour shades display association with high odds ratios (OR). Some of the countries of strain collection are shown (**Table S1C**).

(B) *Hp*GP genomes of the LA cluster divided into 11 subclusters (**Table S1A**). LA-6_7 shows a strong association with a high OR.

(C) "*Hp*GP + NCBI" genomes of the EU cluster divided into 7 subclusters (**Table S1B**).
