# Supplementary material for: Helicobacter pylori base-excision restriction enzyme in stomach carcinogenesis
Source: PNAS Nexus. 2025 Aug 5;4(8):pgaf244. doi: 10.1093/pnasnexus/pgaf244 (PMC12366791; doi:10.1093/pnasnexus/pgaf244)
Supplement: pgaf244_Supplementary_Data [file pgaf244_supplementary_data.zip › PNASNEXUS-PNASNEXUS-2024-00952RR-s05.pdf]

Fig. S4

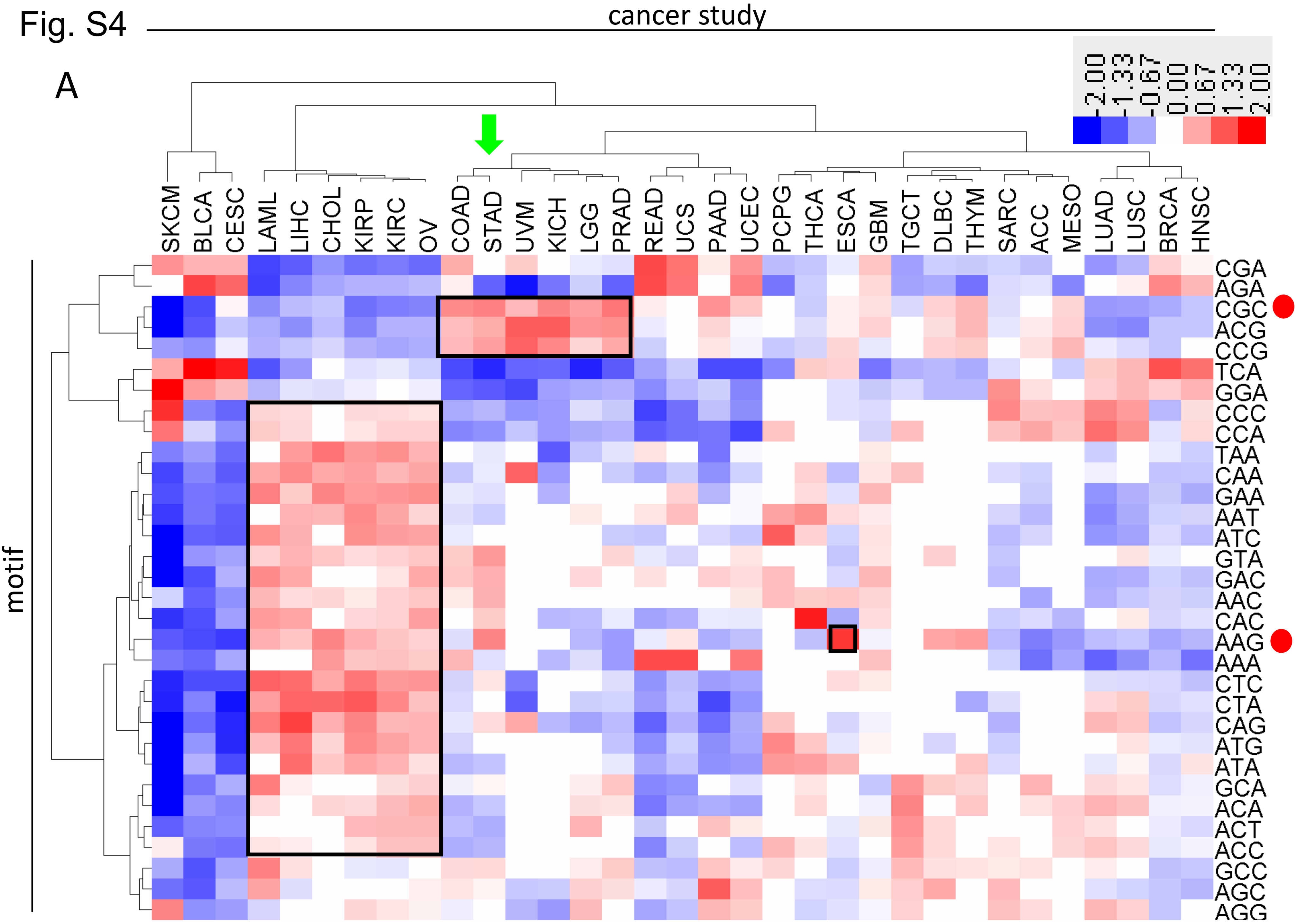

B

motif	number of motif in STAD	relative ratio	(95% CI)
CGC	25276	1.96	(1.93 to 2.00)
AAG	8183	1.95	(1.89 to 2.01)
GTA	3785	1.74	(1.66 to 1.82)
CCG	17540	1.67	(1.63 to 1.70)
ACG	17526	1.57	(1.54 to 1.61)
GAC	3512	1.53	(1.46 to 1.60)
AAC	3828	1.52	(1.45 to 1.58)
CAC	3792	1.32	(1.27 to 1.38)
GCC	9141	1.24	(1.20 to 1.27)
CAG	5431	1.20	(1.16 to 1.25)
CTC	3183	1.14	(1.09 to 1.19)
AGC	6886	1.06	(1.03 to 1.09)
GCA	5919	1.05	(1.02 to 1.08)
ATG	3266	1.03	(0.98 to 1.07)
CGA	11118	1.02	(0.99 to 1.04)
ATC	2385	1.00	(0.95 to 1.05)
AGG	7504	0.97	(0.94 to 1.00)
CTA	1454	0.96	(0.90 to 1.02)
CAA	1970	0.90	(0.85 to 0.95)
TAA	1458	0.89	(0.83 to 0.94)
AAA	2944	0.85	(0.82 to 0.89)
GAA	2143	0.85	(0.81 to 0.89)
AAT	2323	0.77	(0.73 to 0.81)
ACA	3361	0.75	(0.72 to 0.78)
ATA	1817	0.73	(0.69 to 0.77)
CCC	4339	0.68	(0.65 to 0.70)
ACC	3087	0.67	(0.64 to 0.70)
ACT	2466	0.65	(0.62 to 0.68)
CCA	3387	0.55	(0.53 to 0.57)
AGA	5924	0.43	(0.42 to 0.44)
GGA	4057	0.41	(0.39 to 0.42)
TCA	3548	0.31	(0.30 to 0.32)

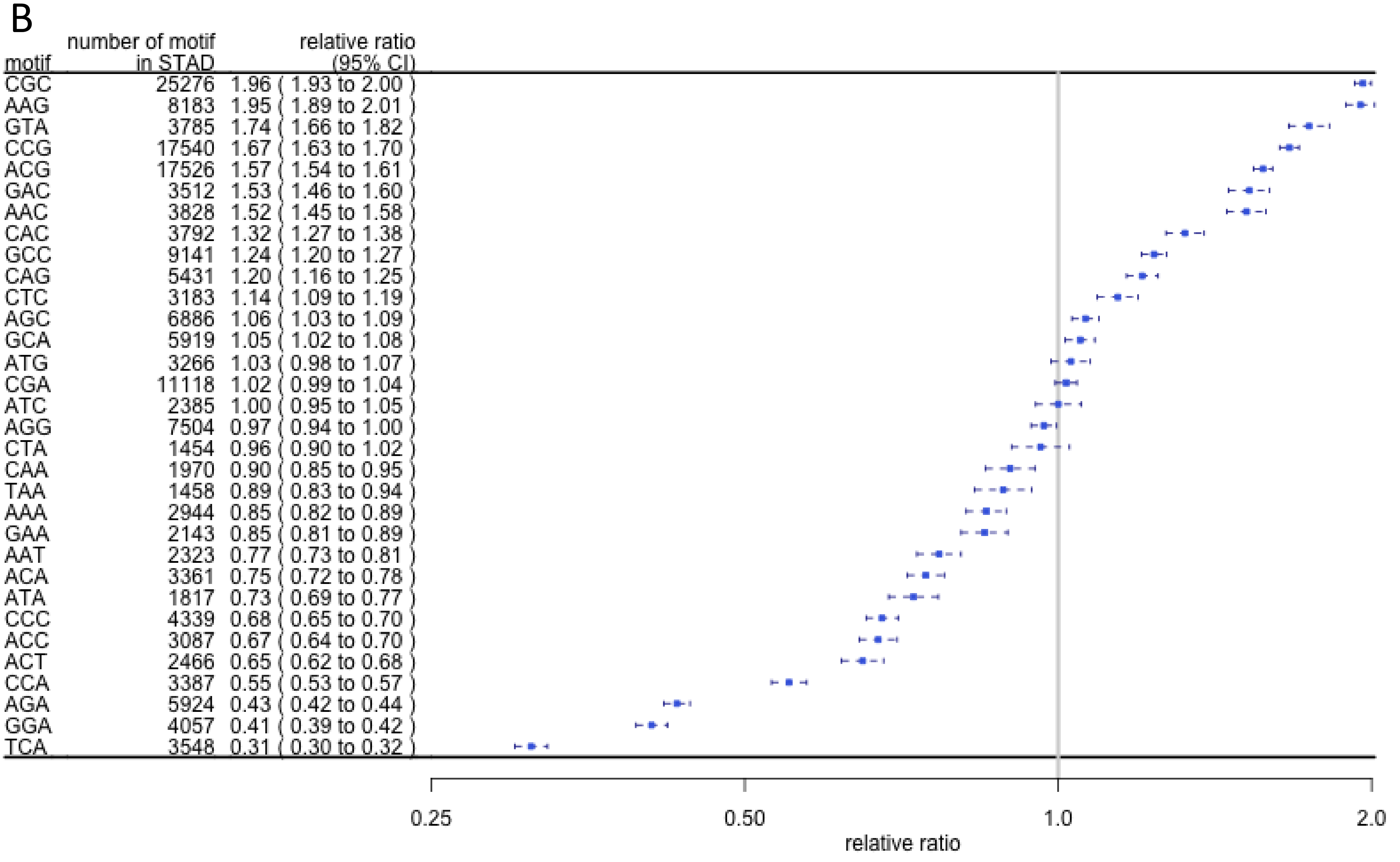

C

motif	number of CGC	relative ratio (95% CI)
ACC	719	1.02 (0.94 to 1.10)
BLCA	3531	0.38 (0.37 to 0.40)
BRCA	4980	0.63 (0.61 to 0.65)
CESC	7161	1.04 (1.01 to 1.07)
CHOL	246	0.74 (0.65 to 0.85)
COAD	30388	1.87 (1.84 to 1.90)
DLBC	555	1.28 (1.17 to 1.40)
ESCA	3151	1.09 (1.05 to 1.13)
GBM	6633	1.19 (1.15 to 1.22)
HNSC	4988	0.73 (0.71 to 0.75)
KICH	369	1.93 (1.73 to 2.16)
KIRC	763	0.50 (0.46 to 0.53)
KIRP	673	0.46 (0.43 to 0.50)
LAML	347	0.54 (0.49 to 0.61)
LGG	3924	1.68 (1.62 to 1.74)
LIHC	2490	0.69 (0.67 to 0.72)
LUAD	8040	0.57 (0.56 to 0.59)
LUSC	7244	0.59 (0.57 to 0.61)
MESO	306	1.24 (1.10 to 1.40)
OV	2392	0.51 (0.49 to 0.54)
PAAD	3743	1.81 (1.75 to 1.88)
PCPG	137	0.87 (0.73 to 1.03)
PRAD	4062	2.11 (2.03 to 2.18)
READ	4881	1.11 (1.07 to 1.14)
SARC	1563	0.84 (0.80 to 0.89)
SKCM	1721	0.06 (0.06 to 0.07)
STAD	25276	1.96 (1.93 to 2.00)
TGCT	169	0.82 (0.70 to 0.96)
THCA	398	0.76 (0.68 to 0.84)
THYM	401	1.47 (1.32 to 1.63)
UCEC	77961	1.34 (1.32 to 1.36)
UCS	653	0.93 (0.86 to 1.01)
UVM	177	1.44 (1.24 to 1.68)

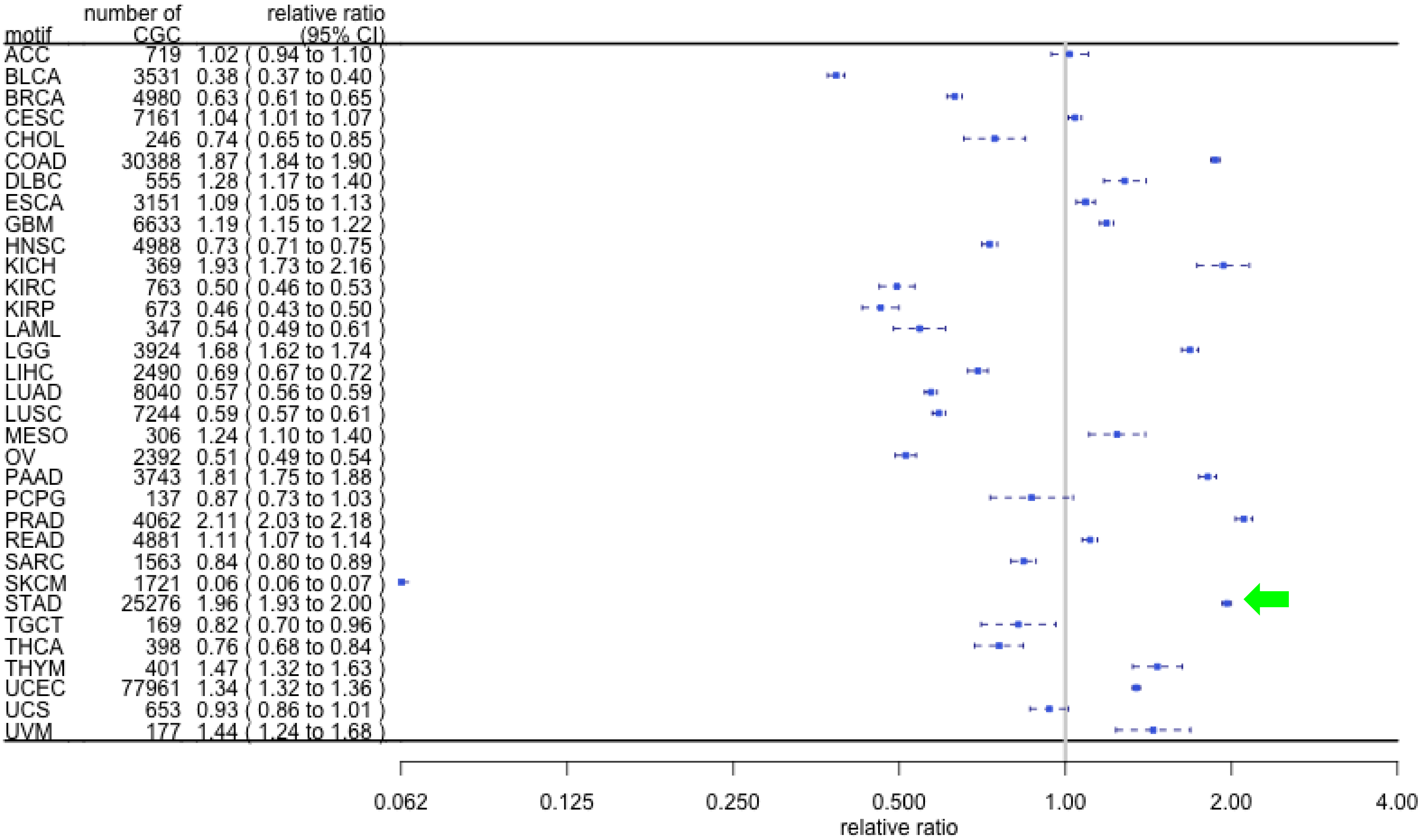

D

motif	number of AAG	relative ratio (95% CI)
ACC	115	0.50 (0.42 to 0.60)
BLCA	1132	0.38 (0.36 to 0.40)
BRCA	1597	0.62 (0.59 to 0.65)
CESC	774	0.35 (0.32 to 0.37)
CHOL	208	1.93 (1.68 to 2.23)
COAD	4452	0.84 (0.81 to 0.87)
DLBC	230	1.63 (1.43 to 1.86)
ESCA	2796	2.97 (2.84 to 3.10)
GBM	1710	0.94 (0.89 to 0.99)
HNSC	1501	0.67 (0.64 to 0.71)
KICH	59	0.95 (0.73 to 1.23)
KIRC	682	1.36 (1.26 to 1.47)
KIRP	726	1.53 (1.42 to 1.66)
LAML	317	1.53 (1.36 to 1.71)
LGG	518	0.68 (0.62 to 0.75)
LIHC	1594	1.37 (1.29 to 1.44)
LUAD	3231	0.70 (0.68 to 0.73)
LUSC	2978	0.74 (0.71 to 0.78)
MESO	46	0.57 (0.43 to 0.77)
OV	1935	1.28 (1.21 to 1.34)
PAAD	435	0.65 (0.59 to 0.71)
PCPG	50	0.97 (0.74 to 1.29)
PRAD	368	0.59 (0.53 to 0.65)
READ	1291	0.90 (0.85 to 0.96)
SARC	420	0.69 (0.63 to 0.77)
SKCM	3656	0.41 (0.39 to 0.42)
STAD	8183	1.95 (1.89 to 2.01)
TGCT	57	0.85 (0.65 to 1.11)
THCA	123	0.72 (0.60 to 0.86)
THYM	152	1.71 (1.45 to 2.01)
UCEC	13502	0.71 (0.69 to 0.74)
UCS	260	1.14 (1.01 to 1.30)
UVM	31	0.78 (0.54 to 1.11)

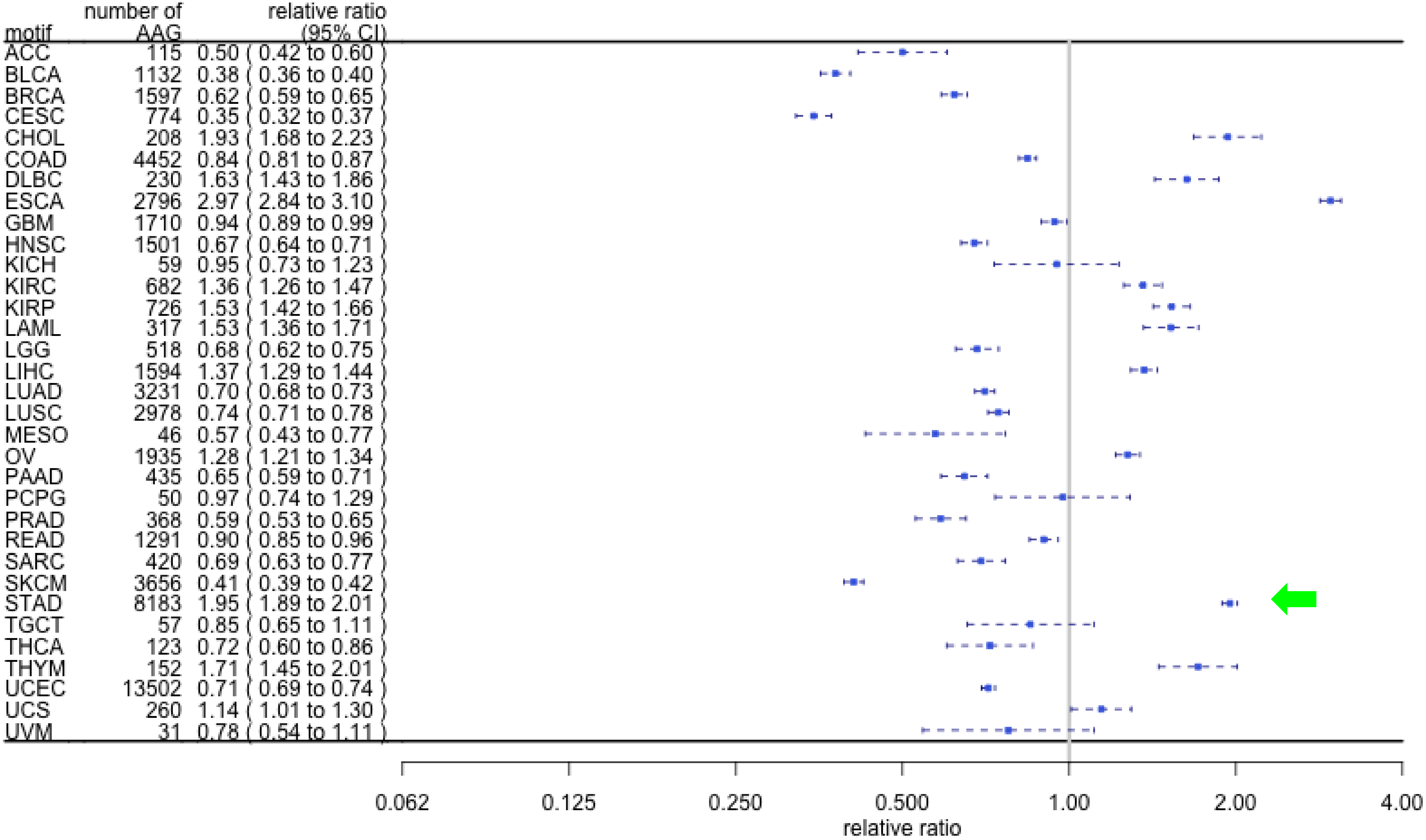

Fig. S4. 3-mer motif characteristics.

(A) Two-way hierarchical clustering of the log2 relative ratio for all motifs across cancer types in TCGA dataset. Ratios without significant difference (where 95% CI intersects hazard ratio = 1.0) are masked as 0. The rectangles indicate a cluster involving STAD and CGC and a larger cluster involving STAD and AAG. The CGC motif, together with ACG (reverse complement of CGT) and CCG (reverse complement of CGG), formed a cluster with high relative ratio, encompassing colorectal adenocarcinoma (COAD), stomach adenocarcinoma (STAD), uveal melanoma (UVM), kidney chromophobe (KICH), low grade glioma (LGG), and prostate adenocarcinoma (PRAD). The AAG motif appeared in a separate cluster and a separate island with esophageal carcinoma (ESCA).

Forest plots of the relative ratio for **(B)** all motifs in STAD, **(C)** CGC motifs in all cancer studies, and **(D)** AAG motifs in all cancer studies. Relative ratio is represented by a blue dot and the 95% CI by whiskers.
